# Single-cell RNA sequencing to detect age-associated genes that identify senescent cells in the liver of aged mice

**DOI:** 10.1038/s41598-023-41352-6

**Published:** 2023-08-30

**Authors:** Yuta Doshida, Shinichi Hashimoto, Sadahiro Iwabuchi, Yuka Takino, Toshiyuki Ishiwata, Toshiro Aigaki, Akihito Ishigami

**Affiliations:** 1 Molecular Regulation of Aging, Tokyo Metropolitan Institute for Geriatrics and Gerontology, 35-2 Sakae-cho, Itabashi-ku, Tokyo, 173-0015 Japan; 2 Department of Biological Sciences, Tokyo Metropolitan University, Tokyo, 192-0397 Japan; 3 Department of Molecular Pathophysiology, Institute of Advanced Medicine, Wakayama Medical University, Wakayama, 641-8509 Japan; 4 Aging and Carcinogenesis, Tokyo Metropolitan Institute for Geriatrics and Gerontology, Tokyo, 173-0015 Japan

**Keywords:** Biomarkers, Geriatrics

## Abstract

Senescent cells are predicted to occur and increase in animal tissues with aging. However, senescent cells in the tissues of aged animals remain to be identified. We refer to the marker genes to identify senescent cells in tissues as “age-associated genes”. In this study, we searched for age-associated genes to identify senescent cells in the livers of aged animals. We performed single-cell RNA sequencing (scRNA-seq) to screen candidates for age-associated genes using young and aged rat primary hepatocytes. To remove animal species specificity, gene expression analyses in mouse livers were performed, confirming age-associated increases in the mRNA expression levels of *Glipr1*, *Clec12a*, and *Phlda3*. Moreover, the mRNA expression levels of *Glipr1* and *Phlda3* were increased by stress-induced premature senescence using doxorubicin in primary hepatocytes and livers of young mice. Transcriptome data of aged rat hepatocytes suggested that *Glipr1*, *Clec12a*, and *Phlda3* were expressed in almost identical cells. Fluorescence in situ hybridization (FISH) confirmed the presence of cells with abundant *Glipr1*, *Clec12a*, and *Phlda3* mRNA in 27-month-old mouse primary hepatocytes, which are considered to be senescent cells. This study is the first to identify *Glipr1*, *Clec12a*, and *Phlda3* as age-associated genes in the mouse liver.

## Introduction

Aging is a functional decline that occurs in individuals with the passage of physical time from the reproductive maturity to death and a chronological risk factor for age-associated diseases, such as chronic kidney disease and osteoporosis^1,2^. Senescence is a cellular response against any damage or stress with the stable cell cycle arrest^3^. Although biological mechanism of aging are still unknown, the occurrence and increase of senescent cells are a potent hypothesis^1,4–6^. In this study, senescent cells are defined as cells whose physiological functions decline with age. It is predicted that senescent cells are almost absent in the tissues of young animals and then occur and increase in animal tissues with aging. Since cellular function is a minimum unit of a tissue or organ, physiological functions of tissues are considered to decline with an increase in senescent cells. However, the properties of senescent cells remain unclear. For example, the distribution of senescent cells in the tissues of aged animals is still unknown, and there might be multiple types of senescent cells that have completely distinct characteristics.

Studies using cultured fibroblasts discovered a phenomenon called cellular senescence, which is caused by telomere shortening and/or severe DNA damage that exceeds the capacity of DNA repair mechanisms, and induces permanent cell cycle arrest, which is accompanied by elevations of expression of *cyclin dependent kinase inhibitor 2A* (*Cdkn2a*, *p16*^*Ink4a*^) and/or *cyclin dependent kinase inhibitor 1A* (*Cdkn1a*, *p21*), which inhibit cell cycle progression^7,8^. Moreover, fibroblasts that cause cellular senescence secrete bioactive substances such as interleukins, chemokines, and matrix metalloproteinases, which are called senescence-associated secretory phenotype (SASP), and secreted bioactive substances by SASP are called SASP factors^9,10^. These observations shed light on the biological mechanisms of aging; however, many aspects of aging are still unknown. Since cellular senescence is a phenomenon confirmed in fibroblasts, studies using senescent cells in each tissue of aged animals are more important to elucidate the biological mechanisms and phenomena of aging. Importantly, senescent cells in the tissues of aged animals are distinct from cells that cause cellular senescence.

Senescent cells in the tissues of aged animals remain to be identified. We refer to the marker gene to identify senescent cells in tissues as “age-associated genes”. The age-associated genes include all genes whose expression increases with aging and are not limited by whether they are involved in the principal mechanism of aging. Single cell RNA sequencing (scRNA-seq), which analyzes the transcriptome of individual cells, is a powerful method to analyze cells whose marker gene is unknown, as well as senescent cells. In this study, we searched for age-associated genes to identify senescent cells in the liver and compare transcriptome data in primary hepatocytes from 7- and 27-month-old Fischer 344 male rats using our previously reported next-generation single cell sequencing^11^, a high-throughput scRNA-seq technology.

## Results

### Screening for the age-associated genes in rat hepatocytes using scRNA-seq

**Figure 1:**
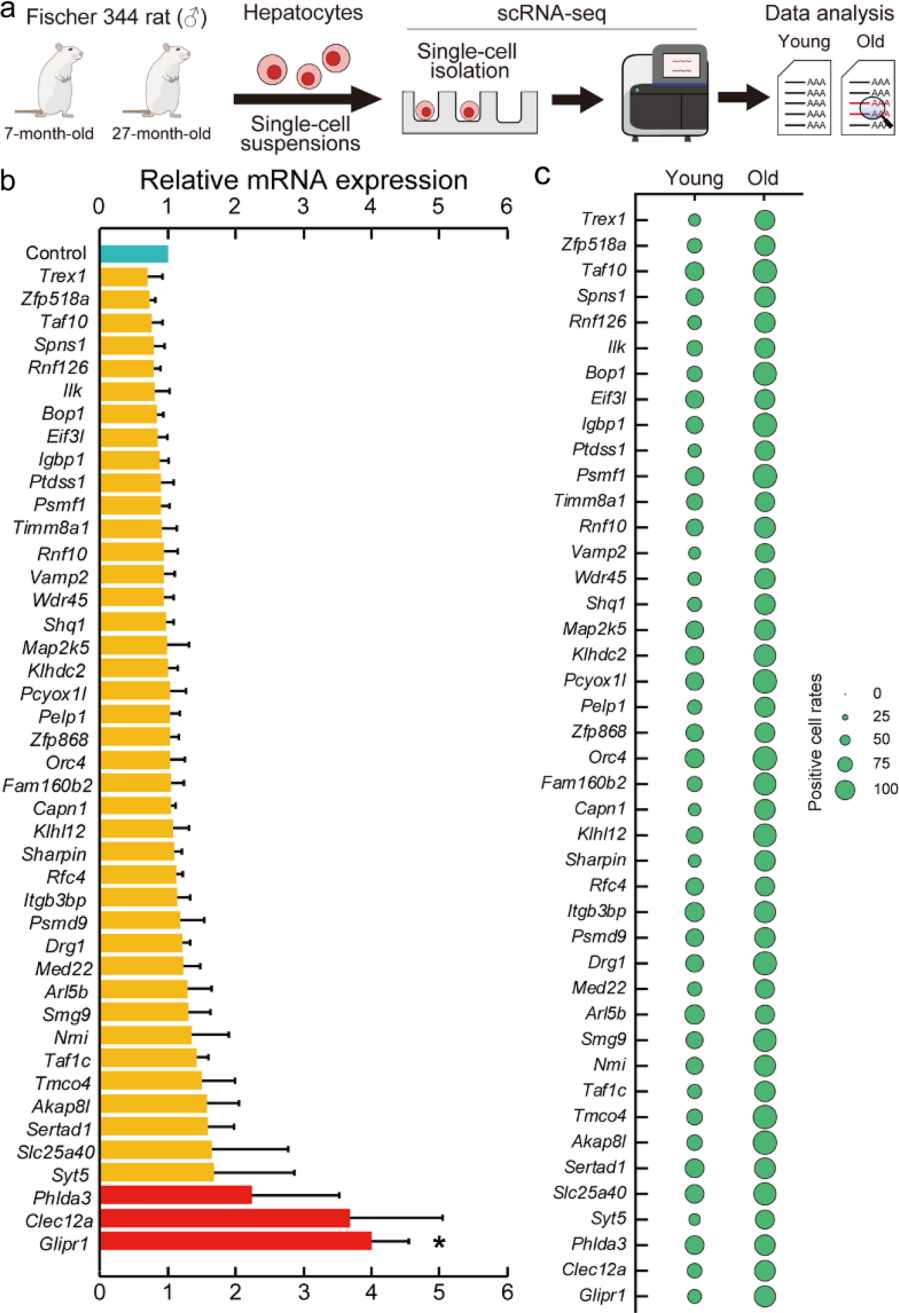
Screening for the age-associated genes in rat primary hepatocytes using scRNA-seq. (**a**) The workflow of the screening using scRNA-seq in the 7-month-old (255 cells) and 27-month-old (1251 cells) Fischer 344 male rat primary hepatocytes. (**b**) RT-qPCR analyzed mRNA expression levels of the top 43 genes selected from Supplementary Table S1. The bar plot shows relative mRNA expression levels in 32–34-month-old C57BL/6J male mouse livers (n = 6) compared to 6-month-old mouse livers (n = 8). Red bars indicate genes whose expression levels more than twofold higher in aged mouse livers than young mouse livers. *β-actin*, a housekeeping gene, was used as the endogenous control gene. Values are presented as the means ± standard error of the means (SEM). The statistical analysis was performed using the two-tailed Welch’s t-test. *p < 0.05. (**c**) This plot was obtained from transcriptome data of 7- and 27-month-old rat primary hepatocytes using R package Seurat 3.2.0^41^. Diameters of circle indicate the percentages of positive-cells per whole primary hepatocytes for each gene 43 genes in (**b**).

We planned the workflow of the screening for the senescent cell markers, age-associated genes, based on the hypothesis that aged animals have more senescent cells than young animals (Fig. 1a). Primary hepatocytes from 7- and 27-month-old Fischer 344 male rats were isolated, and transcriptome data of primary hepatocytes were collected using scRNA-seq. After processing, transcriptome data of cells that express *albumin* (*Alb*) were extracted because *Alb* is a marker gene of hepatocytes. As the first screening strategy, we calculated the positive cell rates, the percentage of cells expressing one gene, and ranked genes on the difference in the positive cell rates between 7- and 27-month-old (Supplementary Table S1). To remove animal species specificity, we searched for genes in Supplementary Table S1 that have orthologs in mice and examined the expression levels of the top 43 genes using reverse transcription-quantitative polymerase chain reaction (RT-qPCR) on 6- and 32–34-month-old C57BL/6J male mouse livers. RT-qPCR analysis revealed three genes, *GLI pathogenesis-related 1* (*Glipr1*), *C-type lectin domain family 12, member a* (*Clec12a*), and *pleckstrin homology-like domain, family A, member 3* (*Phlda3*), whose expression levels in 32–34-month-old mouse livers were twofold higher than those in 6-month-old mouse livers (Fig. 1b). *Glipr1* is a member of the cysteine-rich secretory protein family and shares high sequence similarity among mammals, insects, and plants^12^. *Clec12a* is a C-type lectin receptor that negatively regulates neutrophil activation by inhibiting tyrosine-protein kinase signaling^13,14^. *Phlda3* is a tumor suppressor that inhibits the p53-Akt pathway and induces malignant progression^15^. The positive cell rates of these 43 genes examined by RT-qPCR analysis increased in the transcriptome data of individual rat primary hepatocytes (Fig. 1c).

### Glipr1, Clec12a, and Phlda3 mRNA expression in mouse livers with aging

If senescent cells increase in animal tissues with aging, it follows that the expression levels of age-associated genes should increase with aging. To validate this prediction, we examined the expression levels of *Glipr1*, *Celc12a*, and *Phlda3* in 3-, 6-, 12-, 24-, and 32–34-month-old mouse livers. However, carcinomas and serious diseases are often found in the tissues of aged animals and affect the expression of various genes. To avoid these effects, histological evaluations were carefully performed on 3-, 6-, 12-, 24-, and 32–34-month-old mouse livers for further analyses. Hematoxylin and eosin (HE) staining confirmed the absence of carcinomas or abnormal findings in the livers of all mice used (Supplementary Fig. S1a). Moreover, we performed senescence-associated β-galactosidase (SA-β-gal) staining, a conventional method to stain senescent cells, and confirmed that the staining intensity became stronger with aging in 3-, 6-, 12-, 24-, and 32–34-month-old mouse livers (Supplementary Fig. S1b).

**Figure 2:**
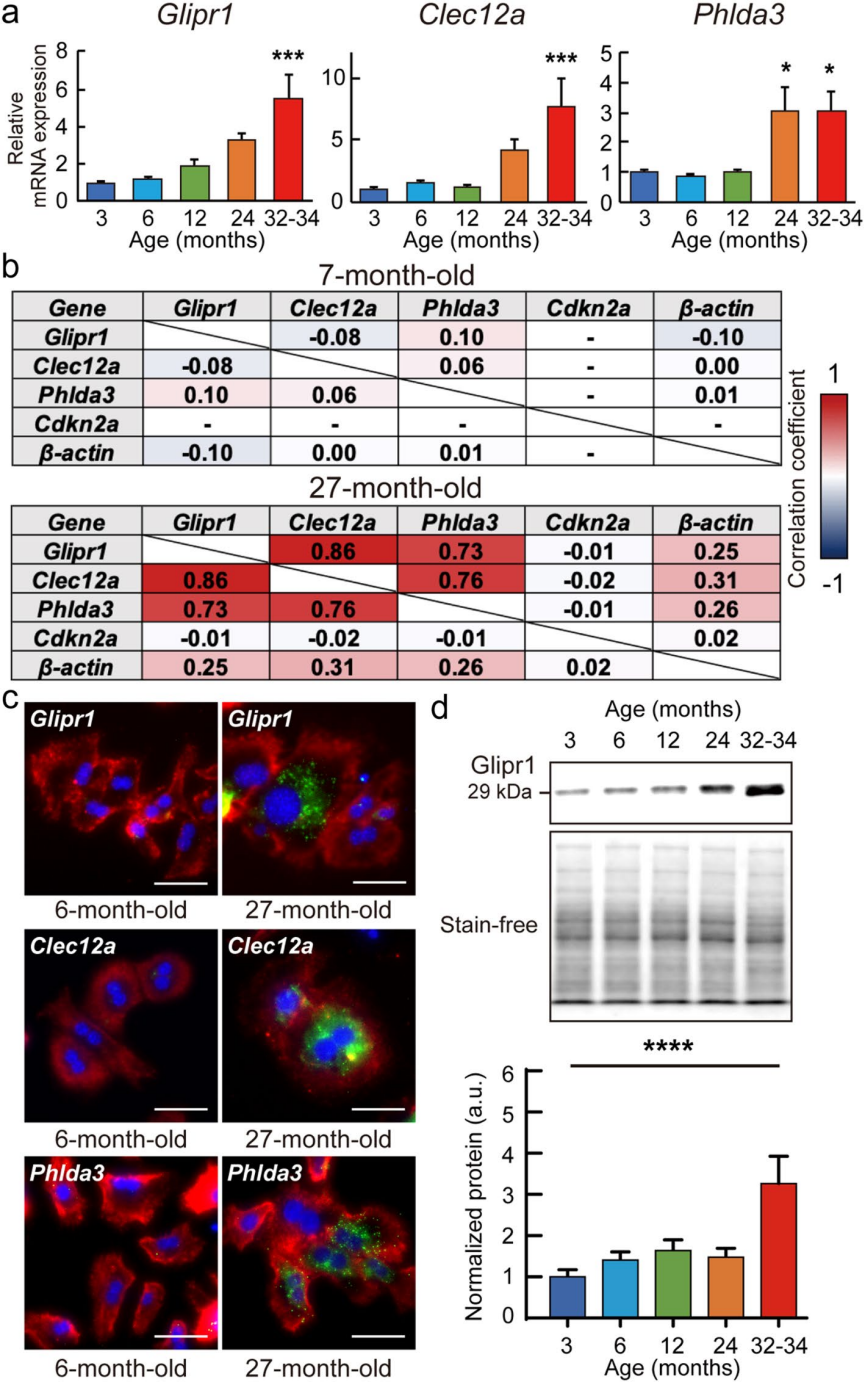
*Glipr1*, *Clec12a*, and *Phlda3* with aging. (**a**) Gene expressions of *Glipr1*, *Clec12a*, and *Phlda3* were analyzed by RT-qPCR in the 3-, 6-, 12-, 24-, and 32–34-month-old mouse livers (n = 5, all ages). Three technical replicates were conducted for each sample. Bar plots show relative mRNA expression levels in 6-, 12-, 24-, and 32–34-month-old mouse livers compared to 3-month-old mouse livers. *β-actin* was used as the endogenous control gene. (**b**) Correlation coefficients calculated from the expression levels of *Glipr1*, *Clec12a*, *Phlda3*, *Cdkn2a*, and *β-actin* in transcriptome data of 7-month-old and 27-month-old rat hepatocytes. The hyphen indicates a pair of genes whose correlation coefficient cannot be calculated by no expression data. (**c**) Representative images stained for *Glipr1*, *Clec12a*, *Phlda3*, and *Alb* mRNA in 6- and 27-month-old primary mouse hepatocytes using fluorescence in situ hybridization (FISH). Green dots indicate *Glipr1*, *Clec12a*, and *Phlda3* mRNA signals. The *Alb* mRNA is shown as red dots. Nuclei were stained by DAPI (blue). Scale bar = 50 μm. (**d**) Glipr1 protein levels were analyzed using western blot analysis in 3-, 6-, 12-, 24-, and 32–34-month-old mouse livers (n = 5, all ages). Upper panels are representative chemiluminescence and stain-free images. Uncropped images are shown in Supplementary Fig. S7a. The right panel shows quantitative data normalized by whole protein levels detected by stain-free. Values are presented as the means ± SEM. The statistical analysis was performed using one-way ANOVA followed by Dunnett’s post hoc test. *p < 0.05, ***p < 0.001, and ****p < 0.0001. a.u., arbitrary units.

Since carcinomas and abnormal findings were not confirmed in the liver, RT-qPCR was performed to examine the expression levels of *Glipr1*, *Clec12a*, and *Phlda3* in 3-, 6-, 12-, 24-, and 32–34-month-old mouse livers. The expression levels of *Glipr1*, *Clec12a*, and *Phlda3* increased with aging and were 5.5, 7.7, and 3.0 times higher in 32–34-month-old mouse livers than in 3-month-old mouse livers, respectively (Fig. 2a).

### Correlations among Glipr1, Clec12a, and Phlda3 gene expression patterns in 7- and 27-month-old rat hepatocytes

To examine the correlation with the gene expression pattern, correlation coefficients were calculated using transcriptome data of 7- and 27-month-old rat hepatocytes. In 7-month-old rat hepatocytes, no significant correlations were observed among *Glipr1*, *Clec12a*, *Phlda3*, and *β-actin*, which are housekeeping genes and gene expression patterns (Fig. 2b). The expression of *Cdkn2a*, which encodes two transcript variants *p16*^*Ink4a*^ and *p19*^*ARF*^, was too low to be detected in the transcriptome data of 7-month-old rat hepatocytes. In 27-month-old rat hepatocytes, high correlations were confirmed among *Glipr1*, *Clec12a*, and *Phlda3* gene expression patterns, and the highest correlation coefficient was 0.86 between *Glipr1* and *Clec12a* (Fig. 2b). *Cdkn2a* expression was detected in 27-month-old rat hepatocytes but did not correlate with other genes such as *Glipr1*, *Clec12a*, and *Phlda3*.

### Fluorescence in situ hybridization (FISH) of Glipr1, Clec12a, and Phlda3

To visualize the expression of *Glipr1*, *Clec12a*, and *Phlda3*, FISH was performed on 6- and 27-month-old mouse primary hepatocytes (Fig. 2c and Supplementary Fig. S2). We confirmed the expression of *Glipr1*, *Clec12a*, and *Phlda3* mRNA in 27-month-old mouse primary hepatocytes with *Alb* mRNA positive, while few cells expressed their mRNA in 6-month-old mouse primary hepatocytes.

### Glipr1 is abundant in aged mouse livers

We focused on *Glipr1* and performed further analyses. Glipr1 protein levels were examined in 3-, 6-, 12-, 24-, and 32–34-month-old mouse livers by western blot analysis. Glipr1 protein levels in 32–34-month-old mouse livers were significantly 3.3-fold higher than those in 3-month-old mouse livers (Fig. 2d). In addition, p16^Ink4a^ protein levels in 24 and 32–34-month-old mouse livers were significantly 2.6- and 3.0-fold higher than those in 3-month-old mouse livers (Supplementary Fig. S3).

### Tissue distribution of Glipr1

**Figure 3:**
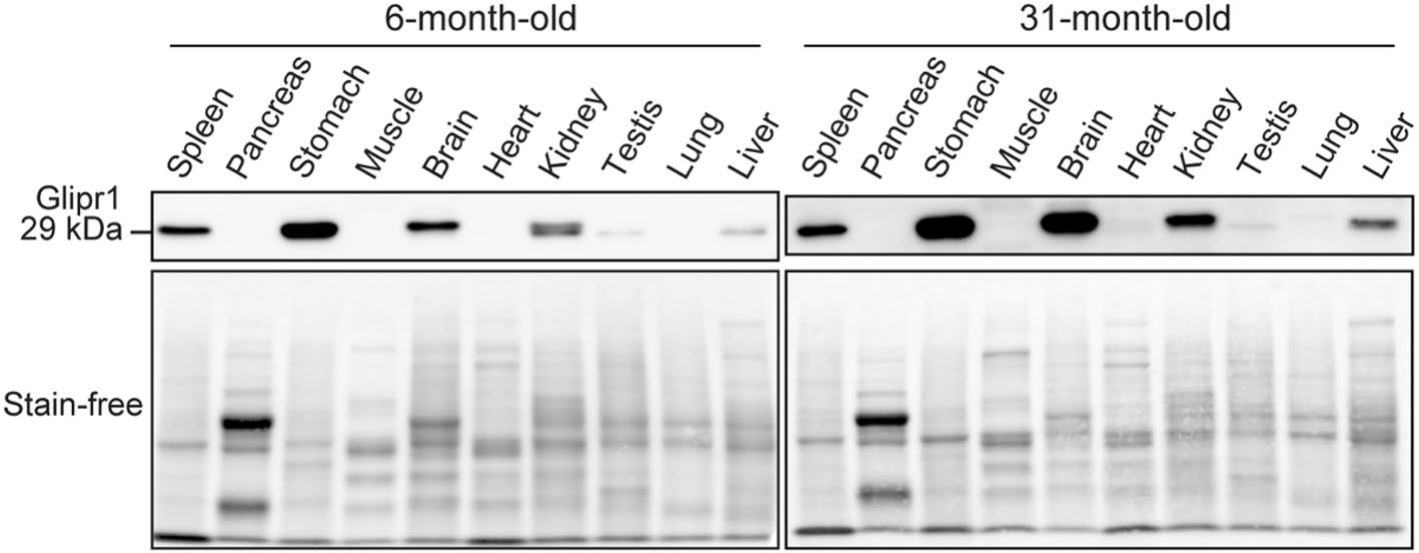
Tissue distribution of Glipr1. Upper panels show the tissue distribution of Glipr1 among ten organs of 6- and 31-month-old mice (n = 1). Bottom panels show the whole proteins detected by stain-free imaging. Uncropped images of western blots are shown in Supplementary Fig. S7b,c.

To understand the tissue distribution of Glipr1, western blot analysis was performed using ten tissues including the spleen, pancreas, stomach, muscle, whole brain, heart, kidney, testis, lung, and liver, of 6- and 31-month-old mice. Glipr1 was abundant in that order in the stomach, whole brain, spleen, kidney, liver, and testis (Fig. 3). No obvious changes in tissue distribution of Glipr1 were observed between 6- and 31-month-old mice.

### Gene expression associated with senescence and cellular senescence

It is known that fibroblasts that cause cellular senescence induce cell cycle arrest and SASP factors, including interleukins, chemokines, matrix metalloproteinases, peptidase inhibitors, and other inflammatory factors^9,10^. It is still unclear how the expression levels of genes associated with cellular senescence changes in mouse livers with aging. To identify genes that reflect aging properties of the liver at each age, RT-qPCR was performed in 3-, 6-, 12-, 24-, and 32–34-month-old mouse livers to examine mRNA expression levels of genes associated with senescence and cellular senescence.

**Figure 4:**
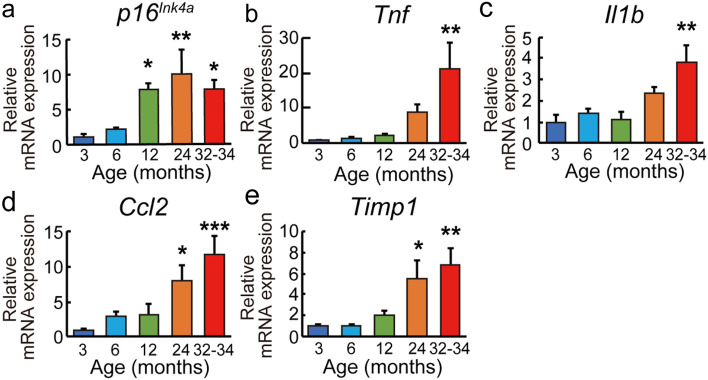
Changes of genes associated with senescence and cellular senescence with aging. Gene expressions of *p16*^*Ink4a*^ (**a**), *Tnf* (**b**), *Il1b* (**c**), *Ccl2* (**d**), and *Timp1* (**e**) were analyzed by RT-qPCR in the 3-, 6-, 12-, 24-, and 32–34-month-old mouse livers (n = 5, all ages). Three technical replicates were conducted for each sample. Bar plots show relative mRNA expression levels in 6-, 12-, 24-, and 32–34-month-old mouse livers compared to 3-month-old mouse livers. *β-actin* was used as the endogenous control gene. Values are presented as the means ± SEM. The statistical analysis was performed using the one-way ANOVA followed by Dunnett’s post hoc test. *p < 0.05, **p < 0.01, and ***p < 0.001.

The expression levels of 43 genes associated with cellular senescence were observed, and expression levels of 13 genes increased with aging. We show representative 5 genes whose expression levels in mouse livers increased with aging: *p16*^*Ink4a*^, *interleukin 1 beta* (*Il1b*), *tumor necrosis factor* (*Tnf*), *chemokine (C-C motif) ligand 2* (*Ccl2*), and *TIMP metallopeptidase inhibitor 1* (*Timp1*) (Fig. 4). Moreover, expression levels of 8 SASP factors, *interleukin 7* (*Il7*), *chemokine (C-X-C motif) ligand 2* (*Cxcl2*), *chemokine (C-X-C motif) ligand 5* (*Cxcl5*), *chemokine (C-X-C motif) ligand 15* (*Cxcl15*), *matrix metallopeptidase 12* (*Mmp12*), and *TIMP metallopeptidase inhibitor 2* (*Timp2*), increased with aging (Supplementary Fig. S4a-c). There was no significant increase with aging in the expression levels of 7 SASP factors, 10 genes associated with cell cycle arrest^16–20^, 2 genes associated with anti-apoptosis^21^, and 11 genes associated with tumorigenesis^16,22,23^ (Supplementary Fig. S4).

Furthermore, to examine the correlation between the expression pattern of genes associated with senescence and cellular senescence, correlation coefficients were analyzed using transcriptome data of 7- and 27-month-old rat hepatocytes. In 27-month-old rat hepatocytes, expressions of *angiogenin* (*Ang*), *complement C3* (*C3*), *cathepsin B* (*Ctsb*), *ETS proto-oncogene 2, transcription factor* (*Ets2*), and *insulin-like growth factor binding protein 1* (*Igfbp1*), *CCAAT/enhancer binding protein beta* (*Cebpb*), *angiopoietin-like 4* (*Angptl4*), *catenin beta 1* (*Ctnnb1*), *C-X-C motif chemokine ligand 10* (*Cxcl10*), *polypyrimidine tract binding protein 1* (*Ptbp1*), and *vascular endothelial growth factor A* (*Vegfa*), showed high correlation coefficients with the expressions of *Glipr1*, *Clec12a*, and *Phlda3* (Supplementary Fig. S5). These genes included in the SenMayo gene set, which indicates transcriptional features of senescent cells in several tissues of humans and mice ^24^.

### Stress-induced premature senescence on primary hepatocytes from 6-month-old mice

**Figure 5:**
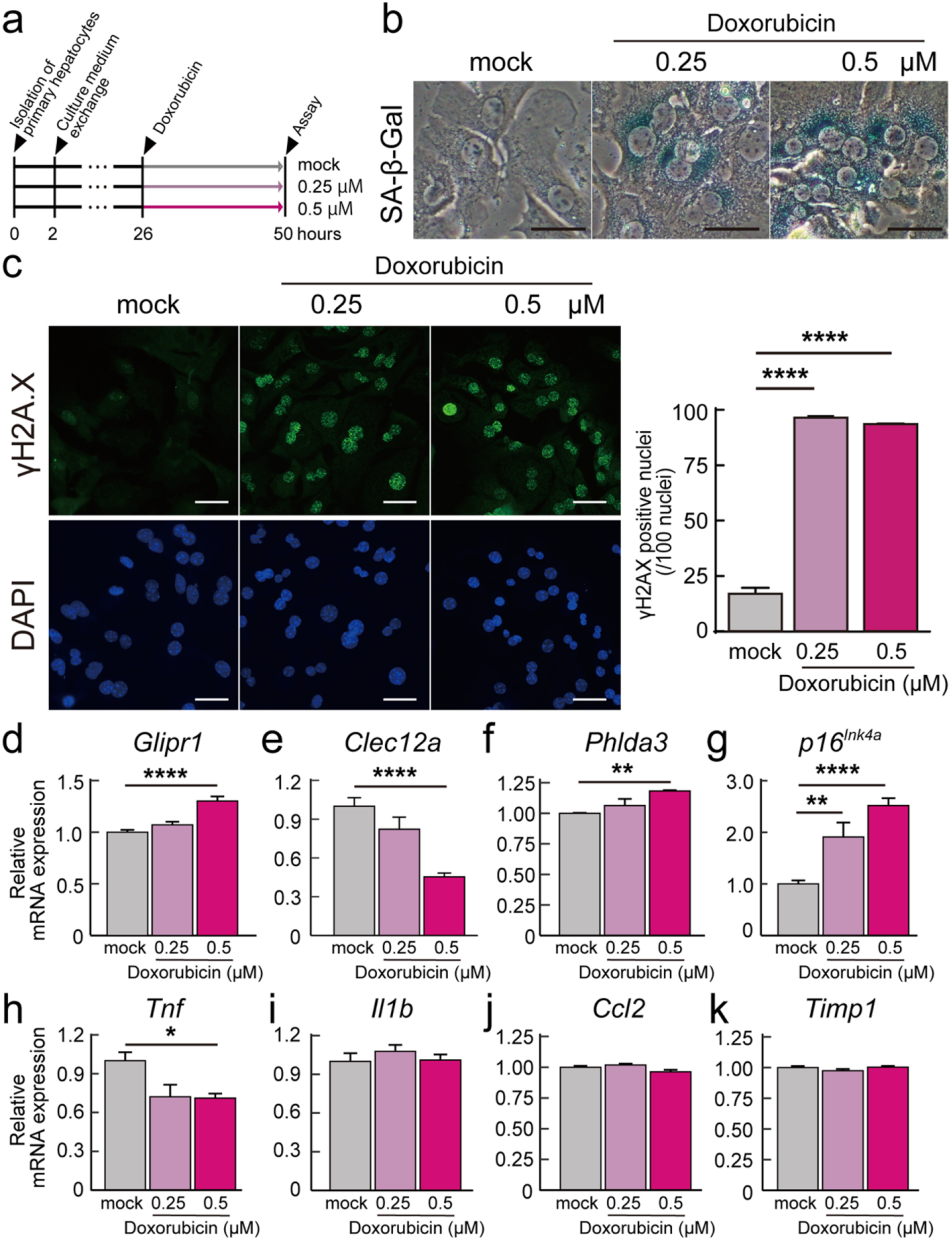
Stress-induced premature senescence on primary hepatocytes administration of doxorubicin. (**a**) Timeline of experiments. Isolated hepatocytes from 6-month-old mice were cultured in William’s Medium E contained 5% fetal bovine serum for 2 h and in William’s Medium E for 24 h. The experiments were performed with three groups: mock group without doxorubicin, and two groups with doxorubicin added at final concentrations of 0.25 μM and 0.5 μM after 26 h of isolation. Six biological replicates were used for each group. (**b**) Representative images of senescence-associated β-galactosidase (SA-β-gal) staining on 6-month-old mouse primary hepatocytes of three experimental groups (mock, 0.25 μM, and 0.5 μM). Scale bar = 100 μm. (**c**) On the left, representative immunofluorescence images of 6-month-old mouse primary hepatocytes stained with γ-H2A.X antibody (green) and DAPI (blue). Scale bar = 50 μm. The right figure shows quantitative data of γ-H2A.X-positive nuclei per DAPI-positive nuclei (n = 6). (**d**–**k**) The mRNA expression levels of *Glipr1*, *Clec12a*, *Phlda3*, *p16*^*Ink4a*^, *Tnf*, *Il1b*, *Ccl2*, and *Timp1* were analyzed by RT-qPCR (n = 6). *β-actin* was used as the endogenous control gene. Values are presented as the means ± SEM. The statistical analysis was performed using one-way ANOVA followed by Dunnett’s post hoc test. *p < 0.05, **p < 0.01, and ****p < 0.0001.

In recent years, many studies have been conducted to induce premature senescence in cells and animals by administering drugs such as doxorubicin^18,25^. Doxorubicin is an anticancer drug commonly used to induce premature senescence in cells and animals and induces double-strand breaks in DNA through interactions with DNA topoisomerase II^26,27^. Therefore, we used doxorubicin to induce premature senescence and examined the mRNA expression levels of *Glipr1*, *Clec12a*, and *Phlda3*. We induced premature senescence in primary hepatocytes obtained from 6-month-old mice by the administration of doxorubicin (Fig. 5a). The experiments were performed in three groups: mock group without doxorubicin, and two groups with doxorubicin added at final concentrations of 0.25 μM and 0.5 μM. After 24 h of doxorubicin administration, we performed SA-β-Gal staining and confirmed staining on mouse primary hepatocytes when treated with both 0.25 μM and 0.5 μM doxorubicin (Fig. 5b). γ-H2A.X, a phosphorylated histone H2 variant, is commonly used as a marker of DNA repair^28^. We performed immunofluorescence staining for γ-H2A.X and confirmed the presence of γ-H2A.X-positive nuclei were higher in the doxorubicin-treated groups than in the mock group (Fig. 5c). Since these results suggest that doxorubicin successfully induced premature senescence in mouse primary hepatocytes, we performed RT-qPCR to examine the mRNA expression levels of *Glipr1*, *Clec12a*, and *Phlda3*. The mRNA expression levels of *Glipr1* and *Phlda3* were significantly higher in the 0.5 μM doxorubicin treated group than in the mock group, while the mRNA expression level of *Clec12a* was significantly lower in the 0.5 μM doxorubicin treated group than in the mock group (Fig. 5d–f). Moreover, we examined the mRNA expression levels of *p16*^*Ink4a*^, *Tnf*, *Il1b*, *Ccl2*, and *Timp1*, genes whose mRNA expression levels increased with aging in mouse livers (Fig. 4) as well as *Glipr1*, *Clec12a*, and *Phlda3*. The expression levels of *p16*^*Ink4a*^ were significantly higher in the 0.25 μM and 0.5 μM doxorubicin treated groups than in the mock group, while the expression level of *Tnf* was significantly lower in the 0.5 μM doxorubicin treated group than in the mock group (Fig. 5g,h). There were no significant differences in the mRNA expression levels of *Il1b*, *Ccl2*, and *Timp1* among any of the groups (Fig. 5i–k).

### Stress-induced senescence in vivo using 6-month-old mice by administration of doxorubicin

**Figure 6:**
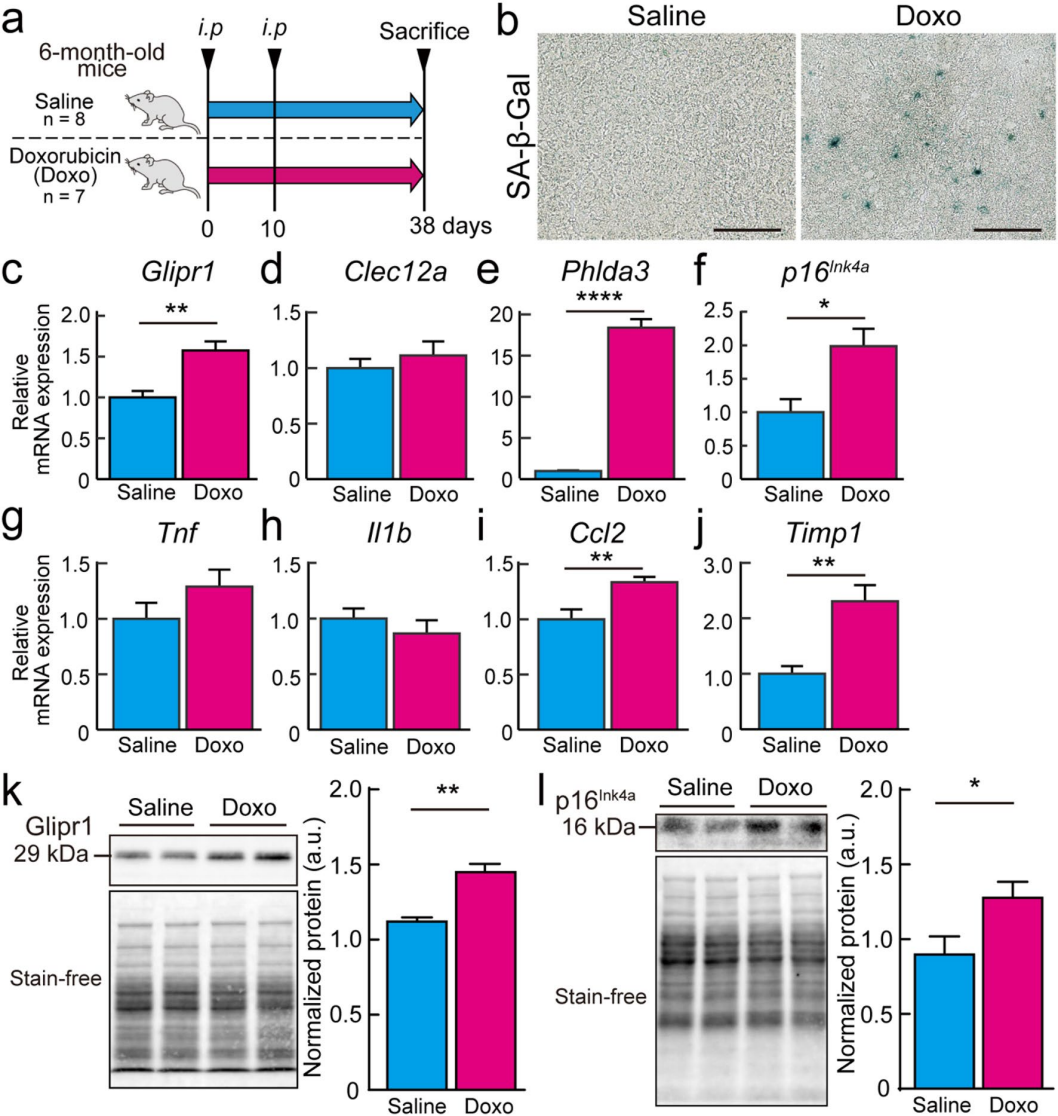
Stress-induced senescence in vivo by administration of doxorubicin. (**a**) Timeline of experiments. We administrated saline (n = 8) or doxorubicin (10 mg/kg, n = 7) by intraperitoneal injection (*i.p*) to 6-month-old mice to induce senescence. The second *i.p* was administered 10 days after the first *i.p*, and the animals were dissected 38 days after the first *i.p*. Two experimental groups that administrated saline or doxorubicin are indicated as “Saline” and “Doxo”. (**b**) Representative images of SA-β-gal staining on mouse liver frozen sections of two experimental groups. Scale bar = 100 μm. (**c**–**j**) The mRNA expression levels of *Glipr1*, *Clec12a*, *Phlda3*, *p16*^*Ink4a*^, *Tnf*, *Il1b*, *Ccl2*, and *Timp1* in the livers of mice administrated with saline (n = 8) or doxorubicin (n = 7) were analyzed by RT-qPCR. Three technical replicates were conducted for each sample. *β-actin* was used as endogenous control gene. (**k**–**l**) Protein levels of Glipr1 (k) and p16^Ink4a^ (**l**) in the livers of mice administrated with saline (n = 8) or doxorubicin (n = 7) were examined using western blot analysis. Left panels are representative images of the chemiluminescence and stain-free. The right panel shows quantitative data normalized by whole protein levels detected by stain-free imaging. Uncropped images of western blots are shown in Supplementary Fig. S7d,e. Values are presented as the means ± SEM. The statistical analysis was performed using the two-tailed Welch’s t-test. *p < 0.05, **p < 0.01, and ****p < 0.0001.

Next, we administered saline or doxorubicin by intraperitoneal injection (*i.p.*) to 6-month-old mice to induce senescence in vivo and examined whether the mRNA expression of *Glipr1*, *Clec12a*, and *Phlda3* was increased (Fig. 6a). The second *i.p.* was administered 10 days after the first *i.p.*, and mice were dissected 38 days after the first *i.p*. These experimental conditions, such as administration schedule and dose of doxorubicin, were followed by Baar’s work^18^. SA-β-Gal staining of frozen sections of mouse livers showed stronger staining in livers from mice administered doxorubicin than in livers from mice administered saline (Fig. 6b). This confirmed that stress-induced senescence in mouse livers. Doxorubicin has been reported to induce damage to the liver and kidneys^26,27^. Therefore, we examined biochemical blood plasma components and histology of the livers and kidneys of mice to confirm the presence of serious organ damage caused by doxorubicin. The effects of doxorubicin were analyzed using alanine aminotransferase (ALT) and aspartate aminotransferase (AST) as indicators of liver damage, urea nitrogen, and creatinine as indicators of kidney damage, and total cholesterol (T-CHO) and triglycerides as indicators of nutritional status. There were no significant differences in ALT, AST, urea nitrogen, creatinine, T-CHO, and triglyceride levels in the plasma of mice treated with saline and doxorubicin (Supplementary Fig. S6a). Moreover, the rate of change in body weight from the first *i.p.* to sacrifice was significantly lower in mice administered doxorubicin than in those administered saline, while there was no significant difference in the liver weight per body weight of mice administered saline and doxorubicin (Supplementary Fig. S6b,c). HE staining showed no carcinomas or abnormal findings in the liver and kidneys of all mice used (Supplementary Fig. S6d,e). These results confirmed that doxorubicin induced no severe damage to the liver and kidneys. Therefore, we performed RT-qPCR to examine the mRNA expression levels of *Glipr1*, *Clec12a*, *Phlda3*, *p16*^*Ink4a*^, *Tnf*, *Il1b*, *Ccl2*, and *Timp1*. The mRNA expression levels of *Glipr1*, *Phlda3*, *p16*^*Ink4a*^, *Ccl2*, and *Timp1* were significantly higher in the livers of mice administered doxorubicin than in those of mice administered saline, while there were no significant differences in the expression levels of *Clec12a*, *Tnf*, and *Il1b* between the two experimental groups (Fig. 6c–j). Likewise, western blot analysis showed that protein levels of Glipr1 and p16^Ink4a^ were significantly higher in the livers of mice administered doxorubicin than in those of mice administered saline (Fig. 6k,l).

## Discussion

In this study, we performed scRNA-seq using young and old rat primary hepatocytes to identify the many age-associated genes and marker genes to identify senescent cells in the liver, and found *Glipr1*, *Clec12a*, and *Phlda3*, whose mRNA expression levels were more than two-fold higher in aged mouse livers than in young mouse livers. Additionally, we confirmed the abundance of *Glipr1*, *Clec12a*, and *Phlda3* mRNA in 27-month-old mouse primary hepatocytes using FISH. Recently, many studies have been conducted to induce senescence in cells and animals by administering drugs such as doxorubicin^18,25^. Therefore, we performed experiments using mouse primary hepatocytes and mice treated with doxorubicin and found that the mRNA expression levels of *Glipr1* and *Phlda3* were increased in primary hepatocytes and livers of young mice.

*Glipr1* is a member of the cysteine-rich secretory protein family and shares high sequence similarity among mammals, insects, and plants^12^. Overexpression of *Glipr1* induces apoptosis in some cancer cell lines derived from prostates, human lungs, and human colons^29^. *Clec12a* is a C-type lectin receptor that negatively regulates neutrophil activation by inhibiting tyrosine-protein kinase signaling^13,14^. *Phlda3* is a tumor suppressor that inhibits the p53-Akt pathway and induces malignant progression^15^. The relationships between aging and these three genes (*Glipr1*, *Clec12a*, and *Phlda3*) have not yet been reported. Orthologs of these genes in the human genome have also been confirmed (*GLIPR1*, *CLEC12A*, and *PHLDA3*)^30–32^. This suggests the possibility that *Glipr1*, *Clec12a*, and *Phlda3* could be applied to age-associated genes in human livers. If senescent cells increase in animal tissues with aging, the expression levels of age-associated genes should increase with aging. To validate this prediction, RT-qPCR was performed to examine expression levels in 3-, 6-, 12-, 24-, and 32–34-month-old mouse livers and to confirm that the expression levels of *Glipr1*, *Clec12a*, and *Phlda3* increased with aging. Based on the above prediction, we considered that *Glipr1*, *Clec12a*, and *Phlda3* are age-associated genes in the liver.

The expression patterns of *Glipr1*, *Clec12a*, and *Phlda3* were highly correlated in the transcriptome data of 27-month-old rat hepatocytes, while there was no correlation among the genes in the transcriptome data of 7-month-old rat hepatocytes. This suggests that *Glipr1*, *Clec12a*, and *Phlda3* are highly expressed in the same primary hepatocytes of old rats, and this cell might occur and increase in rat livers with aging.

The SenMayo gene set had been validated as a good indicator of transcriptional features of senescent cells in human bone, human adipose tissue, murine brain, and murine bone marrow^24^. In this study, high correlation coefficients of *Glipr1*, *Clec12a*, and *Phlda3* against eleven genes (*Ang*, *C3*, *Ctsb*, *Ets2*, *Igfbp1*, *Cebpb*, *Angptl4*, *Ctnnb1*, *Cxcl10*, *Ptbp1*, and *Vegfa*) of the SenMayo gene set were confirmed. This suggests that *Glipr1*, *Clec12a*, and *Phlda3* might be potential markers of senescent cells in the livers of aged animals. Additionally, FISH on 27-month-old mouse primary hepatocytes indicated the presence of cells abundantly expressing *Glipr1*, *Clec12a*, and *Phlda3* mRNA.

Western blot analysis confirmed that Glipr1 was abundant in the stomach, whole brain, spleen, and kidney, while little was found in the liver and testis of a 6 and 31-month-old mice. This suggests that the Glipr1 protein level might be maintained at low levels in the 6-month-old mouse liver. However, the tissue distribution of Glipr1 among the 10 organs was independent of developmental lineage and organ function.

We used 7- and 27-month-old rat primary hepatocytes and 3-, 6-, 12-, 24-, and 32–34-month-old mouse livers to search for age-associated genes. The average lifespan of Fischer344 male rats and C57BL/6 male mice is approximately 31 and 29 months, respectively^33,34^. Therefore, the aging progression of 27-month-old rats and 24-month-old mice is presumed to be comparable.

To validate genes reflecting aging properties of the mouse liver at each age, RT-qPCR was performed to examine the expression levels of genes associated with senescence and cellular senescence in mouse livers. We confirmed the increase in the expression levels of 13 genes, including *Il1b*, *Il7*, *Ccl2*, *Ccl8*, *Cxcl2*, *Cxcl5*, *Cxcl15*, *Mmp9*, *Mmp12*, *Timp1*, *Timp2*, *Tnf*, and *p16*^*Ink4a*^, as well as *Glipr1*, *Clec12a*, and *Phlda3* with aging*.* Moreover, p16^Ink4a^ protein levels in mouse livers increased with aging. *p16*^*Ink4a*^ is a cycle-dependent kinase inhibitor that inhibits cell cycle progression^8^, and the other 12 genes are SASP factors that are secreted from fibroblasts that cause cellular senescence^9,10^. These results suggest that old mouse livers tend to cause chronic inflammation and cell cycle arrest, and that these 13 genes might reflect aging properties of the liver at each age. However, it is unclear whether these genes are functionally related to *Glipr1*, *Clec12a*, and *Phlda3*. There was no increase with aging in the expression levels of genes associated with anti-apoptosis and tumorigenesis, which is reasonable because we confirmed the absence of carcinomas in 3-, 6-, 12-, 24-, and 32–34-month-old mouse livers before gene expression analyses.

Based on our results, we discovered that the expression levels of *Glipr1*, *Clec12a*, and *Phlda3* increased with aging in mouse livers. Next, we introduced stress-induced premature senescence to primary hepatocytes and livers of 6-month-old mice using doxorubicin and investigated the mRNA expression levels of *Glipr1*, *Clec12a*, and *Phlda3*. Our results suggest that *Glipr1*, *Phlda3*, and *p16*^*Ink4a*^ were age-associated genes whose expression levels increased with stress-induced premature senescence using doxorubicin and with aging. Since the expression level of *Clec12a* did not increase by stress-induced premature senescence using doxorubicin, *Clec12a* might be an age-associated gene whose expression levels increase only with aging but not with stress-induced premature senescence. On the other hand, the expression levels of *Tnf*, *Il1b*, *Ccl2*, and *Timp1* were independent of *Glipr1*, *Clec12a*, and *Phlda3* in mouse primary hepatocytes and mouse livers induced by stress-induced premature senescence.

Since the properties of senescent cells in animal tissues are largely unknown, we need to consider that there might be multiple types of senescent cells with completely different properties. Actually, Ogrodnik et al*.*^35^ reported that senescent cells in liver promotes hepatic fat accumulation and steatosis, and a close correlation between hepatic fat accumulation and markers of hepatocyte senescence such as telomere-associated DNA damage foci. Therefore, primary hepatocytes expressing *Glipr1*, *Clec12a*, and *Phlda3* are predicted to be one of the multiple types of senescent cells in the liver of aged animals. In further analyses, we will examine the distributions of *Glipr1*, *Clec12a*, *Phlda3*, and other genes associated with aging in aged mouse livers. Recently, many researchers have attempted to develop senolytic drugs^18,36^, which are drugs that specifically induce apoptosis in senescent cells. We also expect that *Glipr1*, *Clec12a*, and *Phlda3* will contribute to the development of senolytic drugs.

In conclusion, this study is the first to identify *Glipr1*, *Clec12a*, and *Phlda3* as age-associated genes that identify senescent cells in the liver. Our results suggest a high possibility that *Glipr1*, *Clec12a*, and *Phlda3* are expressed in senescent cells in the liver, and that primary hepatocytes with abundant *Glipr1*, *Clec12a*, and *Phlda3* mRNA confirmed by FISH might be senescent cells.

### Limitations

In the present study, scRNA-seq was performed by using liver parenchymal cells expressing albumin, a differentiation marker for liver parenchymal cells. Therefore, liver nonparenchymal cells, inflammatory cells, and liver stem cells that do not express albumin were not included in this scRNA-seq analysis.

## Methods

### Ethics statement

All animal experiments were approved by the Animal Care and Use Committee of the Tokyo Metropolitan Institute for Geriatrics and Gerontology (TMIG) (Permit Number: 21012) and conducted in the Guidelines for the Care and Use of Laboratory Animals of TMIG. All methods of animal experiments are reported in accordance with ARRIVE (Animal Research: Reporting of In Vivo Experiments) guidelines.

### Animals

Male Fischer 344 rats and male C57BL/6J mice were obtained from the animal facility of TMIG. The animals were fed CRF-1 (Oriental Yeast Ltd., Tokyo, Japan)^37^ ad libitum and maintained under a 12-h light/dark cycle in a controlled environment. The sample size was determined using G*Power software^38^ with a power of 80% and alpha rate of 5%. Animal discomfort was kept to a minimum in all animal experiments. Animals were randomly divided in each experimental group, but blinding was not performed in this study. After the mice were anesthetized with isoflurane (Pfizer Inc., New York, NY, USA), blood was collected from the inferior vena cava using ethylenediaminetetraacetic acid (EDTA)-containing syringes and perfused using phosphate-buffered saline (PBS) to drain blood from tissues. Livers were frozen using liquid nitrogen and stored at − 80 °C until use. Plasma was obtained by centrifuging the blood samples at 880 × g for 15 min at 4 °C and stored at − 80 °C until use. The plasma levels of ALT, AST, urea nitrogen, creatinine, T-CHO, and triglycerides were assessed biochemically (Oriental Yeast Co., Ltd., Tokyo, Japan).

### Isolation of hepatocytes

Rats were perfused with medium containing type IV collagenase from Clostridium histolyticum (Merck KGaA, Darmstadt, Germany) from the portal vein to degrade cell–cell adhesion^39^. The liver was minced in cold medium containing ethylene-bis (oxyethylenenitrilo) tetraacetic acid (Merck KGaA, Darmstadt, Germany), filtered through a 100 μm nylon mesh filter (Corning, Corning, NY, USA), and centrifuged at 90 × g for 5 min at 4 °C to remove nonparenchymal cells. The cell pellet was suspended in William’s Medium E (Thermo Fisher Scientific, Waltham, MA, USA) containing 5% fetal bovine serum, and cultured (1.25 × 10^5^ cells/ml) at 37 °C under 5% CO_2_ in air for 2 h on dishes coated with collagen (Merck KGaA, Darmstadt, Germany). Hepatocytes were collected using 0.025% trypsin–EDTA and centrifuged at 90 × g for 5 min at 4 °C.

### scRNA-seq

Single-cell transcriptome analysis was performed as described previously^11^. Briefly, poly (dT) barcoded beads were first added to a microwell slide (1.6 × 10^5^ wells, 2 × 2 mm) at 1 bead/well. Cells were allowed to settle into the wells of a polydimethylsiloxane slide via gravity. After the slides were incubated with a cell lysis solution, poly (dT) barcoded beads bound to cellular mRNA and collected in a microtube, cDNA was synthesized using SuperScript IV reverse transcriptase (Invitrogen, Carlsbad, CA, USA) in buffer (1 × SSIV buffer, 1 mM dNTPs (Invitrogen, Carlsbad, CA, USA), 20% betaine, 6 mM MgCl_2_, 1.65 units/μl RNasin (Promega, Madison, WI, USA), 5 mM dithiothreitol) with template switching oligo (TSO; 5′-Biotin-CTATGCGCCTTGCCAGCCCGCTCAGGAAT-rGrGrG-3′) at 42 °C for 90 min, 10 cycles of 50 °C for 2 min and 42 °C for 2 min, then 70 °C for 15 min, and held at 4 °C. The cDNA was then stored at − 20 °C until use.

### Preparation of sequencing library

The cDNA was amplified by using custom primers (5′-Biotin-CTATGCGCCTTGCCAGCCCGCTCAG-3′) based on bar-code beads, and cDNA of 500–2000 bp size was extracted, following the fragmentation to 300–350 bp using the M220 Focused-ultrasonicator (Covaris Inc., Woburn, MS, USA). A sequence library was produced following the instructions of the Illumina TruSeq™ library prep kit (Illumina, San Diego, CA, USA). The quality and quantity of the sequencing libraries were confirmed using an Agilent 4200 TapeStation (Agilent, Santa Clara, CA, USA) and Roche® KAPA Library Quantification Kits (Merck KGaA, Darmstadt, Merck KGaA, Darmstadt, Germany). Sequence libraries were sequenced for 60 bases from the side of the barcode sequence and 90 bases from the side of the mRNA using the paired-end sequencing mode of MiniSeq or HiSeq2500 (Illumina, San Diego, CA, USA) with custom primers (5′-GCCTGTCCGCGG CTATGCGCCTTGCCAGCCCGCTCAGAC-3′)^11^.

### Generation of transcriptome data

The scRNA-seq data were aligned and annotated as described previously^11^. Briefly, barcode sequences were extracted from the read 1 FastQ files. The read 2 FastQ files, which included each cell mRNA, were directly aligned to Refseq transcript sequences using bowtie 2.2.6^40^. The aligned reads were linked to the paired extracted barcode sequences. By counting mapped reads per barcode, the gene count data in individual cells were obtained, and the reads per kilobase of transcript per million reads mapped (RPKM) of each gene were calculated for each cell. Transcriptome data from individual cells obtained from primary hepatocytes were analyzed using the R package Seurat 3.2.0^41^. Gene expression matrices of primary hepatocytes of 7-month-old (255 cells, 13,751 genes) and 27-month-old (1251 cells, 14,996 genes) rats were obtained. To check for quality of scRNA-seq data, cells expressing less than 200 genes, and cells in which expression of mitochondrial genes accounts for more than 20% of total expression were excluded. A cell expressing a gene with expression level higher than 1 RPKM was counted as a positive cell^42^. The positive cell rate for a gene is calculated by dividing the number of cells positive for a gene by the total number of cells.

### HE staining

Formalin-fixed paraffin-embedded Sects. (6 μm) of the livers and kidneys were obtained from mice and HE staining was performed. Images were obtained using a NanoZoomer 2.0 RS virtual slide scanner and NDPview2 viewing software (Hamamatsu Photonics, Shizuoka, Japan).

### SA-β-Gal staining

Primary hepatocytes or frozen liver Sects. (14 μm) were stained with a senescence detection kit (BioVision, Milpitas, CA, USA), following the manufacturer’s protocol. Images were obtained using a NanoZoomer 2.0 RS virtual slide scanner and NDPview2 viewing software (Hamamatsu Photonics, Shizuoka, Japan).

### RT-qPCR

Total RNA was extracted from the liver using ISOGEN (FUJIFILM Wako Pure Chemical, Osaka, Japan). cDNA was synthesized using SuperScript III reverse transcriptase (Invitrogen, Carlsbad, CA, USA) and stored at − 80 °C until use. The primer sequences are provided in Supplementary Table S2. RT-qPCR was performed using StepOne Plus (Applied Biosystems, Foster City, CA, USA) and the THUNDERBIRD® SYBR qPCR Mix (Toyobo, Osaka, Japan), following the manufacturer’s protocol. The amplification protocol consisted of denaturation at 95 °C for 1 min, 40 cycles of 95 °C for 15 s, and 60 °C for 1 min. For quantitative analysis of each expression level, a standard curve method was performed; that is, an aliquot from each experimental sample was used to generate standard curves. The expression levels of each gene were normalized to those of β-actin.

### FISH

Mouse primary hepatocytes were stained with the ViewRNA Cell Assay Kit (Thermo Fisher Scientific, Waltham, MA, USA), following the manufacturer’s protocol. ViewRNA probes of *Glipr1*, *Clec12a*, *Phlda3*, and *Alb* were synthesized by Thermo Fisher. Fluorescence signals were detected using a BZ-X710 microscope (Keyence, Neu-Isenburg, Germany). Images were processed using Fiji software (http://fiji.sc/Fiji).

### Immunofluorescence

Isolated mouse primary hepatocytes were reseeded on a coated 35 mm glass bottom dish (Matsunami Glass Ind., Ltd., Osaka, Japan) and incubated at 37 °C under 5% CO_2_ in air for 24 h. After washing with PBS, cells were fixed with 10N mildform (FUJIFILM Wako Pure Chemical, Osaka, Japan) for 2 min at room temperature, and permeabilized with 0.5% TritonX-100/PBS for 2 min. After blocking with 2% bovine serum albumin/PBS for 20 min, cells were incubated with rabbit anti-γ-H2A.X (1:400 dilution, Cell Signaling Technology, Danvers, MA, USA, #2577) primary antibodies diluted in 2% bovine serum albumin/PBS overnight at 4 °C. After washing with PBS, antigen–antibody complexes were incubated with Alexa Fluor488-conjugated goat anti-rabbit IgG (1:2500 dilution, Invitrogen, Carlsbad, CA, USA, A11070) and 4,6-diamidino-phenyl indole dihydrochloride (DAPI) (1:10,000 dilution, Merck KGaA, Darmstadt, Germany) for 1 h at room temperature. After mounting with Fluoromount/Plus (Diagnostic BioSystems Inc., Pleasanton, CA, USA), the fluorescence signals were detected using a BZ-X710 microscope (Keyence, Neu-Isenburg, Germany). Images were processed using Fiji software (http://fiji.sc/Fiji). The percentages of γ-H2A.X-positive nuclei per DAPI-positive nuclei were calculated in four randomly selected 0.4 mm^2^ fields (726 μm × 544 μm) from each group.

### Western blot analysis

The organs and primary hepatocytes of mice were homogenized in lysis buffer (10 mM Tris–HCl 7.6, 0.5 mM EDTA, 0.1% sodium dodecyl sulfate) on ice. The samples were mixed with sample buffer containing 4% sodium dodecyl sulfate and 10% 2-mercaptoethanol and boiled for 5 min. Proteins (10–30 μg per well) were loaded in 10% or 12% Mini-PROTEAN® TGX Stain-Free™ Protein Gels (Bio-Rad, Hercules, CA, USA). Stain-free signals were activated by UV irradiation for 5 min. Semi-dry transfer to 0.2 μm poly vinylidene difluoride (PVDF) membranes were performed using a Trans-Blot Turbo Transfer System (Bio-Rad, Hercules, CA, USA). Stain-free images of the PVDF membranes were captured using the ChemiDoc Touch MP Imaging System (Bio-Rad, Hercules, CA, USA). The blots were blocked with 5% skim milk in tris-buffered saline (TBS) containing 0.1% Tween-20 (TBS-T) for 1h at room temperature, and then incubated with the following primary antibodies diluted in 5% skim-milk in TBS-T overnight at 4 °C: rabbit anti-Glipr1 (1:4000 dilution, Abcam, Cambridge, UK, ab198215); mouse anti-p16 INK4a (1:100 dilution, Santa Cruz Biotechnology, Dallas, TX, USA, sc-1661). After washing with TBS-T, the blots were incubated with horseradish peroxidase-conjugated anti-rabbit or anti-mouse IgG (Bio-Rad, Hercules, CA, USA) diluted in 5% skim milk in TBS-T for 1 h at room temperature, and the chemiluminescence was visualized using Amersham ECL select (Cytiva, Marlborough, MA, USA). The bands were quantified using Image Lab Software (Bio-Rad, Hercules, CA, USA) and normalized to the whole stain-free signals in each lane.

### Statistical analysis

The results were expressed as mean ± standard error of the mean (SEM). The probability of statistical differences between experimental groups was determined using two-tailed Welch's t-test and Dunnett’s test. All statistical tests were conducted by the R and R packages. Differences were considered statistically significant at *P* < 0.05.

### Supplementary Information

Supplementary Information.

## Data Availability

The datasets generated and/or analyzed during the current study are available from the corresponding author upon reasonable request. The scRNA-seq data were deposited in the DNA Databank of Japan (DDBJ) with the accession number DRA013363.

## Abbreviations

ALT: Alanine aminotransferase
AST: Asparagine aminotransferase
DAPI: 4,6-Diamidino-phenyl indole dihydrochloride
EDTA: Ethylenediaminetetraacetic acid
FISH: Fluorescence in situ hybridization
HE: Hematoxylin and eosin
*i.p*: Intraperitoneal injection
PBS: Phosphate buffered saline
PVDF: Poly vinylidene difluoride
RPKM: Reads per kilobase of transcript per million reads mapped
RT-qPCR: Reverse transcription-quantitative polymerase chain reaction
SA-β-gal: Senescence-associated β-galactosidase
SASP: Senescence-associated secretory phenotype
scRNA-seq: Single cell RNA sequencing
RT-qPCR: Reverse transcription-quantitative polymerase chain reaction
SEM: Standard error of the means
TBS: Tris-buffered saline
T-CHO: Total cholesterol
