# Supplementary material for: Single-cell RNA sequencing to detect age-associated genes that identify senescent cells in the liver of aged mice
Source: Sci Rep. 2023 Aug 30;13:14186. doi: 10.1038/s41598-023-41352-6 (PMC10468526; doi:10.1038/s41598-023-41352-6)
Supplement: Supplementary file 1 — Supplementary Information. [file 41598_2023_41352_MOESM1_ESM.pdf]

Supplementary Materials

Single-cell RNA sequencing to detect age-associated genes that identify senescent cells in the liver of aged mice

Yuta Doshida[a,b], Shinichi Hashimoto[c], Sadahiro Iwabuchi[c], Yuka Takino[a], Toshiyuki Ishiwata[c], Toshiro Aigaki[b], Akihito Ishigami [a,b,1]

[a] Molecular Regulation of Aging, Tokyo Metropolitan Institute of Gerontology, Tokyo 173-0015, Japan; [b] Department of Biological Sciences, Tokyo Metropolitan University, Tokyo 192-0397, Japan; [c] Department of Molecular Pathophysiology, Institute of Advanced Medicine, Wakayama Medical University, Wakayama 641-8509, Japan; [d] Aging and Carcinogenesis, Tokyo Metropolitan Institute of Gerontology, Tokyo 173-0015, Japan

[1] Corresponding author: Akihito Ishigami, Ph.D.
Email: ishigami@tmig.or.jp

Supplementary Figures S1 to S7

Supplementary Tables S1-S2

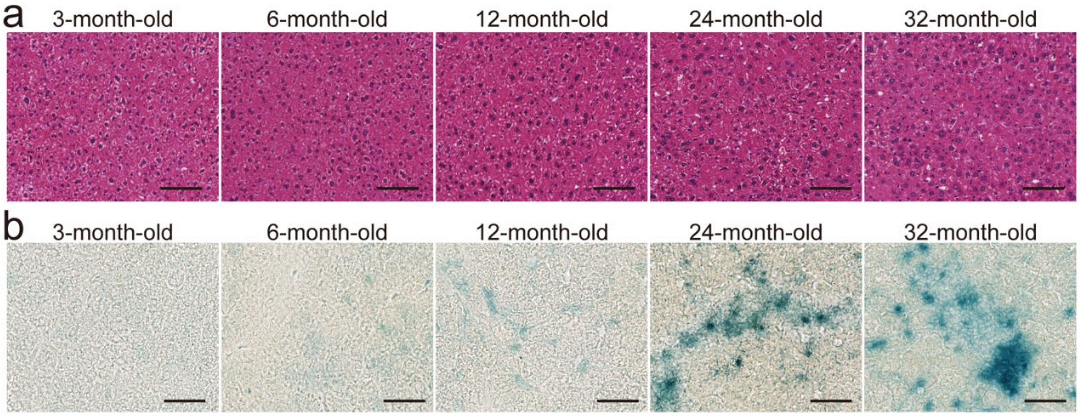

Supplementary Figure S1

Supplementary Figure S1. Histological evaluations on 3-, 6-, 12-, 24-, and 32-34-month-old mouse livers. (a) Representative images of hematoxylin and eosin (HE) staining on formalin fixed paraffin embedded sections of 3-, 6-, 12-, 24-, and 32-34-month-old mice. Scale bar = 100 μm. (b) Representative images of SA-β-gal staining on frozen sections of 3-, 6-, 12-, 24-, and 32-34-month-old mice. Scale bar = 50 μm.

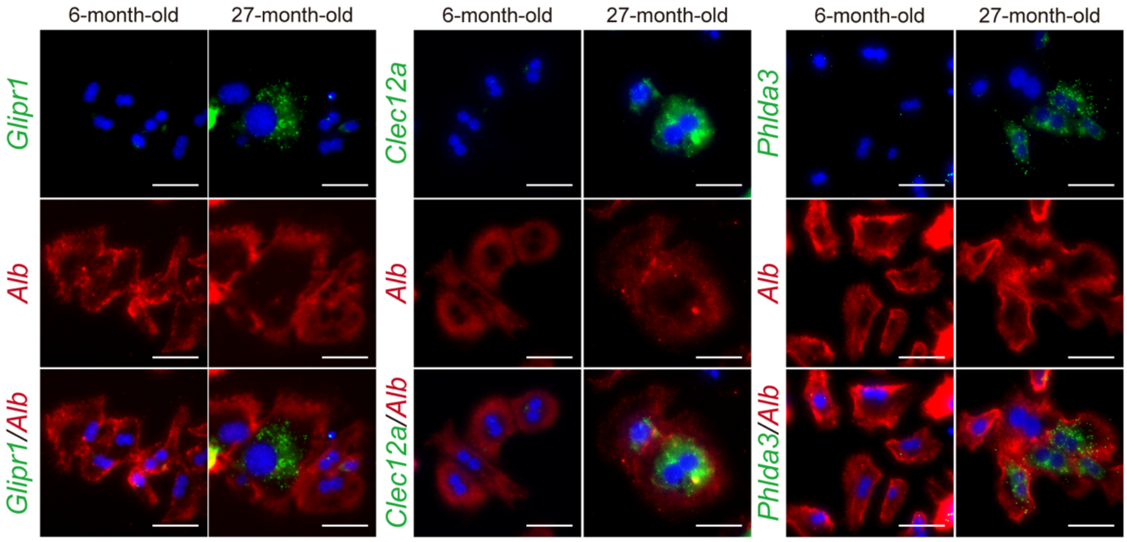

Supplementary Figure S2

Supplementary Figure S2. Fluorescence in situ hybridization (FISH) of *Glipr1*, *Clec12a*, and *Phlda3* in the primary hepatocytes of 6- and 27-month-old mice. Representative images stained for *Glipr1*, *Clec12a*, *Phlda3*, and *Alb* mRNA in 6- and 27-month-old primary mouse hepatocytes using the FISH. Green dots indicate *Glipr1*, *Clec12a*, and *Phlda3* mRNA signals. The *Alb* mRNA is shown as red dots. Nuclei were stained by DAPI (blue). Scale bar = 50 μm.

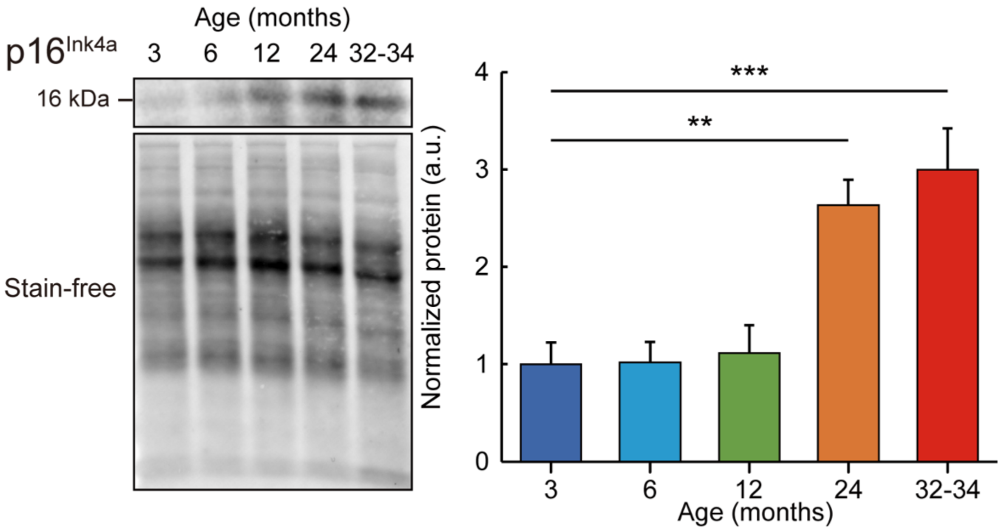

Supplementary Figure S3

Supplementary Figure S3. Protein levels of p16^{Ink4a} in the livers of 3-, 6-, 12-, 24-, and 32-34-month-old mice. Left panels are representative chemiluminescence and stain-free images. Uncropped images are shown in Supplementary Fig. S6f. Right bar plot shows relative protein levels in the livers of 3-, 6-, 12-, 24-, and 32-34-month-old mice (n=5, all age) relative to the livers of 3-month-old mice. Protein levels were normalized by whole protein levels detected by stain-free for each lane. Values are presented as the means ± SEM. The statistical analysis was performed using one-way ANOVA followed by Dunnett's post hoc test. ***p* < 0.01 and ****p* < 0.001. a.u., arbitrary units.

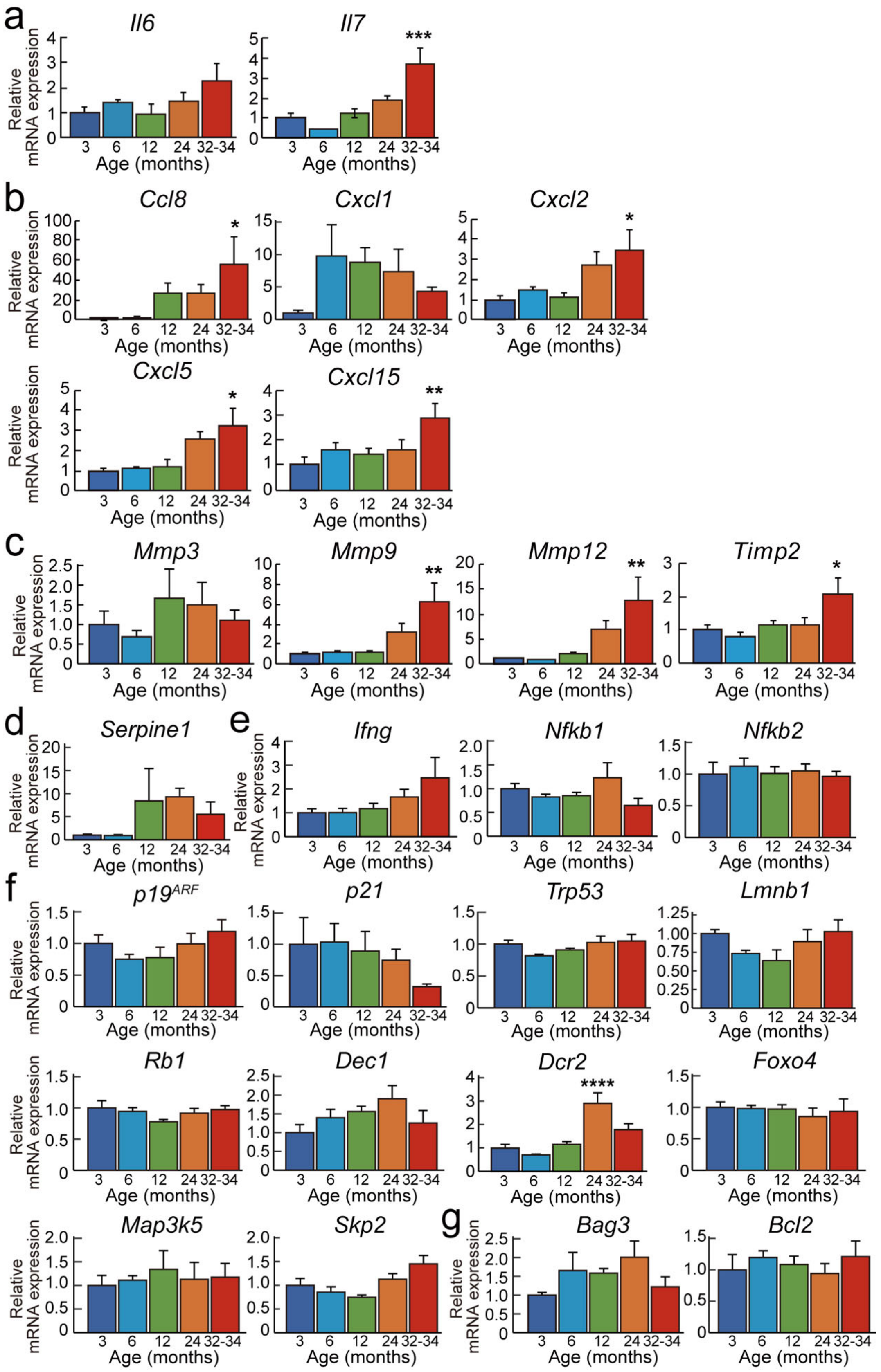

a
Il6
Il7
***
b
Ccl8
Cxcl1
Cxcl2
Cxcl5
Cxcl15
c
Mmp3
Mmp9
Mmp12
Timp2
d
Serpine1
e
Ifng
Nfkb1
Nfkb2
f
p19ARF
p21
Trp53
Lmnb1
Rb1
Dec1
Dcr2
****
Foxo4
Map3k5
Skp2
g
Bag3
Bcl2
Relative mRNA expression
Age (months)
3
6
12
24
32-34

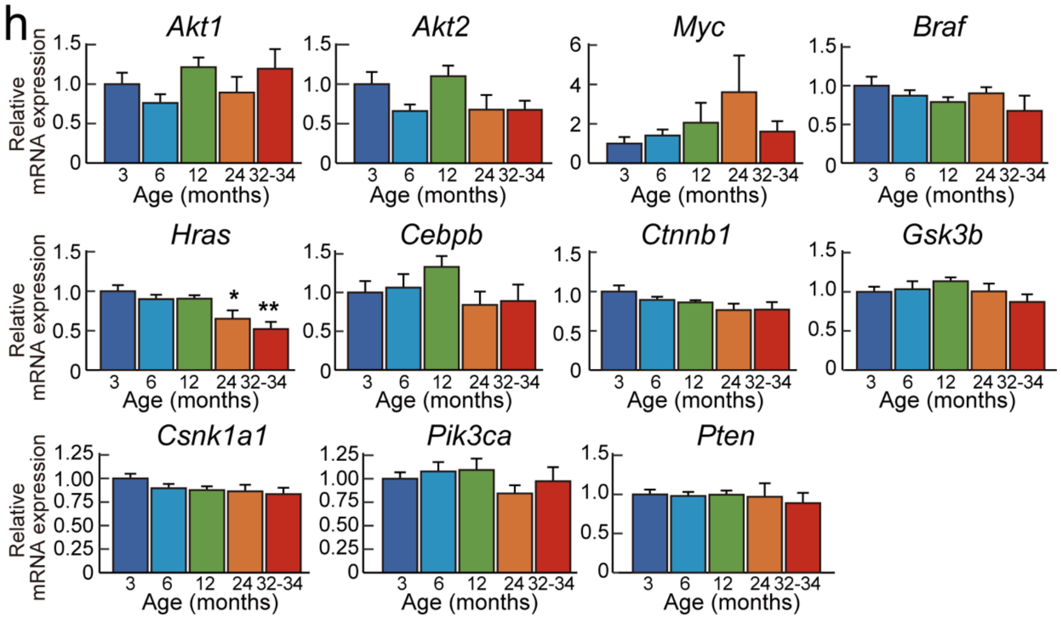

Supplementary Figure S4

Supplementary Figure S4. Expression levels of genes associated with senescence and cellular senescence in 3-, 6-, 12-, 24-, and 32-34-month-old mouse livers. RT-qPCR was performed to examined the mRNA expression levels of interleukins (a), chemokines (b), matrix proteinases and an inhibitor of matrix proteinases (c), peptidase inhibitors (d), other inflammatory factor (e), and genes associated with cell cycle arrest (f), anti-apoptosis (g), and tumorigenesis (h) in the 3-, 6-, 12-, 24-, and 32-34-month-old mouse livers (n=5, all ages). Bar plots show relative mRNA levels in the livers of mice at each age relative to the livers of 3-month-old mice. *β-actin* was used as the endogenous control gene. Values are presented as the means ± SEM. The statistical analysis was performed using the one-way ANOVA followed by Dunnett's post hoc test. *p<0.05, **p<0.01, ***p<0.001, and ****p < 0.0001. Il6, interleukin 6; Il7, interleukin 7; Ccl8, chemokine (C-C motif) ligand 8; Cxcl1, C-X-C motif chemokine ligand 1; Cxcl2, chemokine (C-X-C motif) ligand 2; Cxcl5, chemokine (C-X-C motif) ligand 5; Cxcl15, chemokine (C-X-C motif) ligand 15; Mmp3, matrix metallopeptidase 3; Mmp9, matrix metallopeptidase 9; Mmp12, matrix metallopeptidase 12; Timp2, TIMP metallopeptidase inhibitor 2; Serpine1, serine (or cysteine) peptidase inhibitor, clade E, member 1; Ifng, interferon gamma; Nfkb1, nuclear factor of kappa light polypeptide gene enhancer in B cells 1, p105; Nfkb2, nuclear factor of kappa light polypeptide gene enhancer in B cells 2, p49/p100; Trp53, transformation related protein 53; Lmnb1, lamin B1; Rb1, RB transcriptional corepressor 1; Dec1, basic helix-loop-helix family, member e40; Dcr2, tumor necrosis factor receptor superfamily, member 10b; Foxo4, forkhead box O4; Map3k5, mitogen-activated protein kinase 5; Skp2, S-phase kinase-associated protein 2; Bag3, BCL2-associated athanogene 3; Bcl2, B cell leukemia/lymphoma 2; Akt1, thymoma viral proto-oncogene 1; Akt2, thymoma viral proto-oncogene 2; Myc, myelocytomatosis oncogene; Braf, Braf transforming gene; Hras, Harvey rat sarcoma virus oncogene; Cebpb, CCAAT/enhancer binding protein (C/EBP) beta; Ctnnb1,

catenin (cadherin-associated protein), beta 1; Gsk3b, glycogen synthase kinase 3 beta; Csnk1a1, casein kinase 1, alpha 1; Pik3ca, phosphatidylinositol-4,5-bisphosphate 3-kinase catalytic subunit alpha; and Pten, phosphatase and tensin homolog.

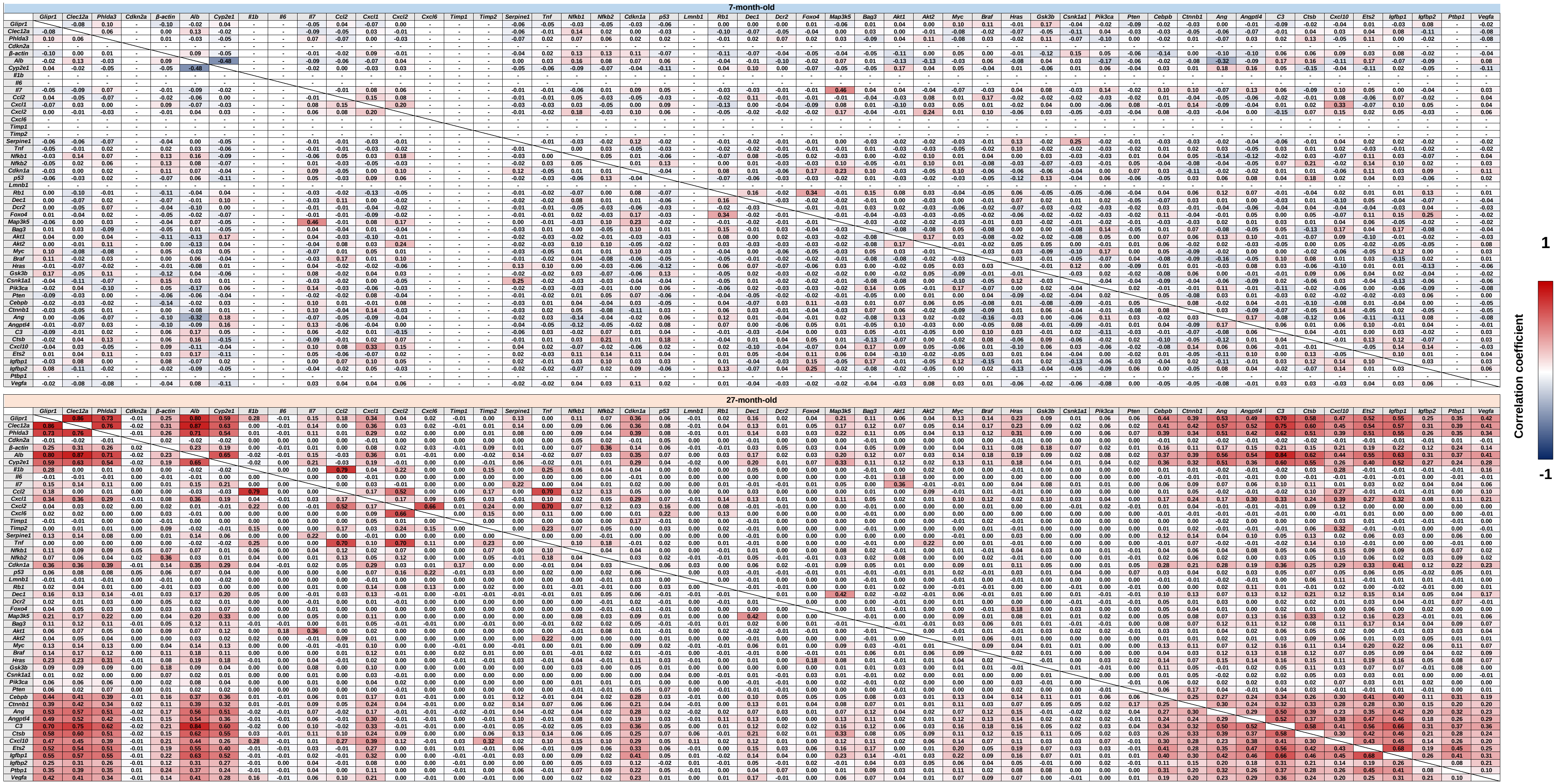

Supplementary Figure S5

Supplementary Figure S5. Correlation with expression patterns of genes associated with senescence and cellular senescence. In transcriptome data of 7-month-old and 27-month-old rat hepatocytes, correlation coefficients were calculated from the expression levels of genes included in Fig. 2a, Fig. 4, Supplementary Fig. S2, and the SenMayo gene set. The hyphen indicates a pair of genes whose correlation coefficient cannot be calculated by no expression data.

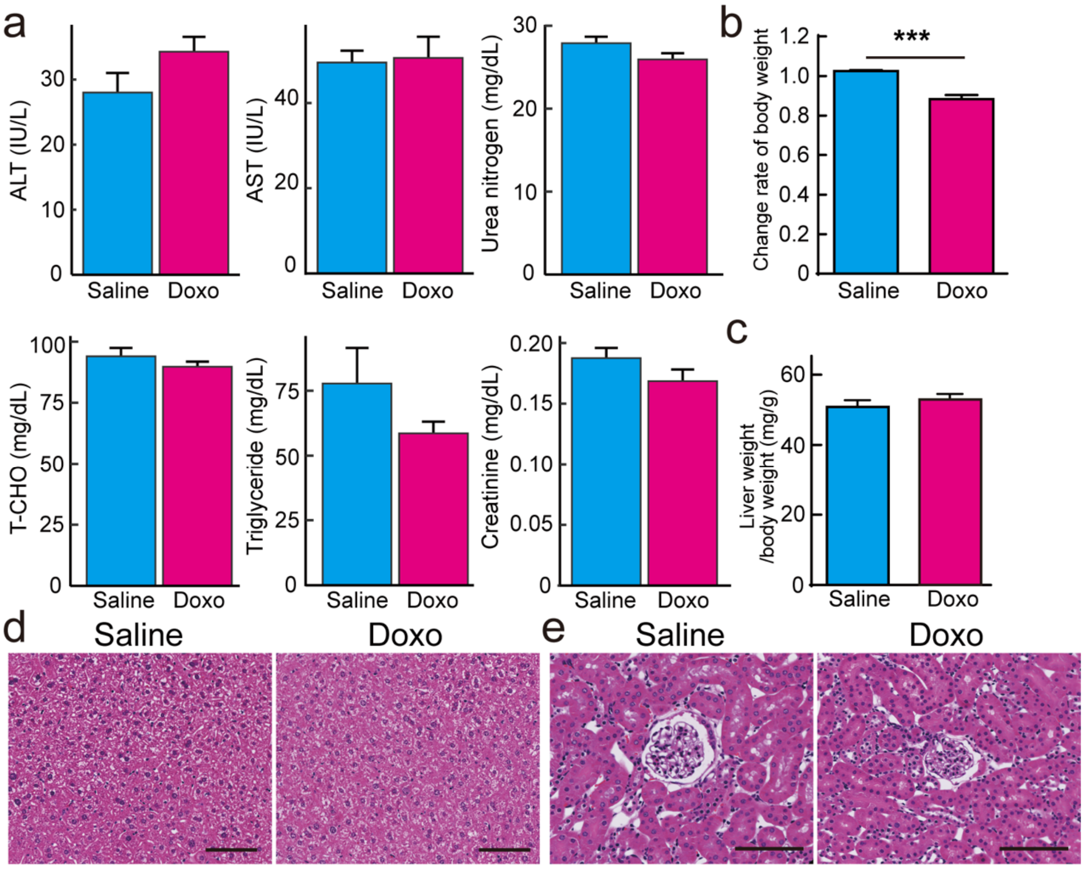

Supplementary Figure S6

Supplementary Figure S6. Biochemical and histological evaluations of mice administrated saline or doxorubicin. (a) Plasma levels of alanine aminotransferase (ALT), asparagine aminotransferase (AST), urea nitrogen, creatinine, total cholesterol (T-CHO), and triglycerides in mice administrated saline (n=8) or doxorubicin (Doxo) (n=7). (b) The change rate of body weight from the first *i.p* until sacrifice of mice. (c) The liver weight per body weight of mice administrated saline and doxorubicin when are sacrificed. (d-e) Representative images of HE staining on formalin fixed paraffin embedded sections of livers (d) and kidneys (e). Scale bar = 100 μm. Values are expressed as the mean ± SEM. The statistical analysis was performed using the two-tailed Welch's t-test. ***$p < 0.001$.

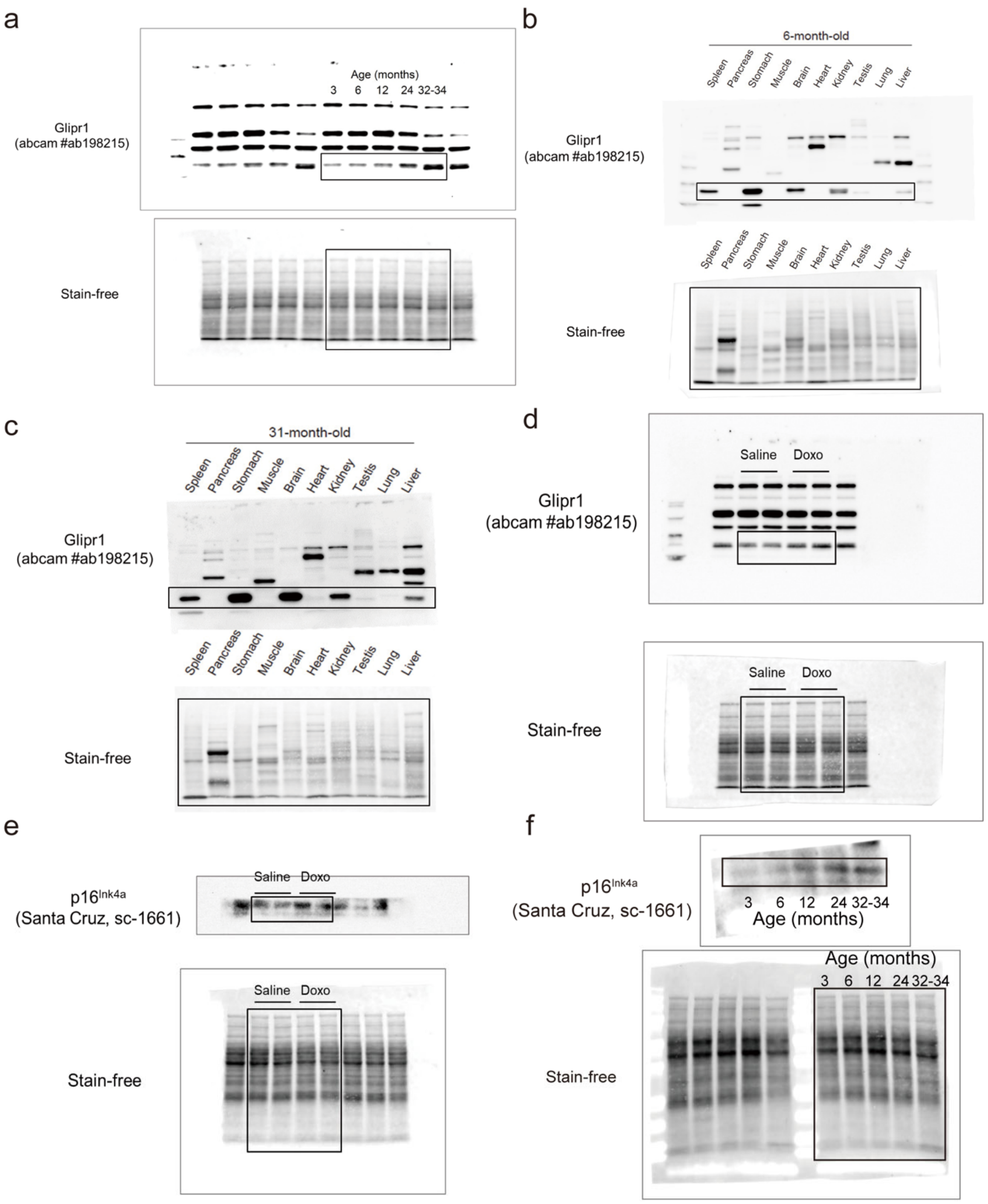

Supplementary Figure S7

Supplementary Figure S7. Original images of western blot and stain-free. The following panels are original images of western blot (top) and stain-free (bottom). Black line squares indicate the areas of cropped images shown in Fig. 2d (**a**), Fig. 3 (**b** and **c**), Fig. 6k (**d**), Fig. 6l (**e**), and Supplementary Fig. S2(**f**).

Supplemental Table S1 The percentage of positive cells of every genes in scRNA-seq transcriptome data of rat hepatocytes

Gene	Positive cells %		Difference (Old-Young)	Rank
	7-month-old	27-month-old		
LOC102552121	3.1%	82.8%	79.7	1
Cyp2c12	3.9%	82.2%	78.3	2
Vav1	42.7%	82.9%	40.1	3
Gdap1l1	42.7%	80.3%	37.5	4
Pear1	37.6%	74.8%	37.2	5
Klc3	42.7%	78.7%	36.0	6
Dgcr2	35.7%	70.7%	35.0	7
Syt5	41.6%	76.5%	34.9	8
Capn1	48.2%	82.7%	34.4	9
Trex1	45.5%	79.5%	34.0	10
Sharpin	49.4%	83.5%	34.0	11
LOC310926	18.0%	52.0%	33.9	12
Lrrc73	42.7%	76.4%	33.7	13
Tlcd1	43.9%	75.9%	31.9	14
Erich6	53.3%	84.9%	31.6	15
Akap8l	63.9%	95.4%	31.5	16
Slc25a29	45.1%	76.5%	31.4	17
Fam160b2	58.4%	89.8%	31.3	18
Hrasls5	43.9%	75.0%	31.1	19
Taf1c	52.2%	83.0%	30.8	20
Smg9	60.8%	91.4%	30.7	21
Bop1	63.1%	93.6%	30.5	22
Cldn14	65.5%	95.2%	29.7	23
Pelp1	51.0%	80.7%	29.7	24
Atp13a2	65.5%	95.1%	29.6	25
Wdr45	52.9%	82.3%	29.3	26
Rnf126	53.3%	82.1%	28.8	27
Klhl12	63.5%	92.2%	28.7	28
Arhgap9	51.0%	79.6%	28.6	29
Gipc2	68.2%	96.8%	28.6	30
Sostdc1	36.9%	65.2%	28.4	31
Lgi4	57.6%	85.9%	28.2	32
RGD1306063	65.1%	93.3%	28.2	33
Brms1	52.2%	80.3%	28.2	34
LOC687056	51.4%	79.3%	27.9	35
Fbxo16	66.7%	94.5%	27.8	36
Vamp2	49.4%	77.2%	27.8	37
LOC689316	8.6%	36.4%	27.7	38
LOC290876	65.9%	93.4%	27.6	39
Glipr1	58.4%	85.9%	27.5	40
Zfp518a	53.7%	81.1%	27.4	41
Vil1	38.8%	66.1%	27.3	42
Akr1b7	0.0%	27.3%	27.3	43
RGD1564534	68.6%	95.8%	27.1	44
Prl2a1	56.5%	83.5%	27.0	45
Ptdss1	51.8%	78.7%	26.9	46
Psmf1	69.0%	95.8%	26.8	47
Tmco4	68.6%	95.4%	26.8	48
Usp51	3.1%	29.8%	26.7	49
Rn28s	48.6%	75.3%	26.7	50
Malt1	6.3%	32.9%	26.6	51
Arvcf	70.2%	96.4%	26.2	52
Eef1akmt1	67.5%	93.6%	26.2	53
Igbp1	69.4%	95.5%	26.1	54
Clcn4	52.9%	78.7%	25.8	55
Nfkbib	59.6%	85.3%	25.7	56
LOC100362172	14.1%	39.5%	25.4	57
RGD1309106	71.8%	96.8%	25.0	58
Klhdc2	63.1%	88.2%	25.0	59
Clec12a	58.8%	83.9%	25.0	60

Tagln	43.5%	68.5%	25.0	61
Pcyox1l	69.8%	94.7%	24.9	62
Tctn2	55.3%	80.1%	24.8	63
Marc2	0.0%	24.8%	24.8	64
Slc7a13	70.2%	95.0%	24.8	65
LOC690813	2.4%	26.9%	24.6	66
Mthfr	4.3%	28.8%	24.5	67
Cd74	11.4%	35.7%	24.4	68
Grhl2	48.6%	72.8%	24.2	69
LOC367975	58.4%	82.6%	24.1	70
Taf10	71.0%	95.0%	24.1	71
Mroh7	40.4%	64.4%	24.0	72
Tlr6	71.4%	95.4%	24.0	73
Capn13	58.0%	81.7%	23.7	74
Wdfy1	63.1%	86.7%	23.5	75
Sstr3	71.4%	94.6%	23.2	76
Zfp717	9.4%	32.5%	23.0	77
Ccdc8	30.2%	52.8%	22.6	78
Shq1	60.8%	83.3%	22.5	79
Creb3	71.0%	93.4%	22.4	80
Zfp12	6.3%	28.5%	22.3	81
Gtf2ird2	9.4%	31.5%	22.1	82
LOC103691170	19.6%	41.2%	21.6	83
Atg7	38.4%	60.0%	21.5	84
LOC102557319	72.9%	94.4%	21.5	85
LOC102552172	9.0%	30.5%	21.4	86
RGD1309594	76.5%	97.8%	21.4	87
Mtm1	62.7%	83.6%	20.9	88
Ugt1a1	21.6%	42.4%	20.8	89
LOC108352109	3.9%	24.6%	20.7	90
RGD1306072	11.8%	32.5%	20.7	91
Drg1	73.7%	94.4%	20.7	92
Sept15	0.0%	20.6%	20.6	93
Akr1c12l1	56.9%	77.5%	20.6	94
Rpl37a	18.0%	38.6%	20.6	95
Ip6k2	61.2%	81.6%	20.4	96
Ttc9c	2.0%	22.4%	20.4	97
Zfp426	76.5%	96.8%	20.3	98
Ugt1a5	22.4%	42.6%	20.3	99
Orc4	75.3%	95.5%	20.2	100
LOC100363112	2.7%	22.9%	20.2	101
LOC108352810	4.3%	24.3%	20.0	102
Zfp1	11.8%	31.6%	19.8	103
LOC102554055	2.7%	22.4%	19.6	104
Limd1	77.6%	97.2%	19.6	105
LOC501180	76.1%	95.6%	19.5	106
Nmi	66.3%	85.7%	19.4	107
Mt1m	16.1%	35.4%	19.3	108
Ugt1a9	23.9%	43.1%	19.2	109
Serpinb10	64.7%	83.9%	19.1	110
LOC108351425	13.3%	32.5%	19.1	111
Dffa	76.1%	95.0%	19.0	112
Abtb1	78.0%	96.7%	18.7	113
Rfxank	72.9%	91.6%	18.7	114
RGD1564325	16.5%	35.1%	18.6	115
Dnajb13	10.6%	29.1%	18.5	116
Sarnp	78.8%	97.3%	18.5	117
Ubtd1	58.8%	77.0%	18.2	118
Oip5	40.4%	58.4%	18.0	119
Coro2a	7.5%	25.3%	17.9	120
Ugt1a3	23.9%	41.8%	17.9	121
Ugt1a8	24.3%	42.1%	17.8	122
Lrrc41	80.4%	98.1%	17.7	123
Med22	58.8%	76.5%	17.7	124
Slc37a3	32.2%	49.7%	17.6	125
Myom3	19.6%	37.2%	17.6	126

LOC100158225	9.0%	26.5%	17.5	127
Rnf225	5.5%	22.9%	17.5	128
Foxp1	78.0%	95.4%	17.4	129
Prrc1	66.3%	83.6%	17.3	130
LOC690435	1.6%	18.9%	17.3	131
Cxadrl1	80.4%	97.7%	17.3	132
Itm2c	82.4%	99.4%	17.0	133
Slc25a40	73.7%	90.5%	16.8	134
Ilk	63.5%	80.2%	16.6	135
Slc26a8	8.2%	24.9%	16.6	136
Cdc6	3.5%	20.0%	16.5	137
Smpd2	80.4%	96.8%	16.4	138
Comt	16.5%	32.9%	16.4	139
Cdv3	82.0%	98.3%	16.4	140
Ctnnbl1	47.8%	64.2%	16.3	141
Pex11b	31.0%	47.3%	16.3	142
Luzp2	48.2%	64.5%	16.3	143
RGD1309534	67.8%	84.1%	16.2	144
LOC499407	2.4%	18.3%	16.0	145
Wfdc21	38.8%	54.8%	15.9	146
MGC114483	76.9%	92.7%	15.9	147
Ugt1a7c	27.1%	42.8%	15.8	148
Mrpl1	81.2%	96.8%	15.6	149
Sertad1	68.6%	84.3%	15.6	150
Zfp82	23.5%	39.0%	15.5	151
Rnf10	68.6%	84.1%	15.5	152
LOC102555751	1.6%	16.9%	15.3	153
Srpk1	48.2%	63.3%	15.1	154
Odf2	9.8%	24.8%	15.0	155
Ugt1a2	26.3%	41.2%	15.0	156
Uap1l1	82.0%	96.9%	14.9	157
Mt2A	45.9%	60.6%	14.7	158
Imp4	81.6%	96.1%	14.5	159
LOC685963	9.0%	23.4%	14.4	160
Xirp2	83.1%	97.5%	14.4	161
Jund	8.6%	22.9%	14.2	162
Gsdma	12.5%	26.8%	14.2	163
Muc15	70.2%	84.4%	14.2	164
Mitf	4.7%	18.9%	14.2	165
Timm8a1	63.9%	78.0%	14.1	166
Psmd9	68.2%	82.3%	14.0	167
LOC103690015	18.0%	32.1%	14.0	168
Atxn2l	79.6%	93.6%	14.0	169
Hspa1b	7.1%	21.0%	14.0	170
Peo1	7.5%	21.3%	13.9	171
Myadml2	11.4%	25.3%	13.9	172
LOC100362339	16.9%	30.7%	13.8	173
Efcab2	2.4%	16.1%	13.7	174
Map2k5	69.4%	83.1%	13.6	175
Sept7	0.0%	13.6%	13.6	176
Zfp868	65.9%	79.5%	13.6	177
Slfn5	2.7%	16.3%	13.6	178
Spns1	69.0%	82.5%	13.5	179
Raly	82.4%	95.8%	13.4	180
LOC102552179	11.4%	24.8%	13.4	181
LOC682793	6.3%	19.7%	13.4	182
Rpl35a	5.9%	19.2%	13.3	183
Ugt1a6	34.9%	48.2%	13.3	184
Dynll1	84.7%	98.0%	13.3	185
Zfp69	7.1%	20.3%	13.2	186
LOC691392	13.7%	26.9%	13.2	187
LOC108349632	2.7%	15.9%	13.2	188
Pou1f1	85.5%	98.6%	13.2	189
Gdf15	7.5%	20.5%	13.1	190
Vps18	80.8%	93.8%	13.1	191
Asb5	83.9%	97.0%	13.0	192

Map6	71.8%	84.7%	13.0	193
LOC303448	11.0%	23.8%	12.8	194
Vegfb	9.4%	22.2%	12.8	195
Rpl38	17.3%	29.8%	12.6	196
LOC299282	16.5%	29.0%	12.5	197
Cdh19	7.1%	19.6%	12.5	198
Rnf4	63.5%	76.0%	12.5	199
Gstp1	0.0%	12.5%	12.5	200
Timm21	83.1%	95.6%	12.5	201
LOC102548155	7.5%	19.9%	12.5	202
Itgb3bp	73.7%	86.2%	12.4	203
Cdkl1	1.6%	13.9%	12.3	204
Yipf1	85.5%	97.8%	12.3	205
Wwc1	8.6%	20.9%	12.2	206
Prlr	3.1%	15.3%	12.2	207
LOC100912132	22.4%	34.5%	12.2	208
Scd	46.3%	58.4%	12.1	209
Semg1	45.9%	58.0%	12.1	210
Angptl8	30.2%	42.2%	12.0	211
Gle1	11.4%	23.3%	12.0	212
Acot9	49.0%	60.9%	11.9	213
Lrrc19	2.4%	14.2%	11.9	214
LOC103692489	5.1%	16.9%	11.8	215
Prr5l	86.7%	98.5%	11.8	216
Ifi47	0.4%	12.1%	11.7	217
Sh3rf2	28.2%	39.8%	11.6	218
Lama1	11.4%	22.9%	11.5	219
LOC103690508	2.4%	13.8%	11.5	220
LOC108352299	2.7%	14.1%	11.4	221
LOC102551949	2.0%	13.3%	11.4	222
Rasgrp3	5.5%	16.9%	11.4	223
Jun	32.9%	44.3%	11.3	224
LOC108348509	11.4%	22.7%	11.3	225
LOC108352121	2.0%	13.3%	11.3	226
LOC100366054	0.0%	11.3%	11.3	227
Slc46a3	87.5%	98.7%	11.3	228
Mob1a	43.9%	55.2%	11.2	229
Zbtb24	3.9%	15.1%	11.2	230
Apaf1	2.4%	13.5%	11.2	231
LOC100360645	27.1%	38.2%	11.2	232
LOC690468	18.4%	29.6%	11.1	233
LOC108348502	1.2%	12.3%	11.1	234
LOC102550111	3.9%	15.0%	11.1	235
LOC100909912	19.6%	30.7%	11.1	236
LOC498826	2.4%	13.4%	11.1	237
Armc7	5.1%	16.1%	11.0	238
Plekhs1	5.1%	16.1%	11.0	239
Dusp1	14.5%	25.5%	11.0	240
LOC108352688	9.8%	20.8%	11.0	241
Prickle3	85.5%	96.4%	10.9	242
LOC102548393	2.0%	12.9%	10.9	243
Wdr5	86.3%	97.1%	10.8	244
LOC108349602	19.2%	30.1%	10.8	245
Cpm	5.5%	16.3%	10.8	246
N4bp2l1	7.8%	18.6%	10.8	247
Tmem233	2.7%	13.4%	10.7	248
Prune2	2.4%	13.0%	10.7	249
Cc2d1a	26.7%	37.3%	10.7	250
RGD1564062	9.8%	20.5%	10.7	251
Lix1l	87.8%	98.5%	10.6	252
Fam102b	3.5%	14.1%	10.5	253
LOC103693430	3.5%	14.1%	10.5	254
Srgn	3.1%	13.7%	10.5	255
Ccdc134	10.2%	20.7%	10.5	256
LOC108349221	1.6%	12.1%	10.5	257
LOC100909904	3.5%	14.0%	10.5	258

Slc25a25	15.3%	25.7%	10.4	259
LOC294154	89.0%	99.4%	10.4	260
LOC100912502	8.2%	18.6%	10.4	261
Pgrmc2	89.0%	99.4%	10.3	262
LOC108351703	10.2%	20.5%	10.3	263
Onecut1	2.4%	12.5%	10.2	264
Bmi1	78.0%	88.2%	10.1	265
LOC102546693	2.0%	12.1%	10.1	266
LOC100362967	25.1%	35.2%	10.1	267
Plekhj1	78.4%	88.5%	10.1	268
Adck5	87.5%	97.4%	10.0	269
Spock2	3.5%	13.5%	10.0	270
Zfp90	2.4%	12.3%	10.0	271
LOC103693494	4.3%	14.2%	9.9	272
Fam65b	20.8%	30.7%	9.9	273
LOC108353525	10.2%	20.1%	9.9	274
Pik3r3	1.2%	11.0%	9.8	275
Phlda3	73.3%	83.1%	9.7	276
Rhebl1	2.4%	12.0%	9.6	277
LOC100910721	8.6%	18.1%	9.5	278
Hbb	0.0%	9.5%	9.5	279
LOC108350845	7.8%	17.3%	9.5	280
Fbxl13	78.4%	87.9%	9.5	281
RGD1563482	2.4%	11.8%	9.5	282
Ube2n	80.0%	89.4%	9.4	283
Sec11a	87.5%	96.8%	9.4	284
LOC108348777	0.0%	9.3%	9.3	285
LOC691716	10.6%	19.8%	9.2	286
Zbed5	11.8%	20.9%	9.2	287
Rpl39	9.0%	18.1%	9.1	288
LOC100359916	4.7%	13.8%	9.1	289
Eif3l	70.6%	79.7%	9.1	290
Gins2	3.5%	12.6%	9.1	291
LOC102551514	3.1%	12.2%	9.1	292
Rmdn2	77.6%	86.7%	9.1	293
Angel2	18.8%	27.9%	9.1	294
Emc1	13.7%	22.8%	9.1	295
Laptm5	83.5%	92.6%	9.0	296
Ppp1r3b	1.2%	10.2%	9.0	297
Zfp709	3.5%	12.5%	8.9	298
Dusp6	5.9%	14.8%	8.9	299
LOC100361180	26.7%	35.6%	8.9	300
Rnf135	4.7%	13.6%	8.9	301
Bst2	25.5%	34.4%	8.9	302
Sult2a6	51.0%	59.8%	8.8	303
LOC100910973	0.8%	9.6%	8.8	304
Fos	6.7%	15.3%	8.7	305
Asah1	89.8%	98.5%	8.7	306
Ncl	85.5%	94.2%	8.7	307
Faim	10.2%	18.8%	8.6	308
LOC103692397	0.4%	9.0%	8.6	309
Ctla2a	3.1%	11.7%	8.5	310
Prob1	1.2%	9.7%	8.5	311
LOC102549703	7.8%	16.3%	8.5	312
LOC108351058	15.3%	23.7%	8.4	313
Etv6	81.2%	89.6%	8.4	314
Vegfa	9.8%	18.2%	8.4	315
LOC685431	13.7%	22.1%	8.4	316
Ccl5	0.4%	8.8%	8.4	317
Agtrap	3.5%	11.9%	8.4	318
LOC100364116	38.4%	46.8%	8.3	319
Jakmip1	83.1%	91.4%	8.3	320
LOC685187	3.1%	11.4%	8.3	321
Crp	7.5%	15.7%	8.2	322
Chrna1	7.1%	15.3%	8.2	323
Iqsec3	6.7%	14.9%	8.2	324

LOC100359649	3.5%	11.7%	8.1	325
Sult2a2	1.6%	9.7%	8.1	326
Cacna1c	21.2%	29.3%	8.1	327
Ipp	6.3%	14.3%	8.0	328
LOC103693120	0.8%	8.8%	8.0	329
Hba1	0.0%	8.0%	8.0	330
RGD1563300	11.0%	18.9%	8.0	331
Anp32a	11.0%	18.9%	8.0	332
Lrrc14	89.0%	97.0%	7.9	333
Qsox2	9.4%	17.3%	7.9	334
RatNP-3b	0.8%	8.7%	7.9	335
Arhgdia	89.0%	96.9%	7.9	336
LOC102547564	2.4%	10.2%	7.8	337
LOC108351137	9.8%	17.6%	7.8	338
Creg2	2.0%	9.7%	7.7	339
March2	5.5%	13.2%	7.7	340
Midn	2.0%	9.6%	7.6	341
LOC108352164	0.4%	8.0%	7.6	342
LOC100911891	0.4%	8.0%	7.6	343
LOC100911320	3.9%	11.4%	7.5	344
LOC502923	3.9%	11.4%	7.5	345
RT1-Bb	2.4%	9.8%	7.5	346
Akap10	2.0%	9.4%	7.5	347
Acsm2	1.6%	9.0%	7.5	348
Ftsj1	3.5%	11.0%	7.4	349
LOC102546787	2.7%	10.2%	7.4	350
LOC365238	2.7%	10.2%	7.4	351
Rfc5	23.5%	30.9%	7.4	352
Harbi1	3.5%	10.9%	7.3	353
Adam30	2.7%	10.1%	7.3	354
LOC103690996	12.5%	19.8%	7.3	355
Itga4	3.5%	10.8%	7.3	356
Zfp207	86.3%	93.5%	7.3	357
Acer2	22.7%	30.0%	7.2	358
Ccl24	2.7%	9.9%	7.2	359
Ttc29	89.0%	96.2%	7.1	360
Bcl11a	1.2%	8.3%	7.1	361
RT1-Ba	1.2%	8.3%	7.1	362
Proser3	15.7%	22.8%	7.1	363
Mccc2	52.2%	59.2%	7.1	364
Scarb2	76.1%	83.1%	7.1	365
LOC103691944	2.4%	9.4%	7.0	366
Ndel1	10.2%	17.2%	7.0	367
LOC103694060	0.8%	7.8%	7.0	368
Rer1	87.5%	94.4%	7.0	369
Gsta3	3.5%	10.5%	6.9	370
Eps15l1	7.5%	14.4%	6.9	371
Akip1	7.1%	14.0%	6.9	372
RGD1563601	13.7%	20.6%	6.9	373
LOC103691053	0.8%	7.7%	6.9	374
RGD1563861	20.8%	27.7%	6.9	375
Rwdd2a	2.4%	9.2%	6.8	376
LOC100359421	6.3%	13.1%	6.8	377
Casp8	1.2%	8.0%	6.8	378
Ankrd39	7.5%	14.2%	6.8	379
Cyp4f17	23.9%	30.7%	6.8	380
LOC103691631	2.4%	9.1%	6.8	381
Junb	18.0%	24.8%	6.7	382
Exd1	1.2%	7.9%	6.7	383
LOC108353795	20.4%	27.1%	6.7	384
RGD1561618	3.5%	10.2%	6.7	385
Echs1	86.7%	93.4%	6.7	386
LOC108349033	7.1%	13.7%	6.7	387
LOC100360514	3.1%	9.8%	6.6	388
Htra4	2.4%	9.0%	6.6	389
Msr1	2.0%	8.6%	6.6	390

Gcat	80.0%	86.6%	6.6	391
RGD1562381	3.9%	10.5%	6.6	392
Vbp1	1.2%	7.7%	6.5	393
LOC103693581	5.1%	11.6%	6.5	394
Sept2	0.0%	6.5%	6.5	395
Zfp623	8.2%	14.7%	6.5	396
Ccl28	49.0%	55.5%	6.5	397
RGD1564574	6.3%	12.7%	6.4	398
Tfb1m	50.6%	57.0%	6.4	399
Dock2	5.1%	11.4%	6.3	400
Pnpla5	0.8%	7.1%	6.3	401
Pla2g4f	4.7%	11.0%	6.3	402
ATP8	0.0%	6.3%	6.3	403
LOC108352582	0.0%	6.3%	6.3	404
LOC108350152	11.8%	18.1%	6.3	405
LOC103690548	5.9%	12.2%	6.3	406
LOC691807	79.6%	85.9%	6.2	407
Kcng4	23.9%	30.1%	6.2	408
Tor3a	6.7%	12.9%	6.2	409
Abcg3l3	2.4%	8.6%	6.2	410
Cyp3a9	68.6%	74.8%	6.2	411
RGD1562415	8.2%	14.4%	6.2	412
Rwdd4	81.6%	87.7%	6.1	413
LOC100912756	2.0%	8.1%	6.1	414
RGD1562844	0.8%	6.9%	6.1	415
Zfp335	4.3%	10.4%	6.1	416
RT1-Db1	2.4%	8.4%	6.0	417
LOC103691183	3.9%	9.9%	6.0	418
RGD1310212	9.4%	15.3%	5.9	419
LOC103690779	5.1%	11.0%	5.9	420
Dnaaf2	6.3%	12.2%	5.9	421
Efcc1	0.8%	6.6%	5.9	422
Prkaca	8.6%	14.5%	5.8	423
Selenow	1.2%	7.0%	5.8	424
Htr5b	0.8%	6.6%	5.8	425
LOC102551299	0.8%	6.6%	5.8	426
Rpp38	88.2%	94.0%	5.8	427
RGD1565117	4.3%	10.1%	5.8	428
LOC100360647	33.3%	39.1%	5.8	429
LOC100134871	0.0%	5.8%	5.8	430
RGD1310507	31.4%	37.1%	5.7	431
Rfc4	71.8%	77.5%	5.7	432
Ldah	25.5%	31.2%	5.7	433
LOC100361079	20.8%	26.5%	5.7	434
Iah1	91.0%	96.6%	5.7	435
Cryab	2.7%	8.4%	5.6	436
Ksr1	2.0%	7.6%	5.6	437
Pdk4	1.6%	7.2%	5.6	438
Neu1	1.2%	6.8%	5.6	439
LOC102556424	1.2%	6.8%	5.6	440
Lnc001	1.2%	6.8%	5.6	441
RGD1563294	9.4%	15.0%	5.6	442
RGD1560821	0.8%	6.4%	5.6	443
Hmg1l1	9.0%	14.6%	5.6	444
Mid1ip1	15.3%	20.9%	5.6	445
Kcnk5	6.7%	12.2%	5.6	446
LOC108353801	2.4%	7.9%	5.6	447
LOC103689961	1.2%	6.7%	5.5	448
Hnrnpa1	16.9%	22.4%	5.5	449
Slc11a2	54.1%	59.6%	5.5	450
LOC108350501	6.3%	11.8%	5.5	451
LOC100911428	1.2%	6.6%	5.5	452
RGD1559808	5.1%	10.6%	5.5	453
Inca1	0.0%	5.4%	5.4	454
Cmip	7.1%	12.5%	5.4	455
Lcor	2.4%	7.8%	5.4	456

LOC108349682	18.8%	24.2%	5.4	457
Zbtb16	2.0%	7.4%	5.4	458
LOC103695017	0.8%	6.2%	5.4	459
Kdm6b	0.0%	5.4%	5.4	460
RGD1564744	7.8%	13.2%	5.3	461
Nlrc4	2.4%	7.7%	5.3	462
Snx10	2.0%	7.3%	5.3	463
LOC103694169	22.7%	28.1%	5.3	464
LOC100909878	26.3%	31.6%	5.3	465
RGD1565653	5.1%	10.4%	5.3	466
LOC103693593	0.8%	6.1%	5.3	467
Gng4	0.8%	6.1%	5.3	468
LOC100910714	9.0%	14.3%	5.3	469
Nup62	82.7%	88.0%	5.3	470
LOC689271	1.6%	6.8%	5.2	471
RragB	9.4%	14.6%	5.2	472
LOC108352450	0.4%	5.6%	5.2	473
LOC102557529	0.4%	5.6%	5.2	474
Lipogenin	3.5%	8.7%	5.2	475
Tmem175	3.5%	8.7%	5.2	476
LOC690246	3.5%	8.7%	5.2	477
Plagl1	85.9%	91.0%	5.2	478
Sugp2	4.3%	9.4%	5.1	479
Neu3	3.9%	9.0%	5.1	480
Aprt	1.2%	6.2%	5.1	481
Slc22a7	1.2%	6.2%	5.1	482
LOC108350414	0.0%	5.0%	5.0	483
Ska3	3.9%	9.0%	5.0	484
LOC300303	17.3%	22.2%	5.0	485
LOC102548333	2.7%	7.7%	4.9	486
LOC108351743	2.7%	7.7%	4.9	487
Gfi1	2.4%	7.3%	4.9	488
Rph3al	47.1%	52.0%	4.9	489
LOC100911107	0.4%	5.3%	4.9	490
LOC108353549	0.0%	4.9%	4.9	491
LOC103693045	3.5%	8.4%	4.9	492
Ttc38	40.8%	45.6%	4.9	493
Isg15	10.6%	15.4%	4.8	494
Gan	2.0%	6.8%	4.8	495
LOC498555	26.3%	31.1%	4.8	496
LOC103691207	0.8%	5.6%	4.8	497
LOC102555814	0.0%	4.8%	4.8	498
LOC103694442	3.5%	8.3%	4.8	499
LOC100364265	2.7%	7.5%	4.8	500
Fam29a	1.6%	6.3%	4.7	501
Cyb5r1	1.6%	6.3%	4.7	502
Gstt1	92.5%	97.3%	4.7	503
LOC103692831	4.3%	9.0%	4.7	504
LOC102557505	2.7%	7.4%	4.7	505
LOC102556416	2.4%	7.0%	4.7	506
RGD1563917	10.6%	15.3%	4.7	507
Galk1	93.7%	98.4%	4.7	508
Pnpla3	8.6%	13.3%	4.6	509
RGD1306502	4.3%	9.0%	4.6	510
Itgb2	4.7%	9.3%	4.6	511
A1bg	0.0%	4.6%	4.6	512
LOC102551940	6.7%	11.2%	4.5	513
LOC100911361	5.9%	10.4%	4.5	514
B3gat3	1.6%	6.1%	4.5	515
Sh3bgrl2	1.6%	6.1%	4.5	516
RGD1564548	1.6%	6.1%	4.5	517
Rhpn2	12.9%	17.4%	4.5	518
Rsu1	3.9%	8.4%	4.5	519
LOC100360791	32.9%	37.4%	4.5	520
Usf2	3.5%	8.0%	4.5	521
Zfp566	3.5%	8.0%	4.5	522

RGD1560633	2.7%	7.2%	4.4	523
Natd1	1.2%	5.6%	4.4	524
RGD1560010	0.8%	5.2%	4.4	525
LOC102549542	0.4%	4.8%	4.4	526
Ccl4	0.0%	4.4%	4.4	527
Rps20	45.5%	49.9%	4.4	528
Phc3	5.5%	9.8%	4.3	529
LOC102550744	0.8%	5.1%	4.3	530
Id2	4.7%	9.0%	4.3	531
Polr2l	21.2%	25.5%	4.3	532
Esm1	29.4%	33.7%	4.3	533
LOC108348931	0.0%	4.3%	4.3	534
Hbb-b1	0.0%	4.3%	4.3	535
Atf3	45.1%	49.4%	4.3	536
Chrna5	2.0%	6.2%	4.3	537
RT1-Da	1.6%	5.8%	4.3	538
Myl6b	26.3%	30.5%	4.3	539
LOC108352560	0.8%	5.0%	4.3	540
Elovl6	0.4%	4.6%	4.2	541
Itga9	0.0%	4.2%	4.2	542
LOC103690129	7.5%	11.7%	4.2	543
LOC108348938	5.9%	10.1%	4.2	544
Tmsb10	4.7%	8.9%	4.2	545
Sept9	0.4%	4.6%	4.2	546
Tuba1a	0.4%	4.6%	4.2	547
RT1-A1	21.2%	25.3%	4.2	548
LOC102557028	0.0%	4.2%	4.2	549
Rilp	2.7%	6.9%	4.1	550
Catsperg	5.9%	10.0%	4.1	551
Trpt1	4.3%	8.4%	4.1	552
Scml4	4.3%	8.4%	4.1	553
LOC100365570	33.3%	37.4%	4.1	554
Rfk	16.5%	20.5%	4.1	555
Zfp36l2	3.5%	7.6%	4.1	556
LOC102554047	7.5%	11.5%	4.1	557
Snrpf	2.4%	6.4%	4.0	558
Fam46c	2.4%	6.4%	4.0	559
Socs2	6.3%	10.3%	4.0	560
Glul	14.1%	18.1%	4.0	561
Fancm	1.6%	5.6%	4.0	562
Rnf43	5.5%	9.5%	4.0	563
LOC102555980	1.2%	5.2%	4.0	564
Ccr5	0.8%	4.8%	4.0	565
Dazap1	4.3%	8.3%	4.0	566
Orai2	0.0%	4.0%	4.0	567
Rac2	0.0%	4.0%	4.0	568
LOC103693700	3.5%	7.5%	4.0	569
Cyr61	3.1%	7.1%	4.0	570
Phf20	6.7%	10.6%	4.0	571
LOC102547955	2.4%	6.3%	4.0	572
Atp6ap1	2.0%	5.9%	4.0	573
LOC103692946	1.6%	5.5%	3.9	574
Tspan7	1.6%	5.5%	3.9	575
Gadd45g	13.7%	17.7%	3.9	576
LOC317588	5.1%	9.0%	3.9	577
LOC103692555	0.8%	4.7%	3.9	578
LOC102550851	8.6%	12.5%	3.9	579
LOC100362113	15.3%	19.2%	3.9	580
Megf11	14.9%	18.8%	3.9	581
Lin28a	1.6%	5.4%	3.9	582
LOC102550180	1.2%	5.0%	3.9	583
March8	0.8%	4.6%	3.9	584
Fgl2	0.0%	3.8%	3.8	585
Ceacam9	2.4%	6.2%	3.8	586
Gpi	92.9%	96.7%	3.8	587
E230034O05Rik	0.4%	4.2%	3.8	588

Cotl1	0.0%	3.8%	3.8	589
Edil3	0.0%	3.8%	3.8	590
Tmc5	0.0%	3.8%	3.8	591
Dnase1l3	0.0%	3.8%	3.8	592
LOC108349691	3.9%	7.7%	3.8	593
LOC102553278	3.5%	7.3%	3.7	594
Braf	5.9%	9.6%	3.7	595
Ppm1a	26.3%	30.0%	3.7	596
LOC100911830	0.8%	4.5%	3.7	597
Cd40	0.0%	3.7%	3.7	598
LOC102551815	2.0%	5.6%	3.6	599
LOC685619	9.8%	13.4%	3.6	600
Tceb1	5.1%	8.7%	3.6	601
Galnt7	0.4%	4.0%	3.6	602
Scd2	0.4%	4.0%	3.6	603
Coro1a	0.0%	3.6%	3.6	604
Rab36	0.0%	3.6%	3.6	605
Tob1	20.8%	24.4%	3.6	606
LOC103694354	3.5%	7.1%	3.6	607
LOC103694079	3.1%	6.7%	3.6	608
Rpp14	18.4%	22.0%	3.6	609
Zfp605	1.6%	5.1%	3.5	610
LOC108352152	1.6%	5.1%	3.5	611
LOC102556590	1.6%	5.1%	3.5	612
Akr1c1	18.0%	21.6%	3.5	613
Rn18s	96.5%	100.0%	3.5	614
Klf10	4.3%	7.8%	3.5	615
Lyve1	0.0%	3.5%	3.5	616
Sec14l3	0.0%	3.5%	3.5	617
Mrc1	0.0%	3.5%	3.5	618
Prf1	0.0%	3.5%	3.5	619
LOC688981	3.5%	7.0%	3.5	620
Rtp3	15.3%	18.8%	3.5	621
LOC108348789	1.2%	4.6%	3.5	622
LOC102552204	1.2%	4.6%	3.5	623
Tgm4	0.0%	3.4%	3.4	624
LOC108351482	12.2%	15.6%	3.4	625
Dgke	2.7%	6.2%	3.4	626
Sh3bp5	6.7%	10.1%	3.4	627
Piwil1	1.6%	5.0%	3.4	628
LOC108352128	0.8%	4.2%	3.4	629
Dear	0.8%	4.2%	3.4	630
Bc1	0.4%	3.8%	3.4	631
Ifitm1	0.4%	3.8%	3.4	632
Pik3r1	12.5%	15.9%	3.4	633
Abcc3	0.0%	3.4%	3.4	634
Hba2	0.0%	3.4%	3.4	635
LOC100910095	0.0%	3.4%	3.4	636
LOC108351532	0.0%	3.4%	3.4	637
LOC108348926	0.0%	3.4%	3.4	638
Hdgfl1	62.4%	65.7%	3.4	639
Spg7	7.5%	10.8%	3.3	640
Hectd3	2.4%	5.7%	3.3	641
Orc1	2.4%	5.7%	3.3	642
Itgav	2.0%	5.3%	3.3	643
Cd247	5.9%	9.2%	3.3	644
Ddx5	93.3%	96.6%	3.3	645
Mrps21l	1.6%	4.9%	3.3	646
LOC103693867	1.6%	4.9%	3.3	647
Zfp61	5.1%	8.4%	3.3	648
Cd53	0.0%	3.3%	3.3	649
Ptprc	0.0%	3.3%	3.3	650
LOC103694910	3.9%	7.2%	3.3	651
RGD1566373	2.0%	5.2%	3.2	652
Prr32	5.9%	9.1%	3.2	653
Oasl2	1.2%	4.4%	3.2	654

Ube2s	5.1%	8.3%	3.2	655
Icoslg	5.1%	8.3%	3.2	656
Ccdc85c	0.4%	3.6%	3.2	657
Fbxo48	0.4%	3.6%	3.2	658
Psma6	96.1%	99.3%	3.2	659
RGD1562758	29.4%	32.6%	3.2	660
Mrps28	0.0%	3.2%	3.2	661
Lsp1	0.0%	3.2%	3.2	662
Cd36	3.5%	6.7%	3.2	663
LOC100912427	3.1%	6.3%	3.2	664
RGD1566226	2.7%	5.9%	3.2	665
LOC103691744	2.0%	5.1%	3.2	666
Ifit3	1.2%	4.3%	3.1	667
LOC100911030	5.1%	8.2%	3.1	668
LOC103695069	5.1%	8.2%	3.1	669
LOC691422	0.8%	3.9%	3.1	670
RGD1564956	9.0%	12.2%	3.1	671
Il1b	0.0%	3.1%	3.1	672
RGD1359290	41.6%	44.7%	3.1	673
Rps28	19.2%	22.3%	3.1	674
Sorbs3	1.6%	4.6%	3.1	675
Best3	1.2%	4.2%	3.1	676
Maf	0.8%	3.8%	3.1	677
RGD1564400	9.0%	12.1%	3.1	678
RGD1562652	0.4%	3.4%	3.0	679
Ldlrad1	0.4%	3.4%	3.0	680
Foxred1	4.3%	7.4%	3.0	681
Nos3	0.0%	3.0%	3.0	682
Slc44a3	2.0%	5.0%	3.0	683
LOC103690888	2.0%	5.0%	3.0	684
LOC100912143	2.0%	5.0%	3.0	685
Kcp	1.2%	4.2%	3.0	686
Nr1i3	96.9%	99.8%	3.0	687
Rpl30l1	5.1%	8.1%	3.0	688
LOC108352812	0.4%	3.4%	3.0	689
LOC689408	0.0%	3.0%	3.0	690
LOC102549305	0.0%	3.0%	3.0	691
LOC108349472	0.0%	3.0%	3.0	692
LOC108353694	0.0%	3.0%	3.0	693
LOC103694382	2.7%	5.7%	2.9	694
Zfp418	6.7%	9.6%	2.9	695
Timm10	93.7%	96.6%	2.9	696
Csrnp1	9.8%	12.7%	2.9	697
Prss32	1.2%	4.1%	2.9	698
LOC102552031	1.2%	4.1%	2.9	699
Marc1	32.5%	35.5%	2.9	700
LOC108349532	5.1%	8.0%	2.9	701
LOC102555173	0.4%	3.3%	2.9	702
LOC108348241	0.0%	2.9%	2.9	703
LOC100361143	0.0%	2.9%	2.9	704
LOC108349032	0.0%	2.9%	2.9	705
LOC100911319	3.9%	6.8%	2.9	706
LOC100909726	3.1%	6.0%	2.9	707
Serpina7	7.1%	9.9%	2.9	708
LOC108348989	7.1%	9.9%	2.9	709
Hexim2	2.7%	5.6%	2.9	710
LOC108353290	2.0%	4.8%	2.8	711
RGD1565131	14.1%	16.9%	2.8	712
Cirbp	1.6%	4.4%	2.8	713
Nif3l1	5.5%	8.3%	2.8	714
RT1-CE3	0.8%	3.6%	2.8	715
LOC102549932	0.8%	3.6%	2.8	716
LOC100361636	0.4%	3.2%	2.8	717
RGD1564447	0.4%	3.2%	2.8	718
Rdh16	8.6%	11.4%	2.8	719
LOC100365590	37.6%	40.4%	2.8	720

Klhdc8b	0.0%	2.8%	2.8	721
Ctsw	0.0%	2.8%	2.8	722
Hmgb2l1	0.0%	2.8%	2.8	723
Phf11b	2.7%	5.5%	2.8	724
Tp53rk	2.7%	5.5%	2.8	725
Mfsd2a	10.6%	13.3%	2.8	726
Cdkl4	2.0%	4.7%	2.8	727
Lyz2	2.0%	4.7%	2.8	728
LOC103692526	1.6%	4.3%	2.7	729
LOC103693375	1.6%	4.3%	2.7	730
Hist1h2ao	1.2%	3.9%	2.7	731
Shc2	0.8%	3.5%	2.7	732
LOC688340	0.8%	3.5%	2.7	733
Fads2	79.6%	82.3%	2.7	734
Egr1	0.4%	3.1%	2.7	735
Aqp8	91.8%	94.5%	2.7	736
Slfn4	0.0%	2.7%	2.7	737
Il2rb	0.0%	2.7%	2.7	738
Arhgef3	0.0%	2.7%	2.7	739
Cd8a	0.0%	2.7%	2.7	740
Mafb	3.9%	6.6%	2.7	741
Romo1	15.3%	18.0%	2.7	742
LOC103691380	2.0%	4.6%	2.7	743
Tmem64	5.1%	7.8%	2.7	744
LOC498829	0.8%	3.4%	2.7	745
Cdc42ep2	0.8%	3.4%	2.7	746
Rftn1	0.8%	3.4%	2.7	747
LOC108352368	0.8%	3.4%	2.7	748
Ncald	0.8%	3.4%	2.7	749
Crip1	0.8%	3.4%	2.7	750
Tmem25	0.8%	3.4%	2.7	751
Aoc2-ps1	0.4%	3.0%	2.6	752
Ndufs6	0.4%	3.0%	2.6	753
LOC100909892	3.9%	6.6%	2.6	754
LOC100361756	3.5%	6.2%	2.6	755
Socs3	5.9%	8.5%	2.6	756
LOC501116	1.2%	3.8%	2.6	757
Mrpl43	1.2%	3.8%	2.6	758
Parp3	13.3%	15.9%	2.6	759
LOC102554669	0.8%	3.4%	2.6	760
LOC108352424	0.4%	3.0%	2.6	761
Samd3	0.0%	2.6%	2.6	762
Plek	0.0%	2.6%	2.6	763
Gzmm	0.0%	2.6%	2.6	764
Ucp2	0.0%	2.6%	2.6	765
LOC498414	0.0%	2.6%	2.6	766
Phf11	3.5%	6.1%	2.5	767
LOC108350839	3.1%	5.7%	2.5	768
LOC679594	2.4%	4.9%	2.5	769
LOC108350476	1.6%	4.1%	2.5	770
LOC100362350	1.2%	3.7%	2.5	771
LOC103690156	12.9%	15.4%	2.5	772
LOC108351097	0.4%	2.9%	2.5	773
LOC108348450	0.4%	2.9%	2.5	774
Il2rg	0.0%	2.5%	2.5	775
LOC691675	0.0%	2.5%	2.5	776
LOC100359539	0.0%	2.5%	2.5	777
Tyrobp	0.0%	2.5%	2.5	778
Fgf21	0.0%	2.5%	2.5	779
Myh9l1	7.8%	10.3%	2.5	780
Slc12a6	2.7%	5.2%	2.5	781
Rgs16	23.5%	26.0%	2.4	782
Pan3	2.4%	4.8%	2.4	783
Sntb1	2.0%	4.4%	2.4	784
LOC108349333	1.6%	4.0%	2.4	785
LOC102548262	0.8%	3.2%	2.4	786

LOC100910446	0.8%	3.2%	2.4	787
LOC685935	0.8%	3.2%	2.4	788
LOC102551071	0.8%	3.2%	2.4	789
Zfp786	0.4%	2.8%	2.4	790
Mta2	0.4%	2.8%	2.4	791
Myl6l	29.4%	31.8%	2.4	792
Aox2	0.0%	2.4%	2.4	793
Ehd3	0.0%	2.4%	2.4	794
LOC108349927	0.0%	2.4%	2.4	795
LOC108353444	0.0%	2.4%	2.4	796
LOC100911564	15.7%	18.1%	2.4	797
Ccdc32	3.1%	5.5%	2.4	798
LOC102553386	3.1%	5.5%	2.4	799
Cyp4a8	15.3%	17.7%	2.4	800
LOC102551889	2.7%	5.1%	2.4	801
Hist2h2aa3	2.7%	5.1%	2.4	802
Naglt1	6.7%	9.0%	2.4	803
Poc1b	2.0%	4.3%	2.4	804
LOC108352110	5.9%	8.2%	2.4	805
Lnc134	1.6%	3.9%	2.3	806
Lsm6	1.6%	3.9%	2.3	807
Cbln3	1.2%	3.5%	2.3	808
LOC102552476	1.2%	3.5%	2.3	809
Mapre3	1.2%	3.5%	2.3	810
LOC100910180	1.2%	3.5%	2.3	811
LOC102548639	0.8%	3.1%	2.3	812
Tmem30b	0.8%	3.1%	2.3	813
LOC100362333	0.8%	3.1%	2.3	814
LOC682870	4.7%	7.0%	2.3	815
Fosb	0.4%	2.7%	2.3	816
Neu4	0.0%	2.3%	2.3	817
LOC257642	0.0%	2.3%	2.3	818
Cyba	0.0%	2.3%	2.3	819
Mis18a	0.0%	2.3%	2.3	820
Atp5g2	94.9%	97.2%	2.3	821
Pcid2	3.1%	5.4%	2.3	822
Slamf7	7.1%	9.4%	2.3	823
LOC108353644	2.0%	4.2%	2.3	824
Cd46	1.6%	3.8%	2.3	825
Scn2b	1.2%	3.4%	2.3	826
Cep83os	0.8%	3.0%	2.3	827
RGD1565170	9.0%	11.3%	2.3	828
LOC103693373	0.4%	2.6%	2.2	829
Hrc	0.4%	2.6%	2.2	830
LOC689065	0.0%	2.2%	2.2	831
Prickle4	0.0%	2.2%	2.2	832
Slfn2	0.0%	2.2%	2.2	833
Cd274	0.0%	2.2%	2.2	834
Stard6	3.9%	6.2%	2.2	835
LOC100909481	3.9%	6.2%	2.2	836
Prrc2c	7.8%	10.1%	2.2	837
RGD1560099	3.1%	5.4%	2.2	838
Fitm2	6.7%	8.9%	2.2	839
Abcg3l2	10.6%	12.8%	2.2	840
Oas1b	2.0%	4.2%	2.2	841
Aspscr1	18.4%	20.6%	2.2	842
LOC102555473	1.6%	3.8%	2.2	843
Gsto2	1.6%	3.8%	2.2	844
Ppef2	0.8%	3.0%	2.2	845
Zgrf1	0.8%	3.0%	2.2	846
Tcf7l1	0.4%	2.6%	2.2	847
LOC103692920	0.4%	2.6%	2.2	848
Nkg7	0.0%	2.2%	2.2	849
Cd2	0.0%	2.2%	2.2	850
Hnrnpul2	0.0%	2.2%	2.2	851
LOC100125366	3.1%	5.3%	2.1	852

LOC314140	5.5%	7.6%	2.1	853
LOC102551907	0.8%	2.9%	2.1	854
Anapc1	4.7%	6.8%	2.1	855
Zbtb41	4.7%	6.8%	2.1	856
Kcnj6	0.4%	2.5%	2.1	857
Tsx	0.4%	2.5%	2.1	858
LOC102550980	0.4%	2.5%	2.1	859
Mbd4	0.4%	2.5%	2.1	860
Pcyt1b	0.0%	2.1%	2.1	861
Lck	0.0%	2.1%	2.1	862
LOC102556053	0.0%	2.1%	2.1	863
LOC688754	0.0%	2.1%	2.1	864
LOC108352552	0.0%	2.1%	2.1	865
LOC108353803	3.5%	5.6%	2.1	866
Pomk	7.5%	9.5%	2.1	867
Ndufa1	28.2%	30.3%	2.1	868
LOC100362110	2.7%	4.8%	2.1	869
Slc31a1	11.0%	13.0%	2.0	870
Trim31	11.0%	13.0%	2.0	871
Nom1	6.3%	8.3%	2.0	872
LOC100364561	1.6%	3.6%	2.0	873
Stra13	1.6%	3.6%	2.0	874
Abcg3l1	1.2%	3.2%	2.0	875
Top3a	1.2%	3.2%	2.0	876
Il4i1	0.8%	2.8%	2.0	877
Layn	0.8%	2.8%	2.0	878
LOC361914	0.8%	2.8%	2.0	879
Acsf2	0.4%	2.4%	2.0	880
Smyd1	0.4%	2.4%	2.0	881
Blk	0.0%	2.0%	2.0	882
Adam8	0.0%	2.0%	2.0	883
Gzmb	0.0%	2.0%	2.0	884
Ctss	0.0%	2.0%	2.0	885
LOC500846	0.0%	2.0%	2.0	886
Ccl3	0.0%	2.0%	2.0	887
Depdc5	2.4%	4.3%	2.0	888
Rn45s	98.0%	100.0%	2.0	889
LOC102550654	2.0%	3.9%	2.0	890
Hist2h2ac	1.6%	3.5%	1.9	891
LOC682402	5.5%	7.4%	1.9	892
LOC100361492	1.2%	3.1%	1.9	893
Ctps2	5.1%	7.0%	1.9	894
LOC100912218	0.4%	2.3%	1.9	895
LOC108349998	0.4%	2.3%	1.9	896
Hsbp1l1	0.4%	2.3%	1.9	897
LOC103693330	4.3%	6.2%	1.9	898
Pkm	0.0%	1.9%	1.9	899
Rrm2	0.0%	1.9%	1.9	900
LOC100909605	0.0%	1.9%	1.9	901
LOC102555246	0.0%	1.9%	1.9	902
Cd2bp2	52.9%	54.8%	1.9	903
LOC100911674	2.7%	4.6%	1.9	904
LOC108348163	2.4%	4.2%	1.9	905
LOC103691072	2.0%	3.8%	1.9	906
LOC102547853	1.2%	3.0%	1.9	907
LOC103694404	1.2%	3.0%	1.9	908
Tnpo2	0.8%	2.6%	1.9	909
RGD1561102	0.8%	2.6%	1.9	910
Mroh4	0.4%	2.2%	1.8	911
LOC102547883	0.4%	2.2%	1.8	912
Casr	0.4%	2.2%	1.8	913
LOC100911485	0.4%	2.2%	1.8	914
LOC100360260	0.0%	1.8%	1.8	915
LOC103691410	0.0%	1.8%	1.8	916
LOC103692633	0.0%	1.8%	1.8	917
RT1-CE16	0.0%	1.8%	1.8	918

Rgs1	0.0%	1.8%	1.8	919
Cxcr4	0.0%	1.8%	1.8	920
Arhgdib	0.0%	1.8%	1.8	921
LOC108351112	0.0%	1.8%	1.8	922
Aif1	0.0%	1.8%	1.8	923
LOC100912478	7.5%	9.3%	1.8	924
Frmd4b	3.1%	5.0%	1.8	925
Tmem251	7.1%	8.9%	1.8	926
Hnrnph2	2.7%	4.6%	1.8	927
Pura	6.7%	8.5%	1.8	928
RT1-A2	2.0%	3.8%	1.8	929
Ly6e	2.0%	3.8%	1.8	930
LOC102556129	1.6%	3.4%	1.8	931
Hspa4l	5.5%	7.3%	1.8	932
Tmem106a	1.2%	3.0%	1.8	933
RGD1565767	1.2%	3.0%	1.8	934
Alg6	5.1%	6.9%	1.8	935
LOC103689920	9.0%	10.8%	1.8	936
LOC102554918	0.4%	2.2%	1.8	937
Sync	0.4%	2.2%	1.8	938
RGD1563091	0.4%	2.2%	1.8	939
LOC103694855	0.0%	1.8%	1.8	940
RGD1563713	0.0%	1.8%	1.8	941
Gzmbl2	0.0%	1.8%	1.8	942
Spn	0.0%	1.8%	1.8	943
LOC102549812	0.0%	1.8%	1.8	944
Ptprcap	0.0%	1.8%	1.8	945
Amn1	58.0%	59.8%	1.8	946
LOC100910308	11.0%	12.7%	1.7	947
Sfxn1	2.4%	4.1%	1.7	948
Pnpla2	2.4%	4.1%	1.7	949
LOC102548076	1.6%	3.3%	1.7	950
Bcl2a1	1.6%	3.3%	1.7	951
LOC108353612	1.2%	2.9%	1.7	952
Dpp9	1.2%	2.9%	1.7	953
Tnfsf10	1.2%	2.9%	1.7	954
Psmb11	1.2%	2.9%	1.7	955
Gnb5	0.8%	2.5%	1.7	956
Rmi2	0.8%	2.5%	1.7	957
Btg2	42.4%	44.0%	1.7	958
Gadd45b	25.5%	27.2%	1.7	959
Fcer1g	0.4%	2.1%	1.7	960
RGD1564319	0.4%	2.1%	1.7	961
Txnip	0.4%	2.1%	1.7	962
S100a13	0.4%	2.1%	1.7	963
Tigd3	0.4%	2.1%	1.7	964
Rimkla	0.0%	1.7%	1.7	965
Cux2	0.0%	1.7%	1.7	966
RGD1562196	0.0%	1.7%	1.7	967
RGD1562667	0.0%	1.7%	1.7	968
LOC364556	0.0%	1.7%	1.7	969
LOC103694857	0.0%	1.7%	1.7	970
RGD1561662	7.1%	8.7%	1.7	971
Mpv17l	27.8%	29.5%	1.7	972
Vdac1	15.3%	16.9%	1.7	973
LOC684509	2.7%	4.4%	1.7	974
Efhd2	2.4%	4.0%	1.6	975
Man2b2	1.6%	3.2%	1.6	976
LOC108352747	1.2%	2.8%	1.6	977
LOC100360218	0.8%	2.4%	1.6	978
Cybrd1	0.8%	2.4%	1.6	979
Pbx2	0.8%	2.4%	1.6	980
Nlk	0.8%	2.4%	1.6	981
Samd9l	0.4%	2.0%	1.6	982
LOC108352751	0.4%	2.0%	1.6	983
Ahsa2	0.4%	2.0%	1.6	984

Arhgef2	0.4%	2.0%	1.6	985
LOC499136	0.4%	2.0%	1.6	986
Osmr	0.4%	2.0%	1.6	987
LOC102556446	0.0%	1.6%	1.6	988
LOC100910278	0.0%	1.6%	1.6	989
P3h4	0.0%	1.6%	1.6	990
Kdr	0.0%	1.6%	1.6	991
Satb1	0.0%	1.6%	1.6	992
Grap	0.0%	1.6%	1.6	993
RGD1565462	0.0%	1.6%	1.6	994
Ccrl2	0.0%	1.6%	1.6	995
Gpr171	0.0%	1.6%	1.6	996
Tmem258	11.0%	12.5%	1.6	997
Cep68	2.4%	3.9%	1.6	998
Shtn1	98.0%	99.6%	1.6	999
LOC108348229	2.0%	3.5%	1.6	1000
LOC306079	5.9%	7.4%	1.6	1001
Yes1	5.9%	7.4%	1.6	1002
LOC102548189	1.6%	3.1%	1.5	1003
Tpd52l1	1.6%	3.1%	1.5	1004
Traf1	1.2%	2.7%	1.5	1005
LOC108353247	0.8%	2.3%	1.5	1006
LOC24906	0.8%	2.3%	1.5	1007
Acvrl1	0.8%	2.3%	1.5	1008
LOC102550182	0.8%	2.3%	1.5	1009
LOC102550026	0.4%	1.9%	1.5	1010
Chrne	0.4%	1.9%	1.5	1011
Fcgr2b	0.4%	1.9%	1.5	1012
Ripk3	0.4%	1.9%	1.5	1013
LOC108351237	0.4%	1.9%	1.5	1014
Trib1	0.4%	1.9%	1.5	1015
LOC108348765	0.0%	1.5%	1.5	1016
Cd3e	0.0%	1.5%	1.5	1017
Fxyd5	0.0%	1.5%	1.5	1018
LOC102555378	0.0%	1.5%	1.5	1019
LOC680191	0.0%	1.5%	1.5	1020
Cd3d	0.0%	1.5%	1.5	1021
Ifitm2	0.0%	1.5%	1.5	1022
LOC103694190	0.0%	1.5%	1.5	1023
LOC103690821	0.0%	1.5%	1.5	1024
Grm2	0.0%	1.5%	1.5	1025
Slc38a6	0.0%	1.5%	1.5	1026
Comp	0.0%	1.5%	1.5	1027
C1qa	0.0%	1.5%	1.5	1028
Prkch	0.0%	1.5%	1.5	1029
LOC500350	0.0%	1.5%	1.5	1030
Rnf125	2.7%	4.2%	1.5	1031
RT1-T24-4	2.7%	4.2%	1.5	1032
Cdkn1b	2.7%	4.2%	1.5	1033
Pspc1	2.0%	3.4%	1.5	1034
LOC102547604	2.0%	3.4%	1.5	1035
Pts	14.1%	15.6%	1.5	1036
Tceal9	1.6%	3.0%	1.5	1037
Ago3	1.6%	3.0%	1.5	1038
LOC103690031	1.2%	2.6%	1.5	1039
LOC102548346	1.2%	2.6%	1.5	1040
Cwf19l1	1.2%	2.6%	1.5	1041
Zc3h12a	5.1%	6.6%	1.5	1042
LOC103689934	0.8%	2.2%	1.5	1043
RT1-M1-2	0.8%	2.2%	1.5	1044
Gbp2	0.8%	2.2%	1.5	1045
Ska2	0.8%	2.2%	1.5	1046
Grk4	0.8%	2.2%	1.5	1047
LOC100911562	0.8%	2.2%	1.5	1048
Spidr	0.8%	2.2%	1.5	1049
Dhx16	0.8%	2.2%	1.5	1050

Milr1	9.0%	10.5%	1.5	1051
LOC102557219	0.4%	1.8%	1.4	1052
Htr2b	0.4%	1.8%	1.4	1053
Arrdc4	0.4%	1.8%	1.4	1054
LOC102547823	0.0%	1.4%	1.4	1055
Stk11	0.0%	1.4%	1.4	1056
LOC102546394	0.0%	1.4%	1.4	1057
Gpr183	0.0%	1.4%	1.4	1058
LOC100362738	0.0%	1.4%	1.4	1059
LOC681458	0.0%	1.4%	1.4	1060
LOC691695	0.0%	1.4%	1.4	1061
Ckb	0.0%	1.4%	1.4	1062
Myo1g	0.0%	1.4%	1.4	1063
Tsn	0.0%	1.4%	1.4	1064
Rbm20	0.0%	1.4%	1.4	1065
RGD1561635	0.0%	1.4%	1.4	1066
Marcks	0.0%	1.4%	1.4	1067
LOC102551486	0.0%	1.4%	1.4	1068
Zfp410	2.7%	4.2%	1.4	1069
Plat	1.6%	3.0%	1.4	1070
LOC108348142	43.1%	44.5%	1.4	1071
Ddx3	9.8%	11.2%	1.4	1072
Tmem234	1.2%	2.6%	1.4	1073
Tmem258b	1.2%	2.6%	1.4	1074
Rsbn1	1.2%	2.6%	1.4	1075
Mpa2l	0.8%	2.2%	1.4	1076
Zfp668	0.8%	2.2%	1.4	1077
LOC685069	0.8%	2.2%	1.4	1078
Exoc6	0.8%	2.2%	1.4	1079
Smim4	0.8%	2.2%	1.4	1080
RGD1309808	0.8%	2.2%	1.4	1081
Gimap9	4.7%	6.1%	1.4	1082
LOC108352829	0.4%	1.8%	1.4	1083
Igfbp7	0.4%	1.8%	1.4	1084
Tfpi2	0.4%	1.8%	1.4	1085
Deaf1	0.4%	1.8%	1.4	1086
Lcp1	0.4%	1.8%	1.4	1087
Armc9	0.4%	1.8%	1.4	1088
LOC102548883	0.4%	1.8%	1.4	1089
Veph1	0.4%	1.8%	1.4	1090
LOC499219	0.4%	1.8%	1.4	1091
Zkscan5	0.4%	1.8%	1.4	1092
Eif4e2	0.4%	1.8%	1.4	1093
Tmem184a	0.4%	1.8%	1.4	1094
LOC102551980	0.4%	1.8%	1.4	1095
LOC100364500	0.0%	1.4%	1.4	1096
Dennd1c	0.0%	1.4%	1.4	1097
LOC100910581	0.0%	1.4%	1.4	1098
Limd2	0.0%	1.4%	1.4	1099
Cmtm7	0.0%	1.4%	1.4	1100
LOC103693522	0.0%	1.4%	1.4	1101
RGD1565356	0.0%	1.4%	1.4	1102
Slc5a5	0.0%	1.4%	1.4	1103
Cldn20	0.0%	1.4%	1.4	1104
Evl	0.0%	1.4%	1.4	1105
Gzma	0.0%	1.4%	1.4	1106
Icos	0.0%	1.4%	1.4	1107
Mslnl	0.0%	1.4%	1.4	1108
LOC103694953	0.0%	1.4%	1.4	1109
LOC108352541	0.0%	1.4%	1.4	1110
Gzmc	0.0%	1.4%	1.4	1111
Ecm1	0.0%	1.4%	1.4	1112
Irx2	0.0%	1.4%	1.4	1113
Cd3g	0.0%	1.4%	1.4	1114
Prss8	8.2%	9.6%	1.4	1115
Upf1	3.9%	5.3%	1.4	1116

LOC313310	2.7%	4.1%	1.3	1117
LOC688672	2.4%	3.7%	1.3	1118
LOC100910121	2.0%	3.3%	1.3	1119
Pfas	1.6%	2.9%	1.3	1120
Ifitm7	1.6%	2.9%	1.3	1121
Wac	1.6%	2.9%	1.3	1122
LOC100361645	1.2%	2.5%	1.3	1123
Zmynd8	1.2%	2.5%	1.3	1124
Pced1a	0.8%	2.1%	1.3	1125
Ccl2	0.8%	2.1%	1.3	1126
Hexdc	0.8%	2.1%	1.3	1127
Giot1	0.8%	2.1%	1.3	1128
LOC102550127	0.8%	2.1%	1.3	1129
Prl8a5	0.8%	2.1%	1.3	1130
LOC108348882	0.4%	1.7%	1.3	1131
Sfxn2	0.4%	1.7%	1.3	1132
Speg	0.4%	1.7%	1.3	1133
Nbeal1	0.4%	1.7%	1.3	1134
Tlr1	0.4%	1.7%	1.3	1135
March3	0.0%	1.3%	1.3	1136
LOC690183	0.0%	1.3%	1.3	1137
Tfap4	0.0%	1.3%	1.3	1138
Mx1	0.0%	1.3%	1.3	1139
Fmnl1	0.0%	1.3%	1.3	1140
Upp1	0.0%	1.3%	1.3	1141
Sparc	0.0%	1.3%	1.3	1142
Art2b	0.0%	1.3%	1.3	1143
Tnfrsf18	0.0%	1.3%	1.3	1144
LOC103690914	0.0%	1.3%	1.3	1145
Icam4	0.0%	1.3%	1.3	1146
LOC102553117	0.0%	1.3%	1.3	1147
Ankh	0.0%	1.3%	1.3	1148
LOC100363335	0.0%	1.3%	1.3	1149
S100a4	0.0%	1.3%	1.3	1150
LOC108353165	0.0%	1.3%	1.3	1151
Wnt16	0.0%	1.3%	1.3	1152
Sp2	0.0%	1.3%	1.3	1153
LOC103694212	0.0%	1.3%	1.3	1154
Apbb1	0.0%	1.3%	1.3	1155
LOC102552725	0.0%	1.3%	1.3	1156
Lrg1	0.0%	1.3%	1.3	1157
Aqp1	0.0%	1.3%	1.3	1158
Cd69	0.0%	1.3%	1.3	1159
S100a6	0.0%	1.3%	1.3	1160
B3galt1	7.8%	9.1%	1.3	1161
Snrpd3	3.5%	4.8%	1.3	1162
Pla1a	3.1%	4.4%	1.3	1163
Ppidl1	3.1%	4.4%	1.3	1164
Zdhhc2	3.1%	4.4%	1.3	1165
Trnau1ap	3.1%	4.4%	1.3	1166
LOC100360841	15.3%	16.5%	1.3	1167
Ppard	2.4%	3.6%	1.2	1168
Ralb	2.0%	3.2%	1.2	1169
Scaf1	2.0%	3.2%	1.2	1170
LOC102550220	2.0%	3.2%	1.2	1171
Acin1	2.0%	3.2%	1.2	1172
LOC108348149	1.6%	2.8%	1.2	1173
Nr2f2	1.6%	2.8%	1.2	1174
LOC102551714	1.2%	2.4%	1.2	1175
Ccnd3	0.8%	2.0%	1.2	1176
Oraov1	0.8%	2.0%	1.2	1177
Abhd11os	0.8%	2.0%	1.2	1178
Bok	0.8%	2.0%	1.2	1179
Mau2	0.8%	2.0%	1.2	1180
Taf7	0.8%	2.0%	1.2	1181
LOC100909505	4.7%	5.9%	1.2	1182

Mthfd2	0.4%	1.6%	1.2	1183
RT1-CE7	0.4%	1.6%	1.2	1184
Rassf5	0.4%	1.6%	1.2	1185
LOC102555914	0.4%	1.6%	1.2	1186
LOC103692215	0.4%	1.6%	1.2	1187
LOC103690108	0.4%	1.6%	1.2	1188
LOC100910579	8.6%	9.8%	1.2	1189
LOC108352650	16.9%	18.1%	1.2	1190
Rnf19b	4.3%	5.5%	1.2	1191
Mllt6	0.0%	1.2%	1.2	1192
Mzb1	0.0%	1.2%	1.2	1193
LOC108348973	0.0%	1.2%	1.2	1194
Epn1	0.0%	1.2%	1.2	1195
Faslg	0.0%	1.2%	1.2	1196
Chsy1	0.0%	1.2%	1.2	1197
Cd48	0.0%	1.2%	1.2	1198
P2ry10	0.0%	1.2%	1.2	1199
Cnn2	0.0%	1.2%	1.2	1200
Caprin2	0.0%	1.2%	1.2	1201
Nfkbid	0.0%	1.2%	1.2	1202
Ptprb	0.0%	1.2%	1.2	1203
LOC108351010	0.0%	1.2%	1.2	1204
Doc2b	0.0%	1.2%	1.2	1205
Fyb	0.0%	1.2%	1.2	1206
LOC108350364	0.0%	1.2%	1.2	1207
Chmp4c	3.5%	4.7%	1.2	1208
Il1rn	7.5%	8.6%	1.2	1209
RGD1563378	3.1%	4.3%	1.2	1210
LOC690131	6.7%	7.8%	1.2	1211
Usp5	2.0%	3.1%	1.2	1212
Fam192a	1.6%	2.7%	1.1	1213
Cbx3	1.2%	2.3%	1.1	1214
B4gat1	1.2%	2.3%	1.1	1215
LOC100362552	1.2%	2.3%	1.1	1216
Mapk1ip1	1.2%	2.3%	1.1	1217
Map3k7	1.2%	2.3%	1.1	1218
Slamf8	1.2%	2.3%	1.1	1219
LOC102547797	1.2%	2.3%	1.1	1220
Il10rb	1.2%	2.3%	1.1	1221
Ifih1	0.8%	1.9%	1.1	1222
Lrrc25	0.8%	1.9%	1.1	1223
Fam92a1	0.8%	1.9%	1.1	1224
Rabgap1	0.8%	1.9%	1.1	1225
Abcg3	0.8%	1.9%	1.1	1226
LOC100359517	0.8%	1.9%	1.1	1227
LOC103693261	4.7%	5.8%	1.1	1228
Irf2bp2	4.7%	5.8%	1.1	1229
Cdk4	4.7%	5.8%	1.1	1230
Dhfr	4.7%	5.8%	1.1	1231
Dhcr24	0.4%	1.5%	1.1	1232
Blzf1	0.4%	1.5%	1.1	1233
Ralgapa1	0.4%	1.5%	1.1	1234
Aacs	0.4%	1.5%	1.1	1235
LOC102548552	0.4%	1.5%	1.1	1236
Prpf40b	0.4%	1.5%	1.1	1237
Nfia	0.0%	1.1%	1.1	1238
LOC103694191	0.0%	1.1%	1.1	1239
Cpa2	0.0%	1.1%	1.1	1240
Peli3	0.0%	1.1%	1.1	1241
Dennd3	0.0%	1.1%	1.1	1242
Pik3cd	0.0%	1.1%	1.1	1243
Fam234b	0.0%	1.1%	1.1	1244
Fam167b	0.0%	1.1%	1.1	1245
Slc38a1	0.0%	1.1%	1.1	1246
Cenps	0.0%	1.1%	1.1	1247
Lgals1	0.0%	1.1%	1.1	1248

Atp6v1b1	0.0%	1.1%	1.1	1249
Batf3	0.0%	1.1%	1.1	1250
LOC102547230	0.0%	1.1%	1.1	1251
Ms4a4c	0.0%	1.1%	1.1	1252
Gigyf1	0.0%	1.1%	1.1	1253
Fbxo32	0.0%	1.1%	1.1	1254
Pycard	0.0%	1.1%	1.1	1255
LOC102552802	0.0%	1.1%	1.1	1256
Slpi	0.0%	1.1%	1.1	1257
LOC680635	0.0%	1.1%	1.1	1258
Ttc4	8.2%	9.4%	1.1	1259
Hnrnpa2b1	7.8%	9.0%	1.1	1260
RGD1560961	20.0%	21.1%	1.1	1261
Zfhx3	3.1%	4.2%	1.1	1262
Ybx1-ps3	23.5%	24.6%	1.1	1263
LOC103690393	2.0%	3.0%	1.1	1264
LOC500712	1.6%	2.6%	1.1	1265
LOC103689965	1.6%	2.6%	1.1	1266
LOC108348295	1.6%	2.6%	1.1	1267
LOC100910821	1.6%	2.6%	1.1	1268
Mfsd14a	1.6%	2.6%	1.1	1269
LOC100912571	5.5%	6.6%	1.1	1270
Map3k13	1.2%	2.2%	1.1	1271
Ppp4c	1.2%	2.2%	1.1	1272
LOC102549824	1.2%	2.2%	1.1	1273
Plpp6	1.2%	2.2%	1.1	1274
Traf3ip2	1.2%	2.2%	1.1	1275
Lgals9	84.3%	85.4%	1.1	1276
LOC108353440	0.8%	1.8%	1.1	1277
Mkl1	0.8%	1.8%	1.1	1278
Slc4a1	0.8%	1.8%	1.1	1279
Nfatc2	0.8%	1.8%	1.1	1280
LOC100909916	0.8%	1.8%	1.1	1281
Hmbox1	0.4%	1.4%	1.0	1282
Tbx10	0.4%	1.4%	1.0	1283
LOC100361944	0.4%	1.4%	1.0	1284
Exoc4	0.4%	1.4%	1.0	1285
LOC103690379	0.4%	1.4%	1.0	1286
Hdc	0.4%	1.4%	1.0	1287
Taf13	8.6%	9.7%	1.0	1288
LOC108353085	0.0%	1.0%	1.0	1289
Epas1	0.0%	1.0%	1.0	1290
Tbc1d10c	0.0%	1.0%	1.0	1291
Adgrf5	0.0%	1.0%	1.0	1292
LOC108352924	0.0%	1.0%	1.0	1293
Zeb2	0.0%	1.0%	1.0	1294
LOC102548661	0.0%	1.0%	1.0	1295
LOC108348580	0.0%	1.0%	1.0	1296
LOC100363993	0.0%	1.0%	1.0	1297
F7	0.0%	1.0%	1.0	1298
Bgn	0.0%	1.0%	1.0	1299
Rps4x-ps1	0.0%	1.0%	1.0	1300
Xdh	0.0%	1.0%	1.0	1301
Scd4	0.0%	1.0%	1.0	1302
Serpinb6b	0.0%	1.0%	1.0	1303
LOC100912577	0.0%	1.0%	1.0	1304
Irg1	0.0%	1.0%	1.0	1305
Sept11	0.0%	1.0%	1.0	1306
Myo1e	0.0%	1.0%	1.0	1307
LOC108350586	0.0%	1.0%	1.0	1308
LOC102548812	0.0%	1.0%	1.0	1309
Enah	0.0%	1.0%	1.0	1310
Cdk1	0.0%	1.0%	1.0	1311
Stat4	0.0%	1.0%	1.0	1312
Il21r	0.0%	1.0%	1.0	1313
Arl13b	0.0%	1.0%	1.0	1314

Cyfip2	0.0%	1.0%	1.0	1315
LOC108352168	0.0%	1.0%	1.0	1316
Il1a	0.0%	1.0%	1.0	1317
LOC108348299	0.0%	1.0%	1.0	1318
Tgfb1	0.0%	1.0%	1.0	1319
RGD1560263	0.0%	1.0%	1.0	1320
Erich5	3.5%	4.6%	1.0	1321
Edn1	2.7%	3.8%	1.0	1322
Mrpl30	19.2%	20.2%	1.0	1323
Snrpd2l	2.4%	3.4%	1.0	1324
Stk38	2.4%	3.4%	1.0	1325
LOC100911356	2.4%	3.4%	1.0	1326
LOC500354	5.9%	6.9%	1.0	1327
Ptpra	1.6%	2.6%	1.0	1328
Zfp35	1.6%	2.6%	1.0	1329
LOC102556098	1.6%	2.6%	1.0	1330
LOC102550676	1.6%	2.6%	1.0	1331
LOC108351865	1.2%	2.2%	1.0	1332
Ccdc167	1.2%	2.2%	1.0	1333
Mmab	1.2%	2.2%	1.0	1334
Hspa14	1.2%	2.2%	1.0	1335
Klhdc7a	5.1%	6.1%	1.0	1336
Gigyf2	0.8%	1.8%	1.0	1337
Pgbd1	0.8%	1.8%	1.0	1338
Slc25a11	0.8%	1.8%	1.0	1339
Atn1	0.8%	1.8%	1.0	1340
Ttc39a	0.8%	1.8%	1.0	1341
LOC102557158	0.8%	1.8%	1.0	1342
RT1-DOa	0.8%	1.8%	1.0	1343
RGD1563352	0.8%	1.8%	1.0	1344
Plekha6	0.8%	1.8%	1.0	1345
LOC100363406	0.8%	1.8%	1.0	1346
LOC108353198	0.8%	1.8%	1.0	1347
Churc1	58.8%	59.8%	1.0	1348
Hmg20a	0.4%	1.4%	1.0	1349
LOC103692874	0.4%	1.4%	1.0	1350
LOC102550396	0.4%	1.4%	1.0	1351
Il18rap	0.4%	1.4%	1.0	1352
Tatdn3	0.4%	1.4%	1.0	1353
LOC108352003	0.4%	1.4%	1.0	1354
LOC499742	0.4%	1.4%	1.0	1355
Trip10	0.4%	1.4%	1.0	1356
RGD1560854	0.4%	1.4%	1.0	1357
Rhpn1	0.4%	1.4%	1.0	1358
LOC102546452	0.0%	1.0%	1.0	1359
LOC102547588	0.0%	1.0%	1.0	1360
LOC108353458	0.0%	1.0%	1.0	1361
LOC103692403	0.0%	1.0%	1.0	1362
Prelid3a	0.0%	1.0%	1.0	1363
Megf8	0.0%	1.0%	1.0	1364
Prr13	0.0%	1.0%	1.0	1365
Tspan13	0.0%	1.0%	1.0	1366
RGD1559951	0.0%	1.0%	1.0	1367
Pdhx	0.0%	1.0%	1.0	1368
LOC102551372	0.0%	1.0%	1.0	1369
Dpysl2	0.0%	1.0%	1.0	1370
Ldhb	0.0%	1.0%	1.0	1371
LOC102556157	0.0%	1.0%	1.0	1372
Itk	0.0%	1.0%	1.0	1373
Ormdl1	0.0%	1.0%	1.0	1374
Hsd17b1	0.0%	1.0%	1.0	1375
Fam26f	0.0%	1.0%	1.0	1376
LOC103692432	0.0%	1.0%	1.0	1377
Gngt2	0.0%	1.0%	1.0	1378
LOC102548531	0.0%	1.0%	1.0	1379
Zc3h6	0.0%	1.0%	1.0	1380

LOC103692182	0.0%	1.0%	1.0	1381
Slc2a5	0.0%	1.0%	1.0	1382
Ky	0.0%	1.0%	1.0	1383
Slfn3	0.0%	1.0%	1.0	1384
Klrk1	0.0%	1.0%	1.0	1385
LOC108349646	0.0%	1.0%	1.0	1386
Gng2	0.0%	1.0%	1.0	1387
Pde7a	0.0%	1.0%	1.0	1388
LOC102547181	0.0%	1.0%	1.0	1389
Vamp8	0.0%	1.0%	1.0	1390
RGD1564617	0.0%	1.0%	1.0	1391
LOC100911813	0.0%	1.0%	1.0	1392
LOC500013	0.0%	1.0%	1.0	1393
Sfn	0.0%	1.0%	1.0	1394
Zfp184	0.0%	1.0%	1.0	1395
Ccdc80	0.0%	1.0%	1.0	1396
Gatb	0.0%	1.0%	1.0	1397
C1qc	0.0%	1.0%	1.0	1398
Cd44	0.0%	1.0%	1.0	1399
Msln	0.0%	1.0%	1.0	1400
Il7r	0.0%	1.0%	1.0	1401
Lime1	0.0%	1.0%	1.0	1402
LOC108348813	0.0%	1.0%	1.0	1403
Cst7	0.0%	1.0%	1.0	1404
Ermp1	3.9%	4.9%	1.0	1405
LOC100365062	7.5%	8.4%	0.9	1406
LOC108348048	3.1%	4.1%	0.9	1407
Uxs1	7.1%	8.0%	0.9	1408
Alkbh1	7.1%	8.0%	0.9	1409
Bpgm	1.6%	2.5%	0.9	1410
Mpdz	1.6%	2.5%	0.9	1411
Rbbp9	5.5%	6.4%	0.9	1412
Ier3ip1	5.5%	6.4%	0.9	1413
Rfesd	1.2%	2.1%	0.9	1414
Rbmx	1.2%	2.1%	0.9	1415
LOC108349768	1.2%	2.1%	0.9	1416
LOC108350694	5.1%	6.0%	0.9	1417
LOC102557117	0.8%	1.7%	0.9	1418
Ric8b	0.8%	1.7%	0.9	1419
LOC102548358	0.8%	1.7%	0.9	1420
Rbm12	0.8%	1.7%	0.9	1421
LOC100911672	0.8%	1.7%	0.9	1422
LOC688452	0.8%	1.7%	0.9	1423
Chit1	0.8%	1.7%	0.9	1424
Cby1	0.8%	1.7%	0.9	1425
LOC103690394	0.8%	1.7%	0.9	1426
Rab11b	9.0%	9.9%	0.9	1427
Ncr1	0.4%	1.3%	0.9	1428
Trappc1	0.4%	1.3%	0.9	1429
Dnal4	0.4%	1.3%	0.9	1430
RT1-Cl	0.4%	1.3%	0.9	1431
LOC102550895	0.4%	1.3%	0.9	1432
Spry2	0.4%	1.3%	0.9	1433
Calcoco1	0.4%	1.3%	0.9	1434
Slc24a2	0.4%	1.3%	0.9	1435
Csf1	0.4%	1.3%	0.9	1436
LOC108352861	21.2%	22.1%	0.9	1437
Lifr	0.0%	0.9%	0.9	1438
Vps13c	0.0%	0.9%	0.9	1439
Tnfrsf9	0.0%	0.9%	0.9	1440
Gimap4	0.0%	0.9%	0.9	1441
Vsir	0.0%	0.9%	0.9	1442
Gimap7	0.0%	0.9%	0.9	1443
Fcgr3a	0.0%	0.9%	0.9	1444
Batf	0.0%	0.9%	0.9	1445
Hid1	0.0%	0.9%	0.9	1446

Rasl10b	0.0%	0.9%	0.9	1447
Il1r2	0.0%	0.9%	0.9	1448
LOC103691294	0.0%	0.9%	0.9	1449
Sdpr	0.0%	0.9%	0.9	1450
Vim	0.0%	0.9%	0.9	1451
Hmgb1-ps2	0.0%	0.9%	0.9	1452
Cntrl	0.0%	0.9%	0.9	1453
RT1-CE4	0.0%	0.9%	0.9	1454
F2r	0.0%	0.9%	0.9	1455
Sema4d	0.0%	0.9%	0.9	1456
Grm4	0.0%	0.9%	0.9	1457
RGD1560554	0.0%	0.9%	0.9	1458
Trim30	0.0%	0.9%	0.9	1459
LOC108349479	0.0%	0.9%	0.9	1460
Myo18a	0.0%	0.9%	0.9	1461
Dpp7	0.0%	0.9%	0.9	1462
Me2	0.0%	0.9%	0.9	1463
S1pr4	0.0%	0.9%	0.9	1464
RT1-CE11	0.0%	0.9%	0.9	1465
Arl5c	0.0%	0.9%	0.9	1466
Pip4k2a	0.0%	0.9%	0.9	1467
Cwc25	0.0%	0.9%	0.9	1468
Rps6ka3	0.0%	0.9%	0.9	1469
Myh10	0.0%	0.9%	0.9	1470
LOC103692213	0.0%	0.9%	0.9	1471
Sh3rf1	0.0%	0.9%	0.9	1472
Fyn	0.0%	0.9%	0.9	1473
Cytip	0.0%	0.9%	0.9	1474
Ropn1	0.0%	0.9%	0.9	1475
LOC689064	0.0%	0.9%	0.9	1476
LOC100910589	0.0%	0.9%	0.9	1477
Mob3a	0.0%	0.9%	0.9	1478
Gins3	0.0%	0.9%	0.9	1479
LOC498750	0.0%	0.9%	0.9	1480
Ctns	0.0%	0.9%	0.9	1481
Gzmk	0.0%	0.9%	0.9	1482
LOC290595	0.0%	0.9%	0.9	1483
Ubash3b	0.0%	0.9%	0.9	1484
Pogk	16.5%	17.3%	0.9	1485
Rsl24d1	3.9%	4.8%	0.9	1486
LOC100362572	24.7%	25.6%	0.9	1487
LOC108348139	3.5%	4.4%	0.9	1488
LOC102546716	3.5%	4.4%	0.9	1489
Atxn7	3.1%	4.0%	0.9	1490
Pnpo	19.6%	20.5%	0.9	1491
Spag7	2.7%	3.6%	0.9	1492
Ppt1	2.7%	3.6%	0.9	1493
Bnip2	2.0%	2.8%	0.8	1494
LOC100912163	2.0%	2.8%	0.8	1495
Irx1	2.0%	2.8%	0.8	1496
Irs3	2.0%	2.8%	0.8	1497
LOC103691688	5.9%	6.7%	0.8	1498
Rps14	47.5%	48.3%	0.8	1499
LOC108352929	1.6%	2.4%	0.8	1500
Acly	30.6%	31.4%	0.8	1501
Mmp14	5.5%	6.3%	0.8	1502
Hs6st1	1.2%	2.0%	0.8	1503
Dab1	1.2%	2.0%	0.8	1504
Arglu1	13.3%	14.1%	0.8	1505
Slc7a5	0.8%	1.6%	0.8	1506
Prickle1	0.8%	1.6%	0.8	1507
Spsb4	0.8%	1.6%	0.8	1508
Isy1	0.8%	1.6%	0.8	1509
Chd3	0.8%	1.6%	0.8	1510
LOC686461	0.8%	1.6%	0.8	1511
LOC362695	0.8%	1.6%	0.8	1512

Tspyl2	0.8%	1.6%	0.8	1513
Jak3	0.8%	1.6%	0.8	1514
Acer3	4.7%	5.5%	0.8	1515
Cbx8	0.4%	1.2%	0.8	1516
LOC100912534	0.4%	1.2%	0.8	1517
Fhl2	0.4%	1.2%	0.8	1518
Marcksl1	0.4%	1.2%	0.8	1519
Bnc2	0.4%	1.2%	0.8	1520
P2rx7	0.4%	1.2%	0.8	1521
Tmem45b	0.4%	1.2%	0.8	1522
G4	0.4%	1.2%	0.8	1523
LOC691736	0.4%	1.2%	0.8	1524
LOC100909755	0.4%	1.2%	0.8	1525
RGD1563307	0.4%	1.2%	0.8	1526
Vom2r52	0.4%	1.2%	0.8	1527
LOC102549441	0.4%	1.2%	0.8	1528
LOC108352085	0.4%	1.2%	0.8	1529
Rin2	4.3%	5.1%	0.8	1530
Lgals3bp	4.3%	5.1%	0.8	1531
LOC108349187	0.0%	0.8%	0.8	1532
LOC108349159	0.0%	0.8%	0.8	1533
RGD1564515	0.0%	0.8%	0.8	1534
Fscn1	0.0%	0.8%	0.8	1535
LOC102552459	0.0%	0.8%	0.8	1536
Cep85	0.0%	0.8%	0.8	1537
LOC100910979	0.0%	0.8%	0.8	1538
Ptpn22	0.0%	0.8%	0.8	1539
Arhgap25	0.0%	0.8%	0.8	1540
Stambpl1	0.0%	0.8%	0.8	1541
LOC100911104	0.0%	0.8%	0.8	1542
LOC688022	0.0%	0.8%	0.8	1543
Dsn1	0.0%	0.8%	0.8	1544
LOC103694726	0.0%	0.8%	0.8	1545
Scamp5	0.0%	0.8%	0.8	1546
LOC317165	0.0%	0.8%	0.8	1547
LOC102556643	0.0%	0.8%	0.8	1548
Cnr2	0.0%	0.8%	0.8	1549
Rpl39l	0.0%	0.8%	0.8	1550
Esam	0.0%	0.8%	0.8	1551
Pld4	0.0%	0.8%	0.8	1552
Sh2b2	0.0%	0.8%	0.8	1553
MGC114492	0.0%	0.8%	0.8	1554
Ncf1	0.0%	0.8%	0.8	1555
Cd93	0.0%	0.8%	0.8	1556
LOC100911260	0.0%	0.8%	0.8	1557
Gatm	0.0%	0.8%	0.8	1558
Fcgr2a	0.0%	0.8%	0.8	1559
Cd83	0.0%	0.8%	0.8	1560
RT1-T24-3	0.0%	0.8%	0.8	1561
Lat	0.0%	0.8%	0.8	1562
LOC108352212	0.0%	0.8%	0.8	1563
Il15	0.0%	0.8%	0.8	1564
Gbp4	0.0%	0.8%	0.8	1565
Fcna	0.0%	0.8%	0.8	1566
Cd37	0.0%	0.8%	0.8	1567
Pak2	0.0%	0.8%	0.8	1568
Med18	0.0%	0.8%	0.8	1569
Ankmy2	0.0%	0.8%	0.8	1570
LOC102548985	0.0%	0.8%	0.8	1571
Gpnmb	0.0%	0.8%	0.8	1572
Tek	0.0%	0.8%	0.8	1573
Emp3	0.0%	0.8%	0.8	1574
Akr1b8	0.0%	0.8%	0.8	1575
Nusap1	0.0%	0.8%	0.8	1576
Amy2a3	0.0%	0.8%	0.8	1577
Fastkd1	0.0%	0.8%	0.8	1578

Hps3	0.0%	0.8%	0.8	1579
Parm1	0.0%	0.8%	0.8	1580
LOC100909786	0.0%	0.8%	0.8	1581
RT1-CE12	0.0%	0.8%	0.8	1582
LOC102555392	0.0%	0.8%	0.8	1583
Swsap1	0.0%	0.8%	0.8	1584
LOC103690049	0.0%	0.8%	0.8	1585
RGD1560775	0.0%	0.8%	0.8	1586
Pde2a	0.0%	0.8%	0.8	1587
Prkcq	0.0%	0.8%	0.8	1588
RT1-O1	0.0%	0.8%	0.8	1589
Fbxo25	0.0%	0.8%	0.8	1590
Ehf	0.0%	0.8%	0.8	1591
LOC100911215	0.0%	0.8%	0.8	1592
Mmp12	0.0%	0.8%	0.8	1593
Exog	0.0%	0.8%	0.8	1594
LOC304239	0.0%	0.8%	0.8	1595
Sp110	3.5%	4.3%	0.8	1596
Bcas3	3.1%	3.9%	0.8	1597
Dnajb6	3.1%	3.9%	0.8	1598
Agfg2	2.7%	3.5%	0.8	1599
Avpr1a	2.7%	3.5%	0.8	1600
Hdhd2	2.7%	3.5%	0.8	1601
LOC100910474	2.4%	3.1%	0.8	1602
Myo19	2.0%	2.7%	0.8	1603
Dock8	2.0%	2.7%	0.8	1604
LOC100362342	2.0%	2.7%	0.8	1605
Asic1	2.0%	2.7%	0.8	1606
LOC100912037	2.0%	2.7%	0.8	1607
LOC100361479	14.1%	14.9%	0.8	1608
Arl6	1.6%	2.3%	0.7	1609
Hmgb3	1.6%	2.3%	0.7	1610
Alppl2	1.6%	2.3%	0.7	1611
Clic1	1.6%	2.3%	0.7	1612
Peg3	1.6%	2.3%	0.7	1613
Faap20	1.6%	2.3%	0.7	1614
L3hypdh	1.2%	1.9%	0.7	1615
LOC100910660	1.2%	1.9%	0.7	1616
Dnmt3a	1.2%	1.9%	0.7	1617
Adora1	1.2%	1.9%	0.7	1618
Slf2	1.2%	1.9%	0.7	1619
Acat2l1	1.2%	1.9%	0.7	1620
Shisa5	5.1%	5.8%	0.7	1621
LOC681282	0.8%	1.5%	0.7	1622
Vprbp	0.8%	1.5%	0.7	1623
Klhl5	0.8%	1.5%	0.7	1624
LOC497899	0.8%	1.5%	0.7	1625
LOC679565	0.8%	1.5%	0.7	1626
Zfand1	0.8%	1.5%	0.7	1627
LOC102556827	0.8%	1.5%	0.7	1628
Tcf3	0.8%	1.5%	0.7	1629
Rbm14	0.8%	1.5%	0.7	1630
Prkag2	0.8%	1.5%	0.7	1631
Pskh1	0.8%	1.5%	0.7	1632
Csrnp2	0.8%	1.5%	0.7	1633
Trak1	4.7%	5.4%	0.7	1634
Nat9	4.7%	5.4%	0.7	1635
LOC102557259	0.4%	1.1%	0.7	1636
LOC108348114	0.4%	1.1%	0.7	1637
Gcnt2	0.4%	1.1%	0.7	1638
LOC102553302	0.4%	1.1%	0.7	1639
MGC105567	0.4%	1.1%	0.7	1640
G2e3	0.4%	1.1%	0.7	1641
LOC103695357	0.4%	1.1%	0.7	1642
Cd9	0.4%	1.1%	0.7	1643
Ubr1	0.4%	1.1%	0.7	1644

Fam129c	0.4%	1.1%	0.7	1645
Cyp11b1	0.4%	1.1%	0.7	1646
Mnd1	0.4%	1.1%	0.7	1647
LOC102553658	0.4%	1.1%	0.7	1648
LOC301444	0.4%	1.1%	0.7	1649
Npdc1	0.4%	1.1%	0.7	1650
Etfa	96.1%	96.8%	0.7	1651
LOC108348102	0.0%	0.7%	0.7	1652
Nup214	0.0%	0.7%	0.7	1653
Sox4	0.0%	0.7%	0.7	1654
Gzmbl1	0.0%	0.7%	0.7	1655
LOC100912009	0.0%	0.7%	0.7	1656
March9	0.0%	0.7%	0.7	1657
LOC100910270	0.0%	0.7%	0.7	1658
Rars2	0.0%	0.7%	0.7	1659
LOC680329	0.0%	0.7%	0.7	1660
Rcsd1	0.0%	0.7%	0.7	1661
Cpne4	0.0%	0.7%	0.7	1662
Sik1	0.0%	0.7%	0.7	1663
Olr1	0.0%	0.7%	0.7	1664
LOC684193	0.0%	0.7%	0.7	1665
LOC691829	0.0%	0.7%	0.7	1666
LOC100910708	0.0%	0.7%	0.7	1667
Flt1	0.0%	0.7%	0.7	1668
MGC105649	0.0%	0.7%	0.7	1669
Ly6c	0.0%	0.7%	0.7	1670
Rad51b	0.0%	0.7%	0.7	1671
Cdc42ep3	0.0%	0.7%	0.7	1672
Arhgap33	0.0%	0.7%	0.7	1673
Draxin	0.0%	0.7%	0.7	1674
LOC100363289	0.0%	0.7%	0.7	1675
Capg	0.0%	0.7%	0.7	1676
Senp1	0.0%	0.7%	0.7	1677
Pgm2l1	0.0%	0.7%	0.7	1678
Cd5	0.0%	0.7%	0.7	1679
Akr1b1	0.0%	0.7%	0.7	1680
Egr2	0.0%	0.7%	0.7	1681
Cenpo	0.0%	0.7%	0.7	1682
NEWGENE_1311658	0.0%	0.7%	0.7	1683
Tcf7	0.0%	0.7%	0.7	1684
S100a11	0.0%	0.7%	0.7	1685
Acmsd	0.0%	0.7%	0.7	1686
Nr4a3	0.0%	0.7%	0.7	1687
Rnf34	0.0%	0.7%	0.7	1688
LOC102550225	0.0%	0.7%	0.7	1689
RT1-N3	0.0%	0.7%	0.7	1690
LOC102549619	0.0%	0.7%	0.7	1691
Ints7	0.0%	0.7%	0.7	1692
Cyb5d1	0.0%	0.7%	0.7	1693
Spi1	0.0%	0.7%	0.7	1694
Adcy7	0.0%	0.7%	0.7	1695
RGD2320734	0.0%	0.7%	0.7	1696
Ttf2	0.0%	0.7%	0.7	1697
Tpte2	0.0%	0.7%	0.7	1698
Clec7a	0.0%	0.7%	0.7	1699
Tm4sf1	0.0%	0.7%	0.7	1700
LOC103690324	0.0%	0.7%	0.7	1701
Mcm3	0.0%	0.7%	0.7	1702
LOC103694035	0.0%	0.7%	0.7	1703
LOC103693254	0.0%	0.7%	0.7	1704
LOC102555316	0.0%	0.7%	0.7	1705
LOC498276	0.0%	0.7%	0.7	1706
Pltp	0.0%	0.7%	0.7	1707
Inppl1	0.0%	0.7%	0.7	1708
Nkx2-1	0.0%	0.7%	0.7	1709
LOC102552721	0.0%	0.7%	0.7	1710

Ptger4	0.0%	0.7%	0.7	1711
LOC103691023	0.0%	0.7%	0.7	1712
Clic2	0.0%	0.7%	0.7	1713
Evi2a	0.0%	0.7%	0.7	1714
Tmem8b	0.0%	0.7%	0.7	1715
Plac8	0.0%	0.7%	0.7	1716
Hcls1	0.0%	0.7%	0.7	1717
Dusp7	0.0%	0.7%	0.7	1718
Mxd1	0.0%	0.7%	0.7	1719
LOC103691274	0.0%	0.7%	0.7	1720
Rab43	0.0%	0.7%	0.7	1721
LOC100361920	3.9%	4.6%	0.7	1722
LOC100911728	3.5%	4.2%	0.7	1723
Rxrg	3.1%	3.8%	0.7	1724
LOC679881	3.1%	3.8%	0.7	1725
Pet100	7.1%	7.8%	0.7	1726
Adam28	6.7%	7.4%	0.7	1727
Pphln1	6.7%	7.4%	0.7	1728
Adap2	6.7%	7.4%	0.7	1729
Trit1	2.4%	3.0%	0.7	1730
LOC102554194	6.3%	7.0%	0.7	1731
Cxcl16	2.0%	2.6%	0.7	1732
Slc6a6	2.0%	2.6%	0.7	1733
Xpa	2.0%	2.6%	0.7	1734
Sh3bgrl3	2.0%	2.6%	0.7	1735
UST4r	2.0%	2.6%	0.7	1736
LOC108352814	2.0%	2.6%	0.7	1737
Lss	2.0%	2.6%	0.7	1738
Arl14ep	2.0%	2.6%	0.7	1739
Abhd16a	1.6%	2.2%	0.7	1740
Cd2ap	1.2%	1.8%	0.7	1741
Smtn	1.2%	1.8%	0.7	1742
LOC691960	1.2%	1.8%	0.7	1743
Tob2	1.2%	1.8%	0.7	1744
LOC102554034	1.2%	1.8%	0.7	1745
Myh9	9.4%	10.1%	0.7	1746
Hdac11	0.8%	1.4%	0.7	1747
LOC100910181	0.8%	1.4%	0.7	1748
Hmox2-ps1	0.8%	1.4%	0.7	1749
Pde6d	0.8%	1.4%	0.7	1750
LOC108348349	0.8%	1.4%	0.7	1751
LOC108348429	0.8%	1.4%	0.7	1752
Vcam1	0.8%	1.4%	0.7	1753
Pard3b	0.8%	1.4%	0.7	1754
Rnf214	0.8%	1.4%	0.7	1755
Lonrf2	0.8%	1.4%	0.7	1756
LOC100912731	0.8%	1.4%	0.7	1757
LOC108352004	0.8%	1.4%	0.7	1758
LOC690147	0.8%	1.4%	0.7	1759
LOC108350024	0.8%	1.4%	0.7	1760
Serpine3	0.4%	1.0%	0.6	1761
Slc25a45	0.4%	1.0%	0.6	1762
LOC103694863	0.4%	1.0%	0.6	1763
LOC102552469	0.4%	1.0%	0.6	1764
Zfp652	0.4%	1.0%	0.6	1765
Ttc30b	0.4%	1.0%	0.6	1766
LOC102549114	0.4%	1.0%	0.6	1767
Topbp1	0.4%	1.0%	0.6	1768
LOC102552920	0.4%	1.0%	0.6	1769
LOC103689966	0.4%	1.0%	0.6	1770
LOC108348620	0.4%	1.0%	0.6	1771
Irf8	0.4%	1.0%	0.6	1772
Tapbpl	0.4%	1.0%	0.6	1773
LOC367191	0.4%	1.0%	0.6	1774
Trim37	0.4%	1.0%	0.6	1775
LOC103692251	0.4%	1.0%	0.6	1776

Mthfsd	0.4%	1.0%	0.6	1777
Cep85l	0.4%	1.0%	0.6	1778
Msi2	4.3%	5.0%	0.6	1779
LOC108349530	0.0%	0.6%	0.6	1780
Rassf4	0.0%	0.6%	0.6	1781
Gen1	0.0%	0.6%	0.6	1782
Ppp1r16b	0.0%	0.6%	0.6	1783
MGC108823	0.0%	0.6%	0.6	1784
Elk1	0.0%	0.6%	0.6	1785
Ssbp4	0.0%	0.6%	0.6	1786
LOC100911870	0.0%	0.6%	0.6	1787
Pdik1l	0.0%	0.6%	0.6	1788
Tia1	0.0%	0.6%	0.6	1789
Myl4	0.0%	0.6%	0.6	1790
Gimap5	0.0%	0.6%	0.6	1791
Map3k12	0.0%	0.6%	0.6	1792
Gna13	0.0%	0.6%	0.6	1793
LOC102548633	0.0%	0.6%	0.6	1794
Tlr2	0.0%	0.6%	0.6	1795
Lilrb3	0.0%	0.6%	0.6	1796
Pagr1	0.0%	0.6%	0.6	1797
LOC102549747	0.0%	0.6%	0.6	1798
Leng8	0.0%	0.6%	0.6	1799
Camk4	0.0%	0.6%	0.6	1800
Ahnak	0.0%	0.6%	0.6	1801
Mrps10	0.0%	0.6%	0.6	1802
LOC103692137	0.0%	0.6%	0.6	1803
Itsn1	0.0%	0.6%	0.6	1804
LOC102550016	0.0%	0.6%	0.6	1805
Ccdc82	0.0%	0.6%	0.6	1806
Twf2	0.0%	0.6%	0.6	1807
RGD1311164	0.0%	0.6%	0.6	1808
Samsn1	0.0%	0.6%	0.6	1809
Qrich1	0.0%	0.6%	0.6	1810
LOC499235	0.0%	0.6%	0.6	1811
Lipe	0.0%	0.6%	0.6	1812
Csnk1g1	0.0%	0.6%	0.6	1813
Inpp5d	0.0%	0.6%	0.6	1814
Procr	0.0%	0.6%	0.6	1815
Hist1h2ac	0.0%	0.6%	0.6	1816
Mocs3	0.0%	0.6%	0.6	1817
S100a9	0.0%	0.6%	0.6	1818
LOC688906	0.0%	0.6%	0.6	1819
Cdkn1c	0.0%	0.6%	0.6	1820
LOC102550036	0.0%	0.6%	0.6	1821
Knstrn	0.0%	0.6%	0.6	1822
LOC678774	0.0%	0.6%	0.6	1823
Plxnd1	0.0%	0.6%	0.6	1824
LOC102551862	0.0%	0.6%	0.6	1825
LOC103692066	0.0%	0.6%	0.6	1826
Grin2b	0.0%	0.6%	0.6	1827
LOC108351880	0.0%	0.6%	0.6	1828
Klrd1	0.0%	0.6%	0.6	1829
Timp1	0.0%	0.6%	0.6	1830
LOC502618	0.0%	0.6%	0.6	1831
Anxa1	0.0%	0.6%	0.6	1832
Ankrd10	0.0%	0.6%	0.6	1833
Klri1	0.0%	0.6%	0.6	1834
Clec4g	0.0%	0.6%	0.6	1835
Hcst	0.0%	0.6%	0.6	1836
LOC102550530	0.0%	0.6%	0.6	1837
LOC100910710	0.0%	0.6%	0.6	1838
Mmd	0.0%	0.6%	0.6	1839
Pvt1	0.0%	0.6%	0.6	1840
Synpo2l	0.0%	0.6%	0.6	1841
Tm6sf1	0.0%	0.6%	0.6	1842

LOC108348136	0.0%	0.6%	0.6	1843
Irf4	0.0%	0.6%	0.6	1844
RT1-CE1	0.0%	0.6%	0.6	1845
Sash3	0.0%	0.6%	0.6	1846
LOC690862	0.0%	0.6%	0.6	1847
Kctd18	3.9%	4.6%	0.6	1848
LOC100361547	86.7%	87.3%	0.6	1849
Fxr1	3.1%	3.8%	0.6	1850
Rell1	3.1%	3.8%	0.6	1851
Rnf220	2.7%	3.4%	0.6	1852
Nat8f4	2.7%	3.4%	0.6	1853
Aagab	2.7%	3.4%	0.6	1854
LOC108350065	2.7%	3.4%	0.6	1855
Coq2	2.7%	3.4%	0.6	1856
Zbtb11	1.6%	2.2%	0.6	1857
LOC103690102	1.6%	2.2%	0.6	1858
Timm44	1.6%	2.2%	0.6	1859
LOC108348504	1.6%	2.2%	0.6	1860
Rpa2	1.6%	2.2%	0.6	1861
Cptp	1.6%	2.2%	0.6	1862
Matr3-ps2	5.5%	6.1%	0.6	1863
Vti1a	1.2%	1.8%	0.6	1864
Rem2	1.2%	1.8%	0.6	1865
LOC103689941	1.2%	1.8%	0.6	1866
Atxn7l1	1.2%	1.8%	0.6	1867
Rpusd4	1.2%	1.8%	0.6	1868
Rab4a	1.2%	1.8%	0.6	1869
Srgap2	1.2%	1.8%	0.6	1870
Mfsd10	1.2%	1.8%	0.6	1871
LOC108353713	1.2%	1.8%	0.6	1872
Egln3	1.2%	1.8%	0.6	1873
Plcxd2	5.1%	5.7%	0.6	1874
Kcnj15	0.8%	1.4%	0.6	1875
Nuak2	0.8%	1.4%	0.6	1876
Polr2h	0.8%	1.4%	0.6	1877
Aff1	0.8%	1.4%	0.6	1878
LOC100912416	0.8%	1.4%	0.6	1879
LOC108348513	0.8%	1.4%	0.6	1880
LOC103691822	0.8%	1.4%	0.6	1881
LOC102548801	0.8%	1.4%	0.6	1882
Casp1	0.8%	1.4%	0.6	1883
Plekhf1	0.8%	1.4%	0.6	1884
Dll1	0.8%	1.4%	0.6	1885
LOC103693047	0.8%	1.4%	0.6	1886
Slc25a30	4.7%	5.3%	0.6	1887
Gnpat	0.4%	1.0%	0.6	1888
Akr1cl	0.4%	1.0%	0.6	1889
Haus3	0.4%	1.0%	0.6	1890
Zbed6	0.4%	1.0%	0.6	1891
LOC100361056	0.4%	1.0%	0.6	1892
Ogg1	0.4%	1.0%	0.6	1893
LOC103692882	0.4%	1.0%	0.6	1894
Gnpda1	0.4%	1.0%	0.6	1895
Fancl	0.4%	1.0%	0.6	1896
LOC108348140	0.4%	1.0%	0.6	1897
Chmp4b	0.4%	1.0%	0.6	1898
Ppp3cc	0.4%	1.0%	0.6	1899
LOC103694899	0.4%	1.0%	0.6	1900
Trmt11	0.4%	1.0%	0.6	1901
LOC108351251	0.4%	1.0%	0.6	1902
Rmnd5b	0.4%	1.0%	0.6	1903
Rilpl1	0.4%	1.0%	0.6	1904
Ccny	0.4%	1.0%	0.6	1905
LOC102551184	0.4%	1.0%	0.6	1906
Mtmr7	0.4%	1.0%	0.6	1907
Slc41a1	0.4%	1.0%	0.6	1908

LOC108351411	0.4%	1.0%	0.6	1909
RGD1311739	0.4%	1.0%	0.6	1910
LOC102556190	0.4%	1.0%	0.6	1911
Gpr137b	0.4%	1.0%	0.6	1912
RGD1564958	0.4%	1.0%	0.6	1913
LOC103689951	0.4%	1.0%	0.6	1914
Serpine1	0.4%	1.0%	0.6	1915
RGD1309540	0.4%	1.0%	0.6	1916
Zfp57	0.4%	1.0%	0.6	1917
Slc44a4	0.4%	1.0%	0.6	1918
LOC100910706	0.4%	1.0%	0.6	1919
Fdxacb1	0.4%	1.0%	0.6	1920
Trpm2	4.3%	4.9%	0.6	1921
Srsf9	4.3%	4.9%	0.6	1922
LOC108348282	0.0%	0.6%	0.6	1923
Smpd3	0.0%	0.6%	0.6	1924
LOC103690372	0.0%	0.6%	0.6	1925
Spata7	0.0%	0.6%	0.6	1926
Plekho2	0.0%	0.6%	0.6	1927
Stxbp1	0.0%	0.6%	0.6	1928
Sit1	0.0%	0.6%	0.6	1929
Cln6	0.0%	0.6%	0.6	1930
Clec2e	0.0%	0.6%	0.6	1931
LOC108349403	0.0%	0.6%	0.6	1932
Tnfrsf14	0.0%	0.6%	0.6	1933
Sema6a	0.0%	0.6%	0.6	1934
LOC108350684	0.0%	0.6%	0.6	1935
Stk26	0.0%	0.6%	0.6	1936
Nol3	0.0%	0.6%	0.6	1937
LOC103689954	0.0%	0.6%	0.6	1938
Usp13	0.0%	0.6%	0.6	1939
Mtmr9	0.0%	0.6%	0.6	1940
Rgs2	0.0%	0.6%	0.6	1941
Arhgap22	0.0%	0.6%	0.6	1942
LOC103691897	0.0%	0.6%	0.6	1943
LOC103689971	0.0%	0.6%	0.6	1944
Eif4e3	0.0%	0.6%	0.6	1945
B3galnt1	0.0%	0.6%	0.6	1946
Pot1b	0.0%	0.6%	0.6	1947
LOC102548220	0.0%	0.6%	0.6	1948
Ube2c	0.0%	0.6%	0.6	1949
LOC103693959	0.0%	0.6%	0.6	1950
Gpsm3	0.0%	0.6%	0.6	1951
LOC102551019	0.0%	0.6%	0.6	1952
Chrna7	0.0%	0.6%	0.6	1953
Cpg1	0.0%	0.6%	0.6	1954
Aplp1	0.0%	0.6%	0.6	1955
Nrip3	0.0%	0.6%	0.6	1956
Fnbp1	0.0%	0.6%	0.6	1957
Kazn	0.0%	0.6%	0.6	1958
Cntnap5c	0.0%	0.6%	0.6	1959
Hist1h1b	0.0%	0.6%	0.6	1960
Tie1	0.0%	0.6%	0.6	1961
Rarg	0.0%	0.6%	0.6	1962
Lenep	0.0%	0.6%	0.6	1963
LOC102552000	0.0%	0.6%	0.6	1964
Cd79a	0.0%	0.6%	0.6	1965
Abcg1	0.0%	0.6%	0.6	1966
Gas8	0.0%	0.6%	0.6	1967
Apold1	0.0%	0.6%	0.6	1968
LOC100909791	0.0%	0.6%	0.6	1969
LOC103690008	0.0%	0.6%	0.6	1970
Ints13	0.0%	0.6%	0.6	1971
Hist1h2bk	0.0%	0.6%	0.6	1972
LOC102553193	0.0%	0.6%	0.6	1973
LOC102553847	0.0%	0.6%	0.6	1974

Ikzf3	0.0%	0.6%	0.6	1975
Scimp	0.0%	0.6%	0.6	1976
LOC100910056	0.0%	0.6%	0.6	1977
Lrrc8c	0.0%	0.6%	0.6	1978
Pdcd1	0.0%	0.6%	0.6	1979
Fundc2	0.0%	0.6%	0.6	1980
Hopx	0.0%	0.6%	0.6	1981
Tmem88	0.0%	0.6%	0.6	1982
B3gnt7	0.0%	0.6%	0.6	1983
Itgax	0.0%	0.6%	0.6	1984
LOC100910172	0.0%	0.6%	0.6	1985
Adgre1	0.0%	0.6%	0.6	1986
Atp2b3	0.0%	0.6%	0.6	1987
Mki67	0.0%	0.6%	0.6	1988
Adm2	0.0%	0.6%	0.6	1989
Glcci1	0.0%	0.6%	0.6	1990
Mcam	0.0%	0.6%	0.6	1991
Snx20	0.0%	0.6%	0.6	1992
C1qtnf1	0.0%	0.6%	0.6	1993
Ncf4	0.0%	0.6%	0.6	1994
Spint1	0.0%	0.6%	0.6	1995
Tdp1	0.0%	0.6%	0.6	1996
Cfp	0.0%	0.6%	0.6	1997
LOC108349640	0.0%	0.6%	0.6	1998
Abcd2	0.0%	0.6%	0.6	1999
Ect2	0.0%	0.6%	0.6	2000
LOC108348474	0.0%	0.6%	0.6	2001
Ribc1	0.0%	0.6%	0.6	2002
Ccl22	0.0%	0.6%	0.6	2003
LOC684762	0.0%	0.6%	0.6	2004
Sh3bp1	0.0%	0.6%	0.6	2005
Wdr35	0.0%	0.6%	0.6	2006
LOC102557213	0.0%	0.6%	0.6	2007
Slc1a5	0.0%	0.6%	0.6	2008
Slc15a3	0.0%	0.6%	0.6	2009
RT1-M6-1	0.0%	0.6%	0.6	2010
Slc36a4	0.0%	0.6%	0.6	2011
LOC683761	0.0%	0.6%	0.6	2012
LOC100364769	0.0%	0.6%	0.6	2013
LOC689757	0.0%	0.6%	0.6	2014
Map4k1	0.0%	0.6%	0.6	2015
Fam60a	0.0%	0.6%	0.6	2016
Elmod3	0.0%	0.6%	0.6	2017
RT1-CE2	0.0%	0.6%	0.6	2018
Pced1b	0.0%	0.6%	0.6	2019
Birc5	0.0%	0.6%	0.6	2020
Mxra7	0.0%	0.6%	0.6	2021
Slc44a2	0.0%	0.6%	0.6	2022
LOC108349489	0.0%	0.6%	0.6	2023
Znrd1as1	0.0%	0.6%	0.6	2024
LOC108349906	0.0%	0.6%	0.6	2025
Ms4a6c	0.0%	0.6%	0.6	2026
Sh2d2a	0.0%	0.6%	0.6	2027
Fli1	0.0%	0.6%	0.6	2028
LOC102551730	0.0%	0.6%	0.6	2029
Ms4a6bl	0.0%	0.6%	0.6	2030
LOC100911825	0.0%	0.6%	0.6	2031
LOC103691988	0.0%	0.6%	0.6	2032
Arrb2	0.0%	0.6%	0.6	2033
Emcn	0.0%	0.6%	0.6	2034
Snx33	0.0%	0.6%	0.6	2035
Stab1	0.0%	0.6%	0.6	2036
LOC100910385	0.0%	0.6%	0.6	2037
LOC102552718	0.0%	0.6%	0.6	2038
Dync1li1	0.0%	0.6%	0.6	2039
LOC108353253	0.0%	0.6%	0.6	2040

Lima1	3.9%	4.5%	0.6	2041
Gskip	3.9%	4.5%	0.6	2042
Tmem120a	3.9%	4.5%	0.6	2043
Plin3	12.2%	12.7%	0.6	2044
Nme5	3.1%	3.7%	0.5	2045
Gem	2.7%	3.3%	0.5	2046
LOC108351291	2.7%	3.3%	0.5	2047
Gripap1	2.7%	3.3%	0.5	2048
Whsc1l1	2.7%	3.3%	0.5	2049
LOC102549890	2.7%	3.3%	0.5	2050
Sectm1b	2.0%	2.5%	0.5	2051
LOC103693118	2.0%	2.5%	0.5	2052
Mfn1	1.6%	2.1%	0.5	2053
Cnot8	1.6%	2.1%	0.5	2054
Nubpl	1.6%	2.1%	0.5	2055
Slc11a1	1.6%	2.1%	0.5	2056
Snw1	5.5%	6.0%	0.5	2057
Tppp	1.2%	1.7%	0.5	2058
Dis3l2	1.2%	1.7%	0.5	2059
Rap1gds1	1.2%	1.7%	0.5	2060
Stk3	1.2%	1.7%	0.5	2061
Inpp5b	1.2%	1.7%	0.5	2062
Entpd2	1.2%	1.7%	0.5	2063
Fam160a1	0.8%	1.3%	0.5	2064
RGD1565689	0.8%	1.3%	0.5	2065
Ppp1r10	0.8%	1.3%	0.5	2066
Slc45a3	0.8%	1.3%	0.5	2067
Smad7	0.8%	1.3%	0.5	2068
LOC108353238	0.8%	1.3%	0.5	2069
Hsd17b7	4.7%	5.2%	0.5	2070
Stx7	4.7%	5.2%	0.5	2071
Smim22	4.7%	5.2%	0.5	2072
Manea	0.4%	0.9%	0.5	2073
Jak2	0.4%	0.9%	0.5	2074
Usp28	0.4%	0.9%	0.5	2075
Rhobtb3	0.4%	0.9%	0.5	2076
Mgme1	0.4%	0.9%	0.5	2077
Tbc1d4	0.4%	0.9%	0.5	2078
Cdk19	0.4%	0.9%	0.5	2079
Tbc1d2b	0.4%	0.9%	0.5	2080
Car6	0.4%	0.9%	0.5	2081
Them4	0.4%	0.9%	0.5	2082
Gpr15	0.4%	0.9%	0.5	2083
Nlrp3	0.4%	0.9%	0.5	2084
LOC102547897	0.4%	0.9%	0.5	2085
RGD1564887	0.4%	0.9%	0.5	2086
LOC688925	0.4%	0.9%	0.5	2087
Pip5k1c	0.4%	0.9%	0.5	2088
Mnda	0.4%	0.9%	0.5	2089
Cldn23	0.4%	0.9%	0.5	2090
Tspan3	0.4%	0.9%	0.5	2091
Camk2n1	0.4%	0.9%	0.5	2092
Kpna4	0.4%	0.9%	0.5	2093
Themis2	0.4%	0.9%	0.5	2094
Erich6b	0.4%	0.9%	0.5	2095
LOC689229	0.4%	0.9%	0.5	2096
Bicdl2	0.0%	0.5%	0.5	2097
Fam83f	0.0%	0.5%	0.5	2098
Arhgap39	0.0%	0.5%	0.5	2099
Casp16	0.0%	0.5%	0.5	2100
Trpm6	0.0%	0.5%	0.5	2101
LOC103693657	0.0%	0.5%	0.5	2102
Gzmbl3	0.0%	0.5%	0.5	2103
Rnf157	0.0%	0.5%	0.5	2104
LOC108349920	0.0%	0.5%	0.5	2105
LOC102551532	0.0%	0.5%	0.5	2106

Slc7a11	0.0%	0.5%	0.5	2107
LOC103691922	0.0%	0.5%	0.5	2108
Myl9	0.0%	0.5%	0.5	2109
LOC689770	0.0%	0.5%	0.5	2110
Igsf7	0.0%	0.5%	0.5	2111
LOC100363500	0.0%	0.5%	0.5	2112
Rn5-8s	0.0%	0.5%	0.5	2113
Arl4c	0.0%	0.5%	0.5	2114
Lcp2	0.0%	0.5%	0.5	2115
Gfpt2	0.0%	0.5%	0.5	2116
Mcm2	0.0%	0.5%	0.5	2117
Brpf1	0.0%	0.5%	0.5	2118
Duxbl1	0.0%	0.5%	0.5	2119
Lhx2	0.0%	0.5%	0.5	2120
LOC103691005	0.0%	0.5%	0.5	2121
Mip	0.0%	0.5%	0.5	2122
LOC103693587	0.0%	0.5%	0.5	2123
Adamts4	0.0%	0.5%	0.5	2124
Mlh3	0.0%	0.5%	0.5	2125
Syk	0.0%	0.5%	0.5	2126
Gnat3	0.0%	0.5%	0.5	2127
LOC108350581	0.0%	0.5%	0.5	2128
Plcl1	0.0%	0.5%	0.5	2129
Il10ra	0.0%	0.5%	0.5	2130
Amhr2	0.0%	0.5%	0.5	2131
LOC108351407	0.0%	0.5%	0.5	2132
RGD1565679	0.0%	0.5%	0.5	2133
Endod1	0.0%	0.5%	0.5	2134
Casz1	0.0%	0.5%	0.5	2135
C1qb	0.0%	0.5%	0.5	2136
LOC108348047	0.0%	0.5%	0.5	2137
Ica1	0.0%	0.5%	0.5	2138
S100a8	0.0%	0.5%	0.5	2139
LOC102552576	0.0%	0.5%	0.5	2140
Pik3cg	0.0%	0.5%	0.5	2141
Sapcd2	0.0%	0.5%	0.5	2142
Slfn1	0.0%	0.5%	0.5	2143
Glyr1	0.0%	0.5%	0.5	2144
LOC102549383	0.0%	0.5%	0.5	2145
Cd6	0.0%	0.5%	0.5	2146
RT1-CE15	0.0%	0.5%	0.5	2147
LOC108352537	0.0%	0.5%	0.5	2148
LOC102554136	0.0%	0.5%	0.5	2149
LOC102553801	0.0%	0.5%	0.5	2150
LOC102553462	0.0%	0.5%	0.5	2151
Arhgef6	0.0%	0.5%	0.5	2152
LOC499240	0.0%	0.5%	0.5	2153
Oit3	0.0%	0.5%	0.5	2154
Dock10	0.0%	0.5%	0.5	2155
Armc4	0.0%	0.5%	0.5	2156
Cd300ld	0.0%	0.5%	0.5	2157
Sfxn3	0.0%	0.5%	0.5	2158
Tet3	0.0%	0.5%	0.5	2159
Rgs12	0.0%	0.5%	0.5	2160
Eno3	0.0%	0.5%	0.5	2161
Clec14a	0.0%	0.5%	0.5	2162
Ppp1r9b	0.0%	0.5%	0.5	2163
Rab5a	0.0%	0.5%	0.5	2164
Six5	0.0%	0.5%	0.5	2165
Cdca3	0.0%	0.5%	0.5	2166
LOC100910575	0.0%	0.5%	0.5	2167
Cntd1	0.0%	0.5%	0.5	2168
Zfp939	0.0%	0.5%	0.5	2169
LOC103690114	0.0%	0.5%	0.5	2170
LOC108349552	0.0%	0.5%	0.5	2171
LOC108351831	0.0%	0.5%	0.5	2172

Prr12	0.0%	0.5%	0.5	2173
Pla2g7	0.0%	0.5%	0.5	2174
H19	0.0%	0.5%	0.5	2175
Akna	0.0%	0.5%	0.5	2176
Catsper2	0.0%	0.5%	0.5	2177
Rbm43	0.0%	0.5%	0.5	2178
Trmo	0.0%	0.5%	0.5	2179
LOC102548039	0.0%	0.5%	0.5	2180
Ttc8	0.0%	0.5%	0.5	2181
LOC499229	0.0%	0.5%	0.5	2182
LOC108352770	0.0%	0.5%	0.5	2183
Itgb7	0.0%	0.5%	0.5	2184
LOC102551251	0.0%	0.5%	0.5	2185
Plvap	0.0%	0.5%	0.5	2186
Ly86	0.0%	0.5%	0.5	2187
Wipf1	0.0%	0.5%	0.5	2188
LOC103690190	0.0%	0.5%	0.5	2189
Klre1	0.0%	0.5%	0.5	2190
LOC308990	0.0%	0.5%	0.5	2191
LOC500148	0.0%	0.5%	0.5	2192
Cfd	0.0%	0.5%	0.5	2193
Flna	0.0%	0.5%	0.5	2194
Gmip	0.0%	0.5%	0.5	2195
LOC102553429	0.0%	0.5%	0.5	2196
LOC689346	0.0%	0.5%	0.5	2197
Dusp2	0.0%	0.5%	0.5	2198
LOC100910623	0.0%	0.5%	0.5	2199
Chaf1a	0.0%	0.5%	0.5	2200
Grin2d	0.0%	0.5%	0.5	2201
Pfkp	0.0%	0.5%	0.5	2202
Tacc3	0.0%	0.5%	0.5	2203
LOC100360061	0.0%	0.5%	0.5	2204
LOC102555439	0.0%	0.5%	0.5	2205
Sh3kbp1	0.0%	0.5%	0.5	2206
Ggta1	0.0%	0.5%	0.5	2207
Bak1	0.0%	0.5%	0.5	2208
Sytl3	0.0%	0.5%	0.5	2209
Cd4	0.0%	0.5%	0.5	2210
NEWGENE_1305243	0.0%	0.5%	0.5	2211
Gata3	0.0%	0.5%	0.5	2212
Cdhr1	0.0%	0.5%	0.5	2213
Il17b	0.0%	0.5%	0.5	2214
Epsti1	0.0%	0.5%	0.5	2215
Ttbk1	0.0%	0.5%	0.5	2216
Fxyd2	0.0%	0.5%	0.5	2217
Nphs2	8.2%	8.7%	0.5	2218
Khsrp	3.5%	4.0%	0.5	2219
LOC102551209	2.7%	3.2%	0.5	2220
Herc6	2.7%	3.2%	0.5	2221
Nmnat3	2.4%	2.8%	0.4	2222
Lrrc45	2.0%	2.4%	0.4	2223
Rbmx11	2.0%	2.4%	0.4	2224
Hirip3	2.0%	2.4%	0.4	2225
Kpnb1	2.0%	2.4%	0.4	2226
LOC108350531	2.0%	2.4%	0.4	2227
LOC498424	2.0%	2.4%	0.4	2228
Bhlhe40	5.9%	6.3%	0.4	2229
Phf2	1.6%	2.0%	0.4	2230
St3gal4	1.6%	2.0%	0.4	2231
Trem3	1.6%	2.0%	0.4	2232
LOC102555598	1.6%	2.0%	0.4	2233
Wdr6	1.6%	2.0%	0.4	2234
Lhx8	1.6%	2.0%	0.4	2235
Acot3	1.2%	1.6%	0.4	2236
LOC102554161	1.2%	1.6%	0.4	2237
Snapc5	1.2%	1.6%	0.4	2238

Mknk1	1.2%	1.6%	0.4	2239
Rreb1	1.2%	1.6%	0.4	2240
Ndc1	1.2%	1.6%	0.4	2241
LOC299190	1.2%	1.6%	0.4	2242
Echdc1	5.1%	5.5%	0.4	2243
Nphp4	0.8%	1.2%	0.4	2244
LOC681117	0.8%	1.2%	0.4	2245
Vrk1	0.8%	1.2%	0.4	2246
Kdm2b	0.8%	1.2%	0.4	2247
Slc16a13	0.8%	1.2%	0.4	2248
LOC102555233	0.8%	1.2%	0.4	2249
Snx8	0.8%	1.2%	0.4	2250
Gemin2	0.8%	1.2%	0.4	2251
Tas1r1	0.8%	1.2%	0.4	2252
Pdzrn3	0.8%	1.2%	0.4	2253
Mcm4	0.8%	1.2%	0.4	2254
Thrap3	4.7%	5.1%	0.4	2255
Cnp	4.7%	5.1%	0.4	2256
Ntf3	0.4%	0.8%	0.4	2257
LOC102549750	0.4%	0.8%	0.4	2258
Adck1	0.4%	0.8%	0.4	2259
RGD1564613	0.4%	0.8%	0.4	2260
Dhx57	0.4%	0.8%	0.4	2261
LOC102548389	0.4%	0.8%	0.4	2262
Cpd	0.4%	0.8%	0.4	2263
LOC103694380	0.4%	0.8%	0.4	2264
Bcl7a	0.4%	0.8%	0.4	2265
Tmem164	0.4%	0.8%	0.4	2266
Ppwd1	0.4%	0.8%	0.4	2267
Dusp19	0.4%	0.8%	0.4	2268
Il1rap	0.4%	0.8%	0.4	2269
Tab1	0.4%	0.8%	0.4	2270
Knop1	0.4%	0.8%	0.4	2271
Tmem170a	0.4%	0.8%	0.4	2272
Lrig2	0.4%	0.8%	0.4	2273
Adh4	0.4%	0.8%	0.4	2274
Fam49a	0.4%	0.8%	0.4	2275
LOC103690124	0.4%	0.8%	0.4	2276
Mbnl1	0.4%	0.8%	0.4	2277
Eef2k	0.4%	0.8%	0.4	2278
Gpx3	0.4%	0.8%	0.4	2279
Zfp775	0.4%	0.8%	0.4	2280
LOC317456	0.4%	0.8%	0.4	2281
Prnp	0.4%	0.8%	0.4	2282
Cdca8	0.4%	0.8%	0.4	2283
LOC102554096	0.0%	0.4%	0.4	2284
Fam98b	0.0%	0.4%	0.4	2285
LOC100912393	0.0%	0.4%	0.4	2286
Lmnb2	0.0%	0.4%	0.4	2287
Rpe65	0.0%	0.4%	0.4	2288
Sufu	0.0%	0.4%	0.4	2289
Akr1b10	0.0%	0.4%	0.4	2290
Crtc2	0.0%	0.4%	0.4	2291
Agps	0.0%	0.4%	0.4	2292
LOC102549281	0.0%	0.4%	0.4	2293
RGD1309362	0.0%	0.4%	0.4	2294
LOC100363520	0.0%	0.4%	0.4	2295
Ly49s6	0.0%	0.4%	0.4	2296
Bcat1	0.0%	0.4%	0.4	2297
Pcdhgb5	0.0%	0.4%	0.4	2298
Tbx21	0.0%	0.4%	0.4	2299
LOC102547350	0.0%	0.4%	0.4	2300
Eif4e1b	0.0%	0.4%	0.4	2301
Tspan14	0.0%	0.4%	0.4	2302
Agbl4	0.0%	0.4%	0.4	2303
Lat2	0.0%	0.4%	0.4	2304

LOC108348833	0.0%	0.4%	0.4	2305
RGD1563620	0.0%	0.4%	0.4	2306
Chst4	0.0%	0.4%	0.4	2307
Ift80	0.0%	0.4%	0.4	2308
Plscr2	0.0%	0.4%	0.4	2309
LOC499796	0.0%	0.4%	0.4	2310
Sla2	0.0%	0.4%	0.4	2311
Erg	0.0%	0.4%	0.4	2312
RGD1562339	0.0%	0.4%	0.4	2313
Top2a	0.0%	0.4%	0.4	2314
Lpxn	0.0%	0.4%	0.4	2315
Zfp136	0.0%	0.4%	0.4	2316
LOC103694906	0.0%	0.4%	0.4	2317
Man1c1	0.0%	0.4%	0.4	2318
Tmem125	0.0%	0.4%	0.4	2319
LOC108349036	0.0%	0.4%	0.4	2320
Ppip5k2	0.0%	0.4%	0.4	2321
Smim3	0.0%	0.4%	0.4	2322
LOC103694020	0.0%	0.4%	0.4	2323
Gls	0.0%	0.4%	0.4	2324
Ppp1r18	0.0%	0.4%	0.4	2325
Klri2	0.0%	0.4%	0.4	2326
LOC108353083	0.0%	0.4%	0.4	2327
Ttbk2	0.0%	0.4%	0.4	2328
LOC103694908	0.0%	0.4%	0.4	2329
Arhgap27	0.0%	0.4%	0.4	2330
Cass4	0.0%	0.4%	0.4	2331
Adm	0.0%	0.4%	0.4	2332
Slc16a2	0.0%	0.4%	0.4	2333
Osbpl7	0.0%	0.4%	0.4	2334
Itpkb	0.0%	0.4%	0.4	2335
Extl2	0.0%	0.4%	0.4	2336
Trat1	0.0%	0.4%	0.4	2337
Fam129b	0.0%	0.4%	0.4	2338
Pcgf1	0.0%	0.4%	0.4	2339
Agpat4	0.0%	0.4%	0.4	2340
Slamf1	0.0%	0.4%	0.4	2341
Mapk12	0.0%	0.4%	0.4	2342
LOC103694996	0.0%	0.4%	0.4	2343
Pkn3	0.0%	0.4%	0.4	2344
Xpr1	0.0%	0.4%	0.4	2345
Ms4a7	0.0%	0.4%	0.4	2346
Hk1	0.0%	0.4%	0.4	2347
Ptk2b	0.0%	0.4%	0.4	2348
Vamp5	0.0%	0.4%	0.4	2349
LOC108350407	0.0%	0.4%	0.4	2350
Dtx1	0.0%	0.4%	0.4	2351
LOC108349703	0.0%	0.4%	0.4	2352
Bmyc	0.0%	0.4%	0.4	2353
LOC100909955	0.0%	0.4%	0.4	2354
Cd7	0.0%	0.4%	0.4	2355
LOC102547688	0.0%	0.4%	0.4	2356
RGD1304770	0.0%	0.4%	0.4	2357
Ccdc38	0.0%	0.4%	0.4	2358
Pdzk1ip1	0.0%	0.4%	0.4	2359
Nab2	0.0%	0.4%	0.4	2360
LOC103690979	0.0%	0.4%	0.4	2361
LOC108351433	0.0%	0.4%	0.4	2362
Zfp213	0.0%	0.4%	0.4	2363
Abr	0.0%	0.4%	0.4	2364
Unc93a	0.0%	0.4%	0.4	2365
Atp2b4	0.0%	0.4%	0.4	2366
Nlrp1a	0.0%	0.4%	0.4	2367
LOC100362819	0.0%	0.4%	0.4	2368
Atf2	0.0%	0.4%	0.4	2369
Cd5l	0.0%	0.4%	0.4	2370

Sipa1	0.0%	0.4%	0.4	2371
Arrb1	0.0%	0.4%	0.4	2372
Srcap	0.0%	0.4%	0.4	2373
LOC108348118	0.0%	0.4%	0.4	2374
Was	0.0%	0.4%	0.4	2375
N5	0.0%	0.4%	0.4	2376
Msn	0.0%	0.4%	0.4	2377
Tcf4	0.0%	0.4%	0.4	2378
LOC100910864	0.0%	0.4%	0.4	2379
LOC103692786	0.0%	0.4%	0.4	2380
Prpf31	0.0%	0.4%	0.4	2381
Mns1	0.0%	0.4%	0.4	2382
LOC103693122	0.0%	0.4%	0.4	2383
LOC108351135	0.0%	0.4%	0.4	2384
Dok2	0.0%	0.4%	0.4	2385
Slx4ip	0.0%	0.4%	0.4	2386
Nckap1l	0.0%	0.4%	0.4	2387
Porcn	0.0%	0.4%	0.4	2388
Cd27	0.0%	0.4%	0.4	2389
Adora2a	0.0%	0.4%	0.4	2390
Hba-a1	0.0%	0.4%	0.4	2391
LOC102547830	0.0%	0.4%	0.4	2392
Spatc1l	0.0%	0.4%	0.4	2393
Adgrl4	0.0%	0.4%	0.4	2394
RGD1359634	0.0%	0.4%	0.4	2395
Lemd3	0.0%	0.4%	0.4	2396
LOC102548586	0.0%	0.4%	0.4	2397
Lpcat1	0.0%	0.4%	0.4	2398
LOC102548350	0.0%	0.4%	0.4	2399
LOC102556139	0.0%	0.4%	0.4	2400
LOC108348267	0.0%	0.4%	0.4	2401
RGD1565222	0.0%	0.4%	0.4	2402
Fhit	0.0%	0.4%	0.4	2403
LOC102554306	0.0%	0.4%	0.4	2404
Adam23	0.0%	0.4%	0.4	2405
Timp2	0.0%	0.4%	0.4	2406
Aldh1a7	0.0%	0.4%	0.4	2407
LOC102547807	0.0%	0.4%	0.4	2408
Ccdc191	0.0%	0.4%	0.4	2409
LOC108349381	0.0%	0.4%	0.4	2410
Rab31	0.0%	0.4%	0.4	2411
Slco5a1	0.0%	0.4%	0.4	2412
Clec2d2	0.0%	0.4%	0.4	2413
Lrrc61	0.0%	0.4%	0.4	2414
Tor4a	0.0%	0.4%	0.4	2415
Cxcr3	0.0%	0.4%	0.4	2416
Dvl2	0.0%	0.4%	0.4	2417
Trim59	0.0%	0.4%	0.4	2418
Mrpl49	0.0%	0.4%	0.4	2419
Adam9	0.0%	0.4%	0.4	2420
LOC100911038	0.0%	0.4%	0.4	2421
LOC102547400	0.0%	0.4%	0.4	2422
Inhbe	0.0%	0.4%	0.4	2423
Klf7	0.0%	0.4%	0.4	2424
Robo1	0.0%	0.4%	0.4	2425
LOC102554758	0.0%	0.4%	0.4	2426
Cplx3	0.0%	0.4%	0.4	2427
Hck	0.0%	0.4%	0.4	2428
Pptc7	0.0%	0.4%	0.4	2429
LOC684773	0.0%	0.4%	0.4	2430
LOC102549675	0.0%	0.4%	0.4	2431
Pax3	0.0%	0.4%	0.4	2432
LOC100365047	0.0%	0.4%	0.4	2433
Ctgf	0.0%	0.4%	0.4	2434
Ckap2	0.0%	0.4%	0.4	2435
Kif20a	0.0%	0.4%	0.4	2436

Abca9	0.0%	0.4%	0.4	2437
Ly6i	0.0%	0.4%	0.4	2438
Apbb1ip	0.0%	0.4%	0.4	2439
LOC108348276	0.0%	0.4%	0.4	2440
Cyp4b1	0.0%	0.4%	0.4	2441
LOC108348173	0.0%	0.4%	0.4	2442
Tead2	0.0%	0.4%	0.4	2443
Meltf	0.0%	0.4%	0.4	2444
LOC102548772	0.0%	0.4%	0.4	2445
LOC303566	0.0%	0.4%	0.4	2446
LOC108348215	0.0%	0.4%	0.4	2447
Ncapd3	0.0%	0.4%	0.4	2448
RGD1306227	0.0%	0.4%	0.4	2449
LOC100912658	0.0%	0.4%	0.4	2450
Pkib	0.0%	0.4%	0.4	2451
Cd80	0.0%	0.4%	0.4	2452
Trim24	0.0%	0.4%	0.4	2453
Tshz2	0.0%	0.4%	0.4	2454
Ctse	0.0%	0.4%	0.4	2455
Adamts17	0.0%	0.4%	0.4	2456
Ihh	0.0%	0.4%	0.4	2457
LOC102557179	0.0%	0.4%	0.4	2458
LOC102557472	0.0%	0.4%	0.4	2459
RT1-DMb	3.9%	4.3%	0.4	2460
Cecr5	3.1%	3.5%	0.4	2461
Usp32	3.1%	3.5%	0.4	2462
Zmat5	2.7%	3.1%	0.4	2463
Spryd4	2.7%	3.1%	0.4	2464
Dph3	14.9%	15.3%	0.4	2465
RGD1561671	2.4%	2.7%	0.4	2466
Lrch4	2.4%	2.7%	0.4	2467
Rhof	2.4%	2.7%	0.4	2468
Vps53	2.4%	2.7%	0.4	2469
Smim1	2.0%	2.3%	0.4	2470
Rasgef1b	2.0%	2.3%	0.4	2471
LOC108352357	2.0%	2.3%	0.4	2472
Trappc8	2.0%	2.3%	0.4	2473
Trim41	1.6%	1.9%	0.3	2474
Thap2	1.6%	1.9%	0.3	2475
Mical2	1.6%	1.9%	0.3	2476
Pxylp1	1.6%	1.9%	0.3	2477
LOC102556709	1.6%	1.9%	0.3	2478
Arl5b	76.5%	76.8%	0.3	2479
LOC102554538	43.1%	43.5%	0.3	2480
LOC102548522	1.2%	1.5%	0.3	2481
RGD1561796	1.2%	1.5%	0.3	2482
Phf1	1.2%	1.5%	0.3	2483
Wwp2	1.2%	1.5%	0.3	2484
LOC688064	1.2%	1.5%	0.3	2485
Sema4a	1.2%	1.5%	0.3	2486
LOC683674	1.2%	1.5%	0.3	2487
Lin52	1.2%	1.5%	0.3	2488
Ablim1	1.2%	1.5%	0.3	2489
Hdac3	1.2%	1.5%	0.3	2490
Popdc2	1.2%	1.5%	0.3	2491
Helz	0.8%	1.1%	0.3	2492
LOC103694875	0.8%	1.1%	0.3	2493
Lcn2	0.8%	1.1%	0.3	2494
Ccdc34	0.8%	1.1%	0.3	2495
LOC102548056	0.8%	1.1%	0.3	2496
Gfod1	0.8%	1.1%	0.3	2497
Lmbrd2	0.8%	1.1%	0.3	2498
Fkbp15	0.8%	1.1%	0.3	2499
LOC102555377	0.8%	1.1%	0.3	2500
Socs1	0.8%	1.1%	0.3	2501
Ppp1r13b	0.8%	1.1%	0.3	2502

Hyal3	0.8%	1.1%	0.3	2503
Ptpn14	0.8%	1.1%	0.3	2504
Cd320	0.8%	1.1%	0.3	2505
Ralgps2	0.8%	1.1%	0.3	2506
LOC108353172	0.8%	1.1%	0.3	2507
Iffo1	0.8%	1.1%	0.3	2508
LOC100911993	0.8%	1.1%	0.3	2509
Zfp523	0.8%	1.1%	0.3	2510
Suv39h1	0.8%	1.1%	0.3	2511
Cblc	0.8%	1.1%	0.3	2512
Pot1	0.8%	1.1%	0.3	2513
Hdac1	4.7%	5.0%	0.3	2514
Ccdc66	0.4%	0.7%	0.3	2515
Cd68	0.4%	0.7%	0.3	2516
LOC688286	0.4%	0.7%	0.3	2517
LOC102548231	0.4%	0.7%	0.3	2518
LOC102550011	0.4%	0.7%	0.3	2519
LOC102550314	0.4%	0.7%	0.3	2520
Mk1	0.4%	0.7%	0.3	2521
Taok1	0.4%	0.7%	0.3	2522
Fopnl	0.4%	0.7%	0.3	2523
Bex3	0.4%	0.7%	0.3	2524
Prc1	0.4%	0.7%	0.3	2525
Nrp2	0.4%	0.7%	0.3	2526
LOC108349997	0.4%	0.7%	0.3	2527
Sspn	0.4%	0.7%	0.3	2528
Zkscan1	0.4%	0.7%	0.3	2529
Slc25a26	0.4%	0.7%	0.3	2530
Ggt1	0.4%	0.7%	0.3	2531
Dnmt1	0.4%	0.7%	0.3	2532
LOC100912566	0.4%	0.7%	0.3	2533
Hinfp	0.4%	0.7%	0.3	2534
Synrg	0.4%	0.7%	0.3	2535
RGD1564247	0.4%	0.7%	0.3	2536
Pcnx4	0.4%	0.7%	0.3	2537
Gabbr1	0.4%	0.7%	0.3	2538
Rcn2	0.4%	0.7%	0.3	2539
LOC102547698	0.4%	0.7%	0.3	2540
Map2k4	0.4%	0.7%	0.3	2541
Cdkn3	0.4%	0.7%	0.3	2542
Cchcr1	0.4%	0.7%	0.3	2543
Gfm2	0.4%	0.7%	0.3	2544
LOC100910957	0.4%	0.7%	0.3	2545
Izumo4	0.4%	0.7%	0.3	2546
Mylip	0.4%	0.7%	0.3	2547
Terf2	0.4%	0.7%	0.3	2548
Znrf3	0.4%	0.7%	0.3	2549
Exoc6b	0.4%	0.7%	0.3	2550
RGD1564450	0.4%	0.7%	0.3	2551
Tpx2	0.4%	0.7%	0.3	2552
Rab7b	0.4%	0.7%	0.3	2553
LOC102554711	0.4%	0.7%	0.3	2554
Nhej1	0.4%	0.7%	0.3	2555
Ccdc181	0.4%	0.7%	0.3	2556
Rhbg	0.4%	0.7%	0.3	2557
LOC501038	0.4%	0.7%	0.3	2558
Cenph	0.4%	0.7%	0.3	2559
Poc5	0.4%	0.7%	0.3	2560
Pcdhgb7	0.4%	0.7%	0.3	2561
LOC685025	0.4%	0.7%	0.3	2562
Tmtc2	0.4%	0.7%	0.3	2563
LOC102552880	0.4%	0.7%	0.3	2564
Fen1	0.4%	0.7%	0.3	2565
Fubp3	0.4%	0.7%	0.3	2566
Vkorc1l1	4.3%	4.6%	0.3	2567
LOC102555762	0.0%	0.3%	0.3	2568

Ninl	0.0%	0.3%	0.3	2569
LOC100363782	0.0%	0.3%	0.3	2570
LOC108353656	0.0%	0.3%	0.3	2571
LOC100361313	0.0%	0.3%	0.3	2572
Gzmf	0.0%	0.3%	0.3	2573
Gpr182	0.0%	0.3%	0.3	2574
Tmx2	0.0%	0.3%	0.3	2575
Plekho1	0.0%	0.3%	0.3	2576
Ttll5	0.0%	0.3%	0.3	2577
Mfsd2b	0.0%	0.3%	0.3	2578
Gpr174	0.0%	0.3%	0.3	2579
LOC100912124	0.0%	0.3%	0.3	2580
Arhgap15	0.0%	0.3%	0.3	2581
Pdpr	0.0%	0.3%	0.3	2582
RGD1561870	0.0%	0.3%	0.3	2583
LOC102547920	0.0%	0.3%	0.3	2584
LOC500273	0.0%	0.3%	0.3	2585
Apobr	0.0%	0.3%	0.3	2586
Arhgap23	0.0%	0.3%	0.3	2587
LOC102551133	0.0%	0.3%	0.3	2588
Agap2	0.0%	0.3%	0.3	2589
RGD1565410	0.0%	0.3%	0.3	2590
Egf	0.0%	0.3%	0.3	2591
Mthfd1l	0.0%	0.3%	0.3	2592
LOC108353670	0.0%	0.3%	0.3	2593
Renbp	0.0%	0.3%	0.3	2594
Cabyr	0.0%	0.3%	0.3	2595
LOC102555623	0.0%	0.3%	0.3	2596
Prkcb	0.0%	0.3%	0.3	2597
Cxcr5	0.0%	0.3%	0.3	2598
Jag1	0.0%	0.3%	0.3	2599
LOC102556834	0.0%	0.3%	0.3	2600
LOC688649	0.0%	0.3%	0.3	2601
LOC100909470	0.0%	0.3%	0.3	2602
Kcnip4	0.0%	0.3%	0.3	2603
LOC102549089	0.0%	0.3%	0.3	2604
Tnfrsf4	0.0%	0.3%	0.3	2605
RGD1566138	0.0%	0.3%	0.3	2606
Gnat1	0.0%	0.3%	0.3	2607
Ak5	0.0%	0.3%	0.3	2608
Uprt	0.0%	0.3%	0.3	2609
LOC108348741	0.0%	0.3%	0.3	2610
Flrt3	0.0%	0.3%	0.3	2611
LOC108350980	0.0%	0.3%	0.3	2612
RT1-T24-1	0.0%	0.3%	0.3	2613
Siglec5	0.0%	0.3%	0.3	2614
Ptgs1	0.0%	0.3%	0.3	2615
LOC100909857	0.0%	0.3%	0.3	2616
LOC100911426	0.0%	0.3%	0.3	2617
LOC681300	0.0%	0.3%	0.3	2618
Clec4a1	0.0%	0.3%	0.3	2619
Prdm1	0.0%	0.3%	0.3	2620
LOC103690166	0.0%	0.3%	0.3	2621
LOC102548541	0.0%	0.3%	0.3	2622
Arhgap30	0.0%	0.3%	0.3	2623
LOC102548478	0.0%	0.3%	0.3	2624
Tnni1	0.0%	0.3%	0.3	2625
Chn2	0.0%	0.3%	0.3	2626
RGD1310852	0.0%	0.3%	0.3	2627
LOC685267	0.0%	0.3%	0.3	2628
Tlr13	0.0%	0.3%	0.3	2629
Tacc1	0.0%	0.3%	0.3	2630
LOC681193	0.0%	0.3%	0.3	2631
Spag5	0.0%	0.3%	0.3	2632
CA...	0.0%	0.3%	0.3	2633
Styxl2	0.0%	0.3%	0.3	2634

Galnt12	0.0%	0.3%	0.3	2635
Sass6	0.0%	0.3%	0.3	2636
LOC102553369	0.0%	0.3%	0.3	2637
Mtcl1	0.0%	0.3%	0.3	2638
Plk4	0.0%	0.3%	0.3	2639
Ggt5	0.0%	0.3%	0.3	2640
LOC100365212	0.0%	0.3%	0.3	2641
Mapkbp1	0.0%	0.3%	0.3	2642
Ncr3	0.0%	0.3%	0.3	2643
Pdlim2	0.0%	0.3%	0.3	2644
LOC108351556	0.0%	0.3%	0.3	2645
LOC108348270	0.0%	0.3%	0.3	2646
C1qtnf3	0.0%	0.3%	0.3	2647
Setbp1	0.0%	0.3%	0.3	2648
LOC102552779	0.0%	0.3%	0.3	2649
Arhgap26	0.0%	0.3%	0.3	2650
Mef2b	0.0%	0.3%	0.3	2651
Clec4f	0.0%	0.3%	0.3	2652
Mid2	0.0%	0.3%	0.3	2653
Ttc12	0.0%	0.3%	0.3	2654
Tubd1	0.0%	0.3%	0.3	2655
Cd72	0.0%	0.3%	0.3	2656
Thbd	0.0%	0.3%	0.3	2657
LOC686151	0.0%	0.3%	0.3	2658
LOC100912677	0.0%	0.3%	0.3	2659
Hip1	0.0%	0.3%	0.3	2660
LOC108353224	0.0%	0.3%	0.3	2661
Tubb4a	0.0%	0.3%	0.3	2662
LOC100912034	0.0%	0.3%	0.3	2663
Lyst	0.0%	0.3%	0.3	2664
Serpinh1	0.0%	0.3%	0.3	2665
LOC102547042	0.0%	0.3%	0.3	2666
LOC102551693	0.0%	0.3%	0.3	2667
Hdac11	0.0%	0.3%	0.3	2668
Itm2a	0.0%	0.3%	0.3	2669
RGD1564433	0.0%	0.3%	0.3	2670
LOC103692054	0.0%	0.3%	0.3	2671
Ear1	0.0%	0.3%	0.3	2672
Rab19	0.0%	0.3%	0.3	2673
Ints6l	0.0%	0.3%	0.3	2674
Ccl7	0.0%	0.3%	0.3	2675
LOC102549173	0.0%	0.3%	0.3	2676
Edaradd	0.0%	0.3%	0.3	2677
RGD1561730	0.0%	0.3%	0.3	2678
Try4	0.0%	0.3%	0.3	2679
Hira	0.0%	0.3%	0.3	2680
Vcpkmt	0.0%	0.3%	0.3	2681
Itpka	0.0%	0.3%	0.3	2682
Tinagl1	0.0%	0.3%	0.3	2683
Notch1	0.0%	0.3%	0.3	2684
Cmtm3	0.0%	0.3%	0.3	2685
Cpne2	0.0%	0.3%	0.3	2686
Fmo6	0.0%	0.3%	0.3	2687
Ncf2	0.0%	0.3%	0.3	2688
Mum1	0.0%	0.3%	0.3	2689
Mcm6	0.0%	0.3%	0.3	2690
LOC108348110	0.0%	0.3%	0.3	2691
Fstl1	0.0%	0.3%	0.3	2692
Nrg4	0.0%	0.3%	0.3	2693
LOC102548486	0.0%	0.3%	0.3	2694
Zc3h12d	0.0%	0.3%	0.3	2695
Rad51c	0.0%	0.3%	0.3	2696
Zfp949	0.0%	0.3%	0.3	2697
Gng11	0.0%	0.3%	0.3	2698
Agpat1	0.0%	0.3%	0.3	2699
Uap1l2	0.0%	0.3%	0.3	2700

LOC102548308	0.0%	0.3%	0.3	2701
LOC102553405	0.0%	0.3%	0.3	2702
LOC108349064	0.0%	0.3%	0.3	2703
Naif1	0.0%	0.3%	0.3	2704
LOC102553607	0.0%	0.3%	0.3	2705
LOC102547023	0.0%	0.3%	0.3	2706
Ccdc171	0.0%	0.3%	0.3	2707
Acta2	0.0%	0.3%	0.3	2708
LOC100911065	0.0%	0.3%	0.3	2709
Cdc25b	0.0%	0.3%	0.3	2710
Mef2c	0.0%	0.3%	0.3	2711
Agrp	0.0%	0.3%	0.3	2712
Gimap8	0.0%	0.3%	0.3	2713
LOC108350822	0.0%	0.3%	0.3	2714
RGD1560723	0.0%	0.3%	0.3	2715
Gvinp1	0.0%	0.3%	0.3	2716
LOC102550985	0.0%	0.3%	0.3	2717
Paqr8	0.0%	0.3%	0.3	2718
Clnk	0.0%	0.3%	0.3	2719
Zfp167	0.0%	0.3%	0.3	2720
Mus81	0.0%	0.3%	0.3	2721
RT1-M6-2	0.0%	0.3%	0.3	2722
Car12	0.0%	0.3%	0.3	2723
Cd28	0.0%	0.3%	0.3	2724
Spred3	0.0%	0.3%	0.3	2725
Cdk5rap2	0.0%	0.3%	0.3	2726
Svop	0.0%	0.3%	0.3	2727
Bach2	0.0%	0.3%	0.3	2728
Tmem167b	0.0%	0.3%	0.3	2729
LOC102547322	0.0%	0.3%	0.3	2730
Rassf2	0.0%	0.3%	0.3	2731
LOC501406	0.0%	0.3%	0.3	2732
Klrb1a	0.0%	0.3%	0.3	2733
LOC102552138	0.0%	0.3%	0.3	2734
Rab3a	0.0%	0.3%	0.3	2735
LOC100910669	0.0%	0.3%	0.3	2736
LOC102548526	0.0%	0.3%	0.3	2737
Fhl3	0.0%	0.3%	0.3	2738
Ncapg2	0.0%	0.3%	0.3	2739
LOC688090	0.0%	0.3%	0.3	2740
Baz2a	0.0%	0.3%	0.3	2741
Adap1	0.0%	0.3%	0.3	2742
Cd38	0.0%	0.3%	0.3	2743
Eva1b	0.0%	0.3%	0.3	2744
Spns2	0.0%	0.3%	0.3	2745
Gpr37l1	0.0%	0.3%	0.3	2746
LOC103691849	0.0%	0.3%	0.3	2747
Smim10l1	0.0%	0.3%	0.3	2748
Lfng	0.0%	0.3%	0.3	2749
Pcdh7	0.0%	0.3%	0.3	2750
LOC102556971	0.0%	0.3%	0.3	2751
LOC100911498	0.0%	0.3%	0.3	2752
Mmp9	0.0%	0.3%	0.3	2753
Entpd1	0.0%	0.3%	0.3	2754
Ddx60	0.0%	0.3%	0.3	2755
LOC108349901	0.0%	0.3%	0.3	2756
Evi2b	0.0%	0.3%	0.3	2757
Map2	0.0%	0.3%	0.3	2758
Poc1a	0.0%	0.3%	0.3	2759
Colq	0.0%	0.3%	0.3	2760
LOC691670	0.0%	0.3%	0.3	2761
Il16	0.0%	0.3%	0.3	2762
Lrmp	0.0%	0.3%	0.3	2763
Hacd4	0.0%	0.3%	0.3	2764
LOC100909476	0.0%	0.3%	0.3	2765
Cyp2s1	0.0%	0.3%	0.3	2766

LOC103693237	0.0%	0.3%	0.3	2767
Lmcd1	0.0%	0.3%	0.3	2768
LOC102551311	0.0%	0.3%	0.3	2769
LOC100910833	0.0%	0.3%	0.3	2770
Ccdc77	0.0%	0.3%	0.3	2771
Hist1h2ah	0.0%	0.3%	0.3	2772
Suv39h2	0.0%	0.3%	0.3	2773
Dact1	0.0%	0.3%	0.3	2774
Maz	0.0%	0.3%	0.3	2775
Elk3	0.0%	0.3%	0.3	2776
Tmem163	0.0%	0.3%	0.3	2777
Pkhd1	0.0%	0.3%	0.3	2778
Slc16a3	0.0%	0.3%	0.3	2779
Tigar	0.0%	0.3%	0.3	2780
Col4a2	0.0%	0.3%	0.3	2781
Stk24	0.0%	0.3%	0.3	2782
Ptpn18	0.0%	0.3%	0.3	2783
Cd200r1	0.0%	0.3%	0.3	2784
E4f1	0.0%	0.3%	0.3	2785
Csf1r	0.0%	0.3%	0.3	2786
LOC102557535	0.0%	0.3%	0.3	2787
Dlgap5	0.0%	0.3%	0.3	2788
LOC102554532	0.0%	0.3%	0.3	2789
LOC108348412	0.0%	0.3%	0.3	2790
Jam2	0.0%	0.3%	0.3	2791
LOC103693502	0.0%	0.3%	0.3	2792
LOC102557590	0.0%	0.3%	0.3	2793
Map3k10	0.0%	0.3%	0.3	2794
Heyl	0.0%	0.3%	0.3	2795
LOC103690119	0.0%	0.3%	0.3	2796
Cnep1r1	0.0%	0.3%	0.3	2797
Samhd1	0.0%	0.3%	0.3	2798
Rasa4	0.0%	0.3%	0.3	2799
LOC108353809	0.0%	0.3%	0.3	2800
LOC103690014	0.0%	0.3%	0.3	2801
Ackr4	0.0%	0.3%	0.3	2802
LOC100911027	0.0%	0.3%	0.3	2803
Itga2b	0.0%	0.3%	0.3	2804
Apcdd1l	0.0%	0.3%	0.3	2805
Tdh	0.0%	0.3%	0.3	2806
LOC103691848	0.0%	0.3%	0.3	2807
Ly49si1	0.0%	0.3%	0.3	2808
Acap1	0.0%	0.3%	0.3	2809
LOC103691190	0.0%	0.3%	0.3	2810
Tnfaip8l2	0.0%	0.3%	0.3	2811
Ncaph	0.0%	0.3%	0.3	2812
Ap3m2	0.0%	0.3%	0.3	2813
LOC102548286	3.9%	4.2%	0.3	2814
LOC100362987	7.8%	8.2%	0.3	2815
Cd300lf	3.5%	3.8%	0.3	2816
Srek1ip1	3.1%	3.4%	0.3	2817
Mbd6	2.4%	2.6%	0.3	2818
Cyp2c7	89.8%	90.1%	0.3	2819
LOC687679	6.3%	6.6%	0.3	2820
Plcg1	2.0%	2.2%	0.3	2821
Ints6	2.0%	2.2%	0.3	2822
Gsr	2.0%	2.2%	0.3	2823
Rce1	1.6%	1.8%	0.3	2824
Eme2	1.6%	1.8%	0.3	2825
LOC108350588	1.6%	1.8%	0.3	2826
Plekha3	1.6%	1.8%	0.3	2827
LOC498592	1.6%	1.8%	0.3	2828
C3ar1	1.6%	1.8%	0.3	2829
Paip2b	1.6%	1.8%	0.3	2830
Slc9a3r1	1.6%	1.8%	0.3	2831
Cebpb	22.4%	22.6%	0.3	2832

LOC103693015	5.5%	5.8%	0.3	2833
LOC103691767	1.2%	1.4%	0.3	2834
Creb3l1	1.2%	1.4%	0.3	2835
Lsm14b	1.2%	1.4%	0.3	2836
NEWGENE_1562258	1.2%	1.4%	0.3	2837
Sec14l4	1.2%	1.4%	0.3	2838
Fam117b	1.2%	1.4%	0.3	2839
Ergic1	9.4%	9.7%	0.3	2840
Cenpa	0.8%	1.0%	0.3	2841
LOC681090	0.8%	1.0%	0.3	2842
Uck2	0.8%	1.0%	0.3	2843
Bach1	0.8%	1.0%	0.3	2844
Stk35	0.8%	1.0%	0.3	2845
Zcchc4	0.8%	1.0%	0.3	2846
Slc43a2	0.8%	1.0%	0.3	2847
Ldlr	0.8%	1.0%	0.3	2848
Ccdc28b	0.8%	1.0%	0.3	2849
Engase	0.8%	1.0%	0.3	2850
Slc38a7	0.8%	1.0%	0.3	2851
LOC100912282	0.8%	1.0%	0.3	2852
Smchd1	0.8%	1.0%	0.3	2853
Clcn6	0.8%	1.0%	0.3	2854
Rnf40	0.8%	1.0%	0.3	2855
G6pc3	0.8%	1.0%	0.3	2856
Dhx58	0.8%	1.0%	0.3	2857
Dbp	0.8%	1.0%	0.3	2858
Epha3	0.8%	1.0%	0.3	2859
LOC102554859	0.8%	1.0%	0.3	2860
Gnptab	0.8%	1.0%	0.3	2861
Mad2l1	0.8%	1.0%	0.3	2862
LOC100911727	0.8%	1.0%	0.3	2863
Nr3c2	0.4%	0.6%	0.2	2864
Dlgap1	0.4%	0.6%	0.2	2865
LOC100364016	0.4%	0.6%	0.2	2866
Enkd1	0.4%	0.6%	0.2	2867
RGD1304884	0.4%	0.6%	0.2	2868
Lzts3	0.4%	0.6%	0.2	2869
LOC688839	0.4%	0.6%	0.2	2870
Ptprs	0.4%	0.6%	0.2	2871
Aak1	0.4%	0.6%	0.2	2872
Rgma	0.4%	0.6%	0.2	2873
Wars2	0.4%	0.6%	0.2	2874
Hist1h2af	0.4%	0.6%	0.2	2875
LOC102551365	0.4%	0.6%	0.2	2876
Tspan18	0.4%	0.6%	0.2	2877
Sdccag8	0.4%	0.6%	0.2	2878
LOC103690340	0.4%	0.6%	0.2	2879
Trdmt1	0.4%	0.6%	0.2	2880
Ercc4	0.4%	0.6%	0.2	2881
Cyp4f18	0.4%	0.6%	0.2	2882
LOC108351200	0.4%	0.6%	0.2	2883
Oas1g	0.4%	0.6%	0.2	2884
Pigw	0.4%	0.6%	0.2	2885
LOC102554658	0.4%	0.6%	0.2	2886
Dido1	0.4%	0.6%	0.2	2887
LOC102553072	0.4%	0.6%	0.2	2888
Dtwd1	0.4%	0.6%	0.2	2889
Kpna6	0.4%	0.6%	0.2	2890
Fbxl4	0.4%	0.6%	0.2	2891
Ralgds	0.4%	0.6%	0.2	2892
LOC103694346	0.4%	0.6%	0.2	2893
Fzd1	0.4%	0.6%	0.2	2894
LOC100360574	0.4%	0.6%	0.2	2895
LOC103692995	0.4%	0.6%	0.2	2896
Micall1	0.4%	0.6%	0.2	2897
Zfp420	4.3%	4.6%	0.2	2898

Sept5	0.0%	0.2%	0.2	2899
Bcorl1	0.0%	0.2%	0.2	2900
LOC108348284	0.0%	0.2%	0.2	2901
LOC499843	0.0%	0.2%	0.2	2902
Card9	0.0%	0.2%	0.2	2903
Cd163	0.0%	0.2%	0.2	2904
Filip1l	0.0%	0.2%	0.2	2905
LOC108349348	0.0%	0.2%	0.2	2906
LOC108348988	0.0%	0.2%	0.2	2907
Nf1	0.0%	0.2%	0.2	2908
Pcdhga11	0.0%	0.2%	0.2	2909
Plac9	0.0%	0.2%	0.2	2910
LOC102553602	0.0%	0.2%	0.2	2911
Kif12	0.0%	0.2%	0.2	2912
Tesc	0.0%	0.2%	0.2	2913
Traf5	0.0%	0.2%	0.2	2914
Prdm9	0.0%	0.2%	0.2	2915
Cdkn2d	0.0%	0.2%	0.2	2916
LOC108350921	0.0%	0.2%	0.2	2917
LOC685881	0.0%	0.2%	0.2	2918
Glipr1l2	0.0%	0.2%	0.2	2919
LOC103694155	0.0%	0.2%	0.2	2920
Nek2	0.0%	0.2%	0.2	2921
Pik3r6	0.0%	0.2%	0.2	2922
Il18r1	0.0%	0.2%	0.2	2923
Myo1f	0.0%	0.2%	0.2	2924
Chst2	0.0%	0.2%	0.2	2925
Alox5ap	0.0%	0.2%	0.2	2926
Marco	0.0%	0.2%	0.2	2927
Cav1	0.0%	0.2%	0.2	2928
Rrm2b	0.0%	0.2%	0.2	2929
LOC103694708	0.0%	0.2%	0.2	2930
Sectm1a	0.0%	0.2%	0.2	2931
Add3	0.0%	0.2%	0.2	2932
LOC679342	0.0%	0.2%	0.2	2933
Zfp39	0.0%	0.2%	0.2	2934
LOC100360754	0.0%	0.2%	0.2	2935
LOC103694167	0.0%	0.2%	0.2	2936
RGD1566325	0.0%	0.2%	0.2	2937
Eml2	0.0%	0.2%	0.2	2938
LOC108349099	0.0%	0.2%	0.2	2939
Akap17b	0.0%	0.2%	0.2	2940
LOC102556873	0.0%	0.2%	0.2	2941
Scarf1	0.0%	0.2%	0.2	2942
Ly49s5	0.0%	0.2%	0.2	2943
Arhgef15	0.0%	0.2%	0.2	2944
Plet1	0.0%	0.2%	0.2	2945
Gpr132	0.0%	0.2%	0.2	2946
Faap24	0.0%	0.2%	0.2	2947
Tppp3	0.0%	0.2%	0.2	2948
LOC100910636	0.0%	0.2%	0.2	2949
Galnt6	0.0%	0.2%	0.2	2950
Jph4	0.0%	0.2%	0.2	2951
Icam2	0.0%	0.2%	0.2	2952
Lrrc32	0.0%	0.2%	0.2	2953
Sfmbt2	0.0%	0.2%	0.2	2954
Ramp3	0.0%	0.2%	0.2	2955
Jdp2	0.0%	0.2%	0.2	2956
Smpdl3b	0.0%	0.2%	0.2	2957
LOC103695196	0.0%	0.2%	0.2	2958
Tmem154	0.0%	0.2%	0.2	2959
LOC287992	0.0%	0.2%	0.2	2960
Efnb2	0.0%	0.2%	0.2	2961
Prdm6	0.0%	0.2%	0.2	2962
Ska1	0.0%	0.2%	0.2	2963
LOC102550122	0.0%	0.2%	0.2	2964

Zfp324	0.0%	0.2%	0.2	2965
Prrt3	0.0%	0.2%	0.2	2966
Rn5s	0.0%	0.2%	0.2	2967
Cacna2d4	0.0%	0.2%	0.2	2968
Gpc1	0.0%	0.2%	0.2	2969
Rapsn	0.0%	0.2%	0.2	2970
LOC103691468	0.0%	0.2%	0.2	2971
Rasl2-9	0.0%	0.2%	0.2	2972
Tmem229b	0.0%	0.2%	0.2	2973
LOC102556943	0.0%	0.2%	0.2	2974
Map2k6	0.0%	0.2%	0.2	2975
Sh2d1a	0.0%	0.2%	0.2	2976
Palb2	0.0%	0.2%	0.2	2977
LOC103695103	0.0%	0.2%	0.2	2978
LOC100912181	0.0%	0.2%	0.2	2979
Parvb	0.0%	0.2%	0.2	2980
RGD1562161	0.0%	0.2%	0.2	2981
Slamf9	0.0%	0.2%	0.2	2982
Mcoln2	0.0%	0.2%	0.2	2983
Nbeal2	0.0%	0.2%	0.2	2984
LOC102555660	0.0%	0.2%	0.2	2985
Fam189b	0.0%	0.2%	0.2	2986
Mllt1	0.0%	0.2%	0.2	2987
Try5	0.0%	0.2%	0.2	2988
Hist1h2ail1	0.0%	0.2%	0.2	2989
Mcm5	0.0%	0.2%	0.2	2990
LOC102549471	0.0%	0.2%	0.2	2991
Fxyd3	0.0%	0.2%	0.2	2992
Skida1	0.0%	0.2%	0.2	2993
Hk3	0.0%	0.2%	0.2	2994
Krt19	0.0%	0.2%	0.2	2995
Cdkn2c	0.0%	0.2%	0.2	2996
LOC102548014	0.0%	0.2%	0.2	2997
Hpse2	0.0%	0.2%	0.2	2998
Nrp1	0.0%	0.2%	0.2	2999
Colec11	0.0%	0.2%	0.2	3000
Pag1	0.0%	0.2%	0.2	3001
Vof16	0.0%	0.2%	0.2	3002
LOC100910060	0.0%	0.2%	0.2	3003
LOC100911867	0.0%	0.2%	0.2	3004
Ubxn7	0.0%	0.2%	0.2	3005
LOC103689948	0.0%	0.2%	0.2	3006
RGD1561147	0.0%	0.2%	0.2	3007
Tubgcp5	0.0%	0.2%	0.2	3008
Calml4	0.0%	0.2%	0.2	3009
LOC102556057	0.0%	0.2%	0.2	3010
Fhod1	0.0%	0.2%	0.2	3011
Cyp4f40	0.0%	0.2%	0.2	3012
Nap1l3	0.0%	0.2%	0.2	3013
Xkrx	0.0%	0.2%	0.2	3014
Fam196b	0.0%	0.2%	0.2	3015
Tmsbl1	0.0%	0.2%	0.2	3016
LOC108351252	0.0%	0.2%	0.2	3017
Cyp4f37	0.0%	0.2%	0.2	3018
Nr2c2	0.0%	0.2%	0.2	3019
Agpat2	0.0%	0.2%	0.2	3020
Cntn2	0.0%	0.2%	0.2	3021
Gstm6	0.0%	0.2%	0.2	3022
Mvb12b	0.0%	0.2%	0.2	3023
Anxa13	0.0%	0.2%	0.2	3024
Arhgap45	0.0%	0.2%	0.2	3025
Gna14	0.0%	0.2%	0.2	3026
LOC102553861	0.0%	0.2%	0.2	3027
LOC691254	0.0%	0.2%	0.2	3028
Aspm	0.0%	0.2%	0.2	3029
Dcst2	0.0%	0.2%	0.2	3030

Lyl1	0.0%	0.2%	0.2	3031
LOC102547938	0.0%	0.2%	0.2	3032
Wdr92	0.0%	0.2%	0.2	3033
Gpr4	0.0%	0.2%	0.2	3034
Ddias	0.0%	0.2%	0.2	3035
Tmc6	0.0%	0.2%	0.2	3036
Meis2	0.0%	0.2%	0.2	3037
Wfs1	0.0%	0.2%	0.2	3038
Vsig4	0.0%	0.2%	0.2	3039
Otud3	0.0%	0.2%	0.2	3040
LOC102551014	0.0%	0.2%	0.2	3041
Cep128	0.0%	0.2%	0.2	3042
LOC108353419	0.0%	0.2%	0.2	3043
Sh2d7	0.0%	0.2%	0.2	3044
LOC108352841	0.0%	0.2%	0.2	3045
Gnaq	0.0%	0.2%	0.2	3046
Ercc4l1	0.0%	0.2%	0.2	3047
Cdkl2	0.0%	0.2%	0.2	3048
Rgcc	0.0%	0.2%	0.2	3049
LOC102556347	0.0%	0.2%	0.2	3050
LOC100360575	0.0%	0.2%	0.2	3051
Asb2	0.0%	0.2%	0.2	3052
LOC501033	0.0%	0.2%	0.2	3053
LOC102554679	0.0%	0.2%	0.2	3054
Sap25	0.0%	0.2%	0.2	3055
Rai2	0.0%	0.2%	0.2	3056
LOC102549948	0.0%	0.2%	0.2	3057
LOC100911837	0.0%	0.2%	0.2	3058
Macrod2	0.0%	0.2%	0.2	3059
LOC102546511	0.0%	0.2%	0.2	3060
Ly75	0.0%	0.2%	0.2	3061
Mbd5	0.0%	0.2%	0.2	3062
Olfm1	0.0%	0.2%	0.2	3063
Faxdc2	0.0%	0.2%	0.2	3064
LOC103690038	0.0%	0.2%	0.2	3065
LOC108353561	0.0%	0.2%	0.2	3066
LOC108349745	0.0%	0.2%	0.2	3067
Racgap1	0.0%	0.2%	0.2	3068
Kcnab2	0.0%	0.2%	0.2	3069
Ptpre	0.0%	0.2%	0.2	3070
Vnn3	0.0%	0.2%	0.2	3071
Klrc2	0.0%	0.2%	0.2	3072
Hivep3	0.0%	0.2%	0.2	3073
Ube2q2l	0.0%	0.2%	0.2	3074
Fkbp14	0.0%	0.2%	0.2	3075
Kank4	0.0%	0.2%	0.2	3076
Ccdc61	0.0%	0.2%	0.2	3077
LOC102554922	0.0%	0.2%	0.2	3078
Tube1	0.0%	0.2%	0.2	3079
Ehbp1l1	0.0%	0.2%	0.2	3080
Pnliprp2	0.0%	0.2%	0.2	3081
Klrc3	0.0%	0.2%	0.2	3082
Hist2h4	0.0%	0.2%	0.2	3083
Duoxa1	0.0%	0.2%	0.2	3084
LOC102551925	0.0%	0.2%	0.2	3085
Nanos1	0.0%	0.2%	0.2	3086
LOC102554020	0.0%	0.2%	0.2	3087
Plekhg1	0.0%	0.2%	0.2	3088
LOC100910720	0.0%	0.2%	0.2	3089
Cacng2	0.0%	0.2%	0.2	3090
LOC102547294	0.0%	0.2%	0.2	3091
Clec1b	0.0%	0.2%	0.2	3092
Nox1	0.0%	0.2%	0.2	3093
Sumo4	0.0%	0.2%	0.2	3094
LOC679782	0.0%	0.2%	0.2	3095
Atp2a3	0.0%	0.2%	0.2	3096

Fcnb	0.0%	0.2%	0.2	3097
Ugt2a1	0.0%	0.2%	0.2	3098
LOC103693240	0.0%	0.2%	0.2	3099
RGD1307461	0.0%	0.2%	0.2	3100
Trps1	0.0%	0.2%	0.2	3101
LOC102557192	0.0%	0.2%	0.2	3102
Thbs4	0.0%	0.2%	0.2	3103
LOC102551963	0.0%	0.2%	0.2	3104
LOC681658	0.0%	0.2%	0.2	3105
Zfp692	0.0%	0.2%	0.2	3106
LOC108353586	0.0%	0.2%	0.2	3107
Nphs1	0.0%	0.2%	0.2	3108
Itgad	0.0%	0.2%	0.2	3109
Mgat4a	0.0%	0.2%	0.2	3110
Sik2	0.0%	0.2%	0.2	3111
Nav1	0.0%	0.2%	0.2	3112
Cyp3a85-ps	0.0%	0.2%	0.2	3113
LOC102556573	0.0%	0.2%	0.2	3114
LOC100911779	0.0%	0.2%	0.2	3115
Hydin	0.0%	0.2%	0.2	3116
LOC102547890	0.0%	0.2%	0.2	3117
Pcdhga2	0.0%	0.2%	0.2	3118
LOC367277	0.0%	0.2%	0.2	3119
LOC100910286	0.0%	0.2%	0.2	3120
RGD1566251	0.0%	0.2%	0.2	3121
Ube2t	0.0%	0.2%	0.2	3122
RGD1563578	0.0%	0.2%	0.2	3123
Ccdc152	0.0%	0.2%	0.2	3124
Krt79	0.0%	0.2%	0.2	3125
Mmrn2	0.0%	0.2%	0.2	3126
Tmem268	0.0%	0.2%	0.2	3127
Slc38a11	0.0%	0.2%	0.2	3128
RGD1561143	0.0%	0.2%	0.2	3129
Ptch1	0.0%	0.2%	0.2	3130
Cryba4	0.0%	0.2%	0.2	3131
Dcp2	0.0%	0.2%	0.2	3132
Sipa1l3	0.0%	0.2%	0.2	3133
Clec1a	0.0%	0.2%	0.2	3134
Cdh13	0.0%	0.2%	0.2	3135
LOC102548245	0.0%	0.2%	0.2	3136
LOC687508	0.0%	0.2%	0.2	3137
Mmp7	0.0%	0.2%	0.2	3138
LOC108352343	0.0%	0.2%	0.2	3139
Nfxl1	0.0%	0.2%	0.2	3140
Prom1	0.0%	0.2%	0.2	3141
Kcnj2	0.0%	0.2%	0.2	3142
Tead4	0.0%	0.2%	0.2	3143
Fmn1	0.0%	0.2%	0.2	3144
Podnl1	0.0%	0.2%	0.2	3145
LOC108353599	0.0%	0.2%	0.2	3146
Gja1	0.0%	0.2%	0.2	3147
Pecam1	0.0%	0.2%	0.2	3148
Dip2b	0.0%	0.2%	0.2	3149
Mmp13	0.0%	0.2%	0.2	3150
Dck	0.0%	0.2%	0.2	3151
Zbp1	0.0%	0.2%	0.2	3152
Rfwd3	0.0%	0.2%	0.2	3153
LOC102550609	0.0%	0.2%	0.2	3154
RGD1565143	0.0%	0.2%	0.2	3155
Abcg4	0.0%	0.2%	0.2	3156
Fabp4	0.0%	0.2%	0.2	3157
Itpr3	0.0%	0.2%	0.2	3158
Slc25a43	0.0%	0.2%	0.2	3159
Cfap45	0.0%	0.2%	0.2	3160
Rttn	0.0%	0.2%	0.2	3161
Ramp2	0.0%	0.2%	0.2	3162

Mfsd4	0.0%	0.2%	0.2	3163
LOC100909860	0.0%	0.2%	0.2	3164
Foxp3	0.0%	0.2%	0.2	3165
Lama4	0.0%	0.2%	0.2	3166
Bub1	0.0%	0.2%	0.2	3167
RGD1310335	0.0%	0.2%	0.2	3168
LOC108348370	0.0%	0.2%	0.2	3169
LOC108351567	0.0%	0.2%	0.2	3170
Exph5	0.0%	0.2%	0.2	3171
Xkr4	0.0%	0.2%	0.2	3172
Gng13	0.0%	0.2%	0.2	3173
Ldlrad3	0.0%	0.2%	0.2	3174
LOC102552514	0.0%	0.2%	0.2	3175
LOC100911693	0.0%	0.2%	0.2	3176
Mir568	0.0%	0.2%	0.2	3177
Etfbkmt	0.0%	0.2%	0.2	3178
LOC102547448	0.0%	0.2%	0.2	3179
LOC102548141	0.0%	0.2%	0.2	3180
LOC103694924	0.0%	0.2%	0.2	3181
Il17rd	0.0%	0.2%	0.2	3182
LOC100911887	0.0%	0.2%	0.2	3183
Igf2	0.0%	0.2%	0.2	3184
LOC102548856	0.0%	0.2%	0.2	3185
LOC103690095	0.0%	0.2%	0.2	3186
Lst1	0.0%	0.2%	0.2	3187
Rab8b	0.0%	0.2%	0.2	3188
LOC100364304	0.0%	0.2%	0.2	3189
Stard8	0.0%	0.2%	0.2	3190
Prex2	0.0%	0.2%	0.2	3191
LOC102546968	0.0%	0.2%	0.2	3192
LOC108349154	0.0%	0.2%	0.2	3193
Cd300le	0.0%	0.2%	0.2	3194
Qpct	0.0%	0.2%	0.2	3195
Il12rb2	0.0%	0.2%	0.2	3196
Epdr1	0.0%	0.2%	0.2	3197
LOC102556096	0.0%	0.2%	0.2	3198
Colec10	0.0%	0.2%	0.2	3199
LOC100910604	0.0%	0.2%	0.2	3200
Tmem102	0.0%	0.2%	0.2	3201
LOC100365363	0.0%	0.2%	0.2	3202
RGD1562146	0.0%	0.2%	0.2	3203
LOC102551574	0.0%	0.2%	0.2	3204
Tln2	0.0%	0.2%	0.2	3205
Lmo2	0.0%	0.2%	0.2	3206
Reln	0.0%	0.2%	0.2	3207
LOC100912045	0.0%	0.2%	0.2	3208
Arap2	0.0%	0.2%	0.2	3209
Susd2	0.0%	0.2%	0.2	3210
Gins1	0.0%	0.2%	0.2	3211
LOC103692961	0.0%	0.2%	0.2	3212
Pcdh1	0.0%	0.2%	0.2	3213
Tspan32	0.0%	0.2%	0.2	3214
Ankrd42	0.0%	0.2%	0.2	3215
Hpse	0.0%	0.2%	0.2	3216
LOC100909836	0.0%	0.2%	0.2	3217
LOC108348108	0.0%	0.2%	0.2	3218
LOC100912471	0.0%	0.2%	0.2	3219
Atp6v0e2	0.0%	0.2%	0.2	3220
Kitlg	0.0%	0.2%	0.2	3221
LOC103694394	0.0%	0.2%	0.2	3222
LOC108351277	0.0%	0.2%	0.2	3223
Ogdhl	0.0%	0.2%	0.2	3224
Sytl1	0.0%	0.2%	0.2	3225
Tmem249	0.0%	0.2%	0.2	3226
LOC102553476	0.0%	0.2%	0.2	3227
LOC102550584	0.0%	0.2%	0.2	3228

Atl1	0.0%	0.2%	0.2	3229
LOC102548320	0.0%	0.2%	0.2	3230
LOC103690111	0.0%	0.2%	0.2	3231
RGD1564482	0.0%	0.2%	0.2	3232
Traf6	0.0%	0.2%	0.2	3233
LOC108351351	0.0%	0.2%	0.2	3234
Atp1a2	0.0%	0.2%	0.2	3235
LOC102549576	0.0%	0.2%	0.2	3236
Prkd2	0.0%	0.2%	0.2	3237
LOC102552268	0.0%	0.2%	0.2	3238
Pilra	0.0%	0.2%	0.2	3239
Cnn1	0.0%	0.2%	0.2	3240
Itgam	0.0%	0.2%	0.2	3241
LOC102552490	0.0%	0.2%	0.2	3242
LOC102552316	0.0%	0.2%	0.2	3243
LOC365837	0.0%	0.2%	0.2	3244
Col4a1	0.0%	0.2%	0.2	3245
Scn3b	0.0%	0.2%	0.2	3246
LOC108353753	0.0%	0.2%	0.2	3247
Cadps	0.0%	0.2%	0.2	3248
Sp6	0.0%	0.2%	0.2	3249
LOC108348453	0.0%	0.2%	0.2	3250
LOC680910	0.0%	0.2%	0.2	3251
LOC499584	0.0%	0.2%	0.2	3252
LOC102556290	0.0%	0.2%	0.2	3253
Sphk1	0.0%	0.2%	0.2	3254
Nsf	0.0%	0.2%	0.2	3255
Cyth4	0.0%	0.2%	0.2	3256
Tbxas1	0.0%	0.2%	0.2	3257
Mex3b	0.0%	0.2%	0.2	3258
Rec114	0.0%	0.2%	0.2	3259
LOC103691272	0.0%	0.2%	0.2	3260
LOC685352	0.0%	0.2%	0.2	3261
Plcb3	0.0%	0.2%	0.2	3262
LOC102550725	0.0%	0.2%	0.2	3263
Trpv2	0.0%	0.2%	0.2	3264
Sema6b	0.0%	0.2%	0.2	3265
LOC103693523	0.0%	0.2%	0.2	3266
LOC108348157	0.0%	0.2%	0.2	3267
Dusp12	0.0%	0.2%	0.2	3268
LOC100911239	0.0%	0.2%	0.2	3269
Lnc012	0.0%	0.2%	0.2	3270
Serpinb6	0.0%	0.2%	0.2	3271
Tnfrsf11a	0.0%	0.2%	0.2	3272
LOC680435	0.0%	0.2%	0.2	3273
LOC100909739	0.0%	0.2%	0.2	3274
Fgfr1	0.0%	0.2%	0.2	3275
LOC501297	0.0%	0.2%	0.2	3276
Inpp4b	0.0%	0.2%	0.2	3277
LOC102548133	0.0%	0.2%	0.2	3278
Lexm	0.0%	0.2%	0.2	3279
Mmp17	0.0%	0.2%	0.2	3280
LOC108348837	0.0%	0.2%	0.2	3281
RGD1562156	0.0%	0.2%	0.2	3282
LOC103694297	0.0%	0.2%	0.2	3283
Cited4	0.0%	0.2%	0.2	3284
Amh	0.0%	0.2%	0.2	3285
LOC100362366	41.2%	41.4%	0.2	3286
Coq7	3.5%	3.8%	0.2	3287
Tma7	15.7%	15.9%	0.2	3288
Dcaf6	2.7%	3.0%	0.2	3289
Ppp1r21	2.7%	3.0%	0.2	3290
LOC102549099	2.4%	2.6%	0.2	3291
LOC102549726	2.4%	2.6%	0.2	3292
LOC103695026	2.4%	2.6%	0.2	3293
LOC108350082	43.9%	44.1%	0.2	3294

Pycr2	2.0%	2.2%	0.2	3295
Zbtb9	2.0%	2.2%	0.2	3296
LOC102546302	2.0%	2.2%	0.2	3297
Siva1	2.0%	2.2%	0.2	3298
Atp2b2	1.6%	1.8%	0.2	3299
LOC100912029	1.6%	1.8%	0.2	3300
Zfp347	1.6%	1.8%	0.2	3301
Golga1	1.6%	1.8%	0.2	3302
Kifc3	1.6%	1.8%	0.2	3303
Mob3b	1.2%	1.4%	0.2	3304
LOC102548342	1.2%	1.4%	0.2	3305
Tcf20	1.2%	1.4%	0.2	3306
LOC103691872	1.2%	1.4%	0.2	3307
Agk	1.2%	1.4%	0.2	3308
Rab13	1.2%	1.4%	0.2	3309
RGD1564492	1.2%	1.4%	0.2	3310
Thop1	1.2%	1.4%	0.2	3311
Stk4	1.2%	1.4%	0.2	3312
Nudt16	1.2%	1.4%	0.2	3313
Qrsl1	1.2%	1.4%	0.2	3314
LOC103692217	1.2%	1.4%	0.2	3315
Trub1	1.2%	1.4%	0.2	3316
Pgm2	1.2%	1.4%	0.2	3317
Sesn3	1.2%	1.4%	0.2	3318
Rcn1	1.2%	1.4%	0.2	3319
LOC102550711	1.2%	1.4%	0.2	3320
LOC102550520	1.2%	1.4%	0.2	3321
Pm20d2	1.2%	1.4%	0.2	3322
Paip1	9.4%	9.6%	0.2	3323
Ccdc159	0.8%	1.0%	0.2	3324
LOC103691929	0.8%	1.0%	0.2	3325
Tcf19	0.8%	1.0%	0.2	3326
LOC102548600	0.8%	1.0%	0.2	3327
NEWGENE_1304700	0.8%	1.0%	0.2	3328
Pam	0.8%	1.0%	0.2	3329
Pwwp2a	0.8%	1.0%	0.2	3330
Pigm	0.8%	1.0%	0.2	3331
Tctex1d2	0.8%	1.0%	0.2	3332
Sema7a	0.8%	1.0%	0.2	3333
Srbd1	0.8%	1.0%	0.2	3334
Med9	0.8%	1.0%	0.2	3335
Pcyox1	0.8%	1.0%	0.2	3336
Helb	0.8%	1.0%	0.2	3337
Khnyn	0.8%	1.0%	0.2	3338
Foxo4	0.8%	1.0%	0.2	3339
Dapp1	0.8%	1.0%	0.2	3340
Hist1h2ak	0.8%	1.0%	0.2	3341
Cep152	0.8%	1.0%	0.2	3342
Mfsd9	0.4%	0.6%	0.2	3343
RGD621098	0.4%	0.6%	0.2	3344
Ice2	0.4%	0.6%	0.2	3345
Exoc3l4	0.4%	0.6%	0.2	3346
Alkbh8	0.4%	0.6%	0.2	3347
LOC108352194	0.4%	0.6%	0.2	3348
Clasp2	0.4%	0.6%	0.2	3349
Ell3	0.4%	0.6%	0.2	3350
Ces2e	0.4%	0.6%	0.2	3351
Gtf3c6	0.4%	0.6%	0.2	3352
LOC103690120	0.4%	0.6%	0.2	3353
Zfp691	0.4%	0.6%	0.2	3354
Zpbp	0.4%	0.6%	0.2	3355
Lhx6	0.4%	0.6%	0.2	3356
Prr15	0.4%	0.6%	0.2	3357
Rnf111l	0.4%	0.6%	0.2	3358
LOC102546531	0.4%	0.6%	0.2	3359
Zfp512	0.4%	0.6%	0.2	3360

Cemip	0.4%	0.6%	0.2	3361
Sft2d3	0.4%	0.6%	0.2	3362
Zc3h12c	0.4%	0.6%	0.2	3363
Zfp362	0.4%	0.6%	0.2	3364
LOC498601	0.4%	0.6%	0.2	3365
LOC102547189	0.4%	0.6%	0.2	3366
Sh2d1b2	0.4%	0.6%	0.2	3367
LOC102546740	0.4%	0.6%	0.2	3368
Proser1	0.4%	0.6%	0.2	3369
LOC102548682	0.4%	0.6%	0.2	3370
Klhl26	0.4%	0.6%	0.2	3371
Rnf166	0.4%	0.6%	0.2	3372
LOC102553824	0.4%	0.6%	0.2	3373
LOC108350691	0.4%	0.6%	0.2	3374
Tbc1d22b	0.4%	0.6%	0.2	3375
Kiz	0.4%	0.6%	0.2	3376
LOC108348965	0.4%	0.6%	0.2	3377
LOC102549347	0.4%	0.6%	0.2	3378
Mmgt2	0.4%	0.6%	0.2	3379
LOC102548818	0.4%	0.6%	0.2	3380
LOC102552527	0.4%	0.6%	0.2	3381
LOC100911990	0.4%	0.6%	0.2	3382
LOC102548659	0.4%	0.6%	0.2	3383
LOC100912991	0.4%	0.6%	0.2	3384
LOC102553833	0.4%	0.6%	0.2	3385
Nprl3	0.4%	0.6%	0.2	3386
Nup58	0.4%	0.6%	0.2	3387
Gpr19	0.4%	0.6%	0.2	3388
LOC102554909	0.4%	0.6%	0.2	3389
Nup210	0.4%	0.6%	0.2	3390
LOC100911901	0.4%	0.6%	0.2	3391
Mcm9	0.4%	0.6%	0.2	3392
LOC108348117	0.4%	0.6%	0.2	3393
LOC103690175	0.4%	0.6%	0.2	3394
Cep290	0.4%	0.6%	0.2	3395
Pogz	0.4%	0.6%	0.2	3396
Foxo1	0.4%	0.6%	0.2	3397
Ulk3	0.4%	0.6%	0.2	3398
Hdac7	0.4%	0.6%	0.2	3399
Anks1a	0.4%	0.6%	0.2	3400
Kif22	0.4%	0.6%	0.2	3401
N4bp1	4.3%	4.5%	0.2	3402
Acat2	12.5%	12.7%	0.2	3403
Foxr2	0.0%	0.2%	0.2	3404
Zscan18	0.0%	0.2%	0.2	3405
LOC103690060	0.0%	0.2%	0.2	3406
Ttc30a	0.0%	0.2%	0.2	3407
Celf4	0.0%	0.2%	0.2	3408
Kptn	0.0%	0.2%	0.2	3409
LOC108351025	0.0%	0.2%	0.2	3410
Triqk	0.0%	0.2%	0.2	3411
Trib2	0.0%	0.2%	0.2	3412
LOC108348083	0.0%	0.2%	0.2	3413
Actc1	0.0%	0.2%	0.2	3414
LOC100363107	0.0%	0.2%	0.2	3415
LOC100911951	0.0%	0.2%	0.2	3416
Bsph1	0.0%	0.2%	0.2	3417
Lilrb3b	0.0%	0.2%	0.2	3418
Parp8	0.0%	0.2%	0.2	3419
LOC102552805	0.0%	0.2%	0.2	3420
Wdfy4	0.0%	0.2%	0.2	3421
Arhgef39	0.0%	0.2%	0.2	3422
LOC102548949	0.0%	0.2%	0.2	3423
LOC102548542	0.0%	0.2%	0.2	3424
Cmah	0.0%	0.2%	0.2	3425
LOC102551666	0.0%	0.2%	0.2	3426

LOC679149	0.0%	0.2%	0.2	3427
LOC108348155	0.0%	0.2%	0.2	3428
LOC108352204	0.0%	0.2%	0.2	3429
Ccr3	0.0%	0.2%	0.2	3430
Btbd19	0.0%	0.2%	0.2	3431
Zfp7	0.0%	0.2%	0.2	3432
S100a1	0.0%	0.2%	0.2	3433
Pbk	0.0%	0.2%	0.2	3434
Rgs18	0.0%	0.2%	0.2	3435
LOC102554693	0.0%	0.2%	0.2	3436
Anxa3	0.0%	0.2%	0.2	3437
LOC108351914	0.0%	0.2%	0.2	3438
LOC367195	0.0%	0.2%	0.2	3439
LOC100359633	0.0%	0.2%	0.2	3440
LOC103690380	0.0%	0.2%	0.2	3441
Knl1	0.0%	0.2%	0.2	3442
LOC108348107	0.0%	0.2%	0.2	3443
LOC108349384	0.0%	0.2%	0.2	3444
LOC680663	0.0%	0.2%	0.2	3445
LOC102548791	0.0%	0.2%	0.2	3446
LOC108348147	0.0%	0.2%	0.2	3447
Gpr65	0.0%	0.2%	0.2	3448
Kdm6a	0.0%	0.2%	0.2	3449
LOC100912426	0.0%	0.2%	0.2	3450
Morn1	0.0%	0.2%	0.2	3451
Nhsl1	0.0%	0.2%	0.2	3452
LOC100365008	0.0%	0.2%	0.2	3453
LOC102547443	0.0%	0.2%	0.2	3454
Slc25a36	0.0%	0.2%	0.2	3455
LOC100911224	0.0%	0.2%	0.2	3456
Rufy2	0.0%	0.2%	0.2	3457
Fam19a3	0.0%	0.2%	0.2	3458
LOC100910288	0.0%	0.2%	0.2	3459
Ccnd2	0.0%	0.2%	0.2	3460
Klhl41	0.0%	0.2%	0.2	3461
Mpped2	0.0%	0.2%	0.2	3462
Lmnb1	0.0%	0.2%	0.2	3463
Zcwpw1	0.0%	0.2%	0.2	3464
Adam19	0.0%	0.2%	0.2	3465
Ldb2	0.0%	0.2%	0.2	3466
LOC103690421	0.0%	0.2%	0.2	3467
LOC690097	0.0%	0.2%	0.2	3468
Fut11	0.0%	0.2%	0.2	3469
LOC108348624	0.0%	0.2%	0.2	3470
LOC102557432	0.0%	0.2%	0.2	3471
Pdpn	0.0%	0.2%	0.2	3472
LOC103692165	0.0%	0.2%	0.2	3473
Arid3c	0.0%	0.2%	0.2	3474
LOC501467	0.0%	0.2%	0.2	3475
LOC108353796	0.0%	0.2%	0.2	3476
Mical1	0.0%	0.2%	0.2	3477
LOC102556109	0.0%	0.2%	0.2	3478
Pi16	0.0%	0.2%	0.2	3479
Dusp13	0.0%	0.2%	0.2	3480
RGD1564463	0.0%	0.2%	0.2	3481
Sptbn4	0.0%	0.2%	0.2	3482
Bmp5	0.0%	0.2%	0.2	3483
Stom	0.0%	0.2%	0.2	3484
LOC102550737	0.0%	0.2%	0.2	3485
Srgap1	0.0%	0.2%	0.2	3486
Opn3	0.0%	0.2%	0.2	3487
Fam198b	0.0%	0.2%	0.2	3488
LOC108348564	0.0%	0.2%	0.2	3489
LOC102552386	0.0%	0.2%	0.2	3490
Ggn	0.0%	0.2%	0.2	3491
Actbl2	0.0%	0.2%	0.2	3492

B4galt6	0.0%	0.2%	0.2	3493
LOC102547310	0.0%	0.2%	0.2	3494
LOC103692748	0.0%	0.2%	0.2	3495
Kctd13	0.0%	0.2%	0.2	3496
Zfp945	0.0%	0.2%	0.2	3497
Rapgef3	0.0%	0.2%	0.2	3498
LOC108353717	0.0%	0.2%	0.2	3499
Sult1d1	0.0%	0.2%	0.2	3500
LOC102553962	0.0%	0.2%	0.2	3501
LOC687532	0.0%	0.2%	0.2	3502
Uevld	0.0%	0.2%	0.2	3503
Gna11	0.0%	0.2%	0.2	3504
LOC102546647	0.0%	0.2%	0.2	3505
Cenpe	0.0%	0.2%	0.2	3506
Fes	0.0%	0.2%	0.2	3507
Adgra2	0.0%	0.2%	0.2	3508
Mob1b	0.0%	0.2%	0.2	3509
Dok1	0.0%	0.2%	0.2	3510
LOC100360774	0.0%	0.2%	0.2	3511
Fbxl17	0.0%	0.2%	0.2	3512
Ccdc122	0.0%	0.2%	0.2	3513
Tnfrsf13b	0.0%	0.2%	0.2	3514
LOC102548073	0.0%	0.2%	0.2	3515
Oxct1	0.0%	0.2%	0.2	3516
Amotl1	0.0%	0.2%	0.2	3517
Calcrl	0.0%	0.2%	0.2	3518
Mterf2	0.0%	0.2%	0.2	3519
RGD1309104	0.0%	0.2%	0.2	3520
LOC108352894	0.0%	0.2%	0.2	3521
Kif2c	0.0%	0.2%	0.2	3522
LOC100911814	0.0%	0.2%	0.2	3523
LOC108351424	0.0%	0.2%	0.2	3524
Nfam1	0.0%	0.2%	0.2	3525
RGD1305298	0.0%	0.2%	0.2	3526
LOC102555770	0.0%	0.2%	0.2	3527
Psma8	0.0%	0.2%	0.2	3528
Cdca7	0.0%	0.2%	0.2	3529
Pik3ip1	0.0%	0.2%	0.2	3530
Ptp4a3	0.0%	0.2%	0.2	3531
LOC365949	0.0%	0.2%	0.2	3532
Hpgds	0.0%	0.2%	0.2	3533
Slco1a6	0.0%	0.2%	0.2	3534
LOC100912312	0.0%	0.2%	0.2	3535
Treml4	0.0%	0.2%	0.2	3536
Itga2	0.0%	0.2%	0.2	3537
Fcrl2	0.0%	0.2%	0.2	3538
Anln	0.0%	0.2%	0.2	3539
LOC102555813	0.0%	0.2%	0.2	3540
LOC103693555	0.0%	0.2%	0.2	3541
LOC102554672	0.0%	0.2%	0.2	3542
Rpgr	0.0%	0.2%	0.2	3543
Mis18bp1	0.0%	0.2%	0.2	3544
LOC102557094	0.0%	0.2%	0.2	3545
LOC108350488	0.0%	0.2%	0.2	3546
Arhgef4	0.0%	0.2%	0.2	3547
Plcg2	0.0%	0.2%	0.2	3548
LOC102551017	0.0%	0.2%	0.2	3549
Anln11	0.0%	0.2%	0.2	3550
Glb1l2	0.0%	0.2%	0.2	3551
Cdkn2a	0.0%	0.2%	0.2	3552
Ddr2	0.0%	0.2%	0.2	3553
LOC108350508	0.0%	0.2%	0.2	3554
LOC103691004	0.0%	0.2%	0.2	3555
Ikzf2	0.0%	0.2%	0.2	3556
Cacna2d2	0.0%	0.2%	0.2	3557
Fam53b	0.0%	0.2%	0.2	3558

Apobec3b	0.0%	0.2%	0.2	3559
Cox6c-ps1	0.0%	0.2%	0.2	3560
Adamts14	0.0%	0.2%	0.2	3561
LOC103690134	0.0%	0.2%	0.2	3562
RGD1565071	0.0%	0.2%	0.2	3563
LOC102550409	0.0%	0.2%	0.2	3564
Cfap77	0.0%	0.2%	0.2	3565
Efcab6	0.0%	0.2%	0.2	3566
Pla2g4b	0.0%	0.2%	0.2	3567
Nppc	0.0%	0.2%	0.2	3568
Fancg	0.0%	0.2%	0.2	3569
LOC684466	0.0%	0.2%	0.2	3570
Prss2	0.0%	0.2%	0.2	3571
Adamts10	0.0%	0.2%	0.2	3572
Il2ra	0.0%	0.2%	0.2	3573
Cx3cr1	0.0%	0.2%	0.2	3574
LOC102556346	0.0%	0.2%	0.2	3575
Art3	0.0%	0.2%	0.2	3576
Slc27a3	0.0%	0.2%	0.2	3577
Pasd1	0.0%	0.2%	0.2	3578
Ninj2	0.0%	0.2%	0.2	3579
LOC100361706	0.0%	0.2%	0.2	3580
LOC108350368	0.0%	0.2%	0.2	3581
Pcdhga8	0.0%	0.2%	0.2	3582
Gpr161	0.0%	0.2%	0.2	3583
Dph2	0.0%	0.2%	0.2	3584
Col13a1	0.0%	0.2%	0.2	3585
LOC102552590	0.0%	0.2%	0.2	3586
RGD1565987	0.0%	0.2%	0.2	3587
Zfp541	0.0%	0.2%	0.2	3588
Bex4	0.0%	0.2%	0.2	3589
Hist2h3c2	0.0%	0.2%	0.2	3590
LOC102547754	0.0%	0.2%	0.2	3591
Arfgef3	0.0%	0.2%	0.2	3592
Eme1	0.0%	0.2%	0.2	3593
Far1	0.0%	0.2%	0.2	3594
LOC108351605	0.0%	0.2%	0.2	3595
Ms4a6b	0.0%	0.2%	0.2	3596
Card11	0.0%	0.2%	0.2	3597
Gpr18	0.0%	0.2%	0.2	3598
Vwa1	0.0%	0.2%	0.2	3599
LOC102556459	0.0%	0.2%	0.2	3600
LOC100912659	0.0%	0.2%	0.2	3601
Fam107a	0.0%	0.2%	0.2	3602
Pcdhga10	0.0%	0.2%	0.2	3603
Tlr12	0.0%	0.2%	0.2	3604
LOC103690935	0.0%	0.2%	0.2	3605
Lpcat4	0.0%	0.2%	0.2	3606
Gcsam	0.0%	0.2%	0.2	3607
Rhbdl1	0.0%	0.2%	0.2	3608
Cpne7	0.0%	0.2%	0.2	3609
LOC103690249	0.0%	0.2%	0.2	3610
Dcn	0.0%	0.2%	0.2	3611
Evc	0.0%	0.2%	0.2	3612
Npas3	0.0%	0.2%	0.2	3613
LOC102552229	0.0%	0.2%	0.2	3614
Lnc016	0.0%	0.2%	0.2	3615
Doc2g	0.0%	0.2%	0.2	3616
LOC103691921	0.0%	0.2%	0.2	3617
Strip2	0.0%	0.2%	0.2	3618
Ahsp	0.0%	0.2%	0.2	3619
LOC108353603	0.0%	0.2%	0.2	3620
Tmcc2	0.0%	0.2%	0.2	3621
LOC102556093	0.0%	0.2%	0.2	3622
Sgo1	0.0%	0.2%	0.2	3623
Muc1	0.0%	0.2%	0.2	3624

Cldn4	0.0%	0.2%	0.2	3625
Pygb	0.0%	0.2%	0.2	3626
LOC100910801	0.0%	0.2%	0.2	3627
NEWGENE_621351	0.0%	0.2%	0.2	3628
LOC100363314	0.0%	0.2%	0.2	3629
Prr7	0.0%	0.2%	0.2	3630
Gtse1	0.0%	0.2%	0.2	3631
Ccdc64	0.0%	0.2%	0.2	3632
Atp13a3	0.0%	0.2%	0.2	3633
LOC108349370	0.0%	0.2%	0.2	3634
LOC102556034	0.0%	0.2%	0.2	3635
LOC100911964	0.0%	0.2%	0.2	3636
LOC103694912	0.0%	0.2%	0.2	3637
LOC681290	0.0%	0.2%	0.2	3638
LOC108348125	0.0%	0.2%	0.2	3639
Setmar	0.0%	0.2%	0.2	3640
Esco2	0.0%	0.2%	0.2	3641
Mdfi	0.0%	0.2%	0.2	3642
LOC102548638	0.0%	0.2%	0.2	3643
Tnnt1	0.0%	0.2%	0.2	3644
LOC108353666	0.0%	0.2%	0.2	3645
Nlrc3	0.0%	0.2%	0.2	3646
LOC102556463	0.0%	0.2%	0.2	3647
Pank4	0.0%	0.2%	0.2	3648
LOC100361866	0.0%	0.2%	0.2	3649
Clec10a	0.0%	0.2%	0.2	3650
Egfl7	0.0%	0.2%	0.2	3651
LOC102555426	0.0%	0.2%	0.2	3652
LOC691546	0.0%	0.2%	0.2	3653
LOC100909512	0.0%	0.2%	0.2	3654
Rab37	0.0%	0.2%	0.2	3655
Lilrb4	0.0%	0.2%	0.2	3656
Fam131a	0.0%	0.2%	0.2	3657
Cd33	0.0%	0.2%	0.2	3658
Nipal3	0.0%	0.2%	0.2	3659
LOC691141	0.0%	0.2%	0.2	3660
LOC100364435	0.0%	0.2%	0.2	3661
Zfp334	0.0%	0.2%	0.2	3662
LOC108351187	0.0%	0.2%	0.2	3663
Ccdc65	0.0%	0.2%	0.2	3664
Acsbg2	0.0%	0.2%	0.2	3665
Wipf3	0.0%	0.2%	0.2	3666
LOC108353788	0.0%	0.2%	0.2	3667
LOC102552373	0.0%	0.2%	0.2	3668
Wbscr27	0.0%	0.2%	0.2	3669
Emilin1	0.0%	0.2%	0.2	3670
LOC103693417	0.0%	0.2%	0.2	3671
Ednrb	0.0%	0.2%	0.2	3672
LOC680377	0.0%	0.2%	0.2	3673
Mcpt8	0.0%	0.2%	0.2	3674
LOC108352449	0.0%	0.2%	0.2	3675
St14	0.0%	0.2%	0.2	3676
Mefv	0.0%	0.2%	0.2	3677
LOC102557105	0.0%	0.2%	0.2	3678
LOC103692721	0.0%	0.2%	0.2	3679
LOC102555556	0.0%	0.2%	0.2	3680
LOC100912526	0.0%	0.2%	0.2	3681
Rfx8	0.0%	0.2%	0.2	3682
Rnase6	0.0%	0.2%	0.2	3683
LOC108351052	0.0%	0.2%	0.2	3684
Depdc1	0.0%	0.2%	0.2	3685
Coprs	0.0%	0.2%	0.2	3686
LOC100910422	0.0%	0.2%	0.2	3687
Kif21b	0.0%	0.2%	0.2	3688
Tssk4	0.0%	0.2%	0.2	3689
Uhrf1	0.0%	0.2%	0.2	3690

Dusp26	0.0%	0.2%	0.2	3691
Dcdc2	0.0%	0.2%	0.2	3692
RGD1560108	0.0%	0.2%	0.2	3693
LOC108352750	0.0%	0.2%	0.2	3694
Lrfn4	0.0%	0.2%	0.2	3695
LOC102552668	0.0%	0.2%	0.2	3696
Col3a1	0.0%	0.2%	0.2	3697
LOC103692784	0.0%	0.2%	0.2	3698
LOC108352057	0.0%	0.2%	0.2	3699
Sh3gl2	0.0%	0.2%	0.2	3700
LOC108353306	0.0%	0.2%	0.2	3701
Dnajc28	0.0%	0.2%	0.2	3702
Ctdspl2	0.0%	0.2%	0.2	3703
LOC103689957	0.0%	0.2%	0.2	3704
Tssk6	0.0%	0.2%	0.2	3705
Cxcl6	0.0%	0.2%	0.2	3706
LOC102557038	0.0%	0.2%	0.2	3707
LOC102547606	0.0%	0.2%	0.2	3708
LOC685716	0.0%	0.2%	0.2	3709
LOC102547556	0.0%	0.2%	0.2	3710
LOC102557338	0.0%	0.2%	0.2	3711
Cdca2	0.0%	0.2%	0.2	3712
Anxa8	0.0%	0.2%	0.2	3713
Ttll7	0.0%	0.2%	0.2	3714
Tiam1	0.0%	0.2%	0.2	3715
Lpcat2	0.0%	0.2%	0.2	3716
LOC102551257	0.0%	0.2%	0.2	3717
LOC100912350	0.0%	0.2%	0.2	3718
Syngr4	0.0%	0.2%	0.2	3719
Rcn3	0.0%	0.2%	0.2	3720
Chm	0.0%	0.2%	0.2	3721
Slfn13	0.0%	0.2%	0.2	3722
LOC102552195	0.0%	0.2%	0.2	3723
Nat8b	0.0%	0.2%	0.2	3724
Pnmal1	0.0%	0.2%	0.2	3725
Clec9a	0.0%	0.2%	0.2	3726
Dusp15	0.0%	0.2%	0.2	3727
LOC103693676	0.0%	0.2%	0.2	3728
Cenpu	0.0%	0.2%	0.2	3729
Zfp618	0.0%	0.2%	0.2	3730
Sh2d3c	0.0%	0.2%	0.2	3731
Papolb	0.0%	0.2%	0.2	3732
Fam188b	0.0%	0.2%	0.2	3733
LOC103691663	0.0%	0.2%	0.2	3734
Pde4c	0.0%	0.2%	0.2	3735
LOC108352408	0.0%	0.2%	0.2	3736
LOC102551420	0.0%	0.2%	0.2	3737
Fgd6	0.0%	0.2%	0.2	3738
LOC108353657	0.0%	0.2%	0.2	3739
Rasip1	0.0%	0.2%	0.2	3740
Zfp36l3	0.0%	0.2%	0.2	3741
LOC100912485	0.0%	0.2%	0.2	3742
Gdpd5	0.0%	0.2%	0.2	3743
LOC102550403	0.0%	0.2%	0.2	3744
LOC100910447	0.0%	0.2%	0.2	3745
Hist2h2be	0.0%	0.2%	0.2	3746
Afap1l1	0.0%	0.2%	0.2	3747
LOC103694502	0.0%	0.2%	0.2	3748
Tagap	0.0%	0.2%	0.2	3749
Ppp2r2b	0.0%	0.2%	0.2	3750
LOC108350688	0.0%	0.2%	0.2	3751
Ccr6	0.0%	0.2%	0.2	3752
Naaa	0.0%	0.2%	0.2	3753
Fzd7	0.0%	0.2%	0.2	3754
Sirpb2l1	0.0%	0.2%	0.2	3755
LOC108349643	0.0%	0.2%	0.2	3756

Gna15	0.0%	0.2%	0.2	3757
LOC108353448	0.0%	0.2%	0.2	3758
LOC102546977	0.0%	0.2%	0.2	3759
Garnl3	0.0%	0.2%	0.2	3760
LOC102546790	0.0%	0.2%	0.2	3761
Slc24a5	0.0%	0.2%	0.2	3762
Kcnj8	0.0%	0.2%	0.2	3763
Elf5	0.0%	0.2%	0.2	3764
LOC102553924	0.0%	0.2%	0.2	3765
Gsn	0.0%	0.2%	0.2	3766
Bche	0.0%	0.2%	0.2	3767
Ss18l1	0.0%	0.2%	0.2	3768
Tarsl2	0.0%	0.2%	0.2	3769
Nsg1	0.0%	0.2%	0.2	3770
Pdzd9	0.0%	0.2%	0.2	3771
Rhoj	0.0%	0.2%	0.2	3772
Trim63	0.0%	0.2%	0.2	3773
LOC100911610	0.0%	0.2%	0.2	3774
Qk	0.0%	0.2%	0.2	3775
Tspyl4	0.0%	0.2%	0.2	3776
Zbtb37	0.0%	0.2%	0.2	3777
Tex21	0.0%	0.2%	0.2	3778
Camk2d	0.0%	0.2%	0.2	3779
Lmln	0.0%	0.2%	0.2	3780
Tirap	0.0%	0.2%	0.2	3781
Wdr59	0.0%	0.2%	0.2	3782
Pdzd4	0.0%	0.2%	0.2	3783
LOC108353450	0.0%	0.2%	0.2	3784
LOC108352130	0.0%	0.2%	0.2	3785
Dpysl5	0.0%	0.2%	0.2	3786
LOC108350966	0.0%	0.2%	0.2	3787
LOC102548964	0.0%	0.2%	0.2	3788
Kctd14	0.0%	0.2%	0.2	3789
Gstm6l	0.0%	0.2%	0.2	3790
Mxra8	0.0%	0.2%	0.2	3791
RGD1563150	0.0%	0.2%	0.2	3792
Kctd17	0.0%	0.2%	0.2	3793
Cc2d2b	0.0%	0.2%	0.2	3794
RGD1560556	0.0%	0.2%	0.2	3795
Als2cr12	0.0%	0.2%	0.2	3796
Ankrd1	0.0%	0.2%	0.2	3797
Rai1	0.0%	0.2%	0.2	3798
RGD1564405	0.0%	0.2%	0.2	3799
Tlr7	0.0%	0.2%	0.2	3800
Cenpn	0.0%	0.2%	0.2	3801
LOC108348197	0.0%	0.2%	0.2	3802
Nrcam	0.0%	0.2%	0.2	3803
LOC103690082	0.0%	0.2%	0.2	3804
Pclaf	0.0%	0.2%	0.2	3805
Oscp1	0.0%	0.2%	0.2	3806
LOC102555476	0.0%	0.2%	0.2	3807
Klhdc1	0.0%	0.2%	0.2	3808
RGD1565472	0.0%	0.2%	0.2	3809
Msx1	0.0%	0.2%	0.2	3810
Serpini1	0.0%	0.2%	0.2	3811
Limk1	0.0%	0.2%	0.2	3812
LOC298795	0.0%	0.2%	0.2	3813
Arl10	0.0%	0.2%	0.2	3814
Tpm2	0.0%	0.2%	0.2	3815
Fbln5	0.0%	0.2%	0.2	3816
Pafah2	0.0%	0.2%	0.2	3817
Serpinb5	0.0%	0.2%	0.2	3818
LOC100912265	0.0%	0.2%	0.2	3819
LOC103694037	0.0%	0.2%	0.2	3820
LOC102549107	0.0%	0.2%	0.2	3821
Cracr2a	0.0%	0.2%	0.2	3822

Kbtbd6	0.0%	0.2%	0.2	3823
LOC100910792	0.0%	0.2%	0.2	3824
Cda	0.0%	0.2%	0.2	3825
Ccr9	0.0%	0.2%	0.2	3826
Tceal1	0.0%	0.2%	0.2	3827
Cd19	0.0%	0.2%	0.2	3828
Slco3a1	0.0%	0.2%	0.2	3829
Pcdhgc3	0.0%	0.2%	0.2	3830
Mypop	0.0%	0.2%	0.2	3831
LOC100912974	0.0%	0.2%	0.2	3832
Myt1	0.0%	0.2%	0.2	3833
Gpr157	0.0%	0.2%	0.2	3834
Thbs1	0.0%	0.2%	0.2	3835
Nrarp	0.0%	0.2%	0.2	3836
Cd96	0.0%	0.2%	0.2	3837
LOC102547621	0.0%	0.2%	0.2	3838
Slfn14	0.0%	0.2%	0.2	3839
Atp10d	0.0%	0.2%	0.2	3840
Per3	0.0%	0.2%	0.2	3841
Cxcr6	0.0%	0.2%	0.2	3842
Atp6v1c2	0.0%	0.2%	0.2	3843
LOC103690552	0.0%	0.2%	0.2	3844
LOC102556805	0.0%	0.2%	0.2	3845
Zfp2	0.0%	0.2%	0.2	3846
LOC103690399	0.0%	0.2%	0.2	3847
Fhad1	0.0%	0.2%	0.2	3848
LOC100909664	0.0%	0.2%	0.2	3849
Ccdc158	0.0%	0.2%	0.2	3850
LOC108352837	0.0%	0.2%	0.2	3851
Slain1	0.0%	0.2%	0.2	3852
LOC100363228	0.0%	0.2%	0.2	3853
Mixl1	0.0%	0.2%	0.2	3854
LOC102551586	0.0%	0.2%	0.2	3855
LOC100363368	0.0%	0.2%	0.2	3856
Col6a2	0.0%	0.2%	0.2	3857
Mapk4	0.0%	0.2%	0.2	3858
LOC103692073	0.0%	0.2%	0.2	3859
Celsr2	0.0%	0.2%	0.2	3860
Ephb3	0.0%	0.2%	0.2	3861
Zik1	0.0%	0.2%	0.2	3862
Wdr76	0.0%	0.2%	0.2	3863
Ccdc74a	0.0%	0.2%	0.2	3864
LOC108349034	0.0%	0.2%	0.2	3865
Slc25a52	0.0%	0.2%	0.2	3866
Cygb	0.0%	0.2%	0.2	3867
Lzts2	0.0%	0.2%	0.2	3868
Ikzf1	0.0%	0.2%	0.2	3869
LOC102548459	0.0%	0.2%	0.2	3870
Map2k7	0.0%	0.2%	0.2	3871
Ubash3a	0.0%	0.2%	0.2	3872
LOC102550203	0.0%	0.2%	0.2	3873
LOC108351581	0.0%	0.2%	0.2	3874
RT1-T24-2	0.0%	0.2%	0.2	3875
Aptr	0.0%	0.2%	0.2	3876
Gmnc	0.0%	0.2%	0.2	3877
Clmp	0.0%	0.2%	0.2	3878
Sh2d1b	0.0%	0.2%	0.2	3879
LOC102557498	0.0%	0.2%	0.2	3880
Clec4m	0.0%	0.2%	0.2	3881
Asic5	0.0%	0.2%	0.2	3882
Trim72	0.0%	0.2%	0.2	3883
Ccdc88a	0.0%	0.2%	0.2	3884
Pcdhgb8	0.0%	0.2%	0.2	3885
LOC102557303	0.0%	0.2%	0.2	3886
Git1	0.0%	0.2%	0.2	3887
Hnf4g	0.0%	0.2%	0.2	3888

LOC102554945	0.0%	0.2%	0.2	3889
RGD1561157	0.0%	0.2%	0.2	3890
LOC100360809	0.0%	0.2%	0.2	3891
Gja4	0.0%	0.2%	0.2	3892
LOC103694226	0.0%	0.2%	0.2	3893
Myh13	0.0%	0.2%	0.2	3894
Plch1	0.0%	0.2%	0.2	3895
LOC103694580	0.0%	0.2%	0.2	3896
Rbpjl2	0.0%	0.2%	0.2	3897
LOC103695234	0.0%	0.2%	0.2	3898
Smpd5	0.0%	0.2%	0.2	3899
Stk36	0.0%	0.2%	0.2	3900
LOC102548844	0.0%	0.2%	0.2	3901
Slc10a5	0.0%	0.2%	0.2	3902
LOC102554203	0.0%	0.2%	0.2	3903
LOC102549163	0.0%	0.2%	0.2	3904
RGD1563323	0.0%	0.2%	0.2	3905
LOC103692570	0.0%	0.2%	0.2	3906
C1qtnf9	0.0%	0.2%	0.2	3907
LOC108350572	0.0%	0.2%	0.2	3908
Cyp4v3	0.0%	0.2%	0.2	3909
Qrich2	0.0%	0.2%	0.2	3910
Abi2	0.0%	0.2%	0.2	3911
Tspy26	0.0%	0.2%	0.2	3912
LOC102548345	0.0%	0.2%	0.2	3913
Casc3	0.0%	0.2%	0.2	3914
LOC102556515	0.0%	0.2%	0.2	3915
Spire1	0.0%	0.2%	0.2	3916
Sowaha	0.0%	0.2%	0.2	3917
Fbxl19	0.0%	0.2%	0.2	3918
Adam10	0.0%	0.2%	0.2	3919
Zfp192	0.0%	0.2%	0.2	3920
Aldh1a2	0.0%	0.2%	0.2	3921
Pcdhb17	0.0%	0.2%	0.2	3922
Pnlip	0.0%	0.2%	0.2	3923
Pask	0.0%	0.2%	0.2	3924
LOC102546453	0.0%	0.2%	0.2	3925
LOC103695162	0.0%	0.2%	0.2	3926
Adat1	0.0%	0.2%	0.2	3927
Chaf1b	0.0%	0.2%	0.2	3928
LOC108352768	0.0%	0.2%	0.2	3929
Cyp4f5	0.0%	0.2%	0.2	3930
Prr15l	0.0%	0.2%	0.2	3931
Sfxn4	0.0%	0.2%	0.2	3932
Pi4k2a	0.0%	0.2%	0.2	3933
RGD1559482	0.0%	0.2%	0.2	3934
Havcr2	0.0%	0.2%	0.2	3935
LOC102549834	0.0%	0.2%	0.2	3936
Nemp2	0.0%	0.2%	0.2	3937
Pcdhga7	0.0%	0.2%	0.2	3938
Spon1	0.0%	0.2%	0.2	3939
Ackr2	0.0%	0.2%	0.2	3940
LOC102554883	0.0%	0.2%	0.2	3941
LOC103693543	0.0%	0.2%	0.2	3942
Tmem237	0.0%	0.2%	0.2	3943
LOC102549247	0.0%	0.2%	0.2	3944
Hic2	0.0%	0.2%	0.2	3945
LOC103692717	0.0%	0.2%	0.2	3946
Mkl2	0.0%	0.2%	0.2	3947
Marveld2	0.0%	0.2%	0.2	3948
Magi2	0.0%	0.2%	0.2	3949
LOC108349218	0.0%	0.2%	0.2	3950
Dock6	0.0%	0.2%	0.2	3951
Lad1	0.0%	0.2%	0.2	3952
Foxo3	0.0%	0.2%	0.2	3953
LOC100912484	0.0%	0.2%	0.2	3954

Gimap1	0.0%	0.2%	0.2	3955
LOC102550192	0.0%	0.2%	0.2	3956
LOC102547412	0.0%	0.2%	0.2	3957
LOC500845	0.0%	0.2%	0.2	3958
Pclo	0.0%	0.2%	0.2	3959
Cd300lg	0.0%	0.2%	0.2	3960
LOC103691813	0.0%	0.2%	0.2	3961
Mxd3	0.0%	0.2%	0.2	3962
Apol11a	0.0%	0.2%	0.2	3963
Hist2h2ab	0.0%	0.2%	0.2	3964
LOC102557166	0.0%	0.2%	0.2	3965
Lpar1	0.0%	0.2%	0.2	3966
Crhr1	0.0%	0.2%	0.2	3967
LOC108352323	0.0%	0.2%	0.2	3968
Klf2	0.0%	0.2%	0.2	3969
Ankrd26	0.0%	0.2%	0.2	3970
Tmprss12	0.0%	0.2%	0.2	3971
St6galnac2	0.0%	0.2%	0.2	3972
Ttc24	0.0%	0.2%	0.2	3973
Hmgxb4	0.0%	0.2%	0.2	3974
Clic5	0.0%	0.2%	0.2	3975
Exoc3l2	0.0%	0.2%	0.2	3976
Rtkn	0.0%	0.2%	0.2	3977
Xrcc2	0.0%	0.2%	0.2	3978
Jaml	0.0%	0.2%	0.2	3979
Arnt	0.0%	0.2%	0.2	3980
Elmo1	0.0%	0.2%	0.2	3981
LOC108352415	0.0%	0.2%	0.2	3982
Lamb2	0.0%	0.2%	0.2	3983
Mcpt10	0.0%	0.2%	0.2	3984
Ankrd53	0.0%	0.2%	0.2	3985
LOC497796	0.0%	0.2%	0.2	3986
LOC102552367	0.0%	0.2%	0.2	3987
Unc13d	0.0%	0.2%	0.2	3988
Cbx4	0.0%	0.2%	0.2	3989
Col11a2	0.0%	0.2%	0.2	3990
Txk	0.0%	0.2%	0.2	3991
LOC100912391	0.0%	0.2%	0.2	3992
Prmt6	0.0%	0.2%	0.2	3993
LOC108351541	0.0%	0.2%	0.2	3994
LOC103692471	0.0%	0.2%	0.2	3995
Gnb1l	0.0%	0.2%	0.2	3996
LOC102546692	0.0%	0.2%	0.2	3997
Kcnip2	0.0%	0.2%	0.2	3998
B3gnt9	0.0%	0.2%	0.2	3999
Da2-19	0.0%	0.2%	0.2	4000
LOC102557277	0.0%	0.2%	0.2	4001
Fsd1l	0.0%	0.2%	0.2	4002
LOC103692911	0.0%	0.2%	0.2	4003
Asah2	0.0%	0.2%	0.2	4004
RGD1305184	0.0%	0.2%	0.2	4005
Ift81	0.0%	0.2%	0.2	4006
Fcgr1a	0.0%	0.2%	0.2	4007
Prrt1	0.0%	0.2%	0.2	4008
LOC103690046	0.0%	0.2%	0.2	4009
LOC100909847	0.0%	0.2%	0.2	4010
Lacc1	0.0%	0.2%	0.2	4011
LOC103690090	0.0%	0.2%	0.2	4012
Hist1h2bf	0.0%	0.2%	0.2	4013
LOC108351067	0.0%	0.2%	0.2	4014
LOC100365995	0.0%	0.2%	0.2	4015
LOC108351595	0.0%	0.2%	0.2	4016
Gabpb1l	0.0%	0.2%	0.2	4017
Ckmt1	0.0%	0.2%	0.2	4018
Ispd	0.0%	0.2%	0.2	4019
LOC102554815	0.0%	0.2%	0.2	4020

LOC102547868	0.0%	0.2%	0.2	4021
Csf3r	0.0%	0.2%	0.2	4022
RGD1560455	0.0%	0.2%	0.2	4023
Hdac4	0.0%	0.2%	0.2	4024
Tmem204	0.0%	0.2%	0.2	4025
Hist1h2aa	0.0%	0.2%	0.2	4026
LOC102549117	0.0%	0.2%	0.2	4027
LOC102549044	0.0%	0.2%	0.2	4028
Lilra5	0.0%	0.2%	0.2	4029
Mpp3	0.0%	0.2%	0.2	4030
Lax1	0.0%	0.2%	0.2	4031
LOC100909455	0.0%	0.2%	0.2	4032
Pou2af1	0.0%	0.2%	0.2	4033
Exnef	0.0%	0.2%	0.2	4034
LOC108352632	0.0%	0.2%	0.2	4035
LOC108348213	0.0%	0.2%	0.2	4036
Sla	0.0%	0.2%	0.2	4037
LOC100910497	0.0%	0.2%	0.2	4038
LOC102555435	0.0%	0.2%	0.2	4039
Rbm15b	0.0%	0.2%	0.2	4040
Impad1	3.5%	3.7%	0.1	4041
Prorsd1	3.5%	3.7%	0.1	4042
Celf1	7.5%	7.6%	0.1	4043
LOC100359701	3.1%	3.3%	0.1	4044
Naga	7.1%	7.2%	0.1	4045
Usp46	2.7%	2.9%	0.1	4046
Yy1	2.4%	2.5%	0.1	4047
Serpinb9	6.3%	6.4%	0.1	4048
LOC680432	2.0%	2.1%	0.1	4049
Wbp11l1	2.0%	2.1%	0.1	4050
Leng1	2.0%	2.1%	0.1	4051
Gtpbp2	2.0%	2.1%	0.1	4052
Rhobtb1	2.0%	2.1%	0.1	4053
Zfp52	80.8%	80.9%	0.1	4054
RGD1564855	1.6%	1.7%	0.1	4055
Katnal2	1.6%	1.7%	0.1	4056
LOC103690155	1.6%	1.7%	0.1	4057
Eya3	1.6%	1.7%	0.1	4058
RGD1561849	1.6%	1.7%	0.1	4059
Tmem269	1.6%	1.7%	0.1	4060
Tmem165	1.6%	1.7%	0.1	4061
Sema3c	1.6%	1.7%	0.1	4062
LOC100910526	1.6%	1.7%	0.1	4063
Ccdc43	1.6%	1.7%	0.1	4064
Elmo2	1.6%	1.7%	0.1	4065
Lgals3	1.2%	1.3%	0.1	4066
Ndor1	1.2%	1.3%	0.1	4067
Tsen54	1.2%	1.3%	0.1	4068
LOC102554302	1.2%	1.3%	0.1	4069
Pde4d	1.2%	1.3%	0.1	4070
Ino80b	1.2%	1.3%	0.1	4071
Gga3	1.2%	1.3%	0.1	4072
Magohb	1.2%	1.3%	0.1	4073
Mmp19	1.2%	1.3%	0.1	4074
Nup188	1.2%	1.3%	0.1	4075
Arl16	1.2%	1.3%	0.1	4076
Ngly1	1.2%	1.3%	0.1	4077
Rogdi	9.4%	9.5%	0.1	4078
Dnph1	5.1%	5.2%	0.1	4079
Derl3	0.8%	0.9%	0.1	4080
Ift74	0.8%	0.9%	0.1	4081
Ddx17	0.8%	0.9%	0.1	4082
LOC102555570	0.8%	0.9%	0.1	4083
Epb41l4a	0.8%	0.9%	0.1	4084
LOC100910046	0.8%	0.9%	0.1	4085
Tnf	0.8%	0.9%	0.1	4086

Dqx1	0.8%	0.9%	0.1	4087
LOC100910100	0.8%	0.9%	0.1	4088
Ccnb1	0.8%	0.9%	0.1	4089
RGD1565560	0.8%	0.9%	0.1	4090
Zfp395	0.8%	0.9%	0.1	4091
Wrb	0.8%	0.9%	0.1	4092
Gramd1a	0.8%	0.9%	0.1	4093
LOC102550367	0.8%	0.9%	0.1	4094
Diaph2	0.8%	0.9%	0.1	4095
Rfx5	0.8%	0.9%	0.1	4096
Homer2	0.8%	0.9%	0.1	4097
Mtmr1	0.8%	0.9%	0.1	4098
LOC102549203	0.8%	0.9%	0.1	4099
Zc3h4	0.8%	0.9%	0.1	4100
Rnf41	0.8%	0.9%	0.1	4101
LOC499770	0.8%	0.9%	0.1	4102
NEWGENE_1582994	0.8%	0.9%	0.1	4103
Oas1f	0.8%	0.9%	0.1	4104
C2cd5	0.8%	0.9%	0.1	4105
Mettl17	0.8%	0.9%	0.1	4106
Psmc3ip	0.8%	0.9%	0.1	4107
Epg5	0.4%	0.5%	0.1	4108
Usp49	0.4%	0.5%	0.1	4109
Tpcn2	0.4%	0.5%	0.1	4110
Xpnpep3	0.4%	0.5%	0.1	4111
LOC102549694	0.4%	0.5%	0.1	4112
Psmd10	0.4%	0.5%	0.1	4113
LOC102551451	0.4%	0.5%	0.1	4114
Dzf17	0.4%	0.5%	0.1	4115
Ahi1	0.4%	0.5%	0.1	4116
LOC100910945	0.4%	0.5%	0.1	4117
Cep57l1	0.4%	0.5%	0.1	4118
LOC100361476	0.4%	0.5%	0.1	4119
Gins4	0.4%	0.5%	0.1	4120
Ddx11	0.4%	0.5%	0.1	4121
Miip	0.4%	0.5%	0.1	4122
LOC100910771	0.4%	0.5%	0.1	4123
Ccnb2	0.4%	0.5%	0.1	4124
Gnrh1	0.4%	0.5%	0.1	4125
Chek2	0.4%	0.5%	0.1	4126
Ugt2b	0.4%	0.5%	0.1	4127
LOC100911627	0.4%	0.5%	0.1	4128
Tmem181	0.4%	0.5%	0.1	4129
LOC102557454	0.4%	0.5%	0.1	4130
LOC102548648	0.4%	0.5%	0.1	4131
LOC102555670	0.4%	0.5%	0.1	4132
Uhmk1	0.4%	0.5%	0.1	4133
Nek3	0.4%	0.5%	0.1	4134
Mcmdc2	0.4%	0.5%	0.1	4135
LOC102550456	0.4%	0.5%	0.1	4136
Kbtbd12	0.4%	0.5%	0.1	4137
Mex3d	0.4%	0.5%	0.1	4138
Cops7b	0.4%	0.5%	0.1	4139
RGD1563709	0.4%	0.5%	0.1	4140
LOC103694995	0.4%	0.5%	0.1	4141
LOC103692533	0.4%	0.5%	0.1	4142
Nek11	0.4%	0.5%	0.1	4143
LOC680835	0.4%	0.5%	0.1	4144
LOC100909461	0.4%	0.5%	0.1	4145
Ppargc1a	0.4%	0.5%	0.1	4146
Map4k2	0.4%	0.5%	0.1	4147
Cxcl13	0.4%	0.5%	0.1	4148
RGD1560402	0.4%	0.5%	0.1	4149
Aste1	0.4%	0.5%	0.1	4150
Nck2	0.4%	0.5%	0.1	4151
Chrnb1	0.4%	0.5%	0.1	4152

Plpp7	0.4%	0.5%	0.1	4153
Arl4d	0.4%	0.5%	0.1	4154
Dnajb14	0.4%	0.5%	0.1	4155
LOC108348137	0.4%	0.5%	0.1	4156
Pnpla6	0.4%	0.5%	0.1	4157
LOC679581	0.4%	0.5%	0.1	4158
LOC102555398	0.4%	0.5%	0.1	4159
LOC100360320	0.4%	0.5%	0.1	4160
Rex2	0.4%	0.5%	0.1	4161
Clcn2	0.4%	0.5%	0.1	4162
Zmym6	0.4%	0.5%	0.1	4163
Sec22c	0.4%	0.5%	0.1	4164
LOC108351672	0.4%	0.5%	0.1	4165
Slc7a7	0.4%	0.5%	0.1	4166
RGD1563072	0.4%	0.5%	0.1	4167
Nudt13	0.4%	0.5%	0.1	4168
Lnpep	0.4%	0.5%	0.1	4169
LOC100910755	0.4%	0.5%	0.1	4170
Nuf2	0.4%	0.5%	0.1	4171
Pdgfrb	0.4%	0.5%	0.1	4172
Rabl3	0.4%	0.5%	0.1	4173
Fbxl21	0.4%	0.5%	0.1	4174
LOC100912573	0.4%	0.5%	0.1	4175
Slc25a14	0.4%	0.5%	0.1	4176
Efna4	0.4%	0.5%	0.1	4177
Eps15	0.4%	0.5%	0.1	4178
Pttg1	0.4%	0.5%	0.1	4179
RGD1562310	0.4%	0.5%	0.1	4180
LOC108348184	0.4%	0.5%	0.1	4181
LOC680288	0.4%	0.5%	0.1	4182
Slc30a10	0.4%	0.5%	0.1	4183
LOC102548902	0.4%	0.5%	0.1	4184
Sept4	0.0%	0.1%	0.1	4185
LOC102555139	0.0%	0.1%	0.1	4186
Grb10	0.0%	0.1%	0.1	4187
LOC103690147	0.0%	0.1%	0.1	4188
LOC681180	0.0%	0.1%	0.1	4189
Acss1	0.0%	0.1%	0.1	4190
Myo1c	0.0%	0.1%	0.1	4191
Dok4	0.0%	0.1%	0.1	4192
LOC102547504	0.0%	0.1%	0.1	4193
Hs3st3a1	0.0%	0.1%	0.1	4194
Trpm8	0.0%	0.1%	0.1	4195
LOC100910781	0.0%	0.1%	0.1	4196
LOC102557273	0.0%	0.1%	0.1	4197
LOC102552904	0.0%	0.1%	0.1	4198
Ppm1j	0.0%	0.1%	0.1	4199
Dtd2	0.0%	0.1%	0.1	4200
LOC103692061	0.0%	0.1%	0.1	4201
Celsr1	0.0%	0.1%	0.1	4202
Sema3f	0.0%	0.1%	0.1	4203
Pcsk1n	0.0%	0.1%	0.1	4204
Mmp24	0.0%	0.1%	0.1	4205
Rnf224	0.0%	0.1%	0.1	4206
LOC108350923	0.0%	0.1%	0.1	4207
Agbl5	0.0%	0.1%	0.1	4208
LOC102556374	0.0%	0.1%	0.1	4209
LOC100910934	0.0%	0.1%	0.1	4210
Tmem150b	0.0%	0.1%	0.1	4211
LOC108349295	0.0%	0.1%	0.1	4212
Syt11	0.0%	0.1%	0.1	4213
Krt7	0.0%	0.1%	0.1	4214
LOC102546990	0.0%	0.1%	0.1	4215
Cpa1	0.0%	0.1%	0.1	4216
Ccr2	0.0%	0.1%	0.1	4217
Tifab	0.0%	0.1%	0.1	4218

LOC103690834	0.0%	0.1%	0.1	4219
Nkr-p1c	0.0%	0.1%	0.1	4220
Xkr5	0.0%	0.1%	0.1	4221
LOC103694489	0.0%	0.1%	0.1	4222
Fanca	0.0%	0.1%	0.1	4223
LOC102553173	0.0%	0.1%	0.1	4224
LOC103693948	0.0%	0.1%	0.1	4225
LOC108348403	0.0%	0.1%	0.1	4226
Slit3	0.0%	0.1%	0.1	4227
LOC103693784	0.0%	0.1%	0.1	4228
LOC103695080	0.0%	0.1%	0.1	4229
LOC102549944	0.0%	0.1%	0.1	4230
Selp	0.0%	0.1%	0.1	4231
Krt23	0.0%	0.1%	0.1	4232
Cer1	0.0%	0.1%	0.1	4233
Zcchc18	0.0%	0.1%	0.1	4234
LOC103692885	0.0%	0.1%	0.1	4235
Ildr1	0.0%	0.1%	0.1	4236
Ibsp	0.0%	0.1%	0.1	4237
LOC103691999	0.0%	0.1%	0.1	4238
Ccdc136	0.0%	0.1%	0.1	4239
Vcan	0.0%	0.1%	0.1	4240
Olr1352	0.0%	0.1%	0.1	4241
Calhm2	0.0%	0.1%	0.1	4242
LOC108352957	0.0%	0.1%	0.1	4243
Ctcfl	0.0%	0.1%	0.1	4244
Cd244	0.0%	0.1%	0.1	4245
Tmem179	0.0%	0.1%	0.1	4246
LOC102548202	0.0%	0.1%	0.1	4247
Fam25a	0.0%	0.1%	0.1	4248
Kcne4	0.0%	0.1%	0.1	4249
C12H7orf61	0.0%	0.1%	0.1	4250
Zfp157	0.0%	0.1%	0.1	4251
Plcb4	0.0%	0.1%	0.1	4252
LOC103693041	0.0%	0.1%	0.1	4253
LOC103691841	0.0%	0.1%	0.1	4254
LOC102546746	0.0%	0.1%	0.1	4255
Defb14	0.0%	0.1%	0.1	4256
LOC108351281	0.0%	0.1%	0.1	4257
Abca7	0.0%	0.1%	0.1	4258
Wdr27	0.0%	0.1%	0.1	4259
LOC102548820	0.0%	0.1%	0.1	4260
LOC100910201	0.0%	0.1%	0.1	4261
Limch1	0.0%	0.1%	0.1	4262
Afap1l2	0.0%	0.1%	0.1	4263
LOC102557388	0.0%	0.1%	0.1	4264
LOC103693130	0.0%	0.1%	0.1	4265
LOC108349710	0.0%	0.1%	0.1	4266
LOC102547038	0.0%	0.1%	0.1	4267
LOC102546903	0.0%	0.1%	0.1	4268
LOC100912473	0.0%	0.1%	0.1	4269
LOC685181	0.0%	0.1%	0.1	4270
LOC685157	0.0%	0.1%	0.1	4271
LOC100912379	0.0%	0.1%	0.1	4272
Znf235	0.0%	0.1%	0.1	4273
Kbtbd11	0.0%	0.1%	0.1	4274
LOC102553329	0.0%	0.1%	0.1	4275
LOC108351885	0.0%	0.1%	0.1	4276
LOC108349001	0.0%	0.1%	0.1	4277
Fam105a	0.0%	0.1%	0.1	4278
Cdc14b	0.0%	0.1%	0.1	4279
LOC684828	0.0%	0.1%	0.1	4280
LOC103693287	0.0%	0.1%	0.1	4281
LOC102551279	0.0%	0.1%	0.1	4282
LOC103692745	0.0%	0.1%	0.1	4283
Prr11	0.0%	0.1%	0.1	4284

Bambi	0.0%	0.1%	0.1	4285
LOC103692928	0.0%	0.1%	0.1	4286
LOC102551633	0.0%	0.1%	0.1	4287
LOC100912459	0.0%	0.1%	0.1	4288
LOC103693134	0.0%	0.1%	0.1	4289
LOC688442	0.0%	0.1%	0.1	4290
Plau	0.0%	0.1%	0.1	4291
Duox1	0.0%	0.1%	0.1	4292
Mpl	0.0%	0.1%	0.1	4293
Spats2l	0.0%	0.1%	0.1	4294
Acsm4	0.0%	0.1%	0.1	4295
LOC100910200	0.0%	0.1%	0.1	4296
LOC108353216	0.0%	0.1%	0.1	4297
LOC100912615	0.0%	0.1%	0.1	4298
Col9a2	0.0%	0.1%	0.1	4299
Sh3d21	0.0%	0.1%	0.1	4300
LOC108352137	0.0%	0.1%	0.1	4301
Synb	0.0%	0.1%	0.1	4302
RGD1308117	0.0%	0.1%	0.1	4303
LOC103692980	0.0%	0.1%	0.1	4304
Dupd1	0.0%	0.1%	0.1	4305
Fam166a	0.0%	0.1%	0.1	4306
Sfrp5	0.0%	0.1%	0.1	4307
LOC102556076	0.0%	0.1%	0.1	4308
Chst11	0.0%	0.1%	0.1	4309
LOC102553583	0.0%	0.1%	0.1	4310
Prickle2	0.0%	0.1%	0.1	4311
LOC103693133	0.0%	0.1%	0.1	4312
Nhs	0.0%	0.1%	0.1	4313
Gpr39	0.0%	0.1%	0.1	4314
Sox17	0.0%	0.1%	0.1	4315
LOC108352448	0.0%	0.1%	0.1	4316
Katnal1	0.0%	0.1%	0.1	4317
Fgfbp3	0.0%	0.1%	0.1	4318
Dnah1	0.0%	0.1%	0.1	4319
Tas1r3	0.0%	0.1%	0.1	4320
Ache	0.0%	0.1%	0.1	4321
Cdh3	0.0%	0.1%	0.1	4322
LOC100359930	0.0%	0.1%	0.1	4323
LOC499565	0.0%	0.1%	0.1	4324
Smpx	0.0%	0.1%	0.1	4325
Lrriq3	0.0%	0.1%	0.1	4326
LOC100910986	0.0%	0.1%	0.1	4327
LOC100910483	0.0%	0.1%	0.1	4328
LOC108349363	0.0%	0.1%	0.1	4329
LOC103690035	0.0%	0.1%	0.1	4330
LOC102552284	0.0%	0.1%	0.1	4331
Ch25h	0.0%	0.1%	0.1	4332
Runx2	0.0%	0.1%	0.1	4333
Il6	0.0%	0.1%	0.1	4334
LOC103695117	0.0%	0.1%	0.1	4335
LOC100909595	0.0%	0.1%	0.1	4336
RGD1563818	0.0%	0.1%	0.1	4337
Acod1	0.0%	0.1%	0.1	4338
Il36rn	0.0%	0.1%	0.1	4339
Lmod1	0.0%	0.1%	0.1	4340
LOC102550369	0.0%	0.1%	0.1	4341
LOC501599	0.0%	0.1%	0.1	4342
LOC108350623	0.0%	0.1%	0.1	4343
LOC100912564	0.0%	0.1%	0.1	4344
LOC100912596	0.0%	0.1%	0.1	4345
Foxc1	0.0%	0.1%	0.1	4346
Plcd4	0.0%	0.1%	0.1	4347
RGD1566006	0.0%	0.1%	0.1	4348
Hspa2	0.0%	0.1%	0.1	4349
Trim35	0.0%	0.1%	0.1	4350

Hjurp	0.0%	0.1%	0.1	4351
LOC102556859	0.0%	0.1%	0.1	4352
Rbp2	0.0%	0.1%	0.1	4353
LOC108349562	0.0%	0.1%	0.1	4354
Mamdc4	0.0%	0.1%	0.1	4355
Fam78a	0.0%	0.1%	0.1	4356
LOC102557581	0.0%	0.1%	0.1	4357
LOC100911572	0.0%	0.1%	0.1	4358
Loxl1	0.0%	0.1%	0.1	4359
LOC102547956	0.0%	0.1%	0.1	4360
Sgpp2	0.0%	0.1%	0.1	4361
Zfp526	0.0%	0.1%	0.1	4362
LOC102554207	0.0%	0.1%	0.1	4363
LOC108352166	0.0%	0.1%	0.1	4364
LOC102551324	0.0%	0.1%	0.1	4365
LOC108351414	0.0%	0.1%	0.1	4366
Tnfsf13b	0.0%	0.1%	0.1	4367
Igf1r	0.0%	0.1%	0.1	4368
LOC102547478	0.0%	0.1%	0.1	4369
LOC102556662	0.0%	0.1%	0.1	4370
Eml6	0.0%	0.1%	0.1	4371
LOC102553664	0.0%	0.1%	0.1	4372
Tgfb2	0.0%	0.1%	0.1	4373
LOC108351663	0.0%	0.1%	0.1	4374
LOC498205	0.0%	0.1%	0.1	4375
LOC102549869	0.0%	0.1%	0.1	4376
Hoxc4	0.0%	0.1%	0.1	4377
Fam167a	0.0%	0.1%	0.1	4378
Csrnp3	0.0%	0.1%	0.1	4379
LOC102555554	0.0%	0.1%	0.1	4380
Gal3st3	0.0%	0.1%	0.1	4381
LOC100910134	0.0%	0.1%	0.1	4382
Ctrl	0.0%	0.1%	0.1	4383
Frs3	0.0%	0.1%	0.1	4384
Ly6al	0.0%	0.1%	0.1	4385
LOC102549532	0.0%	0.1%	0.1	4386
Bcl9	0.0%	0.1%	0.1	4387
Lama2	0.0%	0.1%	0.1	4388
Slc2a6	0.0%	0.1%	0.1	4389
LOC102546403	0.0%	0.1%	0.1	4390
Cabs1	0.0%	0.1%	0.1	4391
LOC361346	0.0%	0.1%	0.1	4392
LOC100909490	0.0%	0.1%	0.1	4393
Nbea	0.0%	0.1%	0.1	4394
LOC103691879	0.0%	0.1%	0.1	4395
Osm	0.0%	0.1%	0.1	4396
Ush2a	0.0%	0.1%	0.1	4397
Pla2g4c	0.0%	0.1%	0.1	4398
Csf2rb	0.0%	0.1%	0.1	4399
Tubg2	0.0%	0.1%	0.1	4400
LOC103693998	0.0%	0.1%	0.1	4401
Myo9a	0.0%	0.1%	0.1	4402
Itgb1bp2	0.0%	0.1%	0.1	4403
Mapk13	0.0%	0.1%	0.1	4404
Cxxc4	0.0%	0.1%	0.1	4405
LOC108348254	0.0%	0.1%	0.1	4406
Cfap70	0.0%	0.1%	0.1	4407
LOC102554503	0.0%	0.1%	0.1	4408
RGD1310081	0.0%	0.1%	0.1	4409
Ptgdr2	0.0%	0.1%	0.1	4410
RGD1559747	0.0%	0.1%	0.1	4411
Serpinb9d	0.0%	0.1%	0.1	4412
Ophn1	0.0%	0.1%	0.1	4413
Begain	0.0%	0.1%	0.1	4414
Creb5	0.0%	0.1%	0.1	4415
Esrp1	0.0%	0.1%	0.1	4416

Pamr1	0.0%	0.1%	0.1	4417
Arhgef10	0.0%	0.1%	0.1	4418
Syde1	0.0%	0.1%	0.1	4419
LOC316717	0.0%	0.1%	0.1	4420
Rps6ka2	0.0%	0.1%	0.1	4421
Cdca5	0.0%	0.1%	0.1	4422
Pcdhga1	0.0%	0.1%	0.1	4423
Zfp870	0.0%	0.1%	0.1	4424
Nes	0.0%	0.1%	0.1	4425
LOC102547942	0.0%	0.1%	0.1	4426
LOC102548944	0.0%	0.1%	0.1	4427
LOC108352344	0.0%	0.1%	0.1	4428
LOC108351462	0.0%	0.1%	0.1	4429
LOC102552667	0.0%	0.1%	0.1	4430
Runx3	0.0%	0.1%	0.1	4431
Btnl5	0.0%	0.1%	0.1	4432
Tmem132e	0.0%	0.1%	0.1	4433
Nexn	0.0%	0.1%	0.1	4434
RGD1565806	0.0%	0.1%	0.1	4435
Raver2	0.0%	0.1%	0.1	4436
LOC103692249	0.0%	0.1%	0.1	4437
LOC687130	0.0%	0.1%	0.1	4438
Wls	0.0%	0.1%	0.1	4439
Csf3	0.0%	0.1%	0.1	4440
LOC103691261	0.0%	0.1%	0.1	4441
Cecr6	0.0%	0.1%	0.1	4442
LOC102554654	0.0%	0.1%	0.1	4443
Kit	0.0%	0.1%	0.1	4444
LOC108353477	0.0%	0.1%	0.1	4445
LOC688765	0.0%	0.1%	0.1	4446
Ahnak2	0.0%	0.1%	0.1	4447
Gzmn	0.0%	0.1%	0.1	4448
Garem1	0.0%	0.1%	0.1	4449
LOC100911166	0.0%	0.1%	0.1	4450
LOC102554492	0.0%	0.1%	0.1	4451
LOC102550128	0.0%	0.1%	0.1	4452
Nanog	0.0%	0.1%	0.1	4453
LOC108348158	0.0%	0.1%	0.1	4454
Gykl1	0.0%	0.1%	0.1	4455
LOC103691041	0.0%	0.1%	0.1	4456
LOC108349925	0.0%	0.1%	0.1	4457
Adgre4	0.0%	0.1%	0.1	4458
Dysf	0.0%	0.1%	0.1	4459
Rinl	0.0%	0.1%	0.1	4460
Plekha2	0.0%	0.1%	0.1	4461
Kif18b	0.0%	0.1%	0.1	4462
Myof	0.0%	0.1%	0.1	4463
Krt4	0.0%	0.1%	0.1	4464
Myct1	0.0%	0.1%	0.1	4465
LOC108349352	0.0%	0.1%	0.1	4466
Mgat5	0.0%	0.1%	0.1	4467
Rgs9	0.0%	0.1%	0.1	4468
LOC100364958	0.0%	0.1%	0.1	4469
LOC102554670	0.0%	0.1%	0.1	4470
LOC102548698	0.0%	0.1%	0.1	4471
LOC102552003	0.0%	0.1%	0.1	4472
LOC102550864	0.0%	0.1%	0.1	4473
Styxl1	0.0%	0.1%	0.1	4474
LOC102547798	0.0%	0.1%	0.1	4475
LOC103692626	0.0%	0.1%	0.1	4476
LOC108348838	0.0%	0.1%	0.1	4477
Pcdha4	0.0%	0.1%	0.1	4478
LOC103691490	0.0%	0.1%	0.1	4479
LOC103693804	0.0%	0.1%	0.1	4480
RGD1561551	0.0%	0.1%	0.1	4481
Il34	0.0%	0.1%	0.1	4482

Ankmy1	0.0%	0.1%	0.1	4483
Vwa7	0.0%	0.1%	0.1	4484
LOC102554810	0.0%	0.1%	0.1	4485
LOC100909485	0.0%	0.1%	0.1	4486
H2afy2	0.0%	0.1%	0.1	4487
Fam115c	0.0%	0.1%	0.1	4488
LOC103694538	0.0%	0.1%	0.1	4489
LOC108351082	0.0%	0.1%	0.1	4490
Zfp93	0.0%	0.1%	0.1	4491
LOC102548303	0.0%	0.1%	0.1	4492
Mkx	0.0%	0.1%	0.1	4493
LOC108349555	0.0%	0.1%	0.1	4494
Slc29a3	0.0%	0.1%	0.1	4495
LOC103690191	0.0%	0.1%	0.1	4496
Rsph1	0.0%	0.1%	0.1	4497
LOC108349354	0.0%	0.1%	0.1	4498
Tox	0.0%	0.1%	0.1	4499
Slc25a35	0.0%	0.1%	0.1	4500
Cdk14	0.0%	0.1%	0.1	4501
LOC102549500	0.0%	0.1%	0.1	4502
LOC102550455	0.0%	0.1%	0.1	4503
LOC102546596	0.0%	0.1%	0.1	4504
Crabp2	0.0%	0.1%	0.1	4505
LOC102555557	0.0%	0.1%	0.1	4506
LOC102549300	0.0%	0.1%	0.1	4507
Siglec8	0.0%	0.1%	0.1	4508
LOC681355	0.0%	0.1%	0.1	4509
Capsl	0.0%	0.1%	0.1	4510
LOC103690131	0.0%	0.1%	0.1	4511
Cd209b	0.0%	0.1%	0.1	4512
Slc7a12	0.0%	0.1%	0.1	4513
Rasal1	0.0%	0.1%	0.1	4514
Nrros	0.0%	0.1%	0.1	4515
LOC690126	0.0%	0.1%	0.1	4516
LOC108349824	0.0%	0.1%	0.1	4517
LOC102547275	0.0%	0.1%	0.1	4518
Ctla4	0.0%	0.1%	0.1	4519
LOC103691460	0.0%	0.1%	0.1	4520
Ccr1	0.0%	0.1%	0.1	4521
LOC102554307	0.0%	0.1%	0.1	4522
LOC102554294	0.0%	0.1%	0.1	4523
Timeless	0.0%	0.1%	0.1	4524
LOC691592	0.0%	0.1%	0.1	4525
LOC100912084	0.0%	0.1%	0.1	4526
LOC103694069	0.0%	0.1%	0.1	4527
LOC102554576	0.0%	0.1%	0.1	4528
LOC102548205	0.0%	0.1%	0.1	4529
LOC102555659	0.0%	0.1%	0.1	4530
LOC100912611	0.0%	0.1%	0.1	4531
LOC100911949	0.0%	0.1%	0.1	4532
LOC102547852	0.0%	0.1%	0.1	4533
Filip1	0.0%	0.1%	0.1	4534
LOC102547563	0.0%	0.1%	0.1	4535
LOC108352129	0.0%	0.1%	0.1	4536
LOC100910838	0.0%	0.1%	0.1	4537
LOC102547651	0.0%	0.1%	0.1	4538
Vipr1	0.0%	0.1%	0.1	4539
LOC108350792	0.0%	0.1%	0.1	4540
LOC102554605	0.0%	0.1%	0.1	4541
Ns5atp4	0.0%	0.1%	0.1	4542
LOC102550845	0.0%	0.1%	0.1	4543
LOC108351987	0.0%	0.1%	0.1	4544
P3h3	0.0%	0.1%	0.1	4545
LOC108348294	0.0%	0.1%	0.1	4546
LOC103695191	0.0%	0.1%	0.1	4547
Ttll9	0.0%	0.1%	0.1	4548

Neil2	0.0%	0.1%	0.1	4549
P2ry14	0.0%	0.1%	0.1	4550
LOC108349071	0.0%	0.1%	0.1	4551
LOC103691422	0.0%	0.1%	0.1	4552
LOC108351171	0.0%	0.1%	0.1	4553
Plcd1	0.0%	0.1%	0.1	4554
Plekha7	0.0%	0.1%	0.1	4555
Hecw2	0.0%	0.1%	0.1	4556
LOC102551795	0.0%	0.1%	0.1	4557
Ogfod1	0.0%	0.1%	0.1	4558
Adgrg6	0.0%	0.1%	0.1	4559
LOC102550940	0.0%	0.1%	0.1	4560
LOC108349324	0.0%	0.1%	0.1	4561
Pdilt	0.0%	0.1%	0.1	4562
Rbl1	0.0%	0.1%	0.1	4563
Exoc8	0.0%	0.1%	0.1	4564
LOC103694852	0.0%	0.1%	0.1	4565
Jade2	0.0%	0.1%	0.1	4566
Tspan17	0.0%	0.1%	0.1	4567
Dcaf12	0.0%	0.1%	0.1	4568
Fto	0.0%	0.1%	0.1	4569
LOC100912428	0.0%	0.1%	0.1	4570
LOC102554082	0.0%	0.1%	0.1	4571
Plppr4	0.0%	0.1%	0.1	4572
LOC680227	0.0%	0.1%	0.1	4573
Fstl4	0.0%	0.1%	0.1	4574
Olr1351	0.0%	0.1%	0.1	4575
LOC102551377	0.0%	0.1%	0.1	4576
LOC102552776	0.0%	0.1%	0.1	4577
Pou4f1	0.0%	0.1%	0.1	4578
Dscc1	0.0%	0.1%	0.1	4579
Mrap2	0.0%	0.1%	0.1	4580
Lhfpl2	0.0%	0.1%	0.1	4581
Dse	0.0%	0.1%	0.1	4582
Zbtb32	0.0%	0.1%	0.1	4583
Sert1	0.0%	0.1%	0.1	4584
Cdc7	0.0%	0.1%	0.1	4585
Fat4	0.0%	0.1%	0.1	4586
LOC102546414	0.0%	0.1%	0.1	4587
Pla2r1	0.0%	0.1%	0.1	4588
LOC102552170	0.0%	0.1%	0.1	4589
LOC108353474	0.0%	0.1%	0.1	4590
LOC498300	0.0%	0.1%	0.1	4591
LOC500877	0.0%	0.1%	0.1	4592
LOC108350125	0.0%	0.1%	0.1	4593
LOC108351708	0.0%	0.1%	0.1	4594
LOC100911299	0.0%	0.1%	0.1	4595
Ccl6	0.0%	0.1%	0.1	4596
Ccdc88b	0.0%	0.1%	0.1	4597
Prex1	0.0%	0.1%	0.1	4598
LOC102556981	0.0%	0.1%	0.1	4599
LOC100362965	0.0%	0.1%	0.1	4600
LOC103695268	0.0%	0.1%	0.1	4601
Tmem246	0.0%	0.1%	0.1	4602
LOC108350776	0.0%	0.1%	0.1	4603
Rap1gap	0.0%	0.1%	0.1	4604
Slc5a1	0.0%	0.1%	0.1	4605
Wfdc11	0.0%	0.1%	0.1	4606
Zswim5	0.0%	0.1%	0.1	4607
LOC683430	0.0%	0.1%	0.1	4608
Cfap44	0.0%	0.1%	0.1	4609
LOC100911733	0.0%	0.1%	0.1	4610
LOC102553617	0.0%	0.1%	0.1	4611
LOC103692951	0.0%	0.1%	0.1	4612
LOC500594	0.0%	0.1%	0.1	4613
Muc13	0.0%	0.1%	0.1	4614

Wnt5b	0.0%	0.1%	0.1	4615
LOC103694969	0.0%	0.1%	0.1	4616
Tnnt3	0.0%	0.1%	0.1	4617
LOC108353009	0.0%	0.1%	0.1	4618
Csmd3	0.0%	0.1%	0.1	4619
LOC100362023	0.0%	0.1%	0.1	4620
RGD1559545	0.0%	0.1%	0.1	4621
Dnajc27	0.0%	0.1%	0.1	4622
Zfr2	0.0%	0.1%	0.1	4623
LOC108351734	0.0%	0.1%	0.1	4624
LOC102547817	0.0%	0.1%	0.1	4625
Xlr3a	0.0%	0.1%	0.1	4626
LOC108349444	0.0%	0.1%	0.1	4627
LOC102547720	0.0%	0.1%	0.1	4628
LOC108351867	0.0%	0.1%	0.1	4629
LOC100911251	0.0%	0.1%	0.1	4630
LOC100360453	0.0%	0.1%	0.1	4631
Pmaip1	0.0%	0.1%	0.1	4632
LOC501346	0.0%	0.1%	0.1	4633
C1H19orf84	0.0%	0.1%	0.1	4634
Cfap161	0.0%	0.1%	0.1	4635
Rad51ap1	0.0%	0.1%	0.1	4636
Nek2l1	0.0%	0.1%	0.1	4637
Thbs2	0.0%	0.1%	0.1	4638
Kcnk7	0.0%	0.1%	0.1	4639
LOC100909794	0.0%	0.1%	0.1	4640
LOC100911537	0.0%	0.1%	0.1	4641
Dtl	0.0%	0.1%	0.1	4642
Ntrk2	0.0%	0.1%	0.1	4643
LOC103690044	0.0%	0.1%	0.1	4644
Dkk1	0.0%	0.1%	0.1	4645
LOC102553881	0.0%	0.1%	0.1	4646
Cep112	0.0%	0.1%	0.1	4647
LOC108352356	0.0%	0.1%	0.1	4648
Mmp16	0.0%	0.1%	0.1	4649
Taf1b	0.0%	0.1%	0.1	4650
Arhgap11a	0.0%	0.1%	0.1	4651
LOC103692427	0.0%	0.1%	0.1	4652
LOC103694475	0.0%	0.1%	0.1	4653
LOC102551249	0.0%	0.1%	0.1	4654
Klra5	0.0%	0.1%	0.1	4655
Osbpl6	0.0%	0.1%	0.1	4656
Dnm1	0.0%	0.1%	0.1	4657
LOC102549235	0.0%	0.1%	0.1	4658
Dph6	0.0%	0.1%	0.1	4659
LOC108349595	0.0%	0.1%	0.1	4660
LOC102547293	0.0%	0.1%	0.1	4661
LOC103691594	0.0%	0.1%	0.1	4662
LOC108349637	0.0%	0.1%	0.1	4663
Klhl13	0.0%	0.1%	0.1	4664
Bcam	0.0%	0.1%	0.1	4665
LOC691520	0.0%	0.1%	0.1	4666
Ltbp3	0.0%	0.1%	0.1	4667
Cftr	0.0%	0.1%	0.1	4668
Fcgbp	0.0%	0.1%	0.1	4669
Zfat	0.0%	0.1%	0.1	4670
LOC102551408	0.0%	0.1%	0.1	4671
Sfrp1	0.0%	0.1%	0.1	4672
LOC100360690	0.0%	0.1%	0.1	4673
Ccdc57	0.0%	0.1%	0.1	4674
Cryga	0.0%	0.1%	0.1	4675
Wfikkn1	0.0%	0.1%	0.1	4676
Synpo2	0.0%	0.1%	0.1	4677
LOC103692460	0.0%	0.1%	0.1	4678
Grap2	0.0%	0.1%	0.1	4679
LOC102547975	0.0%	0.1%	0.1	4680

LOC103693210	0.0%	0.1%	0.1	4681
LOC108350109	0.0%	0.1%	0.1	4682
Pou6f2	0.0%	0.1%	0.1	4683
Clec4d	0.0%	0.1%	0.1	4684
Arhgap28	0.0%	0.1%	0.1	4685
LOC102553722	0.0%	0.1%	0.1	4686
LOC102551286	0.0%	0.1%	0.1	4687
LOC102553361	0.0%	0.1%	0.1	4688
Cited1	0.0%	0.1%	0.1	4689
Prosc	0.0%	0.1%	0.1	4690
LOC103694331	0.0%	0.1%	0.1	4691
Xrcc3	0.0%	0.1%	0.1	4692
Ryr3	0.0%	0.1%	0.1	4693
Stk39	0.0%	0.1%	0.1	4694
Tcaf2	0.0%	0.1%	0.1	4695
Ifitm5	0.0%	0.1%	0.1	4696
LOC102554575	0.0%	0.1%	0.1	4697
Slc9a7	0.0%	0.1%	0.1	4698
LOC102547954	0.0%	0.1%	0.1	4699
LOC108351606	0.0%	0.1%	0.1	4700
Ms4a1	0.0%	0.1%	0.1	4701
Naip6	0.0%	0.1%	0.1	4702
Lrr1	0.0%	0.1%	0.1	4703
LOC108349558	0.0%	0.1%	0.1	4704
Ptpro	0.0%	0.1%	0.1	4705
Rsph3	0.0%	0.1%	0.1	4706
Cyld	0.0%	0.1%	0.1	4707
Fam174b	0.0%	0.1%	0.1	4708
LOC684792	0.0%	0.1%	0.1	4709
Oosp1	0.0%	0.1%	0.1	4710
Itga3	0.0%	0.1%	0.1	4711
Kctd11	0.0%	0.1%	0.1	4712
Rab25	0.0%	0.1%	0.1	4713
Samd7	0.0%	0.1%	0.1	4714
RGD1559588	0.0%	0.1%	0.1	4715
LOC102548047	0.0%	0.1%	0.1	4716
LOC102548728	0.0%	0.1%	0.1	4717
LOC102552496	0.0%	0.1%	0.1	4718
Lef1	0.0%	0.1%	0.1	4719
Ifitm6	0.0%	0.1%	0.1	4720
LOC102556075	0.0%	0.1%	0.1	4721
Gpr52	0.0%	0.1%	0.1	4722
Raet1e	0.0%	0.1%	0.1	4723
LOC365839	0.0%	0.1%	0.1	4724
LOC691320	0.0%	0.1%	0.1	4725
Cd52	0.0%	0.1%	0.1	4726
LOC103693794	0.0%	0.1%	0.1	4727
Dab2	0.0%	0.1%	0.1	4728
Lrrc39	0.0%	0.1%	0.1	4729
Actl7b	0.0%	0.1%	0.1	4730
RGD1306271	0.0%	0.1%	0.1	4731
Ccdc69	0.0%	0.1%	0.1	4732
LOC102551847	0.0%	0.1%	0.1	4733
LOC103690170	0.0%	0.1%	0.1	4734
LOC103691225	0.0%	0.1%	0.1	4735
LOC102546547	0.0%	0.1%	0.1	4736
LOC100361098	0.0%	0.1%	0.1	4737
LOC103690474	0.0%	0.1%	0.1	4738
LOC103691059	0.0%	0.1%	0.1	4739
LOC100910525	0.0%	0.1%	0.1	4740
Cdhr2	0.0%	0.1%	0.1	4741
LOC100909715	0.0%	0.1%	0.1	4742
LOC498222	0.0%	0.1%	0.1	4743
Plk1	0.0%	0.1%	0.1	4744
LOC102547703	0.0%	0.1%	0.1	4745
Tmc8	0.0%	0.1%	0.1	4746

Rnasel	0.0%	0.1%	0.1	4747
LOC103689937	0.0%	0.1%	0.1	4748
LOC499823	0.0%	0.1%	0.1	4749
Vwce	0.0%	0.1%	0.1	4750
Lin7c	0.0%	0.1%	0.1	4751
LOC685152	0.0%	0.1%	0.1	4752
LOC102555754	0.0%	0.1%	0.1	4753
LOC102549299	0.0%	0.1%	0.1	4754
LOC102550090	0.0%	0.1%	0.1	4755
Slc12a5	0.0%	0.1%	0.1	4756
LOC103690204	0.0%	0.1%	0.1	4757
LOC102552423	0.0%	0.1%	0.1	4758
RGD1562890	0.0%	0.1%	0.1	4759
Plpp4	0.0%	0.1%	0.1	4760
Brca1	0.0%	0.1%	0.1	4761
LOC102546345	0.0%	0.1%	0.1	4762
Slc37a1	0.0%	0.1%	0.1	4763
LOC685668	0.0%	0.1%	0.1	4764
Siglec1	0.0%	0.1%	0.1	4765
LOC102552101	0.0%	0.1%	0.1	4766
E2f8	0.0%	0.1%	0.1	4767
Fads3	0.0%	0.1%	0.1	4768
LOC102550277	0.0%	0.1%	0.1	4769
Prdm8	0.0%	0.1%	0.1	4770
LOC102554202	0.0%	0.1%	0.1	4771
Hist1h2ai	0.0%	0.1%	0.1	4772
LOC103694115	0.0%	0.1%	0.1	4773
Pdcd1lg2	0.0%	0.1%	0.1	4774
LOC100910033	0.0%	0.1%	0.1	4775
Fam228b	0.0%	0.1%	0.1	4776
Vom1r42	0.0%	0.1%	0.1	4777
Zc2hc1b	0.0%	0.1%	0.1	4778
Jpx	0.0%	0.1%	0.1	4779
Wnt2b	0.0%	0.1%	0.1	4780
LOC108352526	0.0%	0.1%	0.1	4781
Gpr35	0.0%	0.1%	0.1	4782
LOC102551151	0.0%	0.1%	0.1	4783
Ddo	0.0%	0.1%	0.1	4784
Nod2	0.0%	0.1%	0.1	4785
Amz1	0.0%	0.1%	0.1	4786
Tgfb3	0.0%	0.1%	0.1	4787
LOC108349046	0.0%	0.1%	0.1	4788
LOC103694853	0.0%	0.1%	0.1	4789
LOC103690128	0.0%	0.1%	0.1	4790
LOC108350081	0.0%	0.1%	0.1	4791
Prdm16	0.0%	0.1%	0.1	4792
Pxt1	0.0%	0.1%	0.1	4793
LOC102554488	0.0%	0.1%	0.1	4794
LOC102549406	0.0%	0.1%	0.1	4795
LOC103693908	0.0%	0.1%	0.1	4796
Tmem26	0.0%	0.1%	0.1	4797
LOC100912489	0.0%	0.1%	0.1	4798
RGD1559578	0.0%	0.1%	0.1	4799
RGD1561306	0.0%	0.1%	0.1	4800
LOC102556339	0.0%	0.1%	0.1	4801
Mastl	0.0%	0.1%	0.1	4802
Mroh8	0.0%	0.1%	0.1	4803
LOC102548734	0.0%	0.1%	0.1	4804
LOC100909671	0.0%	0.1%	0.1	4805
Tbc1d19	0.0%	0.1%	0.1	4806
Pofut1	0.0%	0.1%	0.1	4807
Spata21	0.0%	0.1%	0.1	4808
Fam110d	0.0%	0.1%	0.1	4809
LOC100361083	0.0%	0.1%	0.1	4810
LOC108348390	0.0%	0.1%	0.1	4811
Cep72	0.0%	0.1%	0.1	4812

Zbtb46	0.0%	0.1%	0.1	4813
B3gnt8	0.0%	0.1%	0.1	4814
LOC100911365	0.0%	0.1%	0.1	4815
Arl6ip5	0.0%	0.1%	0.1	4816
RT1-N1	0.0%	0.1%	0.1	4817
Rsph10b	0.0%	0.1%	0.1	4818
Syngr2	0.0%	0.1%	0.1	4819
LOC108353594	0.0%	0.1%	0.1	4820
LOC500213	0.0%	0.1%	0.1	4821
LOC103693073	0.0%	0.1%	0.1	4822
LOC108350373	0.0%	0.1%	0.1	4823
Gpr34	0.0%	0.1%	0.1	4824
Ngp	0.0%	0.1%	0.1	4825
Nav3	0.0%	0.1%	0.1	4826
LOC103695099	0.0%	0.1%	0.1	4827
Bhlhe41	0.0%	0.1%	0.1	4828
LOC102548611	0.0%	0.1%	0.1	4829
LOC103694344	0.0%	0.1%	0.1	4830
Pla2g2f	0.0%	0.1%	0.1	4831
LOC102552970	0.0%	0.1%	0.1	4832
Pld2	0.0%	0.1%	0.1	4833
Sdc3	0.0%	0.1%	0.1	4834
Gab2	0.0%	0.1%	0.1	4835
Ms4a4a	0.0%	0.1%	0.1	4836
Mcpt8l2	0.0%	0.1%	0.1	4837
LOC108352426	0.0%	0.1%	0.1	4838
LOC102546355	0.0%	0.1%	0.1	4839
LOC100362122	0.0%	0.1%	0.1	4840
LOC292603	0.0%	0.1%	0.1	4841
RT1-CE6	0.0%	0.1%	0.1	4842
LOC683605	0.0%	0.1%	0.1	4843
Adamts5	0.0%	0.1%	0.1	4844
LOC100911216	0.0%	0.1%	0.1	4845
LOC102552166	0.0%	0.1%	0.1	4846
LOC108349464	0.0%	0.1%	0.1	4847
Csta	0.0%	0.1%	0.1	4848
Pdgfb	0.0%	0.1%	0.1	4849
LOC100911090	0.0%	0.1%	0.1	4850
LOC103690066	0.0%	0.1%	0.1	4851
Sec31b	0.0%	0.1%	0.1	4852
Sparcl1	0.0%	0.1%	0.1	4853
LOC102546629	0.0%	0.1%	0.1	4854
LOC503053	0.0%	0.1%	0.1	4855
LOC102555425	0.0%	0.1%	0.1	4856
Slc22a24	0.0%	0.1%	0.1	4857
Tbr1	0.0%	0.1%	0.1	4858
Ankrd6	0.0%	0.1%	0.1	4859
LOC501223	0.0%	0.1%	0.1	4860
LOC108348578	0.0%	0.1%	0.1	4861
Cit	0.0%	0.1%	0.1	4862
Map7d2	0.0%	0.1%	0.1	4863
Slc12a8	0.0%	0.1%	0.1	4864
Ldoc1	0.0%	0.1%	0.1	4865
Dync2h1	0.0%	0.1%	0.1	4866
Scara3	0.0%	0.1%	0.1	4867
Trove2	0.0%	0.1%	0.1	4868
LOC102546754	0.0%	0.1%	0.1	4869
LOC102552550	0.0%	0.1%	0.1	4870
Fgd2	0.0%	0.1%	0.1	4871
Ret	0.0%	0.1%	0.1	4872
LOC102547847	0.0%	0.1%	0.1	4873
Gpr84	0.0%	0.1%	0.1	4874
Spef2	0.0%	0.1%	0.1	4875
Yod1	0.0%	0.1%	0.1	4876
LOC100909582	0.0%	0.1%	0.1	4877
LOC108352027	0.0%	0.1%	0.1	4878

LOC108352702	0.0%	0.1%	0.1	4879
RGD1563222	0.0%	0.1%	0.1	4880
LOC108352013	0.0%	0.1%	0.1	4881
LOC102552397	0.0%	0.1%	0.1	4882
LOC103690030	0.0%	0.1%	0.1	4883
Bicc1	0.0%	0.1%	0.1	4884
LOC103691222	0.0%	0.1%	0.1	4885
Iqck	0.0%	0.1%	0.1	4886
LOC100911379	0.0%	0.1%	0.1	4887
Spp1	0.0%	0.1%	0.1	4888
LOC108350763	0.0%	0.1%	0.1	4889
Gcnt3	0.0%	0.1%	0.1	4890
LOC103693611	0.0%	0.1%	0.1	4891
Hoxb3	0.0%	0.1%	0.1	4892
Tmem63c	0.0%	0.1%	0.1	4893
Trpv1	0.0%	0.1%	0.1	4894
Tmem173	0.0%	0.1%	0.1	4895
Kcnk6	0.0%	0.1%	0.1	4896
Tp63	0.0%	0.1%	0.1	4897
LOC102550304	0.0%	0.1%	0.1	4898
LOC102555010	0.0%	0.1%	0.1	4899
Mapk15	0.0%	0.1%	0.1	4900
Mettl24	0.0%	0.1%	0.1	4901
RGD1561931	0.0%	0.1%	0.1	4902
Cd200	0.0%	0.1%	0.1	4903
LOC102555972	0.0%	0.1%	0.1	4904
Trem1	0.0%	0.1%	0.1	4905
LOC685048	0.0%	0.1%	0.1	4906
Ggt7	0.0%	0.1%	0.1	4907
RGD1565622	0.0%	0.1%	0.1	4908
LOC103693384	0.0%	0.1%	0.1	4909
LOC100909998	0.0%	0.1%	0.1	4910
Sema6d	0.0%	0.1%	0.1	4911
LOC108352465	0.0%	0.1%	0.1	4912
Foxj1	0.0%	0.1%	0.1	4913
Ndc80	0.0%	0.1%	0.1	4914
Itgb6	0.0%	0.1%	0.1	4915
Flt3	0.0%	0.1%	0.1	4916
LOC100909964	0.0%	0.1%	0.1	4917
LOC108352917	0.0%	0.1%	0.1	4918
LOC102552712	0.0%	0.1%	0.1	4919
Lrrc58	0.0%	0.1%	0.1	4920
Best1	0.0%	0.1%	0.1	4921
LOC108348151	0.0%	0.1%	0.1	4922
LOC102549239	0.0%	0.1%	0.1	4923
LOC103694936	0.0%	0.1%	0.1	4924
LOC108349984	0.0%	0.1%	0.1	4925
Nap1l2	0.0%	0.1%	0.1	4926
Prok2	0.0%	0.1%	0.1	4927
Stab2	0.0%	0.1%	0.1	4928
LOC102547219	0.0%	0.1%	0.1	4929
LOC108349780	0.0%	0.1%	0.1	4930
Arpp21	0.0%	0.1%	0.1	4931
LOC103693591	0.0%	0.1%	0.1	4932
Ppp1r1b	0.0%	0.1%	0.1	4933
LOC691722	0.0%	0.1%	0.1	4934
F8	0.0%	0.1%	0.1	4935
Fndc5	0.0%	0.1%	0.1	4936
Pcdhga9	0.0%	0.1%	0.1	4937
Nrm	0.0%	0.1%	0.1	4938
Bdkrb2	0.0%	0.1%	0.1	4939
Mfsd4b	0.0%	0.1%	0.1	4940
LOC367117	0.0%	0.1%	0.1	4941
Hist3h2bb	0.0%	0.1%	0.1	4942
LOC108348402	0.0%	0.1%	0.1	4943
Cbarp	0.0%	0.1%	0.1	4944

LOC103695187	0.0%	0.1%	0.1	4945
RGD1311517	0.0%	0.1%	0.1	4946
LOC108350023	0.0%	0.1%	0.1	4947
Rac3	0.0%	0.1%	0.1	4948
Ak7	0.0%	0.1%	0.1	4949
LOC103695124	0.0%	0.1%	0.1	4950
Frmd5	0.0%	0.1%	0.1	4951
LOC102554487	0.0%	0.1%	0.1	4952
Carmil2	0.0%	0.1%	0.1	4953
Lrrcc1	0.0%	0.1%	0.1	4954
Adcy4	0.0%	0.1%	0.1	4955
LOC108348366	0.0%	0.1%	0.1	4956
LOC103693147	0.0%	0.1%	0.1	4957
Mex3a	0.0%	0.1%	0.1	4958
RT1-M5	0.0%	0.1%	0.1	4959
LOC102556426	0.0%	0.1%	0.1	4960
Tal1	0.0%	0.1%	0.1	4961
LOC102555823	0.0%	0.1%	0.1	4962
LOC103692516	0.0%	0.1%	0.1	4963
RGD1561755	0.0%	0.1%	0.1	4964
Cyp1b1	0.0%	0.1%	0.1	4965
Btla	0.0%	0.1%	0.1	4966
Rad9b	0.0%	0.1%	0.1	4967
Ly49i5	0.0%	0.1%	0.1	4968
Frzb	0.0%	0.1%	0.1	4969
Hist1h2an	0.0%	0.1%	0.1	4970
Igfbp3	0.0%	0.1%	0.1	4971
Mcph1	0.0%	0.1%	0.1	4972
LOC108352189	0.0%	0.1%	0.1	4973
Stxbp5l	0.0%	0.1%	0.1	4974
LOC108350799	0.0%	0.1%	0.1	4975
LOC108349966	0.0%	0.1%	0.1	4976
Rnase1l2	0.0%	0.1%	0.1	4977
Spetex-2C	0.0%	0.1%	0.1	4978
LOC108352840	0.0%	0.1%	0.1	4979
Tex13b	0.0%	0.1%	0.1	4980
LOC102552044	0.0%	0.1%	0.1	4981
Irf2bpl	0.0%	0.1%	0.1	4982
LOC103690084	0.0%	0.1%	0.1	4983
LOC100910750	0.0%	0.1%	0.1	4984
Neu2	0.0%	0.1%	0.1	4985
Fam219a	0.0%	0.1%	0.1	4986
Ercc6l	0.0%	0.1%	0.1	4987
RGD1562521	0.0%	0.1%	0.1	4988
Theg	0.0%	0.1%	0.1	4989
Ccdc160	0.0%	0.1%	0.1	4990
Cpt1b	0.0%	0.1%	0.1	4991
LOC102555924	0.0%	0.1%	0.1	4992
LOC108349480	0.0%	0.1%	0.1	4993
LOC100362418	0.0%	0.1%	0.1	4994
Nudcd3	0.0%	0.1%	0.1	4995
Bbs12	0.0%	0.1%	0.1	4996
LOC102553897	0.0%	0.1%	0.1	4997
Stx1a	0.0%	0.1%	0.1	4998
Dctd	0.0%	0.1%	0.1	4999
Dclk1	0.0%	0.1%	0.1	5000
LOC108353708	0.0%	0.1%	0.1	5001
Pate2	0.0%	0.1%	0.1	5002
LOC102547300	0.0%	0.1%	0.1	5003
Cd164l2	0.0%	0.1%	0.1	5004
LOC302827	0.0%	0.1%	0.1	5005
LOC102549146	0.0%	0.1%	0.1	5006
LOC100360601	0.0%	0.1%	0.1	5007
LOC102555995	0.0%	0.1%	0.1	5008
Ubap1l	0.0%	0.1%	0.1	5009
Pnpla1	0.0%	0.1%	0.1	5010

LOC102555437	0.0%	0.1%	0.1	5011
LOC103694018	0.0%	0.1%	0.1	5012
LOC103690057	0.0%	0.1%	0.1	5013
Stra8	0.0%	0.1%	0.1	5014
Oc90	0.0%	0.1%	0.1	5015
Antxr1	0.0%	0.1%	0.1	5016
Ptpn7	0.0%	0.1%	0.1	5017
LOC102547612	0.0%	0.1%	0.1	5018
Tnfrsf17	0.0%	0.1%	0.1	5019
LOC300308	0.0%	0.1%	0.1	5020
Pgf	0.0%	0.1%	0.1	5021
Dnali1	0.0%	0.1%	0.1	5022
LOC108349435	0.0%	0.1%	0.1	5023
Pla2g2d	0.0%	0.1%	0.1	5024
Cd226	0.0%	0.1%	0.1	5025
LOC102550575	0.0%	0.1%	0.1	5026
LOC103690322	0.0%	0.1%	0.1	5027
LOC103691871	0.0%	0.1%	0.1	5028
LOC691135	0.0%	0.1%	0.1	5029
Clvs1	0.0%	0.1%	0.1	5030
Soga1	0.0%	0.1%	0.1	5031
LOC103693367	0.0%	0.1%	0.1	5032
LOC108348906	0.0%	0.1%	0.1	5033
Adcy10	0.0%	0.1%	0.1	5034
Arl11	0.0%	0.1%	0.1	5035
Gmpr	0.0%	0.1%	0.1	5036
LOC108353138	0.0%	0.1%	0.1	5037
Npffr1	0.0%	0.1%	0.1	5038
Eqtn	0.0%	0.1%	0.1	5039
Bean1	0.0%	0.1%	0.1	5040
Dbf4	0.0%	0.1%	0.1	5041
Bcl2l14	0.0%	0.1%	0.1	5042
LOC103691392	0.0%	0.1%	0.1	5043
LOC688812	0.0%	0.1%	0.1	5044
LOC108349086	0.0%	0.1%	0.1	5045
LOC108353709	0.0%	0.1%	0.1	5046
LOC108353255	0.0%	0.1%	0.1	5047
Myo5c	0.0%	0.1%	0.1	5048
Tnfrsf25	0.0%	0.1%	0.1	5049
Ston2	0.0%	0.1%	0.1	5050
LOC108352165	0.0%	0.1%	0.1	5051
LOC108351021	0.0%	0.1%	0.1	5052
LOC108349512	0.0%	0.1%	0.1	5053
Pddc1	0.0%	0.1%	0.1	5054
LOC103694987	0.0%	0.1%	0.1	5055
LOC108352515	0.0%	0.1%	0.1	5056
LOC108348380	0.0%	0.1%	0.1	5057
LOC685279	0.0%	0.1%	0.1	5058
Samd12	0.0%	0.1%	0.1	5059
Slc2a3	0.0%	0.1%	0.1	5060
LOC108349241	0.0%	0.1%	0.1	5061
LOC103690669	0.0%	0.1%	0.1	5062
Dmrta2	0.0%	0.1%	0.1	5063
LOC103695224	0.0%	0.1%	0.1	5064
LOC102548408	0.0%	0.1%	0.1	5065
Ecscr	0.0%	0.1%	0.1	5066
Hdgfrp3	0.0%	0.1%	0.1	5067
LOC100911855	0.0%	0.1%	0.1	5068
Rgl1	0.0%	0.1%	0.1	5069
Zfp780b-ps1	0.0%	0.1%	0.1	5070
Pou3f2	0.0%	0.1%	0.1	5071
LOC102551526	0.0%	0.1%	0.1	5072
Ralgapa2	0.0%	0.1%	0.1	5073
Fam71e1	0.0%	0.1%	0.1	5074
Wdr90	0.0%	0.1%	0.1	5075
LOC103690118	0.0%	0.1%	0.1	5076

Tmem117	0.0%	0.1%	0.1	5077
LOC102553586	0.0%	0.1%	0.1	5078
Cnga3	0.0%	0.1%	0.1	5079
Lmx1a	0.0%	0.1%	0.1	5080
LOC102549061	0.0%	0.1%	0.1	5081
LOC100912247	0.0%	0.1%	0.1	5082
LOC100909725	0.0%	0.1%	0.1	5083
Nid2	0.0%	0.1%	0.1	5084
Ovol2	0.0%	0.1%	0.1	5085
Mrap	0.0%	0.1%	0.1	5086
LOC103691914	0.0%	0.1%	0.1	5087
LOC103690244	0.0%	0.1%	0.1	5088
Gpr150	0.0%	0.1%	0.1	5089
LOC102552830	0.0%	0.1%	0.1	5090
Chi3l1	0.0%	0.1%	0.1	5091
LOC102550788	0.0%	0.1%	0.1	5092
LOC108352975	0.0%	0.1%	0.1	5093
Efcab7	0.0%	0.1%	0.1	5094
LOC108351870	0.0%	0.1%	0.1	5095
Diaph3	0.0%	0.1%	0.1	5096
Lman2l	0.0%	0.1%	0.1	5097
RGD1564053	0.0%	0.1%	0.1	5098
LOC686129	0.0%	0.1%	0.1	5099
Eif5a2	0.0%	0.1%	0.1	5100
Fam217a	0.0%	0.1%	0.1	5101
LOC102549755	0.0%	0.1%	0.1	5102
Srgap3	0.0%	0.1%	0.1	5103
Cdc20b	0.0%	0.1%	0.1	5104
Zfp74	0.0%	0.1%	0.1	5105
LOC103694530	0.0%	0.1%	0.1	5106
Lrp12	0.0%	0.1%	0.1	5107
LOC108348896	0.0%	0.1%	0.1	5108
Cnbd2	0.0%	0.1%	0.1	5109
RGD1563986	0.0%	0.1%	0.1	5110
Zmynd15	0.0%	0.1%	0.1	5111
Ccdc7	0.0%	0.1%	0.1	5112
Cyp27b1	0.0%	0.1%	0.1	5113
LOC102547271	0.0%	0.1%	0.1	5114
LOC102548272	0.0%	0.1%	0.1	5115
Cog6	0.0%	0.1%	0.1	5116
Poln	0.0%	0.1%	0.1	5117
RGD1565752	0.0%	0.1%	0.1	5118
LOC100911807	0.0%	0.1%	0.1	5119
Spt1	0.0%	0.1%	0.1	5120
LOC103692658	0.0%	0.1%	0.1	5121
LOC102550789	0.0%	0.1%	0.1	5122
LOC100362197	0.0%	0.1%	0.1	5123
Snai1	0.0%	0.1%	0.1	5124
Klrg2	0.0%	0.1%	0.1	5125
Cd209a	0.0%	0.1%	0.1	5126
Ldb3	0.0%	0.1%	0.1	5127
Optc	0.0%	0.1%	0.1	5128
Ffar2	0.0%	0.1%	0.1	5129
Sult4a1	0.0%	0.1%	0.1	5130
Rhox10	0.0%	0.1%	0.1	5131
LOC102549303	0.0%	0.1%	0.1	5132
Irf5	0.0%	0.1%	0.1	5133
Tmem74b	0.0%	0.1%	0.1	5134
Sctr	0.0%	0.1%	0.1	5135
LOC102548511	0.0%	0.1%	0.1	5136
Senp8	0.0%	0.1%	0.1	5137
Ucma	0.0%	0.1%	0.1	5138
Il13ra2	0.0%	0.1%	0.1	5139
LOC103693640	0.0%	0.1%	0.1	5140
LOC100911699	0.0%	0.1%	0.1	5141
LOC103690477	0.0%	0.1%	0.1	5142

Lrrc52	0.0%	0.1%	0.1	5143
LOC103692075	0.0%	0.1%	0.1	5144
Hist1h2bl	0.0%	0.1%	0.1	5145
LOC102547056	0.0%	0.1%	0.1	5146
Kcnab1	0.0%	0.1%	0.1	5147
Foxl2	0.0%	0.1%	0.1	5148
LOC100912052	0.0%	0.1%	0.1	5149
Mfsd4a	0.0%	0.1%	0.1	5150
Myzap	0.0%	0.1%	0.1	5151
Pth1r	0.0%	0.1%	0.1	5152
LOC103690593	0.0%	0.1%	0.1	5153
Dnaaf1	0.0%	0.1%	0.1	5154
Abcc5	0.0%	0.1%	0.1	5155
Adh6	0.0%	0.1%	0.1	5156
Gp6	0.0%	0.1%	0.1	5157
LOC108349172	0.0%	0.1%	0.1	5158
LOC103693687	0.0%	0.1%	0.1	5159
Itgb8	0.0%	0.1%	0.1	5160
LOC108353659	0.0%	0.1%	0.1	5161
LOC102554113	0.0%	0.1%	0.1	5162
LOC102555688	0.0%	0.1%	0.1	5163
Klra17	0.0%	0.1%	0.1	5164
LOC108352190	0.0%	0.1%	0.1	5165
Pid1	0.0%	0.1%	0.1	5166
LOC102552083	0.0%	0.1%	0.1	5167
LOC689519	0.0%	0.1%	0.1	5168
LOC103690070	0.0%	0.1%	0.1	5169
Sage1	0.0%	0.1%	0.1	5170
LOC102555319	0.0%	0.1%	0.1	5171
LOC102557501	0.0%	0.1%	0.1	5172
Apol2	0.0%	0.1%	0.1	5173
Dyx1c1	0.0%	0.1%	0.1	5174
Prss55	0.0%	0.1%	0.1	5175
LOC108351267	0.0%	0.1%	0.1	5176
Carmil1	0.0%	0.1%	0.1	5177
Coro6	0.0%	0.1%	0.1	5178
LOC108352340	0.0%	0.1%	0.1	5179
LOC103694579	0.0%	0.1%	0.1	5180
Mab21l2	0.0%	0.1%	0.1	5181
LOC103693505	0.0%	0.1%	0.1	5182
Cplx2	0.0%	0.1%	0.1	5183
LOC103692975	0.0%	0.1%	0.1	5184
Gsg2	0.0%	0.1%	0.1	5185
LOC691661	0.0%	0.1%	0.1	5186
LOC102554312	0.0%	0.1%	0.1	5187
Fev	0.0%	0.1%	0.1	5188
LOC108353675	0.0%	0.1%	0.1	5189
Mtbp	0.0%	0.1%	0.1	5190
LOC102550729	0.0%	0.1%	0.1	5191
Asap1	0.0%	0.1%	0.1	5192
Defal1	0.0%	0.1%	0.1	5193
LOC102547771	0.0%	0.1%	0.1	5194
Rbpjl	0.0%	0.1%	0.1	5195
LOC102555303	0.0%	0.1%	0.1	5196
LOC108349713	0.0%	0.1%	0.1	5197
LOC103690152	0.0%	0.1%	0.1	5198
LOC102556316	0.0%	0.1%	0.1	5199
LOC108352497	0.0%	0.1%	0.1	5200
Zfp59	0.0%	0.1%	0.1	5201
Igsf6	0.0%	0.1%	0.1	5202
LOC102550510	0.0%	0.1%	0.1	5203
LOC102552150	0.0%	0.1%	0.1	5204
LOC102555357	0.0%	0.1%	0.1	5205
Scml2	0.0%	0.1%	0.1	5206
Itpripl1	0.0%	0.1%	0.1	5207
Tcea2	0.0%	0.1%	0.1	5208

Traf3ip3	0.0%	0.1%	0.1	5209
Gramd1b	0.0%	0.1%	0.1	5210
LOC108351639	0.0%	0.1%	0.1	5211
Ctrc	0.0%	0.1%	0.1	5212
Ckmt1b	0.0%	0.1%	0.1	5213
LOC102551624	0.0%	0.1%	0.1	5214
Hormad2	0.0%	0.1%	0.1	5215
LOC102553285	0.0%	0.1%	0.1	5216
LOC102556038	0.0%	0.1%	0.1	5217
Lilrb3a	0.0%	0.1%	0.1	5218
Nxpe1	0.0%	0.1%	0.1	5219
LOC108350069	0.0%	0.1%	0.1	5220
LOC103694989	0.0%	0.1%	0.1	5221
B3gntl1	0.0%	0.1%	0.1	5222
RGD1566029	0.0%	0.1%	0.1	5223
LOC102546966	0.0%	0.1%	0.1	5224
Ccdc175	0.0%	0.1%	0.1	5225
Jag2	0.0%	0.1%	0.1	5226
Fam83g	0.0%	0.1%	0.1	5227
RSA-14-44	0.0%	0.1%	0.1	5228
Rcor3	0.0%	0.1%	0.1	5229
Lnp1	0.0%	0.1%	0.1	5230
Pitpnm3	0.0%	0.1%	0.1	5231
Rnase1	0.0%	0.1%	0.1	5232
Cfap57	0.0%	0.1%	0.1	5233
Cdh15	0.0%	0.1%	0.1	5234
LOC108352807	0.0%	0.1%	0.1	5235
Egfl8	0.0%	0.1%	0.1	5236
Fzr1	0.0%	0.1%	0.1	5237
Emp2	0.0%	0.1%	0.1	5238
LOC103690947	0.0%	0.1%	0.1	5239
Raet1l	0.0%	0.1%	0.1	5240
RGD1565132	0.0%	0.1%	0.1	5241
Hist2h4a	0.0%	0.1%	0.1	5242
Ppp1r3f	0.0%	0.1%	0.1	5243
LOC100363225	0.0%	0.1%	0.1	5244
Ranbp17	0.0%	0.1%	0.1	5245
Mss51	0.0%	0.1%	0.1	5246
Spire2	0.0%	0.1%	0.1	5247
LOC108349419	0.0%	0.1%	0.1	5248
Npb	0.0%	0.1%	0.1	5249
LOC102550271	0.0%	0.1%	0.1	5250
LOC102556526	0.0%	0.1%	0.1	5251
LOC102547473	0.0%	0.1%	0.1	5252
LOC103694368	0.0%	0.1%	0.1	5253
LOC102555445	0.0%	0.1%	0.1	5254
LOC102557062	0.0%	0.1%	0.1	5255
LOC100911374	0.0%	0.1%	0.1	5256
LOC102549723	0.0%	0.1%	0.1	5257
LOC102553576	0.0%	0.1%	0.1	5258
Ces2	0.0%	0.1%	0.1	5259
Eln	0.0%	0.1%	0.1	5260
LOC679863	0.0%	0.1%	0.1	5261
LOC108348172	0.0%	0.1%	0.1	5262
LOC108351227	0.0%	0.1%	0.1	5263
Cds2	0.0%	0.1%	0.1	5264
Rprml	0.0%	0.1%	0.1	5265
Galnt18	0.0%	0.1%	0.1	5266
Oscar	0.0%	0.1%	0.1	5267
Arr3	0.0%	0.1%	0.1	5268
Cdkl3	0.0%	0.1%	0.1	5269
LOC103692282	0.0%	0.1%	0.1	5270
Mat2a	0.0%	0.1%	0.1	5271
Pnma2	0.0%	0.1%	0.1	5272
Cttnbp2	0.0%	0.1%	0.1	5273
LOC103690039	0.0%	0.1%	0.1	5274

LOC108348193	0.0%	0.1%	0.1	5275
Col5a2	0.0%	0.1%	0.1	5276
LOC102550664	0.0%	0.1%	0.1	5277
Plxdc2	0.0%	0.1%	0.1	5278
Sh3tc1	0.0%	0.1%	0.1	5279
LOC108353281	0.0%	0.1%	0.1	5280
Eml1	0.0%	0.1%	0.1	5281
Lama5	0.0%	0.1%	0.1	5282
Pate4	0.0%	0.1%	0.1	5283
LOC102547162	0.0%	0.1%	0.1	5284
RGD1565753	0.0%	0.1%	0.1	5285
LOC102554278	0.0%	0.1%	0.1	5286
Nek5	0.0%	0.1%	0.1	5287
LOC100364062	0.0%	0.1%	0.1	5288
LOC103692793	0.0%	0.1%	0.1	5289
Mcub	0.0%	0.1%	0.1	5290
Il27	0.0%	0.1%	0.1	5291
LOC102549265	0.0%	0.1%	0.1	5292
Hap1	0.0%	0.1%	0.1	5293
Hic1	0.0%	0.1%	0.1	5294
Fut7	0.0%	0.1%	0.1	5295
Alas2	0.0%	0.1%	0.1	5296
Lamb1	0.0%	0.1%	0.1	5297
LOC108350503	0.0%	0.1%	0.1	5298
Tspan5	0.0%	0.1%	0.1	5299
Rgs9bp	0.0%	0.1%	0.1	5300
Mir155hg	0.0%	0.1%	0.1	5301
Zfp507	0.0%	0.1%	0.1	5302
Metrn	0.0%	0.1%	0.1	5303
LOC102554505	0.0%	0.1%	0.1	5304
Poli	0.0%	0.1%	0.1	5305
Fcrla	0.0%	0.1%	0.1	5306
Pcdha10	0.0%	0.1%	0.1	5307
Grk3	0.0%	0.1%	0.1	5308
Ttll8	0.0%	0.1%	0.1	5309
LOC108349486	0.0%	0.1%	0.1	5310
LOC103689946	0.0%	0.1%	0.1	5311
LOC102553017	0.0%	0.1%	0.1	5312
Plekhd1	0.0%	0.1%	0.1	5313
Fam111a	0.0%	0.1%	0.1	5314
LOC103692234	0.0%	0.1%	0.1	5315
Ssmem1	0.0%	0.1%	0.1	5316
LOC103692345	0.0%	0.1%	0.1	5317
Cd300a	0.0%	0.1%	0.1	5318
LOC102551952	0.0%	0.1%	0.1	5319
Pfn2	0.0%	0.1%	0.1	5320
Mylk3	0.0%	0.1%	0.1	5321
LOC102552005	0.0%	0.1%	0.1	5322
Wnt9b	0.0%	0.1%	0.1	5323
Fam65c	0.0%	0.1%	0.1	5324
LOC102554987	0.0%	0.1%	0.1	5325
Parvg	0.0%	0.1%	0.1	5326
Fam57a	0.0%	0.1%	0.1	5327
Lxn	0.0%	0.1%	0.1	5328
LOC100912361	0.0%	0.1%	0.1	5329
Cenpt	0.0%	0.1%	0.1	5330
RGD1559441	0.0%	0.1%	0.1	5331
Hgf	0.0%	0.1%	0.1	5332
LOC102554188	0.0%	0.1%	0.1	5333
LOC108350715	0.0%	0.1%	0.1	5334
Osbpl10	0.0%	0.1%	0.1	5335
LOC100363712	0.0%	0.1%	0.1	5336
Col6a1	0.0%	0.1%	0.1	5337
Wdr78	0.0%	0.1%	0.1	5338
LOC102550441	0.0%	0.1%	0.1	5339
LOC108352904	0.0%	0.1%	0.1	5340

Slpil3	0.0%	0.1%	0.1	5341
Vav3	0.0%	0.1%	0.1	5342
RGD1563400	0.0%	0.1%	0.1	5343
RGD1563015	0.0%	0.1%	0.1	5344
LOC679818	0.0%	0.1%	0.1	5345
LOC685796	0.0%	0.1%	0.1	5346
RGD1307182	0.0%	0.1%	0.1	5347
Ak9	0.0%	0.1%	0.1	5348
LOC102551268	0.0%	0.1%	0.1	5349
Reck	0.0%	0.1%	0.1	5350
LOC102554570	0.0%	0.1%	0.1	5351
Kif23	0.0%	0.1%	0.1	5352
LOC103692116	0.0%	0.1%	0.1	5353
LOC108350398	0.0%	0.1%	0.1	5354
Spata6	0.0%	0.1%	0.1	5355
LOC103691511	0.0%	0.1%	0.1	5356
Pigb	0.0%	0.1%	0.1	5357
Mphosph9	0.0%	0.1%	0.1	5358
LOC103692227	0.0%	0.1%	0.1	5359
RGD1559859	0.0%	0.1%	0.1	5360
LOC102549740	0.0%	0.1%	0.1	5361
LOC108353151	0.0%	0.1%	0.1	5362
LOC108353655	0.0%	0.1%	0.1	5363
LOC108350947	0.0%	0.1%	0.1	5364
Folr2	0.0%	0.1%	0.1	5365
LOC103695261	0.0%	0.1%	0.1	5366
Jade3	0.0%	0.1%	0.1	5367
LOC108349407	0.0%	0.1%	0.1	5368
Atp6v0a4	0.0%	0.1%	0.1	5369
LOC100909405	0.0%	0.1%	0.1	5370
Fkbp10	0.0%	0.1%	0.1	5371
Podxl2	0.0%	0.1%	0.1	5372
Prss35	0.0%	0.1%	0.1	5373
Ly96	0.0%	0.1%	0.1	5374
LOC102548875	0.0%	0.1%	0.1	5375
LOC108350506	0.0%	0.1%	0.1	5376
LOC102547236	0.0%	0.1%	0.1	5377
Nhsl2	0.0%	0.1%	0.1	5378
Wnt2	0.0%	0.1%	0.1	5379
LOC102552107	0.0%	0.1%	0.1	5380
Hcn2	0.0%	0.1%	0.1	5381
Tcf15	0.0%	0.1%	0.1	5382
LOC102555044	0.0%	0.1%	0.1	5383
Slco4a1	0.0%	0.1%	0.1	5384
LOC102553941	0.0%	0.1%	0.1	5385
LOC691813	0.0%	0.1%	0.1	5386
LOC108350786	0.0%	0.1%	0.1	5387
Hmgn5	8.2%	8.3%	0.1	5388
Iqcd	7.8%	7.9%	0.1	5389
Qsox1	3.5%	3.6%	0.1	5390
Pyroxd1	3.5%	3.6%	0.1	5391
Tmem82	3.1%	3.2%	0.1	5392
Cml2	3.1%	3.2%	0.1	5393
LOC108352063	3.1%	3.2%	0.1	5394
LOC690414	3.1%	3.2%	0.1	5395
Mt1	73.7%	73.8%	0.1	5396
Camlg	2.7%	2.8%	0.1	5397
Nav2	2.7%	2.8%	0.1	5398
LOC100910795	2.7%	2.8%	0.1	5399
Adam5	48.2%	48.3%	0.0	5400
LOC100363494	2.4%	2.4%	0.0	5401
LOC363500	2.4%	2.4%	0.0	5402
Alyref	6.3%	6.3%	0.0	5403
Slc39a10	2.0%	2.0%	0.0	5404
Thg1l	2.0%	2.0%	0.0	5405
Mettl1	2.0%	2.0%	0.0	5406

Fam172a	2.0%	2.0%	0.0	5407
Cep63	2.0%	2.0%	0.0	5408
Mad1l1	2.0%	2.0%	0.0	5409
Nek8	10.2%	10.2%	0.0	5410
Mfsd11	1.6%	1.6%	0.0	5411
Elp4	1.6%	1.6%	0.0	5412
Plscr3	1.6%	1.6%	0.0	5413
Id1	1.6%	1.6%	0.0	5414
Tead1	1.6%	1.6%	0.0	5415
Eif1ax	1.6%	1.6%	0.0	5416
Nipa2	5.5%	5.5%	0.0	5417
Prps2	1.2%	1.2%	0.0	5418
Jarid2	1.2%	1.2%	0.0	5419
Oas1i	1.2%	1.2%	0.0	5420
Vps4a	1.2%	1.2%	0.0	5421
Tmem231	1.2%	1.2%	0.0	5422
RT1-CE13	1.2%	1.2%	0.0	5423
Zfp263	1.2%	1.2%	0.0	5424
Zfp821	1.2%	1.2%	0.0	5425
Trim8	1.2%	1.2%	0.0	5426
Med13l	1.2%	1.2%	0.0	5427
Prdm4	1.2%	1.2%	0.0	5428
Snapc3	1.2%	1.2%	0.0	5429
Mbp	1.2%	1.2%	0.0	5430
Rev1	1.2%	1.2%	0.0	5431
Wrn	1.2%	1.2%	0.0	5432
Coq8b	1.2%	1.2%	0.0	5433
Supt3h	1.2%	1.2%	0.0	5434
Lig1	1.2%	1.2%	0.0	5435
Rnf20	1.2%	1.2%	0.0	5436
LOC100362315	1.2%	1.2%	0.0	5437
Rgmb	0.8%	0.8%	0.0	5438
Rab27a	0.8%	0.8%	0.0	5439
Gstcd	0.8%	0.8%	0.0	5440
Hsf4	0.8%	0.8%	0.0	5441
Cyp2u1	0.8%	0.8%	0.0	5442
LOC102547943	0.8%	0.8%	0.0	5443
Nlrc5	0.8%	0.8%	0.0	5444
Snrpn	0.8%	0.8%	0.0	5445
Vps33b	0.8%	0.8%	0.0	5446
Fam168b	0.8%	0.8%	0.0	5447
LOC108353703	0.8%	0.8%	0.0	5448
Samd11	0.8%	0.8%	0.0	5449
Traf3ip1	0.8%	0.8%	0.0	5450
Slc26a6	0.8%	0.8%	0.0	5451
Thra	0.8%	0.8%	0.0	5452
LOC100360950	0.8%	0.8%	0.0	5453
Ptgfr	0.8%	0.8%	0.0	5454
Fam76a	0.8%	0.8%	0.0	5455
Ces4a	0.8%	0.8%	0.0	5456
Coq4	0.8%	0.8%	0.0	5457
Lysmd4	0.8%	0.8%	0.0	5458
Ogfrl1	0.8%	0.8%	0.0	5459
Emsy	0.8%	0.8%	0.0	5460
LOC690871	9.0%	9.0%	0.0	5461
Vwa5a	4.7%	4.7%	0.0	5462
Fpgt	0.4%	0.4%	0.0	5463
Unc5d	0.4%	0.4%	0.0	5464
Eogt	0.4%	0.4%	0.0	5465
LOC102553540	0.4%	0.4%	0.0	5466
MGC93861	0.4%	0.4%	0.0	5467
Ccne1	0.4%	0.4%	0.0	5468
RGD1304810	0.4%	0.4%	0.0	5469
Pea15	0.4%	0.4%	0.0	5470
Nup107	0.4%	0.4%	0.0	5471
LOC108353425	0.4%	0.4%	0.0	5472

Trim44	0.4%	0.4%	0.0	5473
Dnajc16	0.4%	0.4%	0.0	5474
Gnpda2	0.4%	0.4%	0.0	5475
LOC108348090	0.4%	0.4%	0.0	5476
Gjc3	0.4%	0.4%	0.0	5477
Gltscr1	0.4%	0.4%	0.0	5478
Alg10	0.4%	0.4%	0.0	5479
Anks3	0.4%	0.4%	0.0	5480
LOC103694456	0.4%	0.4%	0.0	5481
E2f1	0.4%	0.4%	0.0	5482
A2m	0.4%	0.4%	0.0	5483
Ptprg	0.4%	0.4%	0.0	5484
LOC103693257	0.4%	0.4%	0.0	5485
Trim13	0.4%	0.4%	0.0	5486
Chst3	0.4%	0.4%	0.0	5487
Zbtb7b	0.4%	0.4%	0.0	5488
LOC102553444	0.4%	0.4%	0.0	5489
Agtpbp1	0.4%	0.4%	0.0	5490
Smug1	0.4%	0.4%	0.0	5491
Recql5	0.4%	0.4%	0.0	5492
LOC102547175	0.4%	0.4%	0.0	5493
Stat5a	0.4%	0.4%	0.0	5494
RGD1306739	0.4%	0.4%	0.0	5495
Fcrl1	0.4%	0.4%	0.0	5496
Tmppe	0.4%	0.4%	0.0	5497
Zhx2	0.4%	0.4%	0.0	5498
LOC103693999	0.4%	0.4%	0.0	5499
Abhd8	0.4%	0.4%	0.0	5500
Nmur1	0.4%	0.4%	0.0	5501
Ap1s2	0.4%	0.4%	0.0	5502
Lgals2	0.4%	0.4%	0.0	5503
Als2cl	0.4%	0.4%	0.0	5504
Pde6c	0.4%	0.4%	0.0	5505
Olr1353	0.4%	0.4%	0.0	5506
Gpn3	0.4%	0.4%	0.0	5507
Cenpk	0.4%	0.4%	0.0	5508
Tubb3	0.4%	0.4%	0.0	5509
Ripply1	0.4%	0.4%	0.0	5510
Zfp560	0.4%	0.4%	0.0	5511
Galr3	0.4%	0.4%	0.0	5512
Mutyh	0.4%	0.4%	0.0	5513
Ctdspl	0.4%	0.4%	0.0	5514
LOC103693274	0.4%	0.4%	0.0	5515
Entpd6	0.4%	0.4%	0.0	5516
Cstf3	0.4%	0.4%	0.0	5517
Invs	0.4%	0.4%	0.0	5518
Meis3	0.4%	0.4%	0.0	5519
Lrp4	0.4%	0.4%	0.0	5520
Nat2	0.4%	0.4%	0.0	5521
Mical3	0.4%	0.4%	0.0	5522
Elavl2	0.4%	0.4%	0.0	5523
Sgsh	0.4%	0.4%	0.0	5524
Jrkl	0.4%	0.4%	0.0	5525
B3galt6	0.4%	0.4%	0.0	5526
Ncapd2	0.4%	0.4%	0.0	5527
Nsmce4a	0.4%	0.4%	0.0	5528
LOC683536	0.4%	0.4%	0.0	5529
Nwd1	0.4%	0.4%	0.0	5530
Smtnl2	0.4%	0.4%	0.0	5531
Slf1	0.4%	0.4%	0.0	5532
Csgalnact2	0.4%	0.4%	0.0	5533
LOC103692512	0.4%	0.4%	0.0	5534
Phactr2	0.4%	0.4%	0.0	5535
Plxnc1	0.4%	0.4%	0.0	5536
LOC100909712	0.4%	0.4%	0.0	5537
LOC102549538	0.4%	0.4%	0.0	5538

LOC257650	0.4%	0.4%	0.0	5539
Tmem80	0.4%	0.4%	0.0	5540
Slc7a4	0.4%	0.4%	0.0	5541
Phospho1	0.4%	0.4%	0.0	5542
Fzd6	0.4%	0.4%	0.0	5543
Gabpb2	0.4%	0.4%	0.0	5544
Tctn3	0.4%	0.4%	0.0	5545
LOC108348308	0.4%	0.4%	0.0	5546
Ear11	0.4%	0.4%	0.0	5547
Alb	100.0%	100.0%	0.0	5548
Tf	100.0%	100.0%	0.0	5549
Hp	100.0%	100.0%	0.0	5550
Apoe	100.0%	100.0%	0.0	5551
Ttr	100.0%	100.0%	0.0	5552
Col25a1	0.0%	0.0%	0.0	5553
LOC102547840	0.0%	0.0%	0.0	5554
LOC102556574	0.0%	0.0%	0.0	5555
31-Aug	0.0%	0.0%	0.0	5556
Abca16	0.0%	0.0%	0.0	5557
Adam1a	0.0%	0.0%	0.0	5558
Aifm3	0.0%	0.0%	0.0	5559
Ammecr1	0.0%	0.0%	0.0	5560
Areg	0.0%	0.0%	0.0	5561
Arhgap19	0.0%	0.0%	0.0	5562
Arhgap44	0.0%	0.0%	0.0	5563
Atp1b2	0.0%	0.0%	0.0	5564
Avil	0.0%	0.0%	0.0	5565
Bahcc1	0.0%	0.0%	0.0	5566
C1qtnf4	0.0%	0.0%	0.0	5567
Cacnb4	0.0%	0.0%	0.0	5568
Calcb	0.0%	0.0%	0.0	5569
Ccdc18	0.0%	0.0%	0.0	5570
Ccnf	0.0%	0.0%	0.0	5571
Cdyl2	0.0%	0.0%	0.0	5572
Cenpf	0.0%	0.0%	0.0	5573
Cmtm4	0.0%	0.0%	0.0	5574
Ddit4l	0.0%	0.0%	0.0	5575
Defb24	0.0%	0.0%	0.0	5576
Eef2kmt	0.0%	0.0%	0.0	5577
Fam129a	0.0%	0.0%	0.0	5578
Fam155b	0.0%	0.0%	0.0	5579
Fam214b	0.0%	0.0%	0.0	5580
Fam221a	0.0%	0.0%	0.0	5581
Fam69c	0.0%	0.0%	0.0	5582
Fbxw17	0.0%	0.0%	0.0	5583
Fez1	0.0%	0.0%	0.0	5584
Foxg1	0.0%	0.0%	0.0	5585
Foxm1	0.0%	0.0%	0.0	5586
Ftx	0.0%	0.0%	0.0	5587
Gpx7	0.0%	0.0%	0.0	5588
Hpdl	0.0%	0.0%	0.0	5589
Il17d	0.0%	0.0%	0.0	5590
Inhbb	0.0%	0.0%	0.0	5591
Iqub	0.0%	0.0%	0.0	5592
Irx3	0.0%	0.0%	0.0	5593
Kcnj11	0.0%	0.0%	0.0	5594
Kcnq2	0.0%	0.0%	0.0	5595
Klf1	0.0%	0.0%	0.0	5596
Klhl35	0.0%	0.0%	0.0	5597
Lamb3	0.0%	0.0%	0.0	5598
Lin7a	0.0%	0.0%	0.0	5599
Lin9	0.0%	0.0%	0.0	5600
Lmod2	0.0%	0.0%	0.0	5601
LOC100363116	0.0%	0.0%	0.0	5602
LOC100909657	0.0%	0.0%	0.0	5603
LOC100909824	0.0%	0.0%	0.0	5604

LOC100909897	0.0%	0.0%	0.0	5605
LOC100910117	0.0%	0.0%	0.0	5606
LOC100910596	0.0%	0.0%	0.0	5607
LOC100910628	0.0%	0.0%	0.0	5608
LOC100911180	0.0%	0.0%	0.0	5609
LOC100912787	0.0%	0.0%	0.0	5610
LOC102546868	0.0%	0.0%	0.0	5611
LOC102547212	0.0%	0.0%	0.0	5612
LOC102547266	0.0%	0.0%	0.0	5613
LOC102547278	0.0%	0.0%	0.0	5614
LOC102547718	0.0%	0.0%	0.0	5615
LOC102548361	0.0%	0.0%	0.0	5616
LOC102548781	0.0%	0.0%	0.0	5617
LOC102549468	0.0%	0.0%	0.0	5618
LOC102550168	0.0%	0.0%	0.0	5619
LOC102550236	0.0%	0.0%	0.0	5620
LOC102550357	0.0%	0.0%	0.0	5621
LOC102550491	0.0%	0.0%	0.0	5622
LOC102550543	0.0%	0.0%	0.0	5623
LOC102550637	0.0%	0.0%	0.0	5624
LOC102550786	0.0%	0.0%	0.0	5625
LOC102550863	0.0%	0.0%	0.0	5626
LOC102550910	0.0%	0.0%	0.0	5627
LOC102551276	0.0%	0.0%	0.0	5628
LOC102551348	0.0%	0.0%	0.0	5629
LOC102551683	0.0%	0.0%	0.0	5630
LOC102552417	0.0%	0.0%	0.0	5631
LOC102552566	0.0%	0.0%	0.0	5632
LOC102552812	0.0%	0.0%	0.0	5633
LOC102552971	0.0%	0.0%	0.0	5634
LOC102553092	0.0%	0.0%	0.0	5635
LOC102553236	0.0%	0.0%	0.0	5636
LOC102553417	0.0%	0.0%	0.0	5637
LOC102553634	0.0%	0.0%	0.0	5638
LOC102554680	0.0%	0.0%	0.0	5639
LOC102555083	0.0%	0.0%	0.0	5640
LOC102555509	0.0%	0.0%	0.0	5641
LOC102556151	0.0%	0.0%	0.0	5642
LOC102556687	0.0%	0.0%	0.0	5643
LOC102557556	0.0%	0.0%	0.0	5644
LOC102557559	0.0%	0.0%	0.0	5645
LOC103690073	0.0%	0.0%	0.0	5646
LOC103690137	0.0%	0.0%	0.0	5647
LOC103690319	0.0%	0.0%	0.0	5648
LOC103690347	0.0%	0.0%	0.0	5649
LOC103690523	0.0%	0.0%	0.0	5650
LOC103690608	0.0%	0.0%	0.0	5651
LOC103691017	0.0%	0.0%	0.0	5652
LOC103691752	0.0%	0.0%	0.0	5653
LOC103692250	0.0%	0.0%	0.0	5654
LOC103692417	0.0%	0.0%	0.0	5655
LOC103692524	0.0%	0.0%	0.0	5656
LOC103692770	0.0%	0.0%	0.0	5657
LOC103693356	0.0%	0.0%	0.0	5658
LOC103695115	0.0%	0.0%	0.0	5659
LOC108348191	0.0%	0.0%	0.0	5660
LOC108348311	0.0%	0.0%	0.0	5661
LOC108348384	0.0%	0.0%	0.0	5662
LOC108348946	0.0%	0.0%	0.0	5663
LOC108349037	0.0%	0.0%	0.0	5664
LOC108349075	0.0%	0.0%	0.0	5665
LOC108349243	0.0%	0.0%	0.0	5666
LOC108349707	0.0%	0.0%	0.0	5667
LOC108350107	0.0%	0.0%	0.0	5668
LOC108350666	0.0%	0.0%	0.0	5669
LOC108350796	0.0%	0.0%	0.0	5670

LOC108350932	0.0%	0.0%	0.0	5671
LOC108350964	0.0%	0.0%	0.0	5672
LOC108350981	0.0%	0.0%	0.0	5673
LOC108351221	0.0%	0.0%	0.0	5674
LOC108351961	0.0%	0.0%	0.0	5675
LOC108352385	0.0%	0.0%	0.0	5676
LOC108352417	0.0%	0.0%	0.0	5677
LOC108352943	0.0%	0.0%	0.0	5678
LOC108353639	0.0%	0.0%	0.0	5679
LOC685081	0.0%	0.0%	0.0	5680
LOC685203	0.0%	0.0%	0.0	5681
LOC685655	0.0%	0.0%	0.0	5682
LOC689561	0.0%	0.0%	0.0	5683
LOC691249	0.0%	0.0%	0.0	5684
Lrrc51	0.0%	0.0%	0.0	5685
Lurap1	0.0%	0.0%	0.0	5686
Mdh1b	0.0%	0.0%	0.0	5687
Mertk	0.0%	0.0%	0.0	5688
Mlf1	0.0%	0.0%	0.0	5689
Mogat1	0.0%	0.0%	0.0	5690
Mroh2a	0.0%	0.0%	0.0	5691
Myo1h	0.0%	0.0%	0.0	5692
Napb	0.0%	0.0%	0.0	5693
Nckap5l	0.0%	0.0%	0.0	5694
Neurl1	0.0%	0.0%	0.0	5695
Nfe2	0.0%	0.0%	0.0	5696
Nrxn1	0.0%	0.0%	0.0	5697
Nsg2	0.0%	0.0%	0.0	5698
Nt5dc3	0.0%	0.0%	0.0	5699
Nxpe2	0.0%	0.0%	0.0	5700
Papln	0.0%	0.0%	0.0	5701
Parn	0.0%	0.0%	0.0	5702
Plxna4	0.0%	0.0%	0.0	5703
Pmel	0.0%	0.0%	0.0	5704
Ppic	0.0%	0.0%	0.0	5705
Prdm11	0.0%	0.0%	0.0	5706
Prkd1	0.0%	0.0%	0.0	5707
Progesterone	0.0%	0.0%	0.0	5708
Prx	0.0%	0.0%	0.0	5709
Rccd1	0.0%	0.0%	0.0	5710
Rftn2	0.0%	0.0%	0.0	5711
RGD1304624	0.0%	0.0%	0.0	5712
RGD1560171	0.0%	0.0%	0.0	5713
RGD1560291	0.0%	0.0%	0.0	5714
RGD1560470	0.0%	0.0%	0.0	5715
RGD1560925	0.0%	0.0%	0.0	5716
RGD1562608	0.0%	0.0%	0.0	5717
RGD1564937	0.0%	0.0%	0.0	5718
Rhov	0.0%	0.0%	0.0	5719
Rp1	0.0%	0.0%	0.0	5720
Rsph9	0.0%	0.0%	0.0	5721
Rundc3b	0.0%	0.0%	0.0	5722
Scn4b	0.0%	0.0%	0.0	5723
Sdcbp2	0.0%	0.0%	0.0	5724
Seh1l	0.0%	0.0%	0.0	5725
Shbg	0.0%	0.0%	0.0	5726
Slc15a1	0.0%	0.0%	0.0	5727
Slc1a4	0.0%	0.0%	0.0	5728
Slc3a1	0.0%	0.0%	0.0	5729
Slco1a5	0.0%	0.0%	0.0	5730
Smarcd3	0.0%	0.0%	0.0	5731
Smyd5	0.0%	0.0%	0.0	5732
Sox21	0.0%	0.0%	0.0	5733
Spata25	0.0%	0.0%	0.0	5734
Spdya	0.0%	0.0%	0.0	5735
Stat6	0.0%	0.0%	0.0	5736

Tbc1d24	0.0%	0.0%	0.0	5737
Tctex1d4	0.0%	0.0%	0.0	5738
Tdrkh	0.0%	0.0%	0.0	5739
Tmem121	0.0%	0.0%	0.0	5740
Tmem18	0.0%	0.0%	0.0	5741
Tmem198b	0.0%	0.0%	0.0	5742
Tmem35b	0.0%	0.0%	0.0	5743
Tmem51	0.0%	0.0%	0.0	5744
Tmprss5	0.0%	0.0%	0.0	5745
Tnks	0.0%	0.0%	0.0	5746
Tnn	0.0%	0.0%	0.0	5747
Tonsl	0.0%	0.0%	0.0	5748
Tp73	0.0%	0.0%	0.0	5749
Trim16	0.0%	0.0%	0.0	5750
Trim68	0.0%	0.0%	0.0	5751
Trim7	0.0%	0.0%	0.0	5752
Trip13	0.0%	0.0%	0.0	5753
Ugt2b7	0.0%	0.0%	0.0	5754
Vom2r80	0.0%	0.0%	0.0	5755
Wbp2nl	0.0%	0.0%	0.0	5756
Wfdc1	0.0%	0.0%	0.0	5757
Wif1	0.0%	0.0%	0.0	5758
Xkr6	0.0%	0.0%	0.0	5759
Zfp275	0.0%	0.0%	0.0	5760
Zfp296	0.0%	0.0%	0.0	5761
Zfp316	0.0%	0.0%	0.0	5762
Zfp382	0.0%	0.0%	0.0	5763
Zfp398	0.0%	0.0%	0.0	5764
Zfp597	0.0%	0.0%	0.0	5765
Zfp78	0.0%	0.0%	0.0	5766
Zranb3	0.0%	0.0%	0.0	5767
Cbx5	3.9%	3.9%	0.0	5768
FAM120C	3.5%	3.5%	0.0	5769
Mcu	3.1%	3.1%	0.0	5770
Abcb8	7.1%	7.0%	0.0	5771
Tnfrsf1b	2.7%	2.7%	0.0	5772
Enpp5	2.7%	2.7%	0.0	5773
Chfr	2.7%	2.7%	0.0	5774
Mink1	2.7%	2.7%	0.0	5775
Bloc1s5	2.7%	2.7%	0.0	5776
Fgfr3	2.7%	2.7%	0.0	5777
Gbp5	2.4%	2.3%	0.0	5778
LOC102552818	2.4%	2.3%	0.0	5779
Isg20	2.0%	1.9%	0.0	5780
Tbl2	2.0%	1.9%	0.0	5781
Fbxo27	2.0%	1.9%	0.0	5782
Sult2b1	2.0%	1.9%	0.0	5783
Pdxk	2.0%	1.9%	0.0	5784
Crnde	2.0%	1.9%	0.0	5785
Pip5k1a	1.6%	1.5%	0.0	5786
Rfc3	1.6%	1.5%	0.0	5787
Zfp710	1.6%	1.5%	0.0	5788
Nupr2	1.6%	1.5%	0.0	5789
Diablo	1.6%	1.5%	0.0	5790
Zrsr2	1.6%	1.5%	0.0	5791
Gemin7l1	1.6%	1.5%	0.0	5792
Oxsr1	1.6%	1.5%	0.0	5793
LOC100365112	1.6%	1.5%	0.0	5794
Lrit2	1.6%	1.5%	0.0	5795
LOC103689978	1.6%	1.5%	0.0	5796
LOC680627	1.6%	1.5%	0.0	5797
RT1-M3-1	1.2%	1.1%	-0.1	5798
Cyth1	1.2%	1.1%	-0.1	5799
Lrp2	1.2%	1.1%	-0.1	5800
RGD1305704	1.2%	1.1%	-0.1	5801
Bloc1s6	1.2%	1.1%	-0.1	5802

Stxbp6	1.2%	1.1%	-0.1	5803
LOC108351964	1.2%	1.1%	-0.1	5804
Rilpl2	1.2%	1.1%	-0.1	5805
LOC500959	1.2%	1.1%	-0.1	5806
Cdc25a	1.2%	1.1%	-0.1	5807
S100pbp	1.2%	1.1%	-0.1	5808
Sars2	1.2%	1.1%	-0.1	5809
Med12	1.2%	1.1%	-0.1	5810
Nup37	1.2%	1.1%	-0.1	5811
Maml1	1.2%	1.1%	-0.1	5812
Sri	17.6%	17.6%	-0.1	5813
Rassf9	0.8%	0.7%	-0.1	5814
Klhl8	0.8%	0.7%	-0.1	5815
Epha4	0.8%	0.7%	-0.1	5816
Herc3	0.8%	0.7%	-0.1	5817
Smg7	0.8%	0.7%	-0.1	5818
Nvl	0.8%	0.7%	-0.1	5819
LOC103689927	0.8%	0.7%	-0.1	5820
LOC100911660	0.8%	0.7%	-0.1	5821
Nphp1	0.8%	0.7%	-0.1	5822
Klrg1	0.8%	0.7%	-0.1	5823
LOC102551473	0.8%	0.7%	-0.1	5824
Ecd	0.8%	0.7%	-0.1	5825
Caap1	0.8%	0.7%	-0.1	5826
LOC103690160	0.8%	0.7%	-0.1	5827
Cep131	0.8%	0.7%	-0.1	5828
LOC102552889	0.8%	0.7%	-0.1	5829
Znf768	0.8%	0.7%	-0.1	5830
LOC100910810	0.8%	0.7%	-0.1	5831
Tldc1	0.8%	0.7%	-0.1	5832
Rab11fip4	0.8%	0.7%	-0.1	5833
RGD1562289	0.8%	0.7%	-0.1	5834
Hltf	0.8%	0.7%	-0.1	5835
Wdr20	0.8%	0.7%	-0.1	5836
Rbm46	0.8%	0.7%	-0.1	5837
Arid2	0.8%	0.7%	-0.1	5838
Zfp280d	0.8%	0.7%	-0.1	5839
Plcb1	0.8%	0.7%	-0.1	5840
Ints14	0.8%	0.7%	-0.1	5841
Sfxn5	0.8%	0.7%	-0.1	5842
Spats2	0.8%	0.7%	-0.1	5843
Zdhhc18	0.8%	0.7%	-0.1	5844
Kif13a	0.8%	0.7%	-0.1	5845
Gltscr11	0.8%	0.7%	-0.1	5846
Fam110a	0.8%	0.7%	-0.1	5847
Nudt15	0.8%	0.7%	-0.1	5848
Camk2g	0.8%	0.7%	-0.1	5849
Zfp161	0.4%	0.3%	-0.1	5850
Smcr8	0.4%	0.3%	-0.1	5851
Cercam	0.4%	0.3%	-0.1	5852
LOC102554737	0.4%	0.3%	-0.1	5853
Zfp282	0.4%	0.3%	-0.1	5854
Ptn	0.4%	0.3%	-0.1	5855
Chek1	0.4%	0.3%	-0.1	5856
LOC103693421	0.4%	0.3%	-0.1	5857
Rell2	0.4%	0.3%	-0.1	5858
Ddah2	0.4%	0.3%	-0.1	5859
Defb1	0.4%	0.3%	-0.1	5860
Zfp346	0.4%	0.3%	-0.1	5861
RT1-N2	0.4%	0.3%	-0.1	5862
LOC108351007	0.4%	0.3%	-0.1	5863
LOC102549391	0.4%	0.3%	-0.1	5864
Supt7l	0.4%	0.3%	-0.1	5865
LOC102555503	0.4%	0.3%	-0.1	5866
Aurkc	0.4%	0.3%	-0.1	5867
Zc4h2	0.4%	0.3%	-0.1	5868

LOC103694537	0.4%	0.3%	-0.1	5869
Per1	0.4%	0.3%	-0.1	5870
LOC108350968	0.4%	0.3%	-0.1	5871
Zc2hc1a	0.4%	0.3%	-0.1	5872
Pcdhga5	0.4%	0.3%	-0.1	5873
LOC102555188	0.4%	0.3%	-0.1	5874
Sox6	0.4%	0.3%	-0.1	5875
Tp53i11	0.4%	0.3%	-0.1	5876
Mdm1	0.4%	0.3%	-0.1	5877
Fam114a1l1	0.4%	0.3%	-0.1	5878
Fam83d	0.4%	0.3%	-0.1	5879
Mettl15	0.4%	0.3%	-0.1	5880
Tsc22d4	0.4%	0.3%	-0.1	5881
Chst13	0.4%	0.3%	-0.1	5882
LOC102547959	0.4%	0.3%	-0.1	5883
LOC501266	0.4%	0.3%	-0.1	5884
Ppp1r12b	0.4%	0.3%	-0.1	5885
LOC108352556	0.4%	0.3%	-0.1	5886
LOC100359515	0.4%	0.3%	-0.1	5887
Rfx3	0.4%	0.3%	-0.1	5888
Pidd1	0.4%	0.3%	-0.1	5889
Qtrt2	0.4%	0.3%	-0.1	5890
Lonrf1	0.4%	0.3%	-0.1	5891
LOC108352106	0.4%	0.3%	-0.1	5892
Pbx1	0.4%	0.3%	-0.1	5893
Asf1b	0.4%	0.3%	-0.1	5894
Iqcb1	0.4%	0.3%	-0.1	5895
Mtfr2	0.4%	0.3%	-0.1	5896
Lig4	0.4%	0.3%	-0.1	5897
Papolg	0.4%	0.3%	-0.1	5898
Tnrc18	0.4%	0.3%	-0.1	5899
LOC680322	0.4%	0.3%	-0.1	5900
LOC100910577	0.4%	0.3%	-0.1	5901
LOC102553975	0.4%	0.3%	-0.1	5902
Ccdc14	0.4%	0.3%	-0.1	5903
LOC103692399	0.4%	0.3%	-0.1	5904
Plekhg6	0.4%	0.3%	-0.1	5905
Map3k9	0.4%	0.3%	-0.1	5906
Atp6ap2	0.4%	0.3%	-0.1	5907
Slc35a5	0.4%	0.3%	-0.1	5908
Disc1	0.4%	0.3%	-0.1	5909
Alms1	0.4%	0.3%	-0.1	5910
Mybl2	0.4%	0.3%	-0.1	5911
Galc	0.4%	0.3%	-0.1	5912
Ptprt	0.4%	0.3%	-0.1	5913
Tbx3	0.4%	0.3%	-0.1	5914
Pparg	0.4%	0.3%	-0.1	5915
Ropn1l	0.4%	0.3%	-0.1	5916
LOC100910487	0.4%	0.3%	-0.1	5917
Alpk1	0.4%	0.3%	-0.1	5918
Ccdc9	0.4%	0.3%	-0.1	5919
Zkscan4	0.4%	0.3%	-0.1	5920
Ddx31	0.4%	0.3%	-0.1	5921
Gucd1	0.4%	0.3%	-0.1	5922
Cep76	0.4%	0.3%	-0.1	5923
Rpgrip1l	0.4%	0.3%	-0.1	5924
MGC94199	0.4%	0.3%	-0.1	5925
LOC684016	0.4%	0.3%	-0.1	5926
Eml5	0.4%	0.3%	-0.1	5927
Arhgap6	0.4%	0.3%	-0.1	5928
Kif1bp	0.4%	0.3%	-0.1	5929
Thumpd3-as1	0.4%	0.3%	-0.1	5930
Snn	0.4%	0.3%	-0.1	5931
Cbx7	0.4%	0.3%	-0.1	5932
Ppm1n	0.4%	0.3%	-0.1	5933
Ptgis	0.4%	0.3%	-0.1	5934

Spc25	0.4%	0.3%	-0.1	5935
Rnf212	0.4%	0.3%	-0.1	5936
LOC108349088	0.4%	0.3%	-0.1	5937
Map1s	0.4%	0.3%	-0.1	5938
LOC100910106	0.4%	0.3%	-0.1	5939
Nova1	0.4%	0.3%	-0.1	5940
Iqgap1	0.4%	0.3%	-0.1	5941
Zfp867	0.4%	0.3%	-0.1	5942
Plscr1	0.4%	0.3%	-0.1	5943
Rbfox2	0.4%	0.3%	-0.1	5944
Camkmt	0.4%	0.3%	-0.1	5945
RGD1561847	0.4%	0.3%	-0.1	5946
C2cd3	0.4%	0.3%	-0.1	5947
LOC108349973	0.4%	0.3%	-0.1	5948
Ebi3	0.4%	0.3%	-0.1	5949
RGD1560248	0.4%	0.3%	-0.1	5950
Abcg8	0.4%	0.3%	-0.1	5951
Aurkb	0.4%	0.3%	-0.1	5952
Nsd3	0.4%	0.3%	-0.1	5953
Ccdc113	0.4%	0.3%	-0.1	5954
LOC102549817	0.4%	0.3%	-0.1	5955
A2ml1	0.4%	0.3%	-0.1	5956
Gcc1	0.4%	0.3%	-0.1	5957
RGD1359158	4.3%	4.2%	-0.1	5958
Top1	3.9%	3.8%	-0.1	5959
Amotl2	3.5%	3.4%	-0.1	5960
Emg1	3.1%	3.0%	-0.1	5961
Pnisr	3.1%	3.0%	-0.1	5962
Rpl37	27.8%	27.7%	-0.1	5963
Abca2	2.7%	2.6%	-0.1	5964
Fam219b	2.7%	2.6%	-0.1	5965
LOC103694866	2.7%	2.6%	-0.1	5966
LOC108352923	2.7%	2.6%	-0.1	5967
Rnf26	2.7%	2.6%	-0.1	5968
Rbbp4	6.7%	6.6%	-0.1	5969
Lgr4	2.4%	2.2%	-0.1	5970
Tex9	2.4%	2.2%	-0.1	5971
Usp21	2.4%	2.2%	-0.1	5972
Ap1g1	2.4%	2.2%	-0.1	5973
Casp12	2.4%	2.2%	-0.1	5974
Snx9	2.4%	2.2%	-0.1	5975
RGD1564541	2.0%	1.8%	-0.1	5976
Tubb2b	2.0%	1.8%	-0.1	5977
LOC103692273	1.6%	1.4%	-0.1	5978
Rprd2	1.6%	1.4%	-0.1	5979
LOC103689940	1.6%	1.4%	-0.1	5980
Gatad2b	1.6%	1.4%	-0.1	5981
Naip5	1.6%	1.4%	-0.1	5982
Tmem245	1.6%	1.4%	-0.1	5983
Txlna	1.6%	1.4%	-0.1	5984
Haus5	1.6%	1.4%	-0.1	5985
Msh2	1.6%	1.4%	-0.1	5986
Nmt2	1.6%	1.4%	-0.1	5987
Ggps1	1.6%	1.4%	-0.1	5988
Msrb3	1.6%	1.4%	-0.1	5989
Cep44	1.6%	1.4%	-0.1	5990
Smpd4	1.6%	1.4%	-0.1	5991
Ncoa2	1.2%	1.0%	-0.1	5992
Prmt2	1.2%	1.0%	-0.1	5993
Nr6a1	1.2%	1.0%	-0.1	5994
Fam117a	1.2%	1.0%	-0.1	5995
Gpbp1l2	1.2%	1.0%	-0.1	5996
Fgr	1.2%	1.0%	-0.1	5997
Pex12	1.2%	1.0%	-0.1	5998
Trim3	1.2%	1.0%	-0.1	5999
Tbc1d10b	1.2%	1.0%	-0.1	6000

Actr6	1.2%	1.0%	-0.1	6001
Pigl	1.2%	1.0%	-0.1	6002
LOC103695227	1.2%	1.0%	-0.1	6003
Cspp1	1.2%	1.0%	-0.1	6004
LOC102555396	1.2%	1.0%	-0.1	6005
Ppp2r5b	1.2%	1.0%	-0.1	6006
Polr3c	1.2%	1.0%	-0.1	6007
Znf660	1.2%	1.0%	-0.1	6008
Taf9b	1.2%	1.0%	-0.1	6009
Stx16	1.2%	1.0%	-0.1	6010
Rassf6	34.5%	34.4%	-0.1	6011
Fdxr	9.4%	9.3%	-0.1	6012
Mapt	0.8%	0.6%	-0.1	6013
RGD1564125	0.8%	0.6%	-0.1	6014
LOC681224	0.8%	0.6%	-0.1	6015
Cnnm2	0.8%	0.6%	-0.1	6016
Dennd6b	0.8%	0.6%	-0.1	6017
Ocrl	0.8%	0.6%	-0.1	6018
Dlg3	0.8%	0.6%	-0.1	6019
Cabin1	0.8%	0.6%	-0.1	6020
XAF1	0.8%	0.6%	-0.1	6021
Asb3	0.8%	0.6%	-0.1	6022
Zfyve26	0.8%	0.6%	-0.1	6023
RGD1311756	0.8%	0.6%	-0.1	6024
LOC102550787	0.8%	0.6%	-0.1	6025
Rapgef5	0.8%	0.6%	-0.1	6026
Apex2	0.8%	0.6%	-0.1	6027
Pitpnc1	0.8%	0.6%	-0.1	6028
Runx1	0.8%	0.6%	-0.1	6029
Zfp133	0.8%	0.6%	-0.1	6030
Btbd3	0.8%	0.6%	-0.1	6031
Ackr3	0.8%	0.6%	-0.1	6032
Ptpn4	0.8%	0.6%	-0.1	6033
Tubgcp3	0.8%	0.6%	-0.1	6034
Mpi	0.8%	0.6%	-0.1	6035
Rexo1	0.8%	0.6%	-0.1	6036
Rgl3	0.8%	0.6%	-0.1	6037
Fam109a	0.8%	0.6%	-0.1	6038
LOC102554565	0.8%	0.6%	-0.1	6039
Zbtb10	0.8%	0.6%	-0.1	6040
Igsf8	0.8%	0.6%	-0.1	6041
Sec14l1	0.8%	0.6%	-0.1	6042
Tcp11l1	0.8%	0.6%	-0.1	6043
Cep120	0.8%	0.6%	-0.1	6044
Mfng	0.8%	0.6%	-0.1	6045
LOC108352229	0.8%	0.6%	-0.1	6046
Nid1	0.8%	0.6%	-0.1	6047
Nelfa	0.8%	0.6%	-0.1	6048
B3gnt2	4.7%	4.6%	-0.1	6049
LOC100911917	0.4%	0.2%	-0.2	6050
Gas1	0.4%	0.2%	-0.2	6051
Havcr1	0.4%	0.2%	-0.2	6052
Vom2r34	0.4%	0.2%	-0.2	6053
Lipt1	0.4%	0.2%	-0.2	6054
LOC691257	0.4%	0.2%	-0.2	6055
C11H22orf29	0.4%	0.2%	-0.2	6056
Arc	0.4%	0.2%	-0.2	6057
Tcaf1	0.4%	0.2%	-0.2	6058
Pcdhga12	0.4%	0.2%	-0.2	6059
RGD1561413	0.4%	0.2%	-0.2	6060
LOC100912483	0.4%	0.2%	-0.2	6061
Abcg2	0.4%	0.2%	-0.2	6062
Rtn4rl1	0.4%	0.2%	-0.2	6063
Tmem267	0.4%	0.2%	-0.2	6064
Top2b	0.4%	0.2%	-0.2	6065
Mlkl	0.4%	0.2%	-0.2	6066

Prdm10	0.4%	0.2%	-0.2	6067
Zfp532	0.4%	0.2%	-0.2	6068
Plek2	0.4%	0.2%	-0.2	6069
LOC102546433	0.4%	0.2%	-0.2	6070
Fbxl18	0.4%	0.2%	-0.2	6071
LOC103693391	0.4%	0.2%	-0.2	6072
LOC102550328	0.4%	0.2%	-0.2	6073
C1rl	0.4%	0.2%	-0.2	6074
LOC100910558	0.4%	0.2%	-0.2	6075
Tmem201	0.4%	0.2%	-0.2	6076
Mpzl1	0.4%	0.2%	-0.2	6077
Tbc1d32	0.4%	0.2%	-0.2	6078
Vom2r31	0.4%	0.2%	-0.2	6079
Pym1	0.4%	0.2%	-0.2	6080
Aldh18a1	0.4%	0.2%	-0.2	6081
LOC103693805	0.4%	0.2%	-0.2	6082
Pde8b	0.4%	0.2%	-0.2	6083
Cdc73	0.4%	0.2%	-0.2	6084
LOC108350523	0.4%	0.2%	-0.2	6085
Map10	0.4%	0.2%	-0.2	6086
P2rx3	0.4%	0.2%	-0.2	6087
Cep162	0.4%	0.2%	-0.2	6088
Dus4l	0.4%	0.2%	-0.2	6089
Aknad1	0.4%	0.2%	-0.2	6090
LOC102547728	0.4%	0.2%	-0.2	6091
LOC100912785	0.4%	0.2%	-0.2	6092
LOC103693817	0.4%	0.2%	-0.2	6093
Acpp	0.4%	0.2%	-0.2	6094
Smyd4	0.4%	0.2%	-0.2	6095
Znf408	0.4%	0.2%	-0.2	6096
Gatsl3	0.4%	0.2%	-0.2	6097
Zbtb26	0.4%	0.2%	-0.2	6098
Map3k1	0.4%	0.2%	-0.2	6099
LOC103691273	0.4%	0.2%	-0.2	6100
Acvr2b	0.4%	0.2%	-0.2	6101
Mkrn3	0.4%	0.2%	-0.2	6102
Sidt1	0.4%	0.2%	-0.2	6103
Lepr	0.4%	0.2%	-0.2	6104
Dhx35	0.4%	0.2%	-0.2	6105
Rps6ka6	0.4%	0.2%	-0.2	6106
Bpifa6	0.4%	0.2%	-0.2	6107
LOC684327	0.4%	0.2%	-0.2	6108
LOC102556556	0.4%	0.2%	-0.2	6109
Patz1	0.4%	0.2%	-0.2	6110
Fam43a	0.4%	0.2%	-0.2	6111
Gcnt6	0.4%	0.2%	-0.2	6112
Ldoc1l	0.4%	0.2%	-0.2	6113
Tspan8	0.4%	0.2%	-0.2	6114
Zmynd10	0.4%	0.2%	-0.2	6115
LOC103694635	0.4%	0.2%	-0.2	6116
Adrb2	0.4%	0.2%	-0.2	6117
S100g	0.4%	0.2%	-0.2	6118
LOC108350772	0.4%	0.2%	-0.2	6119
Plekhm3	0.4%	0.2%	-0.2	6120
Fer	0.4%	0.2%	-0.2	6121
Nckipsd	0.4%	0.2%	-0.2	6122
Intu	0.4%	0.2%	-0.2	6123
Cage1	0.4%	0.2%	-0.2	6124
Kif11	0.4%	0.2%	-0.2	6125
LOC103693478	0.4%	0.2%	-0.2	6126
Zmat3	0.4%	0.2%	-0.2	6127
Fgf11	0.4%	0.2%	-0.2	6128
Rps6ka5	0.4%	0.2%	-0.2	6129
Pdcl	0.4%	0.2%	-0.2	6130
LOC108353171	0.4%	0.2%	-0.2	6131
RGD1562618	0.4%	0.2%	-0.2	6132

Tbx15	0.4%	0.2%	-0.2	6133
LOC102553014	0.4%	0.2%	-0.2	6134
LOC100361898	0.4%	0.2%	-0.2	6135
Rnf122	0.4%	0.2%	-0.2	6136
Arsk	0.4%	0.2%	-0.2	6137
Cpt1c	0.4%	0.2%	-0.2	6138
Dnhd1	0.4%	0.2%	-0.2	6139
Zfp51	0.4%	0.2%	-0.2	6140
Fam169b	0.4%	0.2%	-0.2	6141
LOC100912165	0.4%	0.2%	-0.2	6142
LOC102546432	0.4%	0.2%	-0.2	6143
Carns1	0.4%	0.2%	-0.2	6144
Lrch1	0.4%	0.2%	-0.2	6145
Usp12	0.4%	0.2%	-0.2	6146
Trim15	0.4%	0.2%	-0.2	6147
Morc2	0.4%	0.2%	-0.2	6148
Caskin2	0.4%	0.2%	-0.2	6149
LOC691764	0.4%	0.2%	-0.2	6150
Tspan9	0.4%	0.2%	-0.2	6151
Ms4a6a	0.4%	0.2%	-0.2	6152
LOC102551002	0.4%	0.2%	-0.2	6153
Kazald1	0.4%	0.2%	-0.2	6154
Zfp9	0.4%	0.2%	-0.2	6155
Rgs14	0.4%	0.2%	-0.2	6156
Gdpgp1	0.4%	0.2%	-0.2	6157
Zfp709l1	0.4%	0.2%	-0.2	6158
Tgm7	0.4%	0.2%	-0.2	6159
Ints2	0.4%	0.2%	-0.2	6160
Pomc	0.4%	0.2%	-0.2	6161
Dennd5b	0.4%	0.2%	-0.2	6162
Gmeb2	0.4%	0.2%	-0.2	6163
Tsen2	0.4%	0.2%	-0.2	6164
Rnf168	0.4%	0.2%	-0.2	6165
Crispld2	0.4%	0.2%	-0.2	6166
Rxra	0.4%	0.2%	-0.2	6167
Celf5	0.4%	0.2%	-0.2	6168
Foxj2	0.4%	0.2%	-0.2	6169
Rgs10	0.4%	0.2%	-0.2	6170
LOC102552752	0.4%	0.2%	-0.2	6171
LOC102555302	0.4%	0.2%	-0.2	6172
LOC108350057	0.4%	0.2%	-0.2	6173
Slx4	0.4%	0.2%	-0.2	6174
Sod3	0.4%	0.2%	-0.2	6175
Klhl11	0.4%	0.2%	-0.2	6176
Tulp4	0.4%	0.2%	-0.2	6177
LOC102557195	0.4%	0.2%	-0.2	6178
LOC102546544	0.4%	0.2%	-0.2	6179
Caps2	0.4%	0.2%	-0.2	6180
Cbl	0.4%	0.2%	-0.2	6181
Hes1	8.6%	8.5%	-0.2	6182
Fetub	100.0%	99.8%	-0.2	6183
LOC680406	100.0%	99.8%	-0.2	6184
Mgst1	100.0%	99.8%	-0.2	6185
Med31	3.9%	3.8%	-0.2	6186
LOC685067	3.9%	3.8%	-0.2	6187
Cxcl9	3.5%	3.4%	-0.2	6188
Mfsd1	3.5%	3.4%	-0.2	6189
Amz2	3.1%	3.0%	-0.2	6190
Grk6	3.1%	3.0%	-0.2	6191
Mief1	2.7%	2.6%	-0.2	6192
Wbp11	2.7%	2.6%	-0.2	6193
LOC103690059	2.7%	2.6%	-0.2	6194
Bdp1	2.7%	2.6%	-0.2	6195
Spc24	2.7%	2.6%	-0.2	6196
Kdm7a	2.4%	2.2%	-0.2	6197
LOC102552334	2.4%	2.2%	-0.2	6198

Sp140	2.4%	2.2%	-0.2	6199
LOC100362453	10.6%	10.4%	-0.2	6200
Cstf2	2.0%	1.8%	-0.2	6201
Asap3	2.0%	1.8%	-0.2	6202
Rabgap1l	2.0%	1.8%	-0.2	6203
Poldip3	2.0%	1.8%	-0.2	6204
Ggact	2.0%	1.8%	-0.2	6205
Anapc15	2.0%	1.8%	-0.2	6206
Golph3l	2.0%	1.8%	-0.2	6207
Stx17	2.0%	1.8%	-0.2	6208
LOC691387	2.0%	1.8%	-0.2	6209
Ap2a1	2.0%	1.8%	-0.2	6210
Pim3	5.9%	5.7%	-0.2	6211
Cln5	1.6%	1.4%	-0.2	6212
LOC498154	1.6%	1.4%	-0.2	6213
Msto1	1.6%	1.4%	-0.2	6214
Abhd15	1.6%	1.4%	-0.2	6215
Il7	1.6%	1.4%	-0.2	6216
Arid4a	1.6%	1.4%	-0.2	6217
Taco1	1.6%	1.4%	-0.2	6218
Zmym1	1.6%	1.4%	-0.2	6219
Pml	1.6%	1.4%	-0.2	6220
LOC103690323	1.6%	1.4%	-0.2	6221
LOC108353689	1.6%	1.4%	-0.2	6222
Med30	1.6%	1.4%	-0.2	6223
Sfswap	1.6%	1.4%	-0.2	6224
Ankra2	1.6%	1.4%	-0.2	6225
Rprm	1.6%	1.4%	-0.2	6226
Heg1	1.6%	1.4%	-0.2	6227
St5	1.6%	1.4%	-0.2	6228
Nars2	1.6%	1.4%	-0.2	6229
Socs5	1.6%	1.4%	-0.2	6230
Gpbp1l1	5.5%	5.3%	-0.2	6231
Ube2m	13.7%	13.5%	-0.2	6232
Gpatch1	1.2%	1.0%	-0.2	6233
Dock5	1.2%	1.0%	-0.2	6234
LOC103692307	1.2%	1.0%	-0.2	6235
Dedd	1.2%	1.0%	-0.2	6236
Usp2	1.2%	1.0%	-0.2	6237
LOC108348501	1.2%	1.0%	-0.2	6238
Mblac1	1.2%	1.0%	-0.2	6239
Wdr44	1.2%	1.0%	-0.2	6240
Prmt7	1.2%	1.0%	-0.2	6241
Ap5s1	1.2%	1.0%	-0.2	6242
Cep164	1.2%	1.0%	-0.2	6243
Ada	1.2%	1.0%	-0.2	6244
LOC102555727	1.2%	1.0%	-0.2	6245
Ogt	1.2%	1.0%	-0.2	6246
Gpx2	1.2%	1.0%	-0.2	6247
Tmem68	1.2%	1.0%	-0.2	6248
LOC102547832	1.2%	1.0%	-0.2	6249
Zufsp	1.2%	1.0%	-0.2	6250
Parg	1.2%	1.0%	-0.2	6251
Exosc6	1.2%	1.0%	-0.2	6252
Bmp2k	1.2%	1.0%	-0.2	6253
LOC100910438	1.2%	1.0%	-0.2	6254
Ncoa7	1.2%	1.0%	-0.2	6255
RGD1559962	1.2%	1.0%	-0.2	6256
Pi4k2b	1.2%	1.0%	-0.2	6257
Amt	9.4%	9.2%	-0.2	6258
Pycr1	0.8%	0.6%	-0.2	6259
Sfi1	0.8%	0.6%	-0.2	6260
Disp1	0.8%	0.6%	-0.2	6261
Rp9	0.8%	0.6%	-0.2	6262
Stau2	0.8%	0.6%	-0.2	6263
Gprasp1	0.8%	0.6%	-0.2	6264

Cdr2	0.8%	0.6%	-0.2	6265
LOC684819	0.8%	0.6%	-0.2	6266
Tnrc6c	0.8%	0.6%	-0.2	6267
RGD1306091	0.8%	0.6%	-0.2	6268
Spry4	0.8%	0.6%	-0.2	6269
Nfya	0.8%	0.6%	-0.2	6270
Kalrn	0.8%	0.6%	-0.2	6271
Zfp758	0.8%	0.6%	-0.2	6272
Angptl2	0.8%	0.6%	-0.2	6273
Hs1bp3	0.8%	0.6%	-0.2	6274
Zfp280b	0.8%	0.6%	-0.2	6275
Bend3	0.8%	0.6%	-0.2	6276
Zfp628	0.8%	0.6%	-0.2	6277
Frg1l1	0.8%	0.6%	-0.2	6278
Uaca	0.8%	0.6%	-0.2	6279
Sytl2	0.8%	0.6%	-0.2	6280
Hif1an	0.8%	0.6%	-0.2	6281
Zfp251	0.8%	0.6%	-0.2	6282
LOC100911865	0.8%	0.6%	-0.2	6283
Txnrd3	0.8%	0.6%	-0.2	6284
Aaas	0.8%	0.6%	-0.2	6285
Mipol1	0.8%	0.6%	-0.2	6286
Thumpd2	0.8%	0.6%	-0.2	6287
Ccdc71	0.8%	0.6%	-0.2	6288
Ehmt1	0.8%	0.6%	-0.2	6289
Card19	0.8%	0.6%	-0.2	6290
Rwdd3	0.8%	0.6%	-0.2	6291
Tex36	0.8%	0.6%	-0.2	6292
LOC102548752	0.8%	0.6%	-0.2	6293
LOC102548794	0.8%	0.6%	-0.2	6294
LOC102550986	0.8%	0.6%	-0.2	6295
Bbx	0.8%	0.6%	-0.2	6296
Eif5a	38.0%	37.8%	-0.2	6297
Grwd1	4.7%	4.5%	-0.2	6298
Dtx3l	4.7%	4.5%	-0.2	6299
RGD1560242	4.7%	4.5%	-0.2	6300
LOC103693480	0.4%	0.2%	-0.2	6301
Zw10	0.4%	0.2%	-0.2	6302
Tbc1d30	0.4%	0.2%	-0.2	6303
Klhl20	0.4%	0.2%	-0.2	6304
Clic4	0.4%	0.2%	-0.2	6305
Arid3a	0.4%	0.2%	-0.2	6306
Rsl1	0.4%	0.2%	-0.2	6307
Src	0.4%	0.2%	-0.2	6308
Ccdc163	0.4%	0.2%	-0.2	6309
Tmem184c	0.4%	0.2%	-0.2	6310
Sytl4	0.4%	0.2%	-0.2	6311
Zfp53	0.4%	0.2%	-0.2	6312
Rslcan18	0.4%	0.2%	-0.2	6313
LOC102547290	0.4%	0.2%	-0.2	6314
Shcbp1	0.4%	0.2%	-0.2	6315
Pih1d2	0.4%	0.2%	-0.2	6316
Fam227b	0.4%	0.2%	-0.2	6317
Rbak	0.4%	0.2%	-0.2	6318
Phf19	0.4%	0.2%	-0.2	6319
LOC100911928	0.4%	0.2%	-0.2	6320
LOC679087	0.4%	0.2%	-0.2	6321
Pus7l	0.4%	0.2%	-0.2	6322
Ric3	0.4%	0.2%	-0.2	6323
Asb1	0.4%	0.2%	-0.2	6324
LOC100912076	0.4%	0.2%	-0.2	6325
LOC102547750	0.4%	0.2%	-0.2	6326
Mb21d1	0.4%	0.2%	-0.2	6327
Syne3	0.4%	0.2%	-0.2	6328
Fanci	0.4%	0.2%	-0.2	6329
LOC108353665	0.4%	0.2%	-0.2	6330

LOC689439	0.4%	0.2%	-0.2	6331
LOC102550291	0.4%	0.2%	-0.2	6332
Ccl20	0.4%	0.2%	-0.2	6333
Glis2	0.4%	0.2%	-0.2	6334
Cbx2	0.4%	0.2%	-0.2	6335
LOC108352908	0.4%	0.2%	-0.2	6336
Abca4	0.4%	0.2%	-0.2	6337
Wipf2	0.4%	0.2%	-0.2	6338
Iffo2	0.4%	0.2%	-0.2	6339
RGD1563049	0.4%	0.2%	-0.2	6340
Ciita	0.4%	0.2%	-0.2	6341
Zfp11	0.4%	0.2%	-0.2	6342
Satb2	0.4%	0.2%	-0.2	6343
Zfp174	0.4%	0.2%	-0.2	6344
LOC102557057	0.4%	0.2%	-0.2	6345
LOC108352959	0.4%	0.2%	-0.2	6346
Iqcc	0.4%	0.2%	-0.2	6347
Csmd1	0.4%	0.2%	-0.2	6348
LOC102553752	0.4%	0.2%	-0.2	6349
LOC102556485	0.4%	0.2%	-0.2	6350
Bbs10	0.4%	0.2%	-0.2	6351
LOC102547495	0.4%	0.2%	-0.2	6352
Cetn4	0.4%	0.2%	-0.2	6353
Incenp	0.4%	0.2%	-0.2	6354
Cenpw	0.4%	0.2%	-0.2	6355
LOC102551140	0.4%	0.2%	-0.2	6356
Zfp94	0.4%	0.2%	-0.2	6357
Tpbg	0.4%	0.2%	-0.2	6358
Axin2	0.4%	0.2%	-0.2	6359
Ccdc86	0.4%	0.2%	-0.2	6360
Eddm3b	0.4%	0.2%	-0.2	6361
Rab23	0.4%	0.2%	-0.2	6362
Efna3	0.4%	0.2%	-0.2	6363
LOC102555189	0.4%	0.2%	-0.2	6364
Atrnl1	0.4%	0.2%	-0.2	6365
Exoc3l1	0.4%	0.2%	-0.2	6366
LOC100910478	0.4%	0.2%	-0.2	6367
Ndrg3	0.4%	0.2%	-0.2	6368
Dzip3	0.4%	0.2%	-0.2	6369
LOC103694375	0.4%	0.2%	-0.2	6370
Strada	0.4%	0.2%	-0.2	6371
Stox2	0.4%	0.2%	-0.2	6372
Tinag	0.4%	0.2%	-0.2	6373
LOC103692469	0.4%	0.2%	-0.2	6374
Tbkbp1	0.4%	0.2%	-0.2	6375
LOC103694914	0.4%	0.2%	-0.2	6376
Galns	0.4%	0.2%	-0.2	6377
RGD1311946	0.4%	0.2%	-0.2	6378
Map3k5	0.4%	0.2%	-0.2	6379
Pdk3	0.4%	0.2%	-0.2	6380
Zbtb34	0.4%	0.2%	-0.2	6381
Gca	0.4%	0.2%	-0.2	6382
Dbil5	0.4%	0.2%	-0.2	6383
LOC102555028	0.4%	0.2%	-0.2	6384
Map3k2	0.4%	0.2%	-0.2	6385
LOC103690033	0.4%	0.2%	-0.2	6386
Ap4m1	0.4%	0.2%	-0.2	6387
Chsy3	0.4%	0.2%	-0.2	6388
Cdt1	0.4%	0.2%	-0.2	6389
Pcdhga3	0.4%	0.2%	-0.2	6390
LOC100912537	0.4%	0.2%	-0.2	6391
Zfp329	0.4%	0.2%	-0.2	6392
LOC100912498	0.4%	0.2%	-0.2	6393
Plxna3	0.4%	0.2%	-0.2	6394
Zfp516	0.4%	0.2%	-0.2	6395
Zwilch	0.4%	0.2%	-0.2	6396

LOC100911357	0.4%	0.2%	-0.2	6397
Zfp978	0.4%	0.2%	-0.2	6398
Blnk	0.4%	0.2%	-0.2	6399
Epb42	0.4%	0.2%	-0.2	6400
Naa11	0.4%	0.2%	-0.2	6401
LOC102554932	0.4%	0.2%	-0.2	6402
LOC102550012	0.4%	0.2%	-0.2	6403
Pkia	0.4%	0.2%	-0.2	6404
Slc10a7	0.4%	0.2%	-0.2	6405
Zfp27	0.4%	0.2%	-0.2	6406
LOC108350648	0.4%	0.2%	-0.2	6407
Tmed8	0.4%	0.2%	-0.2	6408
Sbspon	0.4%	0.2%	-0.2	6409
LOC103691206	0.4%	0.2%	-0.2	6410
Aplf	0.4%	0.2%	-0.2	6411
Zfp606	0.4%	0.2%	-0.2	6412
LOC103689949	0.4%	0.2%	-0.2	6413
Smad6	0.4%	0.2%	-0.2	6414
Eid2b	0.4%	0.2%	-0.2	6415
LOC102554479	0.4%	0.2%	-0.2	6416
Zbtb45	0.4%	0.2%	-0.2	6417
Iqce	0.4%	0.2%	-0.2	6418
Tmem120b	0.4%	0.2%	-0.2	6419
Ttk	0.4%	0.2%	-0.2	6420
Zbtb8a	0.4%	0.2%	-0.2	6421
Flrt2	0.4%	0.2%	-0.2	6422
Itgb4	0.4%	0.2%	-0.2	6423
Napepld	0.4%	0.2%	-0.2	6424
LOC108348052	0.4%	0.2%	-0.2	6425
LOC108349921	0.4%	0.2%	-0.2	6426
Mtmr11	0.4%	0.2%	-0.2	6427
Tnfaip6	0.4%	0.2%	-0.2	6428
Ccdc62	0.4%	0.2%	-0.2	6429
Twist1	0.4%	0.2%	-0.2	6430
Nsd2	0.4%	0.2%	-0.2	6431
Arhgef9	0.4%	0.2%	-0.2	6432
Ago4	0.4%	0.2%	-0.2	6433
Znf354b	0.4%	0.2%	-0.2	6434
LOC102550200	0.4%	0.2%	-0.2	6435
Aasdh	0.4%	0.2%	-0.2	6436
Cntrob	0.4%	0.2%	-0.2	6437
Wdhd1	0.4%	0.2%	-0.2	6438
Ccne2	0.4%	0.2%	-0.2	6439
LOC102551626	0.4%	0.2%	-0.2	6440
Glb1l	0.4%	0.2%	-0.2	6441
Ssbp2	0.4%	0.2%	-0.2	6442
Zfp689	0.4%	0.2%	-0.2	6443
Cbx6	0.4%	0.2%	-0.2	6444
NEWGENE_1311521	0.4%	0.2%	-0.2	6445
Pkmyt1	0.4%	0.2%	-0.2	6446
LOC100912849	0.4%	0.2%	-0.2	6447
Zfp407	0.4%	0.2%	-0.2	6448
Gpsm1	0.4%	0.2%	-0.2	6449
Rdh13	0.4%	0.2%	-0.2	6450
LOC501934	8.6%	8.4%	-0.2	6451
Rbm25	8.6%	8.4%	-0.2	6452
Ahsg	100.0%	99.8%	-0.2	6453
Serpina1	100.0%	99.8%	-0.2	6454
Fabp1	100.0%	99.8%	-0.2	6455
Slbp	3.9%	3.7%	-0.2	6456
Eri3	3.9%	3.7%	-0.2	6457
Dtnb	28.6%	28.4%	-0.3	6458
LOC686774	3.5%	3.3%	-0.3	6459
Nin	3.5%	3.3%	-0.3	6460
LOC108348128	3.5%	3.3%	-0.3	6461
Fam114a1	3.1%	2.9%	-0.3	6462

Higd2a	11.4%	11.1%	-0.3	6463
Acox1	36.1%	35.8%	-0.3	6464
Foxa1	2.7%	2.5%	-0.3	6465
Adgre5	2.7%	2.5%	-0.3	6466
Fam122a	2.4%	2.1%	-0.3	6467
LOC100912002	2.4%	2.1%	-0.3	6468
Pnkp	2.4%	2.1%	-0.3	6469
Appl2	2.4%	2.1%	-0.3	6470
Zfp446	2.4%	2.1%	-0.3	6471
Id3	6.3%	6.0%	-0.3	6472
Syde2	2.0%	1.7%	-0.3	6473
RT1-CE14	2.0%	1.7%	-0.3	6474
Camta2	2.0%	1.7%	-0.3	6475
LOC100909645	2.0%	1.7%	-0.3	6476
Serinc5	2.0%	1.7%	-0.3	6477
LOC679822	2.0%	1.7%	-0.3	6478
LOC690155	2.0%	1.7%	-0.3	6479
LOC100912151	2.0%	1.7%	-0.3	6480
Dtwd2	1.6%	1.3%	-0.3	6481
Gmnn	1.6%	1.3%	-0.3	6482
LOC100910754	1.6%	1.3%	-0.3	6483
Pms1	1.6%	1.3%	-0.3	6484
Basp1	1.6%	1.3%	-0.3	6485
Tubgcp2	1.6%	1.3%	-0.3	6486
Bcor	1.6%	1.3%	-0.3	6487
Zfp266	1.6%	1.3%	-0.3	6488
Stap1	1.6%	1.3%	-0.3	6489
C1H10orf76	1.6%	1.3%	-0.3	6490
Frg1	1.6%	1.3%	-0.3	6491
Ppp6r3	9.8%	9.5%	-0.3	6492
G6pd	5.5%	5.2%	-0.3	6493
LOC102556241	1.2%	0.9%	-0.3	6494
Scmh1	1.2%	0.9%	-0.3	6495
Arse	1.2%	0.9%	-0.3	6496
Fam135a	1.2%	0.9%	-0.3	6497
Mettl21a	1.2%	0.9%	-0.3	6498
Ttc5	1.2%	0.9%	-0.3	6499
Ap1s3	1.2%	0.9%	-0.3	6500
Rnpc3	1.2%	0.9%	-0.3	6501
Dnaaf5	1.2%	0.9%	-0.3	6502
Sgsm2	1.2%	0.9%	-0.3	6503
Crkl	1.2%	0.9%	-0.3	6504
Rnf145	1.2%	0.9%	-0.3	6505
LOC103692527	1.2%	0.9%	-0.3	6506
Cinp	1.2%	0.9%	-0.3	6507
Med27	1.2%	0.9%	-0.3	6508
Ikbip	1.2%	0.9%	-0.3	6509
Gnl3l	1.2%	0.9%	-0.3	6510
Dmtn	1.2%	0.9%	-0.3	6511
St8sia3	1.2%	0.9%	-0.3	6512
Ufsp1	1.2%	0.9%	-0.3	6513
Atmin	1.2%	0.9%	-0.3	6514
Scrn1	13.3%	13.0%	-0.3	6515
Stmn3	0.8%	0.5%	-0.3	6516
Slc25a4	0.8%	0.5%	-0.3	6517
Ppm1h	0.8%	0.5%	-0.3	6518
Dpy19l4	0.8%	0.5%	-0.3	6519
Nupl2	0.8%	0.5%	-0.3	6520
Wdr31	0.8%	0.5%	-0.3	6521
Ptprn	0.8%	0.5%	-0.3	6522
Pola1	0.8%	0.5%	-0.3	6523
LOC103693157	0.8%	0.5%	-0.3	6524
Haus1	0.8%	0.5%	-0.3	6525
Plagl2	0.8%	0.5%	-0.3	6526
LOC108348901	0.8%	0.5%	-0.3	6527
Zswim7	0.8%	0.5%	-0.3	6528

Nipa1	0.8%	0.5%	-0.3	6529
LOC498759	0.8%	0.5%	-0.3	6530
Dmc1	0.8%	0.5%	-0.3	6531
Kmt5c	0.8%	0.5%	-0.3	6532
Polk	0.8%	0.5%	-0.3	6533
Gria3	0.8%	0.5%	-0.3	6534
Maged2	0.8%	0.5%	-0.3	6535
Mob3c	0.8%	0.5%	-0.3	6536
Jrk	0.8%	0.5%	-0.3	6537
Polr3b	0.8%	0.5%	-0.3	6538
Hps5	0.8%	0.5%	-0.3	6539
Cracr2b	0.8%	0.5%	-0.3	6540
LOC108348318	0.8%	0.5%	-0.3	6541
LOC103691643	0.8%	0.5%	-0.3	6542
LOC108351673	0.8%	0.5%	-0.3	6543
Rfx1	0.8%	0.5%	-0.3	6544
Tmem79	0.8%	0.5%	-0.3	6545
LOC108348250	0.8%	0.5%	-0.3	6546
RGD1564171	0.8%	0.5%	-0.3	6547
Tbc1d22a	0.8%	0.5%	-0.3	6548
Fbf1	0.8%	0.5%	-0.3	6549
Zfp341	0.8%	0.5%	-0.3	6550
LOC102555634	0.8%	0.5%	-0.3	6551
Pacrgl	0.8%	0.5%	-0.3	6552
LOC102547242	0.8%	0.5%	-0.3	6553
Nrtn	0.8%	0.5%	-0.3	6554
Trappc2	0.8%	0.5%	-0.3	6555
Tchp	0.8%	0.5%	-0.3	6556
Synj2	0.8%	0.5%	-0.3	6557
Pou2f1	0.8%	0.5%	-0.3	6558
Wdr25	0.8%	0.5%	-0.3	6559
Slc7a1	0.8%	0.5%	-0.3	6560
LOC103693974	0.4%	0.1%	-0.3	6561
LOC108352462	0.4%	0.1%	-0.3	6562
Gypa	0.4%	0.1%	-0.3	6563
LOC108349769	0.4%	0.1%	-0.3	6564
Fam171b	0.4%	0.1%	-0.3	6565
RGD1559908	0.4%	0.1%	-0.3	6566
LOC102557427	0.4%	0.1%	-0.3	6567
LOC100909599	0.4%	0.1%	-0.3	6568
Gnai1	0.4%	0.1%	-0.3	6569
Fign	0.4%	0.1%	-0.3	6570
LOC103691441	0.4%	0.1%	-0.3	6571
LOC102549264	0.4%	0.1%	-0.3	6572
LOC102547986	0.4%	0.1%	-0.3	6573
LOC102551718	0.4%	0.1%	-0.3	6574
Stx1b	0.4%	0.1%	-0.3	6575
LOC103692869	0.4%	0.1%	-0.3	6576
LOC108351265	0.4%	0.1%	-0.3	6577
Bag2	0.4%	0.1%	-0.3	6578
LOC108349405	0.4%	0.1%	-0.3	6579
Bmt2	0.4%	0.1%	-0.3	6580
Gstm5	0.4%	0.1%	-0.3	6581
LOC689766	0.4%	0.1%	-0.3	6582
LOC108350356	0.4%	0.1%	-0.3	6583
Cyp46a1	0.4%	0.1%	-0.3	6584
LOC103692052	0.4%	0.1%	-0.3	6585
LOC498265	0.4%	0.1%	-0.3	6586
Cyp2j10	0.4%	0.1%	-0.3	6587
Clip2	0.4%	0.1%	-0.3	6588
Kif4a	0.4%	0.1%	-0.3	6589
Ado	0.4%	0.1%	-0.3	6590
LOC102553884	0.4%	0.1%	-0.3	6591
Zfp780b	0.4%	0.1%	-0.3	6592
Bcat2	0.4%	0.1%	-0.3	6593
Tnfrsf10b	0.4%	0.1%	-0.3	6594

Abcc10	0.4%	0.1%	-0.3	6595
Kif18a	0.4%	0.1%	-0.3	6596
LOC108352570	0.4%	0.1%	-0.3	6597
Tbc1d10a	0.4%	0.1%	-0.3	6598
Zbtb47	0.4%	0.1%	-0.3	6599
Mgarp	0.4%	0.1%	-0.3	6600
LOC103690934	0.4%	0.1%	-0.3	6601
Foxq1	0.4%	0.1%	-0.3	6602
Ccdc24	0.4%	0.1%	-0.3	6603
LOC108349526	0.4%	0.1%	-0.3	6604
RGD1566386	0.4%	0.1%	-0.3	6605
Kctd1	0.4%	0.1%	-0.3	6606
Ralgps1	0.4%	0.1%	-0.3	6607
LOC102552207	0.4%	0.1%	-0.3	6608
Pqlc3	0.4%	0.1%	-0.3	6609
Rsad1	0.4%	0.1%	-0.3	6610
Actn1	0.4%	0.1%	-0.3	6611
LOC108348319	0.4%	0.1%	-0.3	6612
Nebl	0.4%	0.1%	-0.3	6613
LOC102554019	0.4%	0.1%	-0.3	6614
Srpx	0.4%	0.1%	-0.3	6615
Rgs8	0.4%	0.1%	-0.3	6616
Slc2a12	0.4%	0.1%	-0.3	6617
Zfp14	0.4%	0.1%	-0.3	6618
Ampd1	0.4%	0.1%	-0.3	6619
Cdkl5	0.4%	0.1%	-0.3	6620
Greb1l	0.4%	0.1%	-0.3	6621
LOC102550312	0.4%	0.1%	-0.3	6622
Hspb2	0.4%	0.1%	-0.3	6623
RGD1305928	0.4%	0.1%	-0.3	6624
Myh7b	0.4%	0.1%	-0.3	6625
LOC103695042	0.4%	0.1%	-0.3	6626
Prss22	0.4%	0.1%	-0.3	6627
Rps19bp1	0.4%	0.1%	-0.3	6628
Fbxo15	0.4%	0.1%	-0.3	6629
Nos1	0.4%	0.1%	-0.3	6630
Endov	0.4%	0.1%	-0.3	6631
Wdr73	0.4%	0.1%	-0.3	6632
Zfp182	0.4%	0.1%	-0.3	6633
LOC108352291	0.4%	0.1%	-0.3	6634
LOC102547185	0.4%	0.1%	-0.3	6635
LOC108352538	0.4%	0.1%	-0.3	6636
LOC102553451	0.4%	0.1%	-0.3	6637
Hspa1a	0.4%	0.1%	-0.3	6638
Rapgef6	0.4%	0.1%	-0.3	6639
Tnnc1	0.4%	0.1%	-0.3	6640
LOC102556368	0.4%	0.1%	-0.3	6641
LOC103694005	0.4%	0.1%	-0.3	6642
Ap4e1	0.4%	0.1%	-0.3	6643
LOC103694867	0.4%	0.1%	-0.3	6644
Inpp4a	0.4%	0.1%	-0.3	6645
Colgalt2	0.4%	0.1%	-0.3	6646
LOC102554403	0.4%	0.1%	-0.3	6647
Ror1	0.4%	0.1%	-0.3	6648
Recql4	0.4%	0.1%	-0.3	6649
Cxcl3	0.4%	0.1%	-0.3	6650
LOC102550105	0.4%	0.1%	-0.3	6651
Ttc25	0.4%	0.1%	-0.3	6652
LOC102551009	0.4%	0.1%	-0.3	6653
Pstpip2	0.4%	0.1%	-0.3	6654
Eaf2	0.4%	0.1%	-0.3	6655
Slc35g1	0.4%	0.1%	-0.3	6656
LOC102547697	0.4%	0.1%	-0.3	6657
Zfp169	0.4%	0.1%	-0.3	6658
LOC102547849	0.4%	0.1%	-0.3	6659
Gprc5a	0.4%	0.1%	-0.3	6660

LOC100910554	0.4%	0.1%	-0.3	6661
LOC102553491	0.4%	0.1%	-0.3	6662
Pcolce	0.4%	0.1%	-0.3	6663
Man2a2	0.4%	0.1%	-0.3	6664
Adgrg2	0.4%	0.1%	-0.3	6665
Nudt17	0.4%	0.1%	-0.3	6666
Wdr89	0.4%	0.1%	-0.3	6667
Helq	0.4%	0.1%	-0.3	6668
Dhx33	0.4%	0.1%	-0.3	6669
Zswim3	0.4%	0.1%	-0.3	6670
Ren	0.4%	0.1%	-0.3	6671
Wnk4	0.4%	0.1%	-0.3	6672
Tff3	0.4%	0.1%	-0.3	6673
LOC102548289	0.4%	0.1%	-0.3	6674
Slc25a24	0.4%	0.1%	-0.3	6675
LOC108349017	0.4%	0.1%	-0.3	6676
Rnase17	0.4%	0.1%	-0.3	6677
LOC103691413	0.4%	0.1%	-0.3	6678
LOC102553140	0.4%	0.1%	-0.3	6679
LOC100910021	0.4%	0.1%	-0.3	6680
N4bp3	0.4%	0.1%	-0.3	6681
Flrt1	0.4%	0.1%	-0.3	6682
Sarm1	0.4%	0.1%	-0.3	6683
Bbs7	0.4%	0.1%	-0.3	6684
LOC108349448	0.4%	0.1%	-0.3	6685
LOC102551095	0.4%	0.1%	-0.3	6686
LOC100910481	0.4%	0.1%	-0.3	6687
Casq1	0.4%	0.1%	-0.3	6688
RGD1561277	0.4%	0.1%	-0.3	6689
RGD1306474	0.4%	0.1%	-0.3	6690
Trpm5	0.4%	0.1%	-0.3	6691
Snx29	0.4%	0.1%	-0.3	6692
Ighmbp2	0.4%	0.1%	-0.3	6693
Hs6st2	0.4%	0.1%	-0.3	6694
Ttll1	0.4%	0.1%	-0.3	6695
Pgap1	0.4%	0.1%	-0.3	6696
Nfatc2ip	0.4%	0.1%	-0.3	6697
LOC103693072	0.4%	0.1%	-0.3	6698
Slc35e4	0.4%	0.1%	-0.3	6699
Mdc1	0.4%	0.1%	-0.3	6700
Tnfrsf22	0.4%	0.1%	-0.3	6701
LOC102548484	0.4%	0.1%	-0.3	6702
Yy2	0.4%	0.1%	-0.3	6703
RGD1560289	0.4%	0.1%	-0.3	6704
Spetex-2E	0.4%	0.1%	-0.3	6705
Acot1	0.4%	0.1%	-0.3	6706
LOC102553760	0.4%	0.1%	-0.3	6707
LOC102556012	0.4%	0.1%	-0.3	6708
LOC102551164	0.4%	0.1%	-0.3	6709
LOC102556915	0.4%	0.1%	-0.3	6710
Rasl11b	0.4%	0.1%	-0.3	6711
LOC102553244	0.4%	0.1%	-0.3	6712
LOC102554346	0.4%	0.1%	-0.3	6713
Slc30a2	0.4%	0.1%	-0.3	6714
Slc37a2	0.4%	0.1%	-0.3	6715
Bbs9	0.4%	0.1%	-0.3	6716
LOC102556967	0.4%	0.1%	-0.3	6717
Zfp141	0.4%	0.1%	-0.3	6718
LOC100911029	4.3%	4.0%	-0.3	6719
Rhoc	4.3%	4.0%	-0.3	6720
Ambp	100.0%	99.7%	-0.3	6721
Ccpg1	3.9%	3.6%	-0.3	6722
RGD1305713	3.9%	3.6%	-0.3	6723
Luc7l2	7.8%	7.5%	-0.3	6724
Luc7l	3.5%	3.2%	-0.3	6725
Tap2	3.5%	3.2%	-0.3	6726

Rbpj	3.1%	2.8%	-0.3	6727
Polr2d	3.1%	2.8%	-0.3	6728
Syne4	3.1%	2.8%	-0.3	6729
RGD1562378	3.1%	2.8%	-0.3	6730
Nsrp1	2.7%	2.4%	-0.3	6731
LOC108349054	6.7%	6.3%	-0.4	6732
Mycbp2	2.4%	2.0%	-0.4	6733
Ckap4	2.4%	2.0%	-0.4	6734
Brd8	2.4%	2.0%	-0.4	6735
LOC100911553	2.4%	2.0%	-0.4	6736
LOC103690502	2.4%	2.0%	-0.4	6737
Sclt1	2.0%	1.6%	-0.4	6738
Zc3h8	2.0%	1.6%	-0.4	6739
Vps45	2.0%	1.6%	-0.4	6740
Psip1	2.0%	1.6%	-0.4	6741
Neil1	2.0%	1.6%	-0.4	6742
Sh3bp2	2.0%	1.6%	-0.4	6743
Macf1	2.0%	1.6%	-0.4	6744
LOC108348072	2.0%	1.6%	-0.4	6745
Rsf1	2.0%	1.6%	-0.4	6746
Slc25a48	1.6%	1.2%	-0.4	6747
LOC103692976	1.6%	1.2%	-0.4	6748
Crnkl1	1.6%	1.2%	-0.4	6749
Dcaf15	1.6%	1.2%	-0.4	6750
Rrp9	1.6%	1.2%	-0.4	6751
Tbcel	1.6%	1.2%	-0.4	6752
RGD1307947	1.6%	1.2%	-0.4	6753
Tmem159	1.6%	1.2%	-0.4	6754
Tmem50b	1.6%	1.2%	-0.4	6755
Kin	1.6%	1.2%	-0.4	6756
Ces2i	1.6%	1.2%	-0.4	6757
LOC103691577	1.6%	1.2%	-0.4	6758
Rp2	1.6%	1.2%	-0.4	6759
Myef2	1.6%	1.2%	-0.4	6760
Armcx3	1.6%	1.2%	-0.4	6761
Pqlc2	1.6%	1.2%	-0.4	6762
Ccdc141	1.6%	1.2%	-0.4	6763
Mpzl2	1.6%	1.2%	-0.4	6764
Kbtbd3	1.6%	1.2%	-0.4	6765
LOC102550397	1.6%	1.2%	-0.4	6766
Dpp8	5.5%	5.1%	-0.4	6767
Clec2dl1	1.2%	0.8%	-0.4	6768
Tmprss2	1.2%	0.8%	-0.4	6769
LOC100359503	1.2%	0.8%	-0.4	6770
Tamm41	1.2%	0.8%	-0.4	6771
Spata5	1.2%	0.8%	-0.4	6772
Lrfn3	1.2%	0.8%	-0.4	6773
Pms2	1.2%	0.8%	-0.4	6774
Tpm4	1.2%	0.8%	-0.4	6775
LOC102556224	1.2%	0.8%	-0.4	6776
Gtf2a1	1.2%	0.8%	-0.4	6777
Wwtr1	1.2%	0.8%	-0.4	6778
LOC108353284	1.2%	0.8%	-0.4	6779
Adat2	1.2%	0.8%	-0.4	6780
LOC680017	1.2%	0.8%	-0.4	6781
Snurf	1.2%	0.8%	-0.4	6782
Tstd2	1.2%	0.8%	-0.4	6783
Bin2	1.2%	0.8%	-0.4	6784
Dcun1d2	1.2%	0.8%	-0.4	6785
Angel1	1.2%	0.8%	-0.4	6786
Tmem101	1.2%	0.8%	-0.4	6787
Kansl1l	1.2%	0.8%	-0.4	6788
Mitd1	1.2%	0.8%	-0.4	6789
Zmym3	1.2%	0.8%	-0.4	6790
LOC103690017	1.2%	0.8%	-0.4	6791
Clec2g	1.2%	0.8%	-0.4	6792

Fam179b	1.2%	0.8%	-0.4	6793
Jkamp	1.2%	0.8%	-0.4	6794
LOC100911994	1.2%	0.8%	-0.4	6795
Kif3a	1.2%	0.8%	-0.4	6796
Inhba	5.1%	4.7%	-0.4	6797
Idnk	13.3%	12.9%	-0.4	6798
Fancf	0.8%	0.4%	-0.4	6799
Glt1d1	0.8%	0.4%	-0.4	6800
LOC103691699	0.8%	0.4%	-0.4	6801
Ca5b	0.8%	0.4%	-0.4	6802
Pacs1	0.8%	0.4%	-0.4	6803
Nfatc1	0.8%	0.4%	-0.4	6804
Dusp14	0.8%	0.4%	-0.4	6805
LOC100911456	0.8%	0.4%	-0.4	6806
Kifc1	0.8%	0.4%	-0.4	6807
Mcpt8l3	0.8%	0.4%	-0.4	6808
Stn1	0.8%	0.4%	-0.4	6809
Ptrhd1	0.8%	0.4%	-0.4	6810
LOC102553232	0.8%	0.4%	-0.4	6811
Znf761	0.8%	0.4%	-0.4	6812
Amdhd1	0.8%	0.4%	-0.4	6813
Il18	0.8%	0.4%	-0.4	6814
Prss36	0.8%	0.4%	-0.4	6815
Steap4	0.8%	0.4%	-0.4	6816
Agap1	0.8%	0.4%	-0.4	6817
Llgl1	0.8%	0.4%	-0.4	6818
LOC103692167	0.8%	0.4%	-0.4	6819
Parp11	0.8%	0.4%	-0.4	6820
Cers5	0.8%	0.4%	-0.4	6821
LOC102546765	0.8%	0.4%	-0.4	6822
Cul9	0.8%	0.4%	-0.4	6823
Prim2	0.8%	0.4%	-0.4	6824
LOC100910054	0.8%	0.4%	-0.4	6825
LOC316820	0.8%	0.4%	-0.4	6826
Gbas	0.8%	0.4%	-0.4	6827
LOC102549227	0.8%	0.4%	-0.4	6828
Pard6a	0.8%	0.4%	-0.4	6829
Chtf18	0.8%	0.4%	-0.4	6830
LOC100911879	0.8%	0.4%	-0.4	6831
Nat14	0.8%	0.4%	-0.4	6832
Ssc4d	0.8%	0.4%	-0.4	6833
LOC100911545	0.8%	0.4%	-0.4	6834
Zfp111	0.8%	0.4%	-0.4	6835
Zcwpw2	0.8%	0.4%	-0.4	6836
Cyth3	0.8%	0.4%	-0.4	6837
Irs1	0.8%	0.4%	-0.4	6838
Angptl6	0.8%	0.4%	-0.4	6839
Erich1	0.8%	0.4%	-0.4	6840
Mdn1	0.8%	0.4%	-0.4	6841
Zfp26	0.8%	0.4%	-0.4	6842
Bbs2	0.8%	0.4%	-0.4	6843
Mga	0.8%	0.4%	-0.4	6844
Tmem67	0.8%	0.4%	-0.4	6845
LOC100912202	0.8%	0.4%	-0.4	6846
Nat8f1	0.8%	0.4%	-0.4	6847
Cdr2l	0.8%	0.4%	-0.4	6848
Zmynd12	0.8%	0.4%	-0.4	6849
Pcsk5	0.8%	0.4%	-0.4	6850
LOC102549998	0.8%	0.4%	-0.4	6851
Rnf144a	0.8%	0.4%	-0.4	6852
Arid3b	0.8%	0.4%	-0.4	6853
Vrk2	0.8%	0.4%	-0.4	6854
Ano6	0.8%	0.4%	-0.4	6855
LOC100912167	0.8%	0.4%	-0.4	6856
Ift172	0.8%	0.4%	-0.4	6857
Ptpdc1	0.8%	0.4%	-0.4	6858

Ppp3cb	0.8%	0.4%	-0.4	6859
Nek4	0.8%	0.4%	-0.4	6860
Primpol	0.8%	0.4%	-0.4	6861
Smg6	0.8%	0.4%	-0.4	6862
LOC102552900	4.7%	4.3%	-0.4	6863
Ripk1	4.7%	4.3%	-0.4	6864
Zfp667	0.4%	0.0%	-0.4	6865
Aatk	0.4%	0.0%	-0.4	6866
Abcb9	0.4%	0.0%	-0.4	6867
Adam22	0.4%	0.0%	-0.4	6868
Adamts3	0.4%	0.0%	-0.4	6869
Adnp2	0.4%	0.0%	-0.4	6870
Agbl3	0.4%	0.0%	-0.4	6871
Aldoc	0.4%	0.0%	-0.4	6872
Ankrd13b	0.4%	0.0%	-0.4	6873
Ankrd66	0.4%	0.0%	-0.4	6874
Ascl2	0.4%	0.0%	-0.4	6875
Atp13a5	0.4%	0.0%	-0.4	6876
Atxn1	0.4%	0.0%	-0.4	6877
Bcl2	0.4%	0.0%	-0.4	6878
Bdkrb1	0.4%	0.0%	-0.4	6879
C2cd4b	0.4%	0.0%	-0.4	6880
Cahm	0.4%	0.0%	-0.4	6881
Capn5	0.4%	0.0%	-0.4	6882
Car1	0.4%	0.0%	-0.4	6883
Card14	0.4%	0.0%	-0.4	6884
Carmil3	0.4%	0.0%	-0.4	6885
Ccdc88c	0.4%	0.0%	-0.4	6886
Cct6b	0.4%	0.0%	-0.4	6887
Cdan1	0.4%	0.0%	-0.4	6888
Cdc14a	0.4%	0.0%	-0.4	6889
Cdh24	0.4%	0.0%	-0.4	6890
Cep135	0.4%	0.0%	-0.4	6891
Cfap69	0.4%	0.0%	-0.4	6892
Chrdl1	0.4%	0.0%	-0.4	6893
Cngb1	0.4%	0.0%	-0.4	6894
Crybb3	0.4%	0.0%	-0.4	6895
Cspg5	0.4%	0.0%	-0.4	6896
Cx3cl1	0.4%	0.0%	-0.4	6897
Cyb561d1	0.4%	0.0%	-0.4	6898
Dnd1	0.4%	0.0%	-0.4	6899
Dnm3	0.4%	0.0%	-0.4	6900
Dsel	0.4%	0.0%	-0.4	6901
Dusp23	0.4%	0.0%	-0.4	6902
Dydc2	0.4%	0.0%	-0.4	6903
Dzip1	0.4%	0.0%	-0.4	6904
Ect2l	0.4%	0.0%	-0.4	6905
Efcab3	0.4%	0.0%	-0.4	6906
Eid3	0.4%	0.0%	-0.4	6907
Fam13c	0.4%	0.0%	-0.4	6908
Fbxl22	0.4%	0.0%	-0.4	6909
Fhdc1	0.4%	0.0%	-0.4	6910
Fibin	0.4%	0.0%	-0.4	6911
Fjx1	0.4%	0.0%	-0.4	6912
Foxf1	0.4%	0.0%	-0.4	6913
Fsip1	0.4%	0.0%	-0.4	6914
Gadl1	0.4%	0.0%	-0.4	6915
Gbp3	0.4%	0.0%	-0.4	6916
Gorab	0.4%	0.0%	-0.4	6917
Gpr158	0.4%	0.0%	-0.4	6918
Gprasp2	0.4%	0.0%	-0.4	6919
Gprin2	0.4%	0.0%	-0.4	6920
Gspt2	0.4%	0.0%	-0.4	6921
Haghl	0.4%	0.0%	-0.4	6922
Hmcn2	0.4%	0.0%	-0.4	6923
Hmx1	0.4%	0.0%	-0.4	6924

Hpgd	0.4%	0.0%	-0.4	6925
Hrasls	0.4%	0.0%	-0.4	6926
Il5ra	0.4%	0.0%	-0.4	6927
Kdelr3	0.4%	0.0%	-0.4	6928
Klf5	0.4%	0.0%	-0.4	6929
Klhl17	0.4%	0.0%	-0.4	6930
Klrb1c	0.4%	0.0%	-0.4	6931
Lgsn	0.4%	0.0%	-0.4	6932
Lhx4	0.4%	0.0%	-0.4	6933
Liph	0.4%	0.0%	-0.4	6934
LOC100362109	0.4%	0.0%	-0.4	6935
LOC100909464	0.4%	0.0%	-0.4	6936
LOC100909682	0.4%	0.0%	-0.4	6937
LOC100909946	0.4%	0.0%	-0.4	6938
LOC100911588	0.4%	0.0%	-0.4	6939
LOC100912221	0.4%	0.0%	-0.4	6940
LOC100912353	0.4%	0.0%	-0.4	6941
LOC102546648	0.4%	0.0%	-0.4	6942
LOC102546809	0.4%	0.0%	-0.4	6943
LOC102547216	0.4%	0.0%	-0.4	6944
LOC102547391	0.4%	0.0%	-0.4	6945
LOC102547402	0.4%	0.0%	-0.4	6946
LOC102547413	0.4%	0.0%	-0.4	6947
LOC102548264	0.4%	0.0%	-0.4	6948
LOC102548367	0.4%	0.0%	-0.4	6949
LOC102548503	0.4%	0.0%	-0.4	6950
LOC102548575	0.4%	0.0%	-0.4	6951
LOC102548637	0.4%	0.0%	-0.4	6952
LOC102548839	0.4%	0.0%	-0.4	6953
LOC102548945	0.4%	0.0%	-0.4	6954
LOC102549056	0.4%	0.0%	-0.4	6955
LOC102549277	0.4%	0.0%	-0.4	6956
LOC102549328	0.4%	0.0%	-0.4	6957
LOC102549362	0.4%	0.0%	-0.4	6958
LOC102549397	0.4%	0.0%	-0.4	6959
LOC102550008	0.4%	0.0%	-0.4	6960
LOC102550060	0.4%	0.0%	-0.4	6961
LOC102550356	0.4%	0.0%	-0.4	6962
LOC102550474	0.4%	0.0%	-0.4	6963
LOC102550498	0.4%	0.0%	-0.4	6964
LOC102550515	0.4%	0.0%	-0.4	6965
LOC102550673	0.4%	0.0%	-0.4	6966
LOC102551038	0.4%	0.0%	-0.4	6967
LOC102551266	0.4%	0.0%	-0.4	6968
LOC102552536	0.4%	0.0%	-0.4	6969
LOC102552854	0.4%	0.0%	-0.4	6970
LOC102552972	0.4%	0.0%	-0.4	6971
LOC102553001	0.4%	0.0%	-0.4	6972
LOC102553356	0.4%	0.0%	-0.4	6973
LOC102553493	0.4%	0.0%	-0.4	6974
LOC102553732	0.4%	0.0%	-0.4	6975
LOC102553738	0.4%	0.0%	-0.4	6976
LOC102553792	0.4%	0.0%	-0.4	6977
LOC102553864	0.4%	0.0%	-0.4	6978
LOC102553891	0.4%	0.0%	-0.4	6979
LOC102553935	0.4%	0.0%	-0.4	6980
LOC102554162	0.4%	0.0%	-0.4	6981
LOC102554186	0.4%	0.0%	-0.4	6982
LOC102554481	0.4%	0.0%	-0.4	6983
LOC102554599	0.4%	0.0%	-0.4	6984
LOC102554879	0.4%	0.0%	-0.4	6985
LOC102554986	0.4%	0.0%	-0.4	6986
LOC102555147	0.4%	0.0%	-0.4	6987
LOC102557372	0.4%	0.0%	-0.4	6988
LOC102557383	0.4%	0.0%	-0.4	6989
LOC102557514	0.4%	0.0%	-0.4	6990

LOC102557563	0.4%	0.0%	-0.4	6991
LOC103689995	0.4%	0.0%	-0.4	6992
LOC103690234	0.4%	0.0%	-0.4	6993
LOC103690425	0.4%	0.0%	-0.4	6994
LOC103690517	0.4%	0.0%	-0.4	6995
LOC103690591	0.4%	0.0%	-0.4	6996
LOC103691876	0.4%	0.0%	-0.4	6997
LOC103691950	0.4%	0.0%	-0.4	6998
LOC103691977	0.4%	0.0%	-0.4	6999
LOC103692166	0.4%	0.0%	-0.4	7000
LOC103692300	0.4%	0.0%	-0.4	7001
LOC103692696	0.4%	0.0%	-0.4	7002
LOC103692892	0.4%	0.0%	-0.4	7003
LOC103693236	0.4%	0.0%	-0.4	7004
LOC103693329	0.4%	0.0%	-0.4	7005
LOC103693406	0.4%	0.0%	-0.4	7006
LOC103693914	0.4%	0.0%	-0.4	7007
LOC103693916	0.4%	0.0%	-0.4	7008
LOC103694146	0.4%	0.0%	-0.4	7009
LOC103694299	0.4%	0.0%	-0.4	7010
LOC106736471	0.4%	0.0%	-0.4	7011
LOC108348059	0.4%	0.0%	-0.4	7012
LOC108348079	0.4%	0.0%	-0.4	7013
LOC108348145	0.4%	0.0%	-0.4	7014
LOC108348231	0.4%	0.0%	-0.4	7015
LOC108348825	0.4%	0.0%	-0.4	7016
LOC108348935	0.4%	0.0%	-0.4	7017
LOC108349084	0.4%	0.0%	-0.4	7018
LOC108349203	0.4%	0.0%	-0.4	7019
LOC108349207	0.4%	0.0%	-0.4	7020
LOC108349234	0.4%	0.0%	-0.4	7021
LOC108349944	0.4%	0.0%	-0.4	7022
LOC108350099	0.4%	0.0%	-0.4	7023
LOC108350102	0.4%	0.0%	-0.4	7024
LOC108350771	0.4%	0.0%	-0.4	7025
LOC108350967	0.4%	0.0%	-0.4	7026
LOC108351054	0.4%	0.0%	-0.4	7027
LOC108351212	0.4%	0.0%	-0.4	7028
LOC108351338	0.4%	0.0%	-0.4	7029
LOC108351777	0.4%	0.0%	-0.4	7030
LOC108352316	0.4%	0.0%	-0.4	7031
LOC108352353	0.4%	0.0%	-0.4	7032
LOC108352767	0.4%	0.0%	-0.4	7033
LOC108352804	0.4%	0.0%	-0.4	7034
LOC108352946	0.4%	0.0%	-0.4	7035
LOC108353166	0.4%	0.0%	-0.4	7036
LOC303140	0.4%	0.0%	-0.4	7037
LOC360479	0.4%	0.0%	-0.4	7038
LOC500028	0.4%	0.0%	-0.4	7039
LOC685574	0.4%	0.0%	-0.4	7040
LOC689303	0.4%	0.0%	-0.4	7041
Lrrc2	0.4%	0.0%	-0.4	7042
Lrrc56	0.4%	0.0%	-0.4	7043
Lrrn4	0.4%	0.0%	-0.4	7044
Lsmem2	0.4%	0.0%	-0.4	7045
Lypd2	0.4%	0.0%	-0.4	7046
Mafa	0.4%	0.0%	-0.4	7047
Map3k7cl	0.4%	0.0%	-0.4	7048
Matn4	0.4%	0.0%	-0.4	7049
Mcm8	0.4%	0.0%	-0.4	7050
Mdk	0.4%	0.0%	-0.4	7051
Mettl4	0.4%	0.0%	-0.4	7052
Mkrn2os	0.4%	0.0%	-0.4	7053
Mmp3	0.4%	0.0%	-0.4	7054
Mms22l	0.4%	0.0%	-0.4	7055
Mogat2	0.4%	0.0%	-0.4	7056

Mras	0.4%	0.0%	-0.4	7057
Mrnip	0.4%	0.0%	-0.4	7058
Myrip	0.4%	0.0%	-0.4	7059
Mzf1	0.4%	0.0%	-0.4	7060
Neto2	0.4%	0.0%	-0.4	7061
Nkx6-1	0.4%	0.0%	-0.4	7062
Nsun6	0.4%	0.0%	-0.4	7063
Nup62cl	0.4%	0.0%	-0.4	7064
Panx2	0.4%	0.0%	-0.4	7065
Pbx3	0.4%	0.0%	-0.4	7066
Pcnt	0.4%	0.0%	-0.4	7067
Pdxp	0.4%	0.0%	-0.4	7068
Plekhg2	0.4%	0.0%	-0.4	7069
Plekhh3	0.4%	0.0%	-0.4	7070
Plppr2	0.4%	0.0%	-0.4	7071
Pmepa1	0.4%	0.0%	-0.4	7072
Ppargc1b	0.4%	0.0%	-0.4	7073
Ppm1m	0.4%	0.0%	-0.4	7074
Ppp1r14c	0.4%	0.0%	-0.4	7075
Prdm15	0.4%	0.0%	-0.4	7076
Ptprh	0.4%	0.0%	-0.4	7077
Ptprm	0.4%	0.0%	-0.4	7078
Rasa2	0.4%	0.0%	-0.4	7079
Rasal2	0.4%	0.0%	-0.4	7080
Rcan3	0.4%	0.0%	-0.4	7081
RGD1310429	0.4%	0.0%	-0.4	7082
RGD1560436	0.4%	0.0%	-0.4	7083
RGD1560730	0.4%	0.0%	-0.4	7084
RGD1561231	0.4%	0.0%	-0.4	7085
RGD1561282	0.4%	0.0%	-0.4	7086
RGD1563354	0.4%	0.0%	-0.4	7087
Rnase1l1	0.4%	0.0%	-0.4	7088
Rom1	0.4%	0.0%	-0.4	7089
Rpusd2	0.4%	0.0%	-0.4	7090
Rsg1	0.4%	0.0%	-0.4	7091
Rtn2	0.4%	0.0%	-0.4	7092
Ryr1	0.4%	0.0%	-0.4	7093
Scai	0.4%	0.0%	-0.4	7094
Scube3	0.4%	0.0%	-0.4	7095
Sema3b	0.4%	0.0%	-0.4	7096
Sh3bgr	0.4%	0.0%	-0.4	7097
Sh3pxd2b	0.4%	0.0%	-0.4	7098
Slc26a2	0.4%	0.0%	-0.4	7099
Slc28a1	0.4%	0.0%	-0.4	7100
Slc29a2	0.4%	0.0%	-0.4	7101
Slc2a4	0.4%	0.0%	-0.4	7102
Slc7a6	0.4%	0.0%	-0.4	7103
Smo	0.4%	0.0%	-0.4	7104
Sprr1a	0.4%	0.0%	-0.4	7105
St8sia1	0.4%	0.0%	-0.4	7106
Stc1	0.4%	0.0%	-0.4	7107
Stx11	0.4%	0.0%	-0.4	7108
Sybu	0.4%	0.0%	-0.4	7109
Syce3	0.4%	0.0%	-0.4	7110
Terb1	0.4%	0.0%	-0.4	7111
Thnsl1	0.4%	0.0%	-0.4	7112
Ticam2	0.4%	0.0%	-0.4	7113
Tmem100	0.4%	0.0%	-0.4	7114
Tmprss4	0.4%	0.0%	-0.4	7115
Trim80	0.4%	0.0%	-0.4	7116
Trmt11	0.4%	0.0%	-0.4	7117
Ttc9	0.4%	0.0%	-0.4	7118
Ttll3	0.4%	0.0%	-0.4	7119
Tubgcp6	0.4%	0.0%	-0.4	7120
Ubox5	0.4%	0.0%	-0.4	7121
Usp11	0.4%	0.0%	-0.4	7122

Wdr47	0.4%	0.0%	-0.4	7123
Wdr54	0.4%	0.0%	-0.4	7124
Ypel1	0.4%	0.0%	-0.4	7125
Zcchc12	0.4%	0.0%	-0.4	7126
Zfhx2	0.4%	0.0%	-0.4	7127
Zfp287	0.4%	0.0%	-0.4	7128
Zfp3	0.4%	0.0%	-0.4	7129
Zfp575	0.4%	0.0%	-0.4	7130
Zfp583	0.4%	0.0%	-0.4	7131
Zfp879	0.4%	0.0%	-0.4	7132
Zglp1	0.4%	0.0%	-0.4	7133
Parp12	4.3%	3.9%	-0.4	7134
U2surp	4.3%	3.9%	-0.4	7135
Dixdc1	4.3%	3.9%	-0.4	7136
Tmpo	4.3%	3.9%	-0.4	7137
Selenop	100.0%	99.6%	-0.4	7138
Stip1	8.2%	7.8%	-0.4	7139
Itga5	3.9%	3.5%	-0.4	7140
Zcchc11	3.9%	3.5%	-0.4	7141
LOC100359583	3.9%	3.5%	-0.4	7142
Tmem39a	3.9%	3.5%	-0.4	7143
Repin1	3.1%	2.7%	-0.4	7144
Tmod1	3.1%	2.7%	-0.4	7145
LOC100909849	3.1%	2.7%	-0.4	7146
Wdr45b	3.1%	2.7%	-0.4	7147
RGD1560464	3.1%	2.7%	-0.4	7148
Lsm10	2.7%	2.3%	-0.4	7149
LOC108349805	2.7%	2.3%	-0.4	7150
Gyg1	2.7%	2.3%	-0.4	7151
Zkscan3	2.7%	2.3%	-0.4	7152
Zcchc7	2.7%	2.3%	-0.4	7153
Slc12a2	2.4%	1.9%	-0.4	7154
Arl6ip6	2.4%	1.9%	-0.4	7155
LOC100910057	2.4%	1.9%	-0.4	7156
RGD1563991	2.0%	1.5%	-0.4	7157
Naa16	2.0%	1.5%	-0.4	7158
Rbm48	2.0%	1.5%	-0.4	7159
Snx17	2.0%	1.5%	-0.4	7160
Frat1	2.0%	1.5%	-0.4	7161
Fabp2	2.0%	1.5%	-0.4	7162
Ercc6	2.0%	1.5%	-0.4	7163
Cln3	2.0%	1.5%	-0.4	7164
Gpn2	2.0%	1.5%	-0.4	7165
LOC500881	2.0%	1.5%	-0.4	7166
Dnajc14	2.0%	1.5%	-0.4	7167
Tom1l2	2.0%	1.5%	-0.4	7168
Znhit3	2.0%	1.5%	-0.4	7169
Gxylt1	2.0%	1.5%	-0.4	7170
Ap3s2	5.9%	5.4%	-0.4	7171
Ikbkg	1.6%	1.1%	-0.4	7172
Rabl2a	1.6%	1.1%	-0.4	7173
Ankrd37	1.6%	1.1%	-0.4	7174
Bcl6b	1.6%	1.1%	-0.4	7175
Arrdc2	1.6%	1.1%	-0.4	7176
Phgdh	1.6%	1.1%	-0.4	7177
Nectin1	1.6%	1.1%	-0.4	7178
Gtpbp6	1.6%	1.1%	-0.4	7179
Zbtb6	1.6%	1.1%	-0.4	7180
Lemd2	1.6%	1.1%	-0.4	7181
E2f2	1.6%	1.1%	-0.4	7182
Gon4l	1.6%	1.1%	-0.4	7183
Unc93b1	1.6%	1.1%	-0.4	7184
Cic	1.6%	1.1%	-0.4	7185
Sbf2	1.6%	1.1%	-0.4	7186
Cyp17a1	1.6%	1.1%	-0.4	7187
LOC102549942	1.6%	1.1%	-0.4	7188

Dus2	1.6%	1.1%	-0.4	7189
LOC102557384	1.6%	1.1%	-0.4	7190
Unc119	1.6%	1.1%	-0.4	7191
LOC103693608	1.6%	1.1%	-0.4	7192
LOC100912622	1.6%	1.1%	-0.4	7193
Fbrsl1	1.6%	1.1%	-0.4	7194
Rprd1a	1.6%	1.1%	-0.4	7195
NEWGENE_1310139	1.6%	1.1%	-0.4	7196
Eif2ak2	5.5%	5.0%	-0.5	7197
Rit1	1.2%	0.7%	-0.5	7198
Adck4	1.2%	0.7%	-0.5	7199
Slc18b1	1.2%	0.7%	-0.5	7200
LOC100911233	1.2%	0.7%	-0.5	7201
Gpatch2l	1.2%	0.7%	-0.5	7202
Pold2	1.2%	0.7%	-0.5	7203
Ccar2	1.2%	0.7%	-0.5	7204
Ppp1r37	1.2%	0.7%	-0.5	7205
Dtx3	1.2%	0.7%	-0.5	7206
Sgk3	1.2%	0.7%	-0.5	7207
Kif3b	1.2%	0.7%	-0.5	7208
Slc12a9	1.2%	0.7%	-0.5	7209
LOC103690343	1.2%	0.7%	-0.5	7210
Lysmd1	1.2%	0.7%	-0.5	7211
LOC102548266	1.2%	0.7%	-0.5	7212
Trrap	1.2%	0.7%	-0.5	7213
Mtx3	1.2%	0.7%	-0.5	7214
Rfx7	1.2%	0.7%	-0.5	7215
LOC103694506	1.2%	0.7%	-0.5	7216
Paqr4	1.2%	0.7%	-0.5	7217
Rbms2	1.2%	0.7%	-0.5	7218
Nsmf	1.2%	0.7%	-0.5	7219
LOC100359876	9.4%	9.0%	-0.5	7220
Lamtor1	9.4%	9.0%	-0.5	7221
LOC103690068	5.1%	4.6%	-0.5	7222
Gtpbp8	0.8%	0.3%	-0.5	7223
LOC103690357	0.8%	0.3%	-0.5	7224
Kctd21	0.8%	0.3%	-0.5	7225
Acaca	0.8%	0.3%	-0.5	7226
Stam2	0.8%	0.3%	-0.5	7227
P2ry1	0.8%	0.3%	-0.5	7228
Zfp646	0.8%	0.3%	-0.5	7229
Med26	0.8%	0.3%	-0.5	7230
Frmd8	0.8%	0.3%	-0.5	7231
Slc30a3	0.8%	0.3%	-0.5	7232
LOC680254	0.8%	0.3%	-0.5	7233
Urb2	0.8%	0.3%	-0.5	7234
Zfp455	0.8%	0.3%	-0.5	7235
LOC102553772	0.8%	0.3%	-0.5	7236
LOC682206	0.8%	0.3%	-0.5	7237
Capn10	0.8%	0.3%	-0.5	7238
Slc39a6	0.8%	0.3%	-0.5	7239
LOC102554620	0.8%	0.3%	-0.5	7240
LOC108351928	0.8%	0.3%	-0.5	7241
Pou3f1	0.8%	0.3%	-0.5	7242
Zdhhc21	0.8%	0.3%	-0.5	7243
Nek1	0.8%	0.3%	-0.5	7244
Rbm41	0.8%	0.3%	-0.5	7245
LOC102554965	0.8%	0.3%	-0.5	7246
Kank3	0.8%	0.3%	-0.5	7247
Phka2	0.8%	0.3%	-0.5	7248
LOC103694878	0.8%	0.3%	-0.5	7249
Slc35a2	0.8%	0.3%	-0.5	7250
Znf750	0.8%	0.3%	-0.5	7251
LOC103691745	0.8%	0.3%	-0.5	7252
Gba2	0.8%	0.3%	-0.5	7253
Pias3	0.8%	0.3%	-0.5	7254

Tango6	0.8%	0.3%	-0.5	7255
Fam185a	0.8%	0.3%	-0.5	7256
Nckap5	0.8%	0.3%	-0.5	7257
LOC100363361	0.8%	0.3%	-0.5	7258
Dlg1	0.8%	0.3%	-0.5	7259
Mapk7	0.8%	0.3%	-0.5	7260
LOC102550889	0.8%	0.3%	-0.5	7261
Fem1b	0.8%	0.3%	-0.5	7262
LOC102550657	0.8%	0.3%	-0.5	7263
Shprh	0.8%	0.3%	-0.5	7264
LOC103693338	0.8%	0.3%	-0.5	7265
Dopey1	0.8%	0.3%	-0.5	7266
Mycl	0.8%	0.3%	-0.5	7267
Gramd1c	0.8%	0.3%	-0.5	7268
LOC689488	0.8%	0.3%	-0.5	7269
LOC102554398	0.8%	0.3%	-0.5	7270
Atxn7l2	0.8%	0.3%	-0.5	7271
LOC100360750	4.7%	4.2%	-0.5	7272
Gc	100.0%	99.5%	-0.5	7273
Clstn1	3.5%	3.0%	-0.5	7274
Stxbp2	3.5%	3.0%	-0.5	7275
LOC100910688	3.5%	3.0%	-0.5	7276
LOC102546833	3.1%	2.6%	-0.5	7277
Stk19	3.1%	2.6%	-0.5	7278
Zfp385a	3.1%	2.6%	-0.5	7279
Myo1b	3.1%	2.6%	-0.5	7280
Tbc1d23	3.1%	2.6%	-0.5	7281
MGC114499	3.1%	2.6%	-0.5	7282
Cd99l2	2.7%	2.2%	-0.5	7283
Cgn	2.7%	2.2%	-0.5	7284
Me3	2.7%	2.2%	-0.5	7285
Kmt2c	2.7%	2.2%	-0.5	7286
Inf2	2.7%	2.2%	-0.5	7287
Mettl16	2.4%	1.8%	-0.5	7288
Lancl2	2.4%	1.8%	-0.5	7289
Atg14	2.4%	1.8%	-0.5	7290
Exoc7	2.4%	1.8%	-0.5	7291
Orc5	2.0%	1.4%	-0.5	7292
Arhgef26	2.0%	1.4%	-0.5	7293
Marf1	2.0%	1.4%	-0.5	7294
Mfhas1	2.0%	1.4%	-0.5	7295
Zscan12	2.0%	1.4%	-0.5	7296
Chpf	2.0%	1.4%	-0.5	7297
Tns3	2.0%	1.4%	-0.5	7298
Cdc23	2.0%	1.4%	-0.5	7299
Ndufaf1	2.0%	1.4%	-0.5	7300
LOC102548271	2.0%	1.4%	-0.5	7301
Adam15	2.0%	1.4%	-0.5	7302
Taf1	2.0%	1.4%	-0.5	7303
Zdhhc17	2.0%	1.4%	-0.5	7304
LOC102557436	1.6%	1.0%	-0.5	7305
Acvr1	1.6%	1.0%	-0.5	7306
Smc5	1.6%	1.0%	-0.5	7307
Slc25a12	1.6%	1.0%	-0.5	7308
Dcaf7	1.6%	1.0%	-0.5	7309
Ephb4	1.6%	1.0%	-0.5	7310
Foxn2	1.6%	1.0%	-0.5	7311
Plekhg3	1.6%	1.0%	-0.5	7312
Cwc27	1.6%	1.0%	-0.5	7313
Wdr81	1.6%	1.0%	-0.5	7314
Amigo2	1.6%	1.0%	-0.5	7315
Itga1	1.6%	1.0%	-0.5	7316
Gfra3	1.6%	1.0%	-0.5	7317
Smc2	1.6%	1.0%	-0.5	7318
Apbb2	1.6%	1.0%	-0.5	7319
Caln1	1.6%	1.0%	-0.5	7320

Cbwd1	1.6%	1.0%	-0.5	7321
Mettl2b	1.6%	1.0%	-0.5	7322
Apc	1.6%	1.0%	-0.5	7323
LOC100911266	1.6%	1.0%	-0.5	7324
Borcs5	1.6%	1.0%	-0.5	7325
Ints9	1.6%	1.0%	-0.5	7326
Pdxdc1	1.6%	1.0%	-0.5	7327
Hspbap1	1.6%	1.0%	-0.5	7328
Glg1	1.6%	1.0%	-0.5	7329
LOC100910854	1.6%	1.0%	-0.5	7330
LOC100909928	1.6%	1.0%	-0.5	7331
Atad2	1.6%	1.0%	-0.5	7332
Cep57	1.6%	1.0%	-0.5	7333
Tp53	9.8%	9.3%	-0.5	7334
Ubr4	5.5%	5.0%	-0.5	7335
Lsm4	5.5%	5.0%	-0.5	7336
Ppp1r26	1.2%	0.6%	-0.5	7337
Mllt11	1.2%	0.6%	-0.5	7338
Golt1b	1.2%	0.6%	-0.5	7339
Szt2	1.2%	0.6%	-0.5	7340
Nrf1	1.2%	0.6%	-0.5	7341
Pitpnm1	1.2%	0.6%	-0.5	7342
Dyrk1b	1.2%	0.6%	-0.5	7343
Pkd1	1.2%	0.6%	-0.5	7344
Abcc4	1.2%	0.6%	-0.5	7345
Cdhr3	1.2%	0.6%	-0.5	7346
Enox2	1.2%	0.6%	-0.5	7347
Homez	1.2%	0.6%	-0.5	7348
Zfhx4	1.2%	0.6%	-0.5	7349
Rnf32	1.2%	0.6%	-0.5	7350
Cyyr1	1.2%	0.6%	-0.5	7351
Fam118b	1.2%	0.6%	-0.5	7352
Mboat1	1.2%	0.6%	-0.5	7353
LOC690120	1.2%	0.6%	-0.5	7354
Armcx1	1.2%	0.6%	-0.5	7355
LOC102549615	1.2%	0.6%	-0.5	7356
Chst9	1.2%	0.6%	-0.5	7357
Tbp	1.2%	0.6%	-0.5	7358
Gtf2e1	1.2%	0.6%	-0.5	7359
Cerk	1.2%	0.6%	-0.5	7360
Fermt3	1.2%	0.6%	-0.5	7361
LOC679604	5.1%	4.6%	-0.5	7362
Hs3st3b1	0.8%	0.2%	-0.5	7363
Dclre1c	0.8%	0.2%	-0.5	7364
Syt3	0.8%	0.2%	-0.5	7365
LOC102549967	0.8%	0.2%	-0.5	7366
Cep78	0.8%	0.2%	-0.5	7367
Pcbp3	0.8%	0.2%	-0.5	7368
Exosc2	0.8%	0.2%	-0.5	7369
LOC108349648	0.8%	0.2%	-0.5	7370
LOC102551485	0.8%	0.2%	-0.5	7371
Arhgap32	0.8%	0.2%	-0.5	7372
LOC103694063	0.8%	0.2%	-0.5	7373
Stk38l	0.8%	0.2%	-0.5	7374
Zfp202	0.8%	0.2%	-0.5	7375
Fkbp7	0.8%	0.2%	-0.5	7376
Numb	0.8%	0.2%	-0.5	7377
Zfp322a	0.8%	0.2%	-0.5	7378
LOC687035	0.8%	0.2%	-0.5	7379
Dennd4b	0.8%	0.2%	-0.5	7380
Zswim1	0.8%	0.2%	-0.5	7381
LOC100912585	0.8%	0.2%	-0.5	7382
Tnnc2	0.8%	0.2%	-0.5	7383
LOC685590	0.8%	0.2%	-0.5	7384
Fam110c	0.8%	0.2%	-0.5	7385
LOC102555635	0.8%	0.2%	-0.5	7386

Sgcb	0.8%	0.2%	-0.5	7387
LOC100360470	0.8%	0.2%	-0.5	7388
Fbxo46	0.8%	0.2%	-0.5	7389
LOC102554337	0.8%	0.2%	-0.5	7390
Rn7sl1	0.8%	0.2%	-0.5	7391
LOC102552448	0.8%	0.2%	-0.5	7392
Phf8	0.8%	0.2%	-0.5	7393
Purg	0.8%	0.2%	-0.5	7394
Cep250	0.8%	0.2%	-0.5	7395
Fgd1	0.8%	0.2%	-0.5	7396
Tyw3	0.8%	0.2%	-0.5	7397
LOC691290	0.8%	0.2%	-0.5	7398
Camk1d	0.8%	0.2%	-0.5	7399
Abca5	0.8%	0.2%	-0.5	7400
LOC108352416	0.8%	0.2%	-0.5	7401
Cntln	0.8%	0.2%	-0.5	7402
Med12l	0.8%	0.2%	-0.5	7403
Cep70	0.8%	0.2%	-0.5	7404
Samd10	0.8%	0.2%	-0.5	7405
Zfp955a	0.8%	0.2%	-0.5	7406
Rad9a	0.8%	0.2%	-0.5	7407
Il36b	0.8%	0.2%	-0.5	7408
Adam11	0.8%	0.2%	-0.5	7409
LOC108350110	0.8%	0.2%	-0.5	7410
Zfp866	0.8%	0.2%	-0.5	7411
Capn15	0.8%	0.2%	-0.5	7412
Cep41	0.8%	0.2%	-0.5	7413
Rrnad1	0.8%	0.2%	-0.5	7414
Mast3	0.8%	0.2%	-0.5	7415
Osgin2	0.8%	0.2%	-0.5	7416
Gatsl2	0.8%	0.2%	-0.5	7417
Zfp142	0.8%	0.2%	-0.5	7418
Zscan30	0.8%	0.2%	-0.5	7419
Tox3	0.8%	0.2%	-0.5	7420
Stx3	0.8%	0.2%	-0.5	7421
LOC102550588	0.8%	0.2%	-0.5	7422
Slc13a5	0.8%	0.2%	-0.5	7423
Ccna2	0.8%	0.2%	-0.5	7424
Bhlhb9	0.8%	0.2%	-0.5	7425
Acbd7	0.8%	0.2%	-0.5	7426
Apobec1	4.7%	4.2%	-0.5	7427
Sf3a3	4.7%	4.2%	-0.5	7428
Pkig	4.3%	3.8%	-0.6	7429
Necap2	4.3%	3.8%	-0.6	7430
Yrdc	4.3%	3.8%	-0.6	7431
Rab8a	8.2%	7.7%	-0.6	7432
Cwf19l2	3.9%	3.4%	-0.6	7433
Mpg	3.5%	3.0%	-0.6	7434
Abhd12	3.5%	3.0%	-0.6	7435
Mcrip1	3.5%	3.0%	-0.6	7436
Morn2	3.1%	2.6%	-0.6	7437
Svip	3.1%	2.6%	-0.6	7438
Hyou1	3.1%	2.6%	-0.6	7439
Rpa1	2.7%	2.2%	-0.6	7440
LOC103694460	2.7%	2.2%	-0.6	7441
LOC102554622	2.7%	2.2%	-0.6	7442
Plgrkt	2.7%	2.2%	-0.6	7443
Ubac1	2.7%	2.2%	-0.6	7444
Unk	2.7%	2.2%	-0.6	7445
Aph1b	2.7%	2.2%	-0.6	7446
Coq10a	2.7%	2.2%	-0.6	7447
Mta3	2.7%	2.2%	-0.6	7448
Supt16h	2.7%	2.2%	-0.6	7449
Ptpn1	2.7%	2.2%	-0.6	7450
Dlc1	2.7%	2.2%	-0.6	7451
Pde3b	2.7%	2.2%	-0.6	7452

LOC108348064	2.7%	2.2%	-0.6	7453
Thrb	2.4%	1.8%	-0.6	7454
Patj	2.4%	1.8%	-0.6	7455
Senp3	2.4%	1.8%	-0.6	7456
Nedd9	6.3%	5.7%	-0.6	7457
F3	2.0%	1.4%	-0.6	7458
Znf740	2.0%	1.4%	-0.6	7459
Cenpl	2.0%	1.4%	-0.6	7460
Atp8a1	2.0%	1.4%	-0.6	7461
Arhgap24	2.0%	1.4%	-0.6	7462
Ildr2	2.0%	1.4%	-0.6	7463
Map9	2.0%	1.4%	-0.6	7464
Git2	2.0%	1.4%	-0.6	7465
Bckdk	5.9%	5.3%	-0.6	7466
LOC100363642	1.6%	1.0%	-0.6	7467
Inpp5f	1.6%	1.0%	-0.6	7468
Strn	1.6%	1.0%	-0.6	7469
Ddx51	1.6%	1.0%	-0.6	7470
Nr4a2	1.6%	1.0%	-0.6	7471
Mtpn	1.6%	1.0%	-0.6	7472
Phf12	1.6%	1.0%	-0.6	7473
RGD1563962	1.6%	1.0%	-0.6	7474
Aurka	1.6%	1.0%	-0.6	7475
Shf	1.6%	1.0%	-0.6	7476
LOC100909729	1.6%	1.0%	-0.6	7477
Ice1	1.6%	1.0%	-0.6	7478
Tsc2	1.6%	1.0%	-0.6	7479
Per2	1.6%	1.0%	-0.6	7480
LOC100909856	1.6%	1.0%	-0.6	7481
Tfdp2	1.6%	1.0%	-0.6	7482
Ccni	9.8%	9.2%	-0.6	7483
Ctsz	5.5%	4.9%	-0.6	7484
Prr14l	1.2%	0.6%	-0.6	7485
Rrp1b	1.2%	0.6%	-0.6	7486
LOC108351966	1.2%	0.6%	-0.6	7487
Naa40	1.2%	0.6%	-0.6	7488
LOC108348169	1.2%	0.6%	-0.6	7489
Arhgef11	1.2%	0.6%	-0.6	7490
Klhl36	1.2%	0.6%	-0.6	7491
Tuft1	1.2%	0.6%	-0.6	7492
Zbtb39	1.2%	0.6%	-0.6	7493
LOC103690173	1.2%	0.6%	-0.6	7494
Rab3d	1.2%	0.6%	-0.6	7495
Fgf1	1.2%	0.6%	-0.6	7496
Extl3	1.2%	0.6%	-0.6	7497
LOC100125367	1.2%	0.6%	-0.6	7498
Fam76b	1.2%	0.6%	-0.6	7499
Zfp41	1.2%	0.6%	-0.6	7500
Rpusd1	1.2%	0.6%	-0.6	7501
Serf1	1.2%	0.6%	-0.6	7502
Jmjd7	1.2%	0.6%	-0.6	7503
Etaa1	1.2%	0.6%	-0.6	7504
NEWGENE_621802	1.2%	0.6%	-0.6	7505
Mettl8	1.2%	0.6%	-0.6	7506
Dusp28	1.2%	0.6%	-0.6	7507
Ccdc71l	1.2%	0.6%	-0.6	7508
LOC100909869	1.2%	0.6%	-0.6	7509
Cdon	1.2%	0.6%	-0.6	7510
Zc3h11a	1.2%	0.6%	-0.6	7511
Tbc1d1	1.2%	0.6%	-0.6	7512
Ncoa3	1.2%	0.6%	-0.6	7513
LOC682357	1.2%	0.6%	-0.6	7514
Sort1	1.2%	0.6%	-0.6	7515
Tmtc3	1.2%	0.6%	-0.6	7516
Haus4	1.2%	0.6%	-0.6	7517
LOC100910829	5.1%	4.5%	-0.6	7518

Hyls1	0.8%	0.2%	-0.6	7519
LOC102552355	0.8%	0.2%	-0.6	7520
Hbegf	0.8%	0.2%	-0.6	7521
Bcdin3d	0.8%	0.2%	-0.6	7522
Dusp14l1	0.8%	0.2%	-0.6	7523
LOC102548334	0.8%	0.2%	-0.6	7524
LOC108352752	0.8%	0.2%	-0.6	7525
Carf	0.8%	0.2%	-0.6	7526
Fer1l5	0.8%	0.2%	-0.6	7527
Sacs	0.8%	0.2%	-0.6	7528
Kiss1	0.8%	0.2%	-0.6	7529
Nt5m	0.8%	0.2%	-0.6	7530
Kdm4c	0.8%	0.2%	-0.6	7531
LOC100363171	0.8%	0.2%	-0.6	7532
Fndc10	0.8%	0.2%	-0.6	7533
Crybg3	0.8%	0.2%	-0.6	7534
Klhl42	0.8%	0.2%	-0.6	7535
Spag1	0.8%	0.2%	-0.6	7536
Arhgef40	0.8%	0.2%	-0.6	7537
Krtcap3	0.8%	0.2%	-0.6	7538
LOC102546997	0.8%	0.2%	-0.6	7539
Zfp964	0.8%	0.2%	-0.6	7540
LOC102553736	0.8%	0.2%	-0.6	7541
LOC100911489	0.8%	0.2%	-0.6	7542
Rflnb	0.8%	0.2%	-0.6	7543
Zfp846	0.8%	0.2%	-0.6	7544
LOC108351970	0.8%	0.2%	-0.6	7545
Osbpl3	0.8%	0.2%	-0.6	7546
LOC102557083	0.8%	0.2%	-0.6	7547
Slc22a15	0.8%	0.2%	-0.6	7548
Tmem200b	0.8%	0.2%	-0.6	7549
LOC103691014	0.8%	0.2%	-0.6	7550
Inpp5e	0.8%	0.2%	-0.6	7551
Uvssa	0.8%	0.2%	-0.6	7552
LOC108353494	0.8%	0.2%	-0.6	7553
LOC102553965	0.8%	0.2%	-0.6	7554
Eva1c	0.8%	0.2%	-0.6	7555
Lrrc8e	0.8%	0.2%	-0.6	7556
Arsg	0.8%	0.2%	-0.6	7557
LOC108351866	0.8%	0.2%	-0.6	7558
Myom1	0.8%	0.2%	-0.6	7559
Rbm12b	0.8%	0.2%	-0.6	7560
LOC498675	0.8%	0.2%	-0.6	7561
Cenpq	0.8%	0.2%	-0.6	7562
LOC102553902	0.8%	0.2%	-0.6	7563
Dnah9	0.8%	0.2%	-0.6	7564
Dcbld2	0.8%	0.2%	-0.6	7565
Palm	0.8%	0.2%	-0.6	7566
LOC108351299	0.8%	0.2%	-0.6	7567
LOC691354	0.8%	0.2%	-0.6	7568
C2cd4d	0.8%	0.2%	-0.6	7569
Ccdc130	0.8%	0.2%	-0.6	7570
Ntrk1	0.8%	0.2%	-0.6	7571
Wnt7a	0.8%	0.2%	-0.6	7572
Kdm4b	0.8%	0.2%	-0.6	7573
Lrrk1	0.8%	0.2%	-0.6	7574
Ahdc1	0.8%	0.2%	-0.6	7575
Dgkz	4.7%	4.1%	-0.6	7576
Dctn6	4.7%	4.1%	-0.6	7577
Polr3d	4.3%	3.7%	-0.6	7578
Rps6ka1	4.3%	3.7%	-0.6	7579
LOC100912870	100.0%	99.4%	-0.6	7580
Gfpt1	3.9%	3.3%	-0.6	7581
Prpf38b	7.8%	7.2%	-0.6	7582
Ddhd1	3.5%	2.9%	-0.7	7583
LOC691658	3.5%	2.9%	-0.7	7584

Pnn	3.5%	2.9%	-0.7	7585
Tlr3	3.1%	2.5%	-0.7	7586
Sbno2	3.1%	2.5%	-0.7	7587
LOC102548514	3.1%	2.5%	-0.7	7588
Baz2b	3.1%	2.5%	-0.7	7589
LOC103689964	3.1%	2.5%	-0.7	7590
LOC102555167	7.1%	6.4%	-0.7	7591
Triobp	2.7%	2.1%	-0.7	7592
Taok3	2.7%	2.1%	-0.7	7593
Phospho2	2.7%	2.1%	-0.7	7594
Skiv2l2	2.7%	2.1%	-0.7	7595
Sart3	2.7%	2.1%	-0.7	7596
Ldb1	2.7%	2.1%	-0.7	7597
Zfp146	2.7%	2.1%	-0.7	7598
Ccdc126	2.7%	2.1%	-0.7	7599
Gosr1	2.7%	2.1%	-0.7	7600
Ids	2.7%	2.1%	-0.7	7601
Polb	2.7%	2.1%	-0.7	7602
Chd1l	6.7%	6.0%	-0.7	7603
Plce1	2.4%	1.7%	-0.7	7604
Efl1	2.4%	1.7%	-0.7	7605
Cc2d1b	2.4%	1.7%	-0.7	7606
Chtop	2.4%	1.7%	-0.7	7607
Ifngr1	2.4%	1.7%	-0.7	7608
Tfpt	2.4%	1.7%	-0.7	7609
Eea1	2.4%	1.7%	-0.7	7610
Ing3	2.4%	1.7%	-0.7	7611
Dennd6a	2.4%	1.7%	-0.7	7612
Pfkfb3	2.4%	1.7%	-0.7	7613
Fgfr1op2	10.6%	9.9%	-0.7	7614
Etv3	2.0%	1.3%	-0.7	7615
Slc38a9	2.0%	1.3%	-0.7	7616
LOC103689943	2.0%	1.3%	-0.7	7617
RGD1311847	2.0%	1.3%	-0.7	7618
Traf3	2.0%	1.3%	-0.7	7619
Foxk2	2.0%	1.3%	-0.7	7620
Nagk	2.0%	1.3%	-0.7	7621
Ercc8	2.0%	1.3%	-0.7	7622
Wdfy3	2.0%	1.3%	-0.7	7623
Zfp451	1.6%	0.9%	-0.7	7624
Zmiz2	1.6%	0.9%	-0.7	7625
Lrif1	1.6%	0.9%	-0.7	7626
Dcp1b	1.6%	0.9%	-0.7	7627
LOC100912823	1.6%	0.9%	-0.7	7628
Rad50	1.6%	0.9%	-0.7	7629
Cdyl	1.6%	0.9%	-0.7	7630
Hhipl1	1.6%	0.9%	-0.7	7631
Lppos	1.6%	0.9%	-0.7	7632
Prkca	1.6%	0.9%	-0.7	7633
Cbr1	1.6%	0.9%	-0.7	7634
Acap2	1.6%	0.9%	-0.7	7635
Bbs1	1.6%	0.9%	-0.7	7636
LOC102555066	1.6%	0.9%	-0.7	7637
RGD1308742	1.6%	0.9%	-0.7	7638
LOC100910143	1.6%	0.9%	-0.7	7639
Cpsf3l	1.6%	0.9%	-0.7	7640
Rasgrp2	1.6%	0.9%	-0.7	7641
Cramp1	1.6%	0.9%	-0.7	7642
Sdr39u1	1.6%	0.9%	-0.7	7643
Coil	1.2%	0.5%	-0.7	7644
Acap3	1.2%	0.5%	-0.7	7645
Zfp512b	1.2%	0.5%	-0.7	7646
Zfp608	1.2%	0.5%	-0.7	7647
Lcorl	1.2%	0.5%	-0.7	7648
Tug1	1.2%	0.5%	-0.7	7649
Amot	1.2%	0.5%	-0.7	7650

RGD1560398	1.2%	0.5%	-0.7	7651
Zfand4	1.2%	0.5%	-0.7	7652
Tdrd3	1.2%	0.5%	-0.7	7653
Ift88	1.2%	0.5%	-0.7	7654
Dnmt3b	1.2%	0.5%	-0.7	7655
LOC103693265	1.2%	0.5%	-0.7	7656
LOC100911692	1.2%	0.5%	-0.7	7657
LOC102549611	1.2%	0.5%	-0.7	7658
Senp7	1.2%	0.5%	-0.7	7659
Lipt2	1.2%	0.5%	-0.7	7660
LOC100911196	1.2%	0.5%	-0.7	7661
Morc4	1.2%	0.5%	-0.7	7662
Ears2l1	1.2%	0.5%	-0.7	7663
LOC103690139	1.2%	0.5%	-0.7	7664
Ganc	1.2%	0.5%	-0.7	7665
Slc27a4	1.2%	0.5%	-0.7	7666
Serac1	1.2%	0.5%	-0.7	7667
Crtc1	1.2%	0.5%	-0.7	7668
Mllt3	1.2%	0.5%	-0.7	7669
Irak1bp1	1.2%	0.5%	-0.7	7670
LOC102548917	1.2%	0.5%	-0.7	7671
Adcy9	1.2%	0.5%	-0.7	7672
LOC103693493	1.2%	0.5%	-0.7	7673
Banp	1.2%	0.5%	-0.7	7674
Brat1	1.2%	0.5%	-0.7	7675
LOC108348167	1.2%	0.5%	-0.7	7676
Slc9a6	1.2%	0.5%	-0.7	7677
Mlh1	1.2%	0.5%	-0.7	7678
Ccdc93	1.2%	0.5%	-0.7	7679
Erf	5.1%	4.4%	-0.7	7680
Slc39a9	0.8%	0.1%	-0.7	7681
Tbc1d16	0.8%	0.1%	-0.7	7682
Rab11fip2	0.8%	0.1%	-0.7	7683
LOC102551726	0.8%	0.1%	-0.7	7684
LOC108348302	0.8%	0.1%	-0.7	7685
Rnf219	0.8%	0.1%	-0.7	7686
LOC102547321	0.8%	0.1%	-0.7	7687
Calr3	0.8%	0.1%	-0.7	7688
Zbtb49	0.8%	0.1%	-0.7	7689
LOC103690019	0.8%	0.1%	-0.7	7690
LOC102552823	0.8%	0.1%	-0.7	7691
LOC108351448	0.8%	0.1%	-0.7	7692
LOC100912188	0.8%	0.1%	-0.7	7693
Hectd4	0.8%	0.1%	-0.7	7694
Ros1	0.8%	0.1%	-0.7	7695
Srms	0.8%	0.1%	-0.7	7696
Trim69	0.8%	0.1%	-0.7	7697
LOC690319	0.8%	0.1%	-0.7	7698
RGD1560883	0.8%	0.1%	-0.7	7699
Arhgap4	0.8%	0.1%	-0.7	7700
LOC108350736	0.8%	0.1%	-0.7	7701
Lpar2	0.8%	0.1%	-0.7	7702
LOC108352420	0.8%	0.1%	-0.7	7703
Rrp8	0.8%	0.1%	-0.7	7704
Sp5	0.8%	0.1%	-0.7	7705
LOC102555817	0.8%	0.1%	-0.7	7706
Ptges3l	0.8%	0.1%	-0.7	7707
LOC102555191	0.8%	0.1%	-0.7	7708
LOC108348591	0.8%	0.1%	-0.7	7709
Phldb1	0.8%	0.1%	-0.7	7710
Nos1ap	0.8%	0.1%	-0.7	7711
Cib2	0.8%	0.1%	-0.7	7712
Atp8b4	0.8%	0.1%	-0.7	7713
RGD1305733	0.8%	0.1%	-0.7	7714
LOC102551211	0.8%	0.1%	-0.7	7715
Slc26a11	0.8%	0.1%	-0.7	7716

LOC108350783	0.8%	0.1%	-0.7	7717
Apcdd1	0.8%	0.1%	-0.7	7718
Cndp1	0.8%	0.1%	-0.7	7719
LOC102550892	0.8%	0.1%	-0.7	7720
LOC102554368	0.8%	0.1%	-0.7	7721
Prkce	0.8%	0.1%	-0.7	7722
Pigq	0.8%	0.1%	-0.7	7723
Dock3	0.8%	0.1%	-0.7	7724
LOC108351723	0.8%	0.1%	-0.7	7725
Zcchc3	0.8%	0.1%	-0.7	7726
LOC103691283	0.8%	0.1%	-0.7	7727
Eomes	0.8%	0.1%	-0.7	7728
Spata33	0.8%	0.1%	-0.7	7729
LOC690182	0.8%	0.1%	-0.7	7730
Ncam2	0.8%	0.1%	-0.7	7731
LOC100363423	0.8%	0.1%	-0.7	7732
Emd	4.7%	4.0%	-0.7	7733
LOC102547387	4.7%	4.0%	-0.7	7734
Zfp593	4.7%	4.0%	-0.7	7735
Slc35b2	4.7%	4.0%	-0.7	7736
Ddx42	4.7%	4.0%	-0.7	7737
Gps2	4.3%	3.6%	-0.7	7738
Chd2	4.3%	3.6%	-0.7	7739
Narfl	4.3%	3.6%	-0.7	7740
Cnot7	4.3%	3.6%	-0.7	7741
Snrpg	8.2%	7.5%	-0.7	7742
LOC102551340	3.9%	3.2%	-0.7	7743
Sdhaf4	3.5%	2.8%	-0.7	7744
Prr5	3.5%	2.8%	-0.7	7745
Cog2	3.5%	2.8%	-0.7	7746
Zfp703	11.8%	11.0%	-0.7	7747
Large2	3.1%	2.4%	-0.7	7748
Exosc9	3.1%	2.4%	-0.7	7749
Nfat5	3.1%	2.4%	-0.7	7750
Narf	3.1%	2.4%	-0.7	7751
Snrpa	3.1%	2.4%	-0.7	7752
LOC100359687	7.1%	6.3%	-0.7	7753
Vps72	2.7%	2.0%	-0.7	7754
Ttc32	2.7%	2.0%	-0.7	7755
Zfp787	2.7%	2.0%	-0.7	7756
Slc35a3	2.7%	2.0%	-0.7	7757
Nde1	2.7%	2.0%	-0.7	7758
Nol6	6.7%	5.9%	-0.8	7759
Tasp1	6.7%	5.9%	-0.8	7760
Ccdc149	2.4%	1.6%	-0.8	7761
Atp8b1	2.4%	1.6%	-0.8	7762
Inip	2.4%	1.6%	-0.8	7763
Ankzf1	2.4%	1.6%	-0.8	7764
Otub2	2.4%	1.6%	-0.8	7765
Pygo2	2.4%	1.6%	-0.8	7766
Prune	2.4%	1.6%	-0.8	7767
Nlrx1	2.4%	1.6%	-0.8	7768
Pfkfb2	2.4%	1.6%	-0.8	7769
Rnf111l2	2.4%	1.6%	-0.8	7770
Prim1	2.4%	1.6%	-0.8	7771
Bcl2l12	2.0%	1.2%	-0.8	7772
Ap1g2	2.0%	1.2%	-0.8	7773
Cdk12	2.0%	1.2%	-0.8	7774
Emc9	2.0%	1.2%	-0.8	7775
Shank2	2.0%	1.2%	-0.8	7776
Ppp1r35	2.0%	1.2%	-0.8	7777
Lats1	2.0%	1.2%	-0.8	7778
Ccnyl1	2.0%	1.2%	-0.8	7779
Nfkbie	2.0%	1.2%	-0.8	7780
St3gal5	2.0%	1.2%	-0.8	7781
Hpcal1	2.0%	1.2%	-0.8	7782

Selenoi	2.0%	1.2%	-0.8	7783
Idua	2.0%	1.2%	-0.8	7784
LOC685933	2.0%	1.2%	-0.8	7785
Asap2	2.0%	1.2%	-0.8	7786
NEWGENE_1306267	2.0%	1.2%	-0.8	7787
Ccdc146	2.0%	1.2%	-0.8	7788
Enpp4	2.0%	1.2%	-0.8	7789
Fbxw7	1.6%	0.8%	-0.8	7790
Ints1	1.6%	0.8%	-0.8	7791
Mesdc1	1.6%	0.8%	-0.8	7792
Ift43	1.6%	0.8%	-0.8	7793
Large1	1.6%	0.8%	-0.8	7794
Mms19	1.6%	0.8%	-0.8	7795
Cacna1d	1.6%	0.8%	-0.8	7796
Cyb561	1.6%	0.8%	-0.8	7797
Amigo3	1.6%	0.8%	-0.8	7798
Lnp	1.6%	0.8%	-0.8	7799
Mynn	1.6%	0.8%	-0.8	7800
Znf48	1.6%	0.8%	-0.8	7801
Vamp1	1.6%	0.8%	-0.8	7802
Rdh10	1.6%	0.8%	-0.8	7803
RGD1311892	1.6%	0.8%	-0.8	7804
Vps8	1.6%	0.8%	-0.8	7805
Tbc1d12	1.6%	0.8%	-0.8	7806
Gpsm2	1.6%	0.8%	-0.8	7807
LOC102551027	1.6%	0.8%	-0.8	7808
LOC108351917	1.6%	0.8%	-0.8	7809
Rab32	1.6%	0.8%	-0.8	7810
Asb13	1.6%	0.8%	-0.8	7811
Wwox	1.6%	0.8%	-0.8	7812
Telo2	1.6%	0.8%	-0.8	7813
Alkbh6	1.6%	0.8%	-0.8	7814
Tnfaip8	1.6%	0.8%	-0.8	7815
Pdlim7	1.6%	0.8%	-0.8	7816
Pmf1	1.6%	0.8%	-0.8	7817
Spata5l1	1.6%	0.8%	-0.8	7818
LOC102548374	1.6%	0.8%	-0.8	7819
Tsc22d2	9.8%	9.0%	-0.8	7820
Nup133	1.2%	0.4%	-0.8	7821
Trmt13	1.2%	0.4%	-0.8	7822
Sgtb	1.2%	0.4%	-0.8	7823
Gfod2	1.2%	0.4%	-0.8	7824
Mier2	1.2%	0.4%	-0.8	7825
RGD1562699	1.2%	0.4%	-0.8	7826
Polh	1.2%	0.4%	-0.8	7827
LOC102554727	1.2%	0.4%	-0.8	7828
Fbxl16	1.2%	0.4%	-0.8	7829
Cyp2c24	1.2%	0.4%	-0.8	7830
Fry1	1.2%	0.4%	-0.8	7831
Ticam1	1.2%	0.4%	-0.8	7832
Heatr5a	1.2%	0.4%	-0.8	7833
Chrd	1.2%	0.4%	-0.8	7834
Sun1	1.2%	0.4%	-0.8	7835
Zscan21	1.2%	0.4%	-0.8	7836
Tead3	1.2%	0.4%	-0.8	7837
Mapk1ip1l	1.2%	0.4%	-0.8	7838
LOC102550051	1.2%	0.4%	-0.8	7839
Tmco3	1.2%	0.4%	-0.8	7840
Abcc8	1.2%	0.4%	-0.8	7841
Evi5l	1.2%	0.4%	-0.8	7842
Rgs19	1.2%	0.4%	-0.8	7843
Kctd12	1.2%	0.4%	-0.8	7844
Fbxo45	1.2%	0.4%	-0.8	7845
Phf6	1.2%	0.4%	-0.8	7846
Adamdec1	1.2%	0.4%	-0.8	7847
Dcun1d3	1.2%	0.4%	-0.8	7848

Fbxl8	1.2%	0.4%	-0.8	7849
Cenpj	1.2%	0.4%	-0.8	7850
Wdsub1	1.2%	0.4%	-0.8	7851
Pcsk4	1.2%	0.4%	-0.8	7852
Dtx4	1.2%	0.4%	-0.8	7853
Zfp367	1.2%	0.4%	-0.8	7854
Zfp865	1.2%	0.4%	-0.8	7855
Rasd1	1.2%	0.4%	-0.8	7856
LOC103691124	1.2%	0.4%	-0.8	7857
Fnbp1l	1.2%	0.4%	-0.8	7858
Gcfc2	1.2%	0.4%	-0.8	7859
Camta1	1.2%	0.4%	-0.8	7860
Zbtb44	5.1%	4.3%	-0.8	7861
Ranbp3	5.1%	4.3%	-0.8	7862
Yif1b	5.1%	4.3%	-0.8	7863
Adamts6	0.8%	0.0%	-0.8	7864
Adgrf3	0.8%	0.0%	-0.8	7865
Aloxe3	0.8%	0.0%	-0.8	7866
Ar	0.8%	0.0%	-0.8	7867
Bdnf	0.8%	0.0%	-0.8	7868
Ccsap	0.8%	0.0%	-0.8	7869
Cep97	0.8%	0.0%	-0.8	7870
Col27a1	0.8%	0.0%	-0.8	7871
Col4a5	0.8%	0.0%	-0.8	7872
Ddb2	0.8%	0.0%	-0.8	7873
Dsg1	0.8%	0.0%	-0.8	7874
Elac1	0.8%	0.0%	-0.8	7875
Erc2	0.8%	0.0%	-0.8	7876
Fam222a	0.8%	0.0%	-0.8	7877
Klhl15	0.8%	0.0%	-0.8	7878
LOC100909784	0.8%	0.0%	-0.8	7879
LOC100912365	0.8%	0.0%	-0.8	7880
LOC102550337	0.8%	0.0%	-0.8	7881
LOC102550701	0.8%	0.0%	-0.8	7882
LOC103691033	0.8%	0.0%	-0.8	7883
LOC103692862	0.8%	0.0%	-0.8	7884
LOC108349020	0.8%	0.0%	-0.8	7885
LOC108349244	0.8%	0.0%	-0.8	7886
LOC108352728	0.8%	0.0%	-0.8	7887
LOC688335	0.8%	0.0%	-0.8	7888
LOC690035	0.8%	0.0%	-0.8	7889
Mum1l1	0.8%	0.0%	-0.8	7890
Npnt	0.8%	0.0%	-0.8	7891
Nup43	0.8%	0.0%	-0.8	7892
Pcgf3	0.8%	0.0%	-0.8	7893
Pde12	0.8%	0.0%	-0.8	7894
Plekhm2	0.8%	0.0%	-0.8	7895
Plp1	0.8%	0.0%	-0.8	7896
Pole	0.8%	0.0%	-0.8	7897
Pwwp2b	0.8%	0.0%	-0.8	7898
RGD1306626	0.8%	0.0%	-0.8	7899
Sipa1l2	0.8%	0.0%	-0.8	7900
Slc13a2	0.8%	0.0%	-0.8	7901
Slc22a25	0.8%	0.0%	-0.8	7902
Slc29a4	0.8%	0.0%	-0.8	7903
Slc36a1	0.8%	0.0%	-0.8	7904
Spdl1	0.8%	0.0%	-0.8	7905
Spin2a	0.8%	0.0%	-0.8	7906
Spin4	0.8%	0.0%	-0.8	7907
Ube3d	0.8%	0.0%	-0.8	7908
Zbtb40	0.8%	0.0%	-0.8	7909
Zc3hav1l	0.8%	0.0%	-0.8	7910
Zfp617	0.8%	0.0%	-0.8	7911
Zfp641	0.8%	0.0%	-0.8	7912
Zfp763	0.8%	0.0%	-0.8	7913
Zscan20	0.8%	0.0%	-0.8	7914

Tsen34l1	9.0%	8.2%	-0.8	7915
Arid1b	4.7%	3.9%	-0.8	7916
Ubtf	4.7%	3.9%	-0.8	7917
Clk4	4.7%	3.9%	-0.8	7918
Pmvk	12.9%	12.2%	-0.8	7919
LOC102548397	4.3%	3.5%	-0.8	7920
Kng2	100.0%	99.2%	-0.8	7921
Apip	3.9%	3.1%	-0.8	7922
Parp4	3.9%	3.1%	-0.8	7923
Esyt2	3.9%	3.1%	-0.8	7924
Mturn	3.9%	3.1%	-0.8	7925
Setx	3.5%	2.7%	-0.8	7926
LOC100363321	3.5%	2.7%	-0.8	7927
LOC103689996	3.5%	2.7%	-0.8	7928
Epha1	3.5%	2.7%	-0.8	7929
Brf1	3.5%	2.7%	-0.8	7930
Tmem39b	3.5%	2.7%	-0.8	7931
Polr2i	3.5%	2.7%	-0.8	7932
Creb1	3.5%	2.7%	-0.8	7933
Ido2	3.1%	2.3%	-0.8	7934
Cnot10	3.1%	2.3%	-0.8	7935
Tgm1	3.1%	2.3%	-0.8	7936
Ppt2	3.1%	2.3%	-0.8	7937
Eral1	3.1%	2.3%	-0.8	7938
Gapvd1	3.1%	2.3%	-0.8	7939
Adrm1	11.4%	10.6%	-0.8	7940
LOC100362751	32.2%	31.3%	-0.8	7941
Cdk11b	7.1%	6.2%	-0.8	7942
Smarcc2	2.7%	1.9%	-0.8	7943
Myo7b	2.7%	1.9%	-0.8	7944
Washc1	2.7%	1.9%	-0.8	7945
LOC102547136	2.7%	1.9%	-0.8	7946
Haus7	2.7%	1.9%	-0.8	7947
Spcs3	2.7%	1.9%	-0.8	7948
Pigf	2.7%	1.9%	-0.8	7949
Kdf1	2.7%	1.9%	-0.8	7950
RT1-DMa	2.7%	1.9%	-0.8	7951
Tmem110	2.7%	1.9%	-0.8	7952
Med19	2.4%	1.5%	-0.8	7953
Sf3a2	2.4%	1.5%	-0.8	7954
LOC108348065	2.4%	1.5%	-0.8	7955
Kdm4a	2.4%	1.5%	-0.8	7956
Usp19	2.4%	1.5%	-0.8	7957
Zfp874b	2.4%	1.5%	-0.8	7958
Ikbkap	2.0%	1.1%	-0.8	7959
Spen	2.0%	1.1%	-0.8	7960
Lyrm7	2.0%	1.1%	-0.8	7961
LOC108348210	2.0%	1.1%	-0.8	7962
Ccdc137	2.0%	1.1%	-0.8	7963
Gpatch2	2.0%	1.1%	-0.8	7964
LOC108351262	2.0%	1.1%	-0.8	7965
Vsig10l	2.0%	1.1%	-0.8	7966
Fabp7	2.0%	1.1%	-0.8	7967
Fmo4	2.0%	1.1%	-0.8	7968
Cdc42bpg	2.0%	1.1%	-0.8	7969
Slc39a13	2.0%	1.1%	-0.8	7970
Zswim6	2.0%	1.1%	-0.8	7971
Tsen34	10.2%	9.4%	-0.8	7972
Polr3f	1.6%	0.7%	-0.8	7973
Kif1b	1.6%	0.7%	-0.8	7974
Rab2b	1.6%	0.7%	-0.8	7975
LOC102550999	1.6%	0.7%	-0.8	7976
Pinx1	1.6%	0.7%	-0.8	7977
Hectd2	1.6%	0.7%	-0.8	7978
Dcps	1.6%	0.7%	-0.8	7979
Nup155	1.6%	0.7%	-0.8	7980

Spata24	1.6%	0.7%	-0.8	7981
LOC102554057	1.6%	0.7%	-0.8	7982
Rnf151	1.6%	0.7%	-0.8	7983
Irf2bp1	1.6%	0.7%	-0.8	7984
Casp8ap2	1.6%	0.7%	-0.8	7985
Ascc2	1.6%	0.7%	-0.8	7986
Rab11fip3	1.6%	0.7%	-0.8	7987
Zmym4	1.6%	0.7%	-0.8	7988
Ttyh3	1.6%	0.7%	-0.8	7989
Dvl3	1.6%	0.7%	-0.8	7990
Edc4	1.6%	0.7%	-0.8	7991
Arhgef12	1.6%	0.7%	-0.8	7992
Tgif2	1.6%	0.7%	-0.8	7993
Pcgf2	1.6%	0.7%	-0.8	7994
Pdap1	22.4%	21.5%	-0.9	7995
Lsm7	5.5%	4.6%	-0.9	7996
Scamp1	5.5%	4.6%	-0.9	7997
Abcd4	1.2%	0.3%	-0.9	7998
Impdh1	1.2%	0.3%	-0.9	7999
Hist1h1d	1.2%	0.3%	-0.9	8000
Mblac2	1.2%	0.3%	-0.9	8001
LOC102547287	1.2%	0.3%	-0.9	8002
Tfcp2l1	1.2%	0.3%	-0.9	8003
Zfp128	1.2%	0.3%	-0.9	8004
Efnb1	1.2%	0.3%	-0.9	8005
LOC108352854	1.2%	0.3%	-0.9	8006
Bag4	1.2%	0.3%	-0.9	8007
Ankle2	1.2%	0.3%	-0.9	8008
Peli2	1.2%	0.3%	-0.9	8009
RGD1307554	1.2%	0.3%	-0.9	8010
Ms4a18	1.2%	0.3%	-0.9	8011
Zfp472	1.2%	0.3%	-0.9	8012
Zdhhc14	1.2%	0.3%	-0.9	8013
Fbp2	1.2%	0.3%	-0.9	8014
Ptar1	1.2%	0.3%	-0.9	8015
Dph7	1.2%	0.3%	-0.9	8016
Gtpbp1	1.2%	0.3%	-0.9	8017
LOC103690457	1.2%	0.3%	-0.9	8018
B3glct	1.2%	0.3%	-0.9	8019
Nrde2	1.2%	0.3%	-0.9	8020
LOC102547682	1.2%	0.3%	-0.9	8021
Tuba3a	1.2%	0.3%	-0.9	8022
Tp53bp1	1.2%	0.3%	-0.9	8023
LOC108348190	1.2%	0.3%	-0.9	8024
Olr59	1.2%	0.3%	-0.9	8025
Zfp952	1.2%	0.3%	-0.9	8026
Iqsec1	5.1%	4.2%	-0.9	8027
Fmr1	4.7%	3.8%	-0.9	8028
Fam49b	4.7%	3.8%	-0.9	8029
Slmap	4.3%	3.4%	-0.9	8030
MGC95208	4.3%	3.4%	-0.9	8031
Ddx28	4.3%	3.4%	-0.9	8032
Hpx	100.0%	99.1%	-0.9	8033
Rbm39	16.1%	15.2%	-0.9	8034
Ski	3.5%	2.6%	-0.9	8035
Snrnp27	3.5%	2.6%	-0.9	8036
Zfp958	3.5%	2.6%	-0.9	8037
LOC102557254	3.5%	2.6%	-0.9	8038
Gtf2h4	3.1%	2.2%	-0.9	8039
Eif4enif1	3.1%	2.2%	-0.9	8040
Plaur	3.1%	2.2%	-0.9	8041
LOC100910990	3.1%	2.2%	-0.9	8042
Birc3	7.1%	6.2%	-0.9	8043
Tmem161a	2.7%	1.8%	-0.9	8044
Reps2	2.7%	1.8%	-0.9	8045
Rapgef1	2.7%	1.8%	-0.9	8046

Birc6	2.7%	1.8%	-0.9	8047
Fbxo28	2.7%	1.8%	-0.9	8048
Mcm3ap	2.7%	1.8%	-0.9	8049
Ankfy1	2.7%	1.8%	-0.9	8050
Clybl	2.7%	1.8%	-0.9	8051
Dbndd2	2.7%	1.8%	-0.9	8052
Ldlrad4	2.7%	1.8%	-0.9	8053
Igip	2.4%	1.4%	-0.9	8054
Grb7	2.4%	1.4%	-0.9	8055
Hipk2	2.4%	1.4%	-0.9	8056
Thap6	2.4%	1.4%	-0.9	8057
Rad1	2.4%	1.4%	-0.9	8058
Stmn1	6.3%	5.4%	-0.9	8059
Fv1	2.0%	1.0%	-0.9	8060
Ficd	2.0%	1.0%	-0.9	8061
Atrip	2.0%	1.0%	-0.9	8062
Snx12	2.0%	1.0%	-0.9	8063
Tmem260	2.0%	1.0%	-0.9	8064
Gpank1	2.0%	1.0%	-0.9	8065
C5ar2	2.0%	1.0%	-0.9	8066
Svbp	2.0%	1.0%	-0.9	8067
Zdhhc1	2.0%	1.0%	-0.9	8068
Smyd3	2.0%	1.0%	-0.9	8069
Dgcr8	2.0%	1.0%	-0.9	8070
Mark4	2.0%	1.0%	-0.9	8071
Arhgap21	2.0%	1.0%	-0.9	8072
Vps39	2.0%	1.0%	-0.9	8073
RGD1307235	2.0%	1.0%	-0.9	8074
Avl9	2.0%	1.0%	-0.9	8075
LOC102557324	2.0%	1.0%	-0.9	8076
Plekhg5	2.0%	1.0%	-0.9	8077
LOC103694879	2.0%	1.0%	-0.9	8078
Prkdc	2.0%	1.0%	-0.9	8079
Enoph1	2.0%	1.0%	-0.9	8080
Zcchc14	2.0%	1.0%	-0.9	8081
Sh3bp5l	2.0%	1.0%	-0.9	8082
Mov10	2.0%	1.0%	-0.9	8083
Wiz	1.6%	0.6%	-0.9	8084
Taf4	1.6%	0.6%	-0.9	8085
Zfp830	1.6%	0.6%	-0.9	8086
RGD1309621	1.6%	0.6%	-0.9	8087
Phf21a	1.6%	0.6%	-0.9	8088
Rabif	1.6%	0.6%	-0.9	8089
Tgfa	1.6%	0.6%	-0.9	8090
Srd5a3	1.6%	0.6%	-0.9	8091
LOC102548179	1.6%	0.6%	-0.9	8092
Sirt4	1.6%	0.6%	-0.9	8093
Ints11	1.6%	0.6%	-0.9	8094
Fbxl15	1.6%	0.6%	-0.9	8095
LOC103694865	1.6%	0.6%	-0.9	8096
Mapk8ip3	1.6%	0.6%	-0.9	8097
RGD1565059	1.6%	0.6%	-0.9	8098
RGD1309036	1.6%	0.6%	-0.9	8099
Smarcal1	1.6%	0.6%	-0.9	8100
Pias1	1.6%	0.6%	-0.9	8101
Atat1	1.6%	0.6%	-0.9	8102
Med17	1.6%	0.6%	-0.9	8103
Vwa9	1.6%	0.6%	-0.9	8104
LOC100911225	1.6%	0.6%	-0.9	8105
Arhgef18	1.6%	0.6%	-0.9	8106
Paxip1	1.6%	0.6%	-0.9	8107
Dopey2	1.6%	0.6%	-0.9	8108
Zfp384	1.6%	0.6%	-0.9	8109
Dip2c	1.6%	0.6%	-0.9	8110
Wwc2	1.6%	0.6%	-0.9	8111
Rasa1	1.6%	0.6%	-0.9	8112

Myadm	1.6%	0.6%	-0.9	8113
Odf2l	1.6%	0.6%	-0.9	8114
Zfp629	1.2%	0.2%	-0.9	8115
Fbxo5	1.2%	0.2%	-0.9	8116
Myh14	1.2%	0.2%	-0.9	8117
Usp27x	1.2%	0.2%	-0.9	8118
Heatr5b	1.2%	0.2%	-0.9	8119
Ncoa5	1.2%	0.2%	-0.9	8120
Rad52	1.2%	0.2%	-0.9	8121
Tbc1d7	1.2%	0.2%	-0.9	8122
Alkbh5	1.2%	0.2%	-0.9	8123
Wdr5b	1.2%	0.2%	-0.9	8124
Lrsam1	1.2%	0.2%	-0.9	8125
Gtpbp3	1.2%	0.2%	-0.9	8126
Ube2o	1.2%	0.2%	-0.9	8127
Pold1	1.2%	0.2%	-0.9	8128
Rita1	1.2%	0.2%	-0.9	8129
Fancc	1.2%	0.2%	-0.9	8130
Zbtb25	1.2%	0.2%	-0.9	8131
LOC102549668	1.2%	0.2%	-0.9	8132
Nt5dc2	1.2%	0.2%	-0.9	8133
LOC103690085	1.2%	0.2%	-0.9	8134
Akr1c19	1.2%	0.2%	-0.9	8135
LOC103693340	1.2%	0.2%	-0.9	8136
Ssh1	1.2%	0.2%	-0.9	8137
LOC100910668	1.2%	0.2%	-0.9	8138
LOC103693699	1.2%	0.2%	-0.9	8139
LOC103693419	1.2%	0.2%	-0.9	8140
Spice1	1.2%	0.2%	-0.9	8141
Lclat1	1.2%	0.2%	-0.9	8142
Gpr137	1.2%	0.2%	-0.9	8143
Ccdc138	1.2%	0.2%	-0.9	8144
RGD1562080	1.2%	0.2%	-0.9	8145
Il23a	1.2%	0.2%	-0.9	8146
Ifi44	4.7%	3.8%	-0.9	8147
Cnot4	4.7%	3.8%	-0.9	8148
Dda1	4.3%	3.4%	-1.0	8149
Elmsan1	4.3%	3.4%	-1.0	8150
Ccdc97	4.3%	3.4%	-1.0	8151
Arl1	4.3%	3.4%	-1.0	8152
LOC100361913	4.3%	3.4%	-1.0	8153
Ifi27	100.0%	99.0%	-1.0	8154
LOC100365921	3.9%	3.0%	-1.0	8155
Josd2	3.9%	3.0%	-1.0	8156
Raph1	3.9%	3.0%	-1.0	8157
Rfc1	7.8%	6.9%	-1.0	8158
Otub1	7.8%	6.9%	-1.0	8159
Nras	7.8%	6.9%	-1.0	8160
Smc4	3.5%	2.6%	-1.0	8161
Fubp1	3.5%	2.6%	-1.0	8162
Sirt7	3.5%	2.6%	-1.0	8163
Chid1	3.5%	2.6%	-1.0	8164
Ipo9	3.1%	2.2%	-1.0	8165
Exoc2	3.1%	2.2%	-1.0	8166
LOC688583	3.1%	2.2%	-1.0	8167
Sft2d2	3.1%	2.2%	-1.0	8168
Lgals5	2.7%	1.8%	-1.0	8169
Zfyve19	2.7%	1.8%	-1.0	8170
Rbm15	2.7%	1.8%	-1.0	8171
Cdk2	2.7%	1.8%	-1.0	8172
Acyp1	2.7%	1.8%	-1.0	8173
Cep89	2.7%	1.8%	-1.0	8174
Plk3	2.7%	1.8%	-1.0	8175
Ppara	2.7%	1.8%	-1.0	8176
Rrm1	2.7%	1.8%	-1.0	8177
LOC108351513	2.7%	1.8%	-1.0	8178

Srfbp1	2.7%	1.8%	-1.0	8179
RGD1304728	2.7%	1.8%	-1.0	8180
Slc4a1ap	2.4%	1.4%	-1.0	8181
LOC102548233	2.4%	1.4%	-1.0	8182
Ippk	2.4%	1.4%	-1.0	8183
Kat6a	2.4%	1.4%	-1.0	8184
Cav2	2.4%	1.4%	-1.0	8185
Lyrm9	2.4%	1.4%	-1.0	8186
LOC100909782	2.4%	1.4%	-1.0	8187
Pwp2	2.4%	1.4%	-1.0	8188
Rlf	2.4%	1.4%	-1.0	8189
Ybey	2.4%	1.4%	-1.0	8190
Hmgn5b	10.6%	9.6%	-1.0	8191
Pbld2	10.6%	9.6%	-1.0	8192
Arid4b	6.3%	5.3%	-1.0	8193
Mfap3l	2.0%	1.0%	-1.0	8194
Csnk1e	2.0%	1.0%	-1.0	8195
Atp6ap1l	2.0%	1.0%	-1.0	8196
Eid2	2.0%	1.0%	-1.0	8197
Pigg	2.0%	1.0%	-1.0	8198
Rnf123	2.0%	1.0%	-1.0	8199
Pgap3	2.0%	1.0%	-1.0	8200
Bri3bp	2.0%	1.0%	-1.0	8201
Trio	2.0%	1.0%	-1.0	8202
Gata6	2.0%	1.0%	-1.0	8203
LOC103694558	2.0%	1.0%	-1.0	8204
LOC108348055	2.0%	1.0%	-1.0	8205
RGD1309651	2.0%	1.0%	-1.0	8206
Ltbp4	2.0%	1.0%	-1.0	8207
Ptcd1	2.0%	1.0%	-1.0	8208
RGD1566265	2.0%	1.0%	-1.0	8209
LOC288978	2.0%	1.0%	-1.0	8210
Tigd2	2.0%	1.0%	-1.0	8211
LOC102553702	2.0%	1.0%	-1.0	8212
Dcaf4	2.0%	1.0%	-1.0	8213
LOC679894	1.6%	0.6%	-1.0	8214
Wfdc3	1.6%	0.6%	-1.0	8215
Pstk	1.6%	0.6%	-1.0	8216
Gin1	1.6%	0.6%	-1.0	8217
Fbxo42	1.6%	0.6%	-1.0	8218
Atg16l2	1.6%	0.6%	-1.0	8219
Donson	1.6%	0.6%	-1.0	8220
Dock4	1.6%	0.6%	-1.0	8221
Papss1	1.6%	0.6%	-1.0	8222
Hspb6	1.6%	0.6%	-1.0	8223
Zfp212	1.6%	0.6%	-1.0	8224
Phc1	1.6%	0.6%	-1.0	8225
Abhd18	1.6%	0.6%	-1.0	8226
Peak1	1.6%	0.6%	-1.0	8227
LOC102550246	1.6%	0.6%	-1.0	8228
LOC108349934	1.6%	0.6%	-1.0	8229
LOC100365289	1.6%	0.6%	-1.0	8230
Zfp236	1.6%	0.6%	-1.0	8231
Epb41l2	1.6%	0.6%	-1.0	8232
LOC103695087	1.6%	0.6%	-1.0	8233
Usp37	1.6%	0.6%	-1.0	8234
Tmtc4	1.6%	0.6%	-1.0	8235
Naf1	1.6%	0.6%	-1.0	8236
Kdm5d	1.6%	0.6%	-1.0	8237
Atg13	1.6%	0.6%	-1.0	8238
Tbc1d9	1.6%	0.6%	-1.0	8239
Cir1	5.5%	4.5%	-1.0	8240
Supt6h	5.5%	4.5%	-1.0	8241
Tmed3	5.5%	4.5%	-1.0	8242
Prelid2	5.5%	4.5%	-1.0	8243
Ppip5k1	1.2%	0.2%	-1.0	8244

Tmem189	1.2%	0.2%	-1.0	8245
Zfp799	1.2%	0.2%	-1.0	8246
Sel1l3	1.2%	0.2%	-1.0	8247
Dancr	1.2%	0.2%	-1.0	8248
Clspn	1.2%	0.2%	-1.0	8249
LOC108348225	1.2%	0.2%	-1.0	8250
Map3k15	1.2%	0.2%	-1.0	8251
Sorl1	1.2%	0.2%	-1.0	8252
Zfp397	1.2%	0.2%	-1.0	8253
Ttf1	1.2%	0.2%	-1.0	8254
Icam5	1.2%	0.2%	-1.0	8255
LOC108352082	1.2%	0.2%	-1.0	8256
Tspo2	1.2%	0.2%	-1.0	8257
LOC361646	1.2%	0.2%	-1.0	8258
Palm2	1.2%	0.2%	-1.0	8259
Trhde	1.2%	0.2%	-1.0	8260
Bicdl1	1.2%	0.2%	-1.0	8261
Trim45	1.2%	0.2%	-1.0	8262
Amigo1	1.2%	0.2%	-1.0	8263
Arhgef37	1.2%	0.2%	-1.0	8264
Ube2d4	1.2%	0.2%	-1.0	8265
Gnat2	1.2%	0.2%	-1.0	8266
LOC498453	5.1%	4.1%	-1.0	8267
Swt1	4.7%	3.7%	-1.0	8268
Cog7	4.7%	3.7%	-1.0	8269
Nae1	4.7%	3.7%	-1.0	8270
Taf12	4.7%	3.7%	-1.0	8271
Pxmp4	8.2%	7.2%	-1.0	8272
Nup35	3.9%	2.9%	-1.0	8273
Prrg2	3.9%	2.9%	-1.0	8274
Pten	3.9%	2.9%	-1.0	8275
Rpl10l	3.9%	2.9%	-1.0	8276
Pank2	3.9%	2.9%	-1.0	8277
Slc27a5	99.6%	98.6%	-1.0	8278
Sccpdh	7.8%	6.8%	-1.0	8279
Tmem141	3.5%	2.5%	-1.1	8280
Asxl2	3.5%	2.5%	-1.1	8281
Unc5cl	3.1%	2.1%	-1.1	8282
Ythdc2	3.1%	2.1%	-1.1	8283
LOC691921	3.1%	2.1%	-1.1	8284
Gtf2h2	3.1%	2.1%	-1.1	8285
Prss23	2.7%	1.7%	-1.1	8286
Ptrh1	2.7%	1.7%	-1.1	8287
Ube2d1	2.7%	1.7%	-1.1	8288
Arl15	2.7%	1.7%	-1.1	8289
Nme6	2.7%	1.7%	-1.1	8290
Bcl6	2.7%	1.7%	-1.1	8291
Rhoh	2.7%	1.7%	-1.1	8292
Oxsm	2.7%	1.7%	-1.1	8293
Sympk	2.7%	1.7%	-1.1	8294
Vcpip1	2.7%	1.7%	-1.1	8295
Flywch1	2.7%	1.7%	-1.1	8296
Anxa5	6.7%	5.6%	-1.1	8297
Ppl	2.4%	1.3%	-1.1	8298
Bmp7	2.4%	1.3%	-1.1	8299
Pter	2.4%	1.3%	-1.1	8300
Dlgap4	2.4%	1.3%	-1.1	8301
B3galnt2	2.4%	1.3%	-1.1	8302
Atp11a	2.4%	1.3%	-1.1	8303
Snx7	2.4%	1.3%	-1.1	8304
Lrrc57	2.4%	1.3%	-1.1	8305
Clasp1	2.0%	0.9%	-1.1	8306
Exosc3	2.0%	0.9%	-1.1	8307
Zcchc10	2.0%	0.9%	-1.1	8308
LOC100909810	2.0%	0.9%	-1.1	8309
Ckap5	2.0%	0.9%	-1.1	8310

Ctc1	2.0%	0.9%	-1.1	8311
Scaf4	2.0%	0.9%	-1.1	8312
Gemin4	2.0%	0.9%	-1.1	8313
Brpf3	2.0%	0.9%	-1.1	8314
Bbof1	2.0%	0.9%	-1.1	8315
Shkbp1	2.0%	0.9%	-1.1	8316
Tnk2	2.0%	0.9%	-1.1	8317
LOC108348248	2.0%	0.9%	-1.1	8318
Btbd10	2.0%	0.9%	-1.1	8319
Wdpcp	2.0%	0.9%	-1.1	8320
RGD1306746	2.0%	0.9%	-1.1	8321
Homer3	2.0%	0.9%	-1.1	8322
LOC103693776	2.0%	0.9%	-1.1	8323
Rft1	2.0%	0.9%	-1.1	8324
Zc3h3	2.0%	0.9%	-1.1	8325
LOC108348175	2.0%	0.9%	-1.1	8326
Tex12	2.0%	0.9%	-1.1	8327
Toe1	2.0%	0.9%	-1.1	8328
Xrcc4	2.0%	0.9%	-1.1	8329
Fn3k	2.0%	0.9%	-1.1	8330
Aar2	2.0%	0.9%	-1.1	8331
Shc1	10.2%	9.1%	-1.1	8332
Myd88	5.9%	4.8%	-1.1	8333
Hspg2	1.6%	0.5%	-1.1	8334
Tmem132a	1.6%	0.5%	-1.1	8335
Opa3	1.6%	0.5%	-1.1	8336
Neurl4	1.6%	0.5%	-1.1	8337
Aars2	1.6%	0.5%	-1.1	8338
Slc52a2	1.6%	0.5%	-1.1	8339
Nmrk1	1.6%	0.5%	-1.1	8340
Mybl1	1.6%	0.5%	-1.1	8341
Atm	1.6%	0.5%	-1.1	8342
Zrsr1	1.6%	0.5%	-1.1	8343
Myo5b	1.6%	0.5%	-1.1	8344
LOC108348304	1.6%	0.5%	-1.1	8345
Ptpn13	1.6%	0.5%	-1.1	8346
Gtf3c5	1.6%	0.5%	-1.1	8347
Abl1	1.6%	0.5%	-1.1	8348
Abhd14a	1.6%	0.5%	-1.1	8349
Cnst	1.6%	0.5%	-1.1	8350
LOC103690149	1.6%	0.5%	-1.1	8351
Cep104	1.6%	0.5%	-1.1	8352
Stoml1	1.6%	0.5%	-1.1	8353
Tubgcp4	1.6%	0.5%	-1.1	8354
Wdr60	1.6%	0.5%	-1.1	8355
Hcfc2	1.6%	0.5%	-1.1	8356
LOC100911506	1.6%	0.5%	-1.1	8357
Ankrd54	1.6%	0.5%	-1.1	8358
Zbtb38	1.6%	0.5%	-1.1	8359
Slc6a8	1.6%	0.5%	-1.1	8360
LOC103690346	1.6%	0.5%	-1.1	8361
Prox1	9.8%	8.7%	-1.1	8362
Agl	5.5%	4.4%	-1.1	8363
Kpna5	1.2%	0.1%	-1.1	8364
LOC102547848	1.2%	0.1%	-1.1	8365
Socs4	1.2%	0.1%	-1.1	8366
Micu3	1.2%	0.1%	-1.1	8367
LOC103692812	1.2%	0.1%	-1.1	8368
LOC102555341	1.2%	0.1%	-1.1	8369
Hrk	1.2%	0.1%	-1.1	8370
Acvr1c	1.2%	0.1%	-1.1	8371
Snapc4	1.2%	0.1%	-1.1	8372
RGD1562272	1.2%	0.1%	-1.1	8373
LOC690422	1.2%	0.1%	-1.1	8374
Zfp458	1.2%	0.1%	-1.1	8375
Tmem151a	1.2%	0.1%	-1.1	8376

Adamts12	1.2%	0.1%	-1.1	8377
LOC102553866	1.2%	0.1%	-1.1	8378
Gpm6a	1.2%	0.1%	-1.1	8379
Mrm1	1.2%	0.1%	-1.1	8380
Tyro3	1.2%	0.1%	-1.1	8381
LOC100912294	1.2%	0.1%	-1.1	8382
LOC100909688	1.2%	0.1%	-1.1	8383
Tmem265	1.2%	0.1%	-1.1	8384
Tuba3b	1.2%	0.1%	-1.1	8385
Fam13a	5.1%	4.0%	-1.1	8386
Tatdn1	9.0%	7.9%	-1.1	8387
Dao	4.7%	3.6%	-1.1	8388
Hnrnpr	4.7%	3.6%	-1.1	8389
Mylk	4.7%	3.6%	-1.1	8390
Mob2	4.3%	3.2%	-1.1	8391
Ets1	4.3%	3.2%	-1.1	8392
Mysm1	4.3%	3.2%	-1.1	8393
LOC100360368	20.8%	19.7%	-1.1	8394
Exoc1	3.9%	2.8%	-1.1	8395
Plcl2	3.9%	2.8%	-1.1	8396
Mtmr4	3.9%	2.8%	-1.1	8397
Fktn	3.5%	2.4%	-1.1	8398
Rabep2	3.5%	2.4%	-1.1	8399
Dyrk2	3.5%	2.4%	-1.1	8400
Snrnp35	3.5%	2.4%	-1.1	8401
LOC691143	3.1%	2.0%	-1.1	8402
RGD1305178	3.1%	2.0%	-1.1	8403
Ubn2	3.1%	2.0%	-1.1	8404
Taf6	3.1%	2.0%	-1.1	8405
Gpr146	3.1%	2.0%	-1.1	8406
Tmem35	3.1%	2.0%	-1.1	8407
Rassf7	3.1%	2.0%	-1.1	8408
Hnrnpd	11.4%	10.2%	-1.1	8409
Csad	32.2%	31.0%	-1.1	8410
Zbtb12	7.1%	5.9%	-1.1	8411
Irgm	7.1%	5.9%	-1.1	8412
Rictor	2.7%	1.6%	-1.1	8413
Gcn1l1	2.7%	1.6%	-1.1	8414
LOC102555797	2.7%	1.6%	-1.1	8415
Fiz1	2.7%	1.6%	-1.1	8416
Sash1	2.7%	1.6%	-1.1	8417
Cdk17	2.7%	1.6%	-1.1	8418
Ankib1	2.7%	1.6%	-1.1	8419
Ylpm1	2.7%	1.6%	-1.1	8420
Zfyve9	2.7%	1.6%	-1.1	8421
Lars2	2.7%	1.6%	-1.1	8422
Gramd4	2.7%	1.6%	-1.1	8423
Pacsin3	6.7%	5.5%	-1.2	8424
Tstd3	2.4%	1.2%	-1.2	8425
Farp1	2.4%	1.2%	-1.2	8426
Tyms	2.4%	1.2%	-1.2	8427
Ints10	2.4%	1.2%	-1.2	8428
Hps6	2.4%	1.2%	-1.2	8429
Rnf31	2.4%	1.2%	-1.2	8430
Ing1	2.4%	1.2%	-1.2	8431
Phlda2	2.4%	1.2%	-1.2	8432
Abl2	2.4%	1.2%	-1.2	8433
LOC100911661	2.4%	1.2%	-1.2	8434
Zc3h18	2.4%	1.2%	-1.2	8435
Rabl6	2.4%	1.2%	-1.2	8436
LOC100910807	2.4%	1.2%	-1.2	8437
Zfp444	2.4%	1.2%	-1.2	8438
Nod1	2.4%	1.2%	-1.2	8439
Taf8	2.0%	0.8%	-1.2	8440
Ddx55	2.0%	0.8%	-1.2	8441
Sap130	2.0%	0.8%	-1.2	8442

Fam65a	2.0%	0.8%	-1.2	8443
Rab40b	2.0%	0.8%	-1.2	8444
Ubr2	2.0%	0.8%	-1.2	8445
Magi3	2.0%	0.8%	-1.2	8446
Ercc2	2.0%	0.8%	-1.2	8447
Prtfdc1	2.0%	0.8%	-1.2	8448
Nxn	2.0%	0.8%	-1.2	8449
Exoc5	2.0%	0.8%	-1.2	8450
Dennd2d	2.0%	0.8%	-1.2	8451
LOC103690621	2.0%	0.8%	-1.2	8452
LOC102553601	2.0%	0.8%	-1.2	8453
Slc10a3	2.0%	0.8%	-1.2	8454
Slc23a2	2.0%	0.8%	-1.2	8455
Hmces	2.0%	0.8%	-1.2	8456
Atp7b	1.6%	0.4%	-1.2	8457
Gpatch4	1.6%	0.4%	-1.2	8458
Prrg1	1.6%	0.4%	-1.2	8459
Clasrp	1.6%	0.4%	-1.2	8460
RGD1308106	1.6%	0.4%	-1.2	8461
Usp30	1.6%	0.4%	-1.2	8462
Ccl27	1.6%	0.4%	-1.2	8463
Hacd1	1.6%	0.4%	-1.2	8464
Sepn1	1.6%	0.4%	-1.2	8465
MGC116121	1.6%	0.4%	-1.2	8466
Mre11a	1.6%	0.4%	-1.2	8467
Eri2	1.6%	0.4%	-1.2	8468
Thap1	1.6%	0.4%	-1.2	8469
Cdc20	1.6%	0.4%	-1.2	8470
LOC103691602	1.6%	0.4%	-1.2	8471
Plin5	1.6%	0.4%	-1.2	8472
Mbip	1.6%	0.4%	-1.2	8473
Ash1l	5.5%	4.3%	-1.2	8474
Ptprj	5.5%	4.3%	-1.2	8475
Ifi44l	5.5%	4.3%	-1.2	8476
Cyp26a1	5.5%	4.3%	-1.2	8477
Aldh1b1	5.5%	4.3%	-1.2	8478
Atad2b	1.2%	0.0%	-1.2	8479
Bhlha15	1.2%	0.0%	-1.2	8480
Ccdc189	1.2%	0.0%	-1.2	8481
Ccp110	1.2%	0.0%	-1.2	8482
Dpp6	1.2%	0.0%	-1.2	8483
Fam118a	1.2%	0.0%	-1.2	8484
Grem2	1.2%	0.0%	-1.2	8485
Grhl1	1.2%	0.0%	-1.2	8486
Hsd3b1	1.2%	0.0%	-1.2	8487
Kctd7	1.2%	0.0%	-1.2	8488
LOC102557398	1.2%	0.0%	-1.2	8489
LOC103690187	1.2%	0.0%	-1.2	8490
LOC103691238	1.2%	0.0%	-1.2	8491
LOC103692228	1.2%	0.0%	-1.2	8492
Mme	1.2%	0.0%	-1.2	8493
Noxo1	1.2%	0.0%	-1.2	8494
Plxna1	1.2%	0.0%	-1.2	8495
RGD1562963	1.2%	0.0%	-1.2	8496
Tarbp1	1.2%	0.0%	-1.2	8497
Usp43	1.2%	0.0%	-1.2	8498
Zfp37	1.2%	0.0%	-1.2	8499
Zfp563	1.2%	0.0%	-1.2	8500
Dcun1d1	4.7%	3.5%	-1.2	8501
Mxi1	4.7%	3.5%	-1.2	8502
Arhgef1	4.7%	3.5%	-1.2	8503
LOC499179	4.3%	3.1%	-1.2	8504
Oas1a	4.3%	3.1%	-1.2	8505
Tmem199	4.3%	3.1%	-1.2	8506
Letm2	4.3%	3.1%	-1.2	8507
Apoh	100.0%	98.8%	-1.2	8508

Luc7l3	8.2%	7.0%	-1.2	8509
Phyhd1	95.7%	94.5%	-1.2	8510
Fads6	3.9%	2.7%	-1.2	8511
Sdr9c7	3.9%	2.7%	-1.2	8512
R3hdm4	7.8%	6.6%	-1.2	8513
Prpf38a	3.5%	2.3%	-1.2	8514
Map4k4	3.5%	2.3%	-1.2	8515
LOC103689977	3.5%	2.3%	-1.2	8516
Rbm10	3.5%	2.3%	-1.2	8517
Tceanc2	3.5%	2.3%	-1.2	8518
Casd1	3.1%	1.9%	-1.2	8519
Cmss1	3.1%	1.9%	-1.2	8520
Ppil1	3.1%	1.9%	-1.2	8521
Mnt	3.1%	1.9%	-1.2	8522
Golgb1	3.1%	1.9%	-1.2	8523
Dhrs11	3.1%	1.9%	-1.2	8524
Clmn	3.1%	1.9%	-1.2	8525
RGD1565685	3.1%	1.9%	-1.2	8526
LOC100360940	2.7%	1.5%	-1.2	8527
LOC691918	2.7%	1.5%	-1.2	8528
Alpl	2.7%	1.5%	-1.2	8529
Tmem106c	2.7%	1.5%	-1.2	8530
LOC100911946	2.7%	1.5%	-1.2	8531
Ifnar1	11.0%	9.8%	-1.2	8532
Rnls	2.4%	1.1%	-1.2	8533
Ccnt1	2.4%	1.1%	-1.2	8534
Ino80d	2.4%	1.1%	-1.2	8535
Stxbp4	2.4%	1.1%	-1.2	8536
Xrn1	2.4%	1.1%	-1.2	8537
Tomm40l	2.4%	1.1%	-1.2	8538
Usb1	2.4%	1.1%	-1.2	8539
Mios	2.4%	1.1%	-1.2	8540
Gpat3	2.4%	1.1%	-1.2	8541
Stk10	2.4%	1.1%	-1.2	8542
LOC100359816	2.4%	1.1%	-1.2	8543
Clcf1	2.4%	1.1%	-1.2	8544
H1fx	2.4%	1.1%	-1.2	8545
Trip4	2.4%	1.1%	-1.2	8546
Zyg11b	2.4%	1.1%	-1.2	8547
Ralyl	10.6%	9.4%	-1.2	8548
Insr	2.0%	0.7%	-1.2	8549
Mtf2	2.0%	0.7%	-1.2	8550
Ezh2	2.0%	0.7%	-1.2	8551
Zxdc	2.0%	0.7%	-1.2	8552
Haus8	2.0%	0.7%	-1.2	8553
Samd1	2.0%	0.7%	-1.2	8554
Acyp2	2.0%	0.7%	-1.2	8555
Hells	2.0%	0.7%	-1.2	8556
Elf3	2.0%	0.7%	-1.2	8557
RGD1564664	2.0%	0.7%	-1.2	8558
Emp1	2.0%	0.7%	-1.2	8559
LOC108348130	2.0%	0.7%	-1.2	8560
Cdpf1	2.0%	0.7%	-1.2	8561
Fbxo44	2.0%	0.7%	-1.2	8562
Sipa1l1	2.0%	0.7%	-1.2	8563
LOC102548675	2.0%	0.7%	-1.2	8564
Fbxl12	2.0%	0.7%	-1.2	8565
Stxbp5	2.0%	0.7%	-1.2	8566
Pigv	2.0%	0.7%	-1.2	8567
Cfap97	2.0%	0.7%	-1.2	8568
Vps13b	2.0%	0.7%	-1.2	8569
Exd2	2.0%	0.7%	-1.2	8570
Zbtb5	2.0%	0.7%	-1.2	8571
Abcg5	2.0%	0.7%	-1.2	8572
LOC108348324	2.0%	0.7%	-1.2	8573
Dph5	5.9%	4.6%	-1.2	8574

Ncdn	1.6%	0.3%	-1.2	8575
Gab1	1.6%	0.3%	-1.2	8576
Tprn	1.6%	0.3%	-1.2	8577
Atr	1.6%	0.3%	-1.2	8578
Ppp2r3a	1.6%	0.3%	-1.2	8579
Rhobtb2	1.6%	0.3%	-1.2	8580
Pitpnm2	1.6%	0.3%	-1.2	8581
Ep300	1.6%	0.3%	-1.2	8582
Cgref1	1.6%	0.3%	-1.2	8583
Synj1	1.6%	0.3%	-1.2	8584
Dpf3	1.6%	0.3%	-1.2	8585
Urb1	1.6%	0.3%	-1.2	8586
Rspry1	1.6%	0.3%	-1.2	8587
G0s2	43.1%	41.9%	-1.3	8588
Pgs1	5.1%	3.8%	-1.3	8589
LOC102556148	5.1%	3.8%	-1.3	8590
Ezr	4.7%	3.4%	-1.3	8591
Oas1k	4.7%	3.4%	-1.3	8592
Tpk1	4.7%	3.4%	-1.3	8593
Suz12	4.7%	3.4%	-1.3	8594
Prodh	8.6%	7.4%	-1.3	8595
LOC100911840	29.4%	28.1%	-1.3	8596
Cdk16	4.3%	3.0%	-1.3	8597
Dnajc9	4.3%	3.0%	-1.3	8598
Itpk1	4.3%	3.0%	-1.3	8599
LOC682571	4.3%	3.0%	-1.3	8600
Msl3	4.3%	3.0%	-1.3	8601
Hddc2	4.3%	3.0%	-1.3	8602
S100a10	4.3%	3.0%	-1.3	8603
Dnajc8	8.2%	7.0%	-1.3	8604
Cdkn2aipnl	3.9%	2.6%	-1.3	8605
Slc17a9	3.9%	2.6%	-1.3	8606
Mmachc	3.9%	2.6%	-1.3	8607
LOC103690028	3.9%	2.6%	-1.3	8608
Rnf6	3.9%	2.6%	-1.3	8609
Dgcr14	3.5%	2.2%	-1.3	8610
Mtrf1l	3.5%	2.2%	-1.3	8611
Rangrf	3.5%	2.2%	-1.3	8612
Tk1	3.5%	2.2%	-1.3	8613
Stard5	3.5%	2.2%	-1.3	8614
RGD1562394	3.5%	2.2%	-1.3	8615
Relb	3.5%	2.2%	-1.3	8616
Atp8b2	3.5%	2.2%	-1.3	8617
Gpd2	3.5%	2.2%	-1.3	8618
Nt5c3b	3.5%	2.2%	-1.3	8619
Armc8	3.1%	1.8%	-1.3	8620
Kif16b	3.1%	1.8%	-1.3	8621
Senp5	3.1%	1.8%	-1.3	8622
LOC103690164	7.1%	5.8%	-1.3	8623
Rgp1	2.7%	1.4%	-1.3	8624
Vav2	2.7%	1.4%	-1.3	8625
Mb21d2	2.7%	1.4%	-1.3	8626
Dedd2	2.7%	1.4%	-1.3	8627
Sirt6	2.7%	1.4%	-1.3	8628
Prcc	2.7%	1.4%	-1.3	8629
Foxp4	2.7%	1.4%	-1.3	8630
Nfatc3	2.7%	1.4%	-1.3	8631
Rnf38	2.7%	1.4%	-1.3	8632
Gltp	2.7%	1.4%	-1.3	8633
RGD1308601	2.7%	1.4%	-1.3	8634
Faah	6.7%	5.4%	-1.3	8635
Znhit6	6.7%	5.4%	-1.3	8636
Ttll4	6.7%	5.4%	-1.3	8637
Mcm7	2.4%	1.0%	-1.3	8638
Lamc2	2.4%	1.0%	-1.3	8639
Ift122	2.4%	1.0%	-1.3	8640

Zfp385b	2.4%	1.0%	-1.3	8641
Tango2	2.4%	1.0%	-1.3	8642
Slc4a7	2.4%	1.0%	-1.3	8643
Clec16a	2.4%	1.0%	-1.3	8644
Mcur1	2.4%	1.0%	-1.3	8645
Wdr70	2.4%	1.0%	-1.3	8646
LOC108350833	2.4%	1.0%	-1.3	8647
Zfp18	2.4%	1.0%	-1.3	8648
Dhx34	2.4%	1.0%	-1.3	8649
Lrrc20	2.4%	1.0%	-1.3	8650
LOC102554315	2.4%	1.0%	-1.3	8651
Grk5	2.4%	1.0%	-1.3	8652
Uap1	6.3%	5.0%	-1.3	8653
Fuz	2.0%	0.6%	-1.3	8654
Ctu1	2.0%	0.6%	-1.3	8655
Rpp30	2.0%	0.6%	-1.3	8656
Dnajc18	2.0%	0.6%	-1.3	8657
Zfp704	2.0%	0.6%	-1.3	8658
Taf5	2.0%	0.6%	-1.3	8659
Zbtb33	2.0%	0.6%	-1.3	8660
Rbm19	2.0%	0.6%	-1.3	8661
Tcof1	2.0%	0.6%	-1.3	8662
Klf4	2.0%	0.6%	-1.3	8663
Rbmx2	2.0%	0.6%	-1.3	8664
Cdc42bpa	2.0%	0.6%	-1.3	8665
Actr5	2.0%	0.6%	-1.3	8666
Klhl28	2.0%	0.6%	-1.3	8667
Ercc5	2.0%	0.6%	-1.3	8668
Camsap3	2.0%	0.6%	-1.3	8669
Apool	10.2%	8.9%	-1.3	8670
Sbno1	5.9%	4.6%	-1.3	8671
Frrs1	5.9%	4.6%	-1.3	8672
Mok	1.6%	0.2%	-1.3	8673
Fan1	1.6%	0.2%	-1.3	8674
LOC108349129	1.6%	0.2%	-1.3	8675
RGD1305014	1.6%	0.2%	-1.3	8676
MGC95210	1.6%	0.2%	-1.3	8677
Plekhh1	1.6%	0.2%	-1.3	8678
Slc35d1	1.6%	0.2%	-1.3	8679
Dlg5	1.6%	0.2%	-1.3	8680
Uhrf1bp1	1.6%	0.2%	-1.3	8681
Susd1	1.6%	0.2%	-1.3	8682
Meox2	1.6%	0.2%	-1.3	8683
Tada1	1.6%	0.2%	-1.3	8684
Ppp2r3b	1.6%	0.2%	-1.3	8685
RGD1307621	1.6%	0.2%	-1.3	8686
Orc3	1.6%	0.2%	-1.3	8687
Pde5a	1.6%	0.2%	-1.3	8688
Thada	1.6%	0.2%	-1.3	8689
LOC100911305	1.6%	0.2%	-1.3	8690
LOC100911519	1.6%	0.2%	-1.3	8691
Zfp28	1.6%	0.2%	-1.3	8692
Bdh2	5.1%	3.8%	-1.3	8693
Prkd3	5.1%	3.8%	-1.3	8694
Pigbos1	4.7%	3.4%	-1.3	8695
Rnf216	4.7%	3.4%	-1.3	8696
Usp8	4.7%	3.4%	-1.3	8697
Prep	4.7%	3.4%	-1.3	8698
Ppp2r1b	4.7%	3.4%	-1.3	8699
Mff	8.6%	7.3%	-1.4	8700
Tmem38a	25.1%	23.7%	-1.4	8701
Rbp4	100.0%	98.6%	-1.4	8702
Tp53i13	3.9%	2.6%	-1.4	8703
Cdc42ep5	3.9%	2.6%	-1.4	8704
Rsl1d1l1	3.9%	2.6%	-1.4	8705
Ptk2	3.9%	2.6%	-1.4	8706

Dnajc13	3.9%	2.6%	-1.4	8707
A1cf	3.9%	2.6%	-1.4	8708
Stambp	3.9%	2.6%	-1.4	8709
Surf6	3.9%	2.6%	-1.4	8710
Tm7sf3	3.5%	2.2%	-1.4	8711
Asf1a	3.5%	2.2%	-1.4	8712
Tmem219	7.5%	6.1%	-1.4	8713
Utp15	3.1%	1.8%	-1.4	8714
Elmod2	3.1%	1.8%	-1.4	8715
RGD1559904	3.1%	1.8%	-1.4	8716
Baz1b	3.1%	1.8%	-1.4	8717
U2af1l4	3.1%	1.8%	-1.4	8718
Sdr42e1	3.1%	1.8%	-1.4	8719
Klc1	3.1%	1.8%	-1.4	8720
Pak4	2.7%	1.4%	-1.4	8721
LOC108349296	2.7%	1.4%	-1.4	8722
Sergef	2.7%	1.4%	-1.4	8723
Ago1	2.7%	1.4%	-1.4	8724
Dmxl1	2.7%	1.4%	-1.4	8725
Rnaseh1	2.7%	1.4%	-1.4	8726
LOC108348050	2.7%	1.4%	-1.4	8727
Tada3	2.7%	1.4%	-1.4	8728
Usp40	2.7%	1.4%	-1.4	8729
Aqr	2.7%	1.4%	-1.4	8730
Snta1	2.7%	1.4%	-1.4	8731
Unc5b	2.7%	1.4%	-1.4	8732
Smad1	6.7%	5.3%	-1.4	8733
LOC100911055	6.7%	5.3%	-1.4	8734
Mettl25	2.4%	1.0%	-1.4	8735
Nme7	2.4%	1.0%	-1.4	8736
Pdcd2l	2.4%	1.0%	-1.4	8737
Agrn	2.4%	1.0%	-1.4	8738
Ehbp1	2.4%	1.0%	-1.4	8739
Irak4	2.4%	1.0%	-1.4	8740
Frat2	2.4%	1.0%	-1.4	8741
Agpat5	2.4%	1.0%	-1.4	8742
Mettl14	2.4%	1.0%	-1.4	8743
Il3ra	2.4%	1.0%	-1.4	8744
Itga6	2.4%	1.0%	-1.4	8745
Pcif1	2.4%	1.0%	-1.4	8746
LOC102555894	2.4%	1.0%	-1.4	8747
Tcf7l2	2.4%	1.0%	-1.4	8748
Ptbp2	2.4%	1.0%	-1.4	8749
Btrc	2.4%	1.0%	-1.4	8750
Tmem8a	2.4%	1.0%	-1.4	8751
Mtr	2.0%	0.6%	-1.4	8752
Smarcd1	2.0%	0.6%	-1.4	8753
Cox10	2.0%	0.6%	-1.4	8754
L2hgdh	2.0%	0.6%	-1.4	8755
Osgepl1	2.0%	0.6%	-1.4	8756
B9d1	2.0%	0.6%	-1.4	8757
R3hcc1l	2.0%	0.6%	-1.4	8758
LOC683508	2.0%	0.6%	-1.4	8759
Phf7	2.0%	0.6%	-1.4	8760
Vezt	2.0%	0.6%	-1.4	8761
Gtpbp4	2.0%	0.6%	-1.4	8762
Krba1	1.6%	0.2%	-1.4	8763
Kifc2	1.6%	0.2%	-1.4	8764
Flt3lg	1.6%	0.2%	-1.4	8765
Pomt2	1.6%	0.2%	-1.4	8766
Nedd1	1.6%	0.2%	-1.4	8767
Heatr6	1.6%	0.2%	-1.4	8768
Igdcc3	1.6%	0.2%	-1.4	8769
LOC102546541	1.6%	0.2%	-1.4	8770
Wdr34	1.6%	0.2%	-1.4	8771
Usp54	1.6%	0.2%	-1.4	8772

LOC103693416	1.6%	0.2%	-1.4	8773
Vegfc	1.6%	0.2%	-1.4	8774
Zfp622	5.1%	3.7%	-1.4	8775
LOC100912538	4.7%	3.3%	-1.4	8776
Eif4g3	4.7%	3.3%	-1.4	8777
Zfp110	4.7%	3.3%	-1.4	8778
Rab4b	4.3%	2.9%	-1.4	8779
Lrwd1	4.3%	2.9%	-1.4	8780
Golga2	4.3%	2.9%	-1.4	8781
RGD1564469	8.2%	6.8%	-1.4	8782
Mvd	8.2%	6.8%	-1.4	8783
Eri1	3.9%	2.5%	-1.4	8784
Recql	3.9%	2.5%	-1.4	8785
Bcl7b	3.9%	2.5%	-1.4	8786
Lpin3	3.9%	2.5%	-1.4	8787
Mak16	3.9%	2.5%	-1.4	8788
Pik3r2	3.9%	2.5%	-1.4	8789
Dnaja4	99.6%	98.2%	-1.4	8790
Gata4	3.5%	2.1%	-1.5	8791
Adamtsl4	3.5%	2.1%	-1.5	8792
Carm1	3.5%	2.1%	-1.5	8793
Tssc1	3.5%	2.1%	-1.5	8794
Gabpb1	3.5%	2.1%	-1.5	8795
Tfpi	3.5%	2.1%	-1.5	8796
Rbm28	3.5%	2.1%	-1.5	8797
Vps13a	3.5%	2.1%	-1.5	8798
Trim14	7.5%	6.0%	-1.5	8799
Serpina5	3.1%	1.7%	-1.5	8800
Fam222b	3.1%	1.7%	-1.5	8801
Tipin	3.1%	1.7%	-1.5	8802
Fnbp4	3.1%	1.7%	-1.5	8803
Trpm4	3.1%	1.7%	-1.5	8804
Trmt10a	3.1%	1.7%	-1.5	8805
Inpp5a	3.1%	1.7%	-1.5	8806
Mark2	3.1%	1.7%	-1.5	8807
Ctf1	3.1%	1.7%	-1.5	8808
LOC100912042	3.1%	1.7%	-1.5	8809
Noa1	2.7%	1.3%	-1.5	8810
Zfp386	2.7%	1.3%	-1.5	8811
Srr	2.7%	1.3%	-1.5	8812
LOC108351645	2.7%	1.3%	-1.5	8813
Nf2	2.7%	1.3%	-1.5	8814
Dock1	2.7%	1.3%	-1.5	8815
Gid8	2.7%	1.3%	-1.5	8816
Rpain	2.7%	1.3%	-1.5	8817
Magix	2.7%	1.3%	-1.5	8818
LOC100912059	2.7%	1.3%	-1.5	8819
Gas2l1	2.7%	1.3%	-1.5	8820
Coa4	2.7%	1.3%	-1.5	8821
Zzef1	2.7%	1.3%	-1.5	8822
Dcbld1	2.7%	1.3%	-1.5	8823
Pigo	2.7%	1.3%	-1.5	8824
Apbb3	2.7%	1.3%	-1.5	8825
Gstm3	2.7%	1.3%	-1.5	8826
Hus1	2.7%	1.3%	-1.5	8827
Sap30	2.4%	0.9%	-1.5	8828
Slc9a1	2.4%	0.9%	-1.5	8829
Gtdc1	2.4%	0.9%	-1.5	8830
Snx30	2.4%	0.9%	-1.5	8831
Tert	2.4%	0.9%	-1.5	8832
Adarb1	2.4%	0.9%	-1.5	8833
Zdhhc20	2.4%	0.9%	-1.5	8834
Rab40c	2.4%	0.9%	-1.5	8835
Cbfa2t2	2.4%	0.9%	-1.5	8836
Ppcdc	2.4%	0.9%	-1.5	8837
Dcaf10	2.4%	0.9%	-1.5	8838

Gtf2h3	2.4%	0.9%	-1.5	8839
Tsc1	2.4%	0.9%	-1.5	8840
Il15ra	2.4%	0.9%	-1.5	8841
Fam160a2	2.4%	0.9%	-1.5	8842
Bbs4	2.4%	0.9%	-1.5	8843
Dmwd	2.4%	0.9%	-1.5	8844
Mctp2	2.4%	0.9%	-1.5	8845
Zfc3h1	2.4%	0.9%	-1.5	8846
Praf2	2.4%	0.9%	-1.5	8847
Tmem86a	2.4%	0.9%	-1.5	8848
Cmya5	2.4%	0.9%	-1.5	8849
Mlst8	2.4%	0.9%	-1.5	8850
Abcb7	2.4%	0.9%	-1.5	8851
Asb7	2.4%	0.9%	-1.5	8852
Nol10	2.4%	0.9%	-1.5	8853
Isg20l2	2.4%	0.9%	-1.5	8854
Aptx	2.4%	0.9%	-1.5	8855
Mapre2	6.3%	4.8%	-1.5	8856
Nicn1	6.3%	4.8%	-1.5	8857
LOC102551819	2.0%	0.5%	-1.5	8858
Scaper	2.0%	0.5%	-1.5	8859
Adamts7	2.0%	0.5%	-1.5	8860
Foxa3	2.0%	0.5%	-1.5	8861
Sf3b4	2.0%	0.5%	-1.5	8862
Lins1	2.0%	0.5%	-1.5	8863
Slc5a3	2.0%	0.5%	-1.5	8864
Snhg4	2.0%	0.5%	-1.5	8865
Zfp748	2.0%	0.5%	-1.5	8866
Zfp612	2.0%	0.5%	-1.5	8867
Tmem171	2.0%	0.5%	-1.5	8868
Prss53	2.0%	0.5%	-1.5	8869
Npat	2.0%	0.5%	-1.5	8870
Fam110b	2.0%	0.5%	-1.5	8871
Specc1l	2.0%	0.5%	-1.5	8872
Kmt2d	2.0%	0.5%	-1.5	8873
Reg4	2.0%	0.5%	-1.5	8874
Cox11	2.0%	0.5%	-1.5	8875
Lrrc29	2.0%	0.5%	-1.5	8876
Heatr4	2.0%	0.5%	-1.5	8877
Ssh3	2.0%	0.5%	-1.5	8878
Pibf1	2.0%	0.5%	-1.5	8879
Lypla2	10.2%	8.7%	-1.5	8880
Srebf2	10.2%	8.7%	-1.5	8881
Rmdn3	5.9%	4.4%	-1.5	8882
Nim1k	1.6%	0.1%	-1.5	8883
Fam184a	1.6%	0.1%	-1.5	8884
LOC102554419	1.6%	0.1%	-1.5	8885
LOC103694395	1.6%	0.1%	-1.5	8886
Tdrd15	1.6%	0.1%	-1.5	8887
Tesk2	1.6%	0.1%	-1.5	8888
Reps1	1.6%	0.1%	-1.5	8889
Cables1	1.6%	0.1%	-1.5	8890
Slc25a21	1.6%	0.1%	-1.5	8891
Slc1a2	1.6%	0.1%	-1.5	8892
Col6a3	1.6%	0.1%	-1.5	8893
Stk11ip	1.6%	0.1%	-1.5	8894
Zpbp2	1.6%	0.1%	-1.5	8895
Nudt10	1.6%	0.1%	-1.5	8896
H1f0	5.5%	4.0%	-1.5	8897
Sde2	5.1%	3.6%	-1.5	8898
Sall1	5.1%	3.6%	-1.5	8899
Slc35f6	4.7%	3.2%	-1.5	8900
Rffl	4.7%	3.2%	-1.5	8901
Gid4	4.7%	3.2%	-1.5	8902
LOC100364403	4.7%	3.2%	-1.5	8903
Mbd2	4.7%	3.2%	-1.5	8904

RGD1560813	8.6%	7.1%	-1.5	8905
Snx6	4.3%	2.8%	-1.5	8906
Aip	4.3%	2.8%	-1.5	8907
LOC100911959	4.3%	2.8%	-1.5	8908
Arhgef10l	4.3%	2.8%	-1.5	8909
Sec23ip	4.3%	2.8%	-1.5	8910
Cish	12.5%	11.0%	-1.5	8911
Irf7	8.2%	6.7%	-1.5	8912
Fam149b1	3.9%	2.4%	-1.5	8913
Phf3	3.9%	2.4%	-1.5	8914
Klhl7	3.9%	2.4%	-1.5	8915
Tyk2	3.9%	2.4%	-1.5	8916
Med1	3.9%	2.4%	-1.5	8917
Bag5	3.5%	2.0%	-1.5	8918
Slc2a1	3.5%	2.0%	-1.5	8919
Hat1	3.5%	2.0%	-1.5	8920
Gmppb	3.5%	2.0%	-1.5	8921
Them6	3.5%	2.0%	-1.5	8922
Tpr	3.5%	2.0%	-1.5	8923
Rin3	3.5%	2.0%	-1.5	8924
Thap3	3.5%	2.0%	-1.5	8925
Fbrs	3.5%	2.0%	-1.5	8926
LOC287274	7.5%	5.9%	-1.5	8927
Pmm2	7.5%	5.9%	-1.5	8928
Upf2	3.1%	1.6%	-1.5	8929
Slc35c2	3.1%	1.6%	-1.5	8930
Sp3	3.1%	1.6%	-1.5	8931
Slc35b3	3.1%	1.6%	-1.5	8932
RGD1564149	7.1%	5.5%	-1.5	8933
Kmt2a	2.7%	1.2%	-1.5	8934
Vps50	2.7%	1.2%	-1.5	8935
Pim2	2.7%	1.2%	-1.5	8936
Zfp358	2.7%	1.2%	-1.5	8937
Ankrd12	2.7%	1.2%	-1.5	8938
Ubap2	2.7%	1.2%	-1.5	8939
Tmem216	2.7%	1.2%	-1.5	8940
Dis3	2.7%	1.2%	-1.5	8941
Brd9	2.7%	1.2%	-1.5	8942
Tfeb	2.7%	1.2%	-1.5	8943
Ap4b1	2.7%	1.2%	-1.5	8944
LOC108352753	2.7%	1.2%	-1.5	8945
Cnpy4	2.7%	1.2%	-1.5	8946
Slc35e3	2.4%	0.8%	-1.6	8947
Prcp	2.4%	0.8%	-1.6	8948
Cdkal1	2.4%	0.8%	-1.6	8949
Bbip1	2.4%	0.8%	-1.6	8950
Cdk5rap1	2.4%	0.8%	-1.6	8951
Snapc1	2.4%	0.8%	-1.6	8952
Nup93	2.4%	0.8%	-1.6	8953
Pikfyve	2.4%	0.8%	-1.6	8954
Fut8	2.4%	0.8%	-1.6	8955
Tbck	2.4%	0.8%	-1.6	8956
Irgq	2.4%	0.8%	-1.6	8957
Gemin5	2.4%	0.8%	-1.6	8958
Foxk1	2.4%	0.8%	-1.6	8959
Ranbp6	2.4%	0.8%	-1.6	8960
Asns	2.4%	0.8%	-1.6	8961
Ints5	2.4%	0.8%	-1.6	8962
Pknox1	2.4%	0.8%	-1.6	8963
Tanc1	2.4%	0.8%	-1.6	8964
Tmem177	2.4%	0.8%	-1.6	8965
Polr3a	2.4%	0.8%	-1.6	8966
Rasal3	2.4%	0.8%	-1.6	8967
Mbtps2	2.4%	0.8%	-1.6	8968
Anks4b	6.3%	4.7%	-1.6	8969
Zfp503	2.0%	0.4%	-1.6	8970

Adal	2.0%	0.4%	-1.6	8971
LOC103693563	2.0%	0.4%	-1.6	8972
LOC108352595	2.0%	0.4%	-1.6	8973
Sos2	2.0%	0.4%	-1.6	8974
Megf9	2.0%	0.4%	-1.6	8975
Trpc1	2.0%	0.4%	-1.6	8976
Ubtd2	2.0%	0.4%	-1.6	8977
Klhdc8a	2.0%	0.4%	-1.6	8978
Brms1l	2.0%	0.4%	-1.6	8979
Erlin2	5.9%	4.3%	-1.6	8980
Kctd20	5.9%	4.3%	-1.6	8981
Il17rb	5.9%	4.3%	-1.6	8982
NEWGENE_1306455	5.9%	4.3%	-1.6	8983
Aldh3a1	1.6%	0.0%	-1.6	8984
LOC100912610	1.6%	0.0%	-1.6	8985
LOC685482	1.6%	0.0%	-1.6	8986
Mid1	1.6%	0.0%	-1.6	8987
Necab2	1.6%	0.0%	-1.6	8988
Sebox	1.6%	0.0%	-1.6	8989
Zdhhc8	1.6%	0.0%	-1.6	8990
Rbm25l1	9.8%	8.2%	-1.6	8991
Trafd1	5.5%	3.9%	-1.6	8992
Atg4d	5.5%	3.9%	-1.6	8993
LOC100360316	13.7%	12.2%	-1.6	8994
Ltb	5.1%	3.5%	-1.6	8995
Tbl1xr1	5.1%	3.5%	-1.6	8996
Ankrd46	5.1%	3.5%	-1.6	8997
Dut	4.7%	3.1%	-1.6	8998
Pigh	4.7%	3.1%	-1.6	8999
RGD1560523	4.7%	3.1%	-1.6	9000
Cetn3	4.7%	3.1%	-1.6	9001
Setd1b	4.3%	2.7%	-1.6	9002
Epc1	4.3%	2.7%	-1.6	9003
Cox15	4.3%	2.7%	-1.6	9004
RGD1304694	4.3%	2.7%	-1.6	9005
RGD1565766	4.3%	2.7%	-1.6	9006
Trim21	4.3%	2.7%	-1.6	9007
Kng1l1	100.0%	98.4%	-1.6	9008
Serpina3c	100.0%	98.4%	-1.6	9009
LOC102550385	3.9%	2.3%	-1.6	9010
Fam120b	3.9%	2.3%	-1.6	9011
Hdac2	3.9%	2.3%	-1.6	9012
Arpp19	28.6%	27.0%	-1.6	9013
LOC103693777	3.5%	1.9%	-1.6	9014
Armc10	3.5%	1.9%	-1.6	9015
Trim23	3.5%	1.9%	-1.6	9016
Uimc1	3.5%	1.9%	-1.6	9017
Usp4	7.5%	5.8%	-1.6	9018
Setd5	3.1%	1.5%	-1.6	9019
Sike1	3.1%	1.5%	-1.6	9020
Ambra1	3.1%	1.5%	-1.6	9021
Heatr3	3.1%	1.5%	-1.6	9022
Ikbke	3.1%	1.5%	-1.6	9023
Camsap1	2.7%	1.1%	-1.6	9024
Pcgf6	2.7%	1.1%	-1.6	9025
Herc1	2.7%	1.1%	-1.6	9026
Pafah1b3	2.7%	1.1%	-1.6	9027
LOC100912590	2.7%	1.1%	-1.6	9028
Pomgnt1	2.7%	1.1%	-1.6	9029
Ccdc117	2.7%	1.1%	-1.6	9030
Rbm6	2.7%	1.1%	-1.6	9031
Cyp2r1	2.7%	1.1%	-1.6	9032
Wdr33	2.7%	1.1%	-1.6	9033
Riox1	2.7%	1.1%	-1.6	9034
Fam3c	2.7%	1.1%	-1.6	9035
Nup160	2.7%	1.1%	-1.6	9036

Lag3	2.7%	1.1%	-1.6	9037
Mzt1	2.7%	1.1%	-1.6	9038
Msl2	6.7%	5.0%	-1.6	9039
Cecr2	2.4%	0.7%	-1.6	9040
Ercc6l2	2.4%	0.7%	-1.6	9041
Tbc1d8	2.4%	0.7%	-1.6	9042
Nanp	2.4%	0.7%	-1.6	9043
Shroom2	2.4%	0.7%	-1.6	9044
Zbed4	2.4%	0.7%	-1.6	9045
LOC102555672	2.4%	0.7%	-1.6	9046
Dock7	2.4%	0.7%	-1.6	9047
Ppp1r12c	2.4%	0.7%	-1.6	9048
Heatr1	2.4%	0.7%	-1.6	9049
Sh3pxd2a	2.4%	0.7%	-1.6	9050
Cyhr1	2.4%	0.7%	-1.6	9051
Tipinl1	2.4%	0.7%	-1.6	9052
Epn2	2.4%	0.7%	-1.6	9053
Zfp105	2.4%	0.7%	-1.6	9054
Exo5	2.4%	0.7%	-1.6	9055
Prkcz	2.4%	0.7%	-1.6	9056
RGD1305110	2.0%	0.3%	-1.6	9057
Xxylt1	2.0%	0.3%	-1.6	9058
Tcf24	2.0%	0.3%	-1.6	9059
LOC100909803	2.0%	0.3%	-1.6	9060
LOC100362757	2.0%	0.3%	-1.6	9061
Abcc9	2.0%	0.3%	-1.6	9062
LOC102549876	2.0%	0.3%	-1.6	9063
RGD1359108	2.0%	0.3%	-1.6	9064
LOC100910848	2.0%	0.3%	-1.6	9065
Ubxn2b	2.0%	0.3%	-1.6	9066
Pdia4	5.9%	4.2%	-1.6	9067
Dnajc30	5.5%	3.8%	-1.7	9068
Cldn7	9.4%	7.8%	-1.7	9069
Cnpy3	5.1%	3.4%	-1.7	9070
Casp7	5.1%	3.4%	-1.7	9071
Arfip1	5.1%	3.4%	-1.7	9072
Clcn3	4.7%	3.0%	-1.7	9073
Atp11b	4.7%	3.0%	-1.7	9074
RGD1308750	4.3%	2.6%	-1.7	9075
Tfip11	4.3%	2.6%	-1.7	9076
Anxa2	4.3%	2.6%	-1.7	9077
Pex11a	4.3%	2.6%	-1.7	9078
LOC103692785	25.1%	23.4%	-1.7	9079
Kcnn2	3.9%	2.2%	-1.7	9080
Pom121	3.9%	2.2%	-1.7	9081
Cblb	3.9%	2.2%	-1.7	9082
Ppme1	3.9%	2.2%	-1.7	9083
Pithd1	3.9%	2.2%	-1.7	9084
Parp2	3.9%	2.2%	-1.7	9085
Srrt	7.8%	6.2%	-1.7	9086
Nudcd1	3.5%	1.8%	-1.7	9087
LOC103690064	3.5%	1.8%	-1.7	9088
Fam208a	3.5%	1.8%	-1.7	9089
Hcfc1	3.5%	1.8%	-1.7	9090
Cklf	3.5%	1.8%	-1.7	9091
Ccdc174	3.1%	1.4%	-1.7	9092
Arhgap18	3.1%	1.4%	-1.7	9093
Smarcad1	3.1%	1.4%	-1.7	9094
Fam53a	3.1%	1.4%	-1.7	9095
Metrnl	3.1%	1.4%	-1.7	9096
Dnajc11	3.1%	1.4%	-1.7	9097
Tyw5	3.1%	1.4%	-1.7	9098
Mettl10	2.7%	1.0%	-1.7	9099
Ppm1d	2.7%	1.0%	-1.7	9100
Ccdc85b	2.7%	1.0%	-1.7	9101
LOC100360380	2.7%	1.0%	-1.7	9102

Ing5	2.7%	1.0%	-1.7	9103
Hook2	2.7%	1.0%	-1.7	9104
Cped1	2.7%	1.0%	-1.7	9105
Rpap1	2.7%	1.0%	-1.7	9106
Rxrb	2.7%	1.0%	-1.7	9107
Zfp84	2.7%	1.0%	-1.7	9108
LOC679217	52.5%	50.8%	-1.7	9109
Fbxo21	2.4%	0.6%	-1.7	9110
Appl1	2.4%	0.6%	-1.7	9111
Zfp518b	2.4%	0.6%	-1.7	9112
Ears2	2.4%	0.6%	-1.7	9113
Proser2	2.4%	0.6%	-1.7	9114
Katnb1	2.4%	0.6%	-1.7	9115
LOC102548450	2.4%	0.6%	-1.7	9116
Lbh	2.4%	0.6%	-1.7	9117
Bspry	2.4%	0.6%	-1.7	9118
Eml3	2.4%	0.6%	-1.7	9119
Tef	2.4%	0.6%	-1.7	9120
Ift140	2.4%	0.6%	-1.7	9121
Riox2	2.4%	0.6%	-1.7	9122
Nags	10.6%	8.9%	-1.7	9123
Xk	2.0%	0.2%	-1.7	9124
LOC108348251	2.0%	0.2%	-1.7	9125
Fcho2	2.0%	0.2%	-1.7	9126
Rbp7	2.0%	0.2%	-1.7	9127
LOC100909392	2.0%	0.2%	-1.7	9128
Rad18	2.0%	0.2%	-1.7	9129
Pim1	2.0%	0.2%	-1.7	9130
Zxdb	2.0%	0.2%	-1.7	9131
Fchsd1	2.0%	0.2%	-1.7	9132
Taf1a	2.0%	0.2%	-1.7	9133
Man1a2	5.9%	4.2%	-1.7	9134
Hsph1	14.1%	12.4%	-1.7	9135
LOC692005	18.0%	16.3%	-1.7	9136
Gale	5.5%	3.8%	-1.7	9137
Msh3	5.5%	3.8%	-1.7	9138
Topors	5.1%	3.4%	-1.7	9139
Ssbp3	5.1%	3.4%	-1.7	9140
Myo10	4.7%	3.0%	-1.7	9141
Xpo1	4.7%	3.0%	-1.7	9142
Ate1	4.7%	3.0%	-1.7	9143
LOC108349031	4.7%	3.0%	-1.7	9144
Utp14a	4.3%	2.6%	-1.8	9145
Reep4	4.3%	2.6%	-1.8	9146
Stim2	4.3%	2.6%	-1.8	9147
Ahctf1	4.3%	2.6%	-1.8	9148
Lsg1	4.3%	2.6%	-1.8	9149
Me1	12.5%	10.8%	-1.8	9150
Top1mt	3.9%	2.2%	-1.8	9151
Nudt16l1	3.9%	2.2%	-1.8	9152
Slc16a11	3.9%	2.2%	-1.8	9153
Atg10	3.9%	2.2%	-1.8	9154
H2afx	3.9%	2.2%	-1.8	9155
Mfap1a	3.9%	2.2%	-1.8	9156
Rpp25l	3.9%	2.2%	-1.8	9157
Cul5	3.9%	2.2%	-1.8	9158
Cipc	3.9%	2.2%	-1.8	9159
LOC102554269	7.8%	6.1%	-1.8	9160
Arl3	7.8%	6.1%	-1.8	9161
Mtmr3	3.5%	1.8%	-1.8	9162
Prepl	3.5%	1.8%	-1.8	9163
Fgfrl1	3.5%	1.8%	-1.8	9164
Med13	3.1%	1.4%	-1.8	9165
Bahd1	3.1%	1.4%	-1.8	9166
Atf7ip	3.1%	1.4%	-1.8	9167
Tfcp2	3.1%	1.4%	-1.8	9168

Ppp1r8	3.1%	1.4%	-1.8	9169
Nipal2	2.7%	1.0%	-1.8	9170
Slc25a19	2.7%	1.0%	-1.8	9171
Gdpd1	2.7%	1.0%	-1.8	9172
Ccdc94	2.7%	1.0%	-1.8	9173
Bcl2l11	2.7%	1.0%	-1.8	9174
Gpcpd1	2.7%	1.0%	-1.8	9175
Zfp326	2.7%	1.0%	-1.8	9176
Herc2	2.7%	1.0%	-1.8	9177
LOC102555644	2.7%	1.0%	-1.8	9178
Katnbl1	2.7%	1.0%	-1.8	9179
Cep170b	2.7%	1.0%	-1.8	9180
Nfrkb	2.7%	1.0%	-1.8	9181
Zfp318	2.7%	1.0%	-1.8	9182
Pdcd7	2.7%	1.0%	-1.8	9183
Glt8d1	2.7%	1.0%	-1.8	9184
Phlda1	6.7%	4.9%	-1.8	9185
Lrrc46	6.7%	4.9%	-1.8	9186
Dcun1d4	6.7%	4.9%	-1.8	9187
Pggt1b	2.4%	0.6%	-1.8	9188
Trim39	2.4%	0.6%	-1.8	9189
Alkbh4	2.4%	0.6%	-1.8	9190
Mcf2l	2.4%	0.6%	-1.8	9191
Mars2	2.4%	0.6%	-1.8	9192
Ptcd2	2.4%	0.6%	-1.8	9193
LOC103694321	2.4%	0.6%	-1.8	9194
Zfp609	2.4%	0.6%	-1.8	9195
Itfg2	2.4%	0.6%	-1.8	9196
LOC100911221	2.4%	0.6%	-1.8	9197
Zfp428	2.4%	0.6%	-1.8	9198
Tctn1	2.0%	0.2%	-1.8	9199
LOC500124	2.0%	0.2%	-1.8	9200
Micall2	2.0%	0.2%	-1.8	9201
Zc3h10	2.0%	0.2%	-1.8	9202
Dclre1b	2.0%	0.2%	-1.8	9203
LOC103694214	2.0%	0.2%	-1.8	9204
Zdhhc13	2.0%	0.2%	-1.8	9205
RGD1309079	5.9%	4.1%	-1.8	9206
Setd4	5.5%	3.7%	-1.8	9207
RGD1562451	5.5%	3.7%	-1.8	9208
LOC108349189	13.7%	11.9%	-1.8	9209
Cutc	5.1%	3.3%	-1.8	9210
Med11	5.1%	3.3%	-1.8	9211
Scpep1	9.0%	7.2%	-1.8	9212
Lsm8	4.7%	2.9%	-1.8	9213
Pex11g	4.7%	2.9%	-1.8	9214
Ces2c	4.7%	2.9%	-1.8	9215
Ccdc90b	4.7%	2.9%	-1.8	9216
Zfp36l1	37.6%	35.8%	-1.8	9217
Nxt2	4.3%	2.5%	-1.8	9218
Rbl2	4.3%	2.5%	-1.8	9219
Mettl6	4.3%	2.5%	-1.8	9220
Gpalpp1	4.3%	2.5%	-1.8	9221
Mnat1	4.3%	2.5%	-1.8	9222
Ints12	45.9%	44.0%	-1.8	9223
Rup2	100.0%	98.2%	-1.8	9224
LOC103690153	8.2%	6.4%	-1.8	9225
Mknk2	16.5%	14.6%	-1.8	9226
Tmem2	3.9%	2.1%	-1.8	9227
Txlng	3.9%	2.1%	-1.8	9228
Nub1	3.9%	2.1%	-1.8	9229
LOC108348085	3.9%	2.1%	-1.8	9230
Vps33a	3.9%	2.1%	-1.8	9231
Dnajc17	3.9%	2.1%	-1.8	9232
Clk2	3.5%	1.7%	-1.9	9233
LOC100909830	3.5%	1.7%	-1.9	9234

Ddhd2	3.5%	1.7%	-1.9	9235
Tmem69	3.5%	1.7%	-1.9	9236
Ncoa6	3.5%	1.7%	-1.9	9237
Gpatch8	3.5%	1.7%	-1.9	9238
Ctnnbip1	3.5%	1.7%	-1.9	9239
Slk	3.5%	1.7%	-1.9	9240
Med7	3.5%	1.7%	-1.9	9241
Dimt1	3.5%	1.7%	-1.9	9242
Mospd1	3.5%	1.7%	-1.9	9243
Stx2	3.5%	1.7%	-1.9	9244
Set	11.8%	9.9%	-1.9	9245
Apoa2	99.2%	97.4%	-1.9	9246
Tmem186	7.5%	5.6%	-1.9	9247
Uck1	3.1%	1.3%	-1.9	9248
Rfc2	3.1%	1.3%	-1.9	9249
Numa1	3.1%	1.3%	-1.9	9250
Homer1	3.1%	1.3%	-1.9	9251
Myo9b	3.1%	1.3%	-1.9	9252
Taf2	3.1%	1.3%	-1.9	9253
Fam206a	3.1%	1.3%	-1.9	9254
Mrgbp	3.1%	1.3%	-1.9	9255
Fra10ac1	3.1%	1.3%	-1.9	9256
Zfp46	2.7%	0.9%	-1.9	9257
Zcchc8	2.7%	0.9%	-1.9	9258
Nup205	2.7%	0.9%	-1.9	9259
Fam204a	2.7%	0.9%	-1.9	9260
Ssh2	2.7%	0.9%	-1.9	9261
Ttc39b	2.7%	0.9%	-1.9	9262
Lzic	2.7%	0.9%	-1.9	9263
Cyb5d2	2.7%	0.9%	-1.9	9264
Wdyhv1	2.7%	0.9%	-1.9	9265
Fuom	2.7%	0.9%	-1.9	9266
Ndst2	2.7%	0.9%	-1.9	9267
Ift46	6.7%	4.8%	-1.9	9268
Meis1	2.4%	0.5%	-1.9	9269
Tecpr1	2.4%	0.5%	-1.9	9270
Gli4	2.4%	0.5%	-1.9	9271
Zbtb11os1	2.4%	0.5%	-1.9	9272
Faap100	2.4%	0.5%	-1.9	9273
Zfp292	2.4%	0.5%	-1.9	9274
Abhd10	2.4%	0.5%	-1.9	9275
Siah1	2.4%	0.5%	-1.9	9276
Creb3l2	2.4%	0.5%	-1.9	9277
Pou6f1	2.4%	0.5%	-1.9	9278
Ubiad1	2.4%	0.5%	-1.9	9279
Bcl2l2	2.4%	0.5%	-1.9	9280
Las1l	6.3%	4.4%	-1.9	9281
Srf	6.3%	4.4%	-1.9	9282
Prkrip1	6.3%	4.4%	-1.9	9283
Etv5	2.0%	0.1%	-1.9	9284
LOC108351535	2.0%	0.1%	-1.9	9285
Carnmt1	5.9%	4.0%	-1.9	9286
LOC100912380	14.1%	12.2%	-1.9	9287
Agap3	5.5%	3.6%	-1.9	9288
Sumf1	5.5%	3.6%	-1.9	9289
Cab39l	5.5%	3.6%	-1.9	9290
Noct	5.5%	3.6%	-1.9	9291
Taf5l	5.1%	3.2%	-1.9	9292
Rbm4	5.1%	3.2%	-1.9	9293
Poll	5.1%	3.2%	-1.9	9294
Bin1	5.1%	3.2%	-1.9	9295
Pank1	21.6%	19.7%	-1.9	9296
Plekha5	4.7%	2.8%	-1.9	9297
Urm1	4.7%	2.8%	-1.9	9298
Galnt11	4.7%	2.8%	-1.9	9299
Mcrs1	4.3%	2.4%	-1.9	9300

Hykk	4.3%	2.4%	-1.9	9301
Gga1	4.3%	2.4%	-1.9	9302
Tada2b	3.9%	2.0%	-1.9	9303
Rcbtb1	3.9%	2.0%	-1.9	9304
Fgfr1op	3.9%	2.0%	-1.9	9305
Tmem60	3.9%	2.0%	-1.9	9306
Osbpl8	3.9%	2.0%	-1.9	9307
Dgka	3.9%	2.0%	-1.9	9308
Rabepk	3.9%	2.0%	-1.9	9309
RGD1305938	3.9%	2.0%	-1.9	9310
Inha	24.7%	22.8%	-1.9	9311
Pih1d1	3.5%	1.6%	-1.9	9312
Hebp2	3.5%	1.6%	-1.9	9313
Tcf12	3.5%	1.6%	-1.9	9314
Rfxap	3.5%	1.6%	-1.9	9315
Zmat1	3.1%	1.2%	-1.9	9316
Tspan33	3.1%	1.2%	-1.9	9317
Kbtbd4	3.1%	1.2%	-1.9	9318
Gtf3c3	3.1%	1.2%	-1.9	9319
LOC102555289	3.1%	1.2%	-1.9	9320
Plekha4	3.1%	1.2%	-1.9	9321
Focad	3.1%	1.2%	-1.9	9322
Thoc6	3.1%	1.2%	-1.9	9323
Mageh1	3.1%	1.2%	-1.9	9324
Coro7	3.1%	1.2%	-1.9	9325
LOC100909569	3.1%	1.2%	-1.9	9326
Chd6	3.1%	1.2%	-1.9	9327
Neo1	3.1%	1.2%	-1.9	9328
Dock9	7.1%	5.1%	-1.9	9329
Luzp1	2.7%	0.8%	-1.9	9330
Ilf3	2.7%	0.8%	-1.9	9331
Taf6l	2.7%	0.8%	-1.9	9332
Omd	2.7%	0.8%	-1.9	9333
Anapc10	2.7%	0.8%	-1.9	9334
Trmt12	2.7%	0.8%	-1.9	9335
Kbtbd8	2.7%	0.8%	-1.9	9336
RGD1562200	2.7%	0.8%	-1.9	9337
Brwd3	2.7%	0.8%	-1.9	9338
Ccdc6	2.7%	0.8%	-1.9	9339
Epb41l1	2.7%	0.8%	-1.9	9340
Slc35d2	2.7%	0.8%	-1.9	9341
Efna5	6.7%	4.7%	-2.0	9342
Casp2	2.4%	0.4%	-2.0	9343
Bbs5	2.4%	0.4%	-2.0	9344
Kdelc2	2.4%	0.4%	-2.0	9345
Kank2	2.4%	0.4%	-2.0	9346
Ppp1r13l	2.4%	0.4%	-2.0	9347
Pdss1	2.4%	0.4%	-2.0	9348
Mapk8ip1	2.4%	0.4%	-2.0	9349
E2f3	2.4%	0.4%	-2.0	9350
LOC103689945	2.4%	0.4%	-2.0	9351
LOC103690024	6.3%	4.3%	-2.0	9352
Slc50a1	6.3%	4.3%	-2.0	9353
Cbx1	6.3%	4.3%	-2.0	9354
Mfsd12	2.0%	0.0%	-2.0	9355
Dync1li2	5.9%	3.9%	-2.0	9356
Rpia	5.9%	3.9%	-2.0	9357
LOC100361025	5.9%	3.9%	-2.0	9358
Xylt2	5.1%	3.1%	-2.0	9359
Atg4a	5.1%	3.1%	-2.0	9360
Slc39a11	5.1%	3.1%	-2.0	9361
Fyco1	5.1%	3.1%	-2.0	9362
Dhx38	5.1%	3.1%	-2.0	9363
Wtap	9.0%	7.0%	-2.0	9364
Adra1b	9.0%	7.0%	-2.0	9365
Gar1	4.7%	2.7%	-2.0	9366

LOC678796	4.7%	2.7%	-2.0	9367
Vasp	4.7%	2.7%	-2.0	9368
Zfp219	4.7%	2.7%	-2.0	9369
LOC102553272	4.7%	2.7%	-2.0	9370
Cpsf4	8.6%	6.6%	-2.0	9371
RT1-CE10	16.9%	14.9%	-2.0	9372
Scyl3	4.3%	2.3%	-2.0	9373
Det1	4.3%	2.3%	-2.0	9374
Hspb11	4.3%	2.3%	-2.0	9375
RGD1311745	4.3%	2.3%	-2.0	9376
Mtrf1	4.3%	2.3%	-2.0	9377
Dctn1	4.3%	2.3%	-2.0	9378
Rhbdd2	4.3%	2.3%	-2.0	9379
Snd1	12.5%	10.6%	-2.0	9380
C3	100.0%	98.0%	-2.0	9381
Ppp6r2	3.9%	1.9%	-2.0	9382
Tgs1	3.9%	1.9%	-2.0	9383
Fbxo38	3.9%	1.9%	-2.0	9384
Xpo4	3.9%	1.9%	-2.0	9385
Ddx19b	3.9%	1.9%	-2.0	9386
Pi4kb	3.5%	1.5%	-2.0	9387
Orc2	3.5%	1.5%	-2.0	9388
Tmem65	3.5%	1.5%	-2.0	9389
Smad2	3.5%	1.5%	-2.0	9390
Prkx	3.5%	1.5%	-2.0	9391
Jam3	3.5%	1.5%	-2.0	9392
LOC680039	3.5%	1.5%	-2.0	9393
Asun	3.5%	1.5%	-2.0	9394
Utp6	11.8%	9.8%	-2.0	9395
Phykpl	7.5%	5.4%	-2.0	9396
Lmf2	7.5%	5.4%	-2.0	9397
LOC108348422	3.1%	1.1%	-2.0	9398
Hivep2	3.1%	1.1%	-2.0	9399
Ppm1f	3.1%	1.1%	-2.0	9400
Pik3r4	3.1%	1.1%	-2.0	9401
Kdm8	3.1%	1.1%	-2.0	9402
Rapgef2	3.1%	1.1%	-2.0	9403
Zmynd19	3.1%	1.1%	-2.0	9404
Zfp511	3.1%	1.1%	-2.0	9405
Cux1	3.1%	1.1%	-2.0	9406
Slc35c1	3.1%	1.1%	-2.0	9407
Eif2ak4	3.1%	1.1%	-2.0	9408
Dnajc24	3.1%	1.1%	-2.0	9409
Fam114a2	3.1%	1.1%	-2.0	9410
LOC100911177	15.3%	13.3%	-2.0	9411
Myrf	2.7%	0.7%	-2.0	9412
Tmem252	2.7%	0.7%	-2.0	9413
Tle2	2.7%	0.7%	-2.0	9414
Dio3	2.7%	0.7%	-2.0	9415
Extl1	2.7%	0.7%	-2.0	9416
Parp6	2.7%	0.7%	-2.0	9417
Ap5z1	2.7%	0.7%	-2.0	9418
R3hdm1	2.7%	0.7%	-2.0	9419
Fam151b	2.7%	0.7%	-2.0	9420
Dcst1	2.7%	0.7%	-2.0	9421
LOC680989	2.7%	0.7%	-2.0	9422
Wnt4	2.7%	0.7%	-2.0	9423
Sema4b	2.7%	0.7%	-2.0	9424
Nagpa	2.7%	0.7%	-2.0	9425
Sin3a	2.7%	0.7%	-2.0	9426
Mettl18	2.7%	0.7%	-2.0	9427
Ubd	65.1%	63.1%	-2.0	9428
LOC100910885	6.7%	4.6%	-2.0	9429
Yeats2	2.4%	0.3%	-2.0	9430
Atf7	2.4%	0.3%	-2.0	9431
Slc28a2	10.2%	8.2%	-2.0	9432

Cd276	93.3%	91.3%	-2.0	9433
Hmgb2	43.1%	41.1%	-2.1	9434
Aldh8a1	9.8%	7.8%	-2.1	9435
Zbed3	5.5%	3.4%	-2.1	9436
Entpd4	5.5%	3.4%	-2.1	9437
Tmem55a	5.5%	3.4%	-2.1	9438
Inpp1	5.5%	3.4%	-2.1	9439
Cks2	9.4%	7.4%	-2.1	9440
Nxt1	5.1%	3.0%	-2.1	9441
Vps37b	5.1%	3.0%	-2.1	9442
Gmeb1	5.1%	3.0%	-2.1	9443
Atxn7l3	4.7%	2.6%	-2.1	9444
Aspa	4.7%	2.6%	-2.1	9445
Wdr48	4.7%	2.6%	-2.1	9446
Ofd1	4.7%	2.6%	-2.1	9447
Aff4	12.9%	10.9%	-2.1	9448
Denr	8.6%	6.6%	-2.1	9449
Wasf1	8.6%	6.6%	-2.1	9450
LOC102555183	4.3%	2.2%	-2.1	9451
Desi1	4.3%	2.2%	-2.1	9452
Mapkap1	4.3%	2.2%	-2.1	9453
Nsd1	4.3%	2.2%	-2.1	9454
Pdzd8	4.3%	2.2%	-2.1	9455
LOC108351584	4.3%	2.2%	-2.1	9456
Btg3	4.3%	2.2%	-2.1	9457
Gnb1	12.5%	10.5%	-2.1	9458
Foxn3	8.2%	6.2%	-2.1	9459
Shroom1	3.9%	1.8%	-2.1	9460
Rbm27	3.9%	1.8%	-2.1	9461
LOC100911261	3.9%	1.8%	-2.1	9462
Moap1	3.9%	1.8%	-2.1	9463
Wrnip1	3.9%	1.8%	-2.1	9464
Alkbh2	3.9%	1.8%	-2.1	9465
Dpf2	3.9%	1.8%	-2.1	9466
Lace1	3.5%	1.4%	-2.1	9467
Cenpm	3.5%	1.4%	-2.1	9468
Bmpr1a	3.5%	1.4%	-2.1	9469
Trappc9	3.5%	1.4%	-2.1	9470
RGD1562114	3.5%	1.4%	-2.1	9471
Pip4k2b	3.5%	1.4%	-2.1	9472
Rhoq	3.5%	1.4%	-2.1	9473
Pick1	3.5%	1.4%	-2.1	9474
Dennd2a	3.5%	1.4%	-2.1	9475
Epm2aip1	3.5%	1.4%	-2.1	9476
Zfp800	3.5%	1.4%	-2.1	9477
LOC103690163	3.5%	1.4%	-2.1	9478
Zfp280c	3.5%	1.4%	-2.1	9479
Tbcb	7.5%	5.4%	-2.1	9480
Mfap3	3.1%	1.0%	-2.1	9481
Car7	3.1%	1.0%	-2.1	9482
LOC108348326	3.1%	1.0%	-2.1	9483
Sned1	3.1%	1.0%	-2.1	9484
Zfp189	3.1%	1.0%	-2.1	9485
RGD1305455	3.1%	1.0%	-2.1	9486
Akt1s1	7.1%	5.0%	-2.1	9487
Btbd7	2.7%	0.6%	-2.1	9488
Specc1	2.7%	0.6%	-2.1	9489
Gse1	2.7%	0.6%	-2.1	9490
LOC108348069	2.7%	0.6%	-2.1	9491
Zfp467	2.7%	0.6%	-2.1	9492
Pcnx3	2.7%	0.6%	-2.1	9493
Lysmd2	2.7%	0.6%	-2.1	9494
Tns2	2.7%	0.6%	-2.1	9495
Rev3l	2.7%	0.6%	-2.1	9496
Sestd1	2.7%	0.6%	-2.1	9497
Bcr	2.7%	0.6%	-2.1	9498

Cmtr2	2.7%	0.6%	-2.1	9499
Tab3	2.7%	0.6%	-2.1	9500
RGD1560341	6.7%	4.6%	-2.1	9501
Map3k6	2.4%	0.2%	-2.1	9502
Rusc2	2.4%	0.2%	-2.1	9503
Dstyk	2.4%	0.2%	-2.1	9504
Pars2	2.4%	0.2%	-2.1	9505
L3mbtl3	2.4%	0.2%	-2.1	9506
Gmfg	5.9%	3.8%	-2.1	9507
Crebzf	5.5%	3.4%	-2.1	9508
Gsk3a	5.5%	3.4%	-2.1	9509
Larp7	9.4%	7.3%	-2.1	9510
Cbfb	9.4%	7.3%	-2.1	9511
Tmem128	9.4%	7.3%	-2.1	9512
Fuca2	5.1%	3.0%	-2.1	9513
LOC103694876	5.1%	3.0%	-2.1	9514
Rab34	5.1%	3.0%	-2.1	9515
Endog	5.1%	3.0%	-2.1	9516
Cdc27	5.1%	3.0%	-2.1	9517
Rsrc1	5.1%	3.0%	-2.1	9518
Rpf2	5.1%	3.0%	-2.1	9519
LOC100912481	5.1%	3.0%	-2.1	9520
Ncor2	9.0%	6.9%	-2.1	9521
Rsl1d1	4.7%	2.6%	-2.1	9522
Tprkb	4.7%	2.6%	-2.1	9523
Pus7	4.7%	2.6%	-2.1	9524
Mospd2	4.7%	2.6%	-2.1	9525
Dennd4c	4.3%	2.2%	-2.2	9526
Wdr41	4.3%	2.2%	-2.2	9527
Apoc3	100.0%	97.8%	-2.2	9528
Crebbp	3.9%	1.8%	-2.2	9529
LOC100909795	3.9%	1.8%	-2.2	9530
L3mbtl2	3.5%	1.4%	-2.2	9531
Pcnx1	3.5%	1.4%	-2.2	9532
Rcc1	3.5%	1.4%	-2.2	9533
Tnfrsf21	3.5%	1.4%	-2.2	9534
Zfyve27	3.5%	1.4%	-2.2	9535
RGD1305350	3.5%	1.4%	-2.2	9536
Brwd1	3.5%	1.4%	-2.2	9537
RGD1564801	3.5%	1.4%	-2.2	9538
Pak1ip1	3.5%	1.4%	-2.2	9539
Cobll1	3.5%	1.4%	-2.2	9540
Mrm2	3.5%	1.4%	-2.2	9541
Vil1	3.5%	1.4%	-2.2	9542
Cd24	3.5%	1.4%	-2.2	9543
Dbnl	7.5%	5.3%	-2.2	9544
Ptges3l1	15.7%	13.5%	-2.2	9545
Trmt5	3.1%	1.0%	-2.2	9546
Klf16	3.1%	1.0%	-2.2	9547
Tmco6	3.1%	1.0%	-2.2	9548
Ezh1	3.1%	1.0%	-2.2	9549
Pold3	3.1%	1.0%	-2.2	9550
Cdca4	3.1%	1.0%	-2.2	9551
Tepsin	3.1%	1.0%	-2.2	9552
Snupn	3.1%	1.0%	-2.2	9553
Tefm	3.1%	1.0%	-2.2	9554
Adgra3	3.1%	1.0%	-2.2	9555
Zfp317	3.1%	1.0%	-2.2	9556
Trim34	3.1%	1.0%	-2.2	9557
Magt1	3.1%	1.0%	-2.2	9558
LOC100360200	7.1%	4.9%	-2.2	9559
Irf9	7.1%	4.9%	-2.2	9560
Smarca5	7.1%	4.9%	-2.2	9561
Ccdc17	2.7%	0.6%	-2.2	9562
Ric1	2.7%	0.6%	-2.2	9563
Tle3	2.7%	0.6%	-2.2	9564

Tbc1d8b	2.7%	0.6%	-2.2	9565
LOC102556175	2.7%	0.6%	-2.2	9566
Rhbdf1	2.7%	0.6%	-2.2	9567
Lynx1	2.7%	0.6%	-2.2	9568
Btbd11	2.7%	0.6%	-2.2	9569
Rubcn	2.7%	0.6%	-2.2	9570
B3galt4	2.7%	0.6%	-2.2	9571
Sept10	2.7%	0.6%	-2.2	9572
Srsf4	6.7%	4.5%	-2.2	9573
Crtc3	2.4%	0.2%	-2.2	9574
Sox9	2.4%	0.2%	-2.2	9575
Pomgnt2	2.4%	0.2%	-2.2	9576
Whsc1	2.4%	0.2%	-2.2	9577
Txndc16	2.4%	0.2%	-2.2	9578
Marveld3	2.4%	0.2%	-2.2	9579
LOC108351936	23.1%	20.9%	-2.2	9580
RGD1308134	6.3%	4.1%	-2.2	9581
Baiap2	6.3%	4.1%	-2.2	9582
Stag2	6.3%	4.1%	-2.2	9583
Pfkl	5.9%	3.7%	-2.2	9584
Ascc1	5.9%	3.7%	-2.2	9585
Otud6b	5.9%	3.7%	-2.2	9586
LOC103690054	5.9%	3.7%	-2.2	9587
Ube2h	9.8%	7.6%	-2.2	9588
Safb2	5.5%	3.3%	-2.2	9589
Mrps27	5.5%	3.3%	-2.2	9590
Snrnp48	5.5%	3.3%	-2.2	9591
Ddx39b	9.4%	7.2%	-2.2	9592
Dpm3	5.1%	2.9%	-2.2	9593
Trmt61a	5.1%	2.9%	-2.2	9594
Zfp644	5.1%	2.9%	-2.2	9595
Hps4	5.1%	2.9%	-2.2	9596
Snapc2	5.1%	2.9%	-2.2	9597
Dmd	5.1%	2.9%	-2.2	9598
Zc3h7b	5.1%	2.9%	-2.2	9599
Vsig10	4.7%	2.5%	-2.2	9600
Vps37a	4.7%	2.5%	-2.2	9601
Smarcd2	4.7%	2.5%	-2.2	9602
Ptpn9	4.7%	2.5%	-2.2	9603
Srek1	4.7%	2.5%	-2.2	9604
Nsun4	4.7%	2.5%	-2.2	9605
Aggf1	4.7%	2.5%	-2.2	9606
Ubp1	4.7%	2.5%	-2.2	9607
Vmac	4.3%	2.1%	-2.2	9608
Sgk2	4.3%	2.1%	-2.2	9609
RGD1566359	4.3%	2.1%	-2.2	9610
Plxna2	3.9%	1.7%	-2.2	9611
Nup88	3.9%	1.7%	-2.2	9612
Drosha	3.9%	1.7%	-2.2	9613
Cluap1	3.9%	1.7%	-2.2	9614
Atp5s	3.9%	1.7%	-2.2	9615
Ccnt2	3.9%	1.7%	-2.2	9616
Polr1a	3.9%	1.7%	-2.2	9617
Fance	3.9%	1.7%	-2.2	9618
LOC103690937	3.9%	1.7%	-2.2	9619
Atrx	3.9%	1.7%	-2.2	9620
Bloc1s4	7.8%	5.6%	-2.2	9621
Fntb	7.8%	5.6%	-2.2	9622
Bloc1s2	7.8%	5.6%	-2.2	9623
Lonp1	16.1%	13.8%	-2.2	9624
Ddx20	3.5%	1.3%	-2.3	9625
Nr2c1	3.5%	1.3%	-2.3	9626
Cpeb3	3.5%	1.3%	-2.3	9627
Rbbp5	3.5%	1.3%	-2.3	9628
N4bp2	3.5%	1.3%	-2.3	9629
LOC100910540	3.5%	1.3%	-2.3	9630

Rhno1	3.5%	1.3%	-2.3	9631
Uba7	3.5%	1.3%	-2.3	9632
Kat6b	3.5%	1.3%	-2.3	9633
Hdac8	3.5%	1.3%	-2.3	9634
RGD1565775	3.5%	1.3%	-2.3	9635
Tagln2	11.8%	9.5%	-2.3	9636
Cyb561a3	3.1%	0.9%	-2.3	9637
Mlxip	3.1%	0.9%	-2.3	9638
Leng9	3.1%	0.9%	-2.3	9639
Rngtt	3.1%	0.9%	-2.3	9640
Klc2	3.1%	0.9%	-2.3	9641
Casp9	3.1%	0.9%	-2.3	9642
Zfp354a	3.1%	0.9%	-2.3	9643
Msantd2	3.1%	0.9%	-2.3	9644
LOC100910275	3.1%	0.9%	-2.3	9645
Tomm34	3.1%	0.9%	-2.3	9646
Grk2	3.1%	0.9%	-2.3	9647
Snx16	2.7%	0.5%	-2.3	9648
Myo6	2.7%	0.5%	-2.3	9649
LOC100909750	2.7%	0.5%	-2.3	9650
Ndrg1	2.7%	0.5%	-2.3	9651
Kif2a	2.7%	0.5%	-2.3	9652
Usp53	2.7%	0.5%	-2.3	9653
Slc2a8	2.7%	0.5%	-2.3	9654
Fkrp	2.7%	0.5%	-2.3	9655
Ctnnal1	2.7%	0.5%	-2.3	9656
Dhx40	2.7%	0.5%	-2.3	9657
LOC102552147	6.7%	4.4%	-2.3	9658
LOC103693584	2.4%	0.1%	-2.3	9659
Itpripl2	2.4%	0.1%	-2.3	9660
Ankhd1	6.3%	4.0%	-2.3	9661
LOC100911956	6.3%	4.0%	-2.3	9662
Smndc1	5.9%	3.6%	-2.3	9663
Rnf25	5.9%	3.6%	-2.3	9664
LOC100912309	5.9%	3.6%	-2.3	9665
Klf12	5.9%	3.6%	-2.3	9666
Rbm3	9.8%	7.5%	-2.3	9667
Nsmce1	5.5%	3.2%	-2.3	9668
Prpf6	5.5%	3.2%	-2.3	9669
Rybp	5.5%	3.2%	-2.3	9670
Capn7	5.5%	3.2%	-2.3	9671
Rab12	5.1%	2.8%	-2.3	9672
Pld3	5.1%	2.8%	-2.3	9673
Rbm38	5.1%	2.8%	-2.3	9674
LOC108348189	5.1%	2.8%	-2.3	9675
Chtf8	5.1%	2.8%	-2.3	9676
Zfp36	25.9%	23.6%	-2.3	9677
Plekhf2	4.7%	2.4%	-2.3	9678
Ublcp1	4.7%	2.4%	-2.3	9679
Oard1	4.7%	2.4%	-2.3	9680
Pias2	4.7%	2.4%	-2.3	9681
Fam45a	4.7%	2.4%	-2.3	9682
Nr0b2	12.9%	10.6%	-2.3	9683
Chd9	4.3%	2.0%	-2.3	9684
Ick	4.3%	2.0%	-2.3	9685
Kif13b	4.3%	2.0%	-2.3	9686
Paqr3	4.3%	2.0%	-2.3	9687
Tes	4.3%	2.0%	-2.3	9688
Atp9b	4.3%	2.0%	-2.3	9689
Tmem144	4.3%	2.0%	-2.3	9690
Rpusd3	8.2%	5.9%	-2.3	9691
Fam20b	3.9%	1.6%	-2.3	9692
Ifrd2	3.9%	1.6%	-2.3	9693
Slc30a7	3.9%	1.6%	-2.3	9694
LOC102548101	3.9%	1.6%	-2.3	9695
Rc3h2	3.9%	1.6%	-2.3	9696

Prrc2b	3.9%	1.6%	-2.3	9697
LOC102554600	3.9%	1.6%	-2.3	9698
LOC681383	3.9%	1.6%	-2.3	9699
Tmem184b	3.9%	1.6%	-2.3	9700
Fasn	32.9%	30.6%	-2.3	9701
Wdr24	3.5%	1.2%	-2.3	9702
Dcaf17	3.5%	1.2%	-2.3	9703
Trappc10	3.5%	1.2%	-2.3	9704
Mbd1	3.5%	1.2%	-2.3	9705
Inafm1	3.5%	1.2%	-2.3	9706
Mkrn2	3.5%	1.2%	-2.3	9707
Ttc14	3.5%	1.2%	-2.3	9708
Prkab2	3.5%	1.2%	-2.3	9709
Pex10	3.5%	1.2%	-2.3	9710
Rnf186	3.1%	0.8%	-2.3	9711
Pdp1	3.1%	0.8%	-2.3	9712
Wdr7	3.1%	0.8%	-2.3	9713
Trmt44	3.1%	0.8%	-2.3	9714
Ahcyl2	3.1%	0.8%	-2.3	9715
Mettl13	3.1%	0.8%	-2.3	9716
Zfp62	3.1%	0.8%	-2.3	9717
Usp42	3.1%	0.8%	-2.3	9718
Tbc1d25	3.1%	0.8%	-2.3	9719
Lmbr1	3.1%	0.8%	-2.3	9720
Sav1	3.1%	0.8%	-2.3	9721
Krt10	3.1%	0.8%	-2.3	9722
LOC102547949	3.1%	0.8%	-2.3	9723
Cog5	3.1%	0.8%	-2.3	9724
Rabgef1	7.1%	4.7%	-2.3	9725
Fam208b	2.7%	0.4%	-2.3	9726
LOC108348356	2.7%	0.4%	-2.3	9727
Ankrd28	2.7%	0.4%	-2.3	9728
Tecpr2	2.7%	0.4%	-2.3	9729
LOC102549842	2.7%	0.4%	-2.3	9730
Nkiras1	2.7%	0.4%	-2.3	9731
Fam210a	6.7%	4.3%	-2.4	9732
Epha2	6.7%	4.3%	-2.4	9733
Ankrd13a	6.7%	4.3%	-2.4	9734
Esr1	6.7%	4.3%	-2.4	9735
Nelfe	6.7%	4.3%	-2.4	9736
Mpv17l2	6.7%	4.3%	-2.4	9737
Lmo7	2.4%	0.0%	-2.4	9738
Sdr16c6	2.4%	0.0%	-2.4	9739
Rbm47	10.6%	8.2%	-2.4	9740
Pacsin2	6.3%	3.9%	-2.4	9741
Abcd1	5.9%	3.5%	-2.4	9742
Sat2	5.9%	3.5%	-2.4	9743
Nabp1	5.9%	3.5%	-2.4	9744
Adgrl2	5.9%	3.5%	-2.4	9745
Mboat7	5.5%	3.1%	-2.4	9746
Ddx39a	5.5%	3.1%	-2.4	9747
Rps4y2	5.5%	3.1%	-2.4	9748
LOC102547009	5.5%	3.1%	-2.4	9749
Polr3g	5.1%	2.7%	-2.4	9750
Mbtps1	5.1%	2.7%	-2.4	9751
Ruvbl2	5.1%	2.7%	-2.4	9752
Uri1	5.1%	2.7%	-2.4	9753
Vps26b	5.1%	2.7%	-2.4	9754
Sh3gl1	4.7%	2.3%	-2.4	9755
Acp5	4.7%	2.3%	-2.4	9756
Bicd2	4.7%	2.3%	-2.4	9757
Rdh14	4.7%	2.3%	-2.4	9758
Ndufaf7	4.7%	2.3%	-2.4	9759
Anxa4	8.6%	6.2%	-2.4	9760
LOC103690005	8.6%	6.2%	-2.4	9761
LOC100909433	50.2%	47.8%	-2.4	9762

LOC108348081	4.3%	1.9%	-2.4	9763
Smad5	4.3%	1.9%	-2.4	9764
Ankrd9	4.3%	1.9%	-2.4	9765
Fkbpl	4.3%	1.9%	-2.4	9766
Mtif2	4.3%	1.9%	-2.4	9767
Etnk1	4.3%	1.9%	-2.4	9768
Snx27	4.3%	1.9%	-2.4	9769
Gck	4.3%	1.9%	-2.4	9770
Crebl2	4.3%	1.9%	-2.4	9771
Ap1s1	8.2%	5.8%	-2.4	9772
Tk2	3.9%	1.5%	-2.4	9773
Trim36	3.9%	1.5%	-2.4	9774
Tradd	3.9%	1.5%	-2.4	9775
Polr1e	3.9%	1.5%	-2.4	9776
Rmi1	3.9%	1.5%	-2.4	9777
Chd7	3.9%	1.5%	-2.4	9778
Accs	3.9%	1.5%	-2.4	9779
LOC686087	3.9%	1.5%	-2.4	9780
Zmym2	3.9%	1.5%	-2.4	9781
Rprd1b	3.9%	1.5%	-2.4	9782
Isyna1	3.9%	1.5%	-2.4	9783
Mepce	3.9%	1.5%	-2.4	9784
Vps52	3.9%	1.5%	-2.4	9785
Zfp106	7.8%	5.4%	-2.4	9786
Alg2	3.5%	1.1%	-2.4	9787
Fam63b	3.5%	1.1%	-2.4	9788
Tc2n	3.5%	1.1%	-2.4	9789
Hsf1	3.5%	1.1%	-2.4	9790
Phactr4	3.5%	1.1%	-2.4	9791
Sms	3.5%	1.1%	-2.4	9792
Pds5b	3.5%	1.1%	-2.4	9793
Psg16	32.5%	30.1%	-2.4	9794
Kctd6	7.5%	5.0%	-2.4	9795
Hlcs	3.1%	0.7%	-2.4	9796
Zbtb22	3.1%	0.7%	-2.4	9797
Vps9d1	3.1%	0.7%	-2.4	9798
Cyb5r4	7.1%	4.6%	-2.4	9799
Trim65	2.7%	0.3%	-2.4	9800
Lama3	2.7%	0.3%	-2.4	9801
Daam1	6.7%	4.2%	-2.4	9802
Stat3	18.8%	16.4%	-2.4	9803
Amdhd2	6.3%	3.8%	-2.4	9804
Rc3h1	5.9%	3.4%	-2.4	9805
Purb	5.9%	3.4%	-2.4	9806
Taf15	5.9%	3.4%	-2.4	9807
Trim5	5.9%	3.4%	-2.4	9808
Celf2	5.9%	3.4%	-2.4	9809
Etl4	5.5%	3.0%	-2.5	9810
Dctpp1	5.5%	3.0%	-2.5	9811
Mef2d	5.5%	3.0%	-2.5	9812
Slc25a23	13.7%	11.3%	-2.5	9813
RGD1562140	13.7%	11.3%	-2.5	9814
Epb41	5.1%	2.6%	-2.5	9815
Exosc8	5.1%	2.6%	-2.5	9816
Gcc2	4.7%	2.2%	-2.5	9817
Mlxipl	4.7%	2.2%	-2.5	9818
Zcchc9	4.7%	2.2%	-2.5	9819
Jade1	4.7%	2.2%	-2.5	9820
Dhx36	8.6%	6.2%	-2.5	9821
Rcan2	8.6%	6.2%	-2.5	9822
Rbmxrtl	4.3%	1.8%	-2.5	9823
Slx1b	4.3%	1.8%	-2.5	9824
Zfp394	4.3%	1.8%	-2.5	9825
Tep1	4.3%	1.8%	-2.5	9826
Kat14	3.9%	1.4%	-2.5	9827
Cnot11	3.9%	1.4%	-2.5	9828

Lrig1	3.9%	1.4%	-2.5	9829
Wdr11	3.9%	1.4%	-2.5	9830
Npas2	3.9%	1.4%	-2.5	9831
Pgm3	3.9%	1.4%	-2.5	9832
Ttc27	3.9%	1.4%	-2.5	9833
Itgae	3.9%	1.4%	-2.5	9834
LOC108351588	3.9%	1.4%	-2.5	9835
Ccdc68	3.5%	1.0%	-2.5	9836
Vars2	3.5%	1.0%	-2.5	9837
Med14	3.5%	1.0%	-2.5	9838
LOC499331	3.5%	1.0%	-2.5	9839
Aktip	3.5%	1.0%	-2.5	9840
Dhx8	3.5%	1.0%	-2.5	9841
Zbtb4	3.5%	1.0%	-2.5	9842
Ogfod2	3.5%	1.0%	-2.5	9843
Mettl22	3.5%	1.0%	-2.5	9844
Xkr9	7.5%	5.0%	-2.5	9845
Ptpn3	7.5%	5.0%	-2.5	9846
Map7d1	7.5%	5.0%	-2.5	9847
Lsm11	3.1%	0.6%	-2.5	9848
Usf3	3.1%	0.6%	-2.5	9849
Ciz1	3.1%	0.6%	-2.5	9850
Zdhhc23	3.1%	0.6%	-2.5	9851
Kdm3b	3.1%	0.6%	-2.5	9852
Traf7	3.1%	0.6%	-2.5	9853
Tada2a	3.1%	0.6%	-2.5	9854
Ccpg1os	2.7%	0.2%	-2.5	9855
Zfp697	2.7%	0.2%	-2.5	9856
Flot1	6.7%	4.2%	-2.5	9857
Dym	6.3%	3.8%	-2.5	9858
Lrba	5.9%	3.4%	-2.5	9859
Zfp770	5.9%	3.4%	-2.5	9860
Trappc6b	5.9%	3.4%	-2.5	9861
Hnf1a	5.9%	3.4%	-2.5	9862
Rps6kb1	5.9%	3.4%	-2.5	9863
Plekha1	5.9%	3.4%	-2.5	9864
Acot2	14.1%	11.6%	-2.5	9865
Cobl	5.5%	3.0%	-2.5	9866
Kmt2e	5.5%	3.0%	-2.5	9867
Ciao1	5.5%	3.0%	-2.5	9868
Mapk1	5.5%	3.0%	-2.5	9869
Ccdc127	5.5%	3.0%	-2.5	9870
Fam127c	5.1%	2.6%	-2.5	9871
Hmgn3	5.1%	2.6%	-2.5	9872
Exosc1	5.1%	2.6%	-2.5	9873
Safb	5.1%	2.6%	-2.5	9874
Nisch	9.0%	6.5%	-2.5	9875
Pard6b	4.7%	2.2%	-2.5	9876
Ugt2a3	4.7%	2.2%	-2.5	9877
Akap9	4.7%	2.2%	-2.5	9878
Nktr	4.7%	2.2%	-2.5	9879
Slc12a4	4.7%	2.2%	-2.5	9880
Tle4	4.3%	1.8%	-2.6	9881
Sh3glb2	4.3%	1.8%	-2.6	9882
Iqcg	4.3%	1.8%	-2.6	9883
LOC498122	4.3%	1.8%	-2.6	9884
Arf2	4.3%	1.8%	-2.6	9885
RGD1311188	4.3%	1.8%	-2.6	9886
Hsd11b2	4.3%	1.8%	-2.6	9887
Smim11	4.3%	1.8%	-2.6	9888
Ppil3	3.9%	1.4%	-2.6	9889
Adprh	3.9%	1.4%	-2.6	9890
Htt	3.9%	1.4%	-2.6	9891
Tbk1	3.9%	1.4%	-2.6	9892
Hmgxb3	3.9%	1.4%	-2.6	9893
Trappc11	3.9%	1.4%	-2.6	9894

Obfc1	3.9%	1.4%	-2.6	9895
Tbrg1	7.8%	5.3%	-2.6	9896
Crtap	3.5%	1.0%	-2.6	9897
Bcl9l	3.5%	1.0%	-2.6	9898
Strip1	3.5%	1.0%	-2.6	9899
Fam98c	3.5%	1.0%	-2.6	9900
Pan2	3.5%	1.0%	-2.6	9901
Dennd1a	3.5%	1.0%	-2.6	9902
Cttnbp2nl	3.5%	1.0%	-2.6	9903
Cactin	3.5%	1.0%	-2.6	9904
Nf1x	3.5%	1.0%	-2.6	9905
U2af2	7.5%	4.9%	-2.6	9906
Abhd11	7.5%	4.9%	-2.6	9907
App	7.5%	4.9%	-2.6	9908
Gdi2	36.5%	33.9%	-2.6	9909
LOC102547059	3.1%	0.6%	-2.6	9910
Fuk	3.1%	0.6%	-2.6	9911
Piezo1	3.1%	0.6%	-2.6	9912
LOC102553008	3.1%	0.6%	-2.6	9913
Panx1	3.1%	0.6%	-2.6	9914
Adgrl1	3.1%	0.6%	-2.6	9915
Galnt4	3.1%	0.6%	-2.6	9916
Dynlt1	3.1%	0.6%	-2.6	9917
Rbsn	3.1%	0.6%	-2.6	9918
Adat3	3.1%	0.6%	-2.6	9919
Kbtbd7	3.1%	0.6%	-2.6	9920
LOC108348106	3.1%	0.6%	-2.6	9921
Scrib	3.1%	0.6%	-2.6	9922
Fam134c	7.1%	4.5%	-2.6	9923
Tsr3	7.1%	4.5%	-2.6	9924
Fam193b	2.7%	0.2%	-2.6	9925
Zfp64	2.7%	0.2%	-2.6	9926
Zfp746	2.7%	0.2%	-2.6	9927
Ppp1r3g	2.7%	0.2%	-2.6	9928
LOC102555480	11.0%	8.4%	-2.6	9929
Thoc3	6.3%	3.7%	-2.6	9930
Tmub1	6.3%	3.7%	-2.6	9931
Tmem263	6.3%	3.7%	-2.6	9932
Mtap	6.3%	3.7%	-2.6	9933
Fip1l1	6.3%	3.7%	-2.6	9934
LOC108349443	6.3%	3.7%	-2.6	9935
Slc44a1	5.9%	3.3%	-2.6	9936
Pop4	5.9%	3.3%	-2.6	9937
Mcat	5.9%	3.3%	-2.6	9938
Catsperd	18.0%	15.4%	-2.6	9939
Dennd1b	5.5%	2.9%	-2.6	9940
Gemin8	5.5%	2.9%	-2.6	9941
Slc25a44	5.5%	2.9%	-2.6	9942
Nudt2	9.4%	6.8%	-2.6	9943
Top3b	5.1%	2.5%	-2.6	9944
Rsbn1l	5.1%	2.5%	-2.6	9945
Prpf3	5.1%	2.5%	-2.6	9946
Mta1	5.1%	2.5%	-2.6	9947
Akap8	4.7%	2.1%	-2.6	9948
Slc30a4	4.7%	2.1%	-2.6	9949
Dnmbp	4.7%	2.1%	-2.6	9950
Slc25a37	4.7%	2.1%	-2.6	9951
Tnks1bp1	4.7%	2.1%	-2.6	9952
Lrp6	4.7%	2.1%	-2.6	9953
Secisbp2	4.7%	2.1%	-2.6	9954
Dxo	4.7%	2.1%	-2.6	9955
Mul1	4.7%	2.1%	-2.6	9956
Acsm5	21.2%	18.5%	-2.6	9957
Adck2	4.3%	1.7%	-2.6	9958
Lrrc47	4.3%	1.7%	-2.6	9959
Snrk	4.3%	1.7%	-2.6	9960

Nrgn	4.3%	1.7%	-2.6	9961
Ptgfrn	4.3%	1.7%	-2.6	9962
Bod1l1	4.3%	1.7%	-2.6	9963
Supt20h	4.3%	1.7%	-2.6	9964
Brf2	4.3%	1.7%	-2.6	9965
Foxj3	4.3%	1.7%	-2.6	9966
Itpkc	4.3%	1.7%	-2.6	9967
Ubr7	4.3%	1.7%	-2.6	9968
Rere	4.3%	1.7%	-2.6	9969
Sfmbt1	4.3%	1.7%	-2.6	9970
Fgb	100.0%	97.4%	-2.6	9971
Snap23	8.2%	5.6%	-2.6	9972
Nsmaf	3.9%	1.3%	-2.6	9973
Eif2b3	3.9%	1.3%	-2.6	9974
Wdfy2	3.9%	1.3%	-2.6	9975
Wdr55	3.9%	1.3%	-2.6	9976
Mbnl3	3.9%	1.3%	-2.6	9977
Kri1	3.9%	1.3%	-2.6	9978
Ddx10	3.9%	1.3%	-2.6	9979
Chchd5	7.8%	5.2%	-2.6	9980
Thoc1	3.5%	0.9%	-2.7	9981
LOC102551435	3.5%	0.9%	-2.7	9982
Terf1	3.5%	0.9%	-2.7	9983
Cwc22	3.5%	0.9%	-2.7	9984
Prpf39	3.5%	0.9%	-2.7	9985
Ipo13	3.5%	0.9%	-2.7	9986
Xpo5	3.5%	0.9%	-2.7	9987
RGD1564865	7.5%	4.8%	-2.7	9988
Klhl22	3.1%	0.5%	-2.7	9989
Scnm1	3.1%	0.5%	-2.7	9990
Zfp143	3.1%	0.5%	-2.7	9991
LOC299312	3.1%	0.5%	-2.7	9992
LOC102549496	3.1%	0.5%	-2.7	9993
LOC102553363	3.1%	0.5%	-2.7	9994
Klf13	3.1%	0.5%	-2.7	9995
Kdelc1	3.1%	0.5%	-2.7	9996
Pdcd4	11.4%	8.7%	-2.7	9997
Snx1	7.1%	4.4%	-2.7	9998
Sema6c	2.7%	0.1%	-2.7	9999
Nudt11	2.7%	0.1%	-2.7	10000
LOC103693792	11.0%	8.3%	-2.7	10001
Hyal1	6.7%	4.0%	-2.7	10002
Alg3	6.7%	4.0%	-2.7	10003
Tmem41b	14.9%	12.2%	-2.7	10004
Wars	6.3%	3.6%	-2.7	10005
C1d	10.2%	7.5%	-2.7	10006
Dusp10	5.9%	3.2%	-2.7	10007
Itsn2	5.9%	3.2%	-2.7	10008
Dhx30	5.9%	3.2%	-2.7	10009
Atic	5.9%	3.2%	-2.7	10010
LOC100911093	5.9%	3.2%	-2.7	10011
Gda	5.5%	2.8%	-2.7	10012
Fundc1	5.5%	2.8%	-2.7	10013
LOC100366044	9.4%	6.7%	-2.7	10014
Ube2l6	9.4%	6.7%	-2.7	10015
Cisd3	9.4%	6.7%	-2.7	10016
Smyd2	5.1%	2.4%	-2.7	10017
Rps6kb2	5.1%	2.4%	-2.7	10018
Pofut2	5.1%	2.4%	-2.7	10019
Smg1	5.1%	2.4%	-2.7	10020
Gpkow	5.1%	2.4%	-2.7	10021
Tpgs2	5.1%	2.4%	-2.7	10022
Tbc1d9b	5.1%	2.4%	-2.7	10023
Anapc7	5.1%	2.4%	-2.7	10024
Slc31a2	5.1%	2.4%	-2.7	10025
Mtor	4.7%	2.0%	-2.7	10026

Dyrk1a	4.7%	2.0%	-2.7	10027
Tlk2	4.7%	2.0%	-2.7	10028
Phkb	4.7%	2.0%	-2.7	10029
Chd1	4.7%	2.0%	-2.7	10030
Polr3h	4.7%	2.0%	-2.7	10031
Klhl2	4.7%	2.0%	-2.7	10032
Msantd4	4.7%	2.0%	-2.7	10033
Arhgap1	4.7%	2.0%	-2.7	10034
LOC103692531	4.7%	2.0%	-2.7	10035
Spry1	8.6%	5.9%	-2.7	10036
Fam214a	8.6%	5.9%	-2.7	10037
Nmnat1	4.3%	1.6%	-2.7	10038
Nudt1	4.3%	1.6%	-2.7	10039
Irak3	4.3%	1.6%	-2.7	10040
Golga3	4.3%	1.6%	-2.7	10041
Plxnb1	4.3%	1.6%	-2.7	10042
Cmpk2	4.3%	1.6%	-2.7	10043
Hsdl1	4.3%	1.6%	-2.7	10044
Gtf3c1	4.3%	1.6%	-2.7	10045
Fastkd5	4.3%	1.6%	-2.7	10046
Uros	4.3%	1.6%	-2.7	10047
Acvr2a	4.3%	1.6%	-2.7	10048
Dmtf1	3.9%	1.2%	-2.7	10049
Taf3	3.9%	1.2%	-2.7	10050
Pus10	3.9%	1.2%	-2.7	10051
Gopc	3.9%	1.2%	-2.7	10052
Cnot3	3.9%	1.2%	-2.7	10053
Zbtb2	3.9%	1.2%	-2.7	10054
Ube4b	3.9%	1.2%	-2.7	10055
Tpra1	3.9%	1.2%	-2.7	10056
RGD1561149	3.9%	1.2%	-2.7	10057
Txndc12	49.4%	46.7%	-2.7	10058
Npy5r	3.5%	0.8%	-2.7	10059
Zfp414	3.5%	0.8%	-2.7	10060
Pik3cb	3.5%	0.8%	-2.7	10061
Unkl	3.5%	0.8%	-2.7	10062
Lonrf3	3.5%	0.8%	-2.7	10063
Flywch2	3.5%	0.8%	-2.7	10064
Tmem87b	3.5%	0.8%	-2.7	10065
Fam168a	3.5%	0.8%	-2.7	10066
Sgms1	3.5%	0.8%	-2.7	10067
Snx15	3.5%	0.8%	-2.7	10068
Jmy	3.5%	0.8%	-2.7	10069
Ypel3	7.5%	4.7%	-2.7	10070
Rbm7	7.5%	4.7%	-2.7	10071
Ap4s1	7.5%	4.7%	-2.7	10072
Ogdh	7.5%	4.7%	-2.7	10073
Utp20	3.1%	0.4%	-2.7	10074
Gdnf	3.1%	0.4%	-2.7	10075
Glmn	3.1%	0.4%	-2.7	10076
Lpar3	3.1%	0.4%	-2.7	10077
Shoc2	3.1%	0.4%	-2.7	10078
Mettl26	7.1%	4.3%	-2.7	10079
LOC100360143	2.7%	0.0%	-2.7	10080
Zfp653	2.7%	0.0%	-2.7	10081
Cadm1	6.7%	3.9%	-2.7	10082
Rrn3	6.7%	3.9%	-2.7	10083
Rapgef4	6.3%	3.5%	-2.8	10084
Glb1	6.3%	3.5%	-2.8	10085
Stx12	6.3%	3.5%	-2.8	10086
Srrm2	10.2%	7.4%	-2.8	10087
Rhot1	5.9%	3.1%	-2.8	10088
Usp34	5.9%	3.1%	-2.8	10089
Setd2	5.9%	3.1%	-2.8	10090
Fam173b	5.9%	3.1%	-2.8	10091
Tifa	9.8%	7.0%	-2.8	10092

Actr8	5.5%	2.7%	-2.8	10093
Rpap3	5.5%	2.7%	-2.8	10094
RGD1309779	5.5%	2.7%	-2.8	10095
Fam96b	5.5%	2.7%	-2.8	10096
Nek7	9.4%	6.6%	-2.8	10097
Spata13	5.1%	2.3%	-2.8	10098
Clip1	5.1%	2.3%	-2.8	10099
Mllt10	5.1%	2.3%	-2.8	10100
LOC688637	5.1%	2.3%	-2.8	10101
Tmem9	5.1%	2.3%	-2.8	10102
Ube2q2	5.1%	2.3%	-2.8	10103
Paqr7	13.3%	10.6%	-2.8	10104
Ddx52	4.7%	1.9%	-2.8	10105
Efcab14	4.7%	1.9%	-2.8	10106
Adgrv1	4.7%	1.9%	-2.8	10107
Pcgf5	4.7%	1.9%	-2.8	10108
RGD1559896	4.7%	1.9%	-2.8	10109
Daglb	4.7%	1.9%	-2.8	10110
Mzt2b	4.7%	1.9%	-2.8	10111
Igfbp2	8.6%	5.8%	-2.8	10112
Tmed5	8.6%	5.8%	-2.8	10113
Anp32e	8.6%	5.8%	-2.8	10114
Fbxo11	4.3%	1.5%	-2.8	10115
Rad51d	4.3%	1.5%	-2.8	10116
Otud7b	4.3%	1.5%	-2.8	10117
Esrrg	4.3%	1.5%	-2.8	10118
Srpk2	4.3%	1.5%	-2.8	10119
ST7	4.3%	1.5%	-2.8	10120
Nup85	4.3%	1.5%	-2.8	10121
Alg1	4.3%	1.5%	-2.8	10122
Zbtb1	3.9%	1.1%	-2.8	10123
Prmt3	3.9%	1.1%	-2.8	10124
Crym	3.9%	1.1%	-2.8	10125
Tmem131	3.9%	1.1%	-2.8	10126
Ikzf5	3.9%	1.1%	-2.8	10127
Ppfia1	3.9%	1.1%	-2.8	10128
Oxld1	3.9%	1.1%	-2.8	10129
Arid1a	3.9%	1.1%	-2.8	10130
Vps41	7.8%	5.0%	-2.8	10131
Trub2	3.5%	0.7%	-2.8	10132
Shroom3	3.5%	0.7%	-2.8	10133
Ppm1l	3.5%	0.7%	-2.8	10134
Lrch3	3.5%	0.7%	-2.8	10135
Raver1	3.5%	0.7%	-2.8	10136
Fbxo8	3.5%	0.7%	-2.8	10137
Kng1	99.2%	96.4%	-2.8	10138
Gtf2b	7.5%	4.6%	-2.8	10139
Pir	7.5%	4.6%	-2.8	10140
Mier1	7.5%	4.6%	-2.8	10141
Nup98	7.5%	4.6%	-2.8	10142
Lbhd1	7.5%	4.6%	-2.8	10143
Wwc3	3.1%	0.3%	-2.8	10144
Gpatch3	3.1%	0.3%	-2.8	10145
Hemk1	3.1%	0.3%	-2.8	10146
Tmem17	3.1%	0.3%	-2.8	10147
LOC108348257	3.1%	0.3%	-2.8	10148
Tulp3	3.1%	0.3%	-2.8	10149
Ube3b	3.1%	0.3%	-2.8	10150
Frs2	3.1%	0.3%	-2.8	10151
Rps6kc1	3.1%	0.3%	-2.8	10152
Wdr1	11.4%	8.6%	-2.8	10153
Rab22a	7.1%	4.2%	-2.8	10154
Ubr5	7.1%	4.2%	-2.8	10155
Gdi1	15.3%	12.5%	-2.8	10156
LOC102550754	6.7%	3.8%	-2.8	10157
Tspo	6.7%	3.8%	-2.8	10158

RGD1560917	6.7%	3.8%	-2.8	10159
Pbrm1	6.3%	3.4%	-2.8	10160
Appbp2	6.3%	3.4%	-2.8	10161
Brd4	10.2%	7.4%	-2.8	10162
Gba	5.9%	3.0%	-2.8	10163
Atxn3	5.9%	3.0%	-2.8	10164
Nasp	5.9%	3.0%	-2.8	10165
Tmod3	5.9%	3.0%	-2.8	10166
Kdsr	5.9%	3.0%	-2.8	10167
Arl8a	5.9%	3.0%	-2.8	10168
Tmem41a	5.9%	3.0%	-2.8	10169
Pdss2	5.5%	2.6%	-2.9	10170
Mavs	5.5%	2.6%	-2.9	10171
Sugp1	5.5%	2.6%	-2.9	10172
Spg20	5.5%	2.6%	-2.9	10173
Rtn4rl2	5.1%	2.2%	-2.9	10174
Atg2b	5.1%	2.2%	-2.9	10175
Fam103a1	5.1%	2.2%	-2.9	10176
Ift22	21.6%	18.7%	-2.9	10177
Ero1a	9.0%	6.2%	-2.9	10178
Pole3	9.0%	6.2%	-2.9	10179
Snx21	4.7%	1.8%	-2.9	10180
LOC100911248	4.7%	1.8%	-2.9	10181
Vasn	4.7%	1.8%	-2.9	10182
Tmem87a	4.7%	1.8%	-2.9	10183
LOC108349594	8.6%	5.8%	-2.9	10184
Mfsd13a	4.3%	1.4%	-2.9	10185
Pcsk7	4.3%	1.4%	-2.9	10186
Mtf1	4.3%	1.4%	-2.9	10187
Inafm2	4.3%	1.4%	-2.9	10188
Slc15a4	4.3%	1.4%	-2.9	10189
Hnrnpab	25.1%	22.2%	-2.9	10190
Thpo	3.9%	1.0%	-2.9	10191
LOC500584	3.9%	1.0%	-2.9	10192
Bivm	3.9%	1.0%	-2.9	10193
Irx5	3.9%	1.0%	-2.9	10194
Hook3	3.9%	1.0%	-2.9	10195
Tacc2	3.9%	1.0%	-2.9	10196
Ints8	3.9%	1.0%	-2.9	10197
RGD1564093	12.2%	9.3%	-2.9	10198
Sec11c	7.8%	5.0%	-2.9	10199
Tdrp	7.8%	5.0%	-2.9	10200
Golim4	7.8%	5.0%	-2.9	10201
Hps1	3.5%	0.6%	-2.9	10202
Fam216a	3.5%	0.6%	-2.9	10203
LOC680200	3.5%	0.6%	-2.9	10204
Tusc3	3.5%	0.6%	-2.9	10205
Lyn	7.5%	4.6%	-2.9	10206
Magee1	3.1%	0.2%	-2.9	10207
RGD1565498	3.1%	0.2%	-2.9	10208
Eif2b4	6.7%	3.8%	-2.9	10209
Nudt7	6.3%	3.4%	-2.9	10210
Naa30	5.9%	3.0%	-2.9	10211
Asph	5.9%	3.0%	-2.9	10212
Hpf1	5.9%	3.0%	-2.9	10213
Mis12	9.8%	6.9%	-2.9	10214
R3hdm2	9.8%	6.9%	-2.9	10215
Ccdc59	5.5%	2.6%	-2.9	10216
Ddx50	5.5%	2.6%	-2.9	10217
Dguok	5.5%	2.6%	-2.9	10218
Mtfmt	5.5%	2.6%	-2.9	10219
B4galt1	5.5%	2.6%	-2.9	10220
Exoc3	5.5%	2.6%	-2.9	10221
Actb	34.5%	31.6%	-2.9	10222
Ttc33	5.1%	2.2%	-2.9	10223
Styx	5.1%	2.2%	-2.9	10224

Susd6	5.1%	2.2%	-2.9	10225
Kat2b	5.1%	2.2%	-2.9	10226
Zfp707	5.1%	2.2%	-2.9	10227
Xrcc6	5.1%	2.2%	-2.9	10228
Lsm3	5.1%	2.2%	-2.9	10229
St3gal3	5.1%	2.2%	-2.9	10230
Vma21	17.3%	14.3%	-2.9	10231
Svil	4.7%	1.8%	-2.9	10232
Ppp1r16a	4.7%	1.8%	-2.9	10233
Gemin7	4.7%	1.8%	-2.9	10234
Kank1	4.7%	1.8%	-2.9	10235
LOC102547925	4.7%	1.8%	-2.9	10236
Ehd4	4.7%	1.8%	-2.9	10237
Fbxl20	4.7%	1.8%	-2.9	10238
LOC102549712	4.7%	1.8%	-2.9	10239
Wdr75	4.7%	1.8%	-2.9	10240
Ppp2r5c	8.6%	5.7%	-3.0	10241
LOC102552369	8.6%	5.7%	-3.0	10242
Ccdc51	4.3%	1.4%	-3.0	10243
Prr3	4.3%	1.4%	-3.0	10244
Lipo1	4.3%	1.4%	-3.0	10245
Prpf4	4.3%	1.4%	-3.0	10246
Patl1	4.3%	1.4%	-3.0	10247
Oma1	4.3%	1.4%	-3.0	10248
Poglut1	4.3%	1.4%	-3.0	10249
Ovca2	12.5%	9.6%	-3.0	10250
Uqcc3	8.2%	5.3%	-3.0	10251
Armt1	3.9%	1.0%	-3.0	10252
Inpp5k	3.9%	1.0%	-3.0	10253
Mast4	3.9%	1.0%	-3.0	10254
Zzz3	3.9%	1.0%	-3.0	10255
Zfp574	3.9%	1.0%	-3.0	10256
Qser1	3.9%	1.0%	-3.0	10257
Strn4	3.9%	1.0%	-3.0	10258
Socs6	3.9%	1.0%	-3.0	10259
Nrbf2	7.8%	4.9%	-3.0	10260
Gcnt4	3.5%	0.6%	-3.0	10261
Wdr13	3.5%	0.6%	-3.0	10262
Mast2	3.5%	0.6%	-3.0	10263
Mib2	3.5%	0.6%	-3.0	10264
Lrig3	3.5%	0.6%	-3.0	10265
LOC108348101	3.5%	0.6%	-3.0	10266
Cad	3.5%	0.6%	-3.0	10267
Itga7	3.5%	0.6%	-3.0	10268
LOC102546688	3.5%	0.6%	-3.0	10269
Sox13	3.5%	0.6%	-3.0	10270
Zfp772	3.5%	0.6%	-3.0	10271
Ccnj	3.5%	0.6%	-3.0	10272
Nat10	3.5%	0.6%	-3.0	10273
Sgsm3	3.5%	0.6%	-3.0	10274
Epb41l4b	3.5%	0.6%	-3.0	10275
mrpl11	11.8%	8.8%	-3.0	10276
Slc9a3r2	7.5%	4.5%	-3.0	10277
Add1	7.5%	4.5%	-3.0	10278
Nceh1	3.1%	0.2%	-3.0	10279
Adcy1	3.1%	0.2%	-3.0	10280
Nfyb	7.1%	4.1%	-3.0	10281
Thtpa	6.7%	3.7%	-3.0	10282
LOC100910177	6.3%	3.3%	-3.0	10283
Msl1	6.3%	3.3%	-3.0	10284
Ppp5c	5.9%	2.9%	-3.0	10285
Ascc3	5.9%	2.9%	-3.0	10286
Acbd4	9.8%	6.8%	-3.0	10287
Tmem57	5.5%	2.5%	-3.0	10288
Ccdc184	5.5%	2.5%	-3.0	10289
Serpinb8	5.5%	2.5%	-3.0	10290

Kat7	5.5%	2.5%	-3.0	10291
Pawr	9.4%	6.4%	-3.0	10292
Adpgk	5.1%	2.1%	-3.0	10293
Bms1	5.1%	2.1%	-3.0	10294
Ngrn	5.1%	2.1%	-3.0	10295
Snx14	5.1%	2.1%	-3.0	10296
Zfp524	5.1%	2.1%	-3.0	10297
Aig1	5.1%	2.1%	-3.0	10298
Xpnpep1	9.0%	6.0%	-3.0	10299
Tmem185b	4.7%	1.7%	-3.0	10300
Insc	4.7%	1.7%	-3.0	10301
Rab3gap2	4.7%	1.7%	-3.0	10302
Rcc1l	4.7%	1.7%	-3.0	10303
Hyal2	4.7%	1.7%	-3.0	10304
Tshz1	4.7%	1.7%	-3.0	10305
Dolpp1	4.7%	1.7%	-3.0	10306
Pde4dip	4.7%	1.7%	-3.0	10307
Zbtb48	4.7%	1.7%	-3.0	10308
Tti1	4.7%	1.7%	-3.0	10309
Ilf2	4.7%	1.7%	-3.0	10310
Brd1	4.3%	1.3%	-3.0	10311
Prpf18	4.3%	1.3%	-3.0	10312
Rgl2	3.9%	0.9%	-3.0	10313
Ghdc	3.9%	0.9%	-3.0	10314
LOC100359574	3.9%	0.9%	-3.0	10315
Klhl18	3.9%	0.9%	-3.0	10316
Erc1	3.9%	0.9%	-3.0	10317
Borcs6	3.9%	0.9%	-3.0	10318
Lin54	3.9%	0.9%	-3.0	10319
Ralgapb	3.5%	0.5%	-3.0	10320
Cenpc	3.5%	0.5%	-3.0	10321
Tbc1d31	3.5%	0.5%	-3.0	10322
Trim2	3.5%	0.5%	-3.0	10323
LOC100362980	7.5%	4.4%	-3.1	10324
Bcar3	7.5%	4.4%	-3.1	10325
Zbtb7a	7.5%	4.4%	-3.1	10326
Nr1h4	11.4%	8.3%	-3.1	10327
Wbp4	7.1%	4.0%	-3.1	10328
Kbtbd2	7.1%	4.0%	-3.1	10329
Sap30bp	6.7%	3.6%	-3.1	10330
Slu7	6.7%	3.6%	-3.1	10331
Osbpl2	6.3%	3.2%	-3.1	10332
Htatip2	6.3%	3.2%	-3.1	10333
Dtd1	6.3%	3.2%	-3.1	10334
Rfng	6.3%	3.2%	-3.1	10335
Cstf2t	5.9%	2.8%	-3.1	10336
Flcn	5.9%	2.8%	-3.1	10337
Prpf4b	5.9%	2.8%	-3.1	10338
Eng	5.9%	2.8%	-3.1	10339
Fbxo18	5.9%	2.8%	-3.1	10340
Cxcl14	9.8%	6.7%	-3.1	10341
Axin1	5.5%	2.4%	-3.1	10342
St6galnac6	5.5%	2.4%	-3.1	10343
Trip6	5.5%	2.4%	-3.1	10344
LOC102552343	5.5%	2.4%	-3.1	10345
Cxcl2	5.5%	2.4%	-3.1	10346
Mtmr10	5.5%	2.4%	-3.1	10347
Nap1l4	9.4%	6.3%	-3.1	10348
Psd3	5.1%	2.0%	-3.1	10349
Zfp260	5.1%	2.0%	-3.1	10350
Zc3h13	5.1%	2.0%	-3.1	10351
Thap12	5.1%	2.0%	-3.1	10352
LOC102552988	5.1%	2.0%	-3.1	10353
Mapk8	5.1%	2.0%	-3.1	10354
Slc35b4	5.1%	2.0%	-3.1	10355
Cryzl1	5.1%	2.0%	-3.1	10356

Pmm1	5.1%	2.0%	-3.1	10357
Usp48	5.1%	2.0%	-3.1	10358
LOC102553099	5.1%	2.0%	-3.1	10359
Arhgef7	5.1%	2.0%	-3.1	10360
Stat5b	4.7%	1.6%	-3.1	10361
Slc22a5	4.7%	1.6%	-3.1	10362
Magi1	4.7%	1.6%	-3.1	10363
Tmcc1	4.7%	1.6%	-3.1	10364
Atg16l1	4.7%	1.6%	-3.1	10365
Zmiz1	4.7%	1.6%	-3.1	10366
Lrrc42	4.7%	1.6%	-3.1	10367
Atp6v1a	8.6%	5.5%	-3.1	10368
Nfe2l1	8.6%	5.5%	-3.1	10369
Hmgn2	16.9%	13.7%	-3.1	10370
Taf4b	4.3%	1.2%	-3.1	10371
Stx6	4.3%	1.2%	-3.1	10372
Trim25	4.3%	1.2%	-3.1	10373
Zfp238	4.3%	1.2%	-3.1	10374
Crlf2	4.3%	1.2%	-3.1	10375
Neurl2	4.3%	1.2%	-3.1	10376
LOC100912031	4.3%	1.2%	-3.1	10377
Med24	4.3%	1.2%	-3.1	10378
LOC100910424	4.3%	1.2%	-3.1	10379
Stag1	4.3%	1.2%	-3.1	10380
LOC100909949	4.3%	1.2%	-3.1	10381
Tbl1x	8.2%	5.1%	-3.1	10382
Mecp2	3.9%	0.8%	-3.1	10383
Ndufaf6	3.9%	0.8%	-3.1	10384
Lats2	3.9%	0.8%	-3.1	10385
LOC102556092	3.9%	0.8%	-3.1	10386
Atp7a	3.9%	0.8%	-3.1	10387
Nsun5	3.9%	0.8%	-3.1	10388
Zfp777	3.5%	0.4%	-3.1	10389
Arpc4	20.0%	16.9%	-3.1	10390
Meiob	3.1%	0.0%	-3.1	10391
Scoc	7.1%	3.9%	-3.1	10392
Eftud2	7.1%	3.9%	-3.1	10393
Ndufs1	7.1%	3.9%	-3.1	10394
Rab33b	7.1%	3.9%	-3.1	10395
S1pr2	6.7%	3.5%	-3.1	10396
Dgkd	6.7%	3.5%	-3.1	10397
LOC100362724	6.7%	3.5%	-3.1	10398
Hmga1	6.3%	3.1%	-3.2	10399
LOC108348298	6.3%	3.1%	-3.2	10400
Bcl2l13	6.3%	3.1%	-3.2	10401
Arhgap12	6.3%	3.1%	-3.2	10402
Mfsd5	5.9%	2.7%	-3.2	10403
Ccdc22	5.9%	2.7%	-3.2	10404
Tcaim	5.5%	2.3%	-3.2	10405
Csnk1a1	5.5%	2.3%	-3.2	10406
Cyp20a1	5.5%	2.3%	-3.2	10407
Washc2c	5.5%	2.3%	-3.2	10408
Nop2	5.5%	2.3%	-3.2	10409
Nacc2	5.5%	2.3%	-3.2	10410
Pop7	5.5%	2.3%	-3.2	10411
Smim7	22.0%	18.8%	-3.2	10412
Ptpn2	9.4%	6.2%	-3.2	10413
Txnl4b	5.1%	1.9%	-3.2	10414
Snrnp40	5.1%	1.9%	-3.2	10415
Zdhhc12	5.1%	1.9%	-3.2	10416
Kifap3	5.1%	1.9%	-3.2	10417
Tgds	4.7%	1.5%	-3.2	10418
Lcn12	4.7%	1.5%	-3.2	10419
Rbms1	4.7%	1.5%	-3.2	10420
Impact	4.7%	1.5%	-3.2	10421
Kansl1	4.7%	1.5%	-3.2	10422

RGD1308428	4.7%	1.5%	-3.2	10423
Naa20	8.6%	5.4%	-3.2	10424
Idh3a	8.6%	5.4%	-3.2	10425
Usp20	4.3%	1.1%	-3.2	10426
Tbc1d13	4.3%	1.1%	-3.2	10427
Morc3	4.3%	1.1%	-3.2	10428
Lcmt2	4.3%	1.1%	-3.2	10429
Ldlrap1	4.3%	1.1%	-3.2	10430
Tab2	4.3%	1.1%	-3.2	10431
Tex10	3.9%	0.7%	-3.2	10432
LOC100911548	3.9%	0.7%	-3.2	10433
Pld1	3.9%	0.7%	-3.2	10434
Dnajb5	3.9%	0.7%	-3.2	10435
Dtx2	3.9%	0.7%	-3.2	10436
Tnip2	3.9%	0.7%	-3.2	10437
Slc9a8	3.9%	0.7%	-3.2	10438
Ddx59	3.5%	0.3%	-3.2	10439
RGD1310951	3.5%	0.3%	-3.2	10440
Cds1	3.5%	0.3%	-3.2	10441
Rab1b	11.8%	8.6%	-3.2	10442
Acad8	11.8%	8.6%	-3.2	10443
Ring1	7.5%	4.2%	-3.2	10444
Zfp24	7.5%	4.2%	-3.2	10445
Itch	7.5%	4.2%	-3.2	10446
LOC103691375	15.7%	12.5%	-3.2	10447
Txnrd2	6.7%	3.4%	-3.2	10448
Tmem214	6.7%	3.4%	-3.2	10449
LOC108348260	14.9%	11.7%	-3.2	10450
Ppp1r15b	10.6%	7.4%	-3.2	10451
Hddc3	6.3%	3.0%	-3.2	10452
Eif4ebp2	6.3%	3.0%	-3.2	10453
Aqp11	6.3%	3.0%	-3.2	10454
Xiap	6.3%	3.0%	-3.2	10455
LOC103694381	6.3%	3.0%	-3.2	10456
Xpo7	5.9%	2.6%	-3.2	10457
Adar	5.9%	2.6%	-3.2	10458
Cmc2	5.9%	2.6%	-3.2	10459
Bcs1l	5.9%	2.6%	-3.2	10460
Ttc37	5.9%	2.6%	-3.2	10461
LOC103690067	5.9%	2.6%	-3.2	10462
Phf14	5.9%	2.6%	-3.2	10463
Ly49s7	5.9%	2.6%	-3.2	10464
Asmtl	14.1%	10.9%	-3.2	10465
Elavl1	9.8%	6.6%	-3.2	10466
Pla2g6	5.5%	2.2%	-3.3	10467
Frk	5.5%	2.2%	-3.3	10468
Tgfbr1	5.5%	2.2%	-3.3	10469
Arrdc1	5.5%	2.2%	-3.3	10470
Ankrd49	5.5%	2.2%	-3.3	10471
Prpsap2	5.5%	2.2%	-3.3	10472
Nmral1	5.5%	2.2%	-3.3	10473
Gcs1	5.5%	2.2%	-3.3	10474
Slc22a4	5.5%	2.2%	-3.3	10475
Ryk	5.5%	2.2%	-3.3	10476
Ist1	5.5%	2.2%	-3.3	10477
Anapc16	5.5%	2.2%	-3.3	10478
Cxxc1	5.1%	1.8%	-3.3	10479
Pik3c3	5.1%	1.8%	-3.3	10480
Xpnpep2	9.0%	5.8%	-3.3	10481
Vhl	4.7%	1.4%	-3.3	10482
Rhbdd1	4.7%	1.4%	-3.3	10483
Pias4	4.7%	1.4%	-3.3	10484
Nploc4	4.7%	1.4%	-3.3	10485
LOC102550297	4.7%	1.4%	-3.3	10486
Ermard	4.7%	1.4%	-3.3	10487
Cep83	4.7%	1.4%	-3.3	10488

Edrf1	4.7%	1.4%	-3.3	10489
Slc35e1	4.7%	1.4%	-3.3	10490
Rps6ka4	4.7%	1.4%	-3.3	10491
Spopl	4.7%	1.4%	-3.3	10492
Chd8	4.7%	1.4%	-3.3	10493
Sgk1	8.6%	5.4%	-3.3	10494
Mrpl36	8.6%	5.4%	-3.3	10495
Cep170	4.3%	1.0%	-3.3	10496
Nfkbil1	4.3%	1.0%	-3.3	10497
Setd6	4.3%	1.0%	-3.3	10498
Med23	4.3%	1.0%	-3.3	10499
Usp45	4.3%	1.0%	-3.3	10500
Rbm4b	4.3%	1.0%	-3.3	10501
Gzf1	4.3%	1.0%	-3.3	10502
Csf2ra	4.3%	1.0%	-3.3	10503
Fam89a	8.2%	5.0%	-3.3	10504
Cnnm4	3.9%	0.6%	-3.3	10505
Itpr1	3.9%	0.6%	-3.3	10506
Wdtc1	7.8%	4.6%	-3.3	10507
LOC103690101	3.5%	0.2%	-3.3	10508
Vamp3	11.8%	8.5%	-3.3	10509
Fam127b	7.1%	3.8%	-3.3	10510
Ifit2	11.0%	7.7%	-3.3	10511
C1r	98.0%	94.7%	-3.3	10512
Prkci	6.3%	3.0%	-3.3	10513
Aatf	6.3%	3.0%	-3.3	10514
Dnm1l	6.3%	3.0%	-3.3	10515
Nenf	6.3%	3.0%	-3.3	10516
RGD1565033	6.3%	3.0%	-3.3	10517
Nampt	22.7%	19.4%	-3.3	10518
Ppox	10.2%	6.9%	-3.3	10519
Tmx4	5.9%	2.6%	-3.3	10520
Nudt22	5.9%	2.6%	-3.3	10521
Clcn7	5.9%	2.6%	-3.3	10522
Dscr3	5.9%	2.6%	-3.3	10523
Nudt12	5.9%	2.6%	-3.3	10524
Sertad2	5.5%	2.2%	-3.3	10525
Sh2b1	5.5%	2.2%	-3.3	10526
Lyrm1	5.5%	2.2%	-3.3	10527
Kdm5b	5.5%	2.2%	-3.3	10528
Sptlc1	5.5%	2.2%	-3.3	10529
LOC102548740	5.5%	2.2%	-3.3	10530
Resp18	5.1%	1.8%	-3.3	10531
Fam58b	5.1%	1.8%	-3.3	10532
Igf2r	5.1%	1.8%	-3.3	10533
Dusp22	5.1%	1.8%	-3.3	10534
Thap11	5.1%	1.8%	-3.3	10535
Bmf	5.1%	1.8%	-3.3	10536
LOC100911615	9.0%	5.7%	-3.3	10537
Cuedc2	4.7%	1.4%	-3.3	10538
Plpp1	4.7%	1.4%	-3.3	10539
Crim1	4.7%	1.4%	-3.3	10540
Nhp2l1	4.7%	1.4%	-3.3	10541
Gart	4.7%	1.4%	-3.3	10542
NEWGENE_1565481	4.7%	1.4%	-3.3	10543
Strbp	8.6%	5.3%	-3.4	10544
Anxa7	4.3%	1.0%	-3.4	10545
Nt5c	4.3%	1.0%	-3.4	10546
Smurf1	4.3%	1.0%	-3.4	10547
Pex7	45.9%	42.5%	-3.4	10548
Slc1a6	3.9%	0.6%	-3.4	10549
Ankrd50	3.9%	0.6%	-3.4	10550
Dennd2c	3.9%	0.6%	-3.4	10551
Ykt6	7.1%	3.7%	-3.4	10552
Nostrin	7.1%	3.7%	-3.4	10553
Rnf213	7.1%	3.7%	-3.4	10554

Metap1	7.1%	3.7%	-3.4	10555
Larp1	15.3%	11.9%	-3.4	10556
Eefsec	6.7%	3.3%	-3.4	10557
LOC100911453	6.7%	3.3%	-3.4	10558
Exosc7	6.7%	3.3%	-3.4	10559
Fzd8	6.7%	3.3%	-3.4	10560
Wdr46	6.7%	3.3%	-3.4	10561
Vps36	6.3%	2.9%	-3.4	10562
Zcchc24	6.3%	2.9%	-3.4	10563
Rnf152	6.3%	2.9%	-3.4	10564
Wbp1	6.3%	2.9%	-3.4	10565
Hdac10	5.9%	2.5%	-3.4	10566
Zdhhc15	5.9%	2.5%	-3.4	10567
Leo1	5.9%	2.5%	-3.4	10568
Tmem56	5.9%	2.5%	-3.4	10569
Dhx29	5.9%	2.5%	-3.4	10570
Slc4a4	9.8%	6.4%	-3.4	10571
Wnk1	9.8%	6.4%	-3.4	10572
Wasf2	5.5%	2.1%	-3.4	10573
Ankrd24	5.5%	2.1%	-3.4	10574
Cdc26	5.5%	2.1%	-3.4	10575
Gpatch11	13.7%	10.3%	-3.4	10576
Paox	9.4%	6.0%	-3.4	10577
Hsd17b8	9.4%	6.0%	-3.4	10578
Mrs2	5.1%	1.7%	-3.4	10579
Smarcc1	5.1%	1.7%	-3.4	10580
Ajuba	5.1%	1.7%	-3.4	10581
Paxbp1	5.1%	1.7%	-3.4	10582
Fastkd2	5.1%	1.7%	-3.4	10583
Rif1	5.1%	1.7%	-3.4	10584
Ltv1	5.1%	1.7%	-3.4	10585
Fam174a	5.1%	1.7%	-3.4	10586
LOC100910506	5.1%	1.7%	-3.4	10587
Upp2	9.0%	5.6%	-3.4	10588
Bptf	4.7%	1.3%	-3.4	10589
LOC102554026	4.7%	1.3%	-3.4	10590
Rhot2	4.7%	1.3%	-3.4	10591
Pdcd11	4.7%	1.3%	-3.4	10592
Zeb1	4.7%	1.3%	-3.4	10593
Zgpat	4.7%	1.3%	-3.4	10594
Rundc1	4.7%	1.3%	-3.4	10595
Bmpr2	4.7%	1.3%	-3.4	10596
Rfxapl1	4.7%	1.3%	-3.4	10597
Ltn1	4.7%	1.3%	-3.4	10598
LOC102556004	12.9%	9.5%	-3.4	10599
Map3k4	4.3%	0.9%	-3.4	10600
Kif21a	4.3%	0.9%	-3.4	10601
Tnfsf12	4.3%	0.9%	-3.4	10602
Sprtn	4.3%	0.9%	-3.4	10603
Ncbp3	4.3%	0.9%	-3.4	10604
Rad17	8.2%	4.8%	-3.4	10605
Tpd52	8.2%	4.8%	-3.4	10606
Sf3b6	8.2%	4.8%	-3.4	10607
LOC100911353	3.9%	0.5%	-3.4	10608
Dhx37	3.9%	0.5%	-3.4	10609
B9d2	3.9%	0.5%	-3.4	10610
Hexb	7.8%	4.4%	-3.4	10611
Coq5	7.8%	4.4%	-3.4	10612
Gtf2f1	7.8%	4.4%	-3.4	10613
Rbbp6	7.5%	4.0%	-3.5	10614
Slc30a9	7.5%	4.0%	-3.5	10615
RGD1559909	7.1%	3.6%	-3.5	10616
Man2c1	7.1%	3.6%	-3.5	10617
Irs2	7.1%	3.6%	-3.5	10618
LOC100360619	15.3%	11.8%	-3.5	10619
Ttc19	6.7%	3.2%	-3.5	10620

Mrps5	6.7%	3.2%	-3.5	10621
Med15	6.7%	3.2%	-3.5	10622
Hipk1	6.7%	3.2%	-3.5	10623
Fam53c	6.3%	2.8%	-3.5	10624
Znfx1	6.3%	2.8%	-3.5	10625
Rbm18	6.3%	2.8%	-3.5	10626
RGD1304587	10.2%	6.7%	-3.5	10627
Riok1	10.2%	6.7%	-3.5	10628
NEWGENE_1310847	5.9%	2.4%	-3.5	10629
Tnrc6b	5.9%	2.4%	-3.5	10630
Elf2	5.9%	2.4%	-3.5	10631
LOC102548499	5.9%	2.4%	-3.5	10632
Arsb	5.9%	2.4%	-3.5	10633
Ap3b1	5.9%	2.4%	-3.5	10634
Prmt1	9.8%	6.3%	-3.5	10635
Map1lc3a	9.8%	6.3%	-3.5	10636
Nol11	5.5%	2.0%	-3.5	10637
Ostm1	5.5%	2.0%	-3.5	10638
Ttc7b	5.5%	2.0%	-3.5	10639
Zbtb20	5.5%	2.0%	-3.5	10640
Mpp5	13.7%	10.2%	-3.5	10641
Mrto4	17.6%	14.1%	-3.5	10642
Surf2	5.1%	1.6%	-3.5	10643
Ttc23	5.1%	1.6%	-3.5	10644
Slc8b1	5.1%	1.6%	-3.5	10645
Rnf215	5.1%	1.6%	-3.5	10646
Kctd10	5.1%	1.6%	-3.5	10647
Pgpep1	17.3%	13.7%	-3.5	10648
Cpsf2	4.7%	1.2%	-3.5	10649
Mab21l3	4.7%	1.2%	-3.5	10650
Pxn	4.7%	1.2%	-3.5	10651
Rbbp8	8.6%	5.1%	-3.5	10652
Fam50a	8.6%	5.1%	-3.5	10653
Metap1d	4.3%	0.8%	-3.5	10654
Trim11	4.3%	0.8%	-3.5	10655
Nkiras2	4.3%	0.8%	-3.5	10656
Meaf6	4.3%	0.8%	-3.5	10657
Fgg	100.0%	96.5%	-3.5	10658
Med4	8.2%	4.7%	-3.5	10659
Tmem126b	8.2%	4.7%	-3.5	10660
Necap1	8.2%	4.7%	-3.5	10661
Zfp672	3.9%	0.4%	-3.5	10662
Socs7	3.9%	0.4%	-3.5	10663
Gmpr2	7.8%	4.3%	-3.5	10664
Klc4	7.8%	4.3%	-3.5	10665
Tcta	7.8%	4.3%	-3.5	10666
Gpr107	7.8%	4.3%	-3.5	10667
Spata2L	7.5%	3.9%	-3.5	10668
Skap2	7.5%	3.9%	-3.5	10669
Pdp2	7.5%	3.9%	-3.5	10670
Ankrd11	7.5%	3.9%	-3.5	10671
Ptges2	7.1%	3.5%	-3.5	10672
Aph1a	11.0%	7.4%	-3.5	10673
Otud1	19.2%	15.7%	-3.5	10674
Znrd1	6.7%	3.1%	-3.5	10675
Pla2g15	6.7%	3.1%	-3.5	10676
Rnpep	6.7%	3.1%	-3.5	10677
Mphosph10	6.7%	3.1%	-3.5	10678
Gatad2a	6.7%	3.1%	-3.5	10679
Zdhhc5	6.7%	3.1%	-3.5	10680
Ing2	6.3%	2.7%	-3.6	10681
Gabpa	6.3%	2.7%	-3.6	10682
Cstf1	6.3%	2.7%	-3.6	10683
Ulk2	6.3%	2.7%	-3.6	10684
Hnrnpul1	6.3%	2.7%	-3.6	10685
Hnrnpm	14.5%	11.0%	-3.6	10686

Rab3ip	10.2%	6.6%	-3.6	10687
Map3k11	5.9%	2.3%	-3.6	10688
Tbc1d14	5.9%	2.3%	-3.6	10689
Chmp2b	5.9%	2.3%	-3.6	10690
Dhx32	5.9%	2.3%	-3.6	10691
Armc5	5.9%	2.3%	-3.6	10692
Tp53bp2	5.9%	2.3%	-3.6	10693
Card6	5.9%	2.3%	-3.6	10694
Cog8	9.8%	6.2%	-3.6	10695
Utrn	5.5%	1.9%	-3.6	10696
Syngr1	5.5%	1.9%	-3.6	10697
Dhrs13	5.5%	1.9%	-3.6	10698
Cdk5	5.5%	1.9%	-3.6	10699
Fzd4	9.4%	5.8%	-3.6	10700
Ccdc25	9.4%	5.8%	-3.6	10701
Phf20l1	5.1%	1.5%	-3.6	10702
Tmem143	5.1%	1.5%	-3.6	10703
Zfp180	5.1%	1.5%	-3.6	10704
Rbm5	5.1%	1.5%	-3.6	10705
Tmlhe	5.1%	1.5%	-3.6	10706
Pusl1	5.1%	1.5%	-3.6	10707
Rab5b	13.3%	9.8%	-3.6	10708
Saysd1	4.7%	1.1%	-3.6	10709
LOC100151767	4.7%	1.1%	-3.6	10710
Slc16a4	4.7%	1.1%	-3.6	10711
Cog3	4.7%	1.1%	-3.6	10712
Pwp1	4.7%	1.1%	-3.6	10713
Gimd1	4.7%	1.1%	-3.6	10714
Fam102a	4.7%	1.1%	-3.6	10715
Ptcd3	8.6%	5.0%	-3.6	10716
Ptafr	4.3%	0.7%	-3.6	10717
Tti2	4.3%	0.7%	-3.6	10718
Wdr37	4.3%	0.7%	-3.6	10719
LOC103690018	12.5%	9.0%	-3.6	10720
Ppp1r7	8.2%	4.6%	-3.6	10721
Ehhadh	8.2%	4.6%	-3.6	10722
Rad54l2	3.9%	0.3%	-3.6	10723
LOC102551991	3.9%	0.3%	-3.6	10724
Phtf2	3.9%	0.3%	-3.6	10725
Mtg2	7.8%	4.2%	-3.6	10726
Atf6b	7.5%	3.8%	-3.6	10727
Parp10	7.5%	3.8%	-3.6	10728
Map7	7.1%	3.4%	-3.6	10729
Iars2	7.1%	3.4%	-3.6	10730
Pigu	7.1%	3.4%	-3.6	10731
LOC102554630	7.1%	3.4%	-3.6	10732
LOC100911881	7.1%	3.4%	-3.6	10733
Eif2ak3	6.7%	3.0%	-3.6	10734
Yipf6	6.7%	3.0%	-3.6	10735
Samd4b	6.7%	3.0%	-3.6	10736
Nck1	6.7%	3.0%	-3.6	10737
Dlat	6.7%	3.0%	-3.6	10738
Btbd2	6.3%	2.6%	-3.6	10739
Spryd3	6.3%	2.6%	-3.6	10740
Nr2f6	14.5%	10.9%	-3.6	10741
Pank3	10.2%	6.6%	-3.6	10742
Ctu2	5.9%	2.2%	-3.6	10743
Spa17	5.9%	2.2%	-3.6	10744
Uvrag	5.9%	2.2%	-3.6	10745
RGD1561777	5.9%	2.2%	-3.6	10746
Mtif3	5.9%	2.2%	-3.6	10747
Swap70	5.9%	2.2%	-3.6	10748
Zfp11	5.9%	2.2%	-3.6	10749
Ngef	5.9%	2.2%	-3.6	10750
Ppp1r14a	14.1%	10.5%	-3.6	10751
Gns	9.8%	6.2%	-3.6	10752

Cdc40	5.5%	1.8%	-3.7	10753
Cadps2	5.5%	1.8%	-3.7	10754
Kmt5b	5.5%	1.8%	-3.7	10755
Dcaf5	5.5%	1.8%	-3.7	10756
Sowahc	5.1%	1.4%	-3.7	10757
Snx19	5.1%	1.4%	-3.7	10758
RGD1561481	5.1%	1.4%	-3.7	10759
LOC100909694	5.1%	1.4%	-3.7	10760
Mx2	17.3%	13.6%	-3.7	10761
Noc3l	4.7%	1.0%	-3.7	10762
Rb1	4.7%	1.0%	-3.7	10763
Gdap2	4.7%	1.0%	-3.7	10764
Chchd6	4.7%	1.0%	-3.7	10765
Tmem43	4.7%	1.0%	-3.7	10766
Miga2	4.3%	0.6%	-3.7	10767
Adamts1	4.3%	0.6%	-3.7	10768
Arhgef5	4.3%	0.6%	-3.7	10769
Zfyve16	4.3%	0.6%	-3.7	10770
Syne2	4.3%	0.6%	-3.7	10771
Fam220a	4.3%	0.6%	-3.7	10772
Ythdc1	12.2%	8.5%	-3.7	10773
Ano10	7.8%	4.2%	-3.7	10774
Rnf8	7.8%	4.2%	-3.7	10775
Acads	28.6%	24.9%	-3.7	10776
LOC100911599	7.5%	3.8%	-3.7	10777
Pfkfb1	7.5%	3.8%	-3.7	10778
Ubxn8	7.1%	3.4%	-3.7	10779
Nt5dc1	7.1%	3.4%	-3.7	10780
Actl6a	7.1%	3.4%	-3.7	10781
Dr1	7.1%	3.4%	-3.7	10782
Mapkapk5	6.7%	3.0%	-3.7	10783
Snrnp70	10.6%	6.9%	-3.7	10784
Ung	10.6%	6.9%	-3.7	10785
Ccdc91	6.3%	2.6%	-3.7	10786
RGD1307929	6.3%	2.6%	-3.7	10787
Sirt1	6.3%	2.6%	-3.7	10788
Fis1	18.4%	14.7%	-3.7	10789
Mbtd1	5.9%	2.2%	-3.7	10790
LOC691280	5.9%	2.2%	-3.7	10791
LOC100911534	5.9%	2.2%	-3.7	10792
Zhx1	5.9%	2.2%	-3.7	10793
Prkrir	5.9%	2.2%	-3.7	10794
Phip	5.5%	1.8%	-3.7	10795
LOC102555518	5.5%	1.8%	-3.7	10796
Rptor	5.5%	1.8%	-3.7	10797
Fig4	5.5%	1.8%	-3.7	10798
Dusp8	5.5%	1.8%	-3.7	10799
Wdr53	5.5%	1.8%	-3.7	10800
Ablim3	9.4%	5.7%	-3.7	10801
Acot5	9.4%	5.7%	-3.7	10802
Tbc1d5	5.1%	1.4%	-3.7	10803
LOC102547735	5.1%	1.4%	-3.7	10804
Ang2	5.1%	1.4%	-3.7	10805
Dctn4	5.1%	1.4%	-3.7	10806
Sbk1	5.1%	1.4%	-3.7	10807
Pald1	9.0%	5.3%	-3.7	10808
Phtf1	4.7%	1.0%	-3.7	10809
Dhodh	4.7%	1.0%	-3.7	10810
Msh6	4.7%	1.0%	-3.7	10811
Gpr155	4.7%	1.0%	-3.7	10812
Uckl1	4.7%	1.0%	-3.7	10813
Spred1	4.7%	1.0%	-3.7	10814
Zfyve1	4.7%	1.0%	-3.7	10815
Pkn1	4.7%	1.0%	-3.7	10816
Ccdc84	4.7%	1.0%	-3.7	10817
Pex26	4.7%	1.0%	-3.7	10818

Tmem104	4.3%	0.6%	-3.8	10819
Ddx6	8.2%	4.5%	-3.8	10820
Fbxl5	8.2%	4.5%	-3.8	10821
Igf2bp3	7.8%	4.1%	-3.8	10822
Dctn5	7.8%	4.1%	-3.8	10823
Pycrl	11.8%	8.0%	-3.8	10824
Csnk1g2	7.5%	3.7%	-3.8	10825
Klhl9	7.5%	3.7%	-3.8	10826
Cul4b	7.5%	3.7%	-3.8	10827
Acsf3	7.5%	3.7%	-3.8	10828
Trnt1	44.7%	40.9%	-3.8	10829
LOC100910850	19.6%	15.8%	-3.8	10830
Mad2l1bp	7.1%	3.3%	-3.8	10831
Pik3c2g	7.1%	3.3%	-3.8	10832
Siae	7.1%	3.3%	-3.8	10833
Zfand2b	7.1%	3.3%	-3.8	10834
Ksr2	6.7%	2.9%	-3.8	10835
Rae1	6.7%	2.9%	-3.8	10836
Habp4	6.7%	2.9%	-3.8	10837
LOC102554771	10.6%	6.8%	-3.8	10838
Casp6	6.3%	2.5%	-3.8	10839
LOC682812	6.3%	2.5%	-3.8	10840
Gla	5.9%	2.1%	-3.8	10841
Fbxl6	5.9%	2.1%	-3.8	10842
Tpcn1	5.9%	2.1%	-3.8	10843
Ccdc186	5.9%	2.1%	-3.8	10844
Nadsyn1	5.5%	1.7%	-3.8	10845
Zfp68	5.5%	1.7%	-3.8	10846
Lrrc28	5.5%	1.7%	-3.8	10847
Med16	5.5%	1.7%	-3.8	10848
Stxbp3	5.5%	1.7%	-3.8	10849
Trim33	5.5%	1.7%	-3.8	10850
Prmt5	5.5%	1.7%	-3.8	10851
Fam213b	13.7%	9.9%	-3.8	10852
Aldh1l2	9.4%	5.6%	-3.8	10853
Nsmce2	9.4%	5.6%	-3.8	10854
Setdb1	5.1%	1.3%	-3.8	10855
Clp1	5.1%	1.3%	-3.8	10856
Kdm5c	5.1%	1.3%	-3.8	10857
Wee1	5.1%	1.3%	-3.8	10858
Bzw2	5.1%	1.3%	-3.8	10859
Katna1	5.1%	1.3%	-3.8	10860
Map3k14	5.1%	1.3%	-3.8	10861
Asb6	5.1%	1.3%	-3.8	10862
Wsb1	13.3%	9.5%	-3.8	10863
Tp53inp1	9.0%	5.2%	-3.8	10864
Sae1	9.0%	5.2%	-3.8	10865
Slc25a46	9.0%	5.2%	-3.8	10866
Sdad1	4.7%	0.9%	-3.8	10867
Hist3h2a	4.7%	0.9%	-3.8	10868
Fbxw4	4.7%	0.9%	-3.8	10869
Plekha8	4.7%	0.9%	-3.8	10870
Nbas	4.7%	0.9%	-3.8	10871
Camsap2	4.7%	0.9%	-3.8	10872
LOC103690142	12.9%	9.1%	-3.8	10873
Bcl2l1	16.9%	13.0%	-3.8	10874
Kmt2b	4.3%	0.5%	-3.8	10875
LOC300249	58.4%	54.6%	-3.8	10876
Apoc1	100.0%	96.2%	-3.8	10877
Chrac1	8.2%	4.4%	-3.8	10878
Slc43a3	7.8%	4.0%	-3.8	10879
Ppil2	7.8%	4.0%	-3.8	10880
Tra2a	7.5%	3.6%	-3.9	10881
Acbd3	7.5%	3.6%	-3.9	10882
Dhx9	7.5%	3.6%	-3.9	10883
Chordc1	7.1%	3.2%	-3.9	10884

Pex1	7.1%	3.2%	-3.9	10885
Stk25	7.1%	3.2%	-3.9	10886
Fam35a	7.1%	3.2%	-3.9	10887
Mafk	11.0%	7.1%	-3.9	10888
Tmem63a	6.7%	2.8%	-3.9	10889
Chmp6	6.7%	2.8%	-3.9	10890
Tmx3	6.7%	2.8%	-3.9	10891
Chmp1a	10.6%	6.7%	-3.9	10892
Kdm3a	6.3%	2.4%	-3.9	10893
Zfp637	6.3%	2.4%	-3.9	10894
Ppih	6.3%	2.4%	-3.9	10895
LOC103690007	6.3%	2.4%	-3.9	10896
Mafl	6.3%	2.4%	-3.9	10897
Zap70	14.5%	10.6%	-3.9	10898
Srsf7	10.2%	6.3%	-3.9	10899
LOC691113	5.9%	2.0%	-3.9	10900
Adcy6	5.9%	2.0%	-3.9	10901
Vps13d	5.9%	2.0%	-3.9	10902
Kat8	5.9%	2.0%	-3.9	10903
Dhrs7b	9.8%	5.9%	-3.9	10904
Ogfod3	5.5%	1.6%	-3.9	10905
Sulf2	5.5%	1.6%	-3.9	10906
Rbm33	5.5%	1.6%	-3.9	10907
Flvcr2	5.5%	1.6%	-3.9	10908
Arel1	5.5%	1.6%	-3.9	10909
Fam98a	9.4%	5.5%	-3.9	10910
LOC103690116	5.1%	1.2%	-3.9	10911
Pprc1	5.1%	1.2%	-3.9	10912
Ptpn23	5.1%	1.2%	-3.9	10913
Snx25	4.7%	0.8%	-3.9	10914
Kat2a	4.7%	0.8%	-3.9	10915
Ankrd44	4.7%	0.8%	-3.9	10916
LOC102556047	8.6%	4.7%	-3.9	10917
Pstpip1	8.6%	4.7%	-3.9	10918
Nob1	12.5%	8.6%	-3.9	10919
Itih3	100.0%	96.1%	-3.9	10920
Rarres2	100.0%	96.1%	-3.9	10921
Cpne8	8.2%	4.3%	-3.9	10922
Prkar2a	8.2%	4.3%	-3.9	10923
LOC103690020	12.2%	8.2%	-3.9	10924
LOC102546393	12.2%	8.2%	-3.9	10925
Lamtor3	7.8%	3.9%	-3.9	10926
Rcc2	7.8%	3.9%	-3.9	10927
Timm17al1	7.8%	3.9%	-3.9	10928
Lpp	7.8%	3.9%	-3.9	10929
Bclaf1	11.8%	7.8%	-3.9	10930
Snap29	7.5%	3.5%	-3.9	10931
Smarcb1	7.5%	3.5%	-3.9	10932
Galk2	7.5%	3.5%	-3.9	10933
Snx18	7.5%	3.5%	-3.9	10934
Irf2	7.5%	3.5%	-3.9	10935
Zdhhc4	7.5%	3.5%	-3.9	10936
Lpar6	7.5%	3.5%	-3.9	10937
Smc6	11.0%	7.0%	-3.9	10938
Smarca2	6.7%	2.7%	-3.9	10939
Rpf1	6.7%	2.7%	-3.9	10940
Rtel1	6.7%	2.7%	-3.9	10941
Usp38	6.7%	2.7%	-3.9	10942
Klf11	6.7%	2.7%	-3.9	10943
Otud5	10.6%	6.6%	-4.0	10944
Srsf10	10.6%	6.6%	-4.0	10945
Fkbp3	10.6%	6.6%	-4.0	10946
Tmem203	6.3%	2.3%	-4.0	10947
LOC102547622	6.3%	2.3%	-4.0	10948
RGD1359508	6.3%	2.3%	-4.0	10949
Trim26	10.2%	6.2%	-4.0	10950

Crls1	18.4%	14.5%	-4.0	10951
Usp1	5.9%	1.9%	-4.0	10952
Aifm2	9.8%	5.8%	-4.0	10953
Ddx18	5.5%	1.5%	-4.0	10954
Wipi1	5.5%	1.5%	-4.0	10955
Hdac6	5.5%	1.5%	-4.0	10956
Osbpl11	5.5%	1.5%	-4.0	10957
Rbm34	5.5%	1.5%	-4.0	10958
Zcrb1	9.4%	5.4%	-4.0	10959
Nfic	9.4%	5.4%	-4.0	10960
B4galt3	5.1%	1.1%	-4.0	10961
Mtmr6	5.1%	1.1%	-4.0	10962
Snip1	5.1%	1.1%	-4.0	10963
Zfp217	5.1%	1.1%	-4.0	10964
RGD1310553	5.1%	1.1%	-4.0	10965
Ppm1g	9.0%	5.0%	-4.0	10966
Ttl	4.7%	0.7%	-4.0	10967
LOC108350495	4.7%	0.7%	-4.0	10968
LOC360231	4.7%	0.7%	-4.0	10969
Syne1	4.7%	0.7%	-4.0	10970
Ints3	4.7%	0.7%	-4.0	10971
LOC102552141	8.6%	4.6%	-4.0	10972
Pop1	4.3%	0.3%	-4.0	10973
Aldob	100.0%	96.0%	-4.0	10974
Ttc17	8.2%	4.2%	-4.0	10975
P2ry6	12.2%	8.2%	-4.0	10976
Cyb5b	11.8%	7.8%	-4.0	10977
LOC103694328	7.5%	3.4%	-4.0	10978
Arl2	7.5%	3.4%	-4.0	10979
Nxph4	7.5%	3.4%	-4.0	10980
Polr2a	7.5%	3.4%	-4.0	10981
Zfp259	15.7%	11.7%	-4.0	10982
Polr2k	11.4%	7.4%	-4.0	10983
Vwa8	7.1%	3.0%	-4.0	10984
Vac14	7.1%	3.0%	-4.0	10985
Arfrp1	6.7%	2.6%	-4.0	10986
Slc30a1	6.7%	2.6%	-4.0	10987
Vapb	10.6%	6.6%	-4.0	10988
Mon1b	6.3%	2.2%	-4.0	10989
Ctdp1	6.3%	2.2%	-4.0	10990
Rad23a	6.3%	2.2%	-4.0	10991
Emc3	10.2%	6.2%	-4.0	10992
Thumpd3	5.9%	1.8%	-4.0	10993
Pcm1	5.9%	1.8%	-4.0	10994
Ncoa1	5.9%	1.8%	-4.0	10995
Nol9	5.9%	1.8%	-4.0	10996
Alg8	5.9%	1.8%	-4.0	10997
Acd	5.9%	1.8%	-4.0	10998
Tmem209	5.9%	1.8%	-4.0	10999
Zbtb43	5.9%	1.8%	-4.0	11000
Eif3el1	18.0%	14.0%	-4.1	11001
Gmcl1	5.5%	1.4%	-4.1	11002
Tet2	5.5%	1.4%	-4.1	11003
Gtf2ird1	5.5%	1.4%	-4.1	11004
Akap11	5.5%	1.4%	-4.1	11005
Tma16	5.5%	1.4%	-4.1	11006
Zfp638	5.5%	1.4%	-4.1	11007
Ppp1r12a	9.4%	5.4%	-4.1	11008
Mief2	5.1%	1.0%	-4.1	11009
Htra2	9.0%	5.0%	-4.1	11010
Sec23a	9.0%	5.0%	-4.1	11011
Cenpv	9.0%	5.0%	-4.1	11012
Qtrt1	4.7%	0.6%	-4.1	11013
Itpr2	4.3%	0.2%	-4.1	11014
Suco	8.2%	4.2%	-4.1	11015
Ddx54	8.2%	4.2%	-4.1	11016

Gipc1	8.2%	4.2%	-4.1	11017
Pigt	8.2%	4.2%	-4.1	11018
Nqo1	7.8%	3.8%	-4.1	11019
Slc30a5	7.8%	3.8%	-4.1	11020
Tdp2	7.8%	3.8%	-4.1	11021
Pepd	7.8%	3.8%	-4.1	11022
Fam136a	11.8%	7.7%	-4.1	11023
Babam1	11.8%	7.7%	-4.1	11024
Mtfp1	7.5%	3.4%	-4.1	11025
Sos1	7.5%	3.4%	-4.1	11026
Cfap126	7.5%	3.4%	-4.1	11027
Mphosph6	7.5%	3.4%	-4.1	11028
Nhlrc3	7.5%	3.4%	-4.1	11029
Trappc3	11.4%	7.3%	-4.1	11030
LOC100909742	7.1%	3.0%	-4.1	11031
Ikbkb	7.1%	3.0%	-4.1	11032
Rbm45	6.7%	2.6%	-4.1	11033
Twsg1	6.7%	2.6%	-4.1	11034
LOC108348120	6.7%	2.6%	-4.1	11035
Plekhm1	6.7%	2.6%	-4.1	11036
Tbc1d2	6.7%	2.6%	-4.1	11037
N6amt1	6.7%	2.6%	-4.1	11038
Rufy3	6.7%	2.6%	-4.1	11039
Flnb	6.7%	2.6%	-4.1	11040
Zbtb42	6.3%	2.2%	-4.1	11041
Ripk4	6.3%	2.2%	-4.1	11042
Akap2	6.3%	2.2%	-4.1	11043
Pkp3	6.3%	2.2%	-4.1	11044
Chmp7	6.3%	2.2%	-4.1	11045
Hivep1	6.3%	2.2%	-4.1	11046
Klf15	6.3%	2.2%	-4.1	11047
LOC685273	10.2%	6.1%	-4.1	11048
Arfgef2	5.9%	1.8%	-4.1	11049
B4galt5	5.9%	1.8%	-4.1	11050
Osgep	5.9%	1.8%	-4.1	11051
Vamp4	9.8%	5.7%	-4.1	11052
Snap47	5.5%	1.4%	-4.1	11053
Alg12	5.5%	1.4%	-4.1	11054
Rara	5.5%	1.4%	-4.1	11055
Slc22a17	5.5%	1.4%	-4.1	11056
Rnf169	5.1%	1.0%	-4.1	11057
Ccdc112	5.1%	1.0%	-4.1	11058
Exosc10	5.1%	1.0%	-4.1	11059
Ywhae	54.9%	50.8%	-4.1	11060
Sik3	9.0%	4.9%	-4.1	11061
LOC100910130	9.0%	4.9%	-4.1	11062
RGD1597339	9.0%	4.9%	-4.1	11063
Mier3	4.7%	0.6%	-4.1	11064
Smg8	4.7%	0.6%	-4.1	11065
Hnrnph1	12.9%	8.8%	-4.1	11066
B2m	100.0%	95.8%	-4.2	11067
Bhmt	100.0%	95.8%	-4.2	11068
Mcrip2	8.2%	4.1%	-4.2	11069
Mrrf	8.2%	4.1%	-4.2	11070
Mfn2	8.2%	4.1%	-4.2	11071
Ppp2r3c	8.2%	4.1%	-4.2	11072
Kdelr2	29.0%	24.9%	-4.2	11073
Vezf1	7.8%	3.7%	-4.2	11074
Commd8	7.8%	3.7%	-4.2	11075
Trip11	7.8%	3.7%	-4.2	11076
Atxn2	7.5%	3.3%	-4.2	11077
RGD1562218	7.5%	3.3%	-4.2	11078
Cep19	7.5%	3.3%	-4.2	11079
Lrrc40	7.5%	3.3%	-4.2	11080
Atp6v1h	7.5%	3.3%	-4.2	11081
Coa6	15.7%	11.5%	-4.2	11082

Pfdn4	7.1%	2.9%	-4.2	11083
Kctd2	7.1%	2.9%	-4.2	11084
Rexo4	7.1%	2.9%	-4.2	11085
RGD1306215	7.1%	2.9%	-4.2	11086
Synpo	6.7%	2.5%	-4.2	11087
Nr4a1	6.7%	2.5%	-4.2	11088
Esco1	6.7%	2.5%	-4.2	11089
RGD1560065	6.7%	2.5%	-4.2	11090
Dgkq	6.7%	2.5%	-4.2	11091
Tdrd7	6.3%	2.1%	-4.2	11092
Ddx56	6.3%	2.1%	-4.2	11093
Riok2	6.3%	2.1%	-4.2	11094
Pdpk1	6.3%	2.1%	-4.2	11095
LOC691931	5.9%	1.7%	-4.2	11096
P4ha1	5.9%	1.7%	-4.2	11097
Polr3e	5.9%	1.7%	-4.2	11098
Gprin3	9.8%	5.6%	-4.2	11099
Oasl	9.8%	5.6%	-4.2	11100
Bmp4	5.5%	1.3%	-4.2	11101
Lnx2	5.5%	1.3%	-4.2	11102
Trmu	5.5%	1.3%	-4.2	11103
Ddx27	13.7%	9.5%	-4.2	11104
Skil	9.4%	5.2%	-4.2	11105
Tmem242	9.4%	5.2%	-4.2	11106
Adora2b	5.1%	0.9%	-4.2	11107
Spg11	5.1%	0.9%	-4.2	11108
Podxl	5.1%	0.9%	-4.2	11109
Sh2b3	4.7%	0.5%	-4.2	11110
LOC100912599	25.1%	20.9%	-4.2	11111
Klhl25	8.2%	4.0%	-4.2	11112
LOC102549131	16.5%	12.2%	-4.2	11113
Uba5	11.8%	7.5%	-4.3	11114
Nop14	7.5%	3.2%	-4.3	11115
Sgf29	7.5%	3.2%	-4.3	11116
Ino80c	7.5%	3.2%	-4.3	11117
Rnmt	7.1%	2.8%	-4.3	11118
Dnajc10	7.1%	2.8%	-4.3	11119
Tpp2	11.0%	6.7%	-4.3	11120
Cldnd1	11.0%	6.7%	-4.3	11121
Csk	11.0%	6.7%	-4.3	11122
Cpsf3	6.7%	2.4%	-4.3	11123
Pdzd11	6.7%	2.4%	-4.3	11124
Ash2l	6.7%	2.4%	-4.3	11125
Lig3	6.7%	2.4%	-4.3	11126
Golga4	10.6%	6.3%	-4.3	11127
Zmpste24	10.6%	6.3%	-4.3	11128
Dis3l	6.3%	2.0%	-4.3	11129
Coa7	6.3%	2.0%	-4.3	11130
Arpin	6.3%	2.0%	-4.3	11131
Szrd1	14.5%	10.2%	-4.3	11132
Aida	5.9%	1.6%	-4.3	11133
Sh3bp4	5.9%	1.6%	-4.3	11134
RGD1563365	9.8%	5.5%	-4.3	11135
Fgd4	5.5%	1.2%	-4.3	11136
Pacs2	5.5%	1.2%	-4.3	11137
Snrpd1	9.4%	5.1%	-4.3	11138
LOC100911576	17.6%	13.3%	-4.3	11139
Tjap1	5.1%	0.8%	-4.3	11140
Rpap2	5.1%	0.8%	-4.3	11141
LOC367858	9.0%	4.7%	-4.3	11142
Dcaf8	9.0%	4.7%	-4.3	11143
Cox17	21.2%	16.9%	-4.3	11144
RGD1308430	8.6%	4.3%	-4.3	11145
Onecut2	8.6%	4.3%	-4.3	11146
Tmem55b	8.6%	4.3%	-4.3	11147
Cystm1	8.6%	4.3%	-4.3	11148

Dnlz	8.6%	4.3%	-4.3	11149
LOC501416	12.5%	8.2%	-4.3	11150
Ccdc50	8.2%	3.9%	-4.3	11151
Zc3h7a	8.2%	3.9%	-4.3	11152
Utp18	8.2%	3.9%	-4.3	11153
Cs	7.8%	3.5%	-4.3	11154
Tpst2	7.8%	3.5%	-4.3	11155
Galnt2	7.8%	3.5%	-4.3	11156
Cct3	99.2%	94.9%	-4.3	11157
Arl2bp	7.5%	3.1%	-4.3	11158
Tjp3	7.5%	3.1%	-4.3	11159
LOC100365259	11.4%	7.0%	-4.3	11160
Polr3gl	11.4%	7.0%	-4.3	11161
Sun2	11.0%	6.6%	-4.3	11162
Fem1c	6.7%	2.3%	-4.3	11163
Mdm4	6.7%	2.3%	-4.3	11164
Bnip1	6.7%	2.3%	-4.3	11165
RGD1306941	6.7%	2.3%	-4.3	11166
LOC102547102	6.7%	2.3%	-4.3	11167
RT1-CE5	23.1%	18.8%	-4.4	11168
Dbr1	6.3%	1.9%	-4.4	11169
Cpsf7	6.3%	1.9%	-4.4	11170
Ccnc	10.2%	5.8%	-4.4	11171
Nudt3	10.2%	5.8%	-4.4	11172
Ranbp10	5.9%	1.5%	-4.4	11173
LOC102546589	5.9%	1.5%	-4.4	11174
Cbll1	5.9%	1.5%	-4.4	11175
Cdk20	5.9%	1.5%	-4.4	11176
Mon1a	5.9%	1.5%	-4.4	11177
Sp100	51.4%	47.0%	-4.4	11178
Smg5	5.5%	1.1%	-4.4	11179
Cherp	5.5%	1.1%	-4.4	11180
LOC689959	9.4%	5.0%	-4.4	11181
Dus1l	9.4%	5.0%	-4.4	11182
Mapkapk3	5.1%	0.7%	-4.4	11183
LOC691170	5.1%	0.7%	-4.4	11184
Mtpap	5.1%	0.7%	-4.4	11185
Abcb10	5.1%	0.7%	-4.4	11186
LOC100910827	9.0%	4.6%	-4.4	11187
Tmed4	9.0%	4.6%	-4.4	11188
LOC100910875	9.0%	4.6%	-4.4	11189
Utp11	9.0%	4.6%	-4.4	11190
Nprl2	8.6%	4.2%	-4.4	11191
P2rx1	58.4%	54.0%	-4.4	11192
Enc1	8.2%	3.8%	-4.4	11193
Rbm26	8.2%	3.8%	-4.4	11194
Fam175a	8.2%	3.8%	-4.4	11195
Oxnad1	8.2%	3.8%	-4.4	11196
Capn2	8.2%	3.8%	-4.4	11197
Rab35	7.8%	3.4%	-4.4	11198
Hgh1	7.8%	3.4%	-4.4	11199
Cry2	7.8%	3.4%	-4.4	11200
Ncaph2	7.8%	3.4%	-4.4	11201
Arhgap42	7.5%	3.0%	-4.4	11202
Smc3	7.5%	3.0%	-4.4	11203
Eif2b2	7.5%	3.0%	-4.4	11204
RGD1560795	11.4%	7.0%	-4.4	11205
Gsk3b	7.1%	2.6%	-4.4	11206
LOC102557408	15.3%	10.9%	-4.4	11207
Pdrg1	11.0%	6.6%	-4.4	11208
Bad	6.7%	2.2%	-4.4	11209
Wdr91	6.7%	2.2%	-4.4	11210
Tjp2	6.7%	2.2%	-4.4	11211
Plod3	6.7%	2.2%	-4.4	11212
Dnajc25	6.7%	2.2%	-4.4	11213
Taf11	6.3%	1.8%	-4.4	11214

Tywl	6.3%	1.8%	-4.4	11215
Gtpbp10	6.3%	1.8%	-4.4	11216
LOC103690159	6.3%	1.8%	-4.4	11217
Usp14	14.5%	10.1%	-4.4	11218
LOC100910882	22.7%	18.3%	-4.4	11219
Elk4	10.2%	5.8%	-4.4	11220
Tmem161b	5.9%	1.4%	-4.4	11221
Atpif1	18.0%	13.6%	-4.5	11222
Atp23	5.5%	1.0%	-4.5	11223
LOC102546764	5.5%	1.0%	-4.5	11224
Pls1	9.0%	4.6%	-4.5	11225
Fst	4.7%	0.2%	-4.5	11226
Timm17b	8.6%	4.2%	-4.5	11227
Nt5c3a	8.2%	3.8%	-4.5	11228
LOC100910678	20.4%	15.9%	-4.5	11229
Ube2e2	11.8%	7.3%	-4.5	11230
S1pr1	11.8%	7.3%	-4.5	11231
RGD1305089	7.5%	3.0%	-4.5	11232
Bysl	7.5%	3.0%	-4.5	11233
RGD1564854	7.5%	3.0%	-4.5	11234
Ugcg	7.1%	2.6%	-4.5	11235
Fbxw8	7.1%	2.6%	-4.5	11236
Wdr83	7.1%	2.6%	-4.5	11237
Zfp277	7.1%	2.6%	-4.5	11238
Ammecr1l	11.0%	6.5%	-4.5	11239
Abca3	6.7%	2.2%	-4.5	11240
Fastkd3	6.7%	2.2%	-4.5	11241
Nudt5	6.7%	2.2%	-4.5	11242
Slc27a1	6.7%	2.2%	-4.5	11243
Prkab1	14.9%	10.4%	-4.5	11244
Ctnnb1	23.1%	18.6%	-4.5	11245
Btbd9	6.3%	1.8%	-4.5	11246
Rel	5.9%	1.4%	-4.5	11247
Zfp513	5.9%	1.4%	-4.5	11248
Snrpb2	9.8%	5.3%	-4.5	11249
Bmp2	5.5%	1.0%	-4.5	11250
Tfdp1	5.5%	1.0%	-4.5	11251
Wdr4	5.5%	1.0%	-4.5	11252
Nufip1	5.5%	1.0%	-4.5	11253
Coro1b	13.3%	8.8%	-4.5	11254
Map3k8	9.0%	4.5%	-4.5	11255
Sf3b3	9.0%	4.5%	-4.5	11256
Ttyh2	12.9%	8.4%	-4.5	11257
Tsr2	8.6%	4.1%	-4.6	11258
Taf1d	8.6%	4.1%	-4.6	11259
Phkg2	8.6%	4.1%	-4.6	11260
Akt2	7.8%	3.3%	-4.6	11261
Upf3b	7.8%	3.3%	-4.6	11262
Tcerg1	7.8%	3.3%	-4.6	11263
Rsrc2	11.4%	6.8%	-4.6	11264
Tmem222	11.4%	6.8%	-4.6	11265
Rtf1	11.4%	6.8%	-4.6	11266
Kpna1	7.1%	2.5%	-4.6	11267
Nacc1	7.1%	2.5%	-4.6	11268
Rufy1	6.7%	2.1%	-4.6	11269
Arhgap8	10.6%	6.0%	-4.6	11270
Mrpl57	18.8%	14.2%	-4.6	11271
Tmem129	6.3%	1.7%	-4.6	11272
Zfp592	6.3%	1.7%	-4.6	11273
Emc2	10.2%	5.6%	-4.6	11274
Zcchc2	5.9%	1.3%	-4.6	11275
Bud13	5.9%	1.3%	-4.6	11276
Tbpl1	5.9%	1.3%	-4.6	11277
Tlk1	5.9%	1.3%	-4.6	11278
Pcnp	14.1%	9.5%	-4.6	11279
Mdp1	9.8%	5.2%	-4.6	11280

LOC103692169	9.8%	5.2%	-4.6	11281
Naa10	9.8%	5.2%	-4.6	11282
Cnnm3	5.5%	0.9%	-4.6	11283
Scn1b	5.5%	0.9%	-4.6	11284
Bag1	13.7%	9.1%	-4.6	11285
Srrd	9.4%	4.8%	-4.6	11286
Cul2	9.4%	4.8%	-4.6	11287
Gstt2	17.6%	13.0%	-4.6	11288
Azi2	8.6%	4.0%	-4.6	11289
Paf1	8.6%	4.0%	-4.6	11290
Slco1a1	8.2%	3.6%	-4.6	11291
Myo1d	7.8%	3.2%	-4.6	11292
Mprip	7.8%	3.2%	-4.6	11293
LOC100910207	7.8%	3.2%	-4.6	11294
Yme1l1	11.8%	7.1%	-4.7	11295
Rtcd1	7.5%	2.8%	-4.7	11296
Znhit2	7.5%	2.8%	-4.7	11297
Mef2a	7.5%	2.8%	-4.7	11298
Slc17a5	7.5%	2.8%	-4.7	11299
Cmc1	7.5%	2.8%	-4.7	11300
Arhgef19	11.4%	6.7%	-4.7	11301
Aamdc	7.1%	2.4%	-4.7	11302
Trmt2a	7.1%	2.4%	-4.7	11303
Prpf40a	11.0%	6.3%	-4.7	11304
Hnrnpll	6.7%	2.0%	-4.7	11305
Gapdh-ps1	18.8%	14.1%	-4.7	11306
Ube3a	6.3%	1.6%	-4.7	11307
Hars2	6.3%	1.6%	-4.7	11308
Epc2	6.3%	1.6%	-4.7	11309
Lamc1	6.3%	1.6%	-4.7	11310
Tax1bp3	14.5%	9.8%	-4.7	11311
Uqcc2	10.2%	5.5%	-4.7	11312
Snx4	10.2%	5.5%	-4.7	11313
Slc19a1	5.9%	1.2%	-4.7	11314
Utp23	5.9%	1.2%	-4.7	11315
Slc45a4	5.9%	1.2%	-4.7	11316
Ywhag	13.7%	9.0%	-4.7	11317
Klhdc10	9.4%	4.7%	-4.7	11318
Mphosph8	9.4%	4.7%	-4.7	11319
Nthl1	9.4%	4.7%	-4.7	11320
Eif4ebp3	9.0%	4.3%	-4.7	11321
Mrps31	8.6%	3.9%	-4.7	11322
Ngdn	8.6%	3.9%	-4.7	11323
LOC100361457	66.7%	62.0%	-4.7	11324
Fam69a	8.2%	3.5%	-4.7	11325
Ube2w	8.2%	3.5%	-4.7	11326
Dmap1	8.2%	3.5%	-4.7	11327
Poldip2	20.4%	15.7%	-4.7	11328
Nup50	7.8%	3.1%	-4.7	11329
Fchsd2	7.8%	3.1%	-4.7	11330
Tubg1	7.8%	3.1%	-4.7	11331
Ap3s1	7.8%	3.1%	-4.7	11332
Tmem109	7.8%	3.1%	-4.7	11333
Dnm2	7.8%	3.1%	-4.7	11334
Crcp	11.8%	7.0%	-4.7	11335
Fus	20.0%	15.3%	-4.7	11336
Trappc4	7.5%	2.7%	-4.7	11337
Dpy19l1	7.5%	2.7%	-4.7	11338
Atpaf2	7.5%	2.7%	-4.7	11339
Ccdc47	15.7%	11.0%	-4.7	11340
Med6	7.1%	2.3%	-4.7	11341
Rnaseh2a	15.3%	10.6%	-4.7	11342
Rnaseh2b	6.7%	1.9%	-4.7	11343
Josd1	6.7%	1.9%	-4.7	11344
Dapk2	6.7%	1.9%	-4.7	11345
Edem2	10.6%	5.8%	-4.8	11346

LOC681367	10.6%	5.8%	-4.8	11347
Ptdss2	6.3%	1.5%	-4.8	11348
Maff	14.5%	9.8%	-4.8	11349
Osbp	10.2%	5.4%	-4.8	11350
Exosc4	10.2%	5.4%	-4.8	11351
Timp3	18.4%	13.7%	-4.8	11352
Abca8	5.9%	1.1%	-4.8	11353
Zfp445	5.9%	1.1%	-4.8	11354
Mri1	9.8%	5.0%	-4.8	11355
Zbtb8os	9.8%	5.0%	-4.8	11356
Vti1b	9.8%	5.0%	-4.8	11357
RGD1306556	9.4%	4.6%	-4.8	11358
Cnot9	9.4%	4.6%	-4.8	11359
Cxcl10	80.0%	75.2%	-4.8	11360
Cpeb2	13.3%	8.6%	-4.8	11361
Lyar	8.6%	3.8%	-4.8	11362
Ankrd13c	8.6%	3.8%	-4.8	11363
Tle1	8.6%	3.8%	-4.8	11364
Ppie	8.6%	3.8%	-4.8	11365
Akirin1	8.6%	3.8%	-4.8	11366
Foxa2	8.6%	3.8%	-4.8	11367
Nelfcd	8.2%	3.4%	-4.8	11368
Med25	8.2%	3.4%	-4.8	11369
Mettl5	7.8%	3.0%	-4.8	11370
Mrpl47	7.8%	3.0%	-4.8	11371
Flot2	11.8%	7.0%	-4.8	11372
Tom1l1	7.5%	2.6%	-4.8	11373
Lmf1	7.5%	2.6%	-4.8	11374
LOC103692719	7.5%	2.6%	-4.8	11375
Ino80e	7.5%	2.6%	-4.8	11376
Nr1d1	11.4%	6.6%	-4.8	11377
Gpn1	7.1%	2.2%	-4.8	11378
Sumf2	7.1%	2.2%	-4.8	11379
Letm1	7.1%	2.2%	-4.8	11380
Mppe1	6.7%	1.8%	-4.8	11381
Pqbp1	10.6%	5.8%	-4.8	11382
Atp6v1c1	10.6%	5.8%	-4.8	11383
Zfp496	6.3%	1.4%	-4.8	11384
Phldb2	6.3%	1.4%	-4.8	11385
Zdhhc3	6.3%	1.4%	-4.8	11386
Zbtb17	6.3%	1.4%	-4.8	11387
Nle1	6.3%	1.4%	-4.8	11388
Odf3b	6.3%	1.4%	-4.8	11389
Npepo	5.9%	1.0%	-4.8	11390
Zfp869	5.9%	1.0%	-4.8	11391
LOC102555869	5.9%	1.0%	-4.8	11392
Timmdc1	9.8%	5.0%	-4.8	11393
Cdk6	5.5%	0.6%	-4.9	11394
Col4a3bp	9.4%	4.6%	-4.9	11395
Cnot6	9.4%	4.6%	-4.9	11396
Ggh	9.0%	4.2%	-4.9	11397
Zfp422	9.0%	4.2%	-4.9	11398
RGD1309821	8.6%	3.8%	-4.9	11399
Tapt1	8.6%	3.8%	-4.9	11400
Zfp295	8.6%	3.8%	-4.9	11401
Scamp3	12.5%	7.7%	-4.9	11402
Fnip1	8.2%	3.4%	-4.9	11403
Acbd6	8.2%	3.4%	-4.9	11404
Bid	16.5%	11.6%	-4.9	11405
Hdac5	7.8%	3.0%	-4.9	11406
Psmg4	11.8%	6.9%	-4.9	11407
Dync1h1	7.5%	2.6%	-4.9	11408
Kat5	7.5%	2.6%	-4.9	11409
Parp9	15.7%	10.8%	-4.9	11410
Tmx1	11.4%	6.5%	-4.9	11411
Rab14	19.6%	14.7%	-4.9	11412

Slc4a2	7.1%	2.2%	-4.9	11413
Mon2	7.1%	2.2%	-4.9	11414
Sf3a1	7.1%	2.2%	-4.9	11415
Pip4k2c	7.1%	2.2%	-4.9	11416
Bbc3	11.0%	6.1%	-4.9	11417
Gpr108	11.0%	6.1%	-4.9	11418
Atoh8	6.7%	1.8%	-4.9	11419
Pi4ka	6.7%	1.8%	-4.9	11420
Il17rc	6.7%	1.8%	-4.9	11421
Hlf	6.3%	1.4%	-4.9	11422
Uba6	6.3%	1.4%	-4.9	11423
Atp6v1d	14.5%	9.6%	-4.9	11424
Atpaf1	10.2%	5.3%	-4.9	11425
Lamtor5	10.2%	5.3%	-4.9	11426
Ctnnd1	10.2%	5.3%	-4.9	11427
Naaladl2	5.9%	1.0%	-4.9	11428
Atl3	14.1%	9.2%	-4.9	11429
Hp1bp3	9.8%	4.9%	-4.9	11430
Hace1	5.5%	0.6%	-4.9	11431
Ilkap	9.4%	4.5%	-4.9	11432
Tmem261	9.4%	4.5%	-4.9	11433
Zcchc17	9.4%	4.5%	-4.9	11434
Dusp11	9.4%	4.5%	-4.9	11435
Ensa	9.4%	4.5%	-4.9	11436
Sorbs1	9.0%	4.1%	-4.9	11437
Eef1e1	9.0%	4.1%	-4.9	11438
LOC100910717	8.6%	3.7%	-5.0	11439
Slc13a3	12.5%	7.6%	-5.0	11440
Asnsd1	8.2%	3.3%	-5.0	11441
LOC108353616	8.2%	3.3%	-5.0	11442
Syncrip	24.7%	19.7%	-5.0	11443
Cyb561d2	7.8%	2.9%	-5.0	11444
Pex2	7.8%	2.9%	-5.0	11445
Prmt9	7.8%	2.9%	-5.0	11446
Ppp6r1	7.8%	2.9%	-5.0	11447
Golt1a	7.8%	2.9%	-5.0	11448
Crebrf	7.8%	2.9%	-5.0	11449
Fam20a	7.8%	2.9%	-5.0	11450
Dhps	7.5%	2.5%	-5.0	11451
Med20	7.5%	2.5%	-5.0	11452
Mtrr	7.1%	2.1%	-5.0	11453
Med8	7.1%	2.1%	-5.0	11454
Zak	7.1%	2.1%	-5.0	11455
Sbf1	7.1%	2.1%	-5.0	11456
Cdc42se2	7.1%	2.1%	-5.0	11457
Bcl7c	11.0%	6.0%	-5.0	11458
Dcaf13	11.0%	6.0%	-5.0	11459
Llph	11.0%	6.0%	-5.0	11460
Pde8a	6.7%	1.7%	-5.0	11461
Cyld-ps1	6.7%	1.7%	-5.0	11462
Slc30a6	6.7%	1.7%	-5.0	11463
Ppp4r1	6.7%	1.7%	-5.0	11464
Mterf1	6.7%	1.7%	-5.0	11465
RGD1311899	6.3%	1.3%	-5.0	11466
Nepro	6.3%	1.3%	-5.0	11467
RGD1564379	6.3%	1.3%	-5.0	11468
Samd8	6.3%	1.3%	-5.0	11469
LOC102551352	6.3%	1.3%	-5.0	11470
Fbxl14	6.3%	1.3%	-5.0	11471
Nudcd2	10.2%	5.2%	-5.0	11472
Ipo11	5.9%	0.9%	-5.0	11473
Zswim8	5.9%	0.9%	-5.0	11474
Ctbp1	9.4%	4.4%	-5.0	11475
LOC100911313	9.4%	4.4%	-5.0	11476
Gnai2	13.3%	8.3%	-5.0	11477
Eif2b5	9.0%	4.0%	-5.0	11478

Sdhaf1	9.0%	4.0%	-5.0	11479
Papd5	9.0%	4.0%	-5.0	11480
Ccndbp1	8.6%	3.6%	-5.0	11481
Huwe1	8.6%	3.6%	-5.0	11482
Thoc5	8.2%	3.2%	-5.0	11483
Hook1	8.2%	3.2%	-5.0	11484
Rock2	8.2%	3.2%	-5.0	11485
Csnk1g3	8.2%	3.2%	-5.0	11486
Ap1b1	8.2%	3.2%	-5.0	11487
LOC683897	7.8%	2.8%	-5.0	11488
Smc1a	11.8%	6.7%	-5.1	11489
Dab2ip	7.5%	2.4%	-5.1	11490
Pck2	7.5%	2.4%	-5.1	11491
Ptprk	11.4%	6.3%	-5.1	11492
Nt5c2	11.4%	6.3%	-5.1	11493
Wrap53	7.1%	2.0%	-5.1	11494
Yars2	7.1%	2.0%	-5.1	11495
Rai14	7.1%	2.0%	-5.1	11496
Xab2	7.1%	2.0%	-5.1	11497
Pcdh18	7.1%	2.0%	-5.1	11498
Papd7	7.1%	2.0%	-5.1	11499
Abt1	7.1%	2.0%	-5.1	11500
Vcl	7.1%	2.0%	-5.1	11501
Mfsd8	7.1%	2.0%	-5.1	11502
Xkr8	6.7%	1.6%	-5.1	11503
Cog1	6.7%	1.6%	-5.1	11504
Ctps1	6.7%	1.6%	-5.1	11505
Vta1	10.6%	5.5%	-5.1	11506
Spata2	5.9%	0.8%	-5.1	11507
Fkbp5	5.9%	0.8%	-5.1	11508
Rtn4ip1	14.1%	9.0%	-5.1	11509
Arf3	9.8%	4.7%	-5.1	11510
Gtf3c4	5.5%	0.4%	-5.1	11511
RGD1311703	13.7%	8.6%	-5.1	11512
Mecr	9.4%	4.3%	-5.1	11513
Jmjd8	9.4%	4.3%	-5.1	11514
Cd151	12.9%	7.8%	-5.1	11515
RGD1559600	8.6%	3.5%	-5.1	11516
Rwdd2b	8.6%	3.5%	-5.1	11517
Polr2f	12.5%	7.4%	-5.1	11518
Apoa1	100.0%	94.9%	-5.1	11519
Gpx1	100.0%	94.9%	-5.1	11520
Sp1	8.2%	3.1%	-5.1	11521
Wdr74	8.2%	3.1%	-5.1	11522
Prrc2a	8.2%	3.1%	-5.1	11523
Polr3k	8.2%	3.1%	-5.1	11524
Letmd1	8.2%	3.1%	-5.1	11525
Naa25	7.8%	2.7%	-5.1	11526
Dennd4a	7.8%	2.7%	-5.1	11527
Cdk7	7.8%	2.7%	-5.1	11528
Hmgb1	24.3%	19.2%	-5.1	11529
Mapk3	11.8%	6.6%	-5.1	11530
Lsm1	7.5%	2.3%	-5.1	11531
Kdm2a	7.5%	2.3%	-5.1	11532
Sesn1	7.1%	1.9%	-5.1	11533
Kidins220	7.1%	1.9%	-5.1	11534
Dnajc4	7.1%	1.9%	-5.1	11535
LOC100911851	7.1%	1.9%	-5.1	11536
RGD1566099	11.0%	5.8%	-5.1	11537
Gemin6	11.0%	5.8%	-5.1	11538
Sppl3	6.7%	1.5%	-5.1	11539
Rpp40	6.7%	1.5%	-5.1	11540
Gorasp1	6.7%	1.5%	-5.1	11541
Vps11	6.7%	1.5%	-5.1	11542
Ubald1	10.6%	5.4%	-5.2	11543
Tmem179b	10.6%	5.4%	-5.2	11544

Arfip2	6.3%	1.1%	-5.2	11545
LOC103690016	6.3%	1.1%	-5.2	11546
Pja1	10.2%	5.0%	-5.2	11547
Agtr1a	14.1%	9.0%	-5.2	11548
Abcf2	9.8%	4.6%	-5.2	11549
Icmt	9.8%	4.6%	-5.2	11550
LOC100911730	9.8%	4.6%	-5.2	11551
Elob	34.5%	29.3%	-5.2	11552
Gak	9.4%	4.2%	-5.2	11553
Polg2	9.4%	4.2%	-5.2	11554
Rab18	13.3%	8.2%	-5.2	11555
Tmem218	9.0%	3.8%	-5.2	11556
Anapc4	9.0%	3.8%	-5.2	11557
Rtn4	42.0%	36.8%	-5.2	11558
Pde4b	8.6%	3.4%	-5.2	11559
LOC102551606	8.6%	3.4%	-5.2	11560
Esf1	8.6%	3.4%	-5.2	11561
Thyn1	100.0%	94.8%	-5.2	11562
Leprot	8.2%	3.0%	-5.2	11563
Scrn3	8.2%	3.0%	-5.2	11564
RGD1563888	12.2%	7.0%	-5.2	11565
Med10	7.8%	2.6%	-5.2	11566
RT1-S2	16.1%	10.9%	-5.2	11567
Snrpc	11.8%	6.6%	-5.2	11568
Atg2a	7.5%	2.2%	-5.2	11569
Cdkn2aip	7.5%	2.2%	-5.2	11570
Calm3	11.4%	6.2%	-5.2	11571
LOC100910528	11.4%	6.2%	-5.2	11572
Slc5a6	7.1%	1.8%	-5.2	11573
Rhbdd3	7.1%	1.8%	-5.2	11574
Dock11	7.1%	1.8%	-5.2	11575
Dnajc7	11.0%	5.8%	-5.2	11576
Srprb	10.6%	5.4%	-5.2	11577
Gsdmd	10.6%	5.4%	-5.2	11578
Tut1	6.3%	1.0%	-5.2	11579
Polr2b	10.2%	5.0%	-5.2	11580
Lmbr1l	5.9%	0.6%	-5.2	11581
Dmxl2	5.9%	0.6%	-5.2	11582
Spsb1	5.9%	0.6%	-5.2	11583
Cyp7b1	5.5%	0.2%	-5.3	11584
LOC102556337	13.7%	8.5%	-5.3	11585
Arhgap29	9.4%	4.2%	-5.3	11586
LOC680875	9.0%	3.8%	-5.3	11587
Hspbp1	8.2%	3.0%	-5.3	11588
LOC102553046	7.8%	2.6%	-5.3	11589
Ift57	7.8%	2.6%	-5.3	11590
Ccnk	7.8%	2.6%	-5.3	11591
Umps	16.1%	10.8%	-5.3	11592
Ppig	11.8%	6.5%	-5.3	11593
Vangl1	7.5%	2.2%	-5.3	11594
Npm3	7.5%	2.2%	-5.3	11595
Scfd2	7.5%	2.2%	-5.3	11596
Osbpl1a	11.4%	6.1%	-5.3	11597
LOC108348093	11.4%	6.1%	-5.3	11598
Smim14	98.8%	93.5%	-5.3	11599
Arhgap17	7.1%	1.8%	-5.3	11600
Rnf44	7.1%	1.8%	-5.3	11601
LOC108349010	11.0%	5.7%	-5.3	11602
Tceb2	31.8%	26.5%	-5.3	11603
Nrdc	10.6%	5.3%	-5.3	11604
Bax	10.6%	5.3%	-5.3	11605
St7l	6.3%	1.0%	-5.3	11606
Uqcc1	10.2%	4.9%	-5.3	11607
Ncbp2	9.8%	4.5%	-5.3	11608
Ndufb1	13.7%	8.4%	-5.3	11609
Car14	9.4%	4.1%	-5.3	11610

Acbd5	13.3%	8.0%	-5.3	11611
Sptbn1	13.3%	8.0%	-5.3	11612
Nufip2	9.0%	3.7%	-5.3	11613
Asb8	9.0%	3.7%	-5.3	11614
Smap1	9.0%	3.7%	-5.3	11615
Csrp2	9.0%	3.7%	-5.3	11616
Fez2	9.0%	3.7%	-5.3	11617
Pih1d3	8.6%	3.3%	-5.4	11618
RGD1565363	16.9%	11.5%	-5.4	11619
Atp5i	20.8%	15.4%	-5.4	11620
Gtf2h1	8.2%	2.9%	-5.4	11621
Scaf11	8.2%	2.9%	-5.4	11622
Kansl2	12.2%	6.8%	-5.4	11623
Spsb3	7.8%	2.5%	-5.4	11624
Ptpn6	7.8%	2.5%	-5.4	11625
Usp25	7.8%	2.5%	-5.4	11626
Ercc1	7.8%	2.5%	-5.4	11627
Adssl1	7.5%	2.1%	-5.4	11628
Fabp12	11.4%	6.0%	-5.4	11629
Naglu	7.1%	1.7%	-5.4	11630
Adam17	7.1%	1.7%	-5.4	11631
Mrpl39	11.0%	5.6%	-5.4	11632
Gtf3c2	6.7%	1.3%	-5.4	11633
Zswim4	6.7%	1.3%	-5.4	11634
Kctd3	6.3%	0.9%	-5.4	11635
Nudt18	14.5%	9.1%	-5.4	11636
Birc2	10.2%	4.8%	-5.4	11637
Bfar	10.2%	4.8%	-5.4	11638
Psen1	10.2%	4.8%	-5.4	11639
Cacfd1	9.8%	4.4%	-5.4	11640
Hars	9.4%	4.0%	-5.4	11641
Trappc2l	9.4%	4.0%	-5.4	11642
Rnf39	9.4%	4.0%	-5.4	11643
Sqstm1	80.0%	74.6%	-5.4	11644
Ranbp1	9.0%	3.6%	-5.4	11645
Ppial4g	12.9%	7.5%	-5.4	11646
Tor1aip1	8.6%	3.2%	-5.4	11647
Gpaa1	8.2%	2.8%	-5.4	11648
Nsmce3	8.2%	2.8%	-5.4	11649
Brox	8.2%	2.8%	-5.4	11650
Ell2	16.5%	11.0%	-5.4	11651
Anapc13	16.5%	11.0%	-5.4	11652
Psen2	12.2%	6.7%	-5.4	11653
Mtg1	7.8%	2.4%	-5.4	11654
LOC100362149	57.6%	52.2%	-5.4	11655
Slc25a42	7.5%	2.0%	-5.5	11656
Qpctl	7.5%	2.0%	-5.5	11657
Vps54	7.5%	2.0%	-5.5	11658
Ppil4	7.5%	2.0%	-5.5	11659
LOC498426	36.5%	31.0%	-5.5	11660
Ppp6c	11.4%	5.9%	-5.5	11661
RGD1308147	7.1%	1.6%	-5.5	11662
Rdm1	7.1%	1.6%	-5.5	11663
Ino80	7.1%	1.6%	-5.5	11664
Notch2	7.1%	1.6%	-5.5	11665
Emc8	11.0%	5.5%	-5.5	11666
Grtp1	6.7%	1.2%	-5.5	11667
Znhit1	14.9%	9.4%	-5.5	11668
Itgb1bp1	10.2%	4.7%	-5.5	11669
Gsap	10.2%	4.7%	-5.5	11670
Blcap	10.2%	4.7%	-5.5	11671
Dpy30	9.8%	4.3%	-5.5	11672
Polr2g	9.8%	4.3%	-5.5	11673
LOC501110	9.8%	4.3%	-5.5	11674
Lgalsl	9.4%	3.9%	-5.5	11675
Copg2	9.4%	3.9%	-5.5	11676

Edc3	9.0%	3.5%	-5.5	11677
Cse1l	9.0%	3.5%	-5.5	11678
Cnot2	9.0%	3.5%	-5.5	11679
Tmem220	9.0%	3.5%	-5.5	11680
Lrp5	9.0%	3.5%	-5.5	11681
Car2	12.9%	7.4%	-5.5	11682
C2cd2l	8.6%	3.1%	-5.5	11683
Rab38	12.5%	7.0%	-5.5	11684
Nosip	12.5%	7.0%	-5.5	11685
Coq6	8.2%	2.7%	-5.5	11686
Cdc16	8.2%	2.7%	-5.5	11687
Asna1	12.2%	6.6%	-5.5	11688
LOC100360828	32.9%	27.4%	-5.5	11689
Cnksr3	7.8%	2.3%	-5.5	11690
RGD1307100	7.8%	2.3%	-5.5	11691
Slc35a4	7.8%	2.3%	-5.5	11692
Snx11	7.8%	2.3%	-5.5	11693
Noc2l	7.5%	1.9%	-5.5	11694
Sptbn2	7.5%	1.9%	-5.5	11695
Pus3	7.1%	1.5%	-5.5	11696
Map3k3	7.1%	1.5%	-5.5	11697
Cd82	15.3%	9.8%	-5.5	11698
Srsf1	15.3%	9.8%	-5.5	11699
Gtf2e2	11.0%	5.4%	-5.5	11700
Actr3	11.0%	5.4%	-5.5	11701
Ap5m1	19.2%	13.7%	-5.5	11702
Polr1b	6.7%	1.1%	-5.5	11703
Rusc1	6.7%	1.1%	-5.5	11704
Rint1	6.7%	1.1%	-5.5	11705
Eif2s1	14.9%	9.4%	-5.5	11706
LOC100911734	10.6%	5.0%	-5.6	11707
Cpsf6	10.2%	4.6%	-5.6	11708
LOC103690427	10.2%	4.6%	-5.6	11709
Sgpp1	9.8%	4.2%	-5.6	11710
Sugct	9.8%	4.2%	-5.6	11711
Supt5h	9.4%	3.8%	-5.6	11712
Syvn1	9.4%	3.8%	-5.6	11713
Stard10	92.5%	87.0%	-5.6	11714
Mboat7l1	9.0%	3.4%	-5.6	11715
Bloc1s3	9.0%	3.4%	-5.6	11716
Mrps2	8.6%	3.0%	-5.6	11717
Mcts2	8.6%	3.0%	-5.6	11718
Herpud2	8.6%	3.0%	-5.6	11719
Oaz2	16.9%	11.3%	-5.6	11720
Catsperg1	37.6%	32.1%	-5.6	11721
Chic1	8.2%	2.6%	-5.6	11722
Fam89b	8.2%	2.6%	-5.6	11723
NEWGENE_1305281	8.2%	2.6%	-5.6	11724
Htatsf1	8.2%	2.6%	-5.6	11725
Mrpl50	12.2%	6.6%	-5.6	11726
Elovl2	7.8%	2.2%	-5.6	11727
Tgfbrap1	7.5%	1.8%	-5.6	11728
Usp6nl	7.5%	1.8%	-5.6	11729
Mto1	7.5%	1.8%	-5.6	11730
Stx5	11.4%	5.8%	-5.6	11731
Fosl1	7.1%	1.4%	-5.6	11732
Spsb2	6.7%	1.0%	-5.6	11733
Ewsr1	10.6%	5.0%	-5.6	11734
Aldh5a1	10.6%	5.0%	-5.6	11735
Atg9a	10.6%	5.0%	-5.6	11736
Il33	14.5%	8.9%	-5.6	11737
Rras2	9.8%	4.2%	-5.6	11738
Ndufaf5	9.4%	3.8%	-5.7	11739
Timm17a	9.4%	3.8%	-5.7	11740
Smurf2	9.0%	3.4%	-5.7	11741
Ubqln2	9.0%	3.4%	-5.7	11742

RGD1310127	9.0%	3.4%	-5.7	11743
Vps16	9.0%	3.4%	-5.7	11744
Il17ra	12.9%	7.3%	-5.7	11745
Hdgfrp2	8.6%	3.0%	-5.7	11746
Nmd3	8.6%	3.0%	-5.7	11747
Zhx3	8.6%	3.0%	-5.7	11748
Gmppa	16.9%	11.2%	-5.7	11749
Cyp2c6v1	100.0%	94.3%	-5.7	11750
Ptger3	8.2%	2.6%	-5.7	11751
Eepd1	8.2%	2.6%	-5.7	11752
Prkcd	8.2%	2.6%	-5.7	11753
Sox5	8.2%	2.6%	-5.7	11754
Fam107b	12.2%	6.5%	-5.7	11755
Tmf1	7.8%	2.2%	-5.7	11756
Sec24b	7.8%	2.2%	-5.7	11757
LOC103690178	7.8%	2.2%	-5.7	11758
Hn1l	11.8%	6.1%	-5.7	11759
Bcap29	11.8%	6.1%	-5.7	11760
Tbcc	11.4%	5.7%	-5.7	11761
Cabp2	32.2%	26.5%	-5.7	11762
Dars2	7.1%	1.4%	-5.7	11763
Nat8	7.1%	1.4%	-5.7	11764
Pxk	7.1%	1.4%	-5.7	11765
Ephb6	6.7%	1.0%	-5.7	11766
Cdk8	6.7%	1.0%	-5.7	11767
E2f5	6.7%	1.0%	-5.7	11768
Mrps18c	14.9%	9.2%	-5.7	11769
RGD1308706	10.2%	4.5%	-5.7	11770
RGD1566320	14.1%	8.4%	-5.7	11771
Gtf2f2	9.8%	4.1%	-5.7	11772
LOC108348098	9.8%	4.1%	-5.7	11773
Eif2s3	13.7%	8.0%	-5.7	11774
Prps1	9.4%	3.7%	-5.7	11775
RGD1305347	9.4%	3.7%	-5.7	11776
Kpna3	9.0%	3.3%	-5.7	11777
Atg4b	9.0%	3.3%	-5.7	11778
RGD1307830	9.0%	3.3%	-5.7	11779
Bpnt1	9.0%	3.3%	-5.7	11780
Apex1	9.0%	3.3%	-5.7	11781
Atp6v1e1	12.9%	7.2%	-5.7	11782
Pcsk6	8.6%	2.9%	-5.7	11783
Creld1	8.6%	2.9%	-5.7	11784
Pisd	8.6%	2.9%	-5.7	11785
Sec23b	12.5%	6.8%	-5.8	11786
Snrpa1	12.5%	6.8%	-5.8	11787
Setd3	12.5%	6.8%	-5.8	11788
Sod1	100.0%	94.2%	-5.8	11789
Kdm1b	8.2%	2.5%	-5.8	11790
Prdm2	8.2%	2.5%	-5.8	11791
Zscan26	8.2%	2.5%	-5.8	11792
Ing4	12.2%	6.4%	-5.8	11793
Srp9	12.2%	6.4%	-5.8	11794
Dnaja3	24.3%	18.5%	-5.8	11795
Aven	11.8%	6.0%	-5.8	11796
Rcor1	7.5%	1.7%	-5.8	11797
Cep350	7.5%	1.7%	-5.8	11798
Faf1	11.4%	5.6%	-5.8	11799
LOC100361933	61.2%	55.4%	-5.8	11800
Capza1	11.0%	5.2%	-5.8	11801
Champ1	6.7%	0.9%	-5.8	11802
Arrdc3	14.9%	9.1%	-5.8	11803
Ubxn2a	10.6%	4.8%	-5.8	11804
Cyth2	10.2%	4.4%	-5.8	11805
Pdcl3	10.2%	4.4%	-5.8	11806
Ap2b1	10.2%	4.4%	-5.8	11807
Slc25a32	10.2%	4.4%	-5.8	11808

Stard3	9.8%	4.0%	-5.8	11809
Smim15	9.4%	3.6%	-5.8	11810
Mterf3	9.4%	3.6%	-5.8	11811
Cbr4	13.3%	7.5%	-5.8	11812
LOC100912399	9.0%	3.2%	-5.8	11813
Elp3	8.6%	2.8%	-5.8	11814
LOC100125364	8.6%	2.8%	-5.8	11815
Trpc4ap	12.2%	6.3%	-5.8	11816
Spag9	12.2%	6.3%	-5.8	11817
Ift27	7.8%	2.0%	-5.8	11818
Cox18	7.8%	2.0%	-5.8	11819
Ube2i	16.1%	10.2%	-5.8	11820
Mrm3	7.5%	1.6%	-5.9	11821
Nrbp2	7.5%	1.6%	-5.9	11822
Slc12a7	7.5%	1.6%	-5.9	11823
RGD1562392	19.6%	13.7%	-5.9	11824
RGD1563941	11.0%	5.1%	-5.9	11825
Ctdnep1	11.0%	5.1%	-5.9	11826
Sult2a1	77.3%	71.4%	-5.9	11827
Tubb6	10.6%	4.7%	-5.9	11828
Stt3a	18.8%	12.9%	-5.9	11829
Bub3	10.2%	4.3%	-5.9	11830
Ppp4r3a	9.8%	3.9%	-5.9	11831
Ankrd27	9.8%	3.9%	-5.9	11832
Abhd6	9.8%	3.9%	-5.9	11833
Klb	9.4%	3.5%	-5.9	11834
Rnft1	9.4%	3.5%	-5.9	11835
Pex6	9.4%	3.5%	-5.9	11836
Nfyc	9.0%	3.1%	-5.9	11837
Ss18	9.0%	3.1%	-5.9	11838
Rnf111	9.0%	3.1%	-5.9	11839
Pdlim5	12.9%	7.0%	-5.9	11840
Smap2	12.9%	7.0%	-5.9	11841
Bod1	8.6%	2.7%	-5.9	11842
Nop9	8.2%	2.3%	-5.9	11843
Tdg	7.8%	1.9%	-5.9	11844
Psd4	7.8%	1.9%	-5.9	11845
Ftsj3	7.8%	1.9%	-5.9	11846
LOC100361475	11.8%	5.8%	-5.9	11847
Krit1	7.5%	1.5%	-5.9	11848
Cdk13	7.1%	1.1%	-5.9	11849
LOC100910944	27.8%	21.9%	-5.9	11850
Cars2	11.0%	5.0%	-5.9	11851
Fam149a	11.0%	5.0%	-5.9	11852
Crb3	10.6%	4.6%	-6.0	11853
Egln2	14.5%	8.6%	-6.0	11854
Sh3yl1	10.2%	4.2%	-6.0	11855
Cdk18	10.2%	4.2%	-6.0	11856
LOC100361543	14.1%	8.2%	-6.0	11857
Lars	9.8%	3.8%	-6.0	11858
Rbck1	9.0%	3.0%	-6.0	11859
Fam207a	12.9%	7.0%	-6.0	11860
Tsc22d3	8.6%	2.6%	-6.0	11861
Atg4c	8.6%	2.6%	-6.0	11862
March7	12.5%	6.6%	-6.0	11863
Rrp15	8.2%	2.2%	-6.0	11864
Terf2ip	7.8%	1.8%	-6.0	11865
Tpm3	24.3%	18.3%	-6.0	11866
Dst	11.8%	5.8%	-6.0	11867
Fam193a	7.5%	1.4%	-6.0	11868
Sirpa	7.5%	1.4%	-6.0	11869
Micu1	36.1%	30.1%	-6.0	11870
LOC103690317	11.0%	5.0%	-6.0	11871
Ero1b	11.0%	5.0%	-6.0	11872
LOC100174910	11.0%	5.0%	-6.0	11873
Suds3	14.5%	8.5%	-6.0	11874

Sfpq	10.2%	4.2%	-6.0	11875
R3hcc1	10.2%	4.2%	-6.0	11876
Cflar	14.1%	8.1%	-6.0	11877
Farsb	9.8%	3.8%	-6.0	11878
Tmem63b	9.8%	3.8%	-6.0	11879
Upf3a	9.8%	3.8%	-6.0	11880
Ppp3ca	9.8%	3.8%	-6.0	11881
Whamm	9.4%	3.4%	-6.1	11882
Ruvbl1	9.4%	3.4%	-6.1	11883
Uchl5	9.4%	3.4%	-6.1	11884
Fem1a	9.4%	3.4%	-6.1	11885
Pcmtd2	9.0%	3.0%	-6.1	11886
Lims1	9.0%	3.0%	-6.1	11887
RGD1562037	9.0%	3.0%	-6.1	11888
Micu2	12.9%	6.9%	-6.1	11889
Arfgap3	12.9%	6.9%	-6.1	11890
Agfg1	8.6%	2.6%	-6.1	11891
Rrp12	8.6%	2.6%	-6.1	11892
Tmem185a	8.6%	2.6%	-6.1	11893
Rnf128	16.9%	10.8%	-6.1	11894
Dnase2b	12.5%	6.5%	-6.1	11895
Nudc	20.8%	14.7%	-6.1	11896
Ccdc125	8.2%	2.2%	-6.1	11897
Iba57	8.2%	2.2%	-6.1	11898
Pik3c2a	8.2%	2.2%	-6.1	11899
Btaf1	8.2%	2.2%	-6.1	11900
Mcmbp	7.8%	1.8%	-6.1	11901
Fam177a1	7.5%	1.4%	-6.1	11902
Laptm4b	7.5%	1.4%	-6.1	11903
Pitpna	15.7%	9.6%	-6.1	11904
Slc35e2b	15.7%	9.6%	-6.1	11905
Ap2s1	32.2%	26.1%	-6.1	11906
Diexf	7.1%	1.0%	-6.1	11907
Esyt1	69.0%	62.9%	-6.1	11908
Map2k3	10.6%	4.5%	-6.1	11909
Dus3l	10.6%	4.5%	-6.1	11910
Pam16	14.5%	8.4%	-6.1	11911
Pin1	14.5%	8.4%	-6.1	11912
RGD1311595	10.2%	4.1%	-6.1	11913
Tjp1	10.2%	4.1%	-6.1	11914
Immp2l	9.8%	3.7%	-6.1	11915
RGD1566137	22.0%	15.8%	-6.1	11916
Dnajb12	13.3%	7.2%	-6.1	11917
Dpy19l3	13.3%	7.2%	-6.1	11918
LOC103692173	9.0%	2.9%	-6.1	11919
Krr1	8.6%	2.5%	-6.1	11920
Zfpm1	8.6%	2.5%	-6.1	11921
LOC100365810	45.9%	39.7%	-6.2	11922
Arv1	8.2%	2.1%	-6.2	11923
Commd2	8.2%	2.1%	-6.2	11924
Commd10	12.2%	6.0%	-6.2	11925
Txn1	32.9%	26.8%	-6.2	11926
Cabp1	7.5%	1.3%	-6.2	11927
Nans	11.4%	5.2%	-6.2	11928
Sec61a2	7.1%	0.9%	-6.2	11929
Mafg	11.0%	4.8%	-6.2	11930
Arid5b	6.7%	0.5%	-6.2	11931
Gfra1	6.7%	0.5%	-6.2	11932
Cdk10	10.6%	4.4%	-6.2	11933
Ift52	10.6%	4.4%	-6.2	11934
LOC100912557	31.4%	25.2%	-6.2	11935
Aarsd1	14.1%	7.9%	-6.2	11936
Ktn1	9.8%	3.6%	-6.2	11937
Scamp2	9.8%	3.6%	-6.2	11938
Pole4	13.7%	7.5%	-6.2	11939
Xrcc5	9.4%	3.2%	-6.2	11940

Trpm7	9.4%	3.2%	-6.2	11941
Lztr1	9.4%	3.2%	-6.2	11942
Lyrm5	17.6%	11.4%	-6.2	11943
RGD1302996	9.0%	2.8%	-6.2	11944
Mettl3	8.6%	2.4%	-6.2	11945
Chpf2	8.6%	2.4%	-6.2	11946
Zfx	8.6%	2.4%	-6.2	11947
Pcbp4	8.6%	2.4%	-6.2	11948
Sh3d19	12.5%	6.3%	-6.2	11949
Dicer1	8.2%	2.0%	-6.2	11950
Eaf1	8.2%	2.0%	-6.2	11951
Habp2	20.0%	13.7%	-6.3	11952
Tbce	11.0%	4.7%	-6.3	11953
Abhd5	11.0%	4.7%	-6.3	11954
Slc25a16	11.0%	4.7%	-6.3	11955
Psmg2	10.6%	4.3%	-6.3	11956
LOC100912041	10.6%	4.3%	-6.3	11957
Ubxn6	10.6%	4.3%	-6.3	11958
Rnf121	10.6%	4.3%	-6.3	11959
Tmem42	10.2%	3.9%	-6.3	11960
Helz2	10.2%	3.9%	-6.3	11961
LOC100911516	47.5%	41.2%	-6.3	11962
Pum1	14.1%	7.8%	-6.3	11963
Ubac2	14.1%	7.8%	-6.3	11964
Mb	14.1%	7.8%	-6.3	11965
Atg5	9.8%	3.5%	-6.3	11966
RGD1310495	9.8%	3.5%	-6.3	11967
Drg2	9.4%	3.1%	-6.3	11968
Mrpl19	9.4%	3.1%	-6.3	11969
E2f6	9.0%	2.7%	-6.3	11970
LOC103693474	9.0%	2.7%	-6.3	11971
B4galnt1	12.9%	6.6%	-6.3	11972
Trmt10c	8.6%	2.3%	-6.3	11973
LOC108352161	8.2%	1.9%	-6.3	11974
Mocos	8.2%	1.9%	-6.3	11975
Fam13b	8.2%	1.9%	-6.3	11976
Fyttd1	11.4%	5.0%	-6.3	11977
Tmbim1	11.4%	5.0%	-6.3	11978
Zfp330	11.4%	5.0%	-6.3	11979
Lyrm2	11.0%	4.6%	-6.3	11980
Faf2	11.0%	4.6%	-6.3	11981
Gramd3	11.0%	4.6%	-6.3	11982
Brd7	11.0%	4.6%	-6.3	11983
Pdzk1	10.6%	4.2%	-6.4	11984
Tnks2	10.2%	3.8%	-6.4	11985
Fam134b	18.4%	12.1%	-6.4	11986
Nap1l5	9.8%	3.4%	-6.4	11987
Sec31a	9.8%	3.4%	-6.4	11988
Twf1	13.7%	7.4%	-6.4	11989
Sppl2a	13.7%	7.4%	-6.4	11990
Traf2	9.4%	3.0%	-6.4	11991
Ccnh	13.3%	7.0%	-6.4	11992
Kctd5	8.6%	2.2%	-6.4	11993
Trappc2b	12.5%	6.2%	-6.4	11994
Elp5	12.5%	6.2%	-6.4	11995
Ep400	8.2%	1.8%	-6.4	11996
Map4k5	8.2%	1.8%	-6.4	11997
Rassf8	7.8%	1.4%	-6.4	11998
Chac1	16.1%	9.7%	-6.4	11999
Timm10b	11.8%	5.4%	-6.4	12000
Cela1	7.1%	0.6%	-6.4	12001
Pcna	15.3%	8.9%	-6.4	12002
Wdr43	11.0%	4.6%	-6.4	12003
Plec	11.0%	4.6%	-6.4	12004
Sart1	11.0%	4.6%	-6.4	12005
Plk2	14.9%	8.5%	-6.4	12006

Cyp2t1	14.9%	8.5%	-6.4	12007
Phc2	10.6%	4.2%	-6.4	12008
Commd4	10.6%	4.2%	-6.4	12009
Ints4	10.6%	4.2%	-6.4	12010
Pin4	18.8%	12.4%	-6.4	12011
Rab3gap1	10.2%	3.8%	-6.4	12012
Mrps22	10.2%	3.8%	-6.4	12013
LOC100911798	10.2%	3.8%	-6.4	12014
Brap	9.8%	3.4%	-6.4	12015
Tiprl	9.8%	3.4%	-6.4	12016
Aox3	9.8%	3.4%	-6.4	12017
Tns1	9.8%	3.4%	-6.4	12018
Ddit4	9.8%	3.4%	-6.4	12019
RGD1311251	9.8%	3.4%	-6.4	12020
Ctcf	9.4%	3.0%	-6.5	12021
Rnd2	9.4%	3.0%	-6.5	12022
Vps26a	13.3%	6.9%	-6.5	12023
Kctd9	9.0%	2.6%	-6.5	12024
Rnf113a2	8.6%	2.2%	-6.5	12025
Pnpt1	8.6%	2.2%	-6.5	12026
LOC106631776	12.5%	6.1%	-6.5	12027
Hrg	100.0%	93.5%	-6.5	12028
Zdhhc16	8.2%	1.8%	-6.5	12029
Tmem98	16.5%	10.0%	-6.5	12030
Zfyve21	16.1%	9.6%	-6.5	12031
Nudt21	11.8%	5.3%	-6.5	12032
RGD1309748	11.4%	4.9%	-6.5	12033
LOC100909675	10.6%	4.1%	-6.5	12034
Med21	10.6%	4.1%	-6.5	12035
Pex19	14.5%	8.0%	-6.5	12036
Ciapin1	22.7%	16.2%	-6.5	12037
Fam134a	10.2%	3.7%	-6.5	12038
Prkacb	10.2%	3.7%	-6.5	12039
Dhdds	14.1%	7.6%	-6.5	12040
Zmat2	14.1%	7.6%	-6.5	12041
Lurap1l	68.2%	61.7%	-6.5	12042
RGD1564420	9.8%	3.3%	-6.5	12043
Mtmr2	9.4%	2.9%	-6.5	12044
Pex3	9.4%	2.9%	-6.5	12045
Flii	9.4%	2.9%	-6.5	12046
RGD1310769	30.2%	23.7%	-6.5	12047
Clock	9.0%	2.5%	-6.5	12048
Usf1	8.6%	2.1%	-6.5	12049
Simc1	8.6%	2.1%	-6.5	12050
Mplkip	7.8%	1.3%	-6.6	12051
Zfp276	7.8%	1.3%	-6.6	12052
H2afv	16.1%	9.5%	-6.6	12053
Rraga	16.1%	9.5%	-6.6	12054
Adnp	11.4%	4.8%	-6.6	12055
Snx2	11.4%	4.8%	-6.6	12056
Cnot6l	11.4%	4.8%	-6.6	12057
Cyfip1	11.0%	4.4%	-6.6	12058
Alkbh7	11.0%	4.4%	-6.6	12059
Stk17b	11.0%	4.4%	-6.6	12060
LOC298111	6.7%	0.1%	-6.6	12061
LOC102546992	14.5%	7.9%	-6.6	12062
Cfap36	14.5%	7.9%	-6.6	12063
Mgrn1	10.2%	3.6%	-6.6	12064
Atp5sl	10.2%	3.6%	-6.6	12065
Jmjd6	10.2%	3.6%	-6.6	12066
Nudt6	10.2%	3.6%	-6.6	12067
Smco4	14.1%	7.5%	-6.6	12068
LOC100359600	14.1%	7.5%	-6.6	12069
Parva	9.8%	3.2%	-6.6	12070
Slc25a33	9.4%	2.8%	-6.6	12071
Actr1a	9.4%	2.8%	-6.6	12072

Zfp654	9.0%	2.4%	-6.6	12073
Spast	9.0%	2.4%	-6.6	12074
Ifi35	12.9%	6.3%	-6.6	12075
Ddx41	12.5%	5.9%	-6.6	12076
Smox	8.2%	1.6%	-6.6	12077
LOC102546766	8.2%	1.6%	-6.6	12078
Borcs8	16.5%	9.8%	-6.6	12079
Sirt5	12.2%	5.5%	-6.6	12080
Fam91a1	12.2%	5.5%	-6.6	12081
LOC679811	7.8%	1.2%	-6.6	12082
Fbxo22	11.8%	5.1%	-6.6	12083
Tp53i3	11.8%	5.1%	-6.6	12084
LOC498368	11.8%	5.1%	-6.6	12085
Copz2	11.8%	5.1%	-6.6	12086
LOC100359928	20.0%	13.3%	-6.7	12087
Trmt10b	7.5%	0.8%	-6.7	12088
Cpeb4	15.7%	9.0%	-6.7	12089
Ormdl3	15.3%	8.6%	-6.7	12090
Tmem9b	11.0%	4.3%	-6.7	12091
Slc23a1	10.6%	3.9%	-6.7	12092
Irak1	14.5%	7.8%	-6.7	12093
Pja2	9.8%	3.1%	-6.7	12094
Farsa	9.8%	3.1%	-6.7	12095
Slc25a28	9.4%	2.7%	-6.7	12096
Fbxo33	9.4%	2.7%	-6.7	12097
Rnaseh2c	9.4%	2.7%	-6.7	12098
Pds5a	9.4%	2.7%	-6.7	12099
Sptlc2	9.4%	2.7%	-6.7	12100
Lrrc8d	13.3%	6.6%	-6.7	12101
Pef1	12.9%	6.2%	-6.7	12102
Mob4	12.9%	6.2%	-6.7	12103
Scyl2	8.6%	1.9%	-6.7	12104
Nfil3	29.4%	22.7%	-6.7	12105
Fndc3a	12.5%	5.8%	-6.7	12106
Trmt6	8.2%	1.5%	-6.7	12107
Polrmt	8.2%	1.5%	-6.7	12108
Gas6	16.5%	9.8%	-6.7	12109
Slc46a1	24.7%	18.0%	-6.7	12110
Slc7a6os	12.2%	5.4%	-6.7	12111
Ccng2	12.2%	5.4%	-6.7	12112
Lsm14a	12.2%	5.4%	-6.7	12113
Rest	7.8%	1.1%	-6.7	12114
Tpgs1	11.4%	4.6%	-6.7	12115
RGD1562747	11.4%	4.6%	-6.7	12116
LOC108348111	11.4%	4.6%	-6.7	12117
Pitrm1	10.6%	3.8%	-6.8	12118
Cxxc5	14.5%	7.8%	-6.8	12119
Zranb2	10.2%	3.4%	-6.8	12120
Tbc1d20	14.1%	7.4%	-6.8	12121
Fam104b	9.8%	3.0%	-6.8	12122
Ak6	9.8%	3.0%	-6.8	12123
Syf2	18.0%	11.3%	-6.8	12124
Zmym5	9.4%	2.6%	-6.8	12125
Dnase2	9.4%	2.6%	-6.8	12126
Pkn2	9.4%	2.6%	-6.8	12127
Taz	9.4%	2.6%	-6.8	12128
Btbd16	25.9%	19.1%	-6.8	12129
Sec61g	21.6%	14.8%	-6.8	12130
Rbm22	9.0%	2.2%	-6.8	12131
Cpsf1	9.0%	2.2%	-6.8	12132
Nol12	9.0%	2.2%	-6.8	12133
Tinf2	9.0%	2.2%	-6.8	12134
Stam	8.6%	1.8%	-6.8	12135
Phf13	8.6%	1.8%	-6.8	12136
Fam63a	8.6%	1.8%	-6.8	12137
Csde1	16.9%	10.1%	-6.8	12138

rnf141	12.5%	5.8%	-6.8	12139
St6galnac4	8.2%	1.4%	-6.8	12140
Map4k3	8.2%	1.4%	-6.8	12141
Ubn1	8.2%	1.4%	-6.8	12142
Eapp	12.2%	5.4%	-6.8	12143
Cry1	7.8%	1.0%	-6.8	12144
Gpc4	11.8%	5.0%	-6.8	12145
Sh3gl3	11.8%	5.0%	-6.8	12146
Zfp148	11.8%	5.0%	-6.8	12147
Tnfaip8l1	11.4%	4.6%	-6.8	12148
Zmynd11	11.4%	4.6%	-6.8	12149
Fastk	11.0%	4.2%	-6.8	12150
Hdhd3	14.9%	8.1%	-6.8	12151
LOC102551259	10.6%	3.8%	-6.8	12152
Eif2a	10.6%	3.8%	-6.8	12153
Hif1a	10.6%	3.8%	-6.8	12154
Hilpda	10.2%	3.4%	-6.8	12155
Pop5	10.2%	3.4%	-6.8	12156
Ipmk	10.2%	3.4%	-6.8	12157
Fmc1	14.1%	7.3%	-6.8	12158
Cdip1	14.1%	7.3%	-6.8	12159
Xrcc1	9.4%	2.6%	-6.9	12160
Pus1	9.4%	2.6%	-6.9	12161
Mmp15	9.4%	2.6%	-6.9	12162
Mfsd14b	9.4%	2.6%	-6.9	12163
Mrpl51	13.3%	6.5%	-6.9	12164
Fam126b	9.0%	2.2%	-6.9	12165
Camk1	12.9%	6.1%	-6.9	12166
Men1	8.6%	1.8%	-6.9	12167
Lmtk2	8.6%	1.8%	-6.9	12168
Phlpp1	8.6%	1.8%	-6.9	12169
Dgcr6	16.9%	10.0%	-6.9	12170
Tpi1	33.3%	26.5%	-6.9	12171
Ankrd33b	8.2%	1.4%	-6.9	12172
Msmo1	24.7%	17.8%	-6.9	12173
Myg1	12.2%	5.3%	-6.9	12174
Ebna1bp2	12.2%	5.3%	-6.9	12175
LOC103690006	12.2%	5.3%	-6.9	12176
Mrpl4	11.8%	4.9%	-6.9	12177
Cyp4f39	11.8%	4.9%	-6.9	12178
Edar	7.5%	0.6%	-6.9	12179
Tm9sf3	36.5%	29.6%	-6.9	12180
Tln1	11.4%	4.5%	-6.9	12181
Ccdc53	11.4%	4.5%	-6.9	12182
Cfdp1	90.2%	83.3%	-6.9	12183
Afg3l1	10.6%	3.7%	-6.9	12184
Enpep	10.6%	3.7%	-6.9	12185
Entpd8	10.6%	3.7%	-6.9	12186
Rtp4	18.8%	11.9%	-6.9	12187
LOC102546572	10.2%	3.3%	-6.9	12188
Lrrc8a	9.8%	2.9%	-6.9	12189
Stard3nl	9.8%	2.9%	-6.9	12190
Ubfd1	9.8%	2.9%	-6.9	12191
Clcc1	9.4%	2.5%	-6.9	12192
Usp36	9.0%	2.1%	-6.9	12193
Ncbp1	9.0%	2.1%	-6.9	12194
Dnase1l1	9.0%	2.1%	-6.9	12195
Lgmn	12.9%	6.0%	-6.9	12196
Commd7	8.6%	1.7%	-6.9	12197
Nkap	8.6%	1.7%	-6.9	12198
Lyplal1	12.5%	5.6%	-7.0	12199
Mmaa	8.2%	1.3%	-7.0	12200
Abcb1b	16.5%	9.5%	-7.0	12201
Rpl7l1	16.5%	9.5%	-7.0	12202
Cyp3a62	12.2%	5.2%	-7.0	12203
Ube2e3	12.2%	5.2%	-7.0	12204

Tapbp	12.2%	5.2%	-7.0	12205
Tmub2	11.8%	4.8%	-7.0	12206
Sap30l	11.4%	4.4%	-7.0	12207
LOC678760	11.4%	4.4%	-7.0	12208
Tsfm	11.4%	4.4%	-7.0	12209
Commd6	19.6%	12.6%	-7.0	12210
LOC108349561	19.2%	12.2%	-7.0	12211
Lin37	10.6%	3.6%	-7.0	12212
Ercc3	10.6%	3.6%	-7.0	12213
Cap1	14.5%	7.5%	-7.0	12214
Fam21c	10.2%	3.2%	-7.0	12215
Naa35	10.2%	3.2%	-7.0	12216
Rab11fip1	10.2%	3.2%	-7.0	12217
Ankrd40	10.2%	3.2%	-7.0	12218
Trmt1	10.2%	3.2%	-7.0	12219
Senp2	9.8%	2.8%	-7.0	12220
LOC108348046	9.8%	2.8%	-7.0	12221
Abracl	9.8%	2.8%	-7.0	12222
Il6r	9.8%	2.8%	-7.0	12223
Eid1	13.7%	6.7%	-7.0	12224
LOC100911422	22.0%	14.9%	-7.0	12225
Zfp131	9.4%	2.4%	-7.0	12226
Gpr180	9.4%	2.4%	-7.0	12227
RGD1565588	21.6%	14.5%	-7.0	12228
Pak1	9.0%	2.0%	-7.0	12229
Prr16	9.0%	2.0%	-7.0	12230
Utp4	9.0%	2.0%	-7.0	12231
Steap3	12.5%	5.5%	-7.0	12232
Gon7	12.2%	5.1%	-7.0	12233
B4galt7	7.8%	0.8%	-7.0	12234
Nox4	7.8%	0.8%	-7.0	12235
Acot4	16.1%	9.0%	-7.0	12236
Sdf2l1	16.1%	9.0%	-7.0	12237
Cetn2	15.7%	8.6%	-7.1	12238
LOC102551539	11.4%	4.3%	-7.1	12239
Chmp1b	11.4%	4.3%	-7.1	12240
Pcbd2	15.3%	8.2%	-7.1	12241
Smim19	11.0%	3.9%	-7.1	12242
Trappc12	11.0%	3.9%	-7.1	12243
Thap7	10.6%	3.5%	-7.1	12244
Acp6	10.6%	3.5%	-7.1	12245
Slc48a1	10.2%	3.1%	-7.1	12246
Mcts1	26.7%	19.6%	-7.1	12247
Arpc1a	14.1%	7.0%	-7.1	12248
Tmem150c	9.8%	2.7%	-7.1	12249
Zranb1	9.8%	2.7%	-7.1	12250
Tmem168	9.8%	2.7%	-7.1	12251
Shpk	9.8%	2.7%	-7.1	12252
Cd14	97.3%	90.2%	-7.1	12253
Use1	18.0%	11.0%	-7.1	12254
Prkra	9.4%	2.3%	-7.1	12255
Rrp7a	13.3%	6.2%	-7.1	12256
Crbn	13.3%	6.2%	-7.1	12257
Tbcd	9.0%	1.9%	-7.1	12258
Pdk1	20.8%	13.7%	-7.1	12259
Rabep1	12.2%	5.0%	-7.1	12260
Ntmt1	11.4%	4.2%	-7.1	12261
Lta4h	11.4%	4.2%	-7.1	12262
Akap1	11.4%	4.2%	-7.1	12263
Nme1	15.3%	8.2%	-7.1	12264
Polr2c	11.0%	3.8%	-7.1	12265
Tarbp2	10.6%	3.4%	-7.2	12266
Fkbp9	10.6%	3.4%	-7.2	12267
Plp2	10.6%	3.4%	-7.2	12268
Xpo6	10.2%	3.0%	-7.2	12269
Rnf138	10.2%	3.0%	-7.2	12270

Sphk2	10.2%	3.0%	-7.2	12271
Slc6a12	10.2%	3.0%	-7.2	12272
Cryz	14.1%	7.0%	-7.2	12273
Chmp4bl1	14.1%	7.0%	-7.2	12274
Nfib	9.8%	2.6%	-7.2	12275
Rab5c	22.0%	14.8%	-7.2	12276
Lrrc8b	9.4%	2.2%	-7.2	12277
Hscb	9.4%	2.2%	-7.2	12278
Ncor1	13.3%	6.2%	-7.2	12279
Zer1	9.0%	1.8%	-7.2	12280
Arfgef1	9.0%	1.8%	-7.2	12281
Ythdf2	12.9%	5.8%	-7.2	12282
Thap4	8.6%	1.4%	-7.2	12283
Memo1	12.5%	5.4%	-7.2	12284
Fga	100.0%	92.8%	-7.2	12285
Ppid	16.1%	8.9%	-7.2	12286
Rexo2	16.1%	8.9%	-7.2	12287
Hexim1	15.7%	8.5%	-7.2	12288
Ppfibp1	11.4%	4.2%	-7.2	12289
Rb1cc1	11.4%	4.2%	-7.2	12290
Oxa1l	19.6%	12.4%	-7.2	12291
Ddx19a	11.0%	3.8%	-7.2	12292
Btbd1	10.6%	3.4%	-7.2	12293
F8a1	10.6%	3.4%	-7.2	12294
Stard7	14.5%	7.3%	-7.2	12295
Mxd4	18.4%	11.2%	-7.2	12296
Plod1	9.8%	2.6%	-7.2	12297
Thumpd1	9.8%	2.6%	-7.2	12298
Psma3l	26.3%	19.0%	-7.2	12299
Tomm40	13.7%	6.5%	-7.3	12300
Mgea5	13.7%	6.5%	-7.3	12301
Rnf144b	25.5%	18.2%	-7.3	12302
Arid5a	12.9%	5.7%	-7.3	12303
Tusc2	12.9%	5.7%	-7.3	12304
Eif2ak1	12.9%	5.7%	-7.3	12305
Orc6	21.2%	13.9%	-7.3	12306
Spink3	8.6%	1.4%	-7.3	12307
Fpgs	12.5%	5.3%	-7.3	12308
Nudt9	12.5%	5.3%	-7.3	12309
Spop	12.5%	5.3%	-7.3	12310
Ctdsp2	12.2%	4.9%	-7.3	12311
Rsad2	12.2%	4.9%	-7.3	12312
Abi1	11.8%	4.5%	-7.3	12313
Chuk	11.8%	4.5%	-7.3	12314
Rchy1	11.8%	4.5%	-7.3	12315
Ube2j2	11.8%	4.5%	-7.3	12316
Pbxip1	11.4%	4.1%	-7.3	12317
Pcyt1a	11.4%	4.1%	-7.3	12318
Mettl23	11.0%	3.7%	-7.3	12319
Dcakd	11.0%	3.7%	-7.3	12320
LOC100911515	31.8%	24.5%	-7.3	12321
Fibp	10.6%	3.3%	-7.3	12322
Ppat	10.6%	3.3%	-7.3	12323
Bin3	10.2%	2.9%	-7.3	12324
LOC619574	10.2%	2.9%	-7.3	12325
Usp7	14.1%	6.8%	-7.3	12326
Noc4l	9.8%	2.5%	-7.3	12327
Abhd13	9.8%	2.5%	-7.3	12328
Bcas2	13.7%	6.4%	-7.3	12329
Mcoln1	13.7%	6.4%	-7.3	12330
Glce	9.4%	2.1%	-7.3	12331
Mrpl46	13.3%	6.0%	-7.3	12332
Slirp	17.3%	9.9%	-7.3	12333
Dpagt1	12.9%	5.6%	-7.3	12334
LOC100910732	12.9%	5.6%	-7.3	12335
Gusb	12.9%	5.6%	-7.3	12336

Zdhhc7	8.6%	1.3%	-7.3	12337
Cisd2	12.5%	5.2%	-7.4	12338
Ap3m1	12.5%	5.2%	-7.4	12339
Mrpl40	12.5%	5.2%	-7.4	12340
Cpn1	12.2%	4.8%	-7.4	12341
Ccdc12	12.2%	4.8%	-7.4	12342
Fam175b	11.4%	4.0%	-7.4	12343
Trim27	11.4%	4.0%	-7.4	12344
Vrk3	11.0%	3.6%	-7.4	12345
Tbl3	11.0%	3.6%	-7.4	12346
Gnptg	11.0%	3.6%	-7.4	12347
Pik3ap1	14.9%	7.5%	-7.4	12348
Arfgap1	10.6%	3.2%	-7.4	12349
Tbc1d17	10.6%	3.2%	-7.4	12350
Stat2	10.6%	3.2%	-7.4	12351
Sdsl	10.2%	2.8%	-7.4	12352
Psmb10	31.0%	23.6%	-7.4	12353
Ttc7a	9.8%	2.4%	-7.4	12354
Scfd1	13.7%	6.3%	-7.4	12355
Dusp18	46.7%	39.2%	-7.4	12356
Sgms2	9.0%	1.6%	-7.4	12357
Pm20d1	12.9%	5.5%	-7.4	12358
Slco2a1	12.9%	5.5%	-7.4	12359
Alg9	8.6%	1.2%	-7.4	12360
Srrm1	12.5%	5.1%	-7.4	12361
Hypk	20.8%	13.3%	-7.4	12362
Cfap20	16.5%	9.0%	-7.4	12363
Ubl7	12.2%	4.7%	-7.4	12364
Mrps26	12.2%	4.7%	-7.4	12365
Gas5	15.7%	8.2%	-7.5	12366
Cyp7a1	11.4%	3.9%	-7.5	12367
Hspa13	11.4%	3.9%	-7.5	12368
Ndst1	14.9%	7.4%	-7.5	12369
Uba1	56.5%	49.0%	-7.5	12370
Arhgap35	10.6%	3.1%	-7.5	12371
Tec	10.6%	3.1%	-7.5	12372
Immp1l	10.2%	2.7%	-7.5	12373
Stau1	14.1%	6.6%	-7.5	12374
Rhbdf2	9.4%	1.9%	-7.5	12375
Egln1	9.4%	1.9%	-7.5	12376
Sts	12.9%	5.4%	-7.5	12377
Dync1i2	12.9%	5.4%	-7.5	12378
Cldn12	12.5%	5.0%	-7.5	12379
Crat	12.5%	5.0%	-7.5	12380
LOC100363469	54.1%	46.6%	-7.5	12381
LOC679539	20.8%	13.3%	-7.5	12382
Crk	16.5%	9.0%	-7.5	12383
Pfdn5	20.4%	12.9%	-7.5	12384
Akt1	11.4%	3.8%	-7.5	12385
LOC103692171	11.4%	3.8%	-7.5	12386
Hspa9	19.6%	12.1%	-7.5	12387
Ncstn	11.0%	3.4%	-7.5	12388
Pigs	11.0%	3.4%	-7.5	12389
Ulk1	11.0%	3.4%	-7.5	12390
Mmadhc	19.2%	11.7%	-7.5	12391
Gpbp1	14.5%	7.0%	-7.6	12392
Pcf11	10.2%	2.6%	-7.6	12393
Mfsd6	10.2%	2.6%	-7.6	12394
Rbpms2	14.1%	6.6%	-7.6	12395
Ttc3	9.8%	2.2%	-7.6	12396
Cdc42bpb	9.8%	2.2%	-7.6	12397
Epcam	13.7%	6.2%	-7.6	12398
Elof1	13.3%	5.8%	-7.6	12399
Bre	13.3%	5.8%	-7.6	12400
LOC100912618	21.6%	14.0%	-7.6	12401
Serinc2	25.5%	17.9%	-7.6	12402

Rnf114	12.9%	5.4%	-7.6	12403
Srp19	12.5%	5.0%	-7.6	12404
Rnf7	24.7%	17.1%	-7.6	12405
Gatad1	12.2%	4.6%	-7.6	12406
Fas	12.2%	4.6%	-7.6	12407
Akr1c3	7.8%	0.2%	-7.6	12408
Napsa	28.6%	21.0%	-7.6	12409
Mesdc2	11.8%	4.2%	-7.6	12410
Drap1	28.2%	20.6%	-7.6	12411
Abca1	15.3%	7.7%	-7.6	12412
Dpcd	11.0%	3.4%	-7.6	12413
Polr1d	10.6%	3.0%	-7.6	12414
Sepsecs	14.5%	6.9%	-7.6	12415
Gbf1	10.2%	2.6%	-7.6	12416
Tmem5	10.2%	2.6%	-7.6	12417
Ip6k1	10.2%	2.6%	-7.6	12418
Nln	18.4%	10.8%	-7.6	12419
Lias	14.1%	6.5%	-7.6	12420
Plpp3	22.4%	14.7%	-7.6	12421
Nup54	9.8%	2.2%	-7.6	12422
Usp16	13.3%	5.7%	-7.7	12423
Srp68	13.3%	5.7%	-7.7	12424
LOC100364335	17.3%	9.6%	-7.7	12425
Rarres1	12.9%	5.3%	-7.7	12426
Uba3	12.9%	5.3%	-7.7	12427
Phrf1	8.6%	1.0%	-7.7	12428
Pex14	12.5%	4.9%	-7.7	12429
Creld2	12.5%	4.9%	-7.7	12430
C1galt1c1	12.2%	4.5%	-7.7	12431
Tnpo1	12.2%	4.5%	-7.7	12432
LOC108348144	40.8%	33.1%	-7.7	12433
Slc25a22	15.7%	8.0%	-7.7	12434
Ern1	11.4%	3.7%	-7.7	12435
Arntl	11.0%	3.3%	-7.7	12436
Nemf	11.0%	3.3%	-7.7	12437
Ube2g2	14.9%	7.2%	-7.7	12438
Nek9	10.6%	2.9%	-7.7	12439
Rorc	10.2%	2.5%	-7.7	12440
Ccnd1	14.1%	6.4%	-7.7	12441
Rab28	14.1%	6.4%	-7.7	12442
Elp2	9.8%	2.1%	-7.7	12443
E2f4	9.8%	2.1%	-7.7	12444
Pik3ca	9.8%	2.1%	-7.7	12445
Ranbp9	9.8%	2.1%	-7.7	12446
Mrpl48	13.7%	6.0%	-7.7	12447
LOC684557	13.3%	5.6%	-7.7	12448
Fam234a	9.0%	1.3%	-7.7	12449
Gorasp2	17.3%	9.5%	-7.7	12450
St3gal1	12.9%	5.2%	-7.7	12451
Cops3	12.9%	5.2%	-7.7	12452
Mocs1	12.5%	4.8%	-7.8	12453
Abhd4	12.5%	4.8%	-7.8	12454
Rab20	12.2%	4.4%	-7.8	12455
Rbm17	12.2%	4.4%	-7.8	12456
Psme3	16.1%	8.3%	-7.8	12457
Vgll4	11.8%	4.0%	-7.8	12458
Zc3h14	11.8%	4.0%	-7.8	12459
Acsl3	11.8%	4.0%	-7.8	12460
Mreg	32.5%	24.8%	-7.8	12461
Atp13a1	11.4%	3.6%	-7.8	12462
Cebpa	36.1%	28.3%	-7.8	12463
Tnpo3	11.0%	3.2%	-7.8	12464
Nipbl	11.0%	3.2%	-7.8	12465
Ces2g	11.0%	3.2%	-7.8	12466
Ufl1	10.6%	2.8%	-7.8	12467
Dph1	14.5%	6.7%	-7.8	12468

P3h1	10.2%	2.4%	-7.8	12469
Alg14	10.2%	2.4%	-7.8	12470
Polr1c	13.7%	5.9%	-7.8	12471
Uxt	13.7%	5.9%	-7.8	12472
RGD1310209	9.4%	1.6%	-7.8	12473
LOC103690013	13.3%	5.5%	-7.8	12474
Cers6	9.0%	1.2%	-7.8	12475
Il18bp	12.9%	5.1%	-7.8	12476
N4bp2l2	12.9%	5.1%	-7.8	12477
Mrpl42	16.9%	9.0%	-7.8	12478
Cdc5l	12.5%	4.7%	-7.8	12479
Sf3b5	12.5%	4.7%	-7.8	12480
Tssc4	12.5%	4.7%	-7.8	12481
LOC100362400	28.6%	20.8%	-7.8	12482
Pcmtd1	16.1%	8.2%	-7.8	12483
Ltc4s	15.3%	7.4%	-7.9	12484
Kras	14.9%	7.0%	-7.9	12485
Rab21	10.6%	2.7%	-7.9	12486
Pgam5	10.2%	2.3%	-7.9	12487
Senp6	14.1%	6.2%	-7.9	12488
Klf9	22.4%	14.5%	-7.9	12489
Dlst	22.4%	14.5%	-7.9	12490
Otud4	9.8%	1.9%	-7.9	12491
Smad4	9.8%	1.9%	-7.9	12492
Mrpl53	13.7%	5.8%	-7.9	12493
Fbxl3	13.7%	5.8%	-7.9	12494
RGD1565616	9.4%	1.5%	-7.9	12495
Nr1h2	13.3%	5.4%	-7.9	12496
Tars2	12.9%	5.0%	-7.9	12497
Mdfic	12.9%	5.0%	-7.9	12498
Trim32	8.6%	0.7%	-7.9	12499
Stx4	16.9%	9.0%	-7.9	12500
Nono	16.5%	8.6%	-7.9	12501
Ppp1r11	16.5%	8.6%	-7.9	12502
Mrps6	12.2%	4.2%	-7.9	12503
Acot8	11.8%	3.8%	-7.9	12504
Larp4b	11.4%	3.4%	-7.9	12505
Lsm2	11.4%	3.4%	-7.9	12506
Mgmt	23.5%	15.6%	-7.9	12507
Pigc	11.0%	3.0%	-7.9	12508
LOC361635	11.0%	3.0%	-7.9	12509
Ebag9	14.9%	7.0%	-7.9	12510
Daxx	14.5%	6.6%	-8.0	12511
Rnf115	13.3%	5.4%	-8.0	12512
Cyp2e1	100.0%	92.0%	-8.0	12513
Ube2v1	20.8%	12.8%	-8.0	12514
Ces2j	28.6%	20.6%	-8.0	12515
LOC100912008	11.8%	3.8%	-8.0	12516
Ubl5	32.2%	24.1%	-8.0	12517
LOC103689986	14.9%	6.9%	-8.0	12518
Mkks	18.8%	10.8%	-8.0	12519
Prdx1l1	26.7%	18.6%	-8.0	12520
Alkbh3	13.3%	5.3%	-8.1	12521
Gga2	12.9%	4.9%	-8.1	12522
Rab11fip5	8.6%	0.6%	-8.1	12523
Cln8	12.5%	4.5%	-8.1	12524
Auh	20.8%	12.7%	-8.1	12525
Rnf217	12.2%	4.1%	-8.1	12526
Eif2s3y	20.4%	12.3%	-8.1	12527
Cidec	24.3%	16.2%	-8.1	12528
Pbdc1	11.8%	3.7%	-8.1	12529
Eed	11.8%	3.7%	-8.1	12530
Parp1	11.8%	3.7%	-8.1	12531
Ube2j1	20.0%	11.9%	-8.1	12532
Manbal	11.4%	3.3%	-8.1	12533
Arih2	11.4%	3.3%	-8.1	12534

Cd1d1	15.3%	7.2%	-8.1	12535
Metap2	15.3%	7.2%	-8.1	12536
LOC680142	14.5%	6.4%	-8.1	12537
Psmg3	14.5%	6.4%	-8.1	12538
Ipo4	10.2%	2.1%	-8.1	12539
Wwp1	14.1%	6.0%	-8.1	12540
Slc25a38	13.7%	5.6%	-8.1	12541
Il11ra1	13.3%	5.2%	-8.1	12542
Ythdf1	13.3%	5.2%	-8.1	12543
Dnttip1	13.3%	5.2%	-8.1	12544
Agpat3	17.3%	9.1%	-8.1	12545
Rpe	12.5%	4.4%	-8.2	12546
Ybx3	12.5%	4.4%	-8.2	12547
Itih4	100.0%	91.8%	-8.2	12548
Srsf5	27.5%	19.3%	-8.2	12549
Nubp2	14.9%	6.7%	-8.2	12550
Gm2a	43.9%	35.7%	-8.2	12551
Rnf146	10.6%	2.4%	-8.2	12552
Cd3eap	10.6%	2.4%	-8.2	12553
Fbxw9	10.2%	2.0%	-8.2	12554
Slc13a4	10.2%	2.0%	-8.2	12555
Stx18	14.1%	5.9%	-8.2	12556
LOC288913	18.0%	9.8%	-8.2	12557
Ddx46	13.7%	5.5%	-8.2	12558
Ppp4r3b	13.7%	5.5%	-8.2	12559
Fam120a	17.3%	9.0%	-8.2	12560
Dap3	12.9%	4.7%	-8.2	12561
RGD1561113	12.9%	4.7%	-8.2	12562
Sdhaf2	16.9%	8.6%	-8.2	12563
Flad1	16.9%	8.6%	-8.2	12564
Ppm1k	12.5%	4.3%	-8.2	12565
Wipi2	12.2%	3.9%	-8.2	12566
Strn3	12.2%	3.9%	-8.2	12567
Jmjd1c	16.1%	7.8%	-8.2	12568
Rpl36	49.4%	41.2%	-8.2	12569
Malsu1	11.8%	3.5%	-8.2	12570
Smn1	11.0%	2.7%	-8.3	12571
Dbt	14.9%	6.6%	-8.3	12572
Ndufaf2	14.9%	6.6%	-8.3	12573
Fgfr4	10.6%	2.3%	-8.3	12574
Glrx2	14.5%	6.2%	-8.3	12575
Fars2	10.2%	1.9%	-8.3	12576
Tomm5	18.4%	10.2%	-8.3	12577
Glod4	18.4%	10.2%	-8.3	12578
Pdf	14.1%	5.8%	-8.3	12579
Uhrf2	9.4%	1.1%	-8.3	12580
Tspan6	12.9%	4.6%	-8.3	12581
Ppfibp2	12.5%	4.2%	-8.3	12582
Atp2b1	12.5%	4.2%	-8.3	12583
RGD1306954	16.5%	8.2%	-8.3	12584
Dynlt3	16.5%	8.2%	-8.3	12585
Gne	20.4%	12.1%	-8.3	12586
Snx5	16.1%	7.8%	-8.3	12587
Ssfa2	11.8%	3.4%	-8.3	12588
Zcchc6	15.7%	7.4%	-8.3	12589
Nfu1	11.4%	3.0%	-8.3	12590
RGD1306148	15.3%	7.0%	-8.3	12591
RGD1565784	15.3%	7.0%	-8.3	12592
Rdh11	14.9%	6.6%	-8.3	12593
Lsm5	10.6%	2.2%	-8.4	12594
RGD1309730	18.8%	10.5%	-8.4	12595
Hn1	14.5%	6.2%	-8.4	12596
Pnrc2	18.4%	10.1%	-8.4	12597
Ehd1	18.4%	10.1%	-8.4	12598
Nol8	9.8%	1.4%	-8.4	12599
Dnajc5	13.7%	5.4%	-8.4	12600

Prelid3b	13.7%	5.4%	-8.4	12601
Dgat1	13.3%	5.0%	-8.4	12602
Larp4	13.3%	5.0%	-8.4	12603
Casp3	16.9%	8.5%	-8.4	12604
Srm	16.9%	8.5%	-8.4	12605
Rrp1	12.5%	4.2%	-8.4	12606
Fth1	100.0%	91.6%	-8.4	12607
Adh7	16.5%	8.1%	-8.4	12608
Gtf2h5	16.5%	8.1%	-8.4	12609
Brd2	20.4%	12.0%	-8.4	12610
Naa50	16.1%	7.7%	-8.4	12611
Asxl1	11.8%	3.4%	-8.4	12612
Fbxwl1	15.7%	7.3%	-8.4	12613
Ppp2r5d	11.4%	3.0%	-8.4	12614
Mcart1	19.6%	11.2%	-8.4	12615
Sltm	11.0%	2.6%	-8.4	12616
Aco2	27.5%	19.0%	-8.4	12617
Txndc9	14.9%	6.5%	-8.4	12618
Gprc5c	14.9%	6.5%	-8.4	12619
Slc25a17	10.6%	2.2%	-8.4	12620
Pafah1b2	14.5%	6.1%	-8.4	12621
Srd5a1	59.6%	51.2%	-8.4	12622
Tnfsf13	9.4%	1.0%	-8.5	12623
Dapk3	21.6%	13.1%	-8.5	12624
LOC361990	17.3%	8.8%	-8.5	12625
Ss18l2	21.2%	12.7%	-8.5	12626
Pes1	12.2%	3.7%	-8.5	12627
Mro	15.7%	7.2%	-8.5	12628
Uspl1	11.4%	2.9%	-8.5	12629
Brd3	11.4%	2.9%	-8.5	12630
Ier2	32.2%	23.7%	-8.5	12631
LOC688655	44.3%	35.8%	-8.5	12632
Dennd5a	11.0%	2.5%	-8.5	12633
Pigx	14.9%	6.4%	-8.5	12634
Ireb2	14.9%	6.4%	-8.5	12635
Tmc3	10.6%	2.1%	-8.5	12636
Cant1	10.6%	2.1%	-8.5	12637
Dnajc12	22.7%	14.2%	-8.5	12638
Clpb	14.1%	5.6%	-8.5	12639
Nabp2	14.1%	5.6%	-8.5	12640
Dvl1	13.7%	5.2%	-8.5	12641
Sac3d1	9.4%	0.9%	-8.5	12642
RGD1559459	13.3%	4.8%	-8.5	12643
Cars	13.3%	4.8%	-8.5	12644
Anapc2	13.3%	4.8%	-8.5	12645
Dhrs1	12.9%	4.4%	-8.5	12646
Lrpap1	16.9%	8.3%	-8.5	12647
Ddx49	12.5%	4.0%	-8.6	12648
Nbn	12.5%	4.0%	-8.6	12649
Fech	20.8%	12.2%	-8.6	12650
Tor1b	16.5%	7.9%	-8.6	12651
Lsm12	16.5%	7.9%	-8.6	12652
Wbscr22	12.2%	3.6%	-8.6	12653
Slc35f5	12.2%	3.6%	-8.6	12654
Rrs1	12.2%	3.6%	-8.6	12655
Gkap1	11.8%	3.2%	-8.6	12656
Calu	20.0%	11.4%	-8.6	12657
Psat1	11.4%	2.8%	-8.6	12658
Kdm1a	11.4%	2.8%	-8.6	12659
Unc50	11.4%	2.8%	-8.6	12660
Plrg1	27.8%	19.3%	-8.6	12661
Apoo	11.0%	2.4%	-8.6	12662
Hip1r	11.0%	2.4%	-8.6	12663
LOC102546489	14.9%	6.3%	-8.6	12664
Atp6v0a1	14.5%	5.9%	-8.6	12665
Washc3	14.5%	5.9%	-8.6	12666

Sdccag3	14.5%	5.9%	-8.6	12667
Mex3c	10.2%	1.6%	-8.6	12668
Hmox2	18.4%	9.8%	-8.6	12669
Znrf1	14.1%	5.5%	-8.6	12670
Khdrbs1	14.1%	5.5%	-8.6	12671
Aga	18.0%	9.4%	-8.6	12672
Naxe	18.0%	9.4%	-8.6	12673
Furin	22.0%	13.3%	-8.6	12674
Dpp3	13.3%	4.7%	-8.6	12675
Frmd6	9.0%	0.4%	-8.6	12676
Zfp655	12.9%	4.3%	-8.6	12677
Rap2c	12.9%	4.3%	-8.6	12678
Aldh16a1	12.5%	3.9%	-8.6	12679
Apba3	16.5%	7.8%	-8.6	12680
Dsc2	16.1%	7.4%	-8.6	12681
Mccc1	11.8%	3.1%	-8.6	12682
Hmbs	11.8%	3.1%	-8.6	12683
Cdk5rap3	15.7%	7.0%	-8.7	12684
Ik	19.2%	10.6%	-8.7	12685
Gadd45a	56.5%	47.8%	-8.7	12686
Zfand3	14.5%	5.8%	-8.7	12687
Chchd3	22.7%	14.1%	-8.7	12688
P4ha2	10.2%	1.5%	-8.7	12689
Tesk1	10.2%	1.5%	-8.7	12690
Sft2d1	13.7%	5.0%	-8.7	12691
Cdk9	13.7%	5.0%	-8.7	12692
Yipf2	13.7%	5.0%	-8.7	12693
Setdb2	17.6%	9.0%	-8.7	12694
Zfp771	12.9%	4.2%	-8.7	12695
Gtf3a	16.9%	8.2%	-8.7	12696
Aim1l	12.5%	3.8%	-8.7	12697
Cryl1	12.5%	3.8%	-8.7	12698
LOC306766	12.5%	3.8%	-8.7	12699
Arpc1b	20.8%	12.1%	-8.7	12700
LOC367746	11.8%	3.0%	-8.7	12701
Glrx	28.2%	19.5%	-8.7	12702
Ndufaf4	11.4%	2.6%	-8.7	12703
Mycbp	15.3%	6.6%	-8.7	12704
Atf1	14.9%	6.2%	-8.7	12705
Hs2st1	10.6%	1.8%	-8.7	12706
Cops9	26.7%	17.9%	-8.8	12707
LOC690000	13.7%	5.0%	-8.8	12708
Nfkb2	13.7%	5.0%	-8.8	12709
Cables2	13.7%	5.0%	-8.8	12710
Timm29	13.7%	5.0%	-8.8	12711
Mtmr14	12.9%	4.2%	-8.8	12712
Phf5a	16.9%	8.1%	-8.8	12713
Manba	12.5%	3.8%	-8.8	12714
Mtfr1	12.5%	3.8%	-8.8	12715
Card10	12.5%	3.8%	-8.8	12716
Tmed1	12.2%	3.4%	-8.8	12717
Anapc5	20.4%	11.6%	-8.8	12718
Rab24	16.1%	7.3%	-8.8	12719
Fabp5	16.1%	7.3%	-8.8	12720
Fam133b	11.8%	3.0%	-8.8	12721
LOC108348266	11.8%	3.0%	-8.8	12722
Wsb2	11.8%	3.0%	-8.8	12723
Nfs1	20.0%	11.2%	-8.8	12724
Golga5	11.0%	2.2%	-8.8	12725
Epb41l5	11.0%	2.2%	-8.8	12726
Ptbp3	14.9%	6.1%	-8.8	12727
Wrap73	10.6%	1.8%	-8.8	12728
Hccs	10.6%	1.8%	-8.8	12729
Dram1	18.4%	9.6%	-8.8	12730
Cwc15	38.8%	30.0%	-8.8	12731
Cttn	13.7%	4.9%	-8.8	12732

LOC108348082	13.3%	4.5%	-8.9	12733
Dnajc21	12.9%	4.1%	-8.9	12734
Ntpcr	16.9%	8.0%	-8.9	12735
Kpna2	16.9%	8.0%	-8.9	12736
Timm22	12.5%	3.7%	-8.9	12737
Coro1c	12.5%	3.7%	-8.9	12738
Igsf11	12.2%	3.3%	-8.9	12739
Dsg2	16.1%	7.2%	-8.9	12740
Psph	11.4%	2.5%	-8.9	12741
Mrfap1	35.7%	26.8%	-8.9	12742
Rhob	23.1%	14.2%	-8.9	12743
Lage3	18.8%	9.9%	-8.9	12744
Hipk3	14.5%	5.6%	-8.9	12745
Papd4	14.5%	5.6%	-8.9	12746
Scamp4	14.1%	5.2%	-8.9	12747
mrpl24	14.1%	5.2%	-8.9	12748
Grpel2	14.1%	5.2%	-8.9	12749
Mfsd3	14.1%	5.2%	-8.9	12750
Mrpl41	14.1%	5.2%	-8.9	12751
LOC100911130	14.1%	5.2%	-8.9	12752
Stim1	14.1%	5.2%	-8.9	12753
Cct6a	18.0%	9.1%	-8.9	12754
Mrps17	13.7%	4.8%	-8.9	12755
Tmem230	13.7%	4.8%	-8.9	12756
Ddx47	13.7%	4.8%	-8.9	12757
Syap1	17.6%	8.7%	-8.9	12758
Nek6	12.9%	4.0%	-8.9	12759
Ankrd17	12.9%	4.0%	-8.9	12760
Mrpl38	29.4%	20.5%	-8.9	12761
Usmg5	33.3%	24.4%	-9.0	12762
Phf10	12.2%	3.2%	-9.0	12763
Hikeshi	16.1%	7.1%	-9.0	12764
Gufl	16.1%	7.1%	-9.0	12765
Mfge8	15.7%	6.7%	-9.0	12766
Met	15.7%	6.7%	-9.0	12767
Aftph	11.4%	2.4%	-9.0	12768
Fbxo34	11.0%	2.0%	-9.0	12769
Napg	13.3%	4.3%	-9.0	12770
Mrps21	21.6%	12.5%	-9.0	12771
Srsf6	17.3%	8.2%	-9.0	12772
Qars	12.9%	3.9%	-9.0	12773
Zdhhc9	12.9%	3.9%	-9.0	12774
Arhgef16	21.2%	12.2%	-9.0	12775
Cox19	16.9%	7.8%	-9.0	12776
Wdr77	12.5%	3.5%	-9.0	12777
Tial1	12.5%	3.5%	-9.0	12778
Tpt1	100.0%	91.0%	-9.0	12779
Tm9sf1	12.2%	3.1%	-9.0	12780
Sgpl1	11.8%	2.7%	-9.0	12781
Tbc1d15	15.7%	6.6%	-9.1	12782
Galnt1	15.3%	6.2%	-9.1	12783
Lingo4	13.7%	4.6%	-9.1	12784
Pign	13.3%	4.2%	-9.1	12785
Yaf2	12.5%	3.4%	-9.1	12786
Fxr2	12.2%	3.0%	-9.1	12787
Als2	12.2%	3.0%	-9.1	12788
Pgd	24.3%	15.2%	-9.1	12789
Ampd2	11.8%	2.6%	-9.1	12790
Pdgfa	11.8%	2.6%	-9.1	12791
Mthfs	19.6%	10.5%	-9.1	12792
Akap17a	48.6%	39.5%	-9.1	12793
LOC100911160	10.6%	1.4%	-9.1	12794
Rlim	14.5%	5.4%	-9.2	12795
Tmem127	14.1%	5.0%	-9.2	12796
Hgsnat	13.7%	4.6%	-9.2	12797
Mrpl28	22.0%	12.8%	-9.2	12798

Eloc	22.0%	12.8%	-9.2	12799
Tmem183a	25.9%	16.7%	-9.2	12800
Pno1	13.3%	4.2%	-9.2	12801
Yeats4	21.6%	12.4%	-9.2	12802
Tox4	12.9%	3.8%	-9.2	12803
Gpr89b	16.9%	7.7%	-9.2	12804
LOC103694902	25.1%	15.9%	-9.2	12805
Usp24	12.5%	3.4%	-9.2	12806
Ppp2r2d	16.5%	7.3%	-9.2	12807
Rmdn1	16.5%	7.3%	-9.2	12808
Unc45a	12.2%	3.0%	-9.2	12809
Abcb6	11.4%	2.2%	-9.2	12810
Ahcyl1	15.3%	6.1%	-9.2	12811
Irf6	15.3%	6.1%	-9.2	12812
Nsun2	19.2%	10.0%	-9.2	12813
Nxf1	14.1%	4.9%	-9.2	12814
Adprhl2	13.7%	4.5%	-9.2	12815
Rnf187	17.6%	8.4%	-9.3	12816
Rnf5	21.6%	12.3%	-9.3	12817
Clns1a	12.9%	3.7%	-9.3	12818
Slc2a9	12.5%	3.3%	-9.3	12819
Phax	12.5%	3.3%	-9.3	12820
Supv3l1	12.2%	2.9%	-9.3	12821
LOC297568	99.6%	90.3%	-9.3	12822
RGD1304567	16.1%	6.8%	-9.3	12823
Tspyl1	16.1%	6.8%	-9.3	12824
H3f3c	45.1%	35.8%	-9.3	12825
Jagn1	14.9%	5.6%	-9.3	12826
Rpa3	14.9%	5.6%	-9.3	12827
LOC102557039	23.1%	13.8%	-9.3	12828
Ptrh2	31.4%	22.1%	-9.3	12829
Cebpz	14.1%	4.8%	-9.3	12830
Preb	18.0%	8.7%	-9.3	12831
Orai3	13.7%	4.4%	-9.3	12832
Mterf4	13.3%	4.0%	-9.3	12833
Cox14	21.2%	11.8%	-9.3	12834
Mad2l2	12.5%	3.2%	-9.4	12835
Eif2b1	12.2%	2.8%	-9.4	12836
Pgap2	12.2%	2.8%	-9.4	12837
Dars	20.4%	11.0%	-9.4	12838
Gnb2	20.4%	11.0%	-9.4	12839
Lmna	16.1%	6.7%	-9.4	12840
Kmt5a	11.4%	2.0%	-9.4	12841
Eppk1	11.4%	2.0%	-9.4	12842
Fam213a	15.3%	5.9%	-9.4	12843
Wdr18	14.9%	5.5%	-9.4	12844
Eva1a	14.5%	5.1%	-9.4	12845
Get4	14.5%	5.1%	-9.4	12846
LOC103692829	51.8%	42.4%	-9.4	12847
Fbxo7	18.4%	9.0%	-9.4	12848
Soat2	13.7%	4.3%	-9.4	12849
Atp6v0b	21.6%	12.2%	-9.4	12850
LOC100364155	17.3%	7.8%	-9.4	12851
Kif1c	16.9%	7.4%	-9.4	12852
Tysnd1	16.9%	7.4%	-9.4	12853
Cks1b	16.9%	7.4%	-9.4	12854
Atp2c1	12.5%	3.1%	-9.4	12855
Hnrnpc	24.7%	15.3%	-9.4	12856
Irak2	12.2%	2.7%	-9.4	12857
Iars	12.2%	2.7%	-9.4	12858
Ncoa4	16.1%	6.6%	-9.4	12859
LOC100365697	20.0%	10.6%	-9.4	12860
Rpl37a-ps1	69.8%	60.4%	-9.5	12861
Dclre1a	11.4%	1.9%	-9.5	12862
Fam160b1	11.4%	1.9%	-9.5	12863
Srsf11	15.3%	5.8%	-9.5	12864

Sez6	11.0%	1.5%	-9.5	12865
Supt4h1	19.2%	9.8%	-9.5	12866
Stx8	14.9%	5.4%	-9.5	12867
Taok2	18.8%	9.4%	-9.5	12868
LOC367830	31.0%	21.5%	-9.5	12869
Timm50	18.0%	8.6%	-9.5	12870
Mrps9	18.0%	8.6%	-9.5	12871
Rpl22	26.3%	16.8%	-9.5	12872
Iws1	47.1%	37.6%	-9.5	12873
Eloa	13.3%	3.8%	-9.5	12874
Usp3	13.3%	3.8%	-9.5	12875
Ogfr	16.9%	7.4%	-9.5	12876
Mrpl3	16.9%	7.4%	-9.5	12877
Serhl2	12.5%	3.0%	-9.5	12878
Commd5	12.5%	3.0%	-9.5	12879
Def8	12.5%	3.0%	-9.5	12880
Ttpal	12.2%	2.6%	-9.5	12881
Ahr	12.2%	2.6%	-9.5	12882
Rab30	24.3%	14.8%	-9.5	12883
Rnf167	15.7%	6.2%	-9.5	12884
Scaf8	11.4%	1.8%	-9.5	12885
Gnl1	15.3%	5.8%	-9.5	12886
Ube2q1	14.9%	5.4%	-9.5	12887
Alg5	18.8%	9.3%	-9.6	12888
Capns1	22.7%	13.2%	-9.6	12889
Zc3hav1	18.4%	8.9%	-9.6	12890
LOC102554977	22.4%	12.8%	-9.6	12891
Tfe3	12.5%	3.0%	-9.6	12892
RGD1565641	20.8%	11.2%	-9.6	12893
Ccz1b	16.5%	6.9%	-9.6	12894
Fam96a	20.4%	10.8%	-9.6	12895
LOC100363521	15.7%	6.1%	-9.6	12896
Fcf1	19.6%	10.0%	-9.6	12897
Ddah1	19.6%	10.0%	-9.6	12898
Dohh	15.3%	5.7%	-9.6	12899
Morf4l2	31.8%	22.1%	-9.6	12900
Elmo3	19.2%	9.6%	-9.6	12901
Ssbp1	19.2%	9.6%	-9.6	12902
LOC684270	18.8%	9.2%	-9.6	12903
Naxd	26.7%	17.0%	-9.6	12904
Nr5a2	14.1%	4.5%	-9.6	12905
Tm9sf4	17.6%	8.0%	-9.7	12906
Sco1	13.3%	3.7%	-9.7	12907
RGD1359127	12.5%	2.9%	-9.7	12908
Mybbp1a	12.5%	2.9%	-9.7	12909
Fbxo4	12.2%	2.5%	-9.7	12910
Cgnl1	16.1%	6.4%	-9.7	12911
Mlec	16.1%	6.4%	-9.7	12912
Gfm1	15.3%	5.6%	-9.7	12913
Gnl2	15.3%	5.6%	-9.7	12914
Ralbp1	15.3%	5.6%	-9.7	12915
Acad9	15.3%	5.6%	-9.7	12916
Plpp2	14.9%	5.2%	-9.7	12917
Mat2b	14.5%	4.8%	-9.7	12918
Thnsl2	18.4%	8.7%	-9.7	12919
Kdm5a	14.1%	4.4%	-9.7	12920
D2hgdh	13.7%	4.0%	-9.7	12921
Notum	13.3%	3.6%	-9.7	12922
LOC108348180	17.3%	7.5%	-9.7	12923
Tgm2	25.5%	15.7%	-9.7	12924
Ocel1	12.5%	2.8%	-9.8	12925
Spr	20.8%	11.0%	-9.8	12926
Nhlrc2	12.2%	2.4%	-9.8	12927
Rragc	16.1%	6.3%	-9.8	12928
Pum3	15.7%	5.9%	-9.8	12929
Ssx2ip	14.9%	5.1%	-9.8	12930

Araf	14.9%	5.1%	-9.8	12931
Rbx1	52.2%	42.4%	-9.8	12932
Nipal1	14.5%	4.7%	-9.8	12933
Pdcd10	97.6%	87.8%	-9.8	12934
Cebpg	18.4%	8.6%	-9.8	12935
Kyat1	14.1%	4.3%	-9.8	12936
Llgl2	18.0%	8.2%	-9.8	12937
Mrpl44	18.0%	8.2%	-9.8	12938
Grcc10	26.3%	16.5%	-9.8	12939
Rras	17.6%	7.8%	-9.8	12940
Pkp2	13.3%	3.5%	-9.8	12941
Sesn2	13.3%	3.5%	-9.8	12942
Diaph1	17.3%	7.4%	-9.8	12943
LOC100911440	17.3%	7.4%	-9.8	12944
Sgta	25.5%	15.7%	-9.8	12945
Sirt2	20.8%	11.0%	-9.8	12946
Colgalt1	16.5%	6.6%	-9.8	12947
Brcc3	16.5%	6.6%	-9.8	12948
Orm1	99.6%	89.8%	-9.8	12949
Mvp	19.2%	9.4%	-9.9	12950
Dcun1d5	14.9%	5.0%	-9.9	12951
Tm2d1	14.9%	5.0%	-9.9	12952
Rtfdc1	18.8%	9.0%	-9.9	12953
Tcn2	18.4%	8.6%	-9.9	12954
Gphn	14.1%	4.2%	-9.9	12955
Afdn	14.1%	4.2%	-9.9	12956
Ube3c	13.7%	3.8%	-9.9	12957
Dolk	13.7%	3.8%	-9.9	12958
Surf1	13.3%	3.4%	-9.9	12959
LOC100910069	12.9%	3.0%	-9.9	12960
Esrra	12.5%	2.6%	-9.9	12961
Gltscr2	20.4%	10.5%	-9.9	12962
Fam46a	16.1%	6.2%	-9.9	12963
Tap1	20.0%	10.1%	-9.9	12964
LOC100912604	20.0%	10.1%	-9.9	12965
Slc7a2	15.7%	5.8%	-9.9	12966
Cpn2	15.3%	5.4%	-9.9	12967
Ttc39c	15.3%	5.4%	-9.9	12968
Rmnd1	18.8%	8.9%	-10.0	12969
Max	14.5%	4.6%	-10.0	12970
Usp15	47.8%	37.9%	-10.0	12971
Blvra	14.1%	4.2%	-10.0	12972
Snrnp200	13.7%	3.8%	-10.0	12973
Rnf14	17.6%	7.7%	-10.0	12974
Cog4	13.3%	3.4%	-10.0	12975
Mug2	50.6%	40.6%	-10.0	12976
Btf3l4	21.2%	11.2%	-10.0	12977
Borcs7	24.7%	14.7%	-10.0	12978
Ifnar2	16.1%	6.1%	-10.0	12979
Cox20	16.1%	6.1%	-10.0	12980
Ptgr2	15.7%	5.7%	-10.0	12981
Pomt1	11.4%	1.4%	-10.0	12982
Slc16a6	11.0%	1.0%	-10.0	12983
Tfam	14.9%	4.9%	-10.0	12984
Thoc2	14.9%	4.9%	-10.0	12985
Snapin	18.8%	8.8%	-10.0	12986
Cnot1	14.1%	4.1%	-10.0	12987
Rap1a	18.0%	8.0%	-10.0	12988
Mark3	13.7%	3.7%	-10.0	12989
Cdc42ep4	13.7%	3.7%	-10.0	12990
Slc35a1	13.7%	3.7%	-10.0	12991
Tnrc6a	13.7%	3.7%	-10.0	12992
Mrpl45	17.6%	7.6%	-10.1	12993
Sec16a	13.3%	3.3%	-10.1	12994
Usp18	12.9%	2.9%	-10.1	12995
Btbd6	12.9%	2.9%	-10.1	12996

Brix1	12.9%	2.9%	-10.1	12997
Ctsf	16.9%	6.8%	-10.1	12998
Gatc	71.0%	60.9%	-10.1	12999
Aebp2	12.5%	2.5%	-10.1	13000
Vars	12.2%	2.1%	-10.1	13001
Ttc1	20.0%	9.9%	-10.1	13002
Nat1	15.7%	5.6%	-10.1	13003
Psmd5	15.3%	5.2%	-10.1	13004
Elf1	14.9%	4.8%	-10.1	13005
Opa1	14.9%	4.8%	-10.1	13006
Klhdc3	18.8%	8.7%	-10.1	13007
Cript	18.8%	8.7%	-10.1	13008
Tubb5	18.8%	8.7%	-10.1	13009
Sephs1	14.5%	4.4%	-10.1	13010
Ubap1	14.5%	4.4%	-10.1	13011
Polr2m	18.0%	7.9%	-10.1	13012
Parp14	18.0%	7.9%	-10.1	13013
Arap1	13.7%	3.6%	-10.1	13014
Ube2f	17.6%	7.5%	-10.1	13015
Mvk	17.6%	7.5%	-10.1	13016
Ola1	16.9%	6.7%	-10.1	13017
Dot1l	12.5%	2.4%	-10.2	13018
Bscl2	16.1%	5.9%	-10.2	13019
Avpi1	16.1%	5.9%	-10.2	13020
Rbfa	16.1%	5.9%	-10.2	13021
Snrpe	24.3%	14.1%	-10.2	13022
LOC108348078	20.0%	9.8%	-10.2	13023
Selenoh	20.0%	9.8%	-10.2	13024
Bag3	20.0%	9.8%	-10.2	13025
Leprotl1	15.7%	5.5%	-10.2	13026
Ccnl1	27.8%	17.7%	-10.2	13027
Ndufa3	27.5%	17.3%	-10.2	13028
Pfdn2	23.1%	12.9%	-10.2	13029
LOC259245	10.2%	0.0%	-10.2	13030
Zc3hc1	14.1%	3.9%	-10.2	13031
Scly	26.3%	16.1%	-10.2	13032
Clk3	17.6%	7.4%	-10.2	13033
Nhp2	16.9%	6.6%	-10.2	13034
LOC102549052	25.1%	14.9%	-10.2	13035
Ppcs	25.1%	14.9%	-10.2	13036
Zfp639	12.5%	2.3%	-10.2	13037
Smad3	11.8%	1.5%	-10.2	13038
Rangap1	19.6%	9.4%	-10.3	13039
Orai1	14.9%	4.6%	-10.3	13040
Fxn	14.9%	4.6%	-10.3	13041
Sfr1	18.8%	8.6%	-10.3	13042
Tomm7	35.3%	25.0%	-10.3	13043
LOC108352066	18.4%	8.2%	-10.3	13044
Tbrg4	14.1%	3.8%	-10.3	13045
Tnfaip1	18.0%	7.8%	-10.3	13046
Elac2	13.7%	3.4%	-10.3	13047
Impa2	17.6%	7.4%	-10.3	13048
Atad3a	13.3%	3.0%	-10.3	13049
H2afj	21.6%	11.3%	-10.3	13050
Sf3b2	17.3%	7.0%	-10.3	13051
Twistnb	12.9%	2.6%	-10.3	13052
Edem3	12.9%	2.6%	-10.3	13053
RGD1310352	21.2%	10.9%	-10.3	13054
Eif1b	16.9%	6.6%	-10.3	13055
Tex2	16.5%	6.2%	-10.3	13056
Mmgt1	12.2%	1.8%	-10.3	13057
Dtymk	16.1%	5.8%	-10.3	13058
Tiparp	20.0%	9.7%	-10.3	13059
Fndc3b	15.7%	5.4%	-10.3	13060
Coa5	19.6%	9.3%	-10.3	13061
Acot7	15.3%	5.0%	-10.3	13062

Nlrp12	27.5%	17.1%	-10.3	13063
Hgs	14.9%	4.6%	-10.3	13064
Lrp11	14.9%	4.6%	-10.3	13065
Deptor	18.8%	8.5%	-10.4	13066
RGD1561590	18.4%	8.1%	-10.4	13067
Golm1	14.1%	3.8%	-10.4	13068
Ptma	22.4%	12.0%	-10.4	13069
Hacd3	18.0%	7.7%	-10.4	13070
Impa1	13.7%	3.4%	-10.4	13071
Ube2v2	17.6%	7.3%	-10.4	13072
Bet1l	13.3%	3.0%	-10.4	13073
Sema4g	17.3%	6.9%	-10.4	13074
Slc41a2	17.3%	6.9%	-10.4	13075
Bola3	16.9%	6.5%	-10.4	13076
Farp2	12.5%	2.2%	-10.4	13077
Arsa	20.4%	10.0%	-10.4	13078
Hamp	78.0%	67.6%	-10.4	13079
Coq3	19.6%	9.2%	-10.4	13080
Rnf2	15.3%	4.9%	-10.4	13081
Ociad2	19.2%	8.8%	-10.4	13082
Adsl	14.5%	4.1%	-10.4	13083
C1galt1	18.4%	8.0%	-10.4	13084
Rwdd1	14.1%	3.7%	-10.4	13085
Prkaa1	14.1%	3.7%	-10.4	13086
Abcb1a	13.7%	3.3%	-10.4	13087
Mkrn1	17.3%	6.8%	-10.5	13088
Serpina3n	100.0%	89.5%	-10.5	13089
LOC100361854	37.3%	26.8%	-10.5	13090
Nr1i2	20.4%	9.9%	-10.5	13091
Atf6	20.4%	9.9%	-10.5	13092
Blmh	15.7%	5.2%	-10.5	13093
Agxt2	19.6%	9.1%	-10.5	13094
Selenot	23.1%	12.6%	-10.5	13095
Rora	14.5%	4.0%	-10.5	13096
Tm2d3	14.5%	4.0%	-10.5	13097
Acss2	22.7%	12.2%	-10.5	13098
Parl	18.4%	7.9%	-10.5	13099
Map2k1	14.1%	3.6%	-10.5	13100
Xpc	13.7%	3.2%	-10.5	13101
Ext2	13.3%	2.8%	-10.5	13102
Dirc2	12.9%	2.4%	-10.5	13103
Snx24	16.9%	6.3%	-10.5	13104
LOC100302372	12.5%	2.0%	-10.6	13105
Cebpd	12.2%	1.6%	-10.6	13106
Cox7a2	41.2%	30.6%	-10.6	13107
Cdc37	24.3%	13.7%	-10.6	13108
Dhrsx	20.0%	9.4%	-10.6	13109
Atp9a	15.7%	5.1%	-10.6	13110
Ipo7	19.6%	9.0%	-10.6	13111
Aldoa	19.6%	9.0%	-10.6	13112
Dag1	15.3%	4.7%	-10.6	13113
Plpp5	15.3%	4.7%	-10.6	13114
Uhrf1bp1l	14.9%	4.3%	-10.6	13115
Fam104a	14.9%	4.3%	-10.6	13116
Selenok	34.5%	23.9%	-10.6	13117
Tfb2m	17.3%	6.6%	-10.6	13118
Ubqln4	12.9%	2.3%	-10.6	13119
LOC297756	42.0%	31.3%	-10.6	13120
Itgb5	16.1%	5.4%	-10.6	13121
Dnajb1	24.3%	13.7%	-10.6	13122
Tspan12	20.0%	9.4%	-10.6	13123
Pdia3	40.0%	29.3%	-10.7	13124
Id4	18.4%	7.8%	-10.7	13125
LOC103694877	39.2%	28.5%	-10.7	13126
Tex30	14.1%	3.4%	-10.7	13127
Snx13	14.1%	3.4%	-10.7	13128

Abce1	18.0%	7.4%	-10.7	13129
Mapre1	17.6%	7.0%	-10.7	13130
Tmem19	29.8%	19.1%	-10.7	13131
Klhl21	12.9%	2.2%	-10.7	13132
Sdcbp	21.2%	10.5%	-10.7	13133
Wbp2	23.5%	12.8%	-10.7	13134
Ugt2b17	40.0%	29.3%	-10.7	13135
Ptpn11	14.5%	3.8%	-10.8	13136
Wdr61	17.6%	6.9%	-10.8	13137
Pctp	17.3%	6.5%	-10.8	13138
C2cd2	12.9%	2.2%	-10.8	13139
M6pr	20.4%	9.6%	-10.8	13140
Ssb	20.4%	9.6%	-10.8	13141
Atg3	20.0%	9.2%	-10.8	13142
Chchd1	23.9%	13.1%	-10.8	13143
Grn	19.2%	8.4%	-10.8	13144
Polr2j	19.2%	8.4%	-10.8	13145
Abcf3	14.9%	4.1%	-10.8	13146
Tmem115	14.9%	4.1%	-10.8	13147
Slc40a1	17.6%	6.8%	-10.9	13148
Gpt2	21.6%	10.7%	-10.9	13149
LOC100359951	67.1%	56.2%	-10.9	13150
LOC360919	21.2%	10.3%	-10.9	13151
Ndufaf3	21.2%	10.3%	-10.9	13152
Atrn	16.9%	6.0%	-10.9	13153
Pradc1	16.9%	6.0%	-10.9	13154
Tmem134	20.8%	9.9%	-10.9	13155
Errfi1	70.6%	59.7%	-10.9	13156
Magoh	20.4%	9.5%	-10.9	13157
Rad21	20.0%	9.1%	-10.9	13158
Rnps1	20.0%	9.1%	-10.9	13159
Tcirg1	15.3%	4.4%	-10.9	13160
Igtp	27.5%	16.5%	-10.9	13161
Becn1	18.8%	7.9%	-10.9	13162
Aimp2	18.4%	7.5%	-10.9	13163
Bccip	14.1%	3.2%	-10.9	13164
Sec24d	22.4%	11.4%	-10.9	13165
Sf1	18.0%	7.1%	-10.9	13166
LOC102554284	16.5%	5.5%	-11.0	13167
Slc22a23	16.1%	5.1%	-11.0	13168
Slc39a3	15.7%	4.7%	-11.0	13169
Slc39a1	23.5%	12.5%	-11.0	13170
Zfr	18.8%	7.8%	-11.0	13171
Ccdc58	18.8%	7.8%	-11.0	13172
Ppp1r3c	31.0%	20.0%	-11.0	13173
Ccm2	18.0%	7.0%	-11.0	13174
LOC102547700	18.0%	7.0%	-11.0	13175
Vipas39	17.6%	6.6%	-11.0	13176
Sec24a	25.9%	14.9%	-11.0	13177
Bmp1	17.3%	6.2%	-11.0	13178
Gng12	16.9%	5.8%	-11.0	13179
Bcl10	16.1%	5.0%	-11.0	13180
Timm9	16.1%	5.0%	-11.0	13181
Sacm1l	20.0%	9.0%	-11.0	13182
Sbds	49.0%	38.0%	-11.0	13183
Cdkn2b	15.7%	4.6%	-11.0	13184
Sidt2	18.8%	7.8%	-11.1	13185
Ap3d1	18.8%	7.8%	-11.1	13186
Tomm22	18.8%	7.8%	-11.1	13187
Msrb2	18.8%	7.8%	-11.1	13188
Imp3	14.5%	3.4%	-11.1	13189
Cmtm8	17.6%	6.6%	-11.1	13190
Dapk1	12.9%	1.8%	-11.1	13191
Bola1	20.8%	9.7%	-11.1	13192
Smim24	20.8%	9.7%	-11.1	13193
Hax1	20.4%	9.3%	-11.1	13194

Hibch	16.1%	5.0%	-11.1	13195
Sec22a	16.1%	5.0%	-11.1	13196
Itfg1	20.0%	8.9%	-11.1	13197
Kxd1	15.7%	4.6%	-11.1	13198
Limk2	15.7%	4.6%	-11.1	13199
Cfl1	36.5%	25.3%	-11.1	13200
Tmem123	23.9%	12.8%	-11.1	13201
Fggy	19.2%	8.1%	-11.1	13202
Eif3k	27.5%	16.3%	-11.1	13203
Slc16a10	14.9%	3.8%	-11.1	13204
Ubl4a	23.1%	12.0%	-11.1	13205
Lancl1	14.5%	3.4%	-11.2	13206
LOC102554001	14.5%	3.4%	-11.2	13207
Cdkn1a	34.9%	23.7%	-11.2	13208
Baiap2l1	18.0%	6.9%	-11.2	13209
LOC108348051	13.7%	2.6%	-11.2	13210
Trappc13	17.3%	6.1%	-11.2	13211
Tmem70	33.7%	22.5%	-11.2	13212
Pyroxd2	16.5%	5.3%	-11.2	13213
Dnajc1	16.5%	5.3%	-11.2	13214
Dtnbp1	16.1%	4.9%	-11.2	13215
Ufd1l	16.1%	4.9%	-11.2	13216
Bag6	15.7%	4.5%	-11.2	13217
Ten1	23.9%	12.7%	-11.2	13218
Pigp	14.9%	3.7%	-11.2	13219
Ddx23	18.4%	7.2%	-11.2	13220
Ywhah	22.4%	11.1%	-11.2	13221
Gosr2	18.0%	6.8%	-11.2	13222
Stradb	22.0%	10.7%	-11.2	13223
Unc119b	13.3%	2.1%	-11.3	13224
Txndc11	16.5%	5.2%	-11.3	13225
Parp16	16.5%	5.2%	-11.3	13226
Efr3a	16.5%	5.2%	-11.3	13227
Fkbp11	24.3%	13.0%	-11.3	13228
Ubr3	15.7%	4.4%	-11.3	13229
Dsp	14.9%	3.6%	-11.3	13230
Psmd14	18.8%	7.5%	-11.3	13231
Mrps30	18.4%	7.1%	-11.3	13232
Tatdn2	14.1%	2.8%	-11.3	13233
Man1b1	14.1%	2.8%	-11.3	13234
Cdc37l1	17.6%	6.3%	-11.3	13235
Arl6ip4	20.8%	9.4%	-11.4	13236
Ifi30	24.7%	13.3%	-11.4	13237
Ctdsp1	28.2%	16.9%	-11.4	13238
Hfe	15.7%	4.3%	-11.4	13239
Kti12	15.3%	3.9%	-11.4	13240
Plekhb1	18.8%	7.4%	-11.4	13241
LOC103693780	18.8%	7.4%	-11.4	13242
Tsen15	14.5%	3.1%	-11.4	13243
Chchd4	13.7%	2.3%	-11.4	13244
Ube4a	17.6%	6.2%	-11.4	13245
Dnajc2	17.3%	5.8%	-11.4	13246
Cdipt	16.9%	5.4%	-11.4	13247
Otulin	20.8%	9.4%	-11.4	13248
Xpot	16.5%	5.0%	-11.4	13249
Gsta4	32.9%	21.5%	-11.4	13250
Npepl1	20.0%	8.6%	-11.4	13251
Lztfl1	36.5%	25.0%	-11.5	13252
Acacb	19.6%	8.2%	-11.5	13253
Scrn2	15.3%	3.8%	-11.5	13254
Mtfr1l	19.2%	7.8%	-11.5	13255
Rab6a	19.2%	7.8%	-11.5	13256
LOC103689983	26.7%	15.2%	-11.5	13257
Zfp281	14.1%	2.6%	-11.5	13258
Bco2	13.7%	2.2%	-11.5	13259
Atxn10	17.3%	5.8%	-11.5	13260

Loxl4	16.9%	5.4%	-11.5	13261
Trappc6a	25.1%	13.6%	-11.5	13262
Apol3	32.5%	21.0%	-11.5	13263
Apeh	19.6%	8.1%	-11.5	13264
Smim8	15.3%	3.8%	-11.5	13265
Bcl3	23.1%	11.6%	-11.5	13266
Il1r1	26.7%	15.1%	-11.6	13267
Lhpp	26.7%	15.1%	-11.6	13268
Atp1b3	18.0%	6.5%	-11.6	13269
Arhgap5	17.3%	5.7%	-11.6	13270
Timm23	29.0%	17.4%	-11.6	13271
Nucks1	45.5%	33.9%	-11.6	13272
Smarca4	15.7%	4.1%	-11.6	13273
Idh3g	19.6%	8.0%	-11.6	13274
Selenoo	19.6%	8.0%	-11.6	13275
Afg3l2	15.3%	3.7%	-11.6	13276
Mrps11	19.2%	7.6%	-11.6	13277
Tsta3	19.2%	7.6%	-11.6	13278
Usp33	14.5%	2.9%	-11.6	13279
Stub1	22.4%	10.7%	-11.6	13280
Lrpprc	22.0%	10.3%	-11.6	13281
Nop16	17.3%	5.6%	-11.7	13282
Itgb1	21.2%	9.5%	-11.7	13283
Ddit3	29.0%	17.3%	-11.7	13284
Nr2c2ap	16.5%	4.8%	-11.7	13285
Lamtor4	20.0%	8.3%	-11.7	13286
Commd9	19.6%	7.9%	-11.7	13287
Rnf185	15.3%	3.6%	-11.7	13288
Enpp1	14.9%	3.2%	-11.7	13289
Coq10b	23.1%	11.4%	-11.7	13290
Krcc1	22.7%	11.0%	-11.7	13291
Isoc1	22.0%	10.2%	-11.7	13292
Nfx1	17.6%	5.9%	-11.7	13293
Nptn	21.2%	9.4%	-11.7	13294
Yap1	16.9%	5.1%	-11.7	13295
Dek	25.1%	13.3%	-11.7	13296
Reep5	24.7%	12.9%	-11.8	13297
Srp72	20.4%	8.6%	-11.8	13298
Chd4	20.0%	8.2%	-11.8	13299
LOC100911685	23.5%	11.8%	-11.8	13300
Vps4b	19.2%	7.4%	-11.8	13301
Wasl	18.8%	7.0%	-11.8	13302
Usp9x	22.7%	11.0%	-11.8	13303
Usp39	14.1%	2.3%	-11.8	13304
RGD1564804	18.0%	6.2%	-11.8	13305
Azin1	22.0%	10.2%	-11.8	13306
LOC691083	17.6%	5.8%	-11.8	13307
Erbin	17.6%	5.8%	-11.8	13308
Rala	17.6%	5.8%	-11.8	13309
Mbd3	21.6%	9.8%	-11.8	13310
Surf4	33.3%	21.5%	-11.8	13311
Nrep	20.8%	9.0%	-11.8	13312
Dnttip2	16.5%	4.6%	-11.8	13313
Rpl17	58.0%	46.2%	-11.8	13314
Ap2m1	20.4%	8.6%	-11.8	13315
Nomo1	15.7%	3.8%	-11.8	13316
Nolc1	15.7%	3.8%	-11.8	13317
Csnk2a1	19.6%	7.8%	-11.9	13318
Copg1	19.6%	7.8%	-11.9	13319
Cyp2b2	15.3%	3.4%	-11.9	13320
Gldc	14.5%	2.6%	-11.9	13321
Lrtm2	14.5%	2.6%	-11.9	13322
Mrps18a	22.7%	10.9%	-11.9	13323
Pabpc4	18.4%	6.6%	-11.9	13324
Ccdc107	22.4%	10.5%	-11.9	13325
Cnpy2	59.2%	47.3%	-11.9	13326

Sec63	25.9%	14.0%	-11.9	13327
Nudt14	16.9%	5.0%	-11.9	13328
Dusp3	19.6%	7.7%	-11.9	13329
Smim13	14.9%	3.0%	-11.9	13330
LOC100363472	18.4%	6.5%	-12.0	13331
Rpl36a	38.4%	26.5%	-12.0	13332
Nrip1	17.3%	5.3%	-12.0	13333
Rab5al1	21.2%	9.2%	-12.0	13334
Cesl1	29.4%	17.4%	-12.0	13335
Mug1	100.0%	88.0%	-12.0	13336
Chic2	20.4%	8.4%	-12.0	13337
Sppl2b	20.4%	8.4%	-12.0	13338
Zdhhc6	20.0%	8.0%	-12.0	13339
Stk40	15.7%	3.7%	-12.0	13340
Pmpca	23.9%	11.9%	-12.0	13341
Nelfb	15.3%	3.3%	-12.0	13342
Anxa11	55.7%	43.6%	-12.0	13343
Nus1	22.0%	9.9%	-12.0	13344
Cdc123	17.6%	5.6%	-12.1	13345
Pink1	17.6%	5.6%	-12.1	13346
Nr3c1	21.2%	9.1%	-12.1	13347
Ctso	16.9%	4.8%	-12.1	13348
Gmfb	16.9%	4.8%	-12.1	13349
Pigy	20.4%	8.3%	-12.1	13350
Pdcd2	16.1%	4.0%	-12.1	13351
Apol9a	23.9%	11.8%	-12.1	13352
Trip12	19.6%	7.5%	-12.1	13353
Prdx3	27.8%	15.7%	-12.1	13354
Stt3b	15.3%	3.2%	-12.1	13355
Hbs1l	15.3%	3.2%	-12.1	13356
Lrrc23	23.5%	11.4%	-12.1	13357
RGD1305587	23.1%	11.0%	-12.1	13358
RT1-EC2	52.2%	40.0%	-12.1	13359
Arl8b	18.8%	6.7%	-12.1	13360
Tom1	18.4%	6.3%	-12.1	13361
Cltc	30.2%	18.1%	-12.1	13362
Ccar1	17.3%	5.1%	-12.1	13363
Coq8a	33.7%	21.6%	-12.1	13364
Rassf1	24.7%	12.5%	-12.2	13365
Usp47	20.4%	8.2%	-12.2	13366
Trim47	15.3%	3.1%	-12.2	13367
Pitpnb	23.5%	11.4%	-12.2	13368
Naa60	19.2%	7.0%	-12.2	13369
Bsdc1	19.2%	7.0%	-12.2	13370
Pomp	35.7%	23.5%	-12.2	13371
Mlx	22.7%	10.6%	-12.2	13372
LOC100911664	22.7%	10.6%	-12.2	13373
Dact2	14.1%	1.9%	-12.2	13374
RGD1559821	46.7%	34.5%	-12.2	13375
Lcmt1	17.3%	5.0%	-12.2	13376
Csnk2a2	16.5%	4.2%	-12.2	13377
Nutf2	20.4%	8.2%	-12.2	13378
Dnajb2	20.0%	7.8%	-12.2	13379
Mapk9	20.0%	7.8%	-12.2	13380
Slc17a4	15.7%	3.4%	-12.2	13381
P2rx4	19.2%	7.0%	-12.3	13382
Gucy1b2	14.9%	2.6%	-12.3	13383
RGD1305464	14.5%	2.2%	-12.3	13384
Irf1	36.5%	24.1%	-12.3	13385
Aaed1	23.9%	11.6%	-12.3	13386
Tra2b	23.9%	11.6%	-12.3	13387
RGD1309995	19.6%	7.3%	-12.3	13388
Sec22b	19.6%	7.3%	-12.3	13389
Sult1c2	19.6%	7.3%	-12.3	13390
Rab29	19.2%	6.9%	-12.3	13391
Gtf2a2	18.8%	6.5%	-12.3	13392

Stard13	14.5%	2.2%	-12.4	13393
Wdr82	18.0%	5.7%	-12.4	13394
Ypel2	21.6%	9.2%	-12.4	13395
RGD1566085	21.2%	8.8%	-12.4	13396
Fam20c	15.3%	2.9%	-12.4	13397
RGD1311805	14.5%	2.1%	-12.4	13398
Lims2	22.7%	10.3%	-12.4	13399
Tpd52l2	18.0%	5.6%	-12.4	13400
Casp4	21.6%	9.1%	-12.5	13401
Ctnna1	20.8%	8.3%	-12.5	13402
Nifk	20.8%	8.3%	-12.5	13403
Pklr	37.3%	24.8%	-12.5	13404
Cops6	24.3%	11.8%	-12.5	13405
Mien1	20.0%	7.5%	-12.5	13406
Abcg3l4	28.2%	15.7%	-12.5	13407
Nubp1	23.5%	11.0%	-12.5	13408
C4b	31.4%	18.9%	-12.5	13409
Ap1m1	18.8%	6.3%	-12.5	13410
LOC679739	39.6%	27.1%	-12.5	13411
Aco1	22.4%	9.8%	-12.5	13412
Tmem259	22.4%	9.8%	-12.5	13413
Ier5	30.6%	18.1%	-12.5	13414
Smu1	18.0%	5.5%	-12.5	13415
Naa15	17.6%	5.1%	-12.5	13416
Ndufa5	45.1%	32.5%	-12.6	13417
Klf3	19.2%	6.6%	-12.6	13418
Nqo2	18.8%	6.2%	-12.6	13419
Tsg101	18.8%	6.2%	-12.6	13420
Serinc3	21.6%	9.0%	-12.6	13421
Ddx58	21.6%	9.0%	-12.6	13422
Alg13	17.3%	4.6%	-12.6	13423
Dnpep	21.2%	8.6%	-12.6	13424
Mrpl54	33.3%	20.7%	-12.6	13425
Tnip1	20.8%	8.2%	-12.6	13426
Nup153	16.5%	3.8%	-12.6	13427
Sec16b	20.4%	7.8%	-12.6	13428
Mrpl21	20.0%	7.4%	-12.6	13429
Lmo4	19.2%	6.6%	-12.7	13430
Txnl4a	22.7%	10.1%	-12.7	13431
Copb1	18.4%	5.8%	-12.7	13432
LOC100911581	18.0%	5.4%	-12.7	13433
Heca	18.0%	5.4%	-12.7	13434
Trappc5	18.0%	5.4%	-12.7	13435
Ergic2	20.8%	8.1%	-12.7	13436
Akirin2	19.6%	6.9%	-12.7	13437
LOC681410	19.6%	6.9%	-12.7	13438
Banf1	23.1%	10.4%	-12.7	13439
Mlf2	31.4%	18.6%	-12.7	13440
Ppp1r2	18.8%	6.1%	-12.7	13441
Actr10	22.7%	10.0%	-12.8	13442
Maip1	18.4%	5.7%	-12.8	13443
Mdm2	18.4%	5.7%	-12.8	13444
LOC100909597	22.4%	9.6%	-12.8	13445
Idh2	22.0%	9.2%	-12.8	13446
Myc	25.9%	13.1%	-12.8	13447
Nip7	17.3%	4.5%	-12.8	13448
Tfg	29.0%	16.2%	-12.8	13449
Scyl1	16.1%	3.3%	-12.8	13450
LOC102549760	44.3%	31.5%	-12.8	13451
Cyp3a73	14.9%	2.1%	-12.8	13452
Mrpl55	23.1%	10.3%	-12.8	13453
Gstm4	22.4%	9.5%	-12.8	13454
Fam3a	17.6%	4.8%	-12.9	13455
Dalrd3	17.6%	4.8%	-12.9	13456
Evi5	21.6%	8.7%	-12.9	13457
Dkc1	21.6%	8.7%	-12.9	13458

Mtss1	21.6%	8.7%	-12.9	13459
Paics	29.8%	16.9%	-12.9	13460
Igsf5	25.1%	12.2%	-12.9	13461
Pard3	16.5%	3.6%	-12.9	13462
Ppp2r5e	16.5%	3.6%	-12.9	13463
Smim12	20.4%	7.5%	-12.9	13464
Fam8a1	20.0%	7.1%	-12.9	13465
Cited2	20.0%	7.1%	-12.9	13466
LOC100911713	28.2%	15.3%	-12.9	13467
Golph3	28.2%	15.3%	-12.9	13468
Picalm	23.9%	11.0%	-12.9	13469
Slc6a9	19.6%	6.7%	-12.9	13470
Map4	19.2%	6.3%	-12.9	13471
Wdr3	14.9%	2.0%	-12.9	13472
Sec24c	18.8%	5.9%	-12.9	13473
Maged1	22.4%	9.4%	-12.9	13474
Cand1	17.6%	4.7%	-12.9	13475
Npepps	17.3%	4.3%	-12.9	13476
Tars	37.6%	24.7%	-12.9	13477
LOC108348112	25.1%	12.2%	-12.9	13478
Ythdf3	20.8%	7.8%	-13.0	13479
Tsnax	20.4%	7.4%	-13.0	13480
Cul3	20.0%	7.0%	-13.0	13481
Fbxw2	20.0%	7.0%	-13.0	13482
Nsa2	23.9%	11.0%	-13.0	13483
Xrn2	19.6%	6.6%	-13.0	13484
Gpam	23.5%	10.6%	-13.0	13485
Uggt1	18.8%	5.8%	-13.0	13486
H2afy	27.1%	14.1%	-13.0	13487
Rab17	22.7%	9.8%	-13.0	13488
Slc39a4	22.0%	9.0%	-13.0	13489
Phf23	16.9%	3.8%	-13.0	13490
Rdh5	20.8%	7.8%	-13.0	13491
Nap1l1	20.8%	7.8%	-13.0	13492
Rnf170	16.5%	3.4%	-13.0	13493
Acsm3	15.3%	2.2%	-13.1	13494
Rnd3	22.7%	9.7%	-13.1	13495
Mgll	18.4%	5.4%	-13.1	13496
Ecsit	26.7%	13.6%	-13.1	13497
Vps37c	18.0%	5.0%	-13.1	13498
Cep95	25.5%	12.4%	-13.1	13499
Rabggta	16.9%	3.8%	-13.1	13500
LOC103691247	24.7%	11.6%	-13.1	13501
Slc25a51	20.4%	7.3%	-13.1	13502
Fam83h	15.3%	2.2%	-13.1	13503
Ppp1r15a	31.8%	18.6%	-13.1	13504
Pdcd5	27.5%	14.3%	-13.1	13505
Mgat2	22.7%	9.6%	-13.2	13506
Zfp91	22.7%	9.6%	-13.2	13507
Gna12	18.4%	5.3%	-13.2	13508
Pfdn1	26.3%	13.1%	-13.2	13509
Ccdc28a	51.0%	37.8%	-13.2	13510
Wapl	21.2%	8.0%	-13.2	13511
Vat1	25.1%	11.9%	-13.2	13512
LOC108348080	20.8%	7.6%	-13.2	13513
Mrps14	24.3%	11.1%	-13.2	13514
Eif2d	17.6%	4.4%	-13.3	13515
Skiv2l	17.6%	4.4%	-13.3	13516
Mapk6	21.6%	8.3%	-13.3	13517
Urgcp	16.9%	3.6%	-13.3	13518
Cp	100.0%	86.7%	-13.3	13519
Adipor1	23.9%	10.6%	-13.3	13520
Dctn3	19.6%	6.3%	-13.3	13521
Mrpl35	23.5%	10.2%	-13.3	13522
Ufsp2	19.2%	5.9%	-13.3	13523
Plaa	19.2%	5.9%	-13.3	13524

Ddx21	18.8%	5.5%	-13.3	13525
LOC103693189	26.7%	13.3%	-13.3	13526
Vnn1	47.5%	34.1%	-13.3	13527
Chp1	30.6%	17.3%	-13.3	13528
Son	26.3%	12.9%	-13.3	13529
Med29	22.0%	8.6%	-13.3	13530
Scap	17.6%	4.3%	-13.3	13531
Chac2	17.6%	4.3%	-13.3	13532
Bet1	29.4%	16.1%	-13.3	13533
Ube2g1	20.8%	7.4%	-13.4	13534
LOC102552549	20.4%	7.0%	-13.4	13535
Rfwd2	20.4%	7.0%	-13.4	13536
Fbxo6	24.3%	11.0%	-13.4	13537
Ttc13	20.0%	6.6%	-13.4	13538
Arih1	20.0%	6.6%	-13.4	13539
LOC103690002	23.9%	10.6%	-13.4	13540
Sumo3	31.4%	18.0%	-13.4	13541
Atad1	25.9%	12.5%	-13.4	13542
Maea	21.6%	8.2%	-13.4	13543
Brk1	25.5%	12.1%	-13.4	13544
Dnajc15	19.6%	6.2%	-13.5	13545
Arf6	27.5%	14.0%	-13.5	13546
LOC102550442	18.8%	5.4%	-13.5	13547
Mrps36	22.7%	9.3%	-13.5	13548
Hsf2	18.4%	5.0%	-13.5	13549
Sptan1	18.4%	5.0%	-13.5	13550
Cpt1a	39.2%	25.7%	-13.5	13551
Cidea	22.4%	8.9%	-13.5	13552
Keap1	18.0%	4.6%	-13.5	13553
Prkaa2	22.0%	8.5%	-13.5	13554
Lamp1	34.1%	20.6%	-13.5	13555
LOC108351278	29.8%	16.3%	-13.5	13556
Cmtr1	17.3%	3.8%	-13.5	13557
Nrg1	16.5%	3.0%	-13.5	13558
Tpp1	23.9%	10.4%	-13.5	13559
Slc43a1	19.6%	6.1%	-13.5	13560
Mpst	27.8%	14.3%	-13.5	13561
Ntan1	23.5%	10.0%	-13.5	13562
Erbb3	18.8%	5.3%	-13.5	13563
Tmem11	22.7%	9.2%	-13.6	13564
Cgrrf1	18.4%	4.9%	-13.6	13565
Ppp2r1a	25.5%	11.9%	-13.6	13566
Ass1	100.0%	86.4%	-13.6	13567
Paqr9	16.1%	2.5%	-13.6	13568
Plekhb2	20.0%	6.4%	-13.6	13569
Atraid	20.0%	6.4%	-13.6	13570
Rplp2	52.9%	39.3%	-13.6	13571
Lamtor2	40.0%	26.4%	-13.6	13572
Swi5	30.6%	16.9%	-13.6	13573
Cenpb	18.0%	4.4%	-13.6	13574
Chka	29.8%	16.1%	-13.7	13575
LOC499644	41.6%	27.9%	-13.7	13576
Cox5a	37.3%	23.6%	-13.7	13577
LOC103691939	99.6%	85.9%	-13.7	13578
Mrps16	28.2%	14.5%	-13.7	13579
Hfe2	23.1%	9.4%	-13.7	13580
Ost4	31.0%	17.3%	-13.7	13581
Fmo1	18.4%	4.7%	-13.7	13582
Api5	22.4%	8.6%	-13.7	13583
Glmp	22.0%	8.2%	-13.7	13584
Mettl9	21.2%	7.4%	-13.7	13585
Lrrfip2	16.5%	2.7%	-13.8	13586
Acta1	99.6%	85.9%	-13.8	13587
Slc17a1	20.4%	6.6%	-13.8	13588
Ormdl2	20.4%	6.6%	-13.8	13589
Isoc2b	24.3%	10.6%	-13.8	13590

Tcp11l2	19.6%	5.8%	-13.8	13591
Ndufa10l1	23.5%	9.8%	-13.8	13592
Sept1	14.9%	1.1%	-13.8	13593
Reep6	43.9%	30.1%	-13.8	13594
Ric8a	18.8%	5.0%	-13.8	13595
Rmnd5a	21.2%	7.4%	-13.8	13596
LOC100363268	32.9%	19.1%	-13.8	13597
Cnih4	20.0%	6.2%	-13.8	13598
Cops7a	23.1%	9.3%	-13.9	13599
Ddx1	21.2%	7.3%	-13.9	13600
Ccdc124	21.2%	7.3%	-13.9	13601
Zc3h15	28.6%	14.7%	-13.9	13602
Mrpl13	23.1%	9.2%	-13.9	13603
Atg12	18.8%	4.9%	-13.9	13604
Eif4b	27.1%	13.1%	-13.9	13605
Sssca1	18.4%	4.5%	-14.0	13606
Spcs1	22.0%	8.0%	-14.0	13607
Pex5	20.4%	6.4%	-14.0	13608
LOC103694586	20.0%	6.0%	-14.0	13609
Atl2	23.9%	9.9%	-14.0	13610
Fam32a	32.2%	18.1%	-14.0	13611
LOC100363637	39.6%	25.6%	-14.0	13612
LOC100911295	35.3%	21.3%	-14.0	13613
Gng5	26.7%	12.6%	-14.0	13614
Ccl9	88.6%	74.6%	-14.0	13615
Baz1a	17.3%	3.2%	-14.1	13616
Acnat1	28.6%	14.5%	-14.1	13617
Hnf1b	16.1%	2.0%	-14.1	13618
Emc6	24.3%	10.2%	-14.1	13619
Nat6	19.6%	5.5%	-14.1	13620
Mtx1	23.5%	9.4%	-14.1	13621
Lrp1	22.7%	8.6%	-14.1	13622
Tmem126a	22.7%	8.6%	-14.1	13623
Tmem50a	26.3%	12.2%	-14.1	13624
Btd	21.6%	7.4%	-14.1	13625
Timm8b	29.8%	15.7%	-14.1	13626
Mtus1	21.2%	7.0%	-14.1	13627
Ndufa2	37.6%	23.5%	-14.1	13628
Cldn1	20.8%	6.6%	-14.1	13629
Pcmt1	20.4%	6.2%	-14.2	13630
Pla2g16	41.2%	27.0%	-14.2	13631
Pabpc1	28.2%	14.1%	-14.2	13632
Nmt1	19.6%	5.4%	-14.2	13633
Armc1	19.2%	5.0%	-14.2	13634
Eml4	18.8%	4.6%	-14.2	13635
Abcc6	18.0%	3.8%	-14.2	13636
Gspt1	26.3%	12.1%	-14.2	13637
Arfgap2	26.3%	12.1%	-14.2	13638
Rbks	25.9%	11.7%	-14.2	13639
Alg11	21.6%	7.4%	-14.2	13640
Glyatl2	29.8%	15.6%	-14.2	13641
Papola	25.1%	10.9%	-14.2	13642
Oser1	20.8%	6.6%	-14.2	13643
Capzb	29.0%	14.8%	-14.2	13644
Pcp4l1	36.9%	22.6%	-14.2	13645
Rps15al4	48.6%	34.4%	-14.3	13646
Ehmt2	19.2%	5.0%	-14.3	13647
Pmpcb	22.7%	8.5%	-14.3	13648
Ppp3r1	21.6%	7.3%	-14.3	13649
LOC100911991	83.5%	69.2%	-14.3	13650
Mospd3	25.1%	10.8%	-14.3	13651
Rrp36	20.8%	6.5%	-14.3	13652
Slc29a1	20.0%	5.7%	-14.3	13653
Atp11c	23.9%	9.6%	-14.3	13654
Prkag1	19.6%	5.3%	-14.3	13655
Kars	23.1%	8.8%	-14.3	13656

Uba2	18.8%	4.5%	-14.3	13657
Potef	72.9%	58.6%	-14.3	13658
Ibtk	22.7%	8.4%	-14.4	13659
Nab1	22.7%	8.4%	-14.4	13660
Ripk2	18.0%	3.7%	-14.4	13661
Cdc42se1	22.0%	7.6%	-14.4	13662
Ahsa1	25.5%	11.1%	-14.4	13663
Eny2	29.0%	14.6%	-14.4	13664
Ube2e1	24.7%	10.3%	-14.4	13665
Ganab	23.9%	9.5%	-14.4	13666
Chmp3	23.9%	9.5%	-14.4	13667
Smarce1	23.9%	9.5%	-14.4	13668
Arl5a	27.8%	13.4%	-14.4	13669
LOC100294508	18.4%	4.0%	-14.4	13670
Akr7a3	22.0%	7.5%	-14.4	13671
Ndufa12	42.7%	28.3%	-14.4	13672
Mapk14	29.8%	15.3%	-14.5	13673
Zadh2	21.2%	6.7%	-14.5	13674
Wdr26	21.2%	6.7%	-14.5	13675
Serpinc1	100.0%	85.5%	-14.5	13676
Uchl3	20.8%	6.3%	-14.5	13677
Hdgf	28.2%	13.7%	-14.5	13678
Ilvbl	19.6%	5.1%	-14.5	13679
Chchd7	19.2%	4.7%	-14.5	13680
Ifitm3	81.6%	67.1%	-14.5	13681
Ctr9	22.0%	7.4%	-14.5	13682
Tollip	21.6%	7.0%	-14.5	13683
Hspa8	58.4%	43.9%	-14.5	13684
Spg21	25.1%	10.6%	-14.5	13685
Slc39a7	25.1%	10.6%	-14.5	13686
Elp6	54.1%	39.6%	-14.5	13687
Ece1	20.8%	6.2%	-14.5	13688
LOC100360057	32.9%	18.4%	-14.6	13689
Acp1	32.5%	18.0%	-14.6	13690
Eif3j	23.9%	9.4%	-14.6	13691
Commd1	27.5%	12.9%	-14.6	13692
Naa38	22.7%	8.2%	-14.6	13693
Vps35	21.6%	7.0%	-14.6	13694
Isca1	25.1%	10.5%	-14.6	13695
Txndc15	28.2%	13.6%	-14.6	13696
G3bp2	23.5%	8.9%	-14.7	13697
Sirt3	23.5%	8.9%	-14.7	13698
Psmb9	43.9%	29.3%	-14.7	13699
Ppp2r5a	26.7%	12.0%	-14.7	13700
Nedd4	26.7%	12.0%	-14.7	13701
Vps29	26.3%	11.6%	-14.7	13702
Tm2d2	21.6%	6.9%	-14.7	13703
Mrpl2	29.8%	15.1%	-14.7	13704
Exosc5	20.8%	6.1%	-14.7	13705
Siah2	20.0%	5.3%	-14.7	13706
LOC100361934	28.2%	13.5%	-14.7	13707
Macrod1	27.5%	12.7%	-14.7	13708
LOC687780	59.6%	44.8%	-14.8	13709
Mrpl10	25.9%	11.1%	-14.8	13710
Sem1	37.6%	22.9%	-14.8	13711
Nbr1	25.1%	10.3%	-14.8	13712
Tor1a	20.8%	6.0%	-14.8	13713
Tmem135	24.3%	9.5%	-14.8	13714
Ap5b1	40.8%	26.0%	-14.8	13715
Hsd3b7	27.8%	13.0%	-14.8	13716
Prrg4	15.3%	0.5%	-14.8	13717
Coa3	23.1%	8.3%	-14.8	13718
Rpl4	98.0%	83.2%	-14.8	13719
Rps21	64.3%	49.5%	-14.8	13720
LOC100360843	59.6%	44.8%	-14.8	13721
Fkbp2	38.4%	23.6%	-14.9	13722

Jup	20.0%	5.1%	-14.9	13723
Erh	28.2%	13.3%	-14.9	13724
Tceal8	27.5%	12.5%	-14.9	13725
Dhx15	23.1%	8.2%	-14.9	13726
Sf3b1	23.1%	8.2%	-14.9	13727
Thrsp	89.4%	74.5%	-14.9	13728
Hint3	21.2%	6.2%	-14.9	13729
LOC103694869	25.1%	10.2%	-14.9	13730
Slc35b1	20.8%	5.8%	-14.9	13731
Sil1	24.7%	9.8%	-15.0	13732
Lpin2	20.4%	5.4%	-15.0	13733
Ttc26	57.6%	42.7%	-15.0	13734
Alcam	19.2%	4.2%	-15.0	13735
Isca2	23.1%	8.2%	-15.0	13736
Plbd1	31.4%	16.4%	-15.0	13737
Yars	22.7%	7.8%	-15.0	13738
Btg4	63.9%	48.9%	-15.0	13739
Ptbp1	33.7%	18.7%	-15.0	13740
Usp10	20.4%	5.4%	-15.0	13741
Afp	20.4%	5.4%	-15.0	13742
Ube2z	20.4%	5.4%	-15.0	13743
Nudt19	24.3%	9.3%	-15.0	13744
LOC100909548	24.3%	9.3%	-15.0	13745
Ppp1r14b	23.9%	8.9%	-15.0	13746
Kansl3	18.8%	3.8%	-15.1	13747
Erap1	21.6%	6.5%	-15.1	13748
Cox16	25.5%	10.4%	-15.1	13749
Cd59	29.4%	14.3%	-15.1	13750
Gsta1	100.0%	84.9%	-15.1	13751
Atg101	20.8%	5.7%	-15.1	13752
Rhog	20.8%	5.7%	-15.1	13753
S100a16	24.3%	9.2%	-15.1	13754
LOC681544	99.2%	84.1%	-15.1	13755
Cul1	23.9%	8.8%	-15.1	13756
Cdadc1	23.9%	8.8%	-15.1	13757
Aspg	26.7%	11.5%	-15.2	13758
Spin1	22.4%	7.2%	-15.2	13759
Cpne3	21.6%	6.4%	-15.2	13760
Srp54a	25.5%	10.3%	-15.2	13761
Cggbp1	21.2%	6.0%	-15.2	13762
Minos1	29.4%	14.2%	-15.2	13763
Clptm1	24.7%	9.5%	-15.2	13764
Mrps12	24.3%	9.1%	-15.2	13765
Ndufs7	45.1%	29.9%	-15.2	13766
LOC500956	23.9%	8.7%	-15.2	13767
Anapc11	23.5%	8.3%	-15.2	13768
Ssrp1	22.4%	7.1%	-15.2	13769
Trabd	22.0%	6.7%	-15.2	13770
Tmem47	21.6%	6.3%	-15.3	13771
Matr3	25.1%	9.8%	-15.3	13772
Desi2	20.8%	5.5%	-15.3	13773
Trim28	24.7%	9.4%	-15.3	13774
Rsrp1	20.4%	5.1%	-15.3	13775
Mif4gd	20.4%	5.1%	-15.3	13776
Plbd2	32.5%	17.3%	-15.3	13777
Fahd1	20.0%	4.7%	-15.3	13778
Kif5b	23.5%	8.2%	-15.3	13779
Galt	31.8%	16.5%	-15.3	13780
Itpa	24.7%	9.4%	-15.4	13781
MGC94207	23.9%	8.6%	-15.4	13782
Stard4	23.5%	8.2%	-15.4	13783
Erlec1	27.5%	12.1%	-15.4	13784
LOC100362384	93.3%	77.9%	-15.4	13785
Cops4	26.3%	10.9%	-15.4	13786
Ptpa	22.0%	6.6%	-15.4	13787
Selenos	22.0%	6.6%	-15.4	13788

LOC100362684	71.8%	56.4%	-15.4	13789
Sorbs2	21.6%	6.2%	-15.4	13790
Clint1	24.7%	9.3%	-15.4	13791
Ets2	45.5%	30.1%	-15.4	13792
Yae1d1	20.4%	5.0%	-15.4	13793
Gps1	23.9%	8.5%	-15.4	13794
Ap2a2	23.1%	7.7%	-15.5	13795
Etnppl	20.0%	4.5%	-15.5	13796
Sept8	15.7%	0.2%	-15.5	13797
Akap13	19.6%	4.1%	-15.5	13798
Ppp2cb	19.6%	4.1%	-15.5	13799
Ddc	23.5%	8.0%	-15.5	13800
LOC108348061	98.4%	82.9%	-15.5	13801
Fbxw5	27.5%	11.9%	-15.5	13802
Chmp2a	29.4%	13.8%	-15.6	13803
Phpt1	28.6%	13.0%	-15.6	13804
Slc38a10	20.0%	4.4%	-15.6	13805
Guk1	23.5%	7.9%	-15.6	13806
Tank	23.5%	7.9%	-15.6	13807
Arl4a	31.0%	15.3%	-15.6	13808
Irf3	26.7%	11.0%	-15.6	13809
Hectd1	22.0%	6.3%	-15.6	13810
Ranbp2	21.6%	5.9%	-15.7	13811
Cyp2f4	29.8%	14.1%	-15.7	13812
mrpl9	22.7%	7.0%	-15.7	13813
Shb	25.5%	9.8%	-15.7	13814
Fbxo3	33.7%	18.0%	-15.7	13815
Bles03	20.8%	5.0%	-15.7	13816
Tmem256	29.0%	13.3%	-15.8	13817
Sept6	16.5%	0.7%	-15.8	13818
Gtf2i	20.4%	4.6%	-15.8	13819
Ccnl2	24.3%	8.6%	-15.8	13820
LOC361985	23.5%	7.8%	-15.8	13821
Nedd4l	19.2%	3.4%	-15.8	13822
Ell	21.2%	5.4%	-15.8	13823
Yipf4	25.1%	9.3%	-15.8	13824
Zfp598	20.8%	5.0%	-15.8	13825
Alas1	32.5%	16.7%	-15.8	13826
RGD1559786	44.7%	28.9%	-15.8	13827
Asrgl1	23.5%	7.7%	-15.9	13828
Puf60	31.8%	15.9%	-15.9	13829
Cuta	26.7%	10.8%	-15.9	13830
Kdelr1	34.5%	18.6%	-15.9	13831
LOC103689931	42.7%	26.9%	-15.9	13832
Slain2	21.2%	5.3%	-15.9	13833
H6pd	21.2%	5.3%	-15.9	13834
Hsdl2	24.7%	8.8%	-15.9	13835
Slc39a14	23.9%	8.0%	-15.9	13836
Fam210b	27.8%	11.9%	-15.9	13837
Synj2bp	27.5%	11.5%	-15.9	13838
Mrps35	29.8%	13.8%	-16.0	13839
March6	25.1%	9.1%	-16.0	13840
Cnppd1	20.8%	4.8%	-16.0	13841
Mrpl12	27.8%	11.8%	-16.0	13842
Cd99	36.1%	20.1%	-16.0	13843
Erlin1	23.5%	7.5%	-16.0	13844
Clk1	31.8%	15.7%	-16.0	13845
Sqrdl	27.1%	11.0%	-16.0	13846
Larp1b	26.7%	10.6%	-16.0	13847
Mrpl32	22.4%	6.3%	-16.0	13848
Ap1ar	22.4%	6.3%	-16.0	13849
Nudt8	22.4%	6.3%	-16.0	13850
Tm6sf2	25.9%	9.8%	-16.1	13851
PVR	21.6%	5.5%	-16.1	13852
Prpf8	21.2%	5.1%	-16.1	13853
Txnrd1	25.1%	9.0%	-16.1	13854

Pigr	100.0%	83.9%	-16.1	13855
Slc20a2	20.8%	4.7%	-16.1	13856
Hsd3b5	16.1%	0.0%	-16.1	13857
Rhou	27.1%	11.0%	-16.1	13858
Mtx2	22.7%	6.6%	-16.1	13859
Lrp3	22.7%	6.6%	-16.1	13860
Elovl1	25.9%	9.8%	-16.1	13861
Hnrnpdl	25.5%	9.4%	-16.1	13862
Samm50	29.4%	13.3%	-16.1	13863
LOC100911718	25.1%	9.0%	-16.1	13864
Cltb	24.7%	8.6%	-16.2	13865
NEWGENE_1308196	28.2%	12.1%	-16.2	13866
Rps13	61.2%	45.0%	-16.2	13867
Mlc1	23.1%	7.0%	-16.2	13868
Pcsk9	23.1%	7.0%	-16.2	13869
Dpm2	22.7%	6.6%	-16.2	13870
Acad10	22.4%	6.2%	-16.2	13871
Dnajb11	25.5%	9.3%	-16.2	13872
Mipep	21.2%	5.0%	-16.2	13873
Fgfr2	20.8%	4.6%	-16.2	13874
Snrpb	29.0%	12.8%	-16.2	13875
LOC100362709	37.3%	21.0%	-16.2	13876
Dexi	20.4%	4.2%	-16.2	13877
Copa	23.5%	7.3%	-16.3	13878
Pcca	23.1%	6.9%	-16.3	13879
Ube2a	23.1%	6.9%	-16.3	13880
Ppp1cc	26.7%	10.4%	-16.3	13881
Hprt1	34.5%	18.2%	-16.3	13882
Eif4a1	55.3%	39.0%	-16.3	13883
Ppm1b	25.1%	8.8%	-16.3	13884
Clpp	28.2%	11.9%	-16.3	13885
Mrpl58	23.9%	7.6%	-16.3	13886
Fdx1l	27.5%	11.1%	-16.3	13887
Tmem33	35.7%	19.3%	-16.3	13888
Chkb	26.3%	9.9%	-16.4	13889
LOC100364138	91.4%	75.0%	-16.4	13890
Impdh2	24.7%	8.3%	-16.4	13891
RGD1564074	20.0%	3.6%	-16.4	13892
RGD1562136	23.5%	7.1%	-16.4	13893
Ostf1	23.5%	7.1%	-16.4	13894
Ddx3x	35.7%	19.3%	-16.4	13895
Rarb	22.7%	6.3%	-16.4	13896
Pgp	21.6%	5.1%	-16.5	13897
Efna1	42.4%	25.9%	-16.5	13898
Ywhaq	29.8%	13.3%	-16.5	13899
Ssna1	25.5%	9.0%	-16.5	13900
LOC108348287	50.2%	33.7%	-16.5	13901
Tmem106b	23.9%	7.4%	-16.5	13902
Tprg1l	36.1%	19.6%	-16.5	13903
Pigk	23.5%	7.0%	-16.5	13904
Rps12	77.6%	61.2%	-16.5	13905
Eif3b	27.5%	11.0%	-16.5	13906
Mrpl33	30.6%	14.1%	-16.5	13907
Rbbp7	29.8%	13.3%	-16.5	13908
LOC100361067	37.6%	21.1%	-16.5	13909
Coasy	24.7%	8.2%	-16.6	13910
Jtb	24.7%	8.2%	-16.6	13911
Bnip3	32.2%	15.6%	-16.6	13912
Apmap	31.8%	15.2%	-16.6	13913
Ndufa10	35.3%	18.7%	-16.6	13914
Amd1	22.7%	6.2%	-16.6	13915
Manf	34.9%	18.3%	-16.6	13916
Hbp1	26.3%	9.7%	-16.6	13917
Mrpl20	30.2%	13.6%	-16.6	13918
Bckdhb	34.1%	17.5%	-16.6	13919
Samd4a	20.8%	4.2%	-16.6	13920

Sec13	27.8%	11.2%	-16.7	13921
Tardbp	27.1%	10.4%	-16.7	13922
Ube2d2	27.1%	10.4%	-16.7	13923
Ybx1	47.5%	30.8%	-16.7	13924
Esrp2	21.6%	4.9%	-16.7	13925
Tmem243	24.7%	8.0%	-16.7	13926
Rrbp1	45.5%	28.8%	-16.7	13927
Stap2	32.9%	16.2%	-16.7	13928
Mrpl52	32.5%	15.8%	-16.7	13929
Gypc	23.9%	7.2%	-16.7	13930
LOC102553670	35.7%	18.9%	-16.7	13931
Aes	42.4%	25.6%	-16.8	13932
Trmt112	25.5%	8.7%	-16.8	13933
LOC100361558	58.4%	41.6%	-16.8	13934
Ptov1	25.1%	8.3%	-16.8	13935
LOC102552001	18.0%	1.2%	-16.8	13936
Ifngr2	25.5%	8.6%	-16.9	13937
Acadsb	29.0%	12.2%	-16.9	13938
Dbi	99.6%	82.7%	-16.9	13939
Mrpl37	28.6%	11.8%	-16.9	13940
Comtd1	24.3%	7.4%	-16.9	13941
H3f3b	99.2%	82.3%	-16.9	13942
Sqle	28.2%	11.4%	-16.9	13943
Grsf1	23.9%	7.0%	-16.9	13944
Mrps33	23.9%	7.0%	-16.9	13945
Vps25	27.8%	11.0%	-16.9	13946
Cyp2j4	23.5%	6.6%	-16.9	13947
Nop10	35.7%	18.8%	-16.9	13948
LOC100360977	72.9%	56.0%	-16.9	13949
LOC100359498	47.8%	30.9%	-16.9	13950
LOC100911854	39.2%	22.3%	-16.9	13951
Dynlrb1	26.7%	9.8%	-16.9	13952
LOC108350844	26.7%	9.8%	-16.9	13953
Ppp2r2a	22.4%	5.4%	-16.9	13954
Adprm	22.0%	5.0%	-16.9	13955
Cmtm6	21.6%	4.6%	-16.9	13956
Tsr1	21.2%	4.2%	-16.9	13957
Eif3d	29.0%	12.1%	-16.9	13958
Eif4e	27.8%	10.9%	-17.0	13959
Pla2g12a	23.5%	6.6%	-17.0	13960
LOC102549783	34.9%	17.9%	-17.0	13961
Maoa	22.4%	5.4%	-17.0	13962
Sys1	30.6%	13.6%	-17.0	13963
Ppp1ca	34.5%	17.5%	-17.0	13964
Srpra	38.0%	21.0%	-17.0	13965
Traf4	21.2%	4.2%	-17.0	13966
Adss	33.3%	16.3%	-17.0	13967
Rplp1	82.4%	65.3%	-17.0	13968
Rnf19a	22.7%	5.7%	-17.1	13969
Rps27a	97.6%	80.6%	-17.1	13970
Acot13	26.3%	9.2%	-17.1	13971
Bcar1	24.7%	7.6%	-17.1	13972
Mtch1	24.7%	7.6%	-17.1	13973
Abcf1	24.3%	7.2%	-17.1	13974
Acp2	27.8%	10.7%	-17.1	13975
Utp3	23.1%	6.0%	-17.1	13976
Dnajb9	38.8%	21.7%	-17.2	13977
Eif4a3	25.9%	8.7%	-17.2	13978
Ocln	21.2%	4.0%	-17.2	13979
Atp6v1f	29.4%	12.2%	-17.2	13980
LOC100360087	99.6%	82.4%	-17.2	13981
Gmps	24.3%	7.1%	-17.2	13982
Cope	35.7%	18.5%	-17.2	13983
Net1	67.5%	50.2%	-17.3	13984
RGD735029	24.7%	7.4%	-17.3	13985
Pa2g4	24.3%	7.0%	-17.3	13986

Snrpd2	39.2%	21.9%	-17.3	13987
Fbxo31	26.3%	9.0%	-17.3	13988
Cyp51	54.9%	37.6%	-17.3	13989
Fam173a	25.5%	8.2%	-17.3	13990
Stat1	29.4%	12.1%	-17.3	13991
Rpl9	70.2%	52.8%	-17.4	13992
Gpat4	23.9%	6.6%	-17.4	13993
Rnf11	23.5%	6.2%	-17.4	13994
Etnk2	40.0%	22.6%	-17.4	13995
Prkar1a	34.9%	17.5%	-17.4	13996
Dnajb4	30.2%	12.8%	-17.4	13997
RGD1561715	29.4%	12.0%	-17.4	13998
Smim20	24.7%	7.3%	-17.4	13999
Tcea3	28.6%	11.2%	-17.4	14000
Fam188a	28.6%	11.2%	-17.4	14001
Grb2	27.8%	10.4%	-17.5	14002
Mtmr12	47.8%	30.4%	-17.5	14003
Tmem208	35.3%	17.8%	-17.5	14004
Antxr2	21.6%	4.1%	-17.5	14005
Ptpn12	21.6%	4.1%	-17.5	14006
Pafah1b1	29.4%	11.9%	-17.5	14007
Rida	100.0%	82.5%	-17.5	14008
Abhd2	24.3%	6.8%	-17.5	14009
Adck3	36.1%	18.5%	-17.5	14010
Ftl1	98.4%	80.9%	-17.5	14011
Rassf3	31.8%	14.2%	-17.5	14012
Rps26	48.2%	30.7%	-17.5	14013
Ca5a	35.3%	17.7%	-17.5	14014
Fbl	26.7%	9.1%	-17.6	14015
Hao1	29.4%	11.8%	-17.6	14016
RGD1560212	24.7%	7.1%	-17.6	14017
Slc16a12	23.9%	6.3%	-17.6	14018
Tvp23b	29.8%	12.2%	-17.7	14019
LOC100359937	37.6%	20.0%	-17.7	14020
Rap1b	36.5%	18.8%	-17.7	14021
Minpp1	23.5%	5.8%	-17.7	14022
Tspan4	31.8%	14.1%	-17.7	14023
Golga7	27.5%	9.8%	-17.7	14024
LOC100911034	27.1%	9.4%	-17.7	14025
Abca8a	25.5%	7.8%	-17.7	14026
Fuca1	33.3%	15.6%	-17.7	14027
Hnrnpk	53.3%	35.6%	-17.8	14028
Zfp706	28.2%	10.5%	-17.8	14029
Cdh2	32.2%	14.4%	-17.8	14030
Cyp4a1	27.8%	10.1%	-17.8	14031
Tomm70	26.3%	8.5%	-17.8	14032
Emc4	30.2%	12.4%	-17.8	14033
Eno1	53.3%	35.5%	-17.8	14034
Mkln1	23.5%	5.7%	-17.9	14035
Txn2	34.9%	17.0%	-17.9	14036
Mia3	25.9%	8.0%	-17.9	14037
RGD1562987	34.1%	16.2%	-17.9	14038
Stoml2	29.4%	11.5%	-17.9	14039
Serpina4	100.0%	82.1%	-17.9	14040
Adh1	99.6%	81.7%	-17.9	14041
Mrpl34	23.9%	6.0%	-17.9	14042
Abhd1	22.7%	4.8%	-17.9	14043
Higd1a	42.7%	24.8%	-18.0	14044
Fmo5	58.8%	40.8%	-18.0	14045
Ivd	37.6%	19.7%	-18.0	14046
Slc10a2	20.0%	2.0%	-18.0	14047
Rnpepl1	28.2%	10.2%	-18.0	14048
Tmem223	23.9%	5.9%	-18.0	14049
Aifm1	26.3%	8.2%	-18.0	14050
St13	38.0%	20.0%	-18.1	14051
Htra1	24.7%	6.6%	-18.1	14052

Nfkb1	22.4%	4.2%	-18.1	14053
Eif1ad	25.1%	7.0%	-18.1	14054
Triap1	24.3%	6.2%	-18.2	14055
Irgm2	45.1%	26.9%	-18.2	14056
Acaa1b	35.7%	17.5%	-18.2	14057
Eif4ebp1	27.1%	8.9%	-18.2	14058
Abcb4	30.6%	12.4%	-18.2	14059
Fnip2	26.3%	8.1%	-18.2	14060
Srsf2	34.1%	15.9%	-18.2	14061
Commd3	29.8%	11.6%	-18.2	14062
Rps10	52.9%	34.7%	-18.2	14063
Srsf3	31.0%	12.7%	-18.3	14064
Psmb8	51.0%	32.7%	-18.3	14065
Psmc2	38.4%	20.1%	-18.3	14066
Angptl4	58.0%	39.7%	-18.3	14067
Psmd13	28.6%	10.3%	-18.3	14068
Tor2a	24.3%	6.0%	-18.3	14069
LOC100363177	99.2%	80.9%	-18.3	14070
LOC100911766	36.5%	18.1%	-18.3	14071
Hnrnpu	32.2%	13.8%	-18.3	14072
Ginm1	27.8%	9.5%	-18.3	14073
Srp14	34.9%	16.5%	-18.4	14074
LOC100125368	25.5%	7.1%	-18.4	14075
U2af1	32.9%	14.5%	-18.4	14076
C1qbp	32.5%	14.1%	-18.4	14077
Cd47	36.1%	17.7%	-18.4	14078
Abhd17c	31.8%	13.3%	-18.4	14079
Sowahb	22.7%	4.3%	-18.4	14080
Rps15a	55.3%	36.9%	-18.4	14081
Cdc42ep1	25.9%	7.4%	-18.4	14082
LOC683420	28.6%	10.2%	-18.5	14083
Lysmd3	23.5%	5.0%	-18.5	14084
Rcbtb2	31.8%	13.3%	-18.5	14085
Gaa	27.1%	8.6%	-18.5	14086
Aimp1	26.7%	8.2%	-18.5	14087
Cox7a2l	33.7%	15.2%	-18.5	14088
LOC103690265	87.8%	69.3%	-18.5	14089
Mrpl17	37.6%	19.1%	-18.5	14090
Bap1	24.7%	6.2%	-18.6	14091
Cpox	28.6%	10.1%	-18.6	14092
LOC100360117	70.2%	51.6%	-18.6	14093
Rps29	45.1%	26.5%	-18.6	14094
Il13ra1	31.0%	12.4%	-18.6	14095
Eif3a	34.5%	15.9%	-18.6	14096
Reep3	25.5%	6.9%	-18.6	14097
RGD1566239	25.5%	6.9%	-18.6	14098
Cul4a	29.0%	10.4%	-18.6	14099
Degs1	26.7%	8.0%	-18.7	14100
LOC100911784	99.6%	80.9%	-18.7	14101
Tmem192	28.2%	9.5%	-18.7	14102
Slc39a8	27.8%	9.1%	-18.7	14103
RGD1562420	69.4%	50.7%	-18.7	14104
Pla2g12b	31.4%	12.6%	-18.7	14105
Nt5e	39.6%	20.9%	-18.7	14106
Ipo5	26.7%	7.9%	-18.8	14107
RGD1311345	29.8%	11.0%	-18.8	14108
RGD1565002	25.5%	6.7%	-18.8	14109
Cstb	29.0%	10.2%	-18.8	14110
Dera	31.8%	12.9%	-18.8	14111
Pex16	27.1%	8.2%	-18.8	14112
Ppa2	30.2%	11.4%	-18.8	14113
Galm	34.1%	15.3%	-18.8	14114
Stk16	29.8%	11.0%	-18.9	14115
Tmem147	25.1%	6.2%	-18.9	14116
Ctsd	45.9%	27.0%	-18.9	14117
Cdk2ap1	27.5%	8.6%	-18.9	14118

Grb14	31.4%	12.5%	-18.9	14119
Saa4	99.6%	80.7%	-19.0	14120
Rab9a	32.9%	14.0%	-19.0	14121
Ddi2	24.3%	5.4%	-19.0	14122
LOC103690099	53.3%	34.4%	-19.0	14123
Eif5b	35.3%	16.3%	-19.0	14124
Mrps23	31.0%	12.0%	-19.0	14125
Cdk2ap2	33.7%	14.7%	-19.0	14126
Ddx24	24.7%	5.7%	-19.0	14127
LOC689130	78.8%	59.8%	-19.0	14128
Gabarapl2	40.0%	20.9%	-19.1	14129
Nr1h3	35.7%	16.6%	-19.1	14130
Cnn3	30.6%	11.5%	-19.1	14131
Sra1	34.1%	15.0%	-19.1	14132
Acsl4	45.9%	26.8%	-19.1	14133
Ptpmt1	27.8%	8.7%	-19.1	14134
Psme4	27.5%	8.3%	-19.1	14135
LOC100359668	98.0%	78.9%	-19.1	14136
Ncln	31.0%	11.8%	-19.1	14137
RGD1303003	34.1%	14.9%	-19.2	14138
Cndp2	29.4%	10.2%	-19.2	14139
Ptpn21	23.1%	3.9%	-19.2	14140
Sh3glb1	29.0%	9.8%	-19.3	14141
Ssu72	32.9%	13.7%	-19.3	14142
Rela	28.6%	9.4%	-19.3	14143
Cyp2c22	99.2%	79.9%	-19.3	14144
Wdr83os	28.2%	9.0%	-19.3	14145
Ide	27.8%	8.6%	-19.3	14146
Emc10	30.6%	11.3%	-19.3	14147
Ube2k	31.4%	12.0%	-19.4	14148
Pum2	26.7%	7.3%	-19.4	14149
Hcfc1r1	38.4%	19.0%	-19.4	14150
Pck1	80.0%	60.6%	-19.4	14151
Ptprd	25.5%	6.1%	-19.4	14152
Odc1	37.6%	18.2%	-19.4	14153
Nucb1	37.3%	17.8%	-19.4	14154
Tnfaip3	37.3%	17.8%	-19.4	14155
Fkbp4	41.2%	21.7%	-19.4	14156
Rnaset2	32.5%	13.1%	-19.4	14157
Sugt1	32.2%	12.7%	-19.4	14158
Actr1b	27.8%	8.4%	-19.4	14159
Mea1	35.3%	15.8%	-19.5	14160
Tgoln2	32.9%	13.4%	-19.5	14161
Acox2	32.5%	13.0%	-19.5	14162
Marveld1	20.0%	0.5%	-19.5	14163
Eif1	40.0%	20.5%	-19.5	14164
Lactb	31.0%	11.4%	-19.5	14165
Cst3	47.5%	27.9%	-19.6	14166
Lrrfip1	22.7%	3.1%	-19.6	14167
Cops2	30.6%	11.0%	-19.6	14168
Ube2b	38.8%	19.2%	-19.6	14169
Psmc3	41.2%	21.5%	-19.7	14170
Tuba4a	35.7%	16.0%	-19.7	14171
Atp5e	63.9%	44.2%	-19.7	14172
Polr2e	34.5%	14.8%	-19.7	14173
Anxa6	34.5%	14.8%	-19.7	14174
Uso1	34.5%	14.8%	-19.7	14175
Smagp	30.2%	10.5%	-19.7	14176
Pkp4	29.4%	9.7%	-19.7	14177
Etf1	36.5%	16.7%	-19.8	14178
Ggcx	27.5%	7.7%	-19.8	14179
Hist1h4b	38.4%	18.6%	-19.8	14180
Cacybp	34.1%	14.3%	-19.8	14181
Pzp	100.0%	80.2%	-19.8	14182
Aadac	41.2%	21.3%	-19.8	14183
Rnf181	32.5%	12.7%	-19.8	14184

Ppif	28.2%	8.4%	-19.8	14185
Hnrnpl	36.5%	16.6%	-19.8	14186
Npc1	23.5%	3.7%	-19.9	14187
Capza2	35.7%	15.8%	-19.9	14188
Cdh1	31.8%	11.8%	-19.9	14189
Ddrgk1	35.7%	15.7%	-19.9	14190
Fbxo9	27.1%	7.1%	-19.9	14191
F2	100.0%	80.0%	-20.0	14192
Ufm1	32.9%	12.9%	-20.0	14193
Rps18	87.1%	67.1%	-20.0	14194
Suclg2	36.1%	16.1%	-20.0	14195
Nr1d2	26.7%	6.6%	-20.0	14196
LOC100362216	25.5%	5.4%	-20.1	14197
Rpl30	50.2%	30.1%	-20.1	14198
Sel1l	27.5%	7.4%	-20.1	14199
Rdx	39.2%	19.1%	-20.1	14200
RT1-A	67.8%	47.7%	-20.1	14201
Ostc	38.0%	17.9%	-20.1	14202
Osbpl9	28.6%	8.5%	-20.2	14203
Fermt2	28.6%	8.5%	-20.2	14204
Apoa4	94.9%	74.7%	-20.2	14205
Tmem62	28.2%	8.1%	-20.2	14206
LOC108348176	37.6%	17.4%	-20.2	14207
Dazap2	37.6%	17.4%	-20.2	14208
Lpin1	32.9%	12.7%	-20.2	14209
Rabggtb	36.1%	15.8%	-20.3	14210
Atxn7l3b	34.5%	14.2%	-20.3	14211
Glo1	33.7%	13.4%	-20.3	14212
Mgat4b	32.9%	12.6%	-20.3	14213
LOC100909441	32.5%	12.2%	-20.3	14214
Pcbp2	39.2%	18.9%	-20.4	14215
LOC684871	34.5%	14.1%	-20.4	14216
Slc33a1	38.0%	17.7%	-20.4	14217
Ergic3	30.6%	10.2%	-20.4	14218
Ldha	100.0%	79.5%	-20.5	14219
Igf1	100.0%	79.5%	-20.5	14220
Cldn2	37.3%	16.8%	-20.5	14221
Caprin1	32.5%	12.1%	-20.5	14222
Sypl1	32.2%	11.7%	-20.5	14223
Kcmf1	27.8%	7.4%	-20.5	14224
Coq9	31.4%	10.9%	-20.5	14225
Entpd5	34.1%	13.6%	-20.5	14226
Ddost	29.8%	9.3%	-20.5	14227
Zfand6	33.3%	12.8%	-20.5	14228
Rock1	29.0%	8.5%	-20.5	14229
Gas2	36.5%	15.9%	-20.6	14230
RGD1559960	36.5%	15.9%	-20.6	14231
Naprt	36.1%	15.5%	-20.6	14232
Rpsa	96.1%	75.5%	-20.6	14233
Atp2a2	28.6%	8.0%	-20.6	14234
Tcea1	32.5%	11.9%	-20.6	14235
Man2a1	28.2%	7.6%	-20.6	14236
Rbpms	28.2%	7.6%	-20.6	14237
Ak3	36.1%	15.4%	-20.7	14238
Mrps24	27.5%	6.8%	-20.7	14239
Acy1	35.7%	15.0%	-20.7	14240
Slc3a2	26.3%	5.6%	-20.7	14241
Optn	38.0%	17.3%	-20.7	14242
Arcn1	37.6%	16.9%	-20.7	14243
Hacd2	27.1%	6.3%	-20.7	14244
LOC100911558	29.4%	8.6%	-20.8	14245
Cops8	29.0%	8.2%	-20.8	14246
G6pc	69.8%	49.0%	-20.8	14247
Ndufb7	56.1%	35.3%	-20.8	14248
Ppan	26.7%	5.8%	-20.8	14249
Dhrs3	30.6%	9.8%	-20.8	14250

Cacul1	29.4%	8.6%	-20.9	14251
Tuba1b	37.3%	16.4%	-20.9	14252
Psmc5	36.9%	16.0%	-20.9	14253
LOC100362479	52.9%	32.1%	-20.9	14254
Vps28	31.8%	10.9%	-20.9	14255
Pex13	27.5%	6.6%	-20.9	14256
G3bp1	30.6%	9.7%	-20.9	14257
Tmcc3	26.3%	5.4%	-20.9	14258
Sult1c2a	75.3%	54.4%	-20.9	14259
Psmg1	32.9%	12.0%	-21.0	14260
Calr	86.3%	65.3%	-21.0	14261
LOC100360413	90.2%	69.2%	-21.0	14262
Fxyd1	35.7%	14.7%	-21.0	14263
Slc25a5	43.9%	22.9%	-21.0	14264
Rab1a	51.4%	30.4%	-21.0	14265
Hsd17b2	30.2%	9.2%	-21.0	14266
Rab10	29.8%	8.8%	-21.0	14267
Slc16a7	28.2%	7.2%	-21.0	14268
Ypel5	28.2%	7.2%	-21.0	14269
Cfl2	31.8%	10.7%	-21.1	14270
Pdha1	39.2%	18.1%	-21.1	14271
F5	26.3%	5.2%	-21.1	14272
Dnajc19	42.7%	21.7%	-21.1	14273
Tgfbr2	29.0%	7.9%	-21.1	14274
Arf5	45.1%	24.0%	-21.1	14275
Pabpn1	29.8%	8.6%	-21.2	14276
LOC108348062	41.2%	20.0%	-21.2	14277
Cox8a	70.2%	49.0%	-21.2	14278
Csnk1d	32.2%	11.0%	-21.2	14279
Uba52	98.4%	77.2%	-21.2	14280
Pfdn6	31.4%	10.2%	-21.2	14281
Psmd2	31.4%	10.2%	-21.2	14282
Glyatl1	43.5%	22.3%	-21.2	14283
Scarb1	29.0%	7.8%	-21.3	14284
Nol7	32.9%	11.7%	-21.3	14285
Sin3b	32.9%	11.7%	-21.3	14286
Cr1l	27.8%	6.6%	-21.3	14287
Ssr1	36.1%	14.8%	-21.3	14288
Rnf13	31.4%	10.1%	-21.3	14289
Lgals8	31.4%	10.1%	-21.3	14290
Pxdc1	35.3%	14.0%	-21.3	14291
Rpl35	96.9%	75.5%	-21.3	14292
Pdcd6	29.0%	7.7%	-21.3	14293
Mvb12a	37.3%	15.9%	-21.3	14294
LOC680121	61.6%	40.2%	-21.4	14295
Cct8	36.5%	15.1%	-21.4	14296
Aldh1a1	55.7%	34.3%	-21.4	14297
Pelo	84.7%	63.3%	-21.4	14298
Smoc1	29.8%	8.4%	-21.4	14299
Tor1aip2	33.7%	12.3%	-21.4	14300
Hmg20b	29.4%	8.0%	-21.4	14301
Glrx3	36.5%	15.0%	-21.4	14302
Hes6	59.6%	38.1%	-21.5	14303
Fkbp1a	42.7%	21.3%	-21.5	14304
Pold4	42.0%	20.5%	-21.5	14305
Slc38a2	45.9%	24.4%	-21.5	14306
Hist1h1c	33.3%	11.8%	-21.5	14307
Nectin3	28.6%	7.1%	-21.5	14308
Tubb2a	36.9%	15.3%	-21.5	14309
Mcee	32.5%	11.0%	-21.5	14310
Tmem38b	32.5%	11.0%	-21.5	14311
LOC102549144	30.2%	8.6%	-21.6	14312
Hmgn1	45.9%	24.3%	-21.6	14313
Eef1a1	99.6%	78.0%	-21.6	14314
Aplp2	35.7%	14.1%	-21.6	14315
Mrpl23	31.4%	9.8%	-21.6	14316

Inmt	22.0%	0.3%	-21.6	14317
Rbm42	29.0%	7.4%	-21.7	14318
Slc19a2	28.2%	6.6%	-21.7	14319
LOC100910163	32.2%	10.5%	-21.7	14320
Sap18	35.7%	14.0%	-21.7	14321
Gfer	31.0%	9.3%	-21.7	14322
Sigirr	30.2%	8.5%	-21.7	14323
Sumo2	33.3%	11.6%	-21.7	14324
Vapa	40.0%	18.2%	-21.8	14325
NEWGENE_69363	64.7%	42.9%	-21.8	14326
Nedd8	34.5%	12.7%	-21.8	14327
Fbxo30	25.1%	3.3%	-21.8	14328
Psme2	53.3%	31.5%	-21.8	14329
Ndufa7	48.6%	26.8%	-21.8	14330
RGD1564606	54.9%	33.0%	-21.9	14331
Tmem53	38.0%	16.1%	-21.9	14332
Tex264	29.4%	7.5%	-21.9	14333
Uqcrq	79.2%	57.3%	-21.9	14334
Rpl7	100.0%	78.1%	-21.9	14335
Tomm6	33.3%	11.4%	-21.9	14336
Secisbp2l	28.6%	6.7%	-21.9	14337
LOC103689968	39.6%	17.7%	-21.9	14338
Mrpl16	31.0%	9.0%	-21.9	14339
Anp32b	42.4%	20.4%	-22.0	14340
Hmgcs1	79.2%	57.2%	-22.0	14341
March5	33.3%	11.4%	-22.0	14342
Pgm1	37.3%	15.3%	-22.0	14343
Fnta	28.6%	6.6%	-22.0	14344
Eif1a	36.5%	14.5%	-22.0	14345
Tcf25	44.3%	22.3%	-22.0	14346
LOC680491	44.3%	22.3%	-22.0	14347
Nop58	31.0%	9.0%	-22.0	14348
Slc34a2	30.2%	8.2%	-22.0	14349
Peli1	38.0%	16.0%	-22.1	14350
Ugt2b10	32.9%	10.9%	-22.1	14351
Hspe1	53.7%	31.7%	-22.1	14352
Ddb1	32.2%	10.1%	-22.1	14353
Nipsnap3b	38.0%	15.9%	-22.1	14354
Cox6c	70.6%	48.4%	-22.1	14355
Gss	31.8%	9.6%	-22.2	14356
Tgif1	31.0%	8.8%	-22.2	14357
Hspb1	26.3%	4.1%	-22.2	14358
Mrps15	30.2%	8.0%	-22.2	14359
Slc17a3	41.6%	19.3%	-22.2	14360
Mrps25	34.5%	12.2%	-22.3	14361
Fahd2a	45.5%	23.2%	-22.3	14362
Fcgrt	36.9%	14.5%	-22.3	14363
Bnip3l	32.5%	10.2%	-22.3	14364
Slco1b2	64.7%	42.4%	-22.3	14365
Ptgr1	47.8%	25.5%	-22.3	14366
Tex261	35.3%	12.9%	-22.3	14367
Nop56	30.2%	7.8%	-22.4	14368
LOC108349002	67.5%	45.1%	-22.4	14369
Gnai3	31.4%	9.0%	-22.4	14370
Dnaja2	35.3%	12.9%	-22.4	14371
Pccb	39.2%	16.8%	-22.4	14372
Fat1	26.7%	4.2%	-22.4	14373
Pqlc1	34.1%	11.7%	-22.4	14374
Rpl36al	58.0%	35.6%	-22.5	14375
Cyp4f4	30.6%	8.1%	-22.5	14376
Dusp5	33.7%	11.2%	-22.5	14377
Hpd	99.6%	77.1%	-22.5	14378
Mrps34	32.5%	10.0%	-22.6	14379
Il4r	32.2%	9.6%	-22.6	14380
Hint2	51.0%	28.4%	-22.6	14381
Edem1	42.0%	19.3%	-22.6	14382

Nrbp1	32.2%	9.5%	-22.6	14383
LOC100360522	98.4%	75.8%	-22.7	14384
Tmem248	40.0%	17.3%	-22.7	14385
Mif	56.5%	33.8%	-22.7	14386
LOC100360679	97.6%	75.0%	-22.7	14387
Prg4	26.3%	3.6%	-22.7	14388
Med28	34.1%	11.4%	-22.7	14389
Ephx2	29.0%	6.3%	-22.7	14390
Tcp1	37.3%	14.5%	-22.7	14391
LOC100360508	51.8%	29.0%	-22.7	14392
Urod	34.5%	11.8%	-22.8	14393
Pnpla8	34.1%	11.4%	-22.8	14394
Oxr1	29.8%	7.0%	-22.8	14395
Vtn	100.0%	77.2%	-22.8	14396
Mthfd1	36.5%	13.7%	-22.8	14397
Gng10	40.0%	17.2%	-22.8	14398
Klf6	63.1%	40.3%	-22.8	14399
Aamp	37.6%	14.8%	-22.9	14400
Mgat1	32.9%	10.1%	-22.9	14401
Oplah	32.2%	9.3%	-22.9	14402
LOC102556623	38.8%	15.9%	-22.9	14403
Rpl32	96.9%	73.9%	-22.9	14404
Rhoa	51.0%	28.1%	-22.9	14405
Hacl1	38.0%	15.1%	-22.9	14406
Suox	33.7%	10.8%	-22.9	14407
Rnh1	37.3%	14.3%	-22.9	14408
Eif4g1	41.2%	18.2%	-23.0	14409
LOC689899	55.3%	32.3%	-23.0	14410
Glyctk	44.3%	21.3%	-23.1	14411
Ei24	40.0%	16.9%	-23.1	14412
LOC108353745	23.1%	0.1%	-23.1	14413
Txndc5	31.0%	7.9%	-23.1	14414
Lasp1	34.1%	11.0%	-23.1	14415
Uox	100.0%	76.9%	-23.1	14416
Ggct	36.5%	13.3%	-23.1	14417
Eif3h	44.7%	21.6%	-23.1	14418
Ptp4a2	38.4%	15.3%	-23.2	14419
LOC688815	42.4%	19.2%	-23.2	14420
Copb2	33.3%	10.2%	-23.2	14421
Nars	37.3%	14.1%	-23.2	14422
Prkcdbp	48.6%	25.4%	-23.2	14423
Mrps18b	36.1%	12.9%	-23.2	14424
Rpl10	80.8%	57.6%	-23.2	14425
Sumo1	34.9%	11.7%	-23.2	14426
Cib1	34.5%	11.3%	-23.2	14427
LOC100360205	34.5%	11.3%	-23.2	14428
Clptm1l	34.1%	10.9%	-23.2	14429
Gstm1	100.0%	76.7%	-23.3	14430
Gstm2	100.0%	76.7%	-23.3	14431
Rpl31	81.2%	57.9%	-23.3	14432
Trap1	42.7%	19.4%	-23.3	14433
Dram2	34.1%	10.8%	-23.3	14434
Cct2	34.1%	10.8%	-23.3	14435
Cct7	38.0%	14.7%	-23.3	14436
Mrps7	42.0%	18.6%	-23.3	14437
Scp2	100.0%	76.7%	-23.3	14438
Shmt2	41.6%	18.2%	-23.3	14439
Prkcsh	40.0%	16.6%	-23.4	14440
Mars	33.7%	10.3%	-23.4	14441
Aen	29.4%	6.0%	-23.4	14442
Ethe1	45.9%	22.5%	-23.4	14443
Tmem97	40.8%	17.3%	-23.4	14444
Dpp4	38.8%	15.3%	-23.5	14445
LOC100362432	45.5%	22.0%	-23.5	14446
Rbm8a	36.5%	12.9%	-23.5	14447
Cnih1	35.3%	11.8%	-23.5	14448

Aasdhppt	34.5%	11.0%	-23.6	14449
Ldhd	29.8%	6.2%	-23.6	14450
Tat	32.2%	8.6%	-23.6	14451
LOC100910768	98.0%	74.4%	-23.6	14452
LOC103689999	39.6%	16.0%	-23.6	14453
Myh4	47.8%	24.2%	-23.6	14454
Psmd1	38.0%	14.4%	-23.7	14455
Csrp1	42.0%	18.3%	-23.7	14456
Hmgcr	37.3%	13.6%	-23.7	14457
C4bpb	99.2%	75.5%	-23.7	14458
Napa	36.1%	12.4%	-23.7	14459
Herc4	31.4%	7.7%	-23.7	14460
Tuba1c	39.2%	15.5%	-23.7	14461
Sh2d4a	30.2%	6.5%	-23.7	14462
Klhl24	31.8%	8.0%	-23.8	14463
Gsta5	99.6%	75.8%	-23.8	14464
Morf4l1	49.4%	25.6%	-23.8	14465
Tmed9	42.0%	18.1%	-23.9	14466
Nupr1	70.6%	46.7%	-23.9	14467
Cmas	40.8%	16.9%	-23.9	14468
Ivns1abp	35.7%	11.8%	-23.9	14469
Dctn2	42.4%	18.4%	-24.0	14470
Lmbrd1	36.1%	12.1%	-24.0	14471
Erp44	37.3%	13.2%	-24.1	14472
Gchfr	41.2%	17.1%	-24.1	14473
Atp5j2	82.7%	58.7%	-24.1	14474
Taf9	30.6%	6.5%	-24.1	14475
Cab39	34.1%	10.0%	-24.1	14476
Polg	33.3%	9.2%	-24.1	14477
Strap	33.3%	9.2%	-24.1	14478
Chdh	31.4%	7.2%	-24.2	14479
LOC100360781	60.4%	36.2%	-24.2	14480
Fam162a	56.1%	31.9%	-24.2	14481
Rps24	89.0%	64.8%	-24.2	14482
Hadha	43.1%	18.9%	-24.2	14483
Enpp3	34.5%	10.3%	-24.2	14484
Pdcd6ip	35.3%	11.0%	-24.3	14485
Ext1	31.0%	6.7%	-24.3	14486
Ubxn1	39.2%	14.9%	-24.3	14487
Miox	34.1%	9.8%	-24.3	14488
Tp53inp2	42.4%	18.1%	-24.3	14489
Nnt	33.7%	9.4%	-24.3	14490
Cpped1	33.7%	9.4%	-24.3	14491
Acat1	41.6%	17.3%	-24.3	14492
Xbp1	49.4%	25.1%	-24.3	14493
Mpdu1	38.8%	14.5%	-24.4	14494
Psmd7	42.0%	17.6%	-24.4	14495
Erp29	49.4%	24.9%	-24.5	14496
Gys2	36.9%	12.4%	-24.5	14497
Eif4a2	55.3%	30.8%	-24.5	14498
Tmco1	42.0%	17.4%	-24.5	14499
Palmd	37.6%	13.1%	-24.5	14500
Abtb2	29.0%	4.5%	-24.5	14501
LOC100360573	94.1%	69.5%	-24.6	14502
Arpc3	38.4%	13.8%	-24.6	14503
Spp2	100.0%	75.4%	-24.6	14504
Ubald2	28.2%	3.6%	-24.6	14505
RGD1310587	43.9%	19.3%	-24.7	14506
Eps8l2	30.2%	5.5%	-24.7	14507
Ppp1cb	38.4%	13.7%	-24.7	14508
Acot12	32.2%	7.4%	-24.7	14509
Pcbp1	44.3%	19.6%	-24.7	14510
Rars	31.8%	7.0%	-24.7	14511
LOC102555453	85.5%	60.8%	-24.7	14512
Tmem167a	34.9%	10.2%	-24.8	14513
Prpsap1	34.9%	10.2%	-24.8	14514

Arpc5	42.7%	18.0%	-24.8	14515
Ndufb4	42.7%	18.0%	-24.8	14516
Mbl2	46.7%	21.9%	-24.8	14517
Acnat2	40.4%	15.6%	-24.8	14518
Ndufb2	56.9%	32.1%	-24.8	14519
Paip2	47.5%	22.6%	-24.8	14520
Hmgcl	46.7%	21.8%	-24.8	14521
Actg1	87.1%	62.2%	-24.9	14522
Gnl3	36.9%	12.0%	-24.9	14523
Echdc2	36.9%	12.0%	-24.9	14524
Idi1	48.6%	23.7%	-24.9	14525
Znrf2	36.1%	11.2%	-24.9	14526
Psmd3	35.7%	10.8%	-24.9	14527
Mrpl27	43.9%	19.0%	-24.9	14528
Plg	100.0%	75.1%	-24.9	14529
LOC100912578	54.1%	29.2%	-24.9	14530
Npc2	45.1%	20.1%	-25.0	14531
Dpm1	39.2%	14.2%	-25.0	14532
LOC108349548	39.2%	14.2%	-25.0	14533
Glrx5	47.5%	22.5%	-25.0	14534
LOC688932	63.9%	38.9%	-25.0	14535
Rps17	74.5%	49.5%	-25.0	14536
Rheb	41.2%	16.1%	-25.0	14537
Rps6	82.4%	57.3%	-25.0	14538
Rps16	90.6%	65.5%	-25.0	14539
Eef1d	44.7%	19.7%	-25.0	14540
Myl12b	40.4%	15.3%	-25.0	14541
Saraf	40.4%	15.3%	-25.1	14542
Sdf2	36.1%	11.0%	-25.1	14543
Man1a1	44.3%	19.2%	-25.1	14544
Vamp7	43.5%	18.3%	-25.2	14545
Msra	34.9%	9.7%	-25.2	14546
Tmem160	38.4%	13.2%	-25.2	14547
Thoc7	76.9%	51.6%	-25.3	14548
LOC103694903	48.6%	23.3%	-25.4	14549
LOC684988	76.1%	50.7%	-25.4	14550
H2afz	42.7%	17.3%	-25.4	14551
Apob	100.0%	74.6%	-25.4	14552
Crot	49.8%	24.4%	-25.4	14553
Aurkaip1	36.9%	11.4%	-25.4	14554
Pdlim1	34.1%	8.6%	-25.5	14555
Tpst1	32.9%	7.4%	-25.5	14556
Ndufs4	41.2%	15.7%	-25.5	14557
Mpnd	45.1%	19.6%	-25.5	14558
Dld	49.0%	23.5%	-25.5	14559
Ppp4r2	36.1%	10.6%	-25.5	14560
Os9	42.4%	16.8%	-25.6	14561
Chchd2	82.4%	56.8%	-25.6	14562
Fdx1	65.5%	39.9%	-25.6	14563
Ugdh	40.0%	14.4%	-25.6	14564
Yipf3	34.9%	9.3%	-25.6	14565
Prdx6	38.8%	13.2%	-25.6	14566
Tfrc	33.7%	8.1%	-25.7	14567
Nucb2	37.3%	11.6%	-25.7	14568
Nadk	40.8%	15.1%	-25.7	14569
Pnrc1	61.2%	35.5%	-25.7	14570
Hras	34.9%	9.2%	-25.7	14571
Tomm20	40.4%	14.6%	-25.8	14572
Lman2	43.9%	18.1%	-25.8	14573
Psma3	51.8%	26.0%	-25.8	14574
Eef2	71.8%	46.0%	-25.8	14575
LOC690171	83.9%	58.1%	-25.8	14576
Ptges3	53.7%	27.9%	-25.8	14577
Cpt2	36.9%	11.0%	-25.8	14578
Lypla1	39.2%	13.3%	-25.9	14579
Tpmt	42.4%	16.5%	-25.9	14580

Atf5	71.0%	45.1%	-25.9	14581
Gstm7	37.3%	11.4%	-25.9	14582
Arpc5l	37.3%	11.4%	-25.9	14583
Ppp2ca	49.0%	23.1%	-25.9	14584
Slc6a13	36.9%	10.9%	-26.0	14585
Pglyrp2	40.8%	14.8%	-26.0	14586
Bckdha	47.8%	21.8%	-26.0	14587
LOC100911575	68.2%	42.2%	-26.0	14588
Zyx	36.5%	10.4%	-26.1	14589
Mrpl18	36.5%	10.4%	-26.1	14590
Stbd1	44.7%	18.6%	-26.1	14591
Gnpnat1	40.0%	13.9%	-26.1	14592
Mttp	42.4%	16.2%	-26.1	14593
Rpl12	90.2%	64.0%	-26.2	14594
Ces2a	47.8%	21.7%	-26.2	14595
Man2b1	38.8%	12.6%	-26.2	14596
Psma2	45.9%	19.7%	-26.2	14597
Ptprf	36.9%	10.6%	-26.2	14598
Nit2	43.9%	17.7%	-26.3	14599
Psmd11	43.5%	17.3%	-26.3	14600
Nsfl1c	34.9%	8.6%	-26.3	14601
Nfkbia	67.8%	41.6%	-26.3	14602
Cops5	42.7%	16.5%	-26.3	14603
Apopt1	34.1%	7.8%	-26.3	14604
Slco2b1	33.3%	7.0%	-26.3	14605
Immt	36.5%	10.2%	-26.3	14606
LOC100911847	93.7%	67.4%	-26.3	14607
Ccser2	34.1%	7.8%	-26.4	14608
Rab11a	42.0%	15.6%	-26.4	14609
Tmem238	44.7%	18.3%	-26.4	14610
Etfdh	44.3%	17.9%	-26.4	14611
Icam1	48.2%	21.8%	-26.4	14612
RGD1309676	40.4%	13.9%	-26.5	14613
LOC689955	68.6%	42.1%	-26.5	14614
Oat	42.7%	16.2%	-26.5	14615
Derl2	46.7%	20.1%	-26.5	14616
Cyp39a1	45.1%	18.5%	-26.6	14617
LOC100912115	42.7%	16.1%	-26.6	14618
Fdps	96.9%	70.3%	-26.6	14619
Nnmt	38.0%	11.4%	-26.6	14620
Rnf139	33.3%	6.7%	-26.6	14621
Sult1a1	99.6%	73.0%	-26.6	14622
Ubb	99.6%	73.0%	-26.6	14623
RGD1561736	82.7%	56.1%	-26.6	14624
Ggnbp2	40.8%	14.1%	-26.6	14625
Rps23	98.4%	71.8%	-26.6	14626
Cct5	60.8%	34.1%	-26.7	14627
RGD1562690	80.4%	53.7%	-26.7	14628
Crem	49.8%	23.1%	-26.7	14629
C9	99.6%	72.9%	-26.7	14630
Acy3	32.9%	6.2%	-26.7	14631
Tecr	53.7%	27.0%	-26.7	14632
Cox7a2l2	65.9%	39.2%	-26.7	14633
LOC100361008	53.3%	26.6%	-26.7	14634
Arf1	57.3%	30.5%	-26.7	14635
Rpl27a	81.6%	54.8%	-26.7	14636
LOC100909929	71.8%	45.0%	-26.8	14637
P2ry2	38.0%	11.3%	-26.8	14638
Snx3	74.5%	47.7%	-26.8	14639
Rps3a	89.0%	62.2%	-26.8	14640
Pnpla7	34.5%	7.7%	-26.8	14641
Pdia5	38.0%	11.2%	-26.8	14642
Eif6	45.9%	19.0%	-26.9	14643
Cth	93.3%	66.4%	-26.9	14644
Cd63	43.1%	16.2%	-26.9	14645
Got2	58.8%	31.9%	-26.9	14646

Bud31	37.3%	10.3%	-26.9	14647
Cdo1	99.6%	72.7%	-26.9	14648
LOC100911186	61.2%	34.2%	-27.0	14649
Mrpl22	39.2%	12.2%	-27.0	14650
Xylb	42.4%	15.3%	-27.0	14651
Psmb2	47.8%	20.8%	-27.1	14652
Prodh1	38.8%	11.8%	-27.1	14653
Ndufc2	42.7%	15.7%	-27.1	14654
Lman1	38.4%	11.4%	-27.1	14655
Ube2l3	46.7%	19.6%	-27.1	14656
Yipf5	38.0%	11.0%	-27.1	14657
Ier3	62.7%	35.7%	-27.1	14658
Rps19	87.5%	60.4%	-27.1	14659
Ubxn4	41.6%	14.5%	-27.1	14660
Dynll2	53.3%	26.2%	-27.1	14661
Iqgap2	43.1%	16.0%	-27.2	14662
Atp6v1b2	38.8%	11.7%	-27.2	14663
Krtcap2	45.9%	18.7%	-27.2	14664
Lsr	44.3%	17.1%	-27.2	14665
Hmox1	38.8%	11.6%	-27.2	14666
Tkfc	54.5%	27.3%	-27.3	14667
Hnrnpa3	57.6%	30.4%	-27.3	14668
Nckap1	38.8%	11.5%	-27.3	14669
Rnasek	42.4%	15.0%	-27.3	14670
Slc22a8	27.5%	0.1%	-27.4	14671
Ak4	48.2%	20.9%	-27.4	14672
Sds	29.4%	2.0%	-27.4	14673
Serpina3m	99.6%	72.2%	-27.4	14674
Uqcrfs1	53.3%	25.9%	-27.4	14675
LOC100911372	85.9%	58.4%	-27.4	14676
Psmd4	42.4%	14.9%	-27.5	14677
Park7	54.5%	27.0%	-27.5	14678
Bloc1s1	44.3%	16.8%	-27.5	14679
Cyp2d4	47.1%	19.5%	-27.6	14680
Apoc2	99.6%	72.0%	-27.6	14681
Gclm	41.2%	13.6%	-27.6	14682
Abca6	36.1%	8.5%	-27.6	14683
Rab7a	69.0%	41.4%	-27.6	14684
Apoc4	97.6%	69.9%	-27.7	14685
Gars	37.6%	9.9%	-27.7	14686
Uqcr11	53.3%	25.6%	-27.8	14687
Hnrnpf	45.5%	17.7%	-27.8	14688
Sucla2	44.7%	16.9%	-27.8	14689
Apoa5	98.8%	71.0%	-27.8	14690
Derl1	42.7%	14.9%	-27.9	14691
Nme3	38.0%	10.2%	-27.9	14692
Gclc	38.0%	10.2%	-27.9	14693
Lactb2	42.0%	14.1%	-27.9	14694
Phb	54.1%	26.2%	-27.9	14695
Cxadr	36.9%	9.0%	-27.9	14696
Rps2	99.2%	71.3%	-27.9	14697
Zwint	40.4%	12.5%	-27.9	14698
Chmp5	44.3%	16.4%	-27.9	14699
LOC679963	84.3%	56.3%	-28.0	14700
Psma1	42.4%	14.3%	-28.0	14701
LOC100360846	49.4%	21.3%	-28.1	14702
Hm13	44.7%	16.6%	-28.1	14703
Mrpl14	36.1%	8.0%	-28.1	14704
LOC108350502	93.7%	65.6%	-28.1	14705
Rnf130	39.2%	11.1%	-28.1	14706
Ubl3	42.4%	14.2%	-28.1	14707
Spcs2	50.6%	22.5%	-28.1	14708
Atp6v0d1	46.3%	18.1%	-28.1	14709
Nectin2	42.0%	13.8%	-28.1	14710
Fau	83.5%	55.4%	-28.1	14711
Rpl13a	91.8%	63.6%	-28.1	14712

Crip2	47.8%	19.7%	-28.2	14713
Ndufb5	51.8%	23.6%	-28.2	14714
Ndufa11	71.4%	43.2%	-28.2	14715
Hal	41.6%	13.3%	-28.3	14716
Ywhaz	45.5%	17.2%	-28.3	14717
St3gal6	43.9%	15.6%	-28.3	14718
Rpl26	98.0%	69.7%	-28.3	14719
Ndufb3	55.7%	27.3%	-28.3	14720
LOC100360449	79.6%	51.2%	-28.4	14721
Iyd	50.2%	21.8%	-28.4	14722
Dgat2	67.5%	39.0%	-28.4	14723
Dpyd	50.2%	21.7%	-28.5	14724
Spryd7	41.2%	12.6%	-28.5	14725
C4bpa	99.2%	70.7%	-28.6	14726
Adh5	50.6%	22.0%	-28.6	14727
Rdh7	100.0%	71.4%	-28.6	14728
Rhod	40.4%	11.7%	-28.7	14729
Hspb8	60.8%	32.1%	-28.7	14730
Msrb1	42.7%	14.0%	-28.8	14731
Rpl34	80.0%	51.2%	-28.8	14732
Rtcb	42.0%	13.2%	-28.8	14733
Tnfrsf1a	45.9%	17.1%	-28.8	14734
Ctbs	48.6%	19.8%	-28.8	14735
Psmd12	44.3%	15.5%	-28.8	14736
Gpx4	89.0%	60.2%	-28.8	14737
Slc17a2	37.6%	8.8%	-28.9	14738
Pon1	100.0%	71.1%	-28.9	14739
Rps4x	99.6%	70.7%	-28.9	14740
Acss3	44.7%	15.8%	-28.9	14741
Rps10l1	69.4%	40.5%	-28.9	14742
Ptp4a1	47.8%	18.9%	-28.9	14743
Dusp16	34.9%	6.0%	-28.9	14744
Hsbp1	47.1%	18.1%	-28.9	14745
Ephx1	99.6%	70.7%	-28.9	14746
Pgls	44.3%	15.3%	-29.0	14747
Bcap31	51.8%	22.8%	-29.0	14748
Ifrd1	63.9%	34.9%	-29.0	14749
LOC683961	80.8%	51.7%	-29.1	14750
Hhex	60.8%	31.7%	-29.1	14751
Aim1	34.5%	5.4%	-29.2	14752
Rpl5	94.5%	65.3%	-29.2	14753
LOC100362999	51.4%	22.1%	-29.2	14754
Vmp1	42.0%	12.7%	-29.3	14755
Mydgf	45.9%	16.6%	-29.3	14756
Tmed10	45.5%	16.2%	-29.3	14757
Cmpk1	44.3%	15.0%	-29.3	14758
Snf8	48.2%	18.9%	-29.3	14759
Eif3m	47.1%	17.7%	-29.3	14760
Ndufs8	55.3%	26.0%	-29.3	14761
Atp6v1g1	67.1%	37.7%	-29.3	14762
Rbp1	53.7%	24.4%	-29.3	14763
Bphl	39.6%	10.2%	-29.4	14764
RT1-A3	75.7%	46.3%	-29.4	14765
Psmc4	41.2%	11.8%	-29.4	14766
Pls3	44.3%	14.9%	-29.4	14767
Pipox	47.8%	18.4%	-29.5	14768
Raf1	38.0%	8.6%	-29.5	14769
Masp1	42.0%	12.5%	-29.5	14770
Arf4	50.2%	20.7%	-29.5	14771
Rnd1	65.1%	35.6%	-29.5	14772
Rpl41	82.4%	52.8%	-29.6	14773
Dhtkd1	44.3%	14.7%	-29.6	14774
Lrrc59	42.7%	13.1%	-29.6	14775
Rnase4	100.0%	70.3%	-29.7	14776
Atp6v0e1	45.1%	15.4%	-29.7	14777
RGD1560015	54.9%	25.2%	-29.7	14778

Pdk2	53.3%	23.6%	-29.8	14779
Tax1bp1	48.6%	18.9%	-29.8	14780
Slc25a10	43.5%	13.7%	-29.8	14781
LOC108348077	30.6%	0.8%	-29.8	14782
Rnf149	42.7%	12.9%	-29.8	14783
Hmgcs2	99.6%	69.8%	-29.8	14784
Krt18	98.8%	69.0%	-29.8	14785
Serpina11	64.3%	34.5%	-29.9	14786
Rgs3	47.1%	17.2%	-29.9	14787
Ociad1	44.7%	14.8%	-29.9	14788
Slc16a1	40.0%	10.1%	-29.9	14789
Gstz1	64.7%	34.8%	-29.9	14790
C6	67.5%	37.5%	-30.0	14791
Cd22	65.9%	35.9%	-30.0	14792
Rpl28	82.0%	52.0%	-30.0	14793
Ssr3	48.2%	18.2%	-30.0	14794
Atf4	94.5%	64.4%	-30.1	14795
Slc25a13	47.1%	16.9%	-30.1	14796
Ubqln1	46.7%	16.5%	-30.1	14797
Ccs	54.1%	24.0%	-30.1	14798
Ak2	57.3%	27.1%	-30.2	14799
Ppia	89.8%	59.6%	-30.2	14800
Sh3bgrl	55.7%	25.5%	-30.2	14801
Cdc34	38.0%	7.8%	-30.2	14802
Sar1a	47.8%	17.6%	-30.3	14803
Tstd1	58.0%	27.7%	-30.3	14804
Cept1	53.7%	23.4%	-30.3	14805
Aldh2	99.2%	68.9%	-30.3	14806
Timm13	52.9%	22.6%	-30.3	14807
Psenen	43.1%	12.8%	-30.3	14808
Ifi27l2b	95.7%	65.3%	-30.4	14809
Rabac1	49.0%	18.6%	-30.4	14810
Kmo	46.3%	15.8%	-30.4	14811
Lpgat1	45.5%	15.0%	-30.5	14812
Rps7	78.4%	48.0%	-30.5	14813
LOC500473	31.8%	1.3%	-30.5	14814
Arl6ip1	38.8%	8.3%	-30.5	14815
Ces2h	66.7%	36.1%	-30.5	14816
Akr1c14	95.3%	64.7%	-30.5	14817
Mal2	47.8%	17.3%	-30.6	14818
Lrp10	38.0%	7.4%	-30.6	14819
Upb1	96.1%	65.5%	-30.6	14820
Haao	62.0%	31.3%	-30.6	14821
Sult1b1	91.4%	60.7%	-30.7	14822
Yif1a	43.1%	12.4%	-30.7	14823
Abat	44.7%	13.9%	-30.8	14824
LOC100364457	81.6%	50.8%	-30.8	14825
Egfr	42.0%	11.1%	-30.8	14826
Sec62	53.7%	22.9%	-30.9	14827
Map2k2	52.2%	21.3%	-30.9	14828
Tm9sf2	43.5%	12.6%	-30.9	14829
Igfbp1	97.3%	66.3%	-30.9	14830
Hsd17b12	42.7%	11.8%	-30.9	14831
Perp	50.2%	19.3%	-30.9	14832
Jak1	45.5%	14.5%	-30.9	14833
Gm5471	56.1%	25.1%	-31.0	14834
Tmbim4	51.0%	19.9%	-31.1	14835
Smdt1	67.1%	36.0%	-31.1	14836
Papss2	44.7%	13.6%	-31.1	14837
Ugp2	49.8%	18.6%	-31.2	14838
Sec61b	58.0%	26.9%	-31.2	14839
LOC103690154	38.4%	7.2%	-31.2	14840
LOC100911252	91.8%	60.5%	-31.3	14841
Gcgr	45.9%	14.6%	-31.3	14842
Echdc3	57.3%	26.0%	-31.3	14843
Rpl29	93.7%	62.4%	-31.3	14844

Prelid1	69.4%	38.0%	-31.4	14845
Slc25a1	65.1%	33.7%	-31.4	14846
Rpl23a	74.9%	43.5%	-31.4	14847
Cat	99.6%	68.2%	-31.4	14848
Ndufs5	53.3%	21.9%	-31.4	14849
Cox4i1	89.8%	58.4%	-31.5	14850
Sardh	47.5%	16.0%	-31.5	14851
Slc25a39	51.4%	19.9%	-31.5	14852
Rpl24	96.9%	65.4%	-31.5	14853
Akr1c12	49.8%	18.3%	-31.5	14854
Cyp2b1	37.3%	5.8%	-31.5	14855
Arg1	98.8%	67.3%	-31.5	14856
Pdhb	52.9%	21.4%	-31.5	14857
Tpm1	58.4%	26.9%	-31.6	14858
Cxcl12	41.6%	10.0%	-31.6	14859
Eif3f	53.7%	22.1%	-31.6	14860
Psmc6	51.8%	20.1%	-31.6	14861
Ppdpf	55.3%	23.7%	-31.6	14862
Uqcr10	74.9%	43.2%	-31.7	14863
LOC108353446	94.9%	63.2%	-31.7	14864
Chpt1	44.7%	13.0%	-31.7	14865
Rpl21	88.2%	56.5%	-31.7	14866
Hnrnph3	96.5%	64.7%	-31.7	14867
Psmd6	42.0%	10.2%	-31.7	14868
Abcd3	46.3%	14.5%	-31.8	14869
Riok3	41.2%	9.4%	-31.8	14870
Blvrb	49.8%	17.9%	-31.9	14871
LOC100910455	95.3%	63.4%	-31.9	14872
Mpp6	49.0%	17.1%	-31.9	14873
Lpcat3	47.1%	15.1%	-32.0	14874
Prpf19	47.1%	15.1%	-32.0	14875
Sub1	59.2%	27.3%	-32.0	14876
Snrnp25	50.2%	18.2%	-32.0	14877
Anpep	43.1%	11.1%	-32.0	14878
March4	32.2%	0.1%	-32.1	14879
Cfb	100.0%	67.9%	-32.1	14880
Abcb11	54.1%	22.0%	-32.1	14881
Cct4	49.8%	17.7%	-32.1	14882
Tm7sf2	59.2%	27.0%	-32.2	14883
Ift20	50.2%	18.0%	-32.2	14884
Bri3	50.2%	18.0%	-32.2	14885
Nfkbiz	60.0%	27.7%	-32.3	14886
Rpp21	63.1%	30.9%	-32.3	14887
Idh3B	53.7%	21.4%	-32.3	14888
Ubc	99.2%	66.9%	-32.3	14889
Apcs	85.1%	52.8%	-32.3	14890
Lyc2	42.7%	10.4%	-32.4	14891
Rpl19	96.9%	64.5%	-32.4	14892
Mup4	32.9%	0.6%	-32.4	14893
Trib3	35.3%	2.9%	-32.4	14894
Proz	60.0%	27.6%	-32.4	14895
Pnkd	55.7%	23.3%	-32.4	14896
Abhd17b	46.3%	13.8%	-32.4	14897
RGD1566369	70.2%	37.7%	-32.5	14898
Hexa	49.0%	16.5%	-32.5	14899
Cast	43.9%	11.4%	-32.5	14900
Rtn3	47.5%	14.9%	-32.5	14901
Psmb3	51.0%	18.5%	-32.5	14902
Lbp	71.0%	38.4%	-32.5	14903
Nipsnap1	58.4%	25.9%	-32.5	14904
RGD1562402	90.2%	57.6%	-32.6	14905
Rpl8	94.1%	61.6%	-32.6	14906
Acad11	50.6%	18.0%	-32.6	14907
Tmed7	46.3%	13.7%	-32.6	14908
Rpl14	97.6%	65.0%	-32.7	14909
Cyp2c23	100.0%	67.3%	-32.7	14910

Nsdhl	65.5%	32.8%	-32.7	14911
LOC100362895	56.5%	23.7%	-32.8	14912
Dhcr7	47.5%	14.6%	-32.8	14913
Mbnl2	42.7%	9.9%	-32.8	14914
Ndufa8	60.8%	27.9%	-32.9	14915
Rpl23	87.8%	54.9%	-32.9	14916
Eif2s2	54.5%	21.6%	-32.9	14917
Ywhab	49.8%	16.9%	-32.9	14918
Tmem140	53.3%	20.4%	-32.9	14919
Mlycd	48.6%	15.7%	-33.0	14920
Rnf103	42.0%	9.0%	-33.0	14921
Slc22a1	45.5%	12.5%	-33.0	14922
Dnajc3	53.7%	20.7%	-33.0	14923
Cluh	45.1%	12.1%	-33.0	14924
LOC100360491	60.4%	27.3%	-33.1	14925
Akr7a2	56.1%	23.0%	-33.1	14926
Atp5b	97.6%	64.6%	-33.1	14927
Emc7	48.6%	15.5%	-33.1	14928
Btg1	52.2%	19.0%	-33.1	14929
Ssr2	50.2%	17.0%	-33.2	14930
Gjb1	99.2%	66.0%	-33.2	14931
RGD1564963	97.6%	64.4%	-33.2	14932
RGD1305807	45.5%	12.2%	-33.3	14933
Actr2	49.4%	16.1%	-33.3	14934
Psmc1	51.0%	17.7%	-33.3	14935
Tufm	52.5%	19.2%	-33.4	14936
Mrpl15	47.1%	13.7%	-33.4	14937
Aqp9	50.2%	16.7%	-33.5	14938
Cycs	72.2%	38.6%	-33.5	14939
St6gal1	62.7%	29.2%	-33.6	14940
Oaz1	83.1%	49.6%	-33.6	14941
Rpl10a	61.6%	28.0%	-33.6	14942
Gbe1	55.7%	22.1%	-33.6	14943
Ndufv3	55.3%	21.5%	-33.8	14944
Psmb6	58.0%	24.2%	-33.8	14945
Ube2r2	49.0%	15.2%	-33.8	14946
Rab2a	52.5%	18.7%	-33.8	14947
F11r	60.8%	26.9%	-33.8	14948
Qprt	54.9%	21.0%	-33.9	14949
LOC100359922	67.8%	33.9%	-34.0	14950
Dnaja1	63.5%	29.6%	-34.0	14951
Gsto1	57.3%	23.3%	-34.0	14952
Gckr	56.9%	22.9%	-34.0	14953
Akr1e2	49.4%	15.3%	-34.1	14954
Cyp2a1	94.9%	60.8%	-34.1	14955
Phb2	57.3%	23.1%	-34.2	14956
Slc25a20	52.5%	18.4%	-34.2	14957
Ndfip2	47.8%	13.7%	-34.2	14958
Cldn3	50.6%	16.4%	-34.2	14959
Acaa1a	79.6%	45.4%	-34.2	14960
Gapdh	100.0%	65.8%	-34.2	14961
Got1	47.8%	13.6%	-34.3	14962
Enpp2	43.5%	9.3%	-34.3	14963
LOC100365839	97.6%	63.4%	-34.3	14964
Dhrs4	63.1%	28.9%	-34.3	14965
Cpamd8	94.5%	60.2%	-34.3	14966
RGD1564614	47.5%	13.1%	-34.3	14967
Rps5	92.5%	58.2%	-34.4	14968
Gjb2	58.8%	24.5%	-34.4	14969
Hebp1	45.9%	11.5%	-34.4	14970
Gadd45gip1	47.1%	12.6%	-34.4	14971
Pgam1	63.9%	29.4%	-34.5	14972
Sptssa	52.5%	18.0%	-34.6	14973
Dap	67.1%	32.5%	-34.6	14974
Calm2	73.3%	38.7%	-34.6	14975
Rpl13	68.2%	33.5%	-34.7	14976

Arpc2	55.7%	20.9%	-34.7	14977
Shmt1	50.2%	15.4%	-34.8	14978
Gamt	99.2%	64.4%	-34.8	14979
Slc25a6	61.6%	26.8%	-34.8	14980
Fkbp8	63.9%	29.1%	-34.8	14981
Bzw1	51.4%	16.5%	-34.8	14982
Mettl7a	45.5%	10.6%	-34.9	14983
LOC100362830	85.9%	51.0%	-34.9	14984
Lap3	64.3%	29.4%	-34.9	14985
Vdac2	58.8%	23.9%	-34.9	14986
Pfn1	71.0%	36.1%	-34.9	14987
Sars	48.6%	13.7%	-35.0	14988
Ndufb8	65.1%	30.1%	-35.0	14989
Grina	55.3%	20.3%	-35.0	14990
Dmgdh	58.8%	23.8%	-35.0	14991
Atp5l	90.2%	55.2%	-35.0	14992
Gnas	98.0%	63.0%	-35.0	14993
Rps11	93.7%	58.7%	-35.1	14994
Ndufb10	58.8%	23.7%	-35.1	14995
Hnf4a	52.9%	17.8%	-35.1	14996
Fgl1	96.9%	61.7%	-35.2	14997
Tmsb4x	91.4%	56.2%	-35.2	14998
LOC103689947	51.8%	16.5%	-35.3	14999
Mpp1	49.0%	13.7%	-35.4	15000
Afmid	56.5%	21.1%	-35.4	15001
LOC103689992	89.4%	54.0%	-35.4	15002
Ndufv1	56.5%	21.0%	-35.4	15003
LOC100365958	47.8%	12.4%	-35.5	15004
Sec14l2	54.1%	18.5%	-35.6	15005
Rps9	87.5%	51.9%	-35.6	15006
Btf3	62.4%	26.8%	-35.6	15007
Hsp90aa1	73.3%	37.7%	-35.6	15008
Tmed2	68.2%	32.6%	-35.6	15009
Sc5d	67.1%	31.4%	-35.6	15010
Mat1a	100.0%	64.3%	-35.7	15011
Hsd17b6	70.2%	34.5%	-35.7	15012
Cyp4f6	57.6%	22.0%	-35.7	15013
Rpn1	57.3%	21.6%	-35.7	15014
Fbp1	98.8%	63.1%	-35.7	15015
Copz1	52.9%	17.2%	-35.8	15016
Plxnb2	46.7%	10.9%	-35.8	15017
Azgp1	100.0%	64.2%	-35.8	15018
Eef1g	70.2%	34.4%	-35.8	15019
LOC100910788	94.9%	59.1%	-35.8	15020
Urad	50.6%	14.7%	-35.9	15021
Agmat	49.4%	13.5%	-35.9	15022
Dad1	65.1%	29.2%	-35.9	15023
Trak2	47.8%	11.9%	-35.9	15024
Pkdcc	43.1%	7.2%	-35.9	15025
Selenbp1	50.2%	14.2%	-36.0	15026
Adk	82.7%	46.8%	-36.0	15027
Ctsh	61.6%	25.6%	-36.0	15028
Aup1	52.9%	16.9%	-36.0	15029
Asl	98.4%	62.4%	-36.0	15030
Smpdl3a	47.1%	11.0%	-36.0	15031
Aass	49.8%	13.7%	-36.1	15032
Npm1	70.2%	34.1%	-36.1	15033
Psma5	55.3%	19.2%	-36.1	15034
Acaa2	100.0%	63.9%	-36.1	15035
Cyb5a	100.0%	63.9%	-36.1	15036
Sec61a1	57.6%	21.5%	-36.1	15037
Amfr	51.4%	15.2%	-36.2	15038
Smlr1	55.3%	19.1%	-36.2	15039
LOC100362027	71.8%	35.6%	-36.2	15040
RGD1304704	50.6%	14.4%	-36.2	15041
C5	69.4%	33.2%	-36.2	15042

Mocs2	56.9%	20.6%	-36.2	15043
Cyp1a2	60.0%	23.7%	-36.3	15044
Taldo1	54.9%	18.6%	-36.3	15045
C4a	99.6%	63.3%	-36.3	15046
Cyp3a71-ps	36.5%	0.2%	-36.3	15047
LOC100361259	63.1%	26.8%	-36.4	15048
Ndufa4	89.8%	53.4%	-36.4	15049
Ctsb	85.1%	48.7%	-36.4	15050
Rps3	92.9%	56.4%	-36.5	15051
Tkt	75.3%	38.8%	-36.5	15052
Gsta2	96.9%	60.3%	-36.6	15053
Slco1a2	55.7%	19.0%	-36.7	15054
Ebpl	61.6%	24.8%	-36.8	15055
Cox7b	88.6%	51.8%	-36.8	15056
Edf1	62.4%	25.5%	-36.9	15057
Serinc1	56.9%	20.0%	-36.9	15058
Srxn1	43.5%	6.6%	-36.9	15059
Tspan31	59.2%	22.3%	-36.9	15060
Tbca	54.9%	18.0%	-36.9	15061
Nadk2	53.3%	16.3%	-37.0	15062
Rplp0	98.8%	61.8%	-37.0	15063
Slc25a47	89.8%	52.8%	-37.0	15064
Tsku	54.5%	17.4%	-37.1	15065
Vkorc1	81.6%	44.4%	-37.1	15066
Mut	52.9%	15.7%	-37.2	15067
Rpl22l1	56.9%	19.7%	-37.2	15068
Rpl18	94.1%	56.9%	-37.2	15069
Psmb7	68.6%	31.4%	-37.2	15070
LOC103692716	75.7%	38.4%	-37.2	15071
Rps27l	71.0%	33.7%	-37.2	15072
LOC100909524	78.4%	41.2%	-37.3	15073
Ccng1	48.6%	11.4%	-37.3	15074
Ugt2b1	47.1%	9.8%	-37.3	15075
Laptm4a	63.5%	26.2%	-37.3	15076
Scand1	71.8%	34.5%	-37.3	15077
Mcl1	53.7%	16.4%	-37.3	15078
Cyp8b1	62.0%	24.6%	-37.3	15079
LOC301124	69.4%	32.1%	-37.4	15080
Gcsh	63.9%	26.5%	-37.4	15081
RGD1307603	75.7%	38.3%	-37.4	15082
Rps27	67.1%	29.7%	-37.4	15083
Ndufc1	78.8%	41.4%	-37.4	15084
Ppa1	65.9%	28.4%	-37.5	15085
Rad23b	61.2%	23.7%	-37.5	15086
Aadat	54.9%	17.3%	-37.6	15087
Eif3i	49.8%	12.2%	-37.6	15088
Atp5j	70.6%	33.0%	-37.6	15089
Atox1	76.1%	38.4%	-37.6	15090
RGD1309350	73.3%	35.7%	-37.7	15091
Tsc22d1	69.0%	31.3%	-37.7	15092
Ndufb6	59.6%	21.9%	-37.7	15093
Bbox1	59.2%	21.5%	-37.7	15094
Adipor2	60.4%	22.6%	-37.8	15095
Prdx2	62.7%	24.9%	-37.8	15096
Oaf	65.1%	27.3%	-37.8	15097
Serbp1	65.1%	27.3%	-37.8	15098
Clta	58.4%	20.5%	-37.9	15099
Myl12a	54.1%	16.2%	-37.9	15100
LOC291863	58.0%	20.1%	-37.9	15101
Gnmt	97.6%	59.7%	-37.9	15102
Eif3c	53.7%	15.7%	-38.0	15103
Atp1b1	44.7%	6.7%	-38.0	15104
Maob	52.2%	14.1%	-38.0	15105
Ghr	76.9%	38.8%	-38.0	15106
Rpl3	96.5%	58.4%	-38.0	15107
Serpinf1	100.0%	61.9%	-38.1	15108

Sdc4	89.8%	51.6%	-38.2	15109
Gpld1	47.8%	9.7%	-38.2	15110
Ceacam1	51.0%	12.8%	-38.2	15111
Neurl3	67.1%	28.9%	-38.2	15112
Acadl	87.8%	49.6%	-38.2	15113
Hspd1	82.0%	43.7%	-38.2	15114
Pc	56.5%	18.2%	-38.2	15115
Abhd17a	76.5%	38.2%	-38.3	15116
Zfand2a	46.7%	8.4%	-38.3	15117
Aldh1l1	70.6%	32.3%	-38.3	15118
Psma4	62.0%	23.7%	-38.3	15119
Bdh1	80.0%	41.6%	-38.4	15120
Ndufa9	62.4%	24.0%	-38.4	15121
Cbs	55.3%	16.9%	-38.4	15122
LOC691309	65.1%	26.6%	-38.5	15123
Fdft1	64.7%	26.1%	-38.6	15124
Eci3	93.3%	54.8%	-38.6	15125
Rps25	91.8%	53.2%	-38.6	15126
Clpx	62.0%	23.3%	-38.6	15127
Ttc36	87.5%	48.8%	-38.7	15128
Cyp1a1	40.4%	1.7%	-38.7	15129
Bsg	93.7%	55.0%	-38.7	15130
Masp2	72.5%	33.8%	-38.7	15131
LOC108348852	72.2%	33.4%	-38.7	15132
Atp5d	71.0%	32.2%	-38.8	15133
Prodh2	57.6%	18.9%	-38.8	15134
Hsp90b1	69.4%	30.6%	-38.8	15135
P4hb	98.4%	59.6%	-38.8	15136
Mpeg1	56.5%	17.7%	-38.8	15137
Depdc7	58.4%	19.6%	-38.8	15138
Cfh	98.8%	60.0%	-38.9	15139
Hist1h2bh	77.3%	38.4%	-38.9	15140
Pebp1	97.3%	58.4%	-38.9	15141
RGD735065	63.9%	25.0%	-38.9	15142
Krt8	96.5%	57.6%	-38.9	15143
Pygl	63.1%	24.2%	-38.9	15144
Sdhb	78.0%	39.1%	-39.0	15145
LOC100360095	98.4%	59.5%	-39.0	15146
Serpina10	84.7%	45.7%	-39.0	15147
Tmem30a	61.6%	22.5%	-39.0	15148
Srebf1	52.9%	13.8%	-39.1	15149
Baat	77.3%	38.1%	-39.1	15150
Aldh9a1	91.0%	51.8%	-39.2	15151
Ttpa	91.0%	51.7%	-39.3	15152
Rpl27	86.7%	47.4%	-39.3	15153
LOC100910877	98.0%	58.8%	-39.3	15154
Sdha	59.2%	19.9%	-39.3	15155
Ndrg2	96.1%	56.8%	-39.3	15156
Pcyt2	57.3%	17.9%	-39.3	15157
Mapkapk2	48.6%	9.3%	-39.4	15158
LOC108352909	83.1%	43.7%	-39.4	15159
Mpc1	91.0%	51.6%	-39.4	15160
Vdac3	65.5%	26.1%	-39.4	15161
Slc2a2	68.6%	29.2%	-39.5	15162
LOC688869	75.7%	36.2%	-39.5	15163
Herpud1	66.3%	26.8%	-39.5	15164
Mgst2	65.5%	26.0%	-39.5	15165
As3mt	58.0%	18.5%	-39.6	15166
Uqcrb	74.5%	34.9%	-39.6	15167
Dcaf11	56.5%	16.9%	-39.6	15168
Eif3g	58.8%	19.2%	-39.6	15169
Cyp2d2	100.0%	60.4%	-39.6	15170
Uqcrh	66.7%	27.0%	-39.6	15171
Fmo3	74.9%	35.3%	-39.7	15172
Psmb4	87.1%	47.4%	-39.7	15173
Eif4g2	78.4%	38.7%	-39.7	15174

F13b	60.8%	20.9%	-39.8	15175
Hspa4	52.2%	12.3%	-39.8	15176
Atp5h	93.3%	53.5%	-39.9	15177
Cox5b	83.9%	44.0%	-39.9	15178
C8a	81.6%	41.6%	-39.9	15179
LOC683212	92.9%	52.9%	-40.0	15180
Ran	65.9%	25.8%	-40.1	15181
C2	52.9%	12.9%	-40.1	15182
Tmem86b	67.8%	27.7%	-40.1	15183
Glyat	80.0%	39.9%	-40.1	15184
LOC100363452	83.9%	43.8%	-40.1	15185
Csnk2b	56.5%	16.3%	-40.2	15186
Dnajc22	60.4%	20.1%	-40.2	15187
Aspdh	59.6%	19.3%	-40.3	15188
LOC100911483	79.6%	39.3%	-40.3	15189
LOC100911417	78.8%	38.5%	-40.3	15190
Lipa	65.5%	25.2%	-40.3	15191
Carhsp1	67.5%	27.1%	-40.4	15192
Asgr1	88.6%	48.2%	-40.4	15193
Mtdh	55.3%	14.9%	-40.4	15194
Rpl6	96.9%	56.4%	-40.4	15195
Psme1	71.8%	31.3%	-40.4	15196
Rps8	87.1%	46.6%	-40.5	15197
Tgfbi	62.0%	21.5%	-40.5	15198
Cox7c	91.0%	50.5%	-40.5	15199
Slc20a1	52.5%	12.1%	-40.5	15200
Txnl1	62.4%	21.8%	-40.5	15201
Igfbp4	94.9%	54.4%	-40.5	15202
Hgd	76.5%	35.9%	-40.6	15203
LOC689574	67.8%	27.3%	-40.6	15204
Hyi	64.7%	24.1%	-40.6	15205
Serping1	100.0%	59.3%	-40.7	15206
Serf2	87.5%	46.7%	-40.8	15207
LOC100911238	82.7%	42.0%	-40.8	15208
Cdc42	65.9%	25.1%	-40.8	15209
RT1-S3	77.6%	36.9%	-40.8	15210
Ndufa6	84.7%	43.9%	-40.8	15211
Pon3	82.7%	41.9%	-40.9	15212
Mettl7b	93.7%	52.8%	-40.9	15213
Agt	100.0%	59.1%	-40.9	15214
Eprs	60.4%	19.4%	-41.0	15215
Atp5f1	84.3%	43.3%	-41.0	15216
Nxpe4	56.9%	15.8%	-41.0	15217
Hibadh	65.1%	24.1%	-41.0	15218
Rack1	94.1%	53.1%	-41.0	15219
Ufc1	59.6%	18.5%	-41.1	15220
Por	51.4%	10.2%	-41.1	15221
Ltbr	53.7%	12.5%	-41.2	15222
Cisd1	65.1%	23.8%	-41.3	15223
Atp6v0c	87.5%	46.1%	-41.3	15224
Pcbd1	78.0%	36.7%	-41.3	15225
Mcfd2	77.3%	35.9%	-41.4	15226
Rgn	89.0%	47.6%	-41.4	15227
Gabarap	79.2%	37.7%	-41.5	15228
Ctsl	99.2%	57.7%	-41.5	15229
Pah	99.2%	57.7%	-41.5	15230
LOC100363537	75.7%	34.1%	-41.6	15231
Psma7	74.1%	32.5%	-41.6	15232
Aox1	65.1%	23.5%	-41.6	15233
Sephs2	59.6%	18.0%	-41.6	15234
Pon2	66.7%	25.0%	-41.6	15235
LOC100909835	74.5%	32.9%	-41.7	15236
Acsl1	96.9%	55.2%	-41.7	15237
Inhbc	50.2%	8.5%	-41.7	15238
Serpina6	98.0%	56.3%	-41.8	15239
Dstnl1	72.9%	31.2%	-41.8	15240

Otc	72.2%	30.3%	-41.9	15241
Pbld1	64.3%	22.4%	-41.9	15242
Pros1	66.3%	24.3%	-42.0	15243
Ebp	82.7%	40.8%	-42.0	15244
Idh1	76.9%	34.9%	-42.0	15245
Klkb1	58.8%	16.8%	-42.0	15246
Chchd10	98.8%	56.8%	-42.1	15247
Rac1	65.1%	23.0%	-42.1	15248
Nfe2l2	63.1%	21.0%	-42.1	15249
LOC108349606	87.8%	45.7%	-42.1	15250
Psmb5	72.2%	30.0%	-42.2	15251
Itm2b	99.6%	57.4%	-42.2	15252
Atp5g1	94.5%	52.3%	-42.2	15253
Ube2d3	81.6%	39.3%	-42.2	15254
Wbp11	66.3%	24.0%	-42.3	15255
LOC100361907	94.1%	51.8%	-42.3	15256
Pecr	97.6%	55.3%	-42.3	15257
Kyat3	60.0%	17.7%	-42.3	15258
Eif4h	59.2%	16.9%	-42.3	15259
Ces1c	87.5%	45.1%	-42.4	15260
Skp1	78.0%	35.7%	-42.4	15261
Hadhb	61.2%	18.7%	-42.5	15262
Gulo	71.0%	28.5%	-42.5	15263
Cyc1	69.8%	27.3%	-42.5	15264
Map1lc3b	78.0%	35.3%	-42.7	15265
Rpn2	67.8%	25.1%	-42.7	15266
Fah	97.6%	54.8%	-42.8	15267
Aldh7a1	92.2%	49.3%	-42.8	15268
Cxcl11	67.1%	24.2%	-42.8	15269
Mdh1	91.8%	48.9%	-42.8	15270
Il6st	60.4%	17.5%	-42.9	15271
Ndufs3	59.6%	16.7%	-42.9	15272
Hoga1	79.2%	36.3%	-42.9	15273
Sdhd	70.2%	27.3%	-42.9	15274
Cfi	99.2%	56.3%	-42.9	15275
Suclg1	89.8%	46.8%	-43.0	15276
Tmem59	74.5%	31.5%	-43.0	15277
Cd81	88.6%	45.6%	-43.1	15278
Atp1a1	59.2%	16.1%	-43.1	15279
Gstt3	63.1%	20.1%	-43.1	15280
Psap	79.6%	36.5%	-43.1	15281
Tnfaip2	58.4%	15.3%	-43.1	15282
Atp5c1	94.9%	51.8%	-43.1	15283
Cps1	99.6%	56.4%	-43.2	15284
Rpl7a	98.8%	55.6%	-43.3	15285
Etfb	94.5%	51.2%	-43.3	15286
Serp1	77.3%	34.0%	-43.3	15287
Ctsa	65.1%	21.7%	-43.4	15288
Hsd17b13	69.0%	25.7%	-43.4	15289
Wdr12	98.0%	54.4%	-43.7	15290
Qdpr	83.9%	40.2%	-43.7	15291
Amacr	67.1%	23.3%	-43.7	15292
Cxcl1	76.5%	32.7%	-43.8	15293
Ninj1	73.3%	29.5%	-43.8	15294
Rcl1	76.1%	32.2%	-43.9	15295
Rpl18a	92.5%	48.7%	-43.9	15296
Dpys	64.3%	20.4%	-43.9	15297
Tmem205	100.0%	56.0%	-44.0	15298
Mdh2	86.7%	42.7%	-44.0	15299
Pemt	90.2%	46.2%	-44.0	15300
Adi1	76.5%	32.5%	-44.0	15301
Cfhr1	99.6%	55.6%	-44.1	15302
Cyp2b3	98.4%	54.4%	-44.1	15303
Hint1	79.6%	35.4%	-44.2	15304
Actn4	57.3%	13.0%	-44.2	15305
Acox3	66.3%	22.0%	-44.3	15306

Nudt4	74.5%	30.2%	-44.3	15307
Cald1	77.6%	33.3%	-44.3	15308
LOC102551552	61.2%	16.8%	-44.4	15309
Khk	76.1%	31.7%	-44.4	15310
Uqcrc2	67.5%	23.0%	-44.4	15311
Adhfe1	66.3%	21.8%	-44.5	15312
Atp5o	91.0%	46.5%	-44.5	15313
Fads1	77.6%	33.2%	-44.5	15314
Ssr4	72.9%	28.5%	-44.5	15315
Cyb5r3	80.0%	35.5%	-44.5	15316
Cd164	98.0%	53.5%	-44.6	15317
Uqcrc1	91.4%	46.8%	-44.6	15318
Sdc1	70.2%	25.6%	-44.6	15319
Timd2	93.3%	48.7%	-44.7	15320
Tm4sf4	76.5%	31.8%	-44.7	15321
Hsd17b11	94.5%	49.8%	-44.7	15322
LOC103694872	98.0%	53.3%	-44.7	15323
Acsl5	75.7%	30.9%	-44.8	15324
Osgin1	95.7%	50.9%	-44.8	15325
Ech1	65.5%	20.7%	-44.8	15326
Mst1	59.2%	14.3%	-44.9	15327
Eef1b2	89.4%	44.4%	-45.0	15328
Litaf	67.8%	22.8%	-45.1	15329
Afm	99.2%	54.1%	-45.1	15330
Psmd8	71.0%	25.8%	-45.2	15331
Itih1	96.9%	51.6%	-45.3	15332
Sdf4	69.8%	24.4%	-45.4	15333
Prdx1	97.6%	52.1%	-45.5	15334
Uroc1	64.3%	18.8%	-45.5	15335
Gk	72.5%	27.0%	-45.5	15336
Grhpr	98.8%	53.2%	-45.6	15337
Decr2	62.7%	17.1%	-45.6	15338
Myl6	94.9%	49.2%	-45.7	15339
Hdlbp	72.9%	27.3%	-45.7	15340
Tymp	73.3%	27.5%	-45.8	15341
Ndufab1	72.2%	26.3%	-45.9	15342
Sod2	83.5%	37.6%	-45.9	15343
Abhd14b	74.9%	29.0%	-45.9	15344
Angptl3	99.2%	53.2%	-46.0	15345
Calm1	80.4%	34.4%	-46.0	15346
Ppib	83.9%	37.9%	-46.0	15347
Txndc17	66.7%	20.6%	-46.0	15348
Pdia6	72.5%	26.5%	-46.1	15349
Canx	76.9%	30.7%	-46.2	15350
Hpn	72.5%	26.4%	-46.2	15351
Naca	80.4%	34.2%	-46.2	15352
Hgfac	69.8%	23.6%	-46.2	15353
LOC100363502	86.7%	40.4%	-46.3	15354
Tst	82.7%	36.4%	-46.4	15355
Plin2	72.5%	26.1%	-46.4	15356
Cnbp	76.1%	29.7%	-46.4	15357
Ddt	96.9%	50.4%	-46.4	15358
Hsp90ab1	91.4%	44.9%	-46.4	15359
Ghitm	87.1%	40.6%	-46.5	15360
Insig1	90.6%	44.1%	-46.5	15361
Grpel1	76.5%	30.0%	-46.5	15362
Slc22a18	74.1%	27.6%	-46.5	15363
Ndufb9	94.1%	47.6%	-46.6	15364
Tdo2	98.0%	51.5%	-46.6	15365
Serpind1	100.0%	53.4%	-46.6	15366
Wfdc2	64.3%	17.7%	-46.6	15367
Slc26a1	62.7%	16.1%	-46.7	15368
Ang	97.3%	50.4%	-46.8	15369
Ndufs2	70.6%	23.7%	-46.8	15370
Rpl15	91.4%	44.5%	-46.8	15371
Pgrmc1	100.0%	53.1%	-46.9	15372

Prdx5	94.5%	47.6%	-46.9	15373
F12	98.4%	51.5%	-47.0	15374
Acsm1	80.4%	33.3%	-47.1	15375
Ugt2b35	96.9%	49.8%	-47.1	15376
Cpb2	96.9%	49.8%	-47.1	15377
Glud1	95.7%	48.6%	-47.1	15378
Tfr2	62.0%	14.9%	-47.1	15379
Dpt	85.1%	38.0%	-47.1	15380
Hsd11b1	99.2%	52.0%	-47.2	15381
Tram1	80.0%	32.8%	-47.2	15382
Agxt	75.7%	28.3%	-47.4	15383
Lcat	96.1%	48.7%	-47.4	15384
Cyp2j3	71.8%	24.3%	-47.5	15385
F10	94.9%	47.4%	-47.5	15386
Sdhc	69.4%	21.9%	-47.5	15387
Cmbl	82.4%	34.8%	-47.6	15388
C1s	82.0%	34.4%	-47.6	15389
Tmem176a	76.1%	28.5%	-47.6	15390
Pxmp2	92.5%	44.9%	-47.6	15391
Alad	83.1%	35.5%	-47.6	15392
Nme2	82.0%	34.3%	-47.7	15393
Gcdh	72.9%	25.3%	-47.7	15394
Sord	87.8%	39.9%	-48.0	15395
Nit1	67.5%	19.3%	-48.1	15396
Psmb1	78.4%	30.3%	-48.1	15397
Tmprss6	70.6%	22.4%	-48.2	15398
Apom	94.5%	46.3%	-48.2	15399
Bhmt2	87.8%	39.6%	-48.3	15400
F9	83.5%	35.1%	-48.4	15401
Esd	73.3%	24.9%	-48.5	15402
Phyh	100.0%	51.5%	-48.5	15403
Cox6a1	82.4%	33.7%	-48.6	15404
Ndfip1	82.0%	33.3%	-48.6	15405
Zfand5	67.5%	18.8%	-48.7	15406
Pgk1	86.3%	37.6%	-48.7	15407
Atp5a1	96.9%	48.1%	-48.7	15408
Mtch2	83.9%	35.2%	-48.7	15409
Tmbim6	98.0%	49.2%	-48.8	15410
Prdx4	80.4%	31.6%	-48.8	15411
C8b	89.4%	40.5%	-48.9	15412
Fh	76.5%	27.6%	-48.9	15413
Akr1d1	96.1%	47.2%	-48.9	15414
Adtrp	72.2%	23.2%	-49.0	15415
Sigmar1	76.1%	27.1%	-49.0	15416
Dstn	87.5%	38.4%	-49.1	15417
Mbl1	75.7%	26.5%	-49.1	15418
LOC259244	50.2%	1.0%	-49.2	15419
Aars	67.8%	18.6%	-49.2	15420
Gstk1	82.7%	33.5%	-49.3	15421
Ndufb11	76.1%	26.8%	-49.3	15422
Pnp	78.4%	29.1%	-49.3	15423
Gde1	69.8%	20.3%	-49.5	15424
Gpd1	96.5%	46.9%	-49.5	15425
Spint2	78.0%	28.4%	-49.7	15426
Amy1a	94.9%	45.1%	-49.8	15427
Cfhr2	73.7%	23.9%	-49.8	15428
Apon	62.0%	12.1%	-49.9	15429
Ptms	96.9%	46.8%	-50.0	15430
Lipc	96.1%	46.0%	-50.1	15431
Eci2	87.1%	36.9%	-50.2	15432
Itih2	97.3%	46.9%	-50.3	15433
Igfals	75.7%	25.3%	-50.3	15434
Sdc2	99.6%	49.2%	-50.4	15435
LOC100910235	74.5%	24.1%	-50.4	15436
Hspa5	94.9%	44.4%	-50.5	15437
F11	69.0%	18.5%	-50.5	15438

Proc	97.6%	47.1%	-50.6	15439
Tubb4b	78.4%	27.8%	-50.6	15440
Serpinf2	85.1%	34.5%	-50.6	15441
Gabarapl1	78.4%	27.7%	-50.8	15442
Kynu	72.9%	22.1%	-50.8	15443
Mpc2	92.2%	41.2%	-50.9	15444
Hagh	70.6%	19.7%	-50.9	15445
Iscu	82.4%	31.3%	-51.0	15446
Smpd1	69.8%	18.7%	-51.1	15447
Ugt2b37	78.0%	26.9%	-51.1	15448
Lamp2	85.1%	33.9%	-51.2	15449
Cideb	75.7%	24.5%	-51.2	15450
Clu	99.6%	48.4%	-51.2	15451
Dcxr	73.7%	22.5%	-51.3	15452
Dio1	64.7%	13.4%	-51.3	15453
Ndufv2	82.0%	30.6%	-51.3	15454
Aldh3a2	88.6%	37.3%	-51.4	15455
Gpt	65.9%	14.5%	-51.4	15456
Dhrs7	84.3%	32.9%	-51.5	15457
Decr1	74.1%	22.6%	-51.5	15458
Retsat	74.1%	22.6%	-51.5	15459
Rps15	86.3%	34.8%	-51.5	15460
Sult1c3	77.6%	25.9%	-51.7	15461
Slc27a2	93.7%	42.0%	-51.8	15462
LOC108353758	97.6%	45.8%	-51.8	15463
Creb3l3	93.3%	41.3%	-52.0	15464
Car8	61.2%	9.1%	-52.1	15465
Gltpd2	81.6%	29.5%	-52.1	15466
Slc37a4	70.2%	18.0%	-52.2	15467
C8g	89.0%	36.8%	-52.2	15468
Lect2	65.5%	13.2%	-52.3	15469
Ftcd	91.4%	39.0%	-52.4	15470
Elovl5	85.5%	33.0%	-52.5	15471
Akr1a1	80.8%	28.3%	-52.5	15472
Acadvl	78.8%	26.2%	-52.6	15473
Hsd17b4	85.5%	32.4%	-53.1	15474
Gch1	68.6%	15.3%	-53.3	15475
Akr1c13	97.6%	44.3%	-53.4	15476
Cyp4f1	88.2%	34.9%	-53.4	15477
Fn1	96.5%	43.0%	-53.5	15478
Atp5g3	98.4%	44.9%	-53.5	15479
Aldh6a1	87.8%	34.3%	-53.6	15480
Slc25a3	90.6%	37.0%	-53.6	15481
Lonp2	86.7%	33.0%	-53.7	15482
Insig2	96.1%	42.2%	-53.9	15483
Mgst3	78.4%	24.4%	-54.1	15484
Tmem176b	88.2%	34.1%	-54.1	15485
Pttg1ip	89.8%	35.7%	-54.2	15486
Apof	92.9%	38.4%	-54.6	15487
Hadh	86.7%	32.0%	-54.7	15488
Asgr2	81.6%	26.9%	-54.7	15489
RGD1307752	80.4%	25.6%	-54.8	15490
Slc10a1	94.9%	39.7%	-55.2	15491
Vcp	85.9%	30.4%	-55.5	15492
Rpl11	92.9%	37.4%	-55.5	15493
Abcc2	69.8%	14.2%	-55.6	15494
Sult1e1	55.7%	0.1%	-55.6	15495
Tmem14c	87.5%	31.8%	-55.6	15496
Cyp3a23/3a1	74.9%	19.3%	-55.6	15497
Slc38a3	94.1%	38.4%	-55.7	15498
Ust5r	57.3%	1.4%	-55.8	15499
LOC259246	100.0%	44.0%	-56.0	15500
Acadm	94.5%	38.5%	-56.0	15501
Slc25a15	83.5%	27.3%	-56.2	15502
Akr1c2	98.4%	42.0%	-56.4	15503
Ces1f	85.5%	29.1%	-56.4	15504

Ahcy	98.4%	42.0%	-56.5	15505
Eif5	91.0%	34.1%	-56.8	15506
Gls2	76.5%	19.5%	-57.0	15507
Cpq	67.1%	9.9%	-57.1	15508
Cd302	98.8%	41.6%	-57.2	15509
Sar1b	87.5%	30.2%	-57.2	15510
Rcan1	89.8%	32.5%	-57.3	15511
Tmem150a	78.0%	20.7%	-57.3	15512
Hsd17b10	85.9%	27.9%	-58.0	15513
Cyp27a1	91.0%	32.8%	-58.2	15514
Cyp2d1	90.6%	32.4%	-58.2	15515
Tmem37	79.2%	20.7%	-58.5	15516
Slc38a4	86.7%	28.1%	-58.5	15517
Col18a1	80.8%	22.2%	-58.6	15518
Tnfrsf12a	82.4%	23.7%	-58.6	15519
Cers2	85.5%	26.5%	-59.0	15520
Aldh4a1	82.7%	23.7%	-59.1	15521
LOC500300	78.8%	19.4%	-59.4	15522
Ctsc	79.2%	19.6%	-59.6	15523
Cyp2d3	92.2%	32.5%	-59.7	15524
LOC100912565	61.2%	1.0%	-60.2	15525
LOC100912026	84.3%	23.7%	-60.7	15526
Ces1e	77.3%	16.2%	-61.0	15527
Eci1	91.4%	30.3%	-61.1	15528
Creg1	81.2%	19.8%	-61.4	15529
Sept14	62.0%	0.0%	-62.0	15530
Cyp2d5	89.0%	26.9%	-62.1	15531
Sat1	92.5%	30.2%	-62.3	15532
LOC100912818	65.9%	2.2%	-63.7	15533
ste2	64.7%	0.0%	-64.7	15534
Cyp4a3	98.8%	33.6%	-65.3	15535
Ces1d	95.7%	30.1%	-65.6	15536
Agmo	88.2%	22.3%	-65.9	15537
Stac3	69.0%	2.6%	-66.4	15538
LOC100912405	96.5%	27.4%	-69.1	15539
Spink1	86.3%	16.0%	-70.3	15540
Cdh17	72.9%	2.5%	-70.5	15541
LOC108348209	73.3%	2.8%	-70.5	15542
Cyp3a2	74.1%	3.0%	-71.1	15543
Cyp4a2	99.2%	26.7%	-72.5	15544
Cyp3a18	98.4%	23.8%	-74.6	15545
LOC298116	100.0%	22.3%	-77.7	15546
Mup5	82.4%	2.0%	-80.4	15547
Car3	93.3%	9.0%	-84.3	15548
28-Feb	84.7%	0.0%	-84.7	15549
LOC681426	89.8%	1.8%	-88.0	15550
Cyp2c13	98.8%	10.1%	-88.8	15551
Hao2	92.9%	3.7%	-89.3	15552
Cyp2a2	99.2%	9.0%	-90.3	15553
RGD1566134	98.4%	7.4%	-91.1	15554
LOC103695259	98.4%	4.5%	-94.0	15555
Obp3	99.6%	4.7%	-94.9	15556
Cyp2c11	99.2%	1.1%	-98.1	15557

Supplemental Table S2. Primer seqences used on RT-qPCR

Gene	Forward primer (5'-3')	Reverse primer (5'-3')
Trex1	GAGATGGCCCCCATGGTTC	AGTGTTCTCCAGAGCACGTC
Zfp518a	AATGGGGACCACGGCAGATA	AGTATCAACTTTCTCCCAGGAACA
Taf10	AGCAAGAGCAAGGATCGCAA	TGGCTCAGGTGAAGTAGTGC
Spns1	TGGTCTAATCTCTGACCGCC	GAAAGCACAGAGCATGAGCG
Rnf126	ACCATCATCACGCAGCTCCTC	AGCCCACGTGTTCCTCTGTG
Ilk	AAACAGACGCTCAGCAGACA	CAGTGCCACCTTCATCCCAA
Bop1	GCTGTACATCCAGCAGGTGA	GCCTCAGCACTTTGTATGGC
Eif3l	GACTACGAGTCTGAGGCTGC	GTCCTGCTTCGGATCTCCTG
Igbp1	ACTCTCCAAGAGAGGAAACAGC	GCCAGGCTTGGATAACCAGT
Ptdss1	CACACAGTGCAAGCGTGTAG	ACCATGCCGTACAGACACAG
Psmf1	CTCTGTGGAGAACGGCATGA	ATAGGTCCTGTGGAAGTCACTC
Timm8a1	GGAACTTTGCTGGGAGAAGTG	AAGGCTTTCTGAGAAGACGGG
Rnf10	CTCTCTGAAGACAGAGGAGGGAA	AGTGTCACTTGGTGTGGACG
Vamp2	CTGCACCTCCTCCAAACCTTA	TCATGATGTCCACCACCTCATC
Wdr45	GGAGCAAGATCCAAGGGAAGG	ATTGTAGATCCGGACGCCTG
Shq1	TGGATTCAGAAGGCCAAGTCC	CTGGAGTGTGGAGCCTGATG
Map2k5	ACTCAGTGCATGAGGAAGCAG	TCTCCTCTAGAGCTCGGCAC
Klhdc2	TCTGGATACGTGGGAGTGGA	TCCAGGCATCACTTAGTGGC
Pcyox1l	CATGCAGGACTTCGTCAAGC	AGCAGGTACCAGTCGGTTTC
Pelp1	CTGCCTTCTCTGCACCTTGA	TGGTCCGAATGGTGCTGTAA
Zfp868	ACTGAGGAAAGCCGTTGTGG	AGTTCCTGGCAGACCATTGC
Orc4	AGGTGGTGAACATCCTTCGG	TTCAAATCCTCTCCCAGTCAAGC
Fam160b2	AGCTCCTGCATGTATCTGAGC	GCTGGTCAGTGCCTAAGAGG
Capn1	CTGAGCTGCCACCAAGGAAG	AACTCCTCTGTCATCCTGGGGA
Klhl12	TGTCTAGGAGGATATGATGGCTTG	TCAGTAGGGCTACTCCAGCA
Sharpin	AATGTCCAGCTCATGGAGGC	CCGAACTCTGAGAACACCTGA
Rfc4	GTGACGACTGAGAAGAAGCCA	CGTCCTGGAAAGCCACTTCA
Itgb3bp	TTGGTGTTTCCCTAGTACCATGC	GTCAGACTTGAAGGTAACACTCAG
Psmd9	TGCTGGGCTGCAACATTATTC	AAAGCGTCTCTTCCACCTCG
Drg1	AGGCATTAACCTCACAGCCA	ATAAACTCTGTTCCCTTCGACCA
Med22	AGCAGCCAACATCGTTCGAG	GGCTTCATTCACAGACGGGA
Arl5b	TGACAGCAGCTGAAATCTCCA	ACCTTGGCACAACCCTTCTC
Smg9	TACCTGCTGGATCAGACGGA	ACATACGCTCTCTGGTCTTCC
Nmi	GCCAGGTTAGTGTTTTCGAGG	CTACAGAACTCAGCACCCGC
Taf1c	GTTGAGGTTCGGCACTCTGG	AGGTCGGATCCTTGCAGTTC
Tmco4	TGTACCGCACATCTTCCGTG	ATGGCATCCATTTGCTTGGC
Akap8l	GCTACACAGGTTTTGTCCAAGG	AGAGTGTGAAGTGGCCATACC
Sertad1	AAGCTAGCGATGCTACGGC	ACCTTTGCTCAGCATCCTACAG
Slc25a40	GCCTCCCTCCTACTCTAGTGA	CACCAAATCTGGCGACAACC
Syt5	TATCTGCTGCCAGACAAGCG	TCATACACCGCCATGACCAG
Phlda3	ACATGTCAGCTTCTCTGTCCAC	CCCTACTGGCTTCTGCTCTC
Clec12a	CAGCCCTCTCACCTTTTTCG	GCATCGGCGGATACTTTTCCC
Glipr1	ATGCGACTATGGACCAGCAG	CAGAGTAGTAACGTGAGACCTGG
Cxcl1	CTGGGATTCACCTCAAGAACATC	CAGGGTCAAGGCAAGCCTC
Cxcl2	CCAACCACCAGGCTACAGG	GCGTCACACTCAAGCTCTG
Ccl2	GCTACTCATTCACCAGCAAGAT	GGTGCTGAAGACCTTAGGGC
Akt1	ATGAACGACGTAGCCATTGTG	TTGTAGCCAATAAAGGTGCCAT
Akt2	ACGTGGTGAATACATCAAGACC	GCTACAGAGAAATTGTTCAGGGG
p16Ink4a	CCCAACGCCCCGAACT	GCAGAAGAGCTGCTACGTGAA
Mmp3	ACATGGAGACTTTGTCCCTTTTG	TTGGCTGAGTGGTAGAGTCCC
Timp1	GCAACTCGGACCTGGTCATAA	CGGCCCGTGATGAGAAACT
Timp2	TCAGAGCCAAAGCAGTGAGC	GCCGTGTAGATAAACTCGATGTC
Cebpb	GTTTCGGGACTTGATGCAAT	CCCGCAGGAACATCTTTAAG
Dcr2	AGCTAACCCAGCCCATAATCGTC	AGTTCCCTTCTGACAGGTACTGGC
Cxcl5	TGCGTTGTGTTTGCTTAACCG	AGCTATGACTTCCACCGTAGG

Nfkb1	GACGCCATCTATGATAGCAAAGC	ACACATCCTGCTGTTCTGTC
Braf	AGCACTGATGATGAGAGGTC	GAAACCCTGGAAAAGCAGC
Il6	TGTTCTCTGGGAAATCGTGGA	TGCAAGTGCATCATCGTTGTTC
IL-8 (CXCR2)	AAGGGTGGGGAGTTCGTGTA	AGGGAGCAATACTCAGCTTTCA
Ccl8	TCTACGCAGTGCTTCTTTGCC	AAGGGGGATCTTCAGCTTTAGTA
Il7	GATAGTAATTGCCCGAATAATGAACCA	GTTTGTGTGCCTTGTGATACTGTTAG
Mmp12	CCAAGCATCCCATCTGGTAT	GGTCAAAGACAGCTGCATCA
ARF(p19)	TGATGTTTGGAAGTCCAGCAG	AATGTCCATGAGGTTCTGAGC
Serpine1	TTCAGCCCTTGCTTGCCTC	ACACTTTTACTCCGAAGTCGGT
Cdkn1a	ATGTCCAATCCTGGTGATGTC	TCTCTTGCAGAAGACCAATC
Dec1	GGCGGGGAATAAAACGGAGCGA	CCTCACGGGCACAAGTCTGGAA
Bcl2	GCTACCGTCGTGACTTCGC	CCCCACCGAACTCAAAGAAGG
Bag3	AGAAGCGGCACACCGGTTCAC	ACTTTCTGGTTTGTTTTCGGG
Pten	TCTGCAGGAAATCCCATAGC	TCGTTAGCAGAAACAAAAGGA
Pik3ca	CCACGACCATCTTCGGGTGAA	CCTCACGGAGGCATTCTAAAGT
Hras	CTGACACCAGGCTCAGGAC	TCCATGCGAAGGTCTTGGTC
Skp2	CTAGTTCGCGGGTCCAACTG	TTAATTGAAGCGCTGCCTGC
Rb1	AACCCAGCAGTGCGTTATCT	GGGTGTTCGAGGTGAACCAT
Ifng	CCACGGCACAGTCATTGAAA	CTGCAGGATTTTCATGTCACCA
Mmp9	GTCCAGACCAAGGGTACAGC	ATACAGCGGGTACATGAGCG
Nfkb2	CGCCTCTCTTCACCTTAGGC	TCACCAGATAGGGGCCATCA
Trp53	CTCTCCCCCGCAAAAGAAAAA	CGGAACATCTCGAAGCGTTTA
Foxo4	ACGAGTGGATGGTCCGTACT	GTGGCGGATCGAGTTCTTC
Lmnb1	CAGATTGCCCAGCTAGAAGC	CATTGATCTCCTCTTCATAC
Myc	CGCCCAGTGAGGATATCTGG	CGACCGCAACATAGGATGGA
Csnk1a1	ATGGGTATTGGGCGTCACTG	GCATTGATGCTGGCGTATCG
Gsk3b	CGGCGTCTCCTCATTGGTTA	TTACAGGCGTCTGGGAATCG
Map3k5	ACACAGCTGATCCGGAAGAC	GACACAGTGGAGCTTAGCGT
Il1b	CTGGAGAGTGTGGATCCCAAG	GGAAGACACGGATTCCATGGTG
Ctnn1b	TGAAGGCGTGGCAACATACG	GCCATTGGCTCTGTCCTGAA
Tnf	TATGGCCCAGACCCTCACA	GGAGTAGACAAGGTACAACCCATC
